# Multidimensional Gene Set Analysis of Genomic Data

**DOI:** 10.1371/journal.pone.0010348

**Published:** 2010-04-27

**Authors:** David Montaner, Joaquín Dopazo

**Affiliations:** 1 Department of Bioinformatics and Genomics, Centro de Investigación Príncipe Felipe (CIPF), Valencia, Spain; 2 Functional Genomics Node (INB), Centro de Investigación Príncipe Felipe (CIPF), Valencia, Spain; 3 CIBER de Enfermedades Raras (CIBERER), Valencia, Spain; Deutsches Krebsforschungszentrum, Germany

## Abstract

Understanding the functional implications of changes in gene expression, mutations, etc., is the aim of most genomic experiments. To achieve this, several functional profiling methods have been proposed. Such methods study the behaviour of different gene modules (e.g. gene ontology terms) in response to one particular variable (e.g. differential gene expression). In spite to the wealth of information provided by functional profiling methods, a common limitation to all of them is their inherent unidimensional nature. In order to overcome this restriction we present a multidimensional logistic model that allows studying the relationship of gene modules with different genome-scale measurements (e.g. differential expression, genotyping association, methylation, copy number alterations, heterozygosity, etc.) simultaneously. Moreover, the relationship of such functional modules with the interactions among the variables can also be studied, which produces novel results impossible to be derived from the conventional unidimensional functional profiling methods. We report sound results of gene sets associations that remained undetected by the conventional one-dimensional gene set analysis in several examples. Our findings demonstrate the potential of the proposed approach for the discovery of new cell functionalities with complex dependences on more than one variable.

## Introduction

The development of new genomic technologies, such as microarrays of gene expression, genotyping or array-CGH, along with the new next-generation sequencing techniques is increasing the volume of data throughput amazingly. As a direct consequence of this, the bottleneck in functional genomics has shifted from the data production phase to the data analysis steps. In particular, the necessity for providing a functional interpretation at molecular level that accounts for the genome-scale experimental designs has promoted the development of different methods for the functional analysis of this type of data in the last years [1], [2].

It is widely accepted that most of the biological functionality of the cell arises from complex interactions among their molecular components that define operational interacting entities or modules [3]. Functions collectively performed by such modules have conceptually been represented in different ways. Gene ontology (GO) [4] and KEGG pathways [5] are the most popular and widely used module definitions although many other are available in different repositories (e.g., Reactome [6], Biocarta, etc.) For practical purposes, functional modules are henceforth defined as sets of genes sharing functional annotations extracted from any of these repositories. Functional profiling methods exploit different definitions of modules in an attempt of understanding the functional basis of high-throughput experimental results [7]. Initially, functional enrichment methods, in different implementations [7], [8], have been used for this purpose. More sensitive approaches, generically known as gene-set analysis (GSA) methods, pioneered by the Gene Set Enrichment Analysis (GSEA) [9], were later proposed [1], [10]. In the original formulation, GSA methods aimed to directly detect sets of functionally related genes (modules) with a coordinate and significant over- or under-expression across the complete list of genes ranked according to their differential expression [9], [11], [12], [13], [14], [15]. GSA methods can detect such modules even if their gene components are not significantly differentially expressed when tested individually. GSA has been successfully applied to the analysis of microarray experiments and has contributed to the adoption of a systems-biology perspective in distinct fields such as cancer [16]. Recent findings, brought about by the application of GSA methods on microarray experiments [17] are consistent with the idea that pathways, rather than individual genes, appear to govern the course of tumorigenesis [18]. The use of GSA has been extended to other areas beyond transcriptomics, such as evolution [19], QTL analysis [20] or genotyping [21].

Nevertheless, the different versions of GSA published to date [1], [2], [10] are inherently one-dimensional. Its application to the analysis of genomic datasets is at present limited to the study of a unique variable measured for the genes. The experimental conditions studied, even if corrected by other variables (e.g. age, gender, treatments, etc.), are typically summarized into a unique value for each gene (e.g., differential expression in a case-control, risk in the case of survival analysis, etc.) which is used to rank them accordingly.

Nowadays, the extensive use of different high-throughput methodologies allows the obtention of different measurements for the genes such as methylation status, splicing variants, linkage to diseases, etc., in a straightforward manner. As an illustration of this, a pilot study by The Cancer Genome Atlas (http://cancergenome.nih.gov/) consortium on glioblastomas has recently been published [22]. In it, different types of transcriptomic and genomic profiling were obtained and analyzed in an example of application of different genomic methodologies that would become routine soon. In addition, different measurements of the same type in different experimental contexts can easily be done. For instance, gene expression measurements in case-controls of different, but mechanistically related experimental conditions, phenotypes, diseases, treatments, etc. can be easily obtained. In such scenario, more than one measurement could be obtained to rank the genes involved in the study. Under the conventional GSA paradigm the different ranked lists of genes could be analyzed one at a time and still a good deal of information might be obtained. Nevertheless, by taking this approach any list of ranked genes is considered independent from each other and, consequently, behaviour of functional modules which are dependent on the combination of the studied ranking variables will, most likely, remain undetected.

Here we focus on a conceptually different strategy for GSA by extending the gene set based functional analysis to a multidimensional scenario in which more than one variable or genomic measurement is available for all genes in the study. Logistic regression allows for fitting models that include more than one variable. We show here, by means of several examples, how the application of the multidimensional GSA (MD-GSA) uncovers biological processes activated by different combinations of parameters (measured for all the genes and derived from microarray of other experiments) that would have remained undetected if the parameters would have been analysed one at a time, independently.

## Results

### Gene-set activation dependent on the transcription rates and mRNA activities in yeast

Gene expression is a process that involves two steps of synthesis which end when the appropriate level of protein required for performing a given function is reached. Some processes in the cell require of a quick activation and/or deactivation, while others remain in activity for longer periods and their activation processes do not involve any urgency. Thus, it is expectable different cell functionalities will use different strategies of gene and protein expression and degradation. Measurements of these parameters can be found in a recent genome-wide analysis on common gene expression strategies in yeast [23]. Using these data, we have studied two relevant and opposite biological processes that account for the steady-state mRNA level in the cell: transcription and stability [24]. The authors used a functional enrichment strategy [25] to test the GO terms associated to the parameters measured and to their correlations. Essentially, they used quintiles as cut-off values and tested for enrichments in the genes showing a high or low correlation in rates (transcription and translation) or abundances (mRNA and protein copy number), finding a total of 22 GO terms significantly over-represented at different combinations of rates and abundances. Nevertheless, other interesting situations in which the measurements are not correlated (e.g. transcription rate and mRNA stability) could not be analysed with this approach that, in addition, has the disadvantage of requiring an arbitrary threshold.

Here we analysed the dependences of GO terms on two measurements, transcription rate (TR) and mRNA stability (RS), as well as on the interaction between them. When the logistic model was applied to the mRNA stability and to the transcription rate independently, we obtained 170 and 80 GO terms significantly associated to extreme values of these variables (see Table S1). This increase in the number of GO terms found was due to the well known fact that GSA strategies are much more sensitive than threshold-based functional enrichment strategies [1], [10]. Actually, similar results were obtained when other equivalent GSA strategies were used (data not shown) [11], [19].

**Figure 1:**
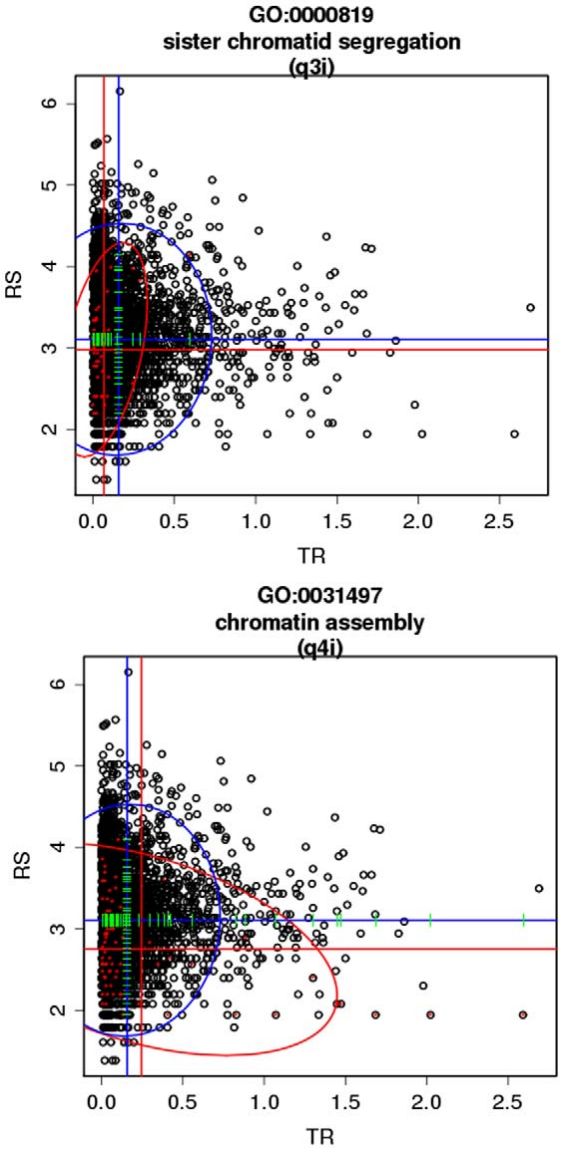
Combined analysis of transcription rates and mRNA stability in yeast with the logistic model. RS (mRNA stability) is represented in vertical axis and TR (transcription rate) is represented in the horizontal axis for GO terms sister chromatid segregation (top) and chromatin assembly (bottom). Blue lines intersect in the mean of the distribution of all the values and red lines intersect in the mean of the distribution of values of the genes corresponding to the GO term analysed. Blue ellipse delimits the confidence interval for all the values and red ellipse delimits the confidence interval for the GO term analysed. The red ellipse marks the trend of the relationship between both variables. MD-GSA assigns patterns q3i and q4i respectively to these functional modules.

Nevertheless, the most interesting aspect of this study is the analysis of the interaction between both variables. Table 1 shows 18 GO terms which were significantly associated to the interaction between transcription rate and mRNA stability. Figure S1 depicts the GO terms within the GO hierarchy. Nine of these GO terms could only be detected when the model takes into account simultaneously both parameters. In most of the cases, the GO was associated to both low transcription rate and mRNA stability (pattern *q3i*, see methods for an explanation of the patterns) such as *sister chromatid segregation* (Figure 1 top) in a subtle way that can only be detected when both parameters are included in the model. On the other hand, other processes, such as *DNA packaging*, *Chromatin assembly* (Figure 1 bottom), *Chromatin assembly or disassembly* and *Establishment and/or maintenance of chromatin architecture* (which are related terms, see File S1), or *protein-DNA complex assembly* are associated to high transcription rates but low mRNA stability (pattern *q4i,* see methods). This last strategy, opposite to the first one, suggest a transient necessity of these processes, whose genes are produced at a fast rate but quickly discarded after their functions have been carried out.

**Table 1:** Significant GO terms when transcription rate and mRNA stability are taken into account in the model.

	Log odds ratio (model coefficients)			Adjusted p-value					
GO id	TR	RS	inter	TR	RS	inter	pattern	new	GO name
GO:0019953	−11.87	−0.82	3.29	0.04	0.01	0.02	q3i	yes	sexual reproduction
GO:0051704	−11.98	−0.69	3.23	0.04	0.02	0.02	q3i	yes	multi-organism process
GO:0000819	−30.49	−0.87	7.1	0.02	0.03	0.02	q3i	yes	sister chromatid segregation
GO:0006260	−20.35	−0.97	4.99	0	0	0.01	q3i	no	DNA replication
GO:0006261	−25.15	−1.31	6.28	0	0	0.01	q3i	no	DNA-dependent DNA replication
GO:0022613	−4.69	−1.78	1.61	0.08	0	0.03	q3i	no	ribonucleoprotein complex biogenesis and assembly
GO:0042254	−5.05	−1.91	1.75	0.09	0	0.03	q3i	no	ribosome biogenesis
GO:0000746	−11.48	−0.73	3.17	0.06	0.02	0.03	q3i	yes	conjugation
GO:0000747	−11.39	−0.74	3.16	0.06	0.02	0.03	q3i	yes	conjugation with cellular fusion
GO:0042221	−6.65	−0.12	2.05	0.02	0.6	0.01	q3i	yes	response to chemical stimulus
GO:0000070	−30.23	−0.78	7.01	0.03	0.07	0.03	q3i	yes	mitotic sister chromatid segregation
GO:0019725	−9.13	−0.38	2.71	0.02	0.15	0.01	q3i	yes	cellular homeostasis
GO:0042592	−8.75	−0.3	2.59	0.02	0.27	0.01	q3i	yes	homeostatic process
GO:0006325	8.01	−0.47	−3.09	0	0.03	0.01	q4i	no	establishment and/or maintenance of chromatin architecture
GO:0065004	12.12	−0.49	−4.6	0	0.21	0.02	q4i	no	protein-DNA complex assembly
GO:0006323	12.63	−0.48	−4.96	0	0.15	0.01	q4i	no	DNA packaging
GO:0006333	12.44	−0.4	−4.84	0	0.23	0.01	q4i	no	chromatin assembly or disassembly
GO:0031497	12.51	−0.44	−4.84	0	0.2	0.01	q4i	no	chromatin assembly

A total of 18 GO terms were found as significant at FDR-adjusted p<0.05, nine of them were also found by the multivariate analysis. Column new indicates if the term as been found only because of the interaction factor (yes) or if it was found also in the univariate analysis in one or both dimensions independently.

Different strategies of production and degradation, corresponding to different biological requirements of the cell, can be thus detected by the combined analysis of these parameters.

#### Gene-set dependences on differential expression and splicing index

Recent studies have shown that more that 70% of the multi-exon genes, corresponding to about 50% of all human genes, are predicted to be alternatively spliced [26]. It is well known that alternative splicing participates in many pathways and processes. Also alterations in splicing function has been implicated in many diseases, including neuropathological conditions such as Alzheimer disease, cystic fibrosis, defects in growth and development, and many human cancers [27].

The magnitude of the alterations in the splicing process can be studied through the splicing index. This index accounts for changes at the exon level that are relative to the expression of the gene. In particular, the intensity value of an exon's probeset is divided by an estimate of the expression level of the transcript cluster to which the exon belongs to. In this way, a gene-level-normalized intensity that can be compared across samples or conditions is created. Changes in this value between case and control samples provide a quantitative measure of alternative splicing between the two conditions [28]. Thus each gene in the data set can be studied both in terms of its differential expression and its alternative splicing. Our multidimensional logistic model can be used to explore this two dimensional gene space.

Here we reanalyze data obtained using Affymetrix exon arrays [29] in which human breast cancer cell lines are compared to non tumorigenic human breast epithelial cell lines. The parameters studied by means of the multidimensional logistic model are: differential gene expression estimates obtained upon the application of a t-test for the above mentioned comparison and a splicing index, that accounts for changes at the exon level that are relative to the expression of the gene [30].

A total of 141 GO terms were found to be significantly associated to high values of the differential gene expression dimension (pattern yh, yl; see methods section). These terms are equivalent to those that would be found by conventional one-dimensional GSA methods and, as expected, GO definitions related to cell proliferation, cell signalling, apoptosis, cellular adhesion, etc., were found among them. One significant GO term, *regulation of viral reproduction*, was significant in the splicing index dimension alone. The trend of the enrichment was towards the positive values of the splicing index (pattern *xh*; see methods section) meaning that genes in the GO term are “subordinately” more spliced in the tumour than in the normal tissue (see File S2A).

**Figure 2:**
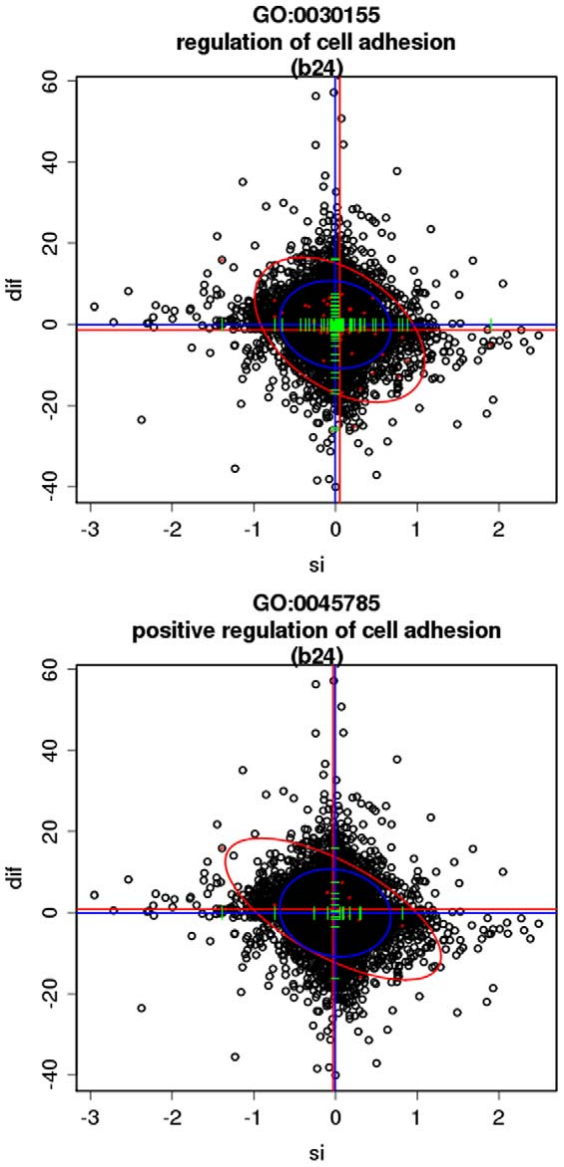
Combined analysis of differential gene expression and splicing index with the logistic model. Differential expression is represented in vertical axis and splicing index is represented in the horizontal axis for GO terms positive regulation of cell adhesion (bottom) and its parent processes regulation of cell adhesion (top). Blue lines intersect in the mean of the distribution of all the values and red lines intersect in the mean of the distribution of values of the genes corresponding to the GO term. Blue ellipse delimits the confidence interval for all the values and red ellipse delimits the confidence interval for the GO term analysed. The red ellipse marks the trend of the relationship between both variables. MD-GSA assigns a bimodal pattern b24 to these functional modules.

Another 12 terms were found by the MD-GSA (see Table 2), whose relationships within the GO hierarchy is depicted in Figure S2. The processes discovered here were related (but yet undetected) to other processes already detected by the conventional analysis of differential expression (see File S2A). For example, *positive regulation of cell adhesion* and its parent *regulation of cell adhesion* are descendants of *cell adhesion*, and two sister processes (*cell-matrix adhesion* and *cell-substrate adhesion*) were found by the model when the two variables were taken into account, and would have remained undetected if a conventional, unidimensional GSA approach would have been used. The patterns for these terms are bimodal in the two dimensional space (pattern *b24*, see methods section) indicating that the genes annotated to them behave as if they were in two sub-modules. For example, *positive regulation of cell adhesion* and its parent processes *regulation of cell adhesion*, which are known to be related to cancer, show a bimodal pattern towards the quadrants 2 and 4 (pattern *b24*). This means that part of the annotated genes are more spliced but underexpressed in the tumour samples while the other part is more spliced but underexpressed in the control samples (see Figure 2).

**Table 2:** Significant GO terms when differential expression and splicing index are taken into account in the model.

	Log odds ratio (model coefficients)			Adjusted p-value				
GO id	splicing	diff.exp	inter	splicing	diff.exp	inter	pattern	GO name
GO:0006767	0.15	−0.15	0.14	1	0.61	0.04	b13	water-soluble vitamin metabolic process
GO:0045216	0.29	−0.04	0.17	1	0.95	0.02	b13	cell-cell junction assembly and maintenance
GO:0007043	0.38	−0.03	0.18	1	0.97	0.02	b13	cell-cell junction assembly
GO:0048706	0.2	0.08	0.17	1	0.89	0.03	b13	embryonic skeletal development
GO:0007034	0.32	−0.18	0.17	1	0.65	0.02	b13	vacuolar transport
GO:0007041	0.32	−0.1	0.18	1	0.86	0.01	b13	lysosomal transport
GO:0048704	0.23	0.12	0.19	1	0.84	0.02	b13	embryonic skeletal morphogenesis
GO:0048705	0.17	0.1	0.17	1	0.85	0.02	b13	skeletal morphogenesis
GO:0016197	0.08	0.1	0.15	1	0.79	0.02	b13	endosome transport
GO:0030155	0.01	−0.16	−0.15	1	0.43	0.01	b24	regulation of cell adhesion
GO:0045785	−0.04	0.06	−0.18	1	0.94	0.02	b24	positive regulation of cell adhesion
GO:0030032	−0.16	−0.17	−0.18	1	0.72	0.03	b24	lamellipodium biogenesis

A total of 12 GO terms were found as significant in the interaction at FDR-adjusted p<0.05.

An equivalent analysis for KEGG can be found in File S2B.

#### Gene-sets differentially activated in related diseases: a case study with psoriasis and dermatitis

The study of gene expression at genomic level in both psoriasis [31] and dermatitis [32] and further functional analysis reveals a considerable number of deregulated pathways when both diseases are compared to their corresponding healthy samples. Thus, when the multivariate logistic model was applied to gene lists arranged by differential expression 172 GO terms were found to be significant only for dermatitis (patterns *xh*, *xl*; see methods section) and 202 only for psoriasis (patterns *yh, yl*). Another 77 GO terms were found to be significant in both, dermatitis and psoriasis but did not show an interaction effect (patterns *q1f, q2f, q3f, q4f*) Most of this terms will also be found by the independent unidimensional analysis of the dermatitis dataset and the psoriasis dataset. In the case of dermatitis, terms related to signalling, cell proliferation, immune system and development of epidermis were found, among others (see Files S3A and S3B). Similar terms can be found in psoriasis with some variations (see Files S3A and S3B). A detailed comparative functional analysis of these diseases is beyond the scope of this manuscript and we will only focus on the results obtained when both diseases are simultaneously analysed.

Table 3 shows the GO terms that are significant when both diseases are taken into account in the logistic model (column labelled with “inter”). Figure S3 shows the GO terms within the GO hierarchy. The GO terms *M phase of mitotic cell cycle* (and their parent terms *M phase* and *cell cycle phase*) and *cell division* where associated to both diseases in their main effects and also in their interaction effect (pattern *q1i*, seemethods ) reinforcing their relevance in the biological mechanisms underlying both skin syndromes. Some other GO terms are only significant in the interaction effect. Their genes show a bimodal behaviour as if the functional module was composed of two sub-units (pattern *b13, b24*; see methods). For instance, GO terms *phosphoinositide-mediated signaling* and *response to reactive oxygen species* have a positive interaction coefficient, which means that some of the genes of the module are being coordinately over-expressed in both diseases while the remaining genes in the GO term are under-expressed also in both diseases. In a symmetric way, *negative regulation of lymphocyte proliferation* (and the parent process *negative regulation of mononuclear cell proliferation*) shows a negative interaction. Part of the genes in these modules increase their expression in dermatitis but decrease it in psoriasis while the rest of them present the opposite behaviour. The reduced cutaneous IFNalpha2 transcription which has been described as a differential characteristic of dermatitis with respect to psoriasis [32] could be causing this effect detectable in the analysis when the two variables are included in the model. All this bimodal terms highlight antagonistic effect, detectable only trough the combined analysis of both diseases.

**Table 3:** Significant GO terms when differential expression of dermatitis and psoriasis are taken into account in the model.

	Log odds ratio (model coefficients)			Adjusted p-value				
GO id	dermatitis	psoriasis	inter	dermatitis	psoriasis	inter	pattern	GO name
GO:0022403	−0.13	0.36	0.11	0.11	0	0.01	q1i	cell cycle phase
GO:0000279	−0.06	0.37	0.12	0.55	0	0.03	q1i	M phase
GO:0051301	−0.1	0.25	0.15	0.36	0	0	q1i	cell division
GO:0000087	−0.11	0.4	0.12	0.32	0	0.05	q1i	M phase of mitotic cell cycle
GO:0048015	0.08	0.07	0.16	0.72	0.68	0.05	b13	phosphoinositide-mediated signaling
GO:0000302	0.24	−0.06	0.29	0.59	0.85	0	b13	response to reactive oxygen species
GO:0032945	0.43	0.33	−0.79	0.26	0.39	0	b24	negative regulation of mononuclear cell proliferation
GO:0050672	0.43	0.33	−0.79	0.26	0.39	0	b24	negative regulation of lymphocyte proliferation
GO:0048589	−0.19	−0.06	−0.59	0.53	0.91	0.04	b24	developmental growth
GO:0007028	0.21	−0.11	−0.75	0.47	0.83	0	b24	cytoplasm organization and biogenesis
GO:0007043	0.07	−0.5	−0.91	0.86	0.22	0	b24	cell-cell junction assembly
GO:0045216	0.12	−0.26	−0.86	0.75	0.59	0	b24	cell-cell junction assembly and maintenance

A total of 12 GO terms were found as significant in the interaction at FDR-adjusted p<0.05.

### Combined analysis of several genomic measurements: a case study with genotyping, gene expression and copy number alterations in breast cancer

It is known that mutations or alteration in copy number are related to cancer and tumour development [33], [34]. Current microarray technologies allow for the measurement of SNP variation and copy number estimation at the same time [35], [36] and have been used to gain insights into breast cancer [37], [38], [39], among other diseases.

Using the multidimensional logistic model proposed we have re-analyzed here data from several separated studies previously collected by us in an integrative analysis of breast cancer disease [38]. In particular we provide a combined description of GO and KEGG relationship to different parameters such as SNP association, copy number alteration and differential gene expression in connection to disease outcome (all the data were taken from the additional information of the above mentioned study, see methods).

When analyzing SNP association data and copy number in luminal B tumours by the proposed MD-GSA, *basal cell carcinoma* KEGG pathway raised up (File S4B) showing a bimodal pattern towards quadrants 1 and 3 (*b13*, see methods). This indicates that the genes in the pathway highly associated to disease are also increased in their copy number, and that genes not associated to disease do not have an increased copy number (they may even have a reduced copy number what would fit with the no association or even protection of the SNPs to disease). Most probably, the SNPs are markers associated either to regions undergoing copy number alterations or to other mutations that affect the *basal cell carcinoma* pathway, which obviously underlies breast cancer disease. The same analysis using the GO reported some negative bimodal terms (Table 4 and File S4B) like *L-amino acid transport* which is known to be involved proliferation processes [40]. A similar analysis with GO terms can be found in File S4A. Figure S4 displays the GO terms in Table 4 within the GO hierarchy.

**Table 4:** Significant GO terms when copy number and gene association to the disease (see text) are taken into account in the model.

	Log odds ratio (model coefficients)			Adjusted p-value				
GO id	association	copy number	inter	association	copy number	inter	pattern	GO name
GO:0015807	−0.09	−0.85	−0.59	0.98	0.46	0.04	b24	L-amino acid transport
GO:0032228	−0.63	−1.21	−0.68	0.65	0.24	0.01	b24	regulation of synaptic transmission, GABAergic
GO:0050805	−0.94	−1.24	−0.63	0.22	0.24	0.04	b24	negative regulation of synaptic transmission
GO:0051932	−0.82	−1.35	−0.67	0.49	0.17	0.02	b24	synaptic transmission, GABAergic
GO:0042398	−0.77	−0.02	0.12	0.04	0.99	1	xl	amino acid derivative biosynthetic process
GO:0042401	−0.93	0.12	0.2	0.01	0.98	1	xl	biogenic amine biosynthetic process
GO:0030216	0.2	0.41	−0.03	0.8	0.03	1	yh	keratinocyte differentiation
GO:0031424	0.29	0.59	−0.01	0.81	0	1	yh	keratinization

A total of 8 GO terms were found as significant at FDR-adjusted p<0.05.

We also applied the MD-GSA to the variables prognosis and differential expression in tumours. In the representation (File S5A), high values in the differential expression dimension indicate under-expression in tumour while low values indicate over-expression. Conversely, high values in the prognosis dimension indicate bad prognosis (if the gene is expressed) while low values in the prognosis dimension indicate good prognosis (if the gene is expressed).

Table 5 (more details in File S5A) show results obtained from the application of the MD-GSA using modules defined with GO terms. The relationships among them within the GO hierarchy are depicted in Figure S5. Most of the GO terms related to *cell division* and *cell cycle* show a *q2i* pattern (see methods) indicating a significant convergence of their genes in the prognosis and differential expression dimensions. From the relatively high prognosis value associated to the genes annotated to this GO terms we know that, if over expressed they indicate bad prognosis. From the low values in the t-statistic we know these GO terms are enriched in the tumours samples. Hence the multivariate logistic model is pointing out those modules which are dangerous to the patient if they are activated, and, that are certainly know to be activated in luminal B tumours. This extended functional analysis provides the researcher not only with a quick an easy interpretation of the combined data but also with the additional information of the interaction term in the model. It is worth pointing out here that better and more detailed results are obtained by combining both datasets under the proposed methodology than by applying independently the univariant methodology to any of the datasets and summing up the results obtained. The equivalent MD-GSA for KEGG pathways can be found in File S5B.

**Table 5:** Significant GO terms when differential expression and prognosis are taken into account in the model.

	Log odds ratio (model coefficients)			Adjusted p-value				
GO id	diff.exp	prognosis	inter	diff.exp	prognosis	inter	pattern	GO name
GO:0000087	−0.45	−0.08	−0.42	0.01	0.81	0	q2i	M phase of mitotic cell cycle
GO:0000279	−0.53	−0.07	−0.38	0.04	0.85	0	q2i	M phase
GO:0000910	−0.27	−0.09	−0.57	0.01	0.95	0	q2i	cytokinesis
GO:0007067	−0.47	−0.07	−0.4	0.04	0.9	0	q2i	mitosis
GO:0022618	−0.22	−0.33	−0.42	0.03	0.21	0	q2i	ribonucleoprotein complex assembly
GO:0051301	−0.38	0	−0.38	0.01	0.99	0	q2i	cell division
GO:0051726	−0.01	0.05	−0.22	0.03	0.91	0.01	q2i	regulation of cell cycle
GO:0045638	0.09	−0.35	−0.6	0.01	0.65	0.04	q4i	negative regulation of myeloid cell differentiation
GO:0000226	−0.08	0.16	−0.31	0.11	0.47	0.02	b24	microtubule cytoskeleton organization and biogenesis
GO:0000278	−0.34	0.04	−0.28	0.11	0.94	0	b24	mitotic cell cycle
GO:0007346	−0.3	−0.08	−0.39	0.07	0.9	0	b24	regulation of mitotic cell cycle
GO:0022403	−0.42	0	−0.31	0.09	0.99	0	b24	cell cycle phase
GO:0042254	−0.4	−0.45	−0.42	0.19	0.1	0.01	b24	ribosome biogenesis
GO:0006412	0.06	−0.28	−0.2	0.02	0.01	0.07	q4f	translation
GO:0006414	0.45	−1.12	−0.43	0	0	0.28	q4f	translational elongation
GO:0042312	0.45	0.08	−0.51	0.03	0.97	0.22	xh	regulation of vasodilation
GO:0000209	−0.25	0.55	0.13	0.94	0.01	1	yh	protein polyubiquitination
GO:0006066	0.08	0.2	−0.02	0.97	0.02	1	yh	alcohol metabolic process
GO:0010033	0.05	0.29	0	0.99	0.02	1	yh	response to organic substance
GO:0032944	−0.17	−0.7	0.06	0.97	0.02	1	yl	regulation of mononuclear cell proliferation
GO:0042098	−0.18	−0.61	0.08	0.95	0.04	1	yl	T cell proliferation
GO:0042110	0.03	−0.38	0.14	0.75	0.03	0.86	yl	T cell activation
GO:0042129	−0.33	−0.74	−0.02	0.99	0.05	1	yl	regulation of T cell proliferation
GO:0045321	−0.04	−0.28	0.06	0.92	0.03	1	yl	leukocyte activation
GO:0046649	−0.06	−0.33	0.07	0.89	0.02	1	yl	lymphocyte activation
GO:0046651	−0.19	−0.49	−0.05	0.99	0.05	1	yl	lymphocyte proliferation
GO:0050670	−0.17	−0.7	0.06	0.97	0.02	1	yl	regulation of lymphocyte proliferation
GO:0051249	−0.06	−0.44	0.24	0.52	0.04	0.71	yl	regulation of lymphocyte activation

Terms were significant at FDR-adjusted p<0.05.

### Advantages and limitations of the logistic regression methodology

The major advantage of the logistic regression methodology is it flexibility. It can be used in any genomic context in which certain biological characteristic of a gene is measured using a numerical scale. This numerical scale may be a continuous “ranking statistic” as described previously [41] or in this paper, but it may also be a categorical variable [42].

Moreover, many modifications of the logistic model with potential applications in biology are already statistically developed and can be used straight forward. Here, for instance we showed how to extend the methodology to study not one but two gene characteristics at a time. It is also straightforward to include the interaction in the model as we showed here. A unidimensional binary logistic model can be used instead the conventional 2×2 contingency table alternative because the logistic model easily allows for weighting genes [42]. This simplicity of extension is not at all intrinsic to most other GSA approaches, what makes the logistic model worth to be explored.

Another advantage of the method is that it does not start from the original observed data set (gene expression matrix for instance) but from a ranking statistic that already summarizes the relevant characteristic under study. This makes the methodology useful in many genomic contexts beyond the microarray paradigm. One example of ranking statistic we have discussed is the classical t-test which, perhaps with some modification, is underneath most GSA methodologies. For each gene, this statistic measures the biological characteristic of “how much” the gene is differentially expressed in a particular biological experiment. But we also exemplified how the ranking statistic can be a hazard ratio form a Cox model or other gene-wise variable[19]. In the case of the hazard ratio, the biological characteristic measured for each gene by the statistic is the association of expression and risk disease. The GSA for this second example can be directly carried out using the logistic methodology and software. On the contrary, most GSA approaches will require major modifications of their methods and software to be applied in a case other than differential gene expression in a class comparison experiment.

Virtually any gene-wise variable can be explored from a GSA perspective using the logistic regression model. In this paper we presented examples for the analysis of transcription rates, mRNA stabilities, splicing, SNP association to disease and copy number estimation. The analysis of other measurements is possible, including the evolutionary selective pressure in the human genome or a study of gene connectivity in the interactome [19]. Other publications also discuss on the advantage of a methodology that starts form a single ranking statistic and not from the whole expression data matrix [42], [43].

Having said that, some remarks and warnings should be given related mainly with the null hypothesis that underpin the method and p-value computation.

In Sator's logistic regression approach [41] and in the extension we are proposing here, the distribution of the ranking statistic within each module is compared to that of its complement. Thus, following Goeman's nomenclature they are “competitive” tests [10]. Also, the way p-values are computed in the logistic model make of this approach a “gene sampling model” methodology [10].

It has been shown that, in general contexts of gene expression, where gene measurements are correlated within modules, GSA approaches that test “competitive” hypothesis based on “gene sampling models” are anticonservative [10]. This undesirable property also applies to the main effects of the bivariate logistic model as we could confirm in simulation studies (only in the case of internal correlation in the gene sets, which is the case of gene expression but not of the rest of the measurements used in this study). Interestingly, the consequence of gene correlation over the interaction effect, which is the main contribution of the proposed methodology, was the opposite and makes the method more conservative (see File S6). One way to avoid the bias of the particular context of gene expression would be to compute p-values based on a subject sampling permutation.

Care should be taken also when interpreting p-values from the method proposed here due to its “competitive” nature and the fact that it starts from a ranking statistic instead of the original data. Consequently, p-values test whether the distribution of the ranking statistic within each module is different to that of the whole genome. Therefore p-values do not extrapolate directly to the individual level class comparison which was done in order to compute the ranking statistic.

## Discussion

Functional annotations, such as GO or KEGG pathways, have been used for the definition of modules of genes, carrying out common functional roles, in functional profiling methods [1], [2]. All these methods, including the most recent versions, such as the GSA, can only deal with data that have been preselected or arranged by a unique variable (e.g. differential gene expression between cases and controls, etc.) The approach we are presenting here constitutes a novel and conceptually different proposal for the functional analysis of genomic experiments. It allows the simultaneous analysis of several variables, which can account for different properties of the genes. This approach can detect interactions between these variables that account for functional roles dependent on several genomic properties or measurements.

We have used for this purpose a logistic model. It has recently been shown that the application of the logistic model to one single variable (differential gene expression in this case) produces results conceptually similar to the outcome of any conventional GSA method [41]. The aim here is not to improve the one dimensional detection of gene modules related to the measurement, but to look for gene modules that have complex dependences on several genomic variables or measurements. Thus, in the first example we show how some functional GO categories depend on particular combinations of their transcription rates and mRNA stabilities. Different strategies can be used by the cellular machinery to ensure, for example, a rapid activation or a long lasting of a particular team of genes that cannot be explained with only one variable. Thus, combinations of several variables (e.g. a rapid transcription rate and a low mRNA stability can be useful for a rapid release and a rapid deactivation of a transient function) are on the root of many biological processes. The variables used can be properties of the genes or can be also measurements of behaviours such as their expression in a given condition. In the second case example we have analyzed a combination of gene property (splicing index) and gene behaviour (differential gene expression). The MD-GSA was able of detecting biological processes that depend on combinations of both variables and would remain undetected if the variables were independently analyzed. Finally, we applied the same concept to the same type of measurement (differential gene expression) in two different but related scenarios: a case control of dermatitis and another case-control of psoriasis. In this example we were able of finding common and distinctive altered functionalities of both related diseases that remained otherwise undetected with the conventional one-dimensional GSA. The combination of measurements that can be studied under this framework and their biological relevance is unimaginable. Thus the relation of biological roles to combinations of different parameters of different types, such as gene intrinsic properties (e.g. mRNA stability), gene behaviours (e.g. level of expression) or gene states (e.g. methylation, SNPs, copy number), etc., can be easily be studied using this approach.

Summarizing, MD-GSA constitutes a novel approach to the functional profiling of genome scale experiments that paves the way for a higher level understanding of the behaviour of functional modules in the cell.

## Materials and Methods

### Datasets and data preprocessing

#### Transcription rates and mRNA stabilities in yeast

Genome-wide values for the transcription rates (TR) and mRNA stabilities (RS) of the genes of yeast used in the first sub-section of results can be found in the supplementary material of the manuscript by Garcia-Martinez et al. [23].

#### Gene expression and splicing index

Okoniewski & Miller [44] used exon arrays to compare breast cancer cell line MCF7 (fetal calf serum) to non tumorgenic breast epithelial cell line MCF10A (horse serum). They estimated differential gene expression using standard t-statistics and alternative splicing using the splicing index described in [30]. Since the splicing index is defined for each exon, we have used here median values to provide splicing measurements at a gene level. Thus, we have two numerical variables recorded for each gene in the study. The first one assesses the variation in the general expression level. The second one quantifies the change in splicing pattern of the gene, independently of its expression levels.

#### Differential expression in psoriasis and dermatitis

Expression data from two separated case control experiments where combined in this analysis. The first experiment consisted of the comparison of lessional and non lessional skin samples in atopic dermatitis patients [32] (data were obtained from the GEO database, accession: GSE5667). The second experiment compared affected and unaffected skin in psoriatic patients [31] (GEO database, accession: GSE6710). Separated gene expression analyses of these two datasets were performed using standard methods: RMA algorithm [45] was used to normalize data within each of the experiments. The limma package [46] from Bioconductor [47] was used to estimate, separately for each of the studies, differential gene expression between diseased and non-diseased skin. Hence, two experimental measurements (limma t-statistics) where generated for each gene and used in the analysis: a first measurement of differential gene expression in dermatitis and a second measurement of differential gene expression in psoriasis.

#### Combined analysis of several breast cancer genomic measurements

Data used in the combined analysis of genomic measurements, in the results section, were taken from the supplementary material of [38]. SNP association to disease was measured using Odds Ratio (OR) of their corresponding minor allele frequencies. Then, the magnitude of the association of each gene to the disease was obtained as the value of association of the SNP more associated to the disease among all the SNPs mapping in the gene (or near the gene and being in linkage disequilibrium) [21], [38]. Differences in gene expression between tumour and normal breast tissues where estimated using t-statistics. Cox regression models where used to correlate survival time and gene expression, yielding a “prognosis” value for each gene (genes with “high” hazard ratios in the Cox model are associated to poor prognosis; genes with “low” hazard ratios associated to good prognosis). Another genomic measurements used was the average copy number for each gene in luminal B tumours, obtained from the hybridization intensity of the probesets corresponding to each gene (taken from the additional material of our study [38]).

#### Annotation Data

Functional modules are defined according the annotations of the GO [4] and the KEGG Pathway [48] repositories. Functional modules of more than 500 genes where considered to be too general to be informative so they where filtered out. Functional modules having less than 10 genes annotated to them where considered to be too small to be properly fitted by the multivariate logistic model and where also discarded.

### Multi dimensional GSA (MD-GSA) using a logistic model that considers more than one variable

Logistic regression is a well established statistical methodology used to model the probability of occurrence of a binary event as a function of some other independent variables [49]. In the context of genomic studies, univariate logistic models have been shown to be suitable to perform gene set enrichment analysis [41].

Modelling functional class membership in terms of some measurement, X, of differential gene expression between two conditions as follows:
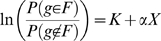
(1)


we can call the gene set F enriched in one of the conditions a significant estimate of the α coefficient is obtained [41].

In this paper we extend the use logistic models to perform a multidimensional gene set enrichment analysis. Our model describes the probability of a gene belonging to a functional class as a function of not one, but several experimental measurements. For two of those measurements the model will be as follows:
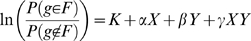
(2)


where α and β are the main effects and γ is the interaction effect.

In a case-control study measuring, for instance, gene expression and genotype, we could model the probability of genes being annotated to a GO term as a function of both, differential gene expression (X) and allelic association to disease (Y).

Modelling not only the additive effects but also the interaction term, we accurately describe how the genes in a gene set are related to both measurements X and Y together, allowing for the detection of enrichment patterns which will remain unnoticed in two independent univariate analyses.

**Figure 3:**
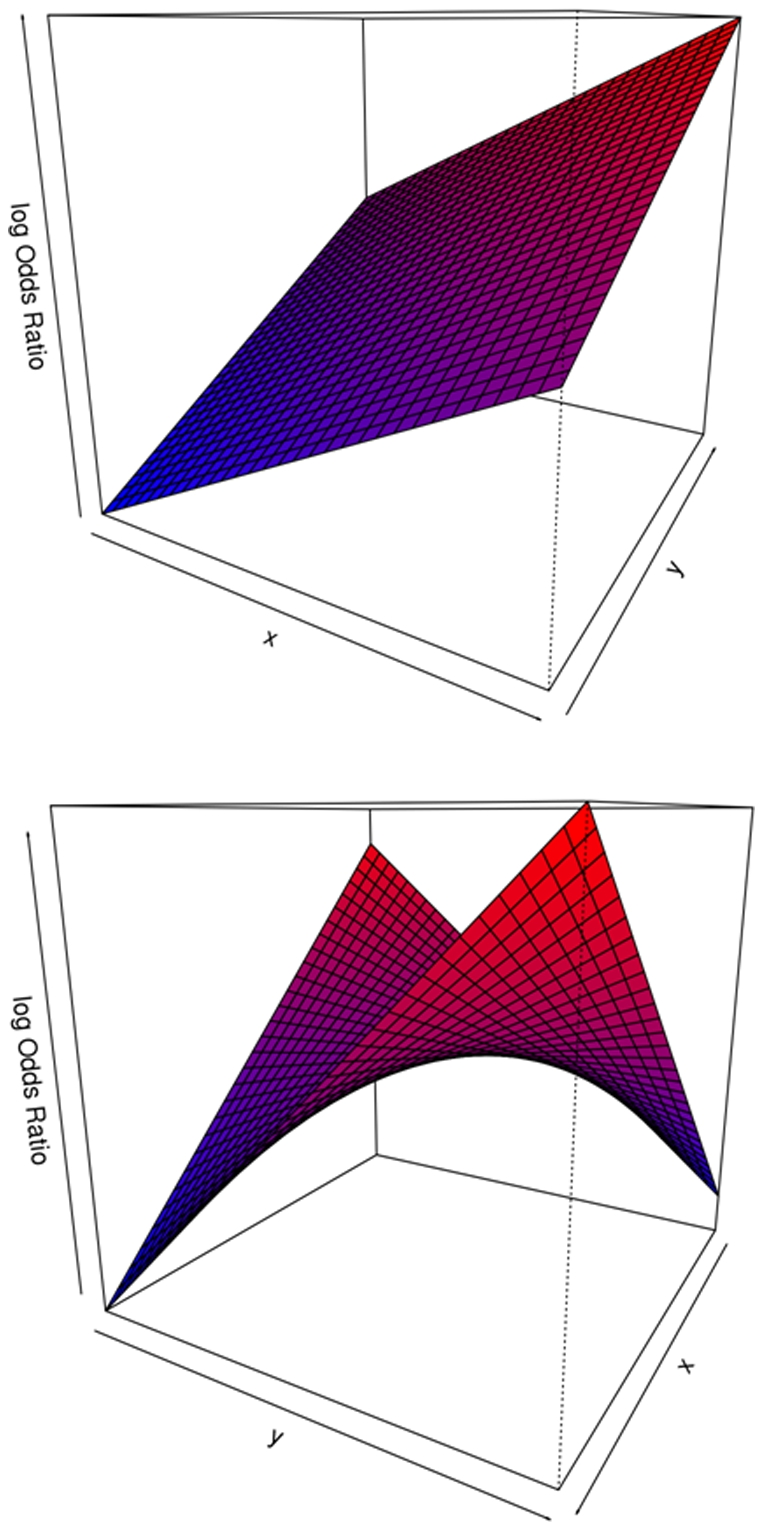
Surfaces described by the logistic model. The surface described by the logistic model is a plane when the interaction term (γ) is 0 (top) and a hyperbolic paraboloid when the interaction term (γ) is not zero (bottom).

The model in equation (2) describes the log odds ratio of a gene being annotated to functional module F as a function of two variables, X and Y. The shape of this surface when embedded in a 3D space is that of a plane if the interaction coefficient γ is zero (Figure 3, top), or a hyperbolic paraboloid, also called saddle surface, when the estimate of γ is different from zero (Figure 3, bottom). Hence, from the sign and significance of the fitted coefficients, we can find the direction in the two dimensional space XY in which the genes annotated to the function F are more likely to be found.

**Figure 4:**
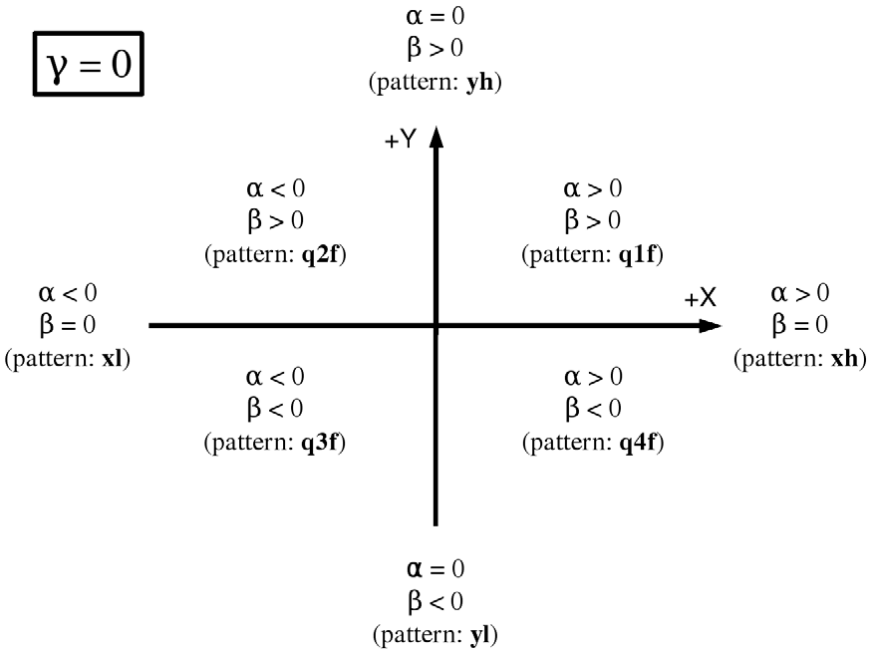
Location of the areas where genes are more likely to be annotated to the function F depending on the coefficients of the fitted model. When γ = 0 the fitted surface is a plane which slope grows towards the area.

When γ is zero the sign of the coefficients α and β describe the slopes of the plane and therefore, the direction towards which the probability of genes being annotated is greater. Figure 4 describes the areas where genes belonging to a functional module are more likely to be found, depending on the estimated α and β coefficients of the logistic model (2) and provided that the estimate of γ is not significantly different from zero.

**Figure 5:**
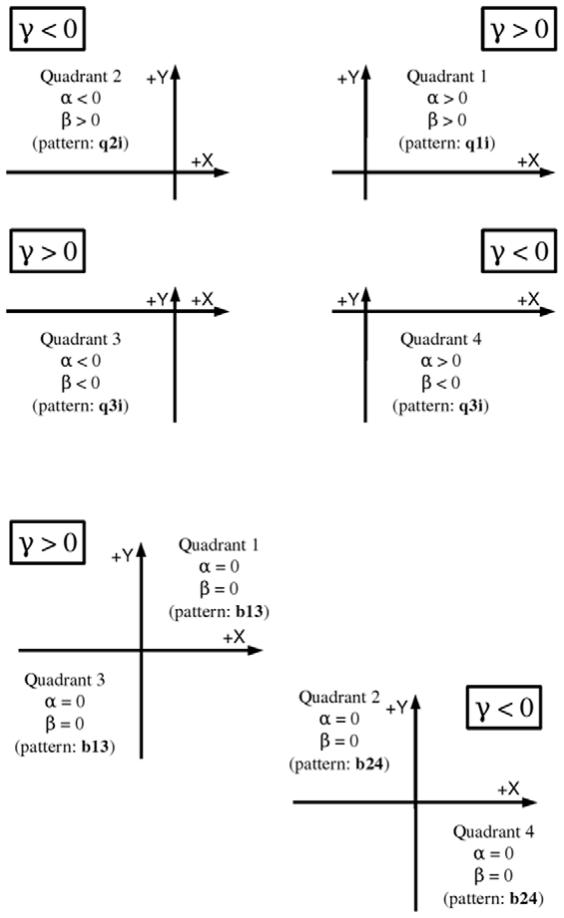
Location of the areas where genes are more likely to be annotated to the function F depending on the coefficients of the fitted model. If γ≠0 the fitted surface is a hyperbolic paraboloid, when α≠0 and β≠0 (top part) the most likely area to find genes annotated to F is the quadrant opposite to the saddle point of the surface. When α = 0 and β = 0 (bottom part) the saddle point of the surface is in the (0,0) and the genes annotated to the function F are more likely to be found in two opposite quadrants, reflecting the bimodality of the function F.

When γ is different from zero the interaction dominates the growth of the log odds ratio while the saddle point in the surface has the coordinates (−β/γ, −α/γ). If for instance, for a particular functional module F, all estimated coefficients are positive, then, the saddle point of the hyperbolic paraboloid will be in the third quadrant and the surface will grow to the infinite in the first quadrant. As the surface represents how likely we are to find genes annotated to module F in the plane XY, we will conclude that the module F is located towards the firs quadrant. Moreover, as the interaction effect is positive we know that the evidence of this localization is greater than the one we will get from separated analysis of each one of the dimensions X and Y on their own (following equation 1). Then, biological interpretation can be done recalling the meaning of the X and Y quantities. Figure 5 (top) describes the areas where genes belonging to a functional module are more likely to be found, depending on the estimates of α, β and γ and when γ is estimated to be different from zero.

If it was the case that just the interaction coefficient γ would be different from zero, then the saddle point will be the (0, 0) and the genes annotated to functional module F will be allocated to opposite quadrants of the XY space; the first and the third quadrant if γ>0; the second and the fourth quadrants if γ<0. In this latest case we will call the functional module F bimodal and the biological interpretation will be that, genes in F are effectively spited up in two groups of opposite patterns. Figure 5 (bottom) describes the areas where genes belonging to a functional module are more likely to be found, if the estimates of α and β are zero.

Table 6 shows how to interpret all possible combinations of α, β and γ estimates.

**Table 6:** Interpretation of all relevant combinations of α, β and γ estimates.

α	β	γ	pattern identifier	pattern	description
+	+	+	q1i	Quadrant 1 with interaction	F is allocated towards one of the quadrants and the evidence is greater than just the additive evidences from the univariate analysis.
+	0	+			
0	+	+			
−	−	+	q3i	Quadrant 3 with interaction	
−	0	+			
0	−	+			
−	+	−	q2i	Quadrant 2 with interaction	
−	0	−			
0	+	−			
+	−	−	q4i	Quadrant 4 with interaction	
+	0	−			
0	−	−			
0	0	+	b13	Bimodal + (quadrants 1 and 3)	F is split in two opposite quadrants.
0	0	+	b24	Bimodal − (quadrants 2 and 4)	
+	+	0	q1f	Quadrant 1 flat	F is allocated towards one of the quadrants and the evidence is similar to the additive evidences from the univariate analysis.
−	−	0	q3f	Quadrant 3 flat	
−	+	0	q2f	Quadrant 2 flat	
+	−	0	q4f	Quadrant 4 flat	
+	0	0	xh	X high (+) values	F is enriched just in the first condition.
−	0	0	xl	X low (−) values	
0	+	0	yh	Y high (+) values	F is enriched just in the second condition.
0	−	0	yl	Y low (−) values	

Wald statistics to test the main effect coefficients and the interaction effects [41]. Other approaches like likelihood ratio tests could also have been used.

As one logistic regression model needs to be fit for each functional module in the analysis, multiple testing occurs and p-value correction must be performed. In this paper we use Benjamini and Hochberg [50] approach to correct all p-values of the same parameter of the model α, β or γ.

### Implementation

The proposed algorithm has been implemented as an R library available at http://bioinfo.cipf.es/supplementary/multidimensional_GSA, released under the GPL license.

## Supporting Information

Figure S1 GO terms significantly associated to the interaction between transcription rate and mRNA stability in yeast. Octagons represent terms with p-values<0.05, after adjustment for multiple testing using the popular FDR [48]. White squares represent non-significant terms connecting the significant terms found. The picture has been obtained using the GOGraphViewer option of the Babelomics package [49].
(1.79 MB JPG)

Figure S2 GO terms significantly associated to the interaction between gene expression and splicing index. Octagons represent terms with p-values<0.05, after adjustment for multiple testing using the popular FDR [48]. White squares represent non-significant terms connecting the significant terms found. The picture has been obtained using the GOGraphViewer option of the Babelomics package [49].
(1.07 MB JPG)

Figure S3 GO terms significantly associated to the interaction between differential gene expression in psoriasis and dermatitis. Octagons represent terms with p-values<0.05, after adjustment for multiple testing using the popular FDR [48]. White squares represent non-significant terms connecting the significant terms found. The picture has been obtained using the GOGraphViewer option of the Babelomics package [49].
(1.66 MB JPG)

Figure S4 GO terms significantly associated to the interaction between copy number and gene association to breast cancer (see text). Octagons represent terms with p-values<0.05, after adjustment for multiple testing using the popular FDR [48]. White squares represent non-significant terms connecting the significant terms found. The picture has been obtained using the GOGraphViewer option of the Babelomics package [49].
(1.12 MB JPG)

Figure S5 GO terms significantly associated to the interaction between differential expression and prognosis of breast cancer. Octagons represent terms with p-values<0.05, after adjustment for multiple testing using the popular FDR [48]. White squares represent non-significant terms connecting the significant terms found. The picture has been obtained using the GOGraphViewer option of the Babelomics package [49].
(0.76 MB JPG)

Table S1 Excel file containing significant GO terms obtained upon the application of the logistic model to the mRNA stability (RS) and to the transcription rate (TR) variables independently.
(0.19 MB XLS)

File S1 A) GO Biological Process terms and B) KEGG pathways, significant for Transcription Rate (TR), RNA Stability (RS) and their interaction, along with the corresponding graphical representations. In the plots blue lines intersect in the mean of the distribution of all the values and red lines intersect in the mean of the distribution of values of the genes corresponding to the GO term analysed. Blue ellipse delimits the confidence interval for all the values and red ellipse delimits the confidence interval for the GO term analysed. The red ellipse marks the trend of the relationship between both variables.
(9.04 MB PDF)

File S2 A) GO Biological Process terms and B) KEGG pathways, significant for alternative splicing and differential gene expression and their interaction, along with the corresponding graphical representations. In the plots blue lines intersect in the mean of the distribution of all the values and red lines intersect in the mean of the distribution of values of the genes corresponding to the term analysed. Blue ellipse delimits the confidence interval for all the values and red ellipse delimits the confidence interval for the term analysed. The red ellipse marks the trend of the relationship between both variables.
(9.22 MB PDF)

File S3 A) GO Biological Process terms, and B) KEGG pathways, significant for differential gene expression in dermatitis and psoriasis case-control studies and their interaction, along with the corresponding graphical representations. In the plots blue lines intersect in the mean of the distribution of all the values and red lines intersect in the mean of the distribution of values of the genes corresponding to the term analysed. Blue ellipse delimits the confidence interval for all the values and red ellipse delimits the confidence interval for the term analysed. The red ellipse marks the trend of the relationship between both variables.
(30.60 MB ZIP)

File S4 A) GO Biological Process terms, and B) KEGG pathways, significant for gene association (derived from genotyping, see text) association data and genomic copy number in breast cancer and their interaction, along with the corresponding graphical representations. In the plots blue lines intersect in the mean of the distribution of all the values and red lines intersect in the mean of the distribution of values of the genes corresponding to the term analysed. Blue ellipse delimits the confidence interval for all the values and red ellipse delimits the confidence interval for the term analysed. The red ellipse marks the trend of the relationship between both variables.
(0.80 MB PDF)

File S5 A) GO Biological Process terms, and B) KEGG pathways, significant for prognosis and differential expression in a case-control study of breast cancer and their interaction, along with the corresponding graphical representations. In the plots blue lines intersect in the mean of the distribution of all the values and red lines intersect in the mean of the distribution of values of the genes corresponding to the term analysed. Blue ellipse delimits the confidence interval for all the values and red ellipse delimits the confidence interval for the term analysed. The red ellipse marks the trend of the relationship between both variables.
(2.22 MB PDF)

File S6 Interaction simulation study. A simulation study of the bias in p-value estimates for the interaction term of the bivariate logistic model.
(0.15 MB DOC)
