# Supplementary figures and images for: Multidimensional Gene Set Analysis of Genomic Data
Source: PLoS One. 2010 Apr 27;5(4):e10348. doi: 10.1371/journal.pone.0010348 (PMC2860497; doi:10.1371/journal.pone.0010348)

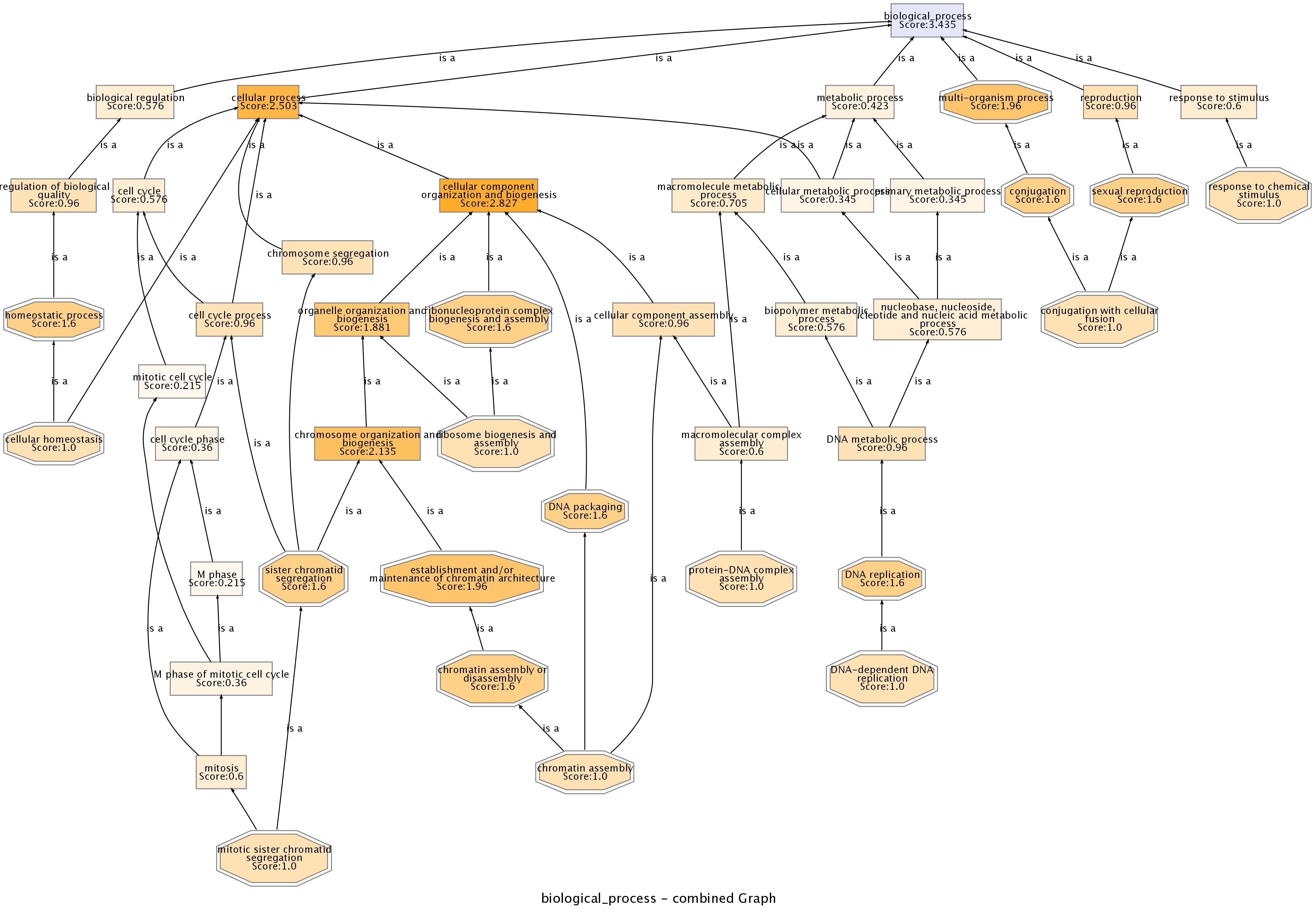

Supplement: Figure S1 — GO terms significantly associated to the interaction between transcription rate and mRNA stability in yeast. Octagons represent terms with p-values<0.05, after adjustment for multiple testing using the popular FDR [48]. White squares represent non-significant terms connecting the significant terms found. The picture has been obtained using the GOGraphViewer option of the Babelomics package [49]. (1.79 MB JPG) [file pone.0010348.s001.jpg]

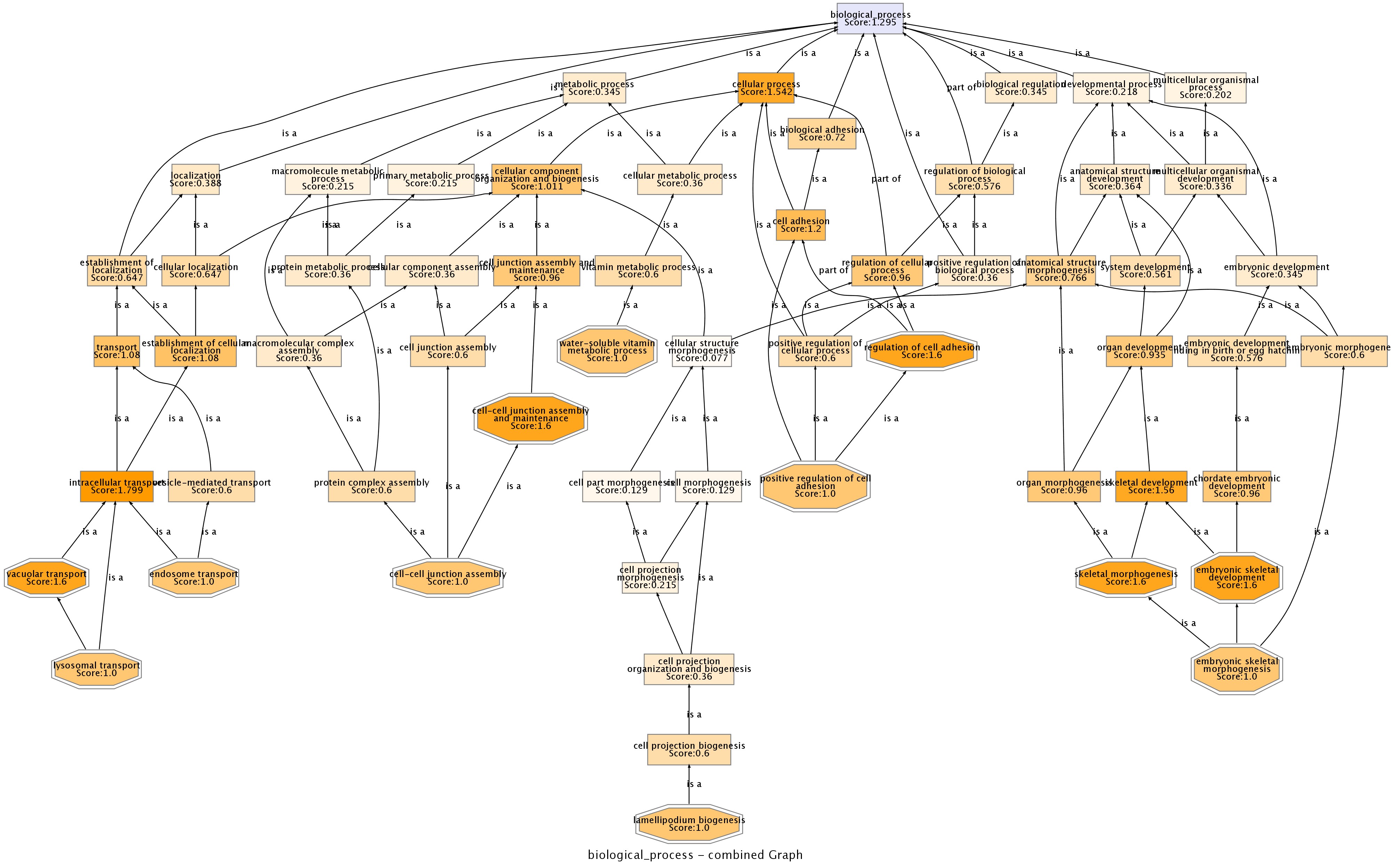

Supplement: Figure S2 — GO terms significantly associated to the interaction between gene expression and splicing index. Octagons represent terms with p-values<0.05, after adjustment for multiple testing using the popular FDR [48]. White squares represent non-significant terms connecting the significant terms found. The picture has been obtained using the GOGraphViewer option of the Babelomics package [49]. (1.07 MB JPG) [file pone.0010348.s002.jpg]

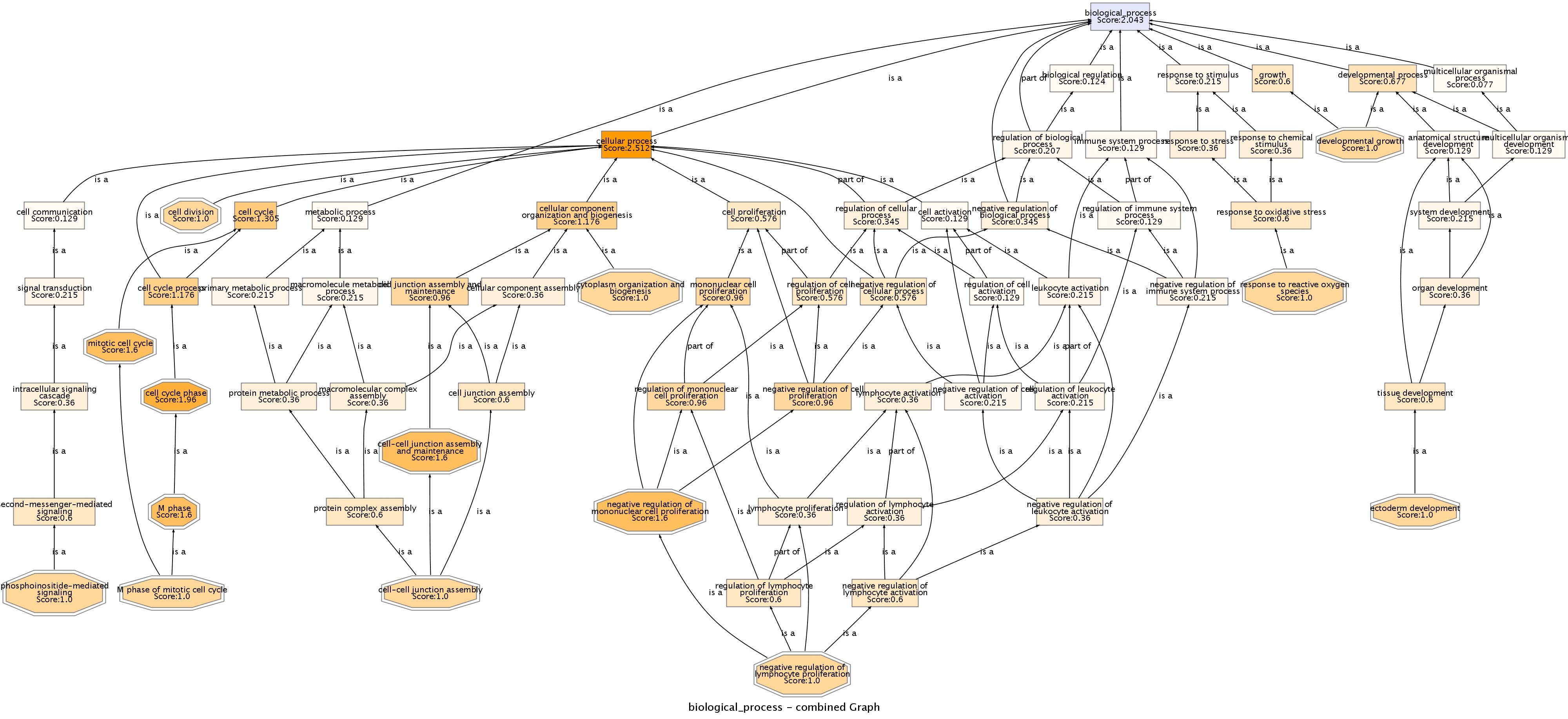

Supplement: Figure S3 — GO terms significantly associated to the interaction between differential gene expression in psoriasis and dermatitis. Octagons represent terms with p-values<0.05, after adjustment for multiple testing using the popular FDR [48]. White squares represent non-significant terms connecting the significant terms found. The picture has been obtained using the GOGraphViewer option of the Babelomics package [49]. (1.66 MB JPG) [file pone.0010348.s003.jpg]

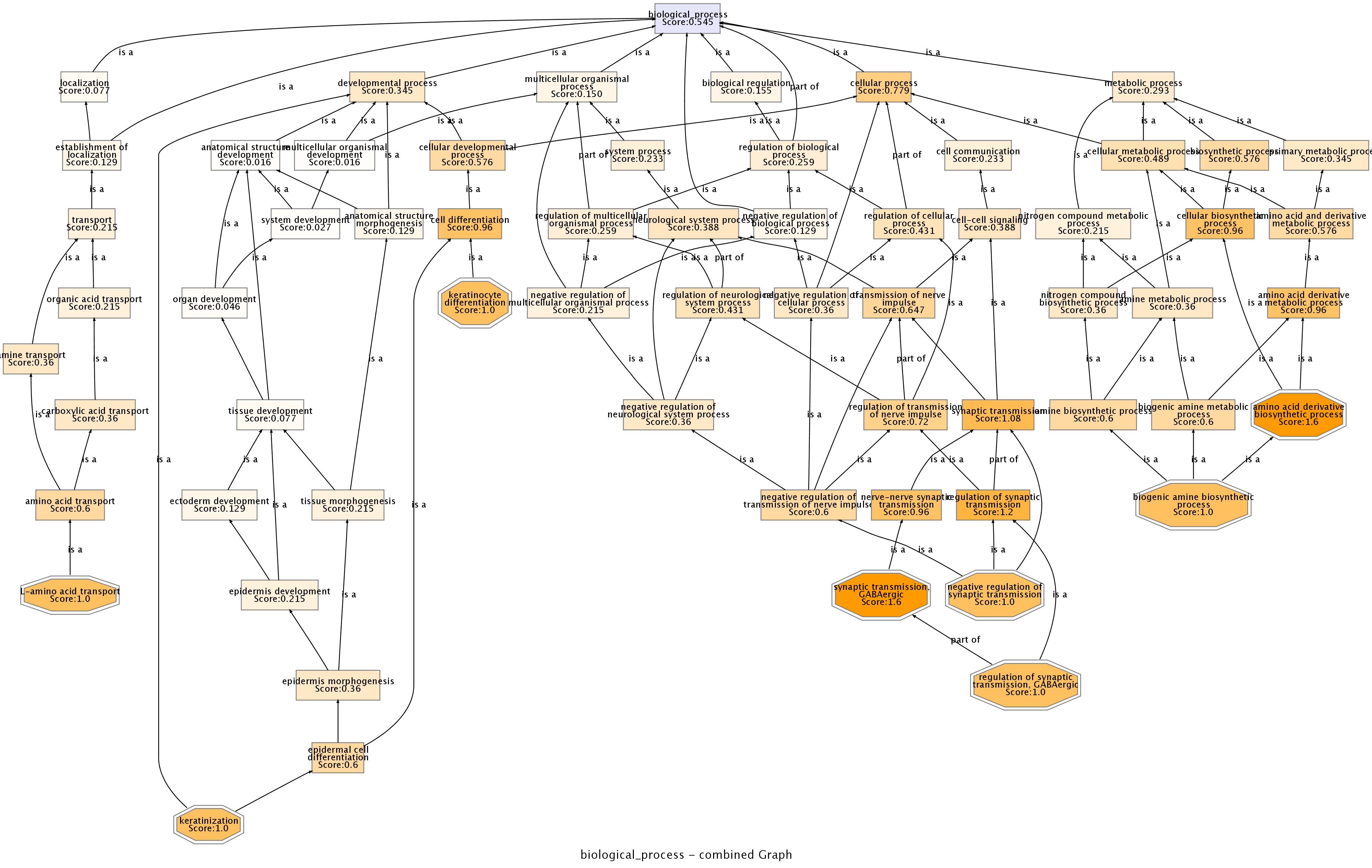

Supplement: Figure S4 — GO terms significantly associated to the interaction between copy number and gene association to breast cancer (see text). Octagons represent terms with p-values<0.05, after adjustment for multiple testing using the popular FDR [48]. White squares represent non-significant terms connecting the significant terms found. The picture has been obtained using the GOGraphViewer option of the Babelomics package [49]. (1.12 MB JPG) [file pone.0010348.s004.jpg]

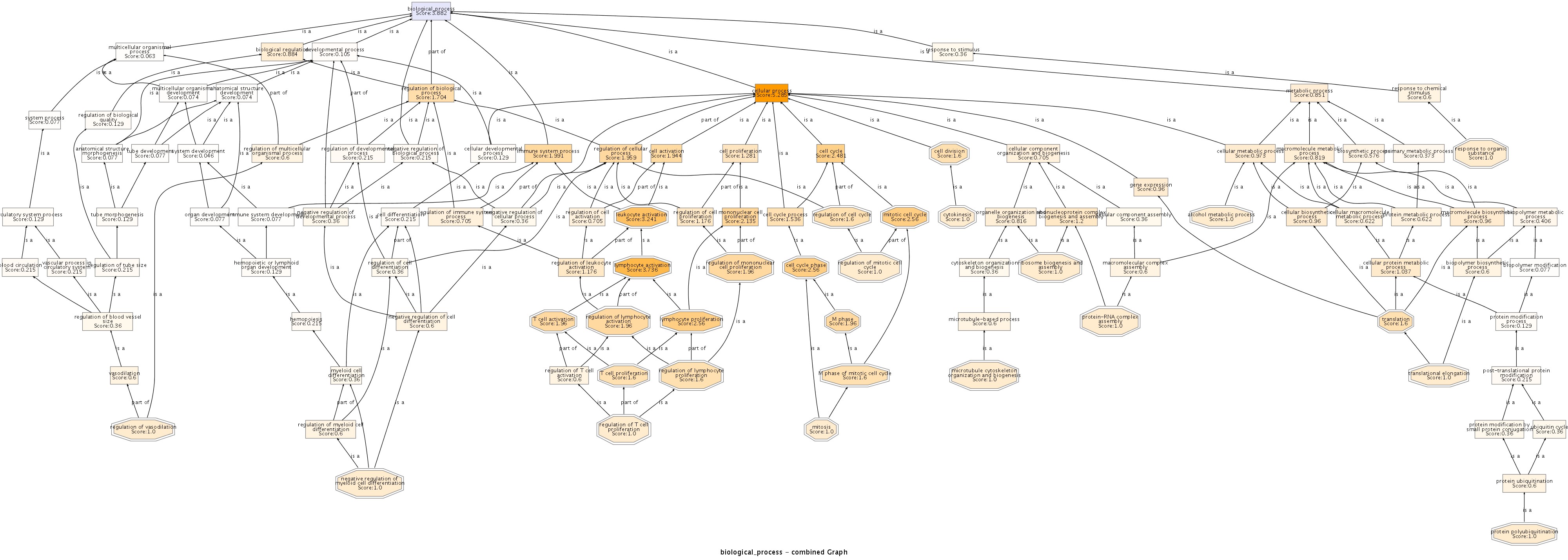

Supplement: Figure S5 — GO terms significantly associated to the interaction between differential expression and prognosis of breast cancer. Octagons represent terms with p-values<0.05, after adjustment for multiple testing using the popular FDR [48]. White squares represent non-significant terms connecting the significant terms found. The picture has been obtained using the GOGraphViewer option of the Babelomics package [49]. (0.76 MB JPG) [file pone.0010348.s005.jpg]
