# Supplementary material for: Multidimensional Gene Set Analysis of Genomic Data
Source: PLoS One. 2010 Apr 27;5(4):e10348. doi: 10.1371/journal.pone.0010348 (PMC2860497; doi:10.1371/journal.pone.0010348)
Supplement: File S1 — A) GO Biological Process terms and B) KEGG pathways, significant for Transcription Rate (TR), RNA Stability (RS) and their interaction, along with the corresponding graphical representations. In the plots blue lines intersect in the mean of the distribution of all the values and red lines intersect in the mean of the distribution of values of the genes corresponding to the GO term analysed. Blue ellipse delimits the confidence interval for all the values and red ellipse delimits the confidence interval for the GO term analysed. The red ellipse marks the trend of the relationship between both variables. (9.04 MB PDF) [file pone.0010348.s007.pdf]

# Combined functional profiling of Transcription Rate (TR) and RNA Stability (RS) using GO Biological Process

**140** significant modules

**LOR** indicates Log Odds Ratio of the coefficient or the interaction

**p** indicates FDR adjusted p-value

| | LOR TR | LOR RS | LOR Inter | p TR | p RS | p Inter | pattern | name |
|---|---|---|---|---|---|---|---|---|
| GO:0022613 | -4.69 | -1.78 | 1.61 | 0.08 | 0.00 | 0.03 | q3i | ribonucleoprotein complex biogenesis and assembly |
| GO:0042254 | -5.05 | -1.91 | 1.75 | 0.09 | 0.00 | 0.03 | q3i | ribosome biogenesis |
| GO:0000746 | -11.48 | -0.73 | 3.17 | 0.06 | 0.02 | 0.03 | q3i | conjugation |
| GO:0000747 | -11.39 | -0.74 | 3.16 | 0.06 | 0.02 | 0.03 | q3i | conjugation with cellular fusion |
| GO:0019953 | -11.87 | -0.82 | 3.29 | 0.04 | 0.01 | 0.02 | q3i | sexual reproduction |
| GO:0042221 | -6.65 | -0.12 | 2.05 | 0.02 | 0.60 | 0.01 | q3i | response to chemical stimulus |
| GO:0051704 | -11.98 | -0.69 | 3.23 | 0.04 | 0.02 | 0.02 | q3i | multi-organism process |
| GO:0000819 | -30.49 | -0.87 | 7.10 | 0.02 | 0.03 | 0.02 | q3i | sister chromatid segregation |
| GO:0000070 | -30.23 | -0.78 | 7.01 | 0.03 | 0.07 | 0.03 | q3i | mitotic sister chromatid segregation |
| GO:0006260 | -20.35 | -0.97 | 4.99 | 0.00 | 0.00 | 0.01 | q3i | DNA replication |
| GO:0006261 | -25.15 | -1.31 | 6.28 | 0.00 | 0.00 | 0.01 | q3i | DNA-dependent DNA replication |
| GO:0019725 | -9.13 | -0.38 | 2.71 | 0.02 | 0.15 | 0.01 | q3i | cellular homeostasis |
| GO:0042592 | -8.75 | -0.30 | 2.59 | 0.02 | 0.27 | 0.01 | q3i | homeostatic process |
| GO:0065004 | 12.12 | -0.49 | -4.60 | 0.00 | 0.21 | 0.02 | q4i | protein-DNA complex assembly |
| GO:0006325 | 8.01 | -0.47 | -3.09 | 0.00 | 0.03 | 0.01 | q4i | establishment and/or maintenance of chromatin architecture |
| GO:0006323 | 12.63 | -0.48 | -4.96 | 0.00 | 0.15 | 0.01 | q4i | DNA packaging |
| GO:0006333 | 12.44 | -0.40 | -4.84 | 0.00 | 0.23 | 0.01 | q4i | chromatin assembly or disassembly |
| GO:0031497 | 12.51 | -0.44 | -4.84 | 0.00 | 0.20 | 0.01 | q4i | chromatin assembly |
| GO:0006082 | 0.58 | 0.56 | -0.18 | 0.99 | 0.00 | 1.00 | yh | organic acid metabolic process |
| GO:0006519 | 2.41 | 0.43 | -0.88 | 0.66 | 0.02 | 0.58 | yh | amino acid and derivative metabolic process |
| GO:0006520 | 2.44 | 0.45 | -0.87 | 0.65 | 0.02 | 0.58 | yh | amino acid metabolic process |
| GO:0006807 | 2.56 | 0.44 | -1.01 | 0.61 | 0.01 | 0.46 | yh | nitrogen compound metabolic process |
| GO:0009308 | 2.41 | 0.41 | -0.89 | 0.65 | 0.03 | 0.57 | yh | amine metabolic process |
| GO:0019752 | 0.58 | 0.56 | -0.18 | 0.99 | 0.00 | 1.00 | yh | carboxylic acid metabolic process |
| GO:0009056 | -0.54 | 0.34 | 0.15 | 0.99 | 0.02 | 1.00 | yh | catabolic process |
| GO:0044248 | -0.21 | 0.38 | 0.09 | 0.99 | 0.01 | 1.00 | yh | cellular catabolic process |
| GO:0005975 | -0.32 | 0.87 | 0.01 | 0.99 | 0.00 | 1.00 | yh | carbohydrate metabolic process |
| GO:0005996 | -0.88 | 0.97 | 0.37 | 0.99 | 0.00 | 1.00 | yh | monosaccharide metabolic process |
| GO:0006066 | -0.97 | 0.76 | 0.31 | 0.99 | 0.00 | 1.00 | yh | alcohol metabolic process |
| GO:0044262 | -0.08 | 0.88 | -0.07 | 1.00 | 0.00 | 1.00 | yh | cellular carbohydrate metabolic process |
| GO:0019318 | -1.03 | 0.93 | 0.43 | 0.99 | 0.00 | 1.00 | yh | hexose metabolic process |
| GO:0032787 | -1.77 | 0.75 | 0.58 | 0.99 | 0.00 | 1.00 | yh | monocarboxylic acid metabolic process |
| GO:0006006 | -1.37 | 0.95 | 0.61 | 0.99 | 0.00 | 1.00 | yh | glucose metabolic process |
| GO:0006007 | 0.02 | 1.23 | 0.35 | 1.00 | 0.00 | 1.00 | yh | glucose catabolic process |
| GO:0016052 | -1.20 | 1.09 | 0.55 | 0.99 | 0.00 | 1.00 | yh | carbohydrate catabolic process |
| GO:0019320 | -0.44 | 1.16 | 0.44 | 0.99 | 0.00 | 1.00 | yh | hexose catabolic process |
| GO:0044275 | -1.20 | 1.09 | 0.55 | 0.99 | 0.00 | 1.00 | yh | cellular carbohydrate catabolic process |
| GO:0046164 | -0.86 | 1.15 | 0.56 | 0.99 | 0.00 | 1.00 | yh | alcohol catabolic process |
| GO:0046365 | -0.15 | 1.25 | 0.35 | 0.99 | 0.00 | 1.00 | yh | monosaccharide catabolic process |
| GO:0006090 | -0.15 | 1.15 | 0.45 | 0.99 | 0.01 | 1.00 | yh | pyruvate metabolic process |
| GO:0006096 | -0.93 | 1.13 | 0.65 | 0.99 | 0.01 | 1.00 | yh | glycolysis |
| GO:0009063 | 5.11 | 1.04 | -1.75 | 0.59 | 0.03 | 0.68 | yh | amino acid catabolic process |
| GO:0009310 | 5.53 | 1.09 | -1.99 | 0.56 | 0.02 | 0.61 | yh | amine catabolic process |
| GO:0044270 | 5.53 | 1.09 | -1.99 | 0.56 | 0.02 | 0.61 | yh | nitrogen compound catabolic process |
| GO:0006084 | 5.12 | 1.35 | -1.65 | 0.46 | 0.00 | 0.52 | yh | acetyl-CoA metabolic process |
| GO:0006732 | 0.20 | 0.63 | -0.10 | 0.99 | 0.01 | 1.00 | yh | coenzyme metabolic process |
| GO:0051186 | 0.45 | 0.58 | -0.10 | 0.99 | 0.01 | 1.00 | yh | cofactor metabolic process |
| GO:0006091 | 1.50 | 0.40 | -0.23 | 0.80 | 0.03 | 1.00 | yh | generation of precursor metabolites and energy |
| GO:0006113 | 5.76 | 1.41 | -1.76 | 0.46 | 0.01 | 0.61 | yh | fermentation |
| GO:0015980 | 2.73 | 0.58 | -0.66 | 0.48 | 0.02 | 0.70 | yh | energy derivation by oxidation of organic compounds |
| GO:0019751 | 4.35 | 1.38 | -1.11 | 0.66 | 0.02 | 0.92 | yh | polyol metabolic process |
| GO:0006536 | 3.21 | 1.30 | -0.86 | 0.99 | 0.03 | 1.00 | yh | glutamate metabolic process |
| GO:0009084 | 3.78 | 0.98 | -1.30 | 0.93 | 0.03 | 0.95 | yh | glutamine family amino acid biosynthetic process |
| GO:0006537 | 3.28 | 1.37 | -0.87 | 0.99 | 0.03 | 1.00 | yh | glutamate biosynthetic process |
| GO:0006766 | -1.01 | 0.63 | 0.23 | 0.99 | 0.05 | 1.00 | yh | vitamin metabolic process |
| GO:0006767 | -1.01 | 0.63 | 0.23 | 0.99 | 0.05 | 1.00 | yh | water-soluble vitamin metabolic process |
| GO:0030435 | 4.76 | 0.56 | -1.98 | 0.43 | 0.02 | 0.27 | yh | sporulation |
| GO:0005984 | -70.69 | 1.26 | 12.40 | 0.49 | 0.05 | 0.61 | yh | disaccharide metabolic process |
| GO:0008645 | -1.41 | 1.40 | 0.02 | 0.99 | 0.02 | 1.00 | yh | hexose transport |
| GO:0015749 | -1.41 | 1.40 | 0.02 | 0.99 | 0.02 | 1.00 | yh | monosaccharide transport |
| GO:0006094 | -0.30 | 1.11 | 0.57 | 0.99 | 0.03 | 1.00 | yh | gluconeogenesis |
| GO:0019319 | -0.14 | 0.98 | 0.50 | 0.99 | 0.05 | 1.00 | yh | hexose biosynthetic process |
| GO:0046165 | 1.85 | 1.14 | -0.05 | 0.99 | 0.01 | 1.00 | yh | alcohol biosynthetic process |
| GO:0046364 | -0.14 | 0.98 | 0.50 | 0.99 | 0.05 | 1.00 | yh | monosaccharide biosynthetic process |
| GO:0006739 | 2.55 | 1.46 | -0.49 | 0.99 | 0.00 | 1.00 | yh | NADP metabolic process |
| GO:0006740 | 3.11 | 1.61 | -0.58 | 0.95 | 0.00 | 1.00 | yh | NADPH regeneration |
| GO:0006769 | -0.60 | 0.89 | 0.32 | 0.99 | 0.03 | 1.00 | yh | nicotinamide metabolic process |
| GO:0019362 | -0.83 | 0.86 | 0.37 | 0.99 | 0.03 | 1.00 | yh | pyridine nucleotide metabolic process |
| GO:0006098 | 2.55 | 1.46 | -0.39 | 0.99 | 0.01 | 1.00 | yh | pentose-phosphate shunt |
| GO:0006067 | 3.53 | 1.79 | -1.08 | 0.99 | 0.00 | 1.00 | yh | ethanol metabolic process |
| GO:0009072 | 7.43 | 1.06 | -3.32 | 0.79 | 0.02 | 0.62 | yh | aromatic amino acid family metabolic process |

| | | | | | | | | |
|---|---|---|---|---|---|---|---|---|
| GO:0016042 | 18.81 | 1.09 | -10.53 | 0.95 | 0.03 | 0.61 | yh | lipid catabolic process |
| GO:0009081 | 5.35 | 1.11 | -1.70 | 0.46 | 0.02 | 0.58 | yh | branched chain family amino acid metabolic process |
| GO:0006081 | -0.84 | 1.39 | 0.01 | 0.99 | 0.02 | 1.00 | yh | aldehyde metabolic process |
| GO:0009109 | 4.88 | 1.30 | -1.52 | 0.49 | 0.00 | 0.58 | yh | coenzyme catabolic process |
| GO:0046356 | 4.97 | 1.35 | -1.52 | 0.47 | 0.00 | 0.57 | yh | acetyl-CoA catabolic process |
| GO:0051187 | 4.77 | 1.40 | -1.46 | 0.49 | 0.00 | 0.59 | yh | cofactor catabolic process |
| GO:0006100 | 5.29 | 1.19 | -1.58 | 0.46 | 0.02 | 0.61 | yh | |
| GO:0006099 | 4.97 | 1.35 | -1.52 | 0.47 | 0.00 | 0.57 | yh | tricarboxylic acid cycle |
| GO:0006071 | 4.12 | 1.26 | -1.02 | 0.73 | 0.04 | 0.95 | yh | glycerol metabolic process |
| GO:0044242 | 19.65 | 1.51 | -9.69 | 0.56 | 0.00 | 0.43 | yh | cellular lipid catabolic process |
| GO:0022607 | 3.20 | -0.68 | -1.01 | 0.17 | 0.00 | 0.29 | yl | cellular component assembly |
| GO:0065003 | 2.62 | -0.90 | -0.67 | 0.42 | 0.00 | 0.64 | yl | macromolecular complex assembly |
| GO:0006366 | 0.16 | -0.63 | -0.37 | 0.99 | 0.00 | 1.00 | yl | transcription from RNA polymerase II promoter |
| GO:0006357 | -0.32 | -0.60 | -0.35 | 0.99 | 0.02 | 1.00 | yl | regulation of transcription from RNA polymerase II promoter |
| GO:0016568 | 2.18 | -0.64 | -1.61 | 0.99 | 0.01 | 0.84 | yl | chromatin modification |
| GO:0009059 | 0.66 | -0.49 | 0.26 | 0.99 | 0.00 | 0.95 | yl | macromolecule biosynthetic process |
| GO:0000278 | -6.84 | -0.75 | 1.60 | 0.38 | 0.00 | 0.53 | yl | mitotic cell cycle |
| GO:0051726 | -7.52 | -0.93 | 1.68 | 0.70 | 0.00 | 0.95 | yl | regulation of cell cycle |
| GO:0006396 | -5.40 | -1.47 | 1.41 | 0.35 | 0.00 | 0.46 | yl | RNA processing |
| GO:0008033 | -0.88 | -0.92 | 0.12 | 0.99 | 0.01 | 1.00 | yl | tRNA processing |
| GO:0009451 | -1.42 | -1.49 | -0.41 | 0.99 | 0.00 | 1.00 | yl | RNA modification |
| GO:0006400 | -5.93 | -1.22 | 1.21 | 0.99 | 0.03 | 1.00 | yl | tRNA modification |
| GO:0006401 | -0.39 | -1.02 | 0.04 | 0.99 | 0.02 | 1.00 | yl | RNA catabolic process |
| GO:0006402 | 0.04 | -1.00 | -0.01 | 1.00 | 0.03 | 1.00 | yl | mRNA catabolic process |
| GO:0016071 | -2.19 | -0.78 | 0.58 | 0.99 | 0.00 | 1.00 | yl | mRNA metabolic process |
| GO:0006397 | -3.22 | -0.82 | 0.90 | 0.93 | 0.00 | 0.95 | yl | mRNA processing |
| GO:0051325 | -4.72 | -0.88 | 1.03 | 0.97 | 0.02 | 1.00 | yl | interphase |
| GO:0051329 | -4.72 | -0.88 | 1.03 | 0.97 | 0.02 | 1.00 | yl | interphase of mitotic cell cycle |
| GO:0006486 | -7.70 | -0.93 | 1.80 | 0.79 | 0.02 | 0.95 | yl | protein amino acid glycosylation |
| GO:0009100 | -2.61 | -0.87 | 0.54 | 0.99 | 0.02 | 1.00 | yl | glycoprotein metabolic process |
| GO:0009101 | -2.70 | -0.90 | 0.59 | 0.99 | 0.02 | 1.00 | yl | glycoprotein biosynthetic process |
| GO:0043413 | -7.70 | -0.93 | 1.80 | 0.79 | 0.02 | 0.95 | yl | biopolymer glycosylation |
| GO:0000087 | -9.82 | -0.87 | 2.40 | 0.28 | 0.00 | 0.39 | yl | M phase of mitotic cell cycle |
| GO:0007067 | -9.74 | -0.88 | 2.33 | 0.35 | 0.00 | 0.46 | yl | mitosis |
| GO:0007088 | -15.73 | -1.30 | 3.79 | 0.58 | 0.02 | 0.74 | yl | regulation of mitosis |
| GO:0016072 | -5.48 | -1.90 | 1.37 | 0.72 | 0.00 | 0.95 | yl | rRNA metabolic process |
| GO:0006364 | -5.46 | -1.96 | 1.41 | 0.70 | 0.00 | 0.94 | yl | rRNA processing |
| GO:0022618 | -2.58 | -1.46 | 1.13 | 0.70 | 0.00 | 0.39 | yl | ribonucleoprotein complex assembly |
| GO:0006413 | -2.22 | -1.47 | 0.97 | 0.99 | 0.00 | 0.95 | yl | translational initiation |
| GO:0007166 | -5.86 | -0.98 | 1.86 | 0.72 | 0.04 | 0.61 | yl | cell surface receptor linked signal transduction |
| GO:0031137 | -7.32 | -1.61 | 2.54 | 0.58 | 0.02 | 0.39 | yl | regulation of conjugation with cellular fusion |
| GO:0032005 | -7.32 | -1.61 | 2.54 | 0.58 | 0.02 | 0.39 | yl | signal transduction during conjugation with cellular fusion |
| GO:0046999 | -7.32 | -1.61 | 2.54 | 0.58 | 0.02 | 0.39 | yl | regulation of conjugation |
| GO:0000750 | -7.32 | -1.61 | 2.54 | 0.58 | 0.02 | 0.39 | yl | pheromone-dependent signal transduction during conjugation with cellular fusion |
| GO:0006730 | -5.70 | -0.86 | 1.50 | 0.93 | 0.04 | 0.95 | yl | one-carbon compound metabolic process |
| GO:0000054 | -6.34 | -1.35 | 1.94 | 0.95 | 0.05 | 0.95 | yl | ribosome export from nucleus |
| GO:0033750 | -6.34 | -1.35 | 1.94 | 0.95 | 0.05 | 0.95 | yl | ribosome localization |
| GO:0033753 | -6.34 | -1.35 | 1.94 | 0.95 | 0.05 | 0.95 | yl | establishment of ribosome localization |
| GO:0006487 | -8.84 | -1.01 | 2.34 | 0.70 | 0.03 | 0.74 | yl | protein amino acid N-linked glycosylation |
| GO:0032259 | 0.54 | -1.21 | -1.23 | 0.99 | 0.03 | 1.00 | yl | methylation |
| GO:0043414 | 0.54 | -1.21 | -1.23 | 0.99 | 0.03 | 1.00 | yl | biopolymer methylation |
| GO:0006338 | 1.03 | -0.84 | -1.39 | 0.99 | 0.01 | 1.00 | yl | chromatin remodeling |
| GO:0040029 | 0.26 | -0.88 | -0.80 | 0.99 | 0.02 | 1.00 | yl | regulation of gene expression, epigenetic |
| GO:0048519 | -0.07 | -0.52 | -0.42 | 1.00 | 0.04 | 1.00 | yl | negative regulation of biological process |
| GO:0048523 | -0.18 | -0.51 | -0.39 | 0.99 | 0.04 | 1.00 | yl | negative regulation of cellular process |
| GO:0006497 | -14.22 | -1.02 | 3.75 | 0.39 | 0.03 | 0.31 | yl | protein amino acid lipidation |
| GO:0042157 | -14.22 | -1.02 | 3.75 | 0.39 | 0.03 | 0.31 | yl | lipoprotein metabolic process |
| GO:0042158 | -14.22 | -1.02 | 3.75 | 0.39 | 0.03 | 0.31 | yl | lipoprotein biosynthetic process |
| GO:0042255 | -2.49 | -1.80 | 1.31 | 0.82 | 0.00 | 0.39 | yl | ribosome assembly |
| GO:0042257 | -2.11 | -1.73 | 1.25 | 0.93 | 0.00 | 0.39 | yl | ribosomal subunit assembly |
| GO:0007062 | -35.59 | -1.24 | 7.67 | 0.46 | 0.04 | 0.58 | yl | sister chromatid cohesion |
| GO:0042273 | -4.79 | -2.86 | 2.04 | 0.47 | 0.00 | 0.14 | yl | ribosomal large subunit biogenesis |
| GO:0007064 | -65.49 | -1.42 | 14.17 | 0.19 | 0.03 | 0.18 | yl | mitotic sister chromatid cohesion |
| GO:0050793 | -15.23 | -1.52 | 4.16 | 0.58 | 0.03 | 0.52 | yl | regulation of developmental process |
| GO:0000154 | 16.04 | -2.21 | -8.62 | 0.95 | 0.02 | 0.95 | yl | rRNA modification |
| GO:0030490 | -12.30 | -2.32 | 3.36 | 0.56 | 0.00 | 0.62 | yl | maturation of SSU-rRNA |
| GO:0000027 | -3.09 | -2.38 | 1.60 | 0.79 | 0.00 | 0.38 | yl | ribosomal large subunit assembly and maintenance |
| GO:0043681 | 0.46 | -1.40 | 0.21 | 0.99 | 0.02 | 1.00 | yl | protein import into mitochondrion |
| GO:0000114 | -5.92 | -2.25 | 1.88 | 0.99 | 0.02 | 1.00 | yl | G1-specific transcription in mitotic cell cycle |

Table 1: LOR: log odds ratio; p: adjusted p-value FDR

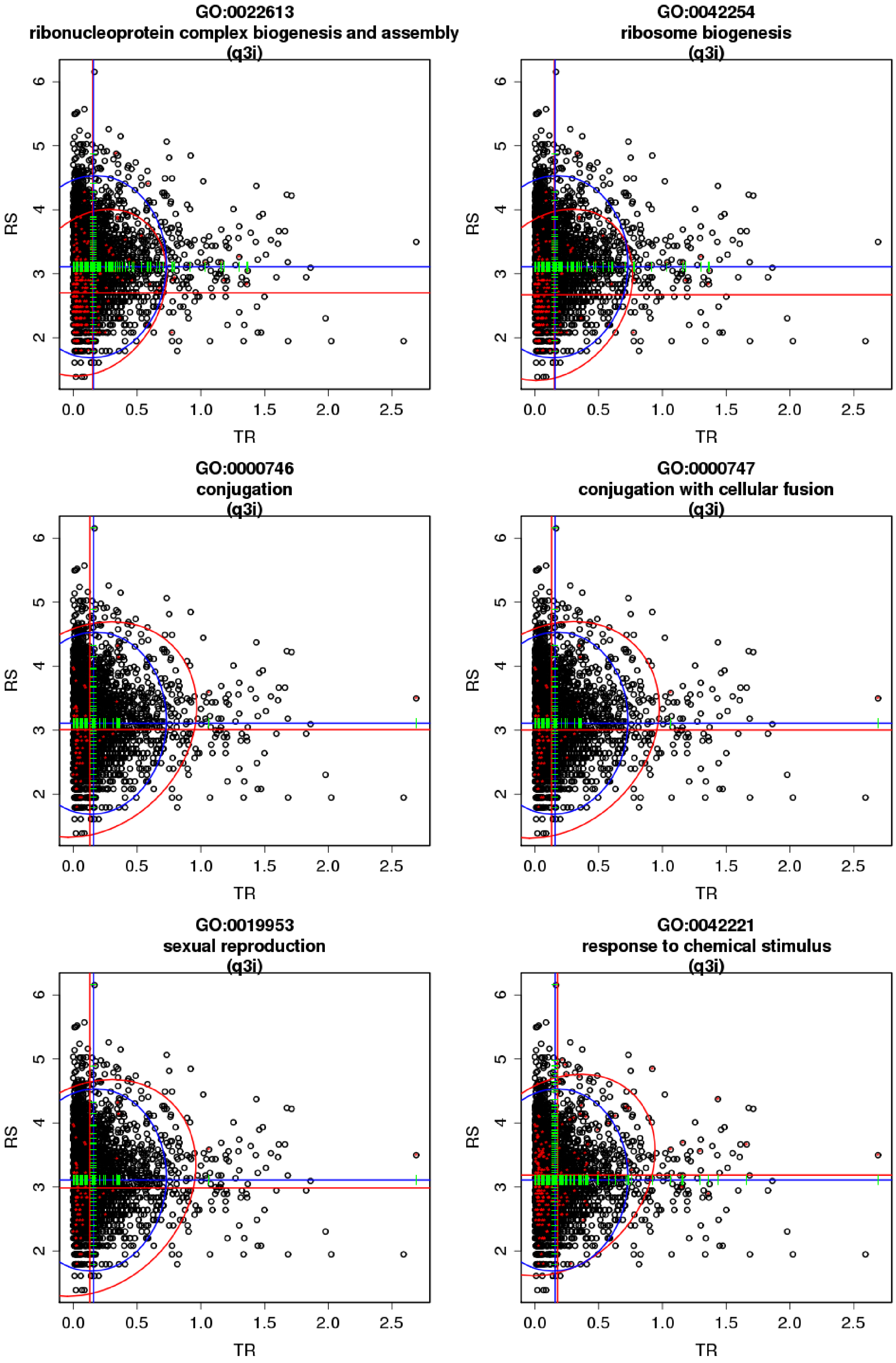
GO:0022613
ribonucleoprotein complex biogenesis and assembly
(q3i)
GO:0042254
ribosome biogenesis
(q3i)
GO:0000746
conjugation
(q3i)
GO:0000747
conjugation with cellular fusion
(q3i)
GO:0019953
sexual reproduction
(q3i)
GO:0042221
response to chemical stimulus
(q3i)
RS
TR
2
3
4
5
6
0.0
0.5
1.0
1.5
2.0
2.5

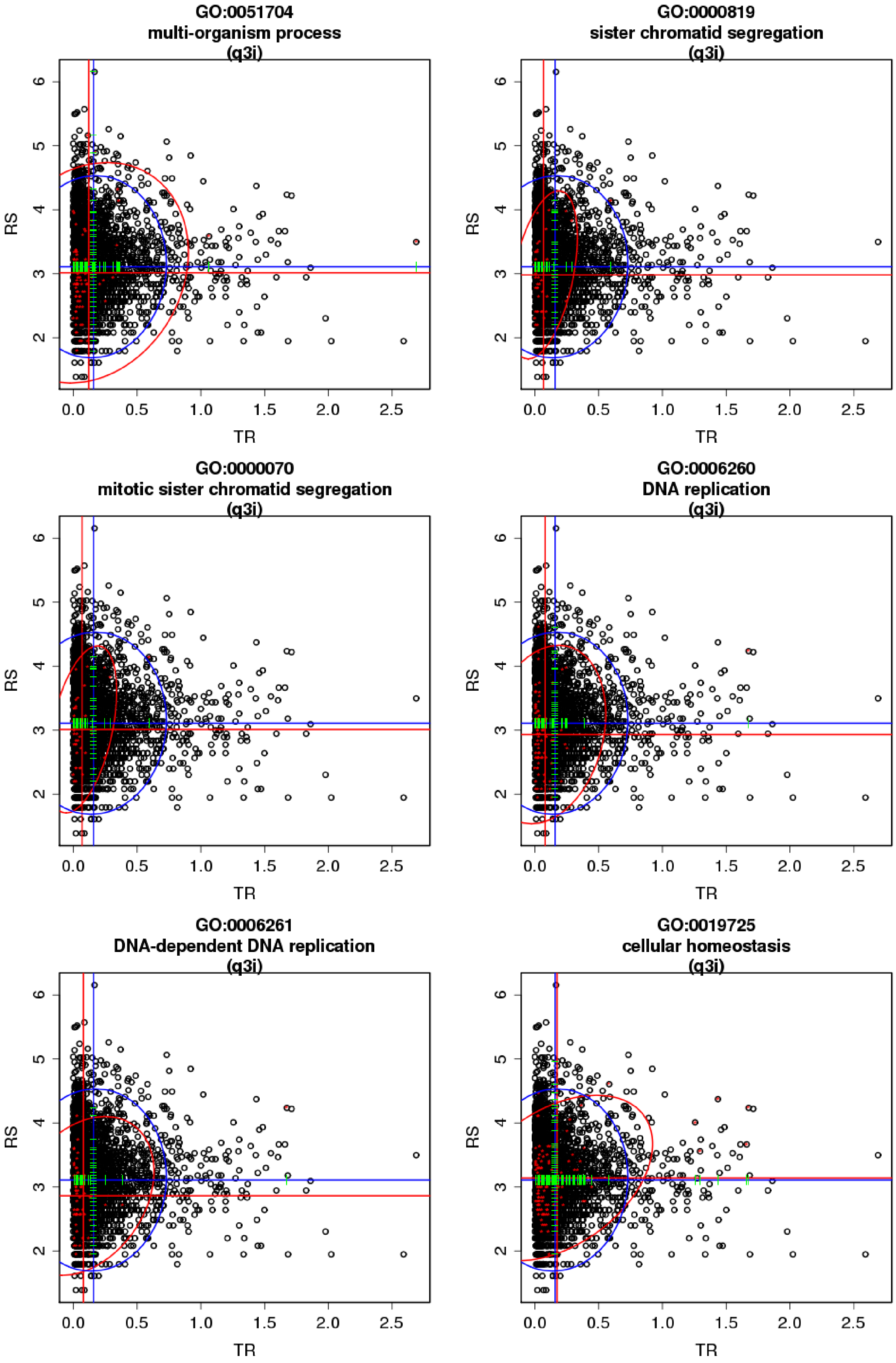
GO:0051704
multi-organism process
(q3i)
GO:0000819
sister chromatid segregation
(q3i)
GO:0000070
mitotic sister chromatid segregation
(q3i)
GO:0006260
DNA replication
(q3i)
GO:0006261
DNA-dependent DNA replication
(q3i)
GO:0019725
cellular homeostasis
(q3i)
RS
TR
0.0
0.5
1.0
1.5
2.0
2.5
2
3
4
5
6

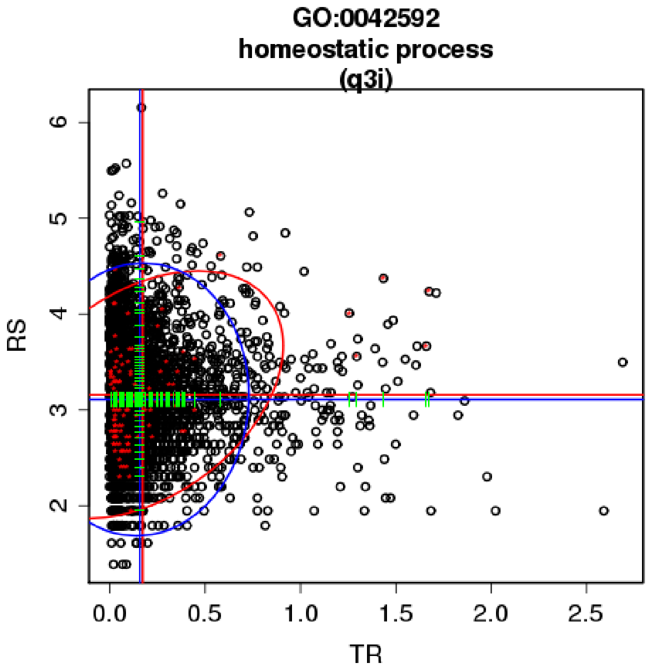
GO:0042592
homeostatic process
(q3i)
RS
TR
2
3
4
5
6
0.0
0.5
1.0
1.5
2.0
2.5

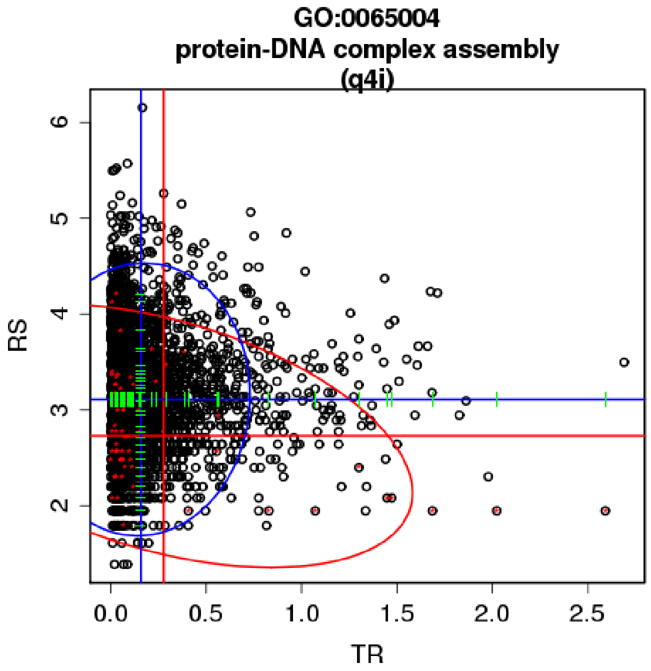
GO:0065004
protein-DNA complex assembly
(q4i)
RS
TR
2
3
4
5
6
0.0
0.5
1.0
1.5
2.0
2.5

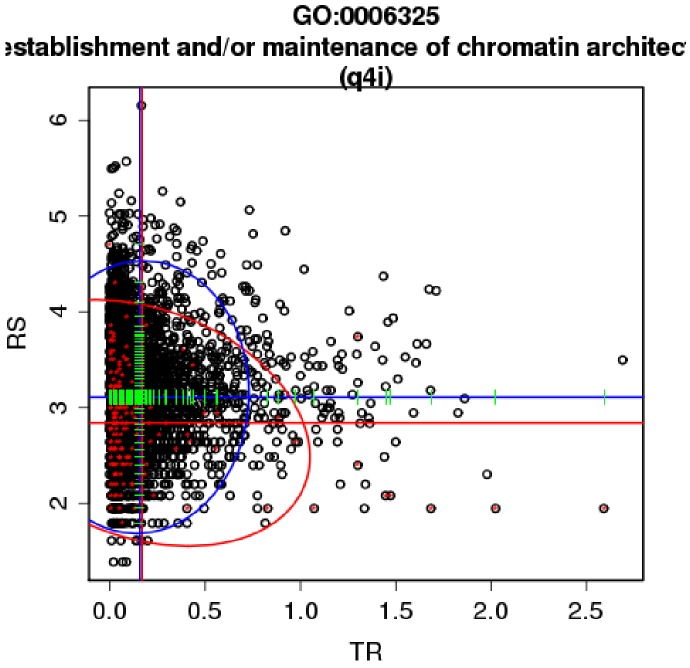
GO:0006325
establishment and/or maintenance of chromatin architec
(q4i)
RS
TR
2
3
4
5
6
0.0
0.5
1.0
1.5
2.0
2.5

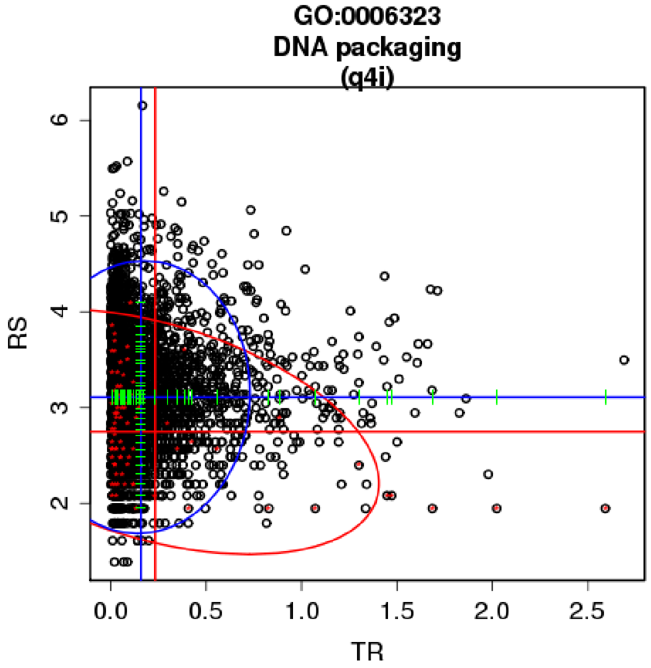
GO:0006323
DNA packaging
(q4i)
RS
TR
2
3
4
5
6
0.0
0.5
1.0
1.5
2.0
2.5

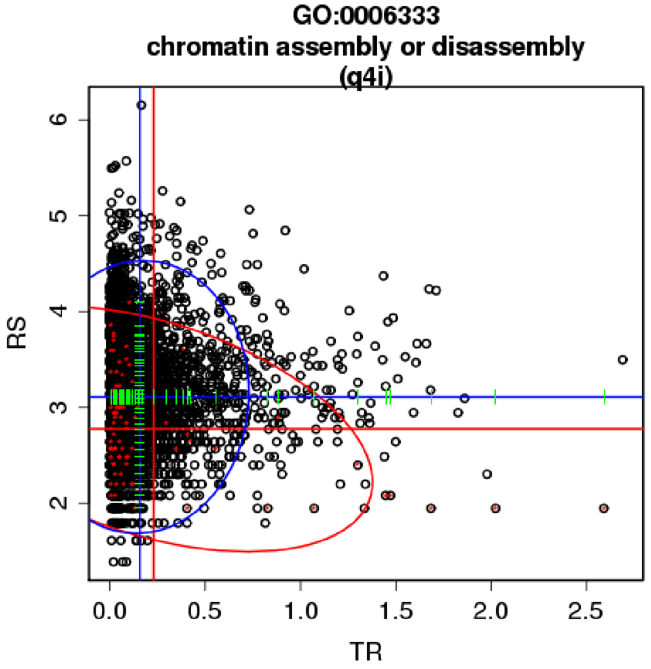
GO:0006333
chromatin assembly or disassembly
(q4i)
RS
TR
2
3
4
5
6
0.0
0.5
1.0
1.5
2.0
2.5

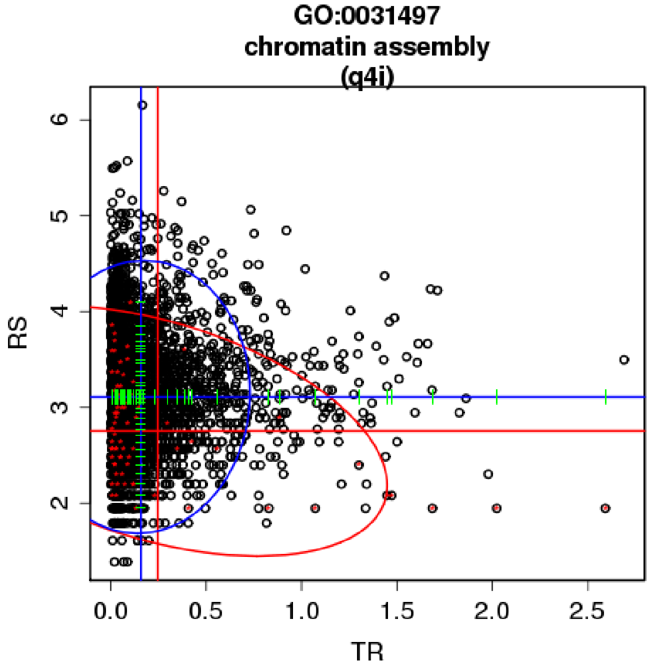
GO:0031497
chromatin assembly
(q4i)
RS
TR
2
3
4
5
6
0.0
0.5
1.0
1.5
2.0
2.5

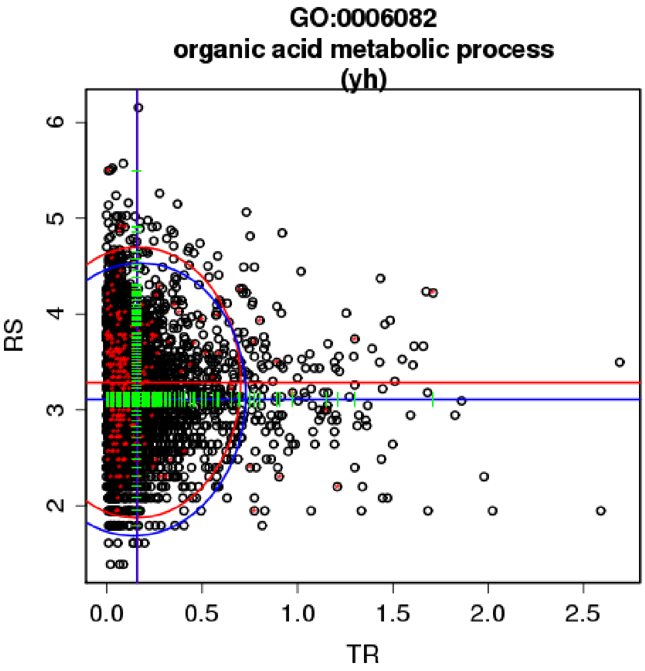
GO:0006082
organic acid metabolic process
(yh)
RS
TR
0.0 0.5 1.0 1.5 2.0 2.5
2 3 4 5 6

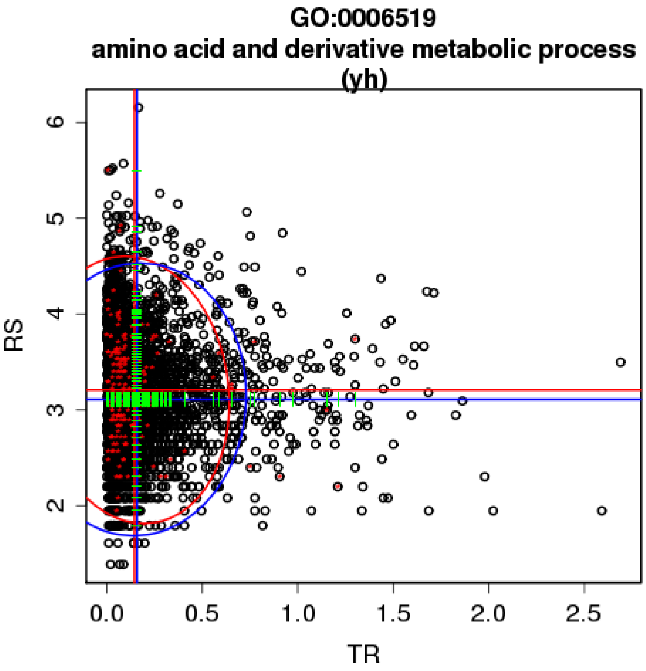
GO:0006519
amino acid and derivative metabolic process
(yh)
RS
TR
0.0 0.5 1.0 1.5 2.0 2.5
2 3 4 5 6

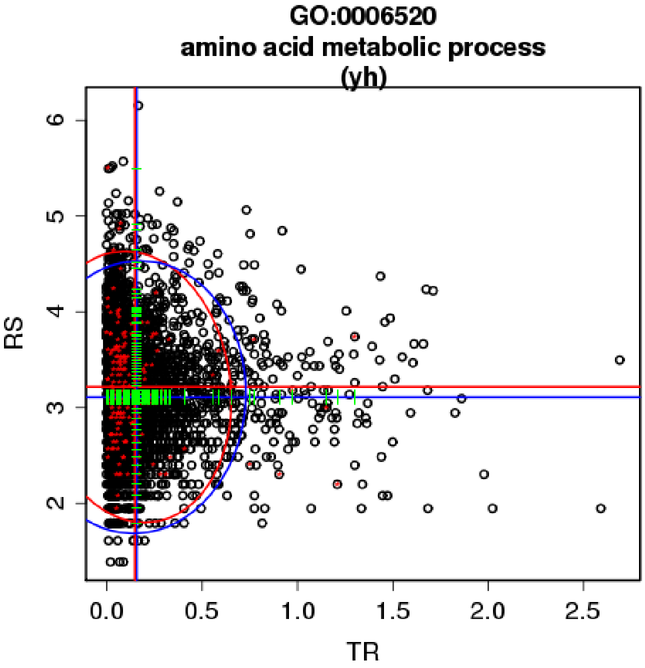
GO:0006520
amino acid metabolic process
(yh)
RS
TR
0.0 0.5 1.0 1.5 2.0 2.5
2 3 4 5 6

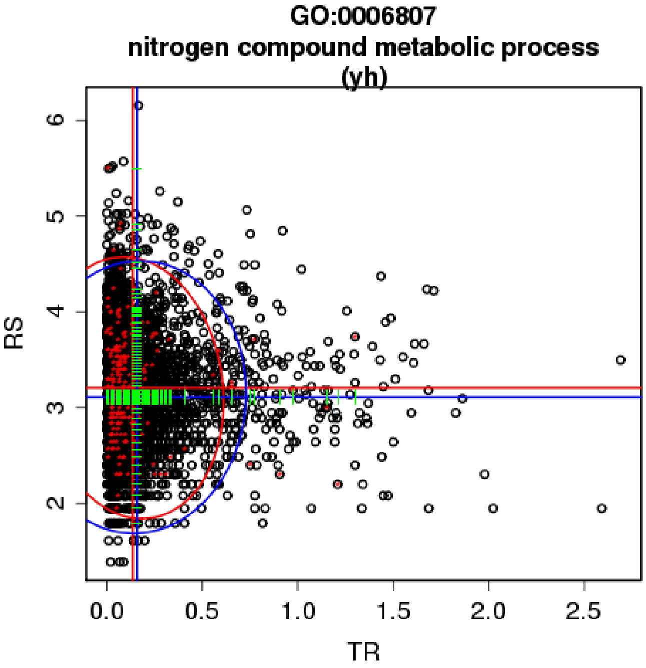
GO:0006807
nitrogen compound metabolic process
(yh)
RS
TR
0.0 0.5 1.0 1.5 2.0 2.5
2 3 4 5 6

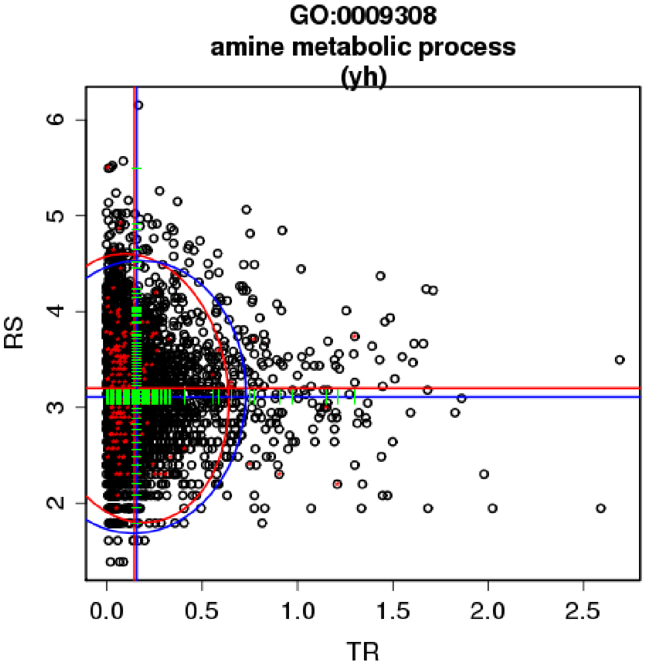
GO:0009308
amine metabolic process
(yh)
RS
TR
0.0 0.5 1.0 1.5 2.0 2.5
2 3 4 5 6

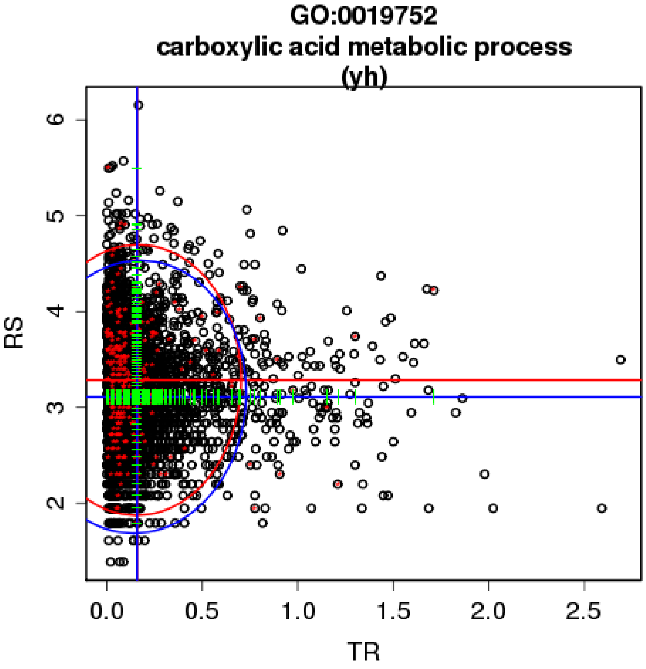
GO:0019752
carboxylic acid metabolic process
(yh)
RS
TR
0.0 0.5 1.0 1.5 2.0 2.5
2 3 4 5 6

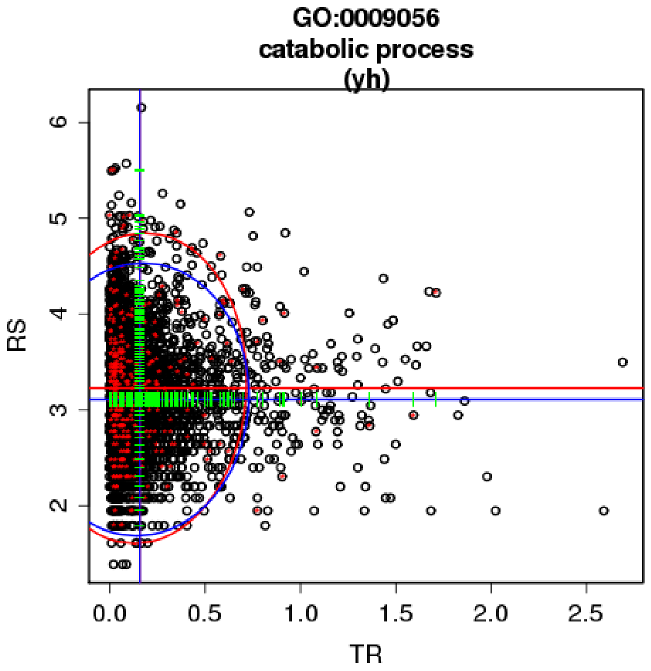
GO:0009056
catabolic process
(yh)
RS
TR
0.0 0.5 1.0 1.5 2.0 2.5
2 3 4 5 6

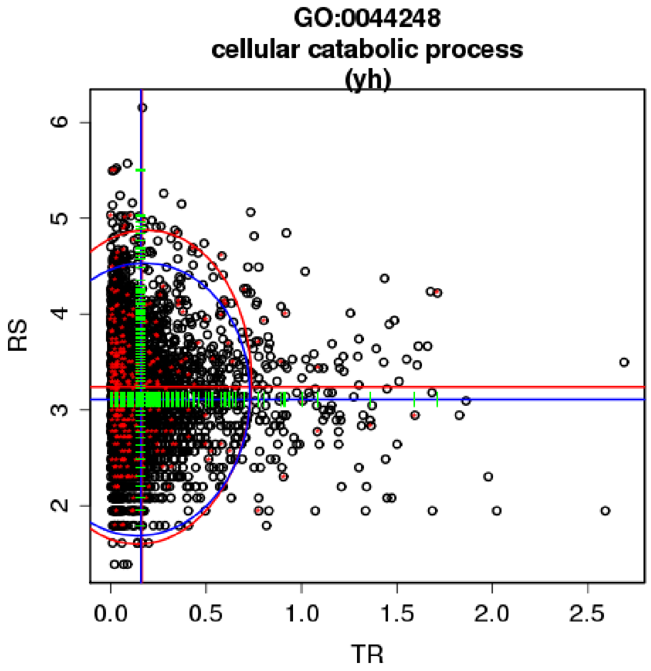
GO:0044248
cellular catabolic process
(yh)
RS
TR
0.0 0.5 1.0 1.5 2.0 2.5
2 3 4 5 6

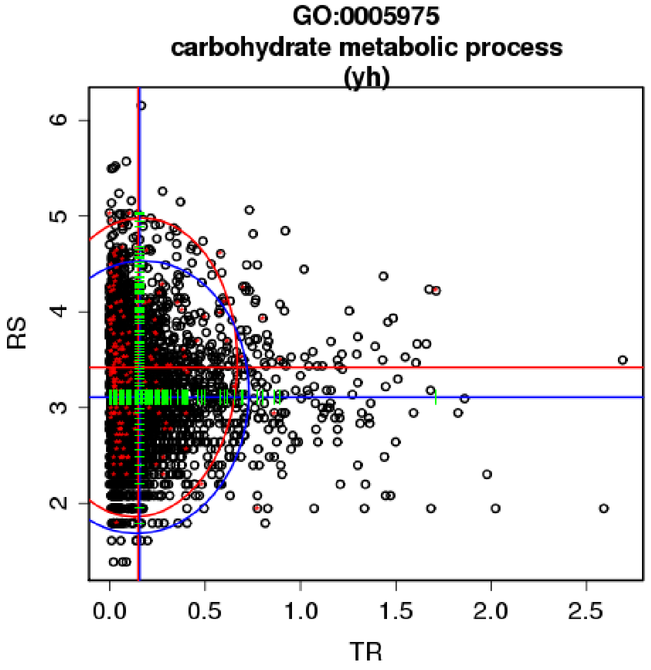
GO:0005975
carbohydrate metabolic process
(yh)
RS
TR
0.0 0.5 1.0 1.5 2.0 2.5
2 3 4 5 6

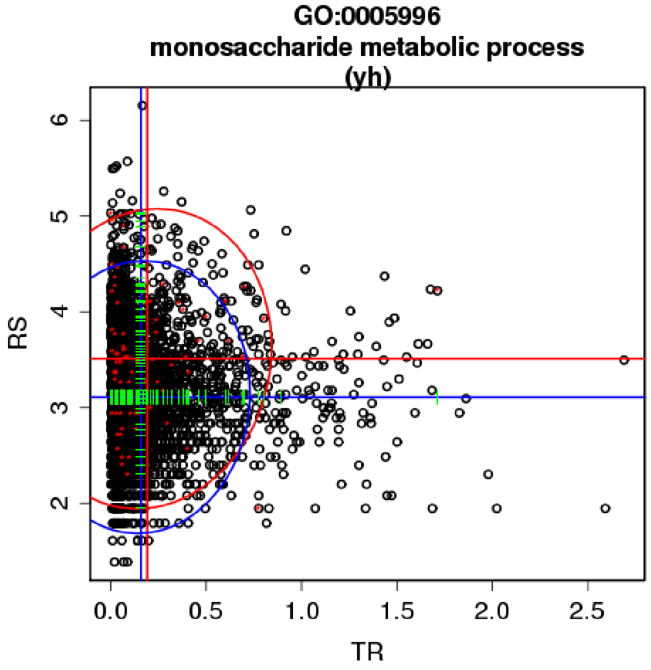
GO:0005996
monosaccharide metabolic process
(yh)
RS
TR
0.0 0.5 1.0 1.5 2.0 2.5
2 3 4 5 6

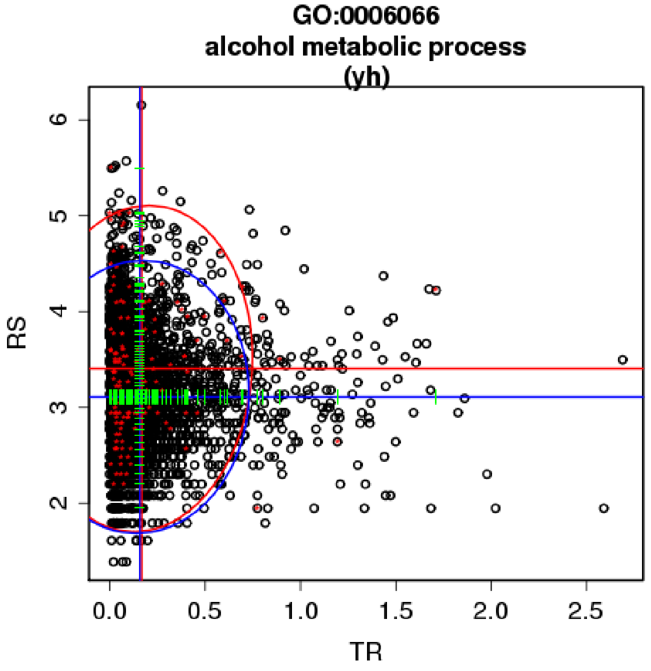
GO:0006066
alcohol metabolic process
(yh)
RS
TR
0.0 0.5 1.0 1.5 2.0 2.5
2 3 4 5 6

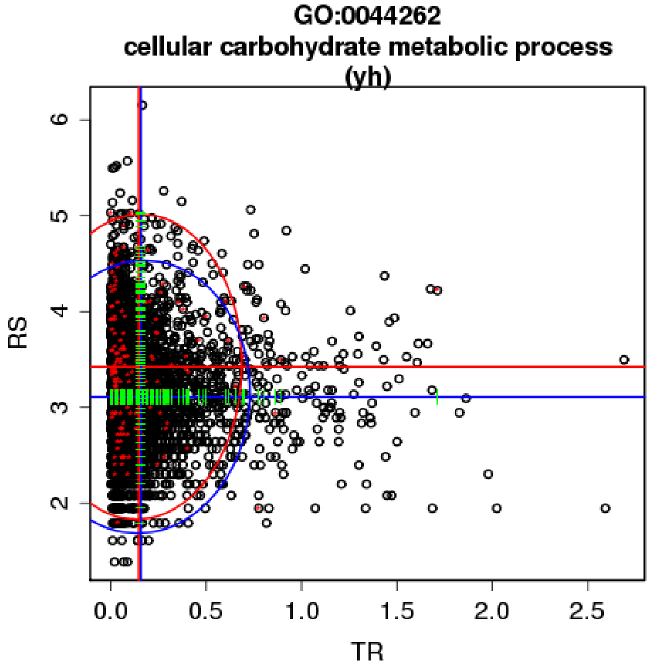
GO:0044262
cellular carbohydrate metabolic process
(yh)
RS
TR
0.0 0.5 1.0 1.5 2.0 2.5
2 3 4 5 6

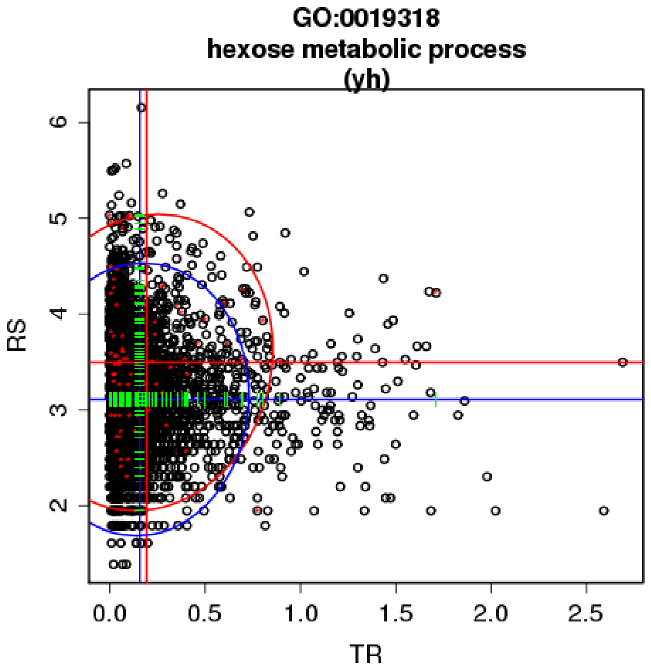
GO:0019318
hexose metabolic process
(yh)
RS
TR

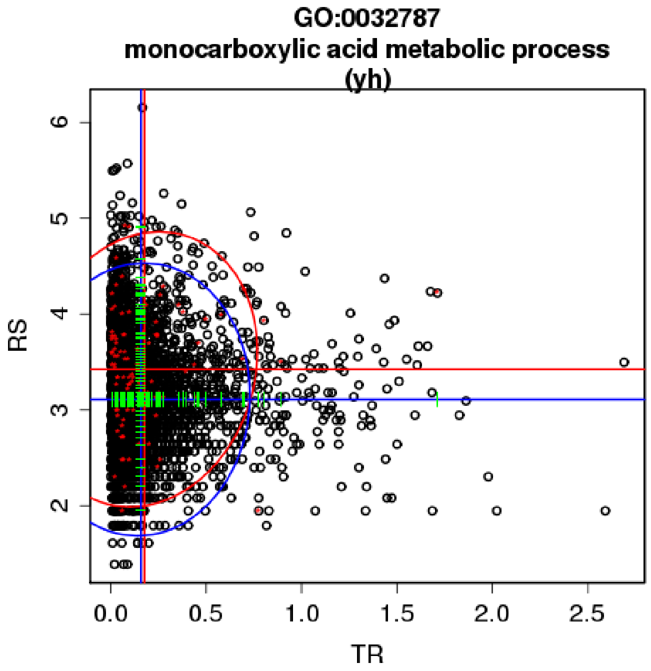
GO:0032787
monocarboxylic acid metabolic process
(yh)
RS
TR

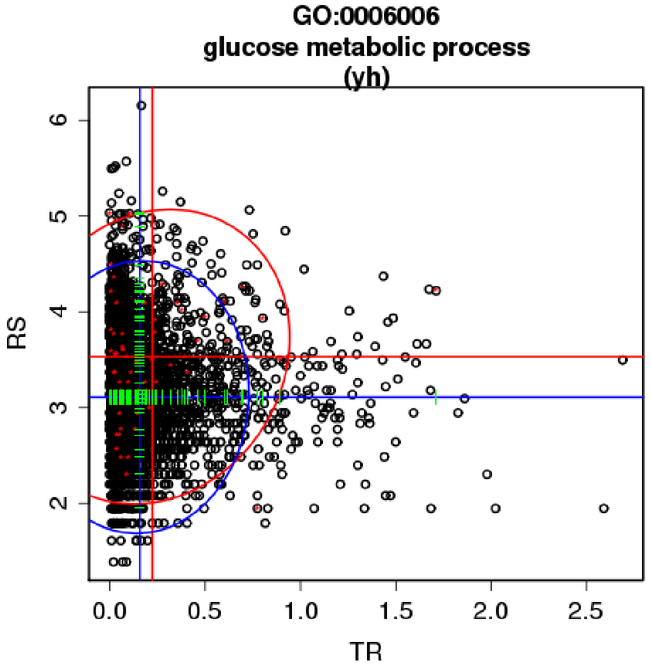
GO:0006006
glucose metabolic process
(yh)
RS
TR

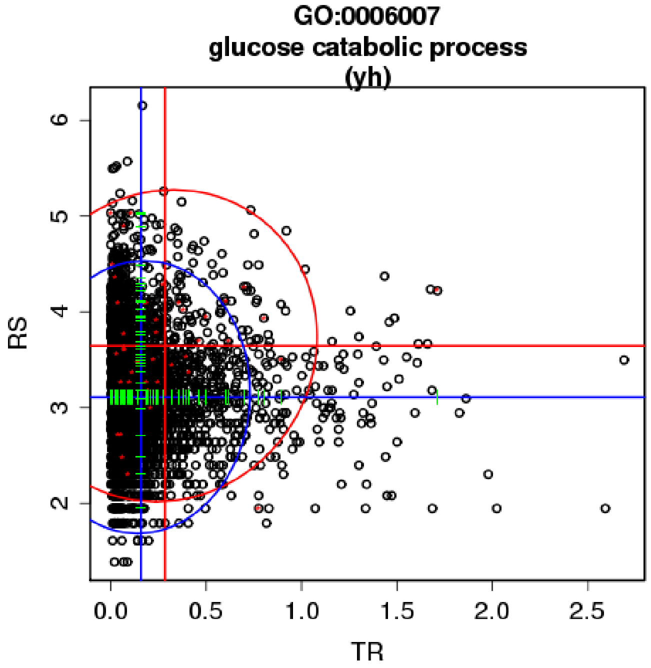
GO:0006007
glucose catabolic process
(yh)
RS
TR

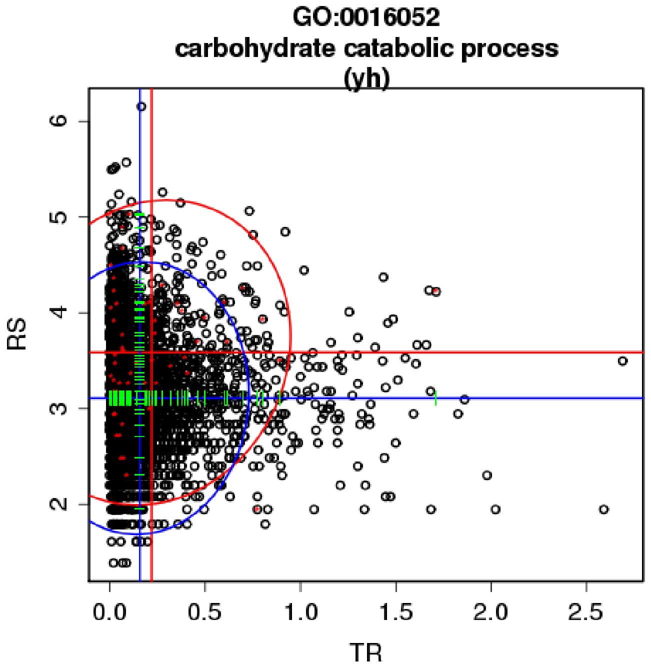
GO:0016052
carbohydrate catabolic process
(yh)
RS
TR

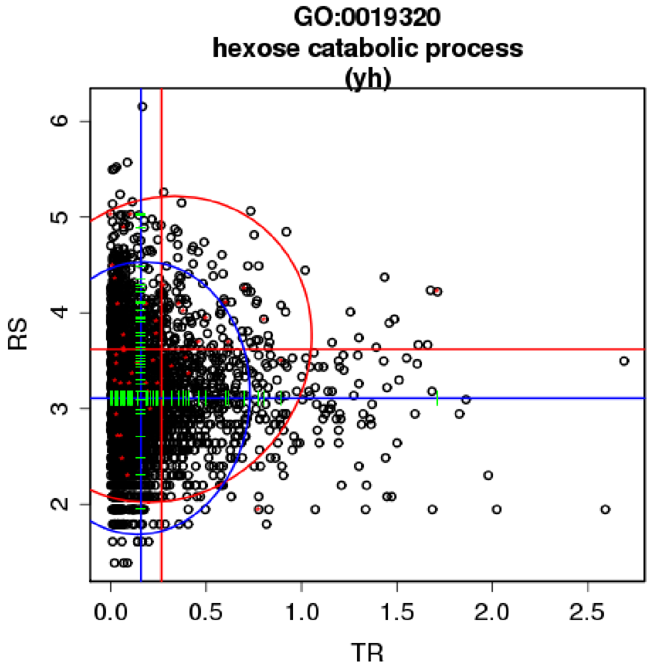
GO:0019320
hexose catabolic process
(yh)
RS
TR

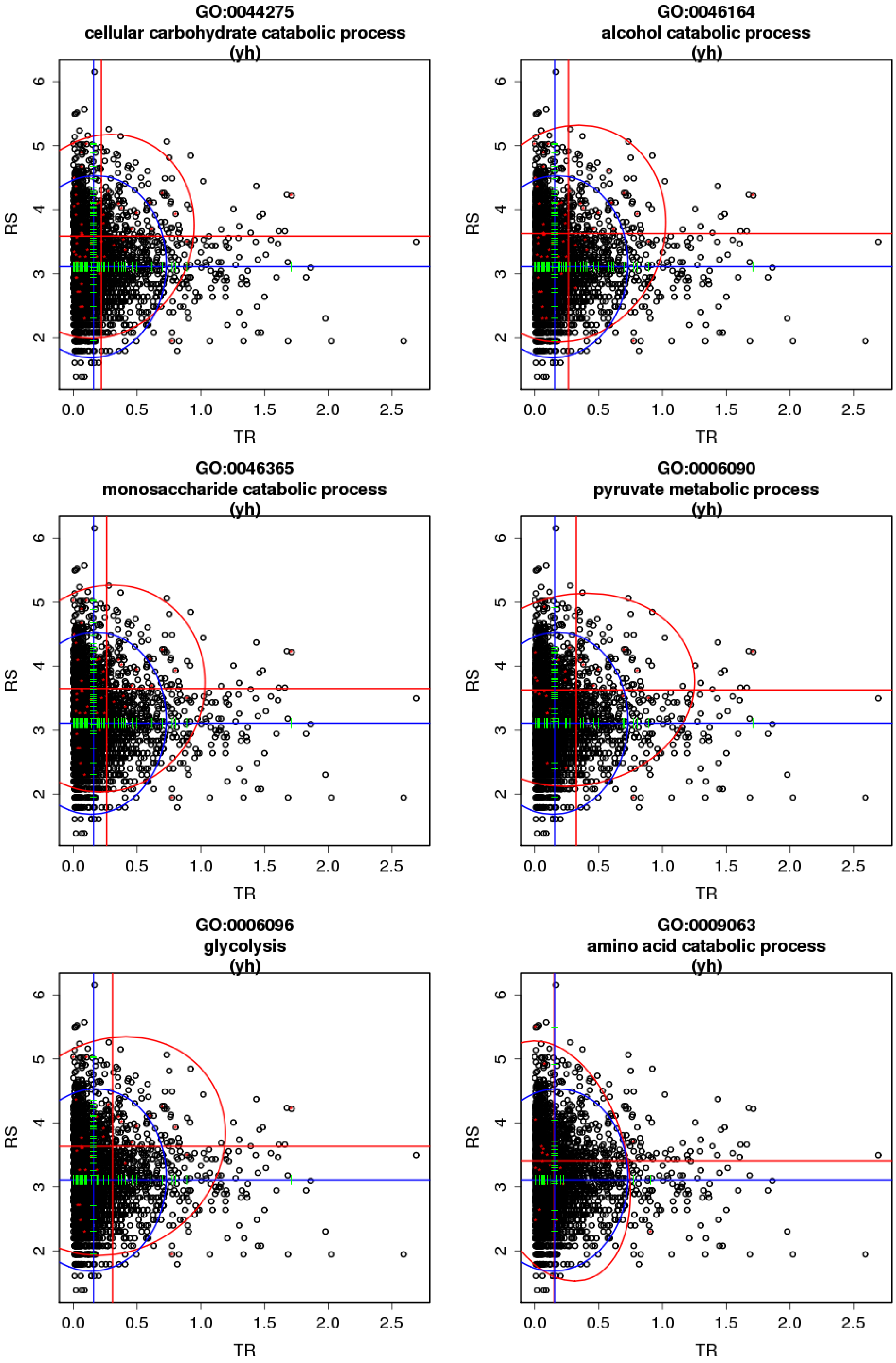
GO:0044275
cellular carbohydrate catabolic process
(yh)
GO:0046164
alcohol catabolic process
(yh)
GO:0046365
monosaccharide catabolic process
(yh)
GO:0006090
pyruvate metabolic process
(yh)
GO:0006096
glycolysis
(yh)
GO:0009063
amino acid catabolic process
(yh)
RS
TR
0.0
0.5
1.0
1.5
2.0
2.5
2
3
4
5
6

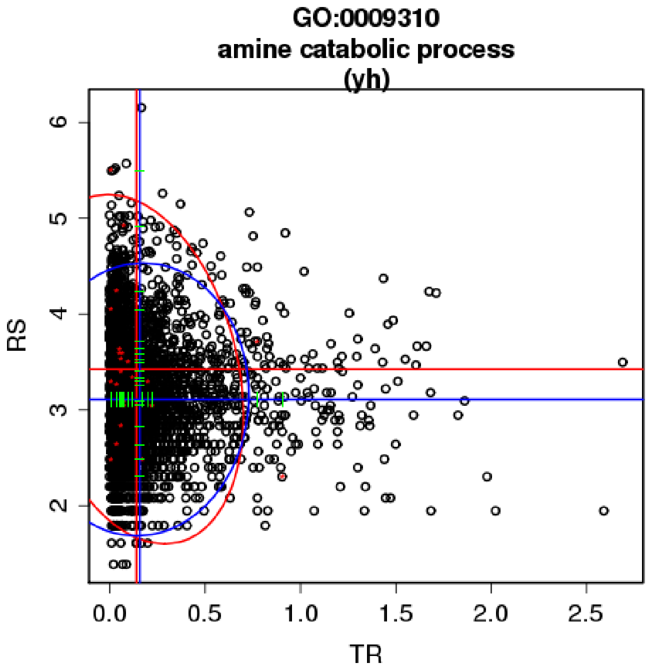
GO:0009310
amine catabolic process
(yh)
RS
TR
0.0 0.5 1.0 1.5 2.0 2.5
2 3 4 5 6

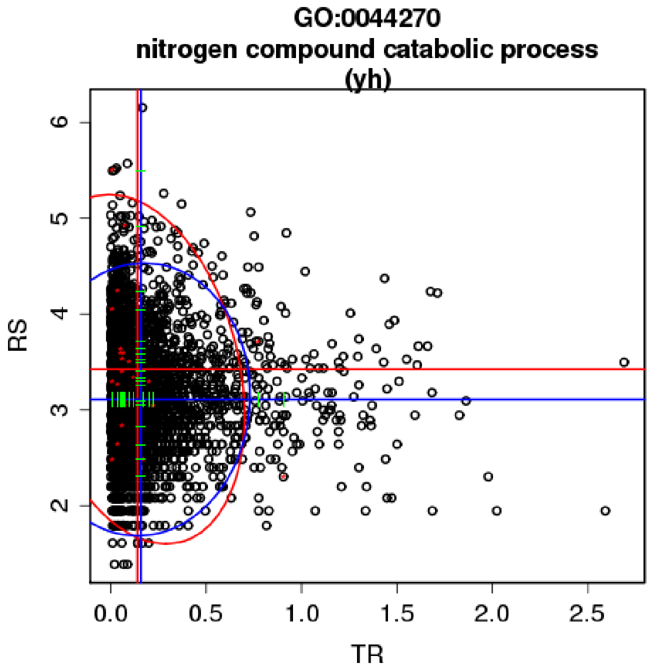
GO:0044270
nitrogen compound catabolic process
(yh)
RS
TR
0.0 0.5 1.0 1.5 2.0 2.5
2 3 4 5 6

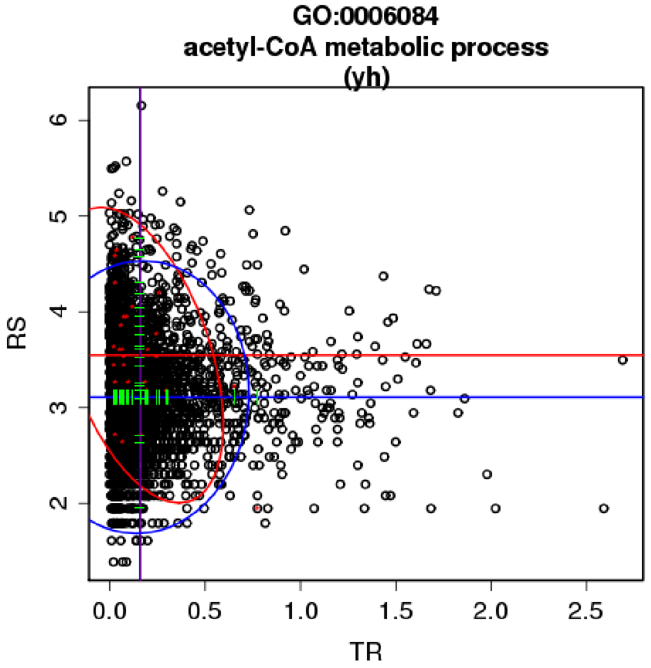
GO:0006084
acetyl-CoA metabolic process
(yh)
RS
TR
0.0 0.5 1.0 1.5 2.0 2.5
2 3 4 5 6

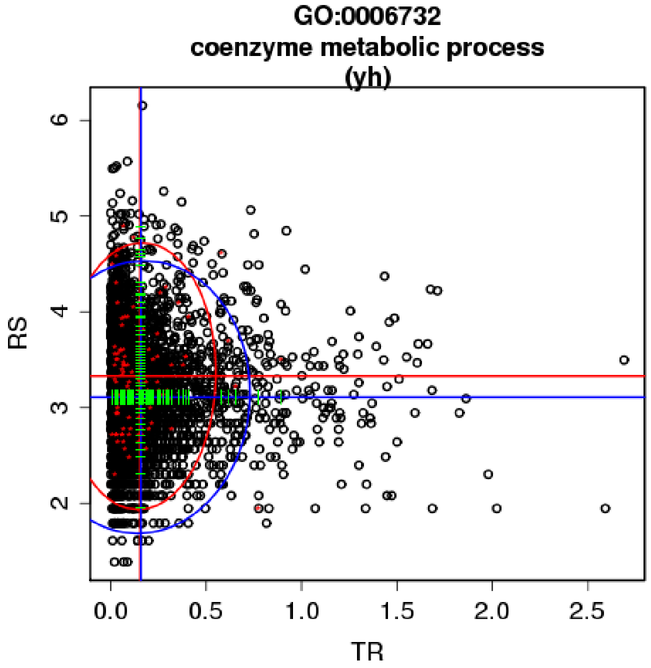
GO:0006732
coenzyme metabolic process
(yh)
RS
TR
0.0 0.5 1.0 1.5 2.0 2.5
2 3 4 5 6

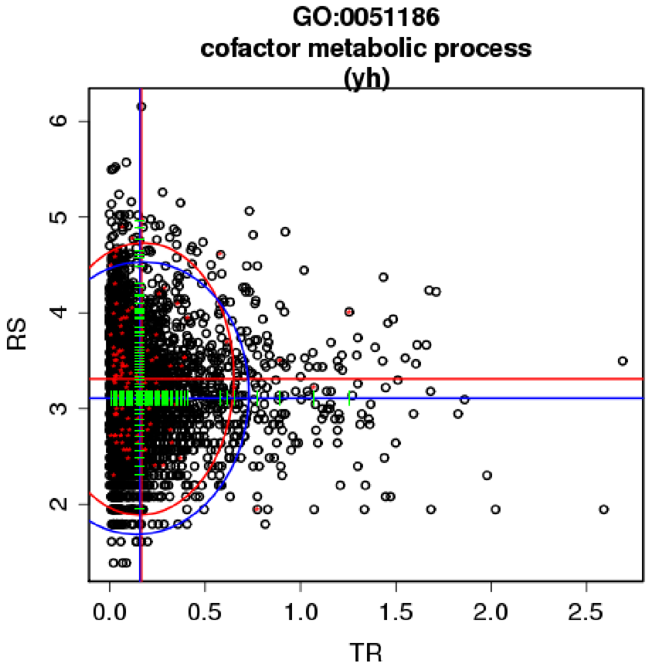
GO:0051186
cofactor metabolic process
(yh)
RS
TR
0.0 0.5 1.0 1.5 2.0 2.5
2 3 4 5 6

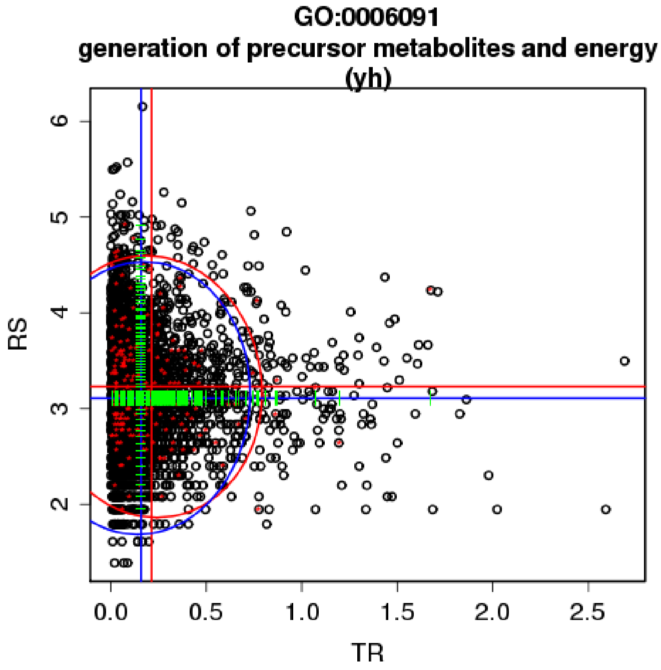
GO:0006091
generation of precursor metabolites and energy
(yh)
RS
TR
0.0 0.5 1.0 1.5 2.0 2.5
2 3 4 5 6

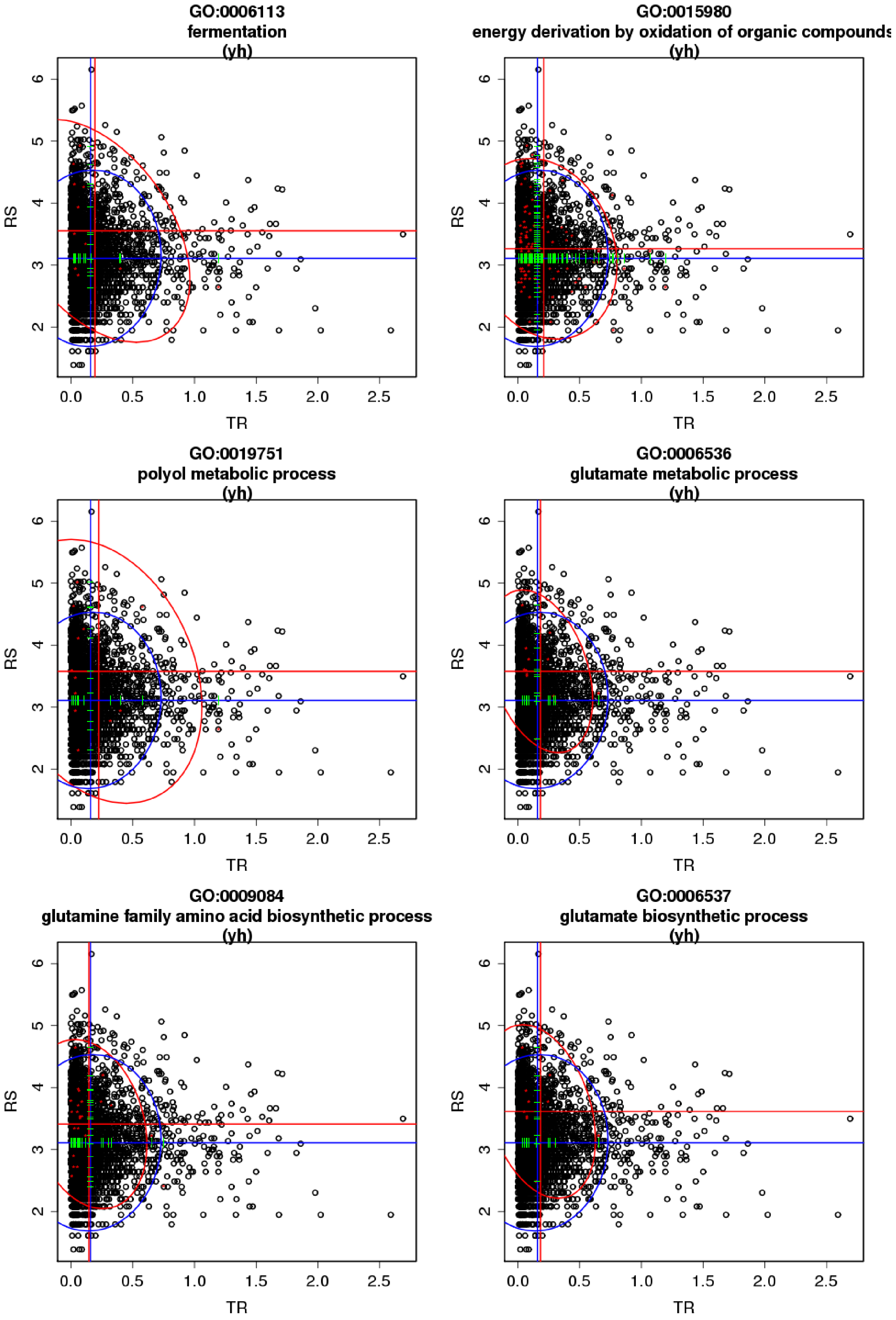
GO:0006113
fermentation
(yh)
GO:0015980
energy derivation by oxidation of organic compounds
(yh)
GO:0019751
polyol metabolic process
(yh)
GO:0006536
glutamate metabolic process
(yh)
GO:0009084
glutamine family amino acid biosynthetic process
(yh)
GO:0006537
glutamate biosynthetic process
(yh)
RS
TR
0.0
0.5
1.0
1.5
2.0
2.5
2
3
4
5
6

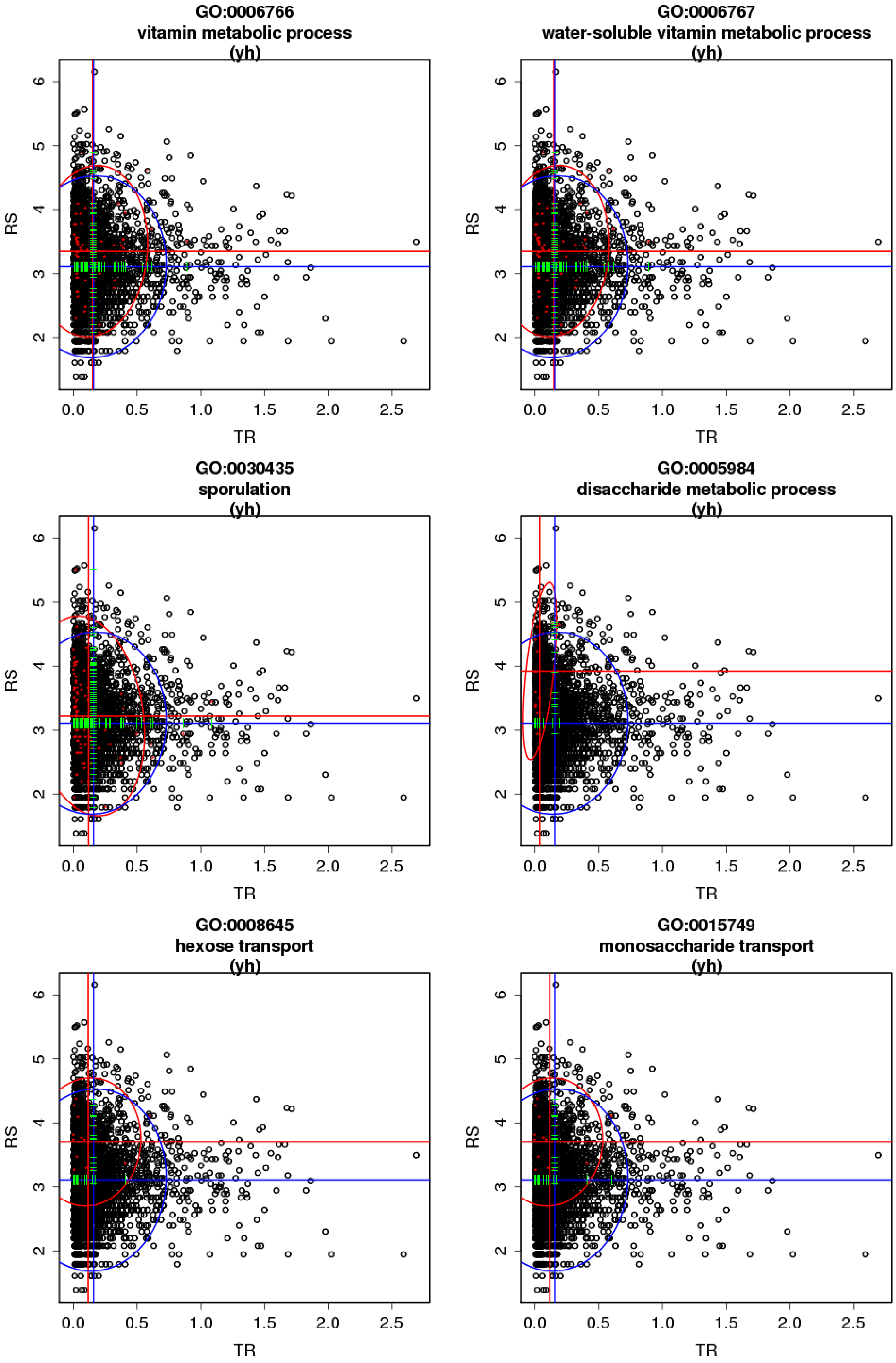
GO:0006766
vitamin metabolic process
(yh)
GO:0006767
water-soluble vitamin metabolic process
(yh)
GO:0030435
sporulation
(yh)
GO:0005984
disaccharide metabolic process
(yh)
GO:0008645
hexose transport
(yh)
GO:0015749
monosaccharide transport
(yh)
RS
TR
0.0
0.5
1.0
1.5
2.0
2.5
2
3
4
5
6

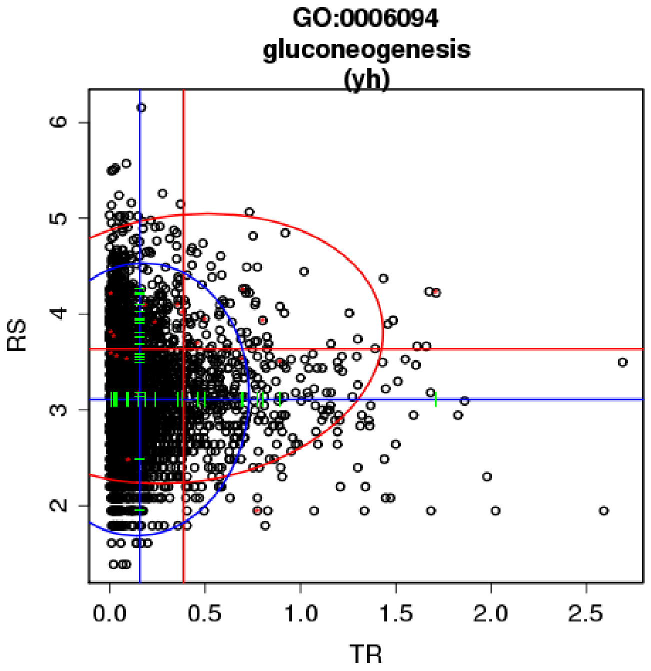
GO:0006094
gluconeogenesis
(yh)
RS
TR
0.0 0.5 1.0 1.5 2.0 2.5
2 3 4 5 6

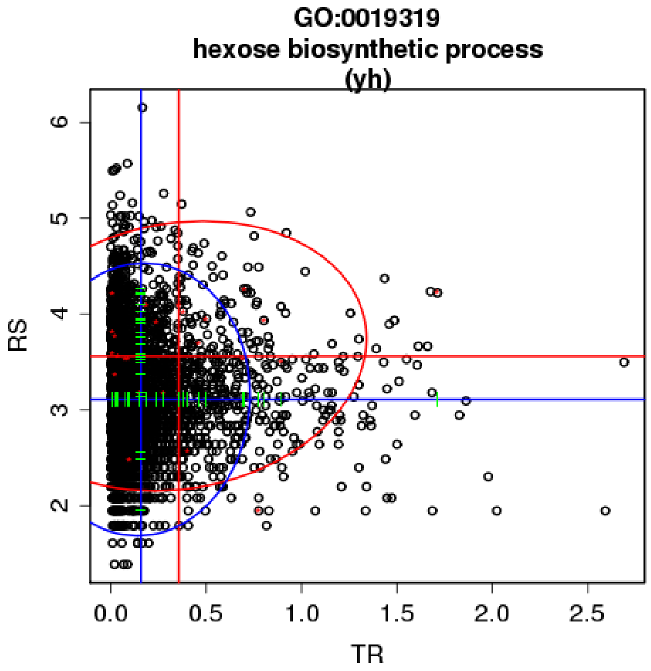
GO:0019319
hexose biosynthetic process
(yh)
RS
TR
0.0 0.5 1.0 1.5 2.0 2.5
2 3 4 5 6

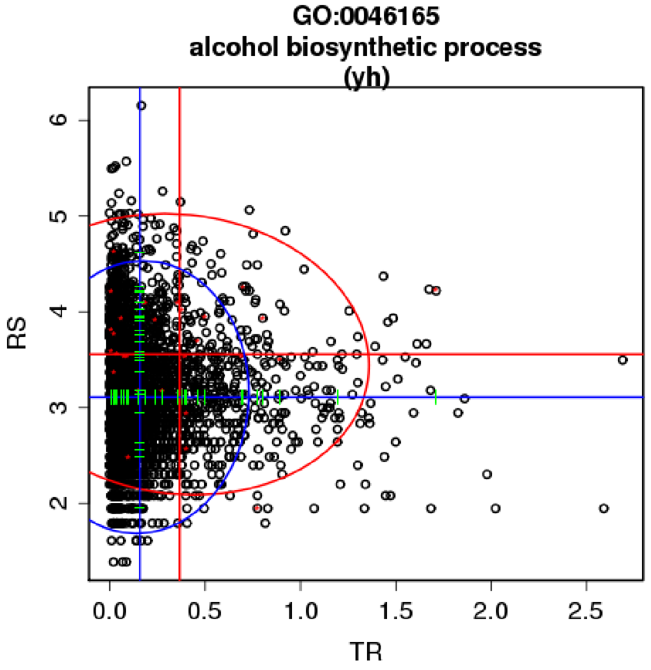
GO:0046165
alcohol biosynthetic process
(yh)
RS
TR
0.0 0.5 1.0 1.5 2.0 2.5
2 3 4 5 6

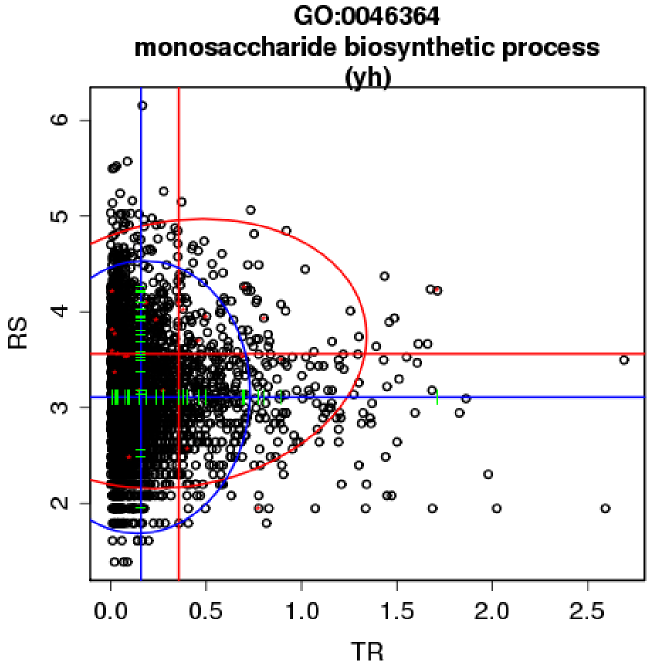
GO:0046364
monosaccharide biosynthetic process
(yh)
RS
TR
0.0 0.5 1.0 1.5 2.0 2.5
2 3 4 5 6

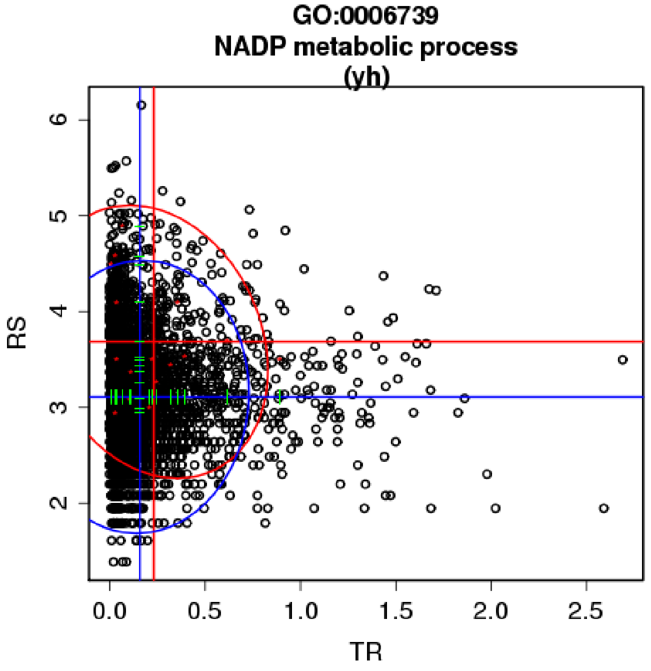
GO:0006739
NADP metabolic process
(yh)
RS
TR
0.0 0.5 1.0 1.5 2.0 2.5
2 3 4 5 6

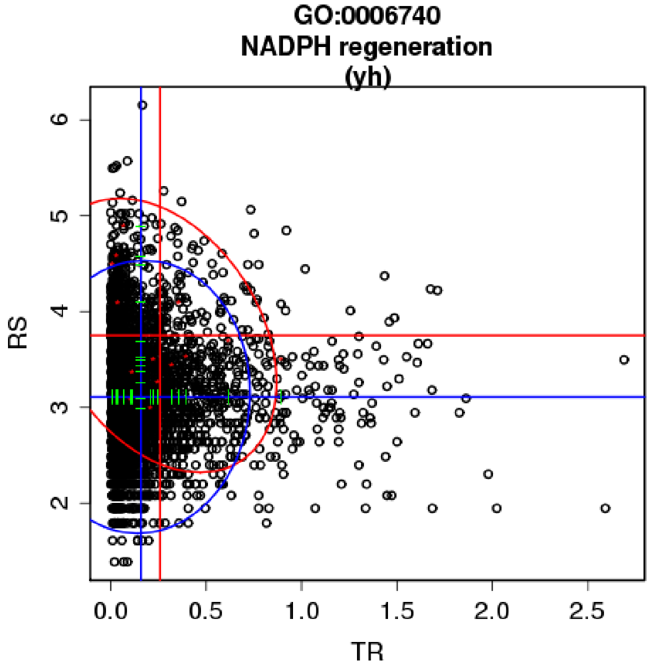
GO:0006740
NADPH regeneration
(yh)
RS
TR
0.0 0.5 1.0 1.5 2.0 2.5
2 3 4 5 6

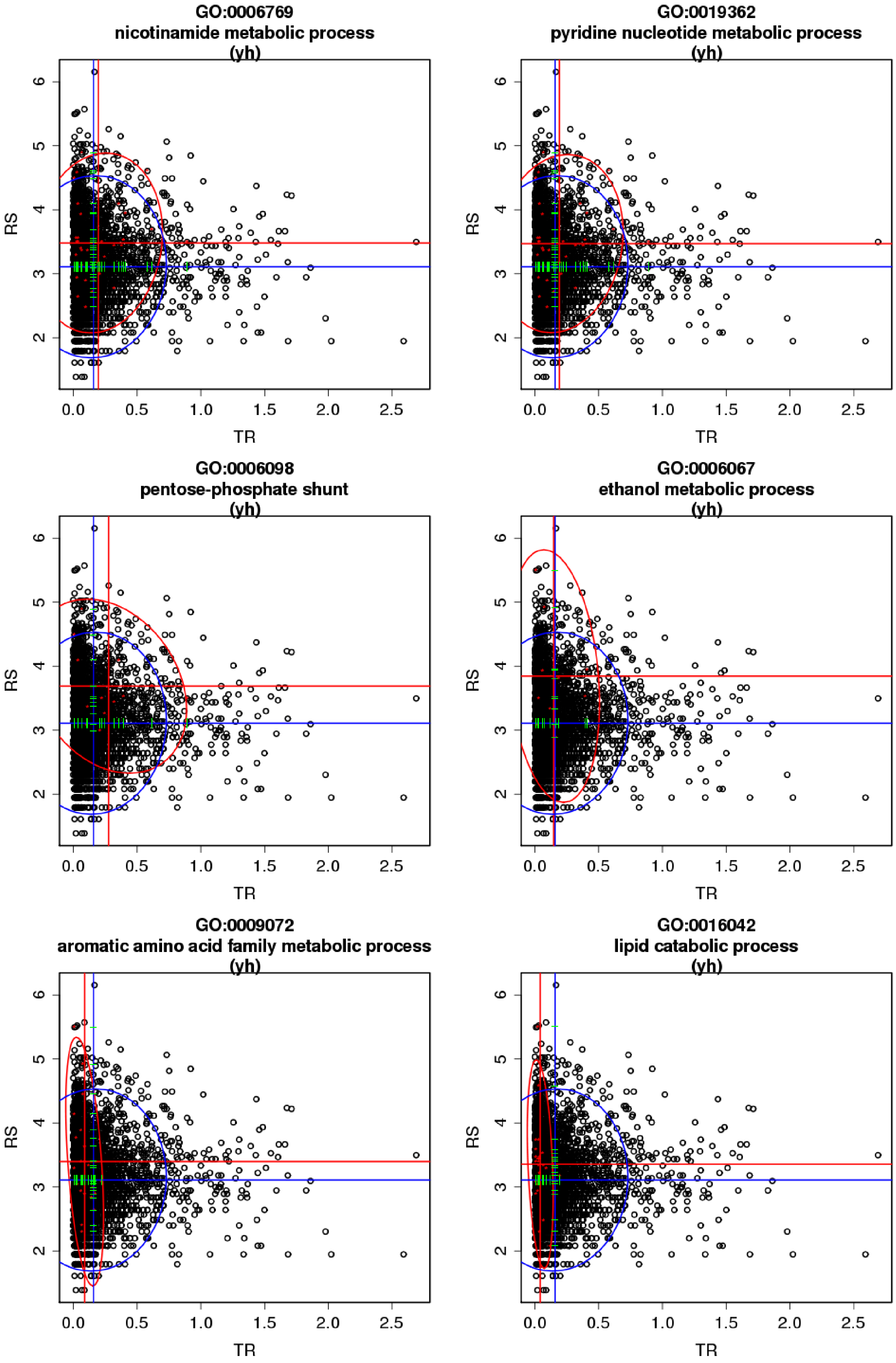

GO:0006769
nicotinamide metabolic process
(yh)
GO:0019362
pyridine nucleotide metabolic process
(yh)
GO:0006098
pentose-phosphate shunt
(yh)
GO:0006067
ethanol metabolic process
(yh)
GO:0009072
aromatic amino acid family metabolic process
(yh)
GO:0016042
lipid catabolic process
(yh)
RS
TR
0.0
0.5
1.0
1.5
2.0
2.5
2
3
4
5
6

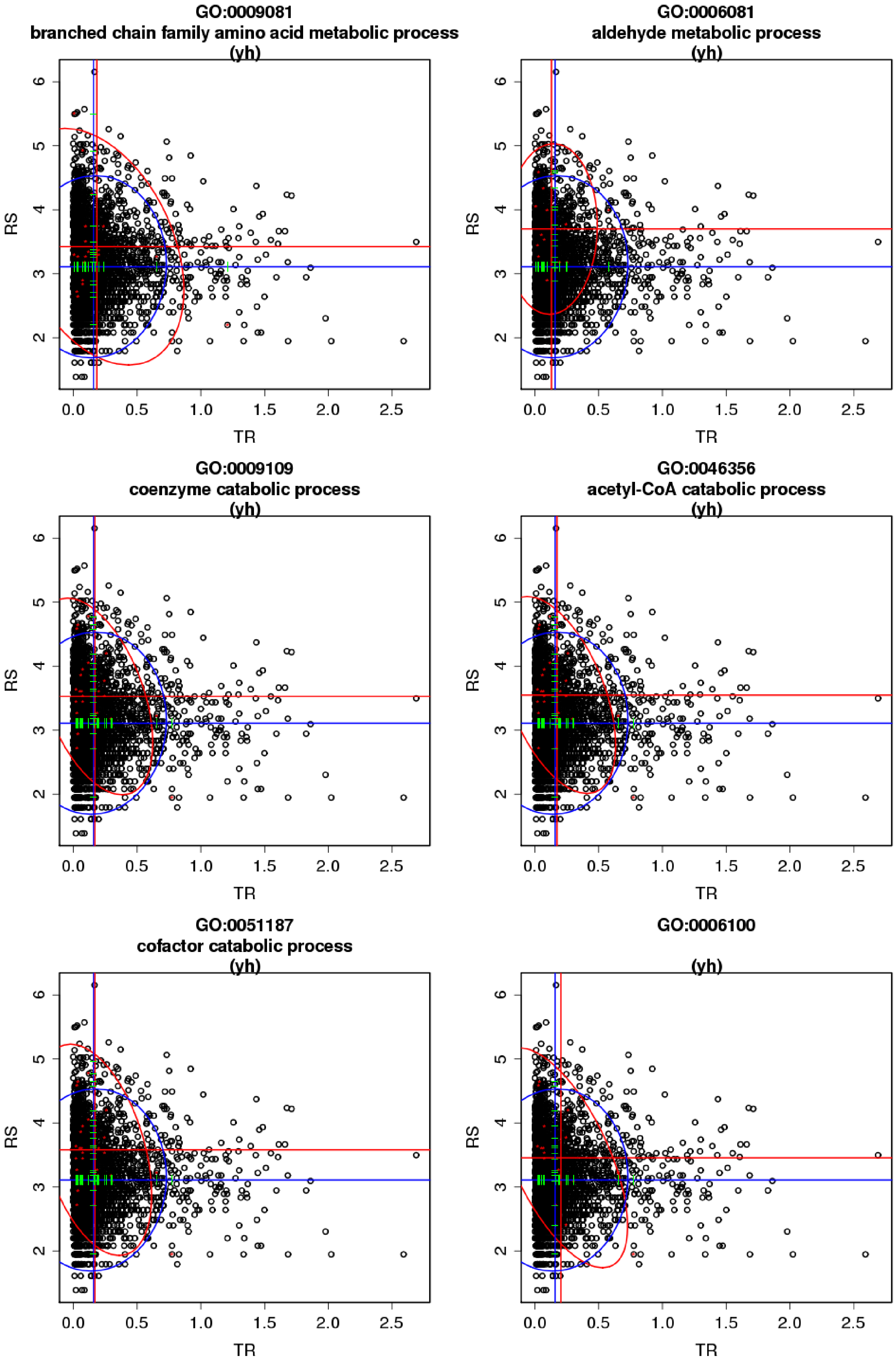
GO:0009081
branched chain family amino acid metabolic process
(yh)
GO:0006081
aldehyde metabolic process
(yh)
GO:0009109
coenzyme catabolic process
(yh)
GO:0046356
acetyl-CoA catabolic process
(yh)
GO:0051187
cofactor catabolic process
(yh)
GO:0006100
(yh)
RS
TR
0.0
0.5
1.0
1.5
2.0
2.5
2
3
4
5
6

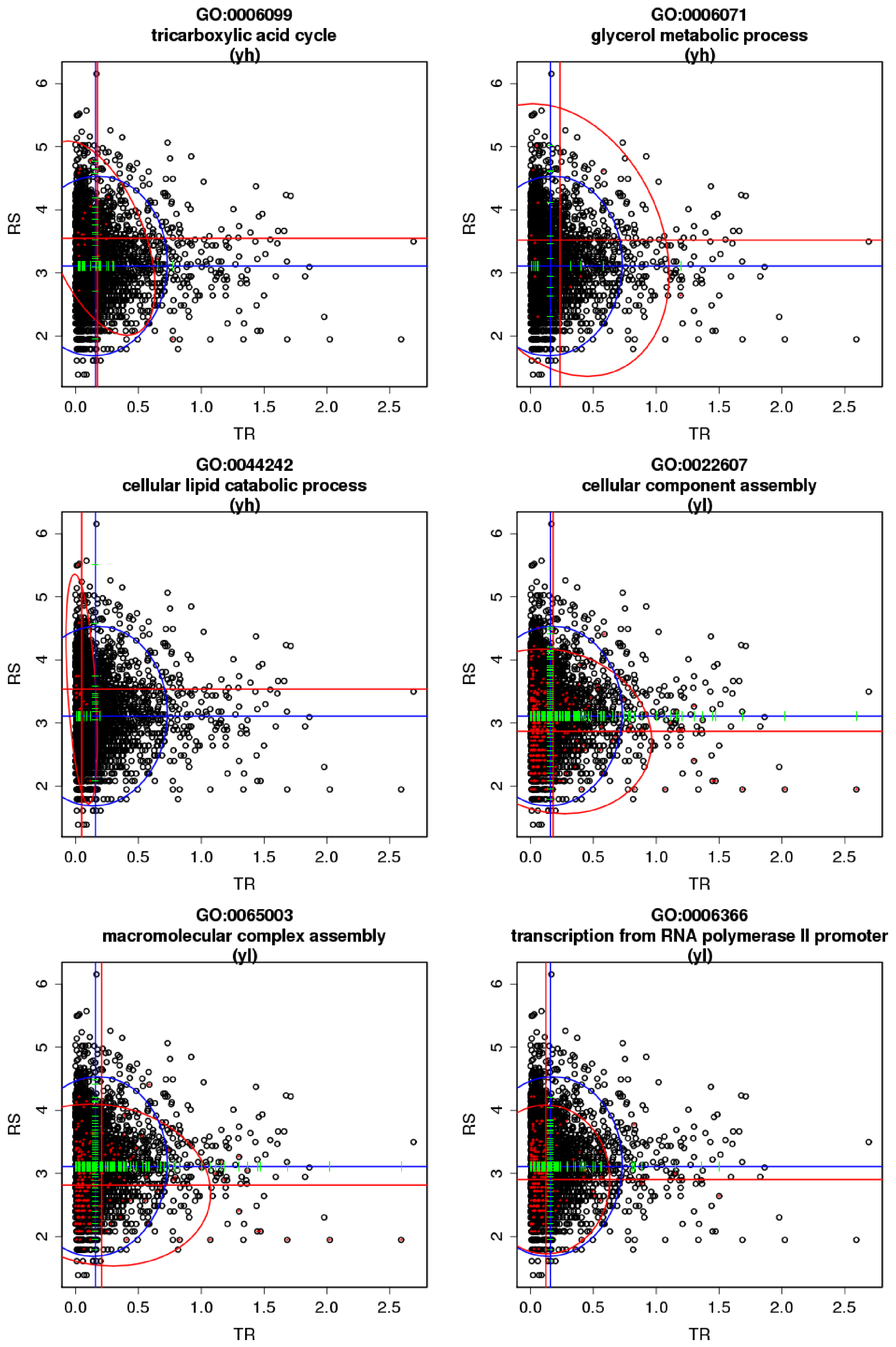
GO:0006099
tricarboxylic acid cycle
(yh)
GO:0006071
glycerol metabolic process
(yh)
GO:0044242
cellular lipid catabolic process
(yh)
GO:0022607
cellular component assembly
(yl)
GO:0065003
macromolecular complex assembly
(yl)
GO:0006366
transcription from RNA polymerase II promoter
(yl)
RS
TR
0.0
0.5
1.0
1.5
2.0
2.5
2
3
4
5
6

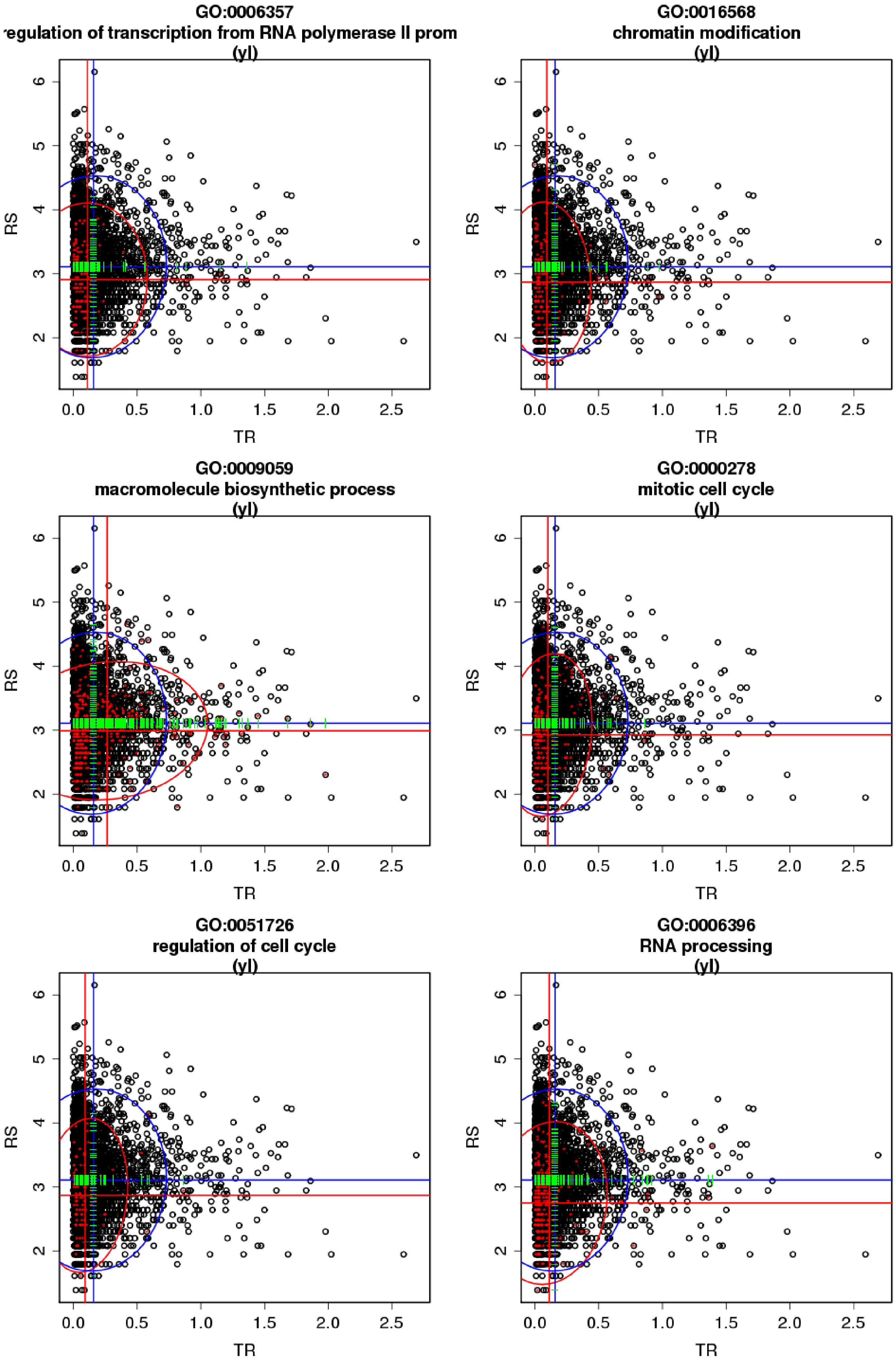
GO:0006357
regulation of transcription from RNA polymerase II prom
(yl)
GO:0016568
chromatin modification
(yl)
GO:0009059
macromolecule biosynthetic process
(yl)
GO:0000278
mitotic cell cycle
(yl)
GO:0051726
regulation of cell cycle
(yl)
GO:0006396
RNA processing
(yl)
RS
TR
2
3
4
5
6
0.0
0.5
1.0
1.5
2.0
2.5

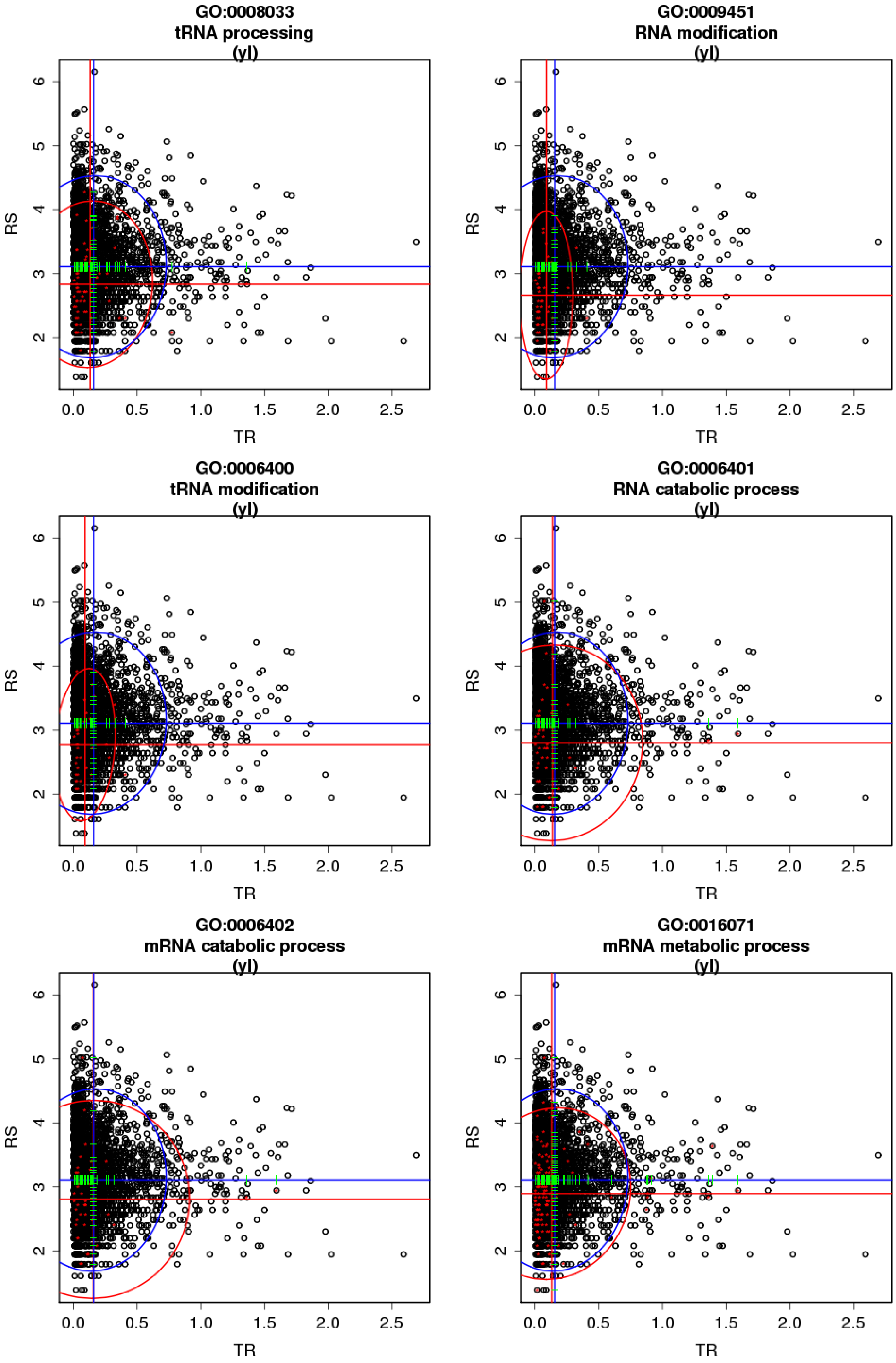

GO:0008033
tRNA processing
(yl)
GO:0009451
RNA modification
(yl)
GO:0006400
tRNA modification
(yl)
GO:0006401
RNA catabolic process
(yl)
GO:0006402
mRNA catabolic process
(yl)
GO:0016071
mRNA metabolic process
(yl)
RS
TR

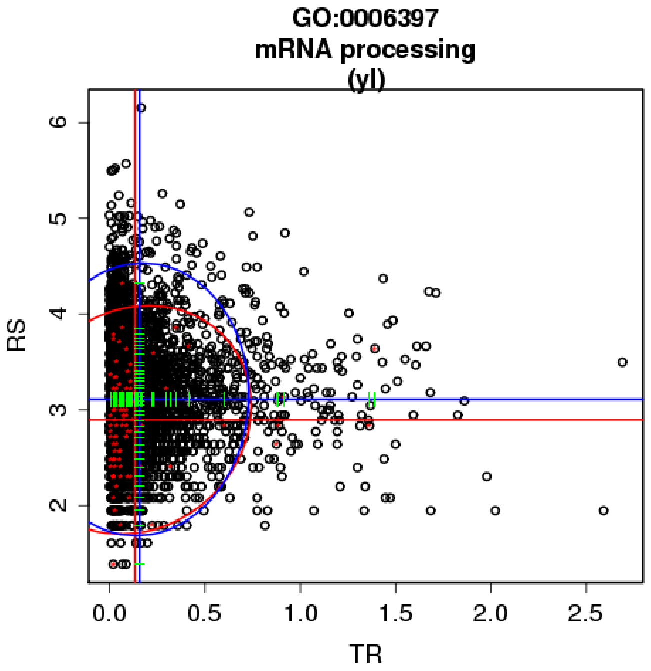
GO:0006397
mRNA processing
(yl)
RS
TR
0.0 0.5 1.0 1.5 2.0 2.5
2 3 4 5 6

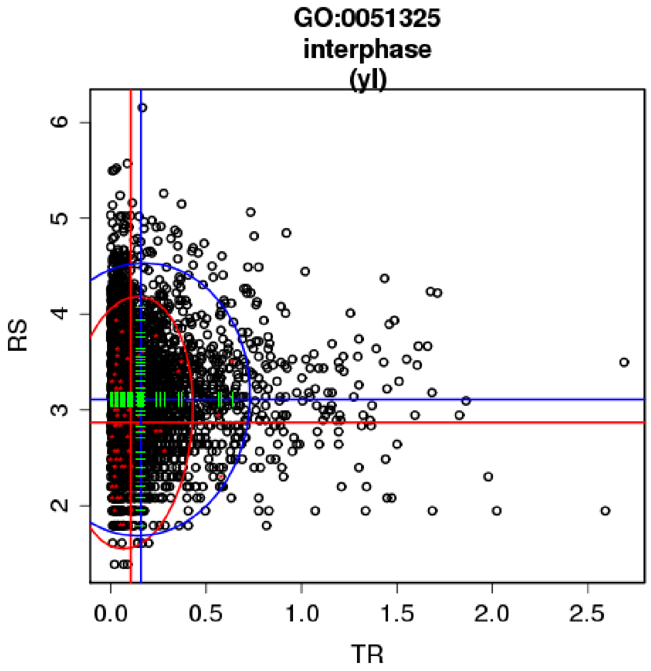
GO:0051325
interphase
(yl)
RS
TR
0.0 0.5 1.0 1.5 2.0 2.5
2 3 4 5 6

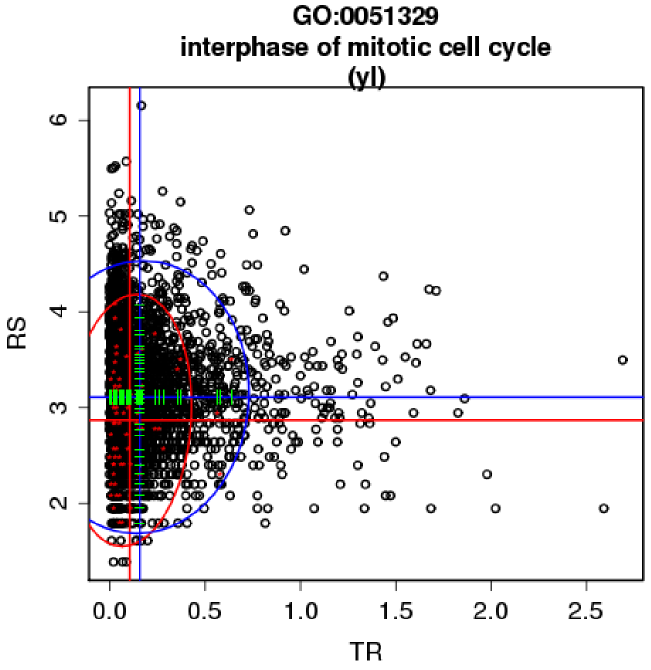
GO:0051329
interphase of mitotic cell cycle
(yl)
RS
TR
0.0 0.5 1.0 1.5 2.0 2.5
2 3 4 5 6

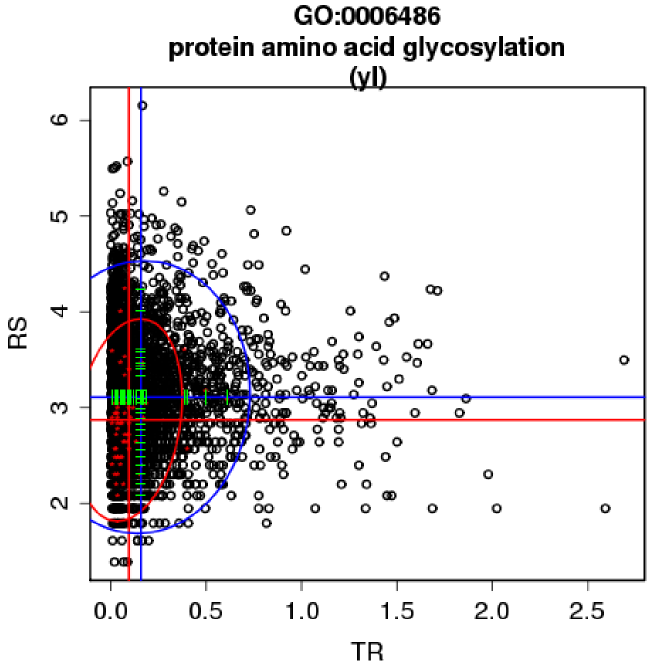
GO:0006486
protein amino acid glycosylation
(yl)
RS
TR
0.0 0.5 1.0 1.5 2.0 2.5
2 3 4 5 6

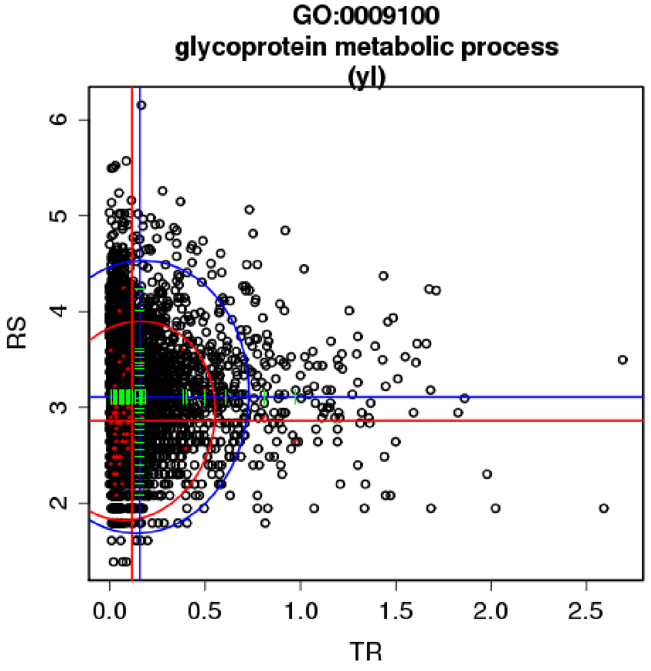
GO:0009100
glycoprotein metabolic process
(yl)
RS
TR
0.0 0.5 1.0 1.5 2.0 2.5
2 3 4 5 6

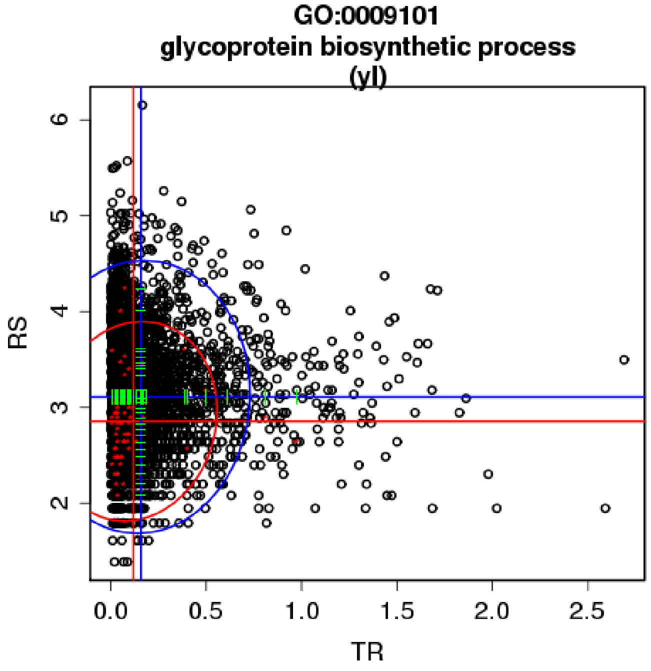
GO:0009101
glycoprotein biosynthetic process
(yl)
RS
TR
0.0 0.5 1.0 1.5 2.0 2.5
2 3 4 5 6

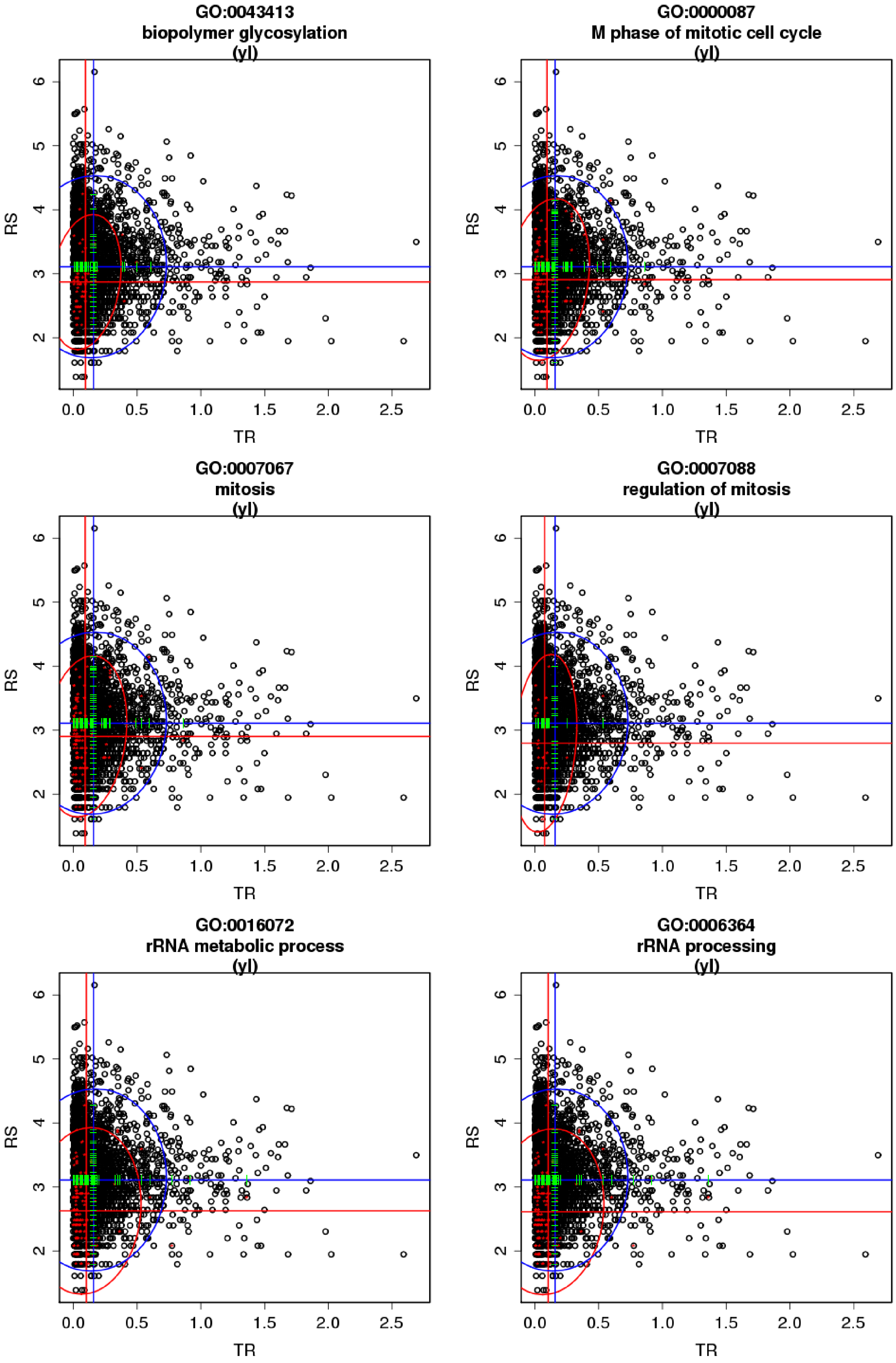
GO:0043413
biopolymer glycosylation
(yl)
GO:0000087
M phase of mitotic cell cycle
(yl)
GO:0007067
mitosis
(yl)
GO:0007088
regulation of mitosis
(yl)
GO:0016072
rRNA metabolic process
(yl)
GO:0006364
rRNA processing
(yl)
RS
TR
2
3
4
5
6
0.0
0.5
1.0
1.5
2.0
2.5

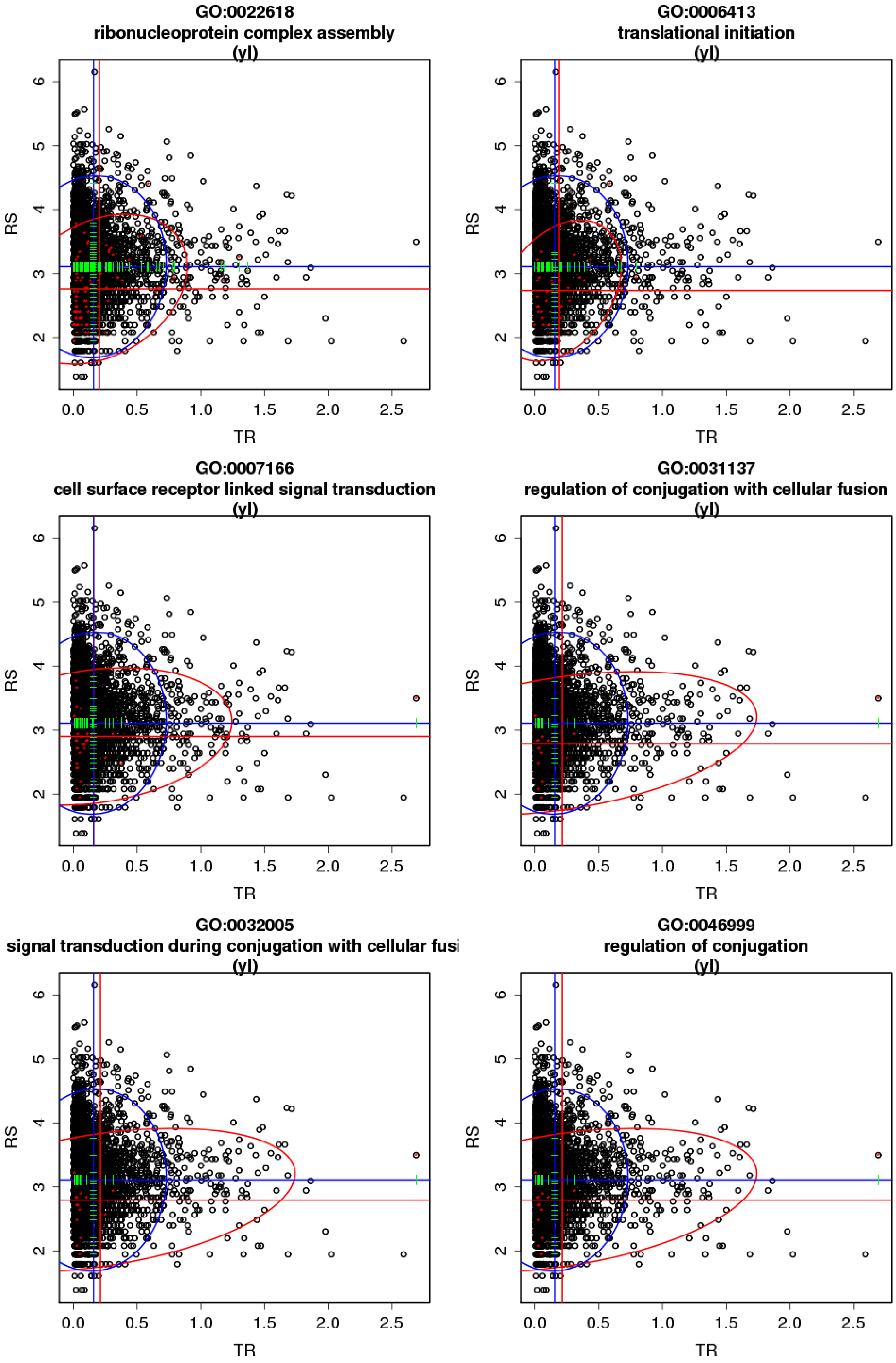
GO:0022618
ribonucleoprotein complex assembly
(yl)
GO:0006413
translational initiation
(yl)
GO:0007166
cell surface receptor linked signal transduction
(yl)
GO:0031137
regulation of conjugation with cellular fusion
(yl)
GO:0032005
signal transduction during conjugation with cellular fusi
(yl)
GO:0046999
regulation of conjugation
(yl)
RS
TR
0.0
0.5
1.0
1.5
2.0
2.5
2
3
4
5
6

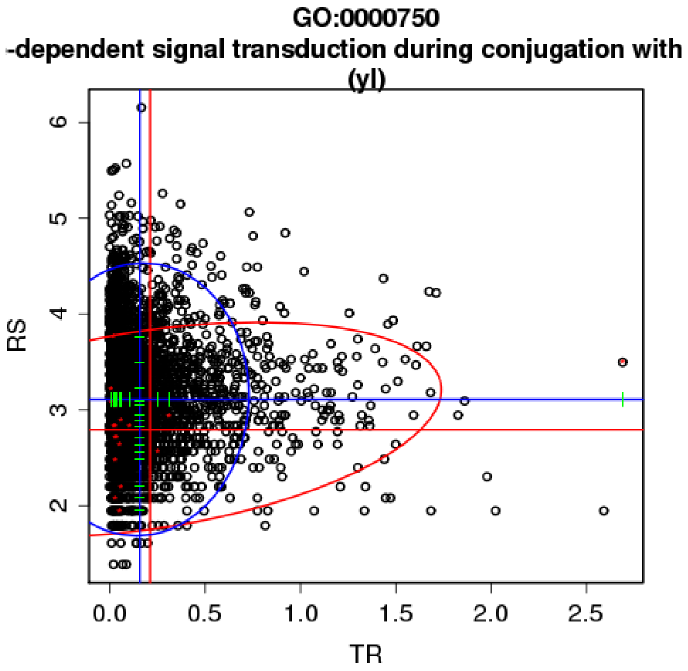
GO:0000750
-dependent signal transduction during conjugation with
(yl)
RS
TR

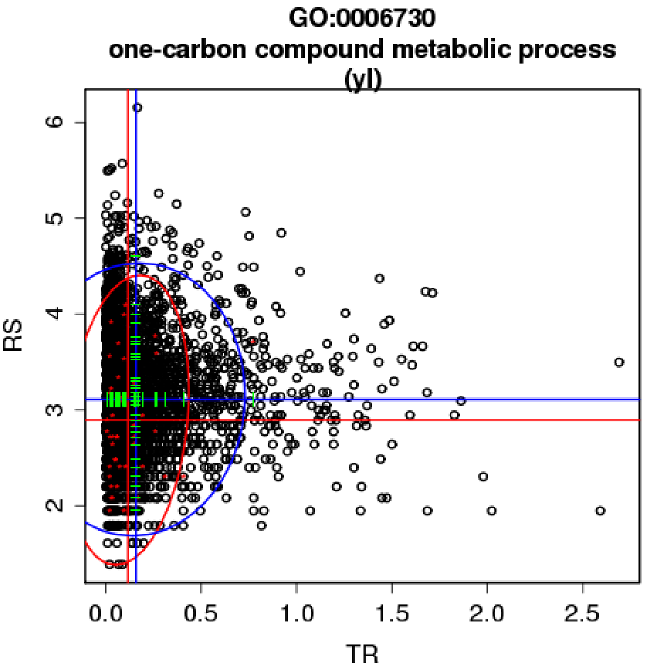
GO:0006730
one-carbon compound metabolic process
(yl)
RS
TR

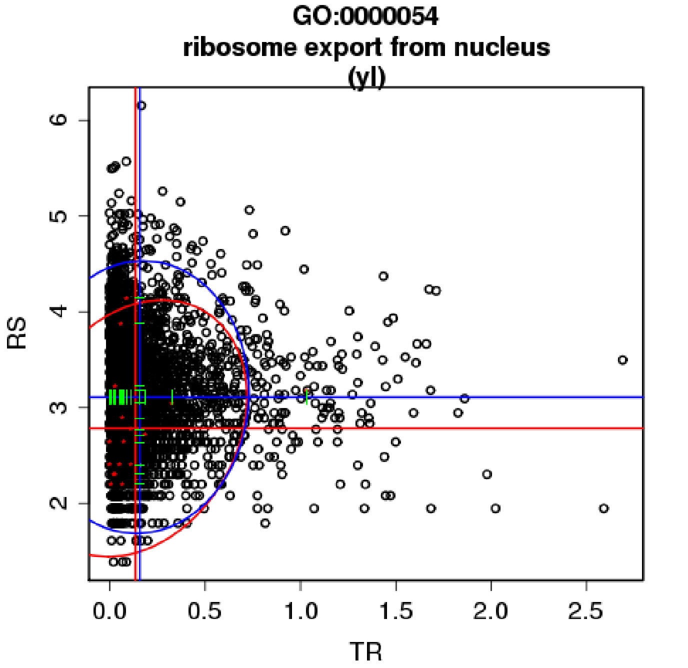
GO:0000054
ribosome export from nucleus
(yl)
RS
TR

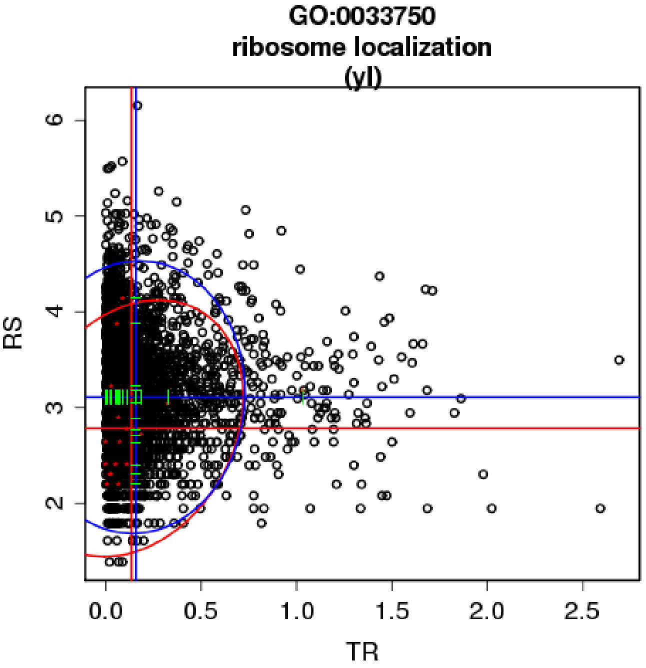
GO:0033750
ribosome localization
(yl)
RS
TR

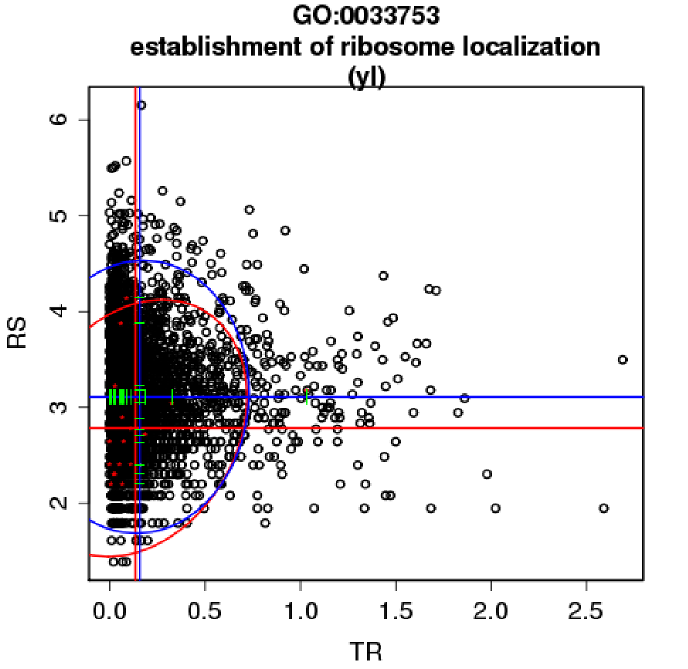
GO:0033753
establishment of ribosome localization
(yl)
RS
TR

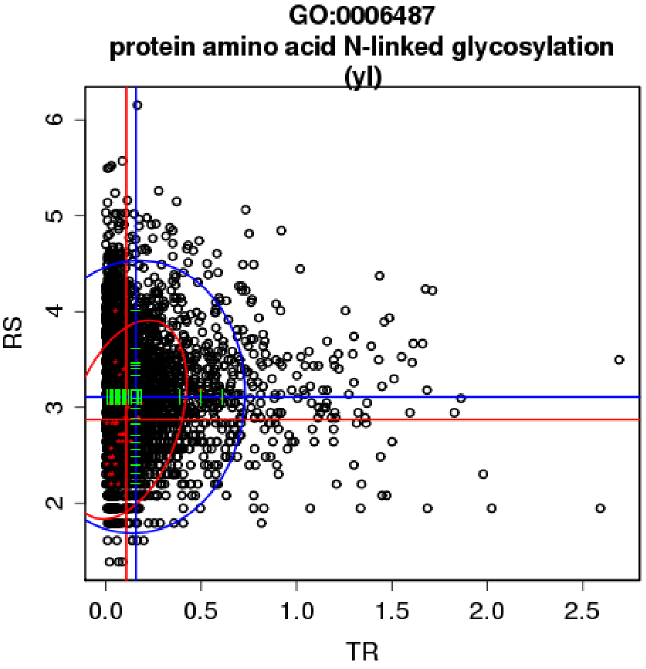
GO:0006487
protein amino acid N-linked glycosylation
(yl)
RS
TR

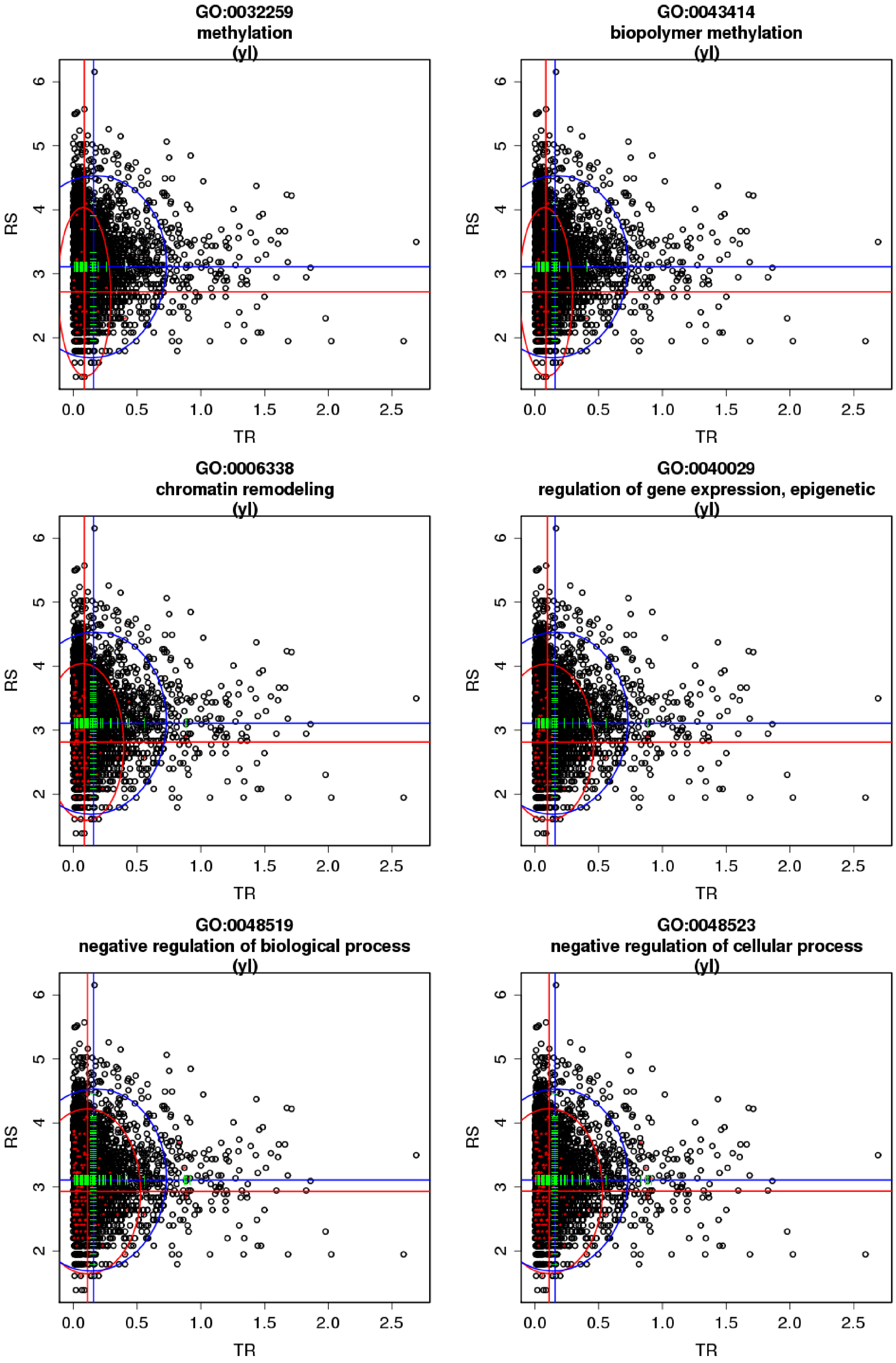
GO:0032259
methylation
(yl)
GO:0043414
biopolymer methylation
(yl)
GO:0006338
chromatin remodeling
(yl)
GO:0040029
regulation of gene expression, epigenetic
(yl)
GO:0048519
negative regulation of biological process
(yl)
GO:0048523
negative regulation of cellular process
(yl)
RS
TR
0.0
0.5
1.0
1.5
2.0
2.5
2
3
4
5
6

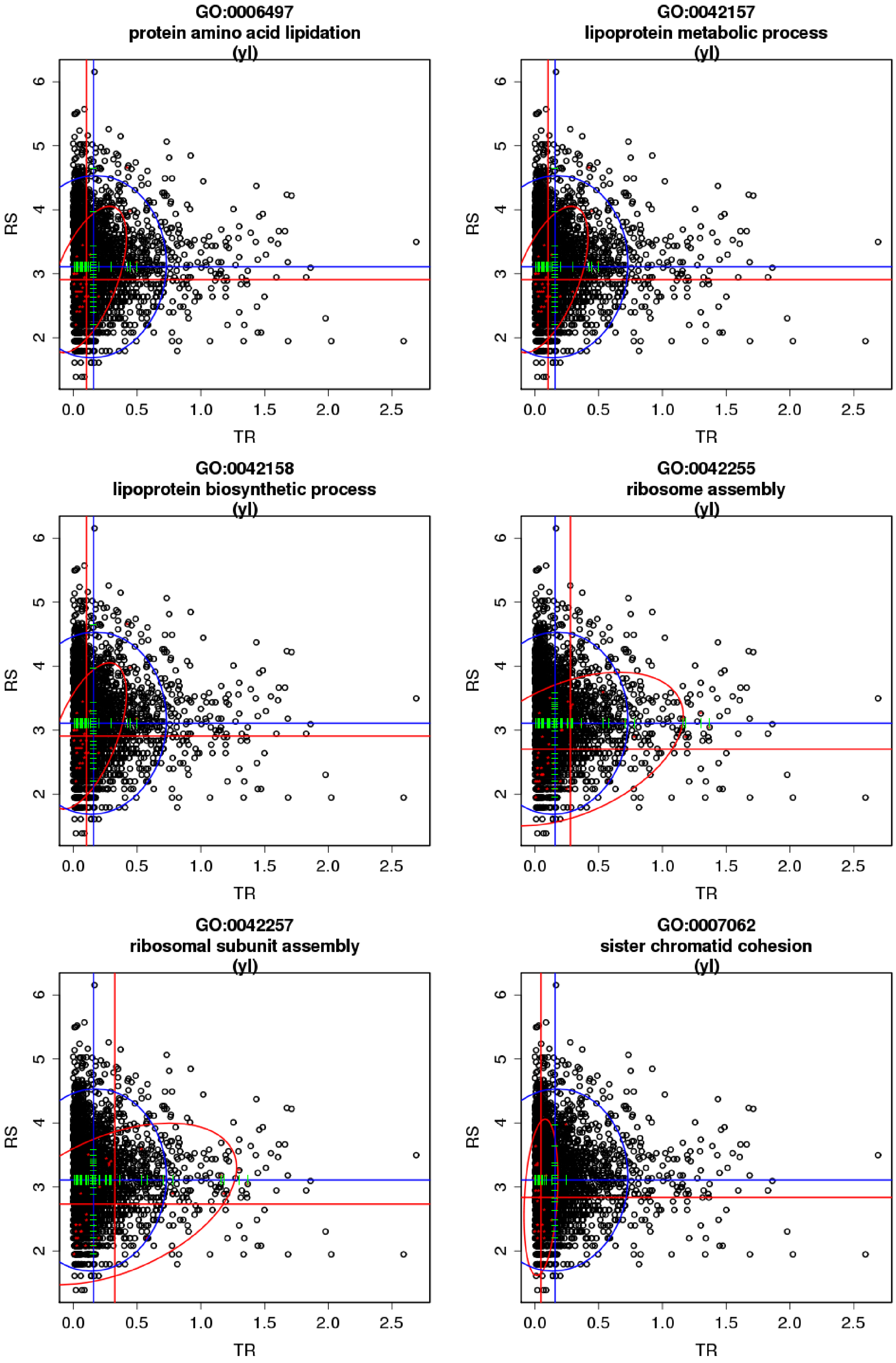
GO:0006497
protein amino acid lipidation
(yl)
GO:0042157
lipoprotein metabolic process
(yl)
GO:0042158
lipoprotein biosynthetic process
(yl)
GO:0042255
ribosome assembly
(yl)
GO:0042257
ribosomal subunit assembly
(yl)
GO:0007062
sister chromatid cohesion
(yl)
RS
TR
2
3
4
5
6
0.0
0.5
1.0
1.5
2.0
2.5

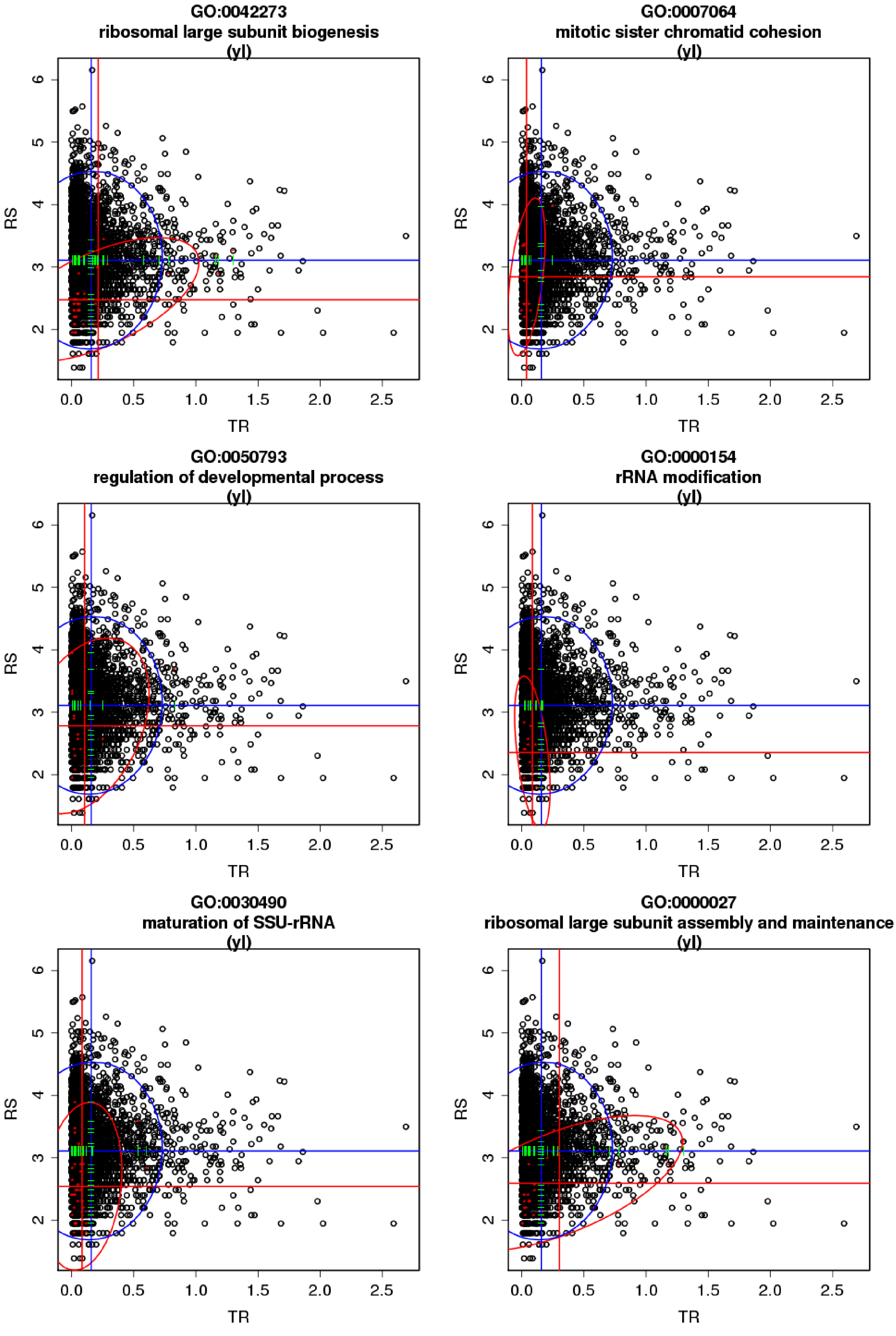
GO:0042273
ribosomal large subunit biogenesis
(yl)
RS
TR
GO:0007064
mitotic sister chromatid cohesion
(yl)
RS
TR
GO:0050793
regulation of developmental process
(yl)
RS
TR
GO:0000154
rRNA modification
(yl)
RS
TR
GO:0030490
maturation of SSU-rRNA
(yl)
RS
TR
GO:0000027
ribosomal large subunit assembly and maintenance
(yl)
RS
TR

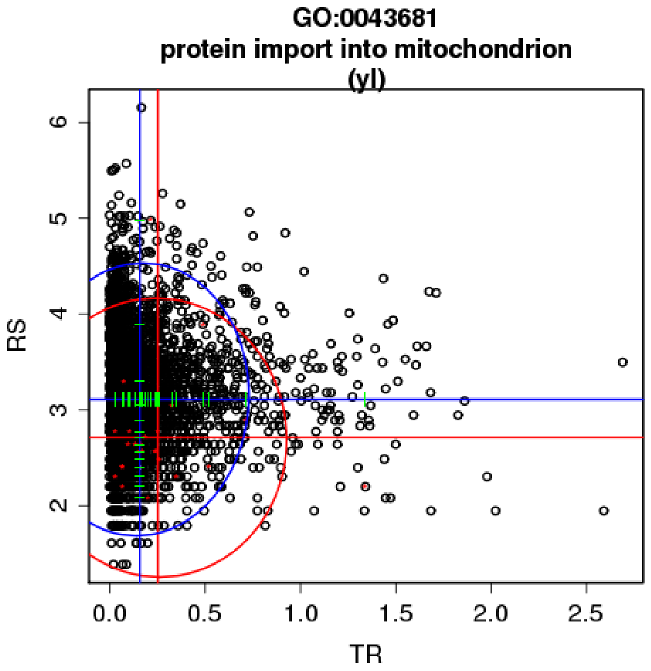
GO:0043681
protein import into mitochondrion
(yl)
RS
TR
0.0
0.5
1.0
1.5
2.0
2.5
2
3
4
5
6

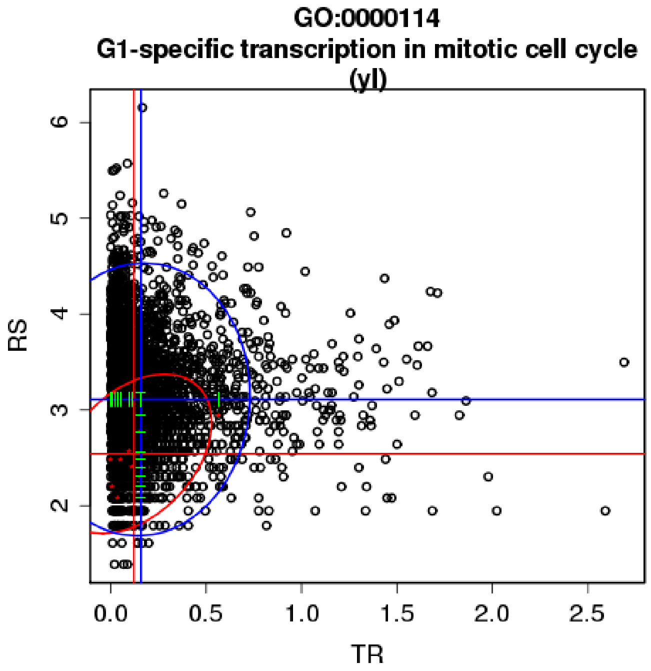
GO:0000114
G1-specific transcription in mitotic cell cycle
(yl)
RS
TR
0.0
0.5
1.0
1.5
2.0
2.5
2
3
4
5
6

# Combined functional profiling of Transcription Rate (TR) and RNA Stability (RS) using KEGG pathways

**13** significant modules

**LOR** indicates Log Odds Ratio of the coefficient or the interaction

**p** indicates FDR adjusted p-value

| | LOR TR | LOR RS | LOR Inter | p TR | p RS | p Inter | pattern | name |
|---|---|---|---|---|---|---|---|---|
| sce00010 | -0.48 | 1.70 | 0.44 | 0.97 | 0.00 | 1.00 | yh | Glycolysis / Gluconeogenesis |
| sce00500 | 3.80 | 1.77 | -1.31 | 0.97 | 0.00 | 1.00 | yh | Starch and sucrose metabolism |
| sce00052 | -2.36 | 1.49 | -0.06 | 0.97 | 0.00 | 1.00 | yh | Galactose metabolism |
| sce00630 | 6.98 | 1.14 | -2.59 | 0.75 | 0.03 | 1.00 | yh | Glyoxylate and dicarboxylate metabolism |
| sce00030 | 0.27 | 0.96 | 0.08 | 0.97 | 0.04 | 1.00 | yh | Pentose phosphate pathway |
| sce00020 | 4.71 | 1.28 | -1.44 | 0.75 | 0.00 | 1.00 | yh | Citrate cycle (TCA cycle) |
| sce04130 | -0.35 | -1.72 | 0.29 | 0.97 | 0.01 | 1.00 | yl | SNARE interactions in vesicular transport |
| sce04111 | -6.06 | -0.78 | 1.09 | 0.93 | 0.01 | 1.00 | yl | Cell cycle |
| sce00230 | 1.32 | -0.97 | -0.24 | 0.97 | 0.00 | 1.00 | yl | Purine metabolism |
| sce04011 | -5.61 | -1.38 | 1.91 | 0.91 | 0.00 | 1.00 | yl | MAPK signaling pathway |
| sce00240 | 1.45 | -0.97 | -0.24 | 0.97 | 0.01 | 1.00 | yl | Pyrimidine metabolism |
| sce03050 | 0.68 | -1.27 | 0.17 | 0.97 | 0.01 | 1.00 | yl | Proteasome |
| sce03020 | 1.20 | -2.20 | -0.11 | 0.97 | 0.00 | 1.00 | yl | RNA polymerase |

Table 1: LOR: log odds ratio; p: adjusted p-value FDR

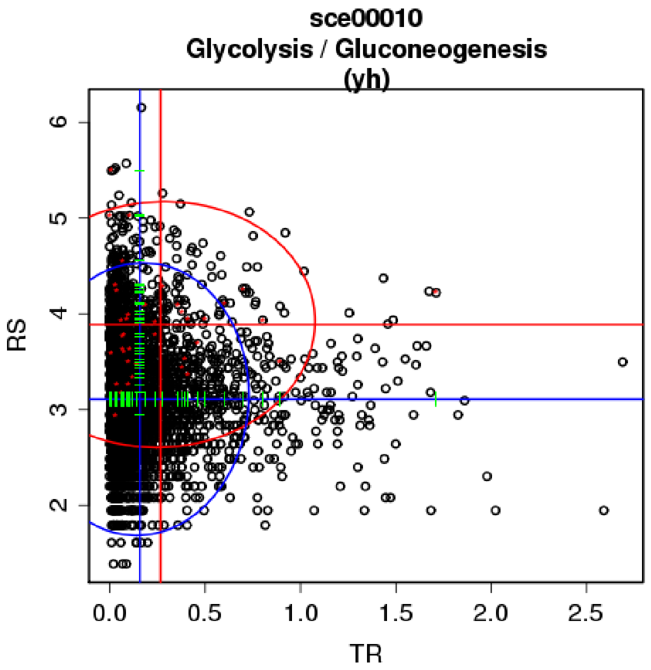


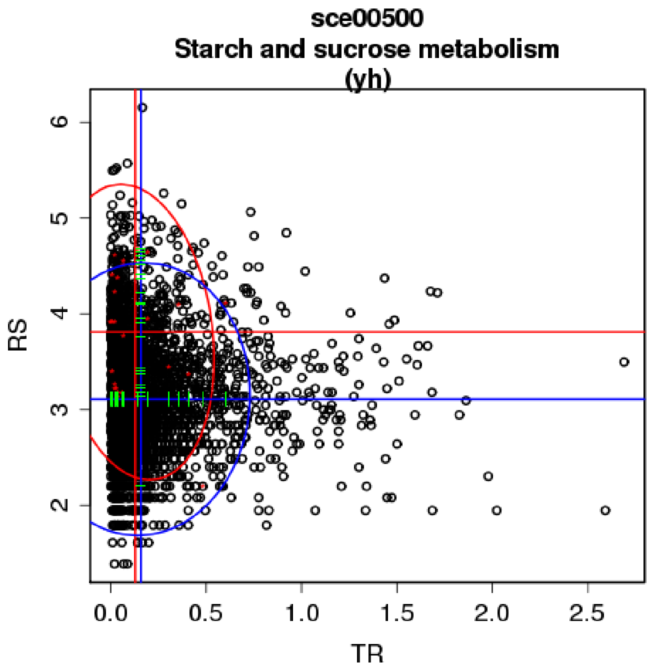

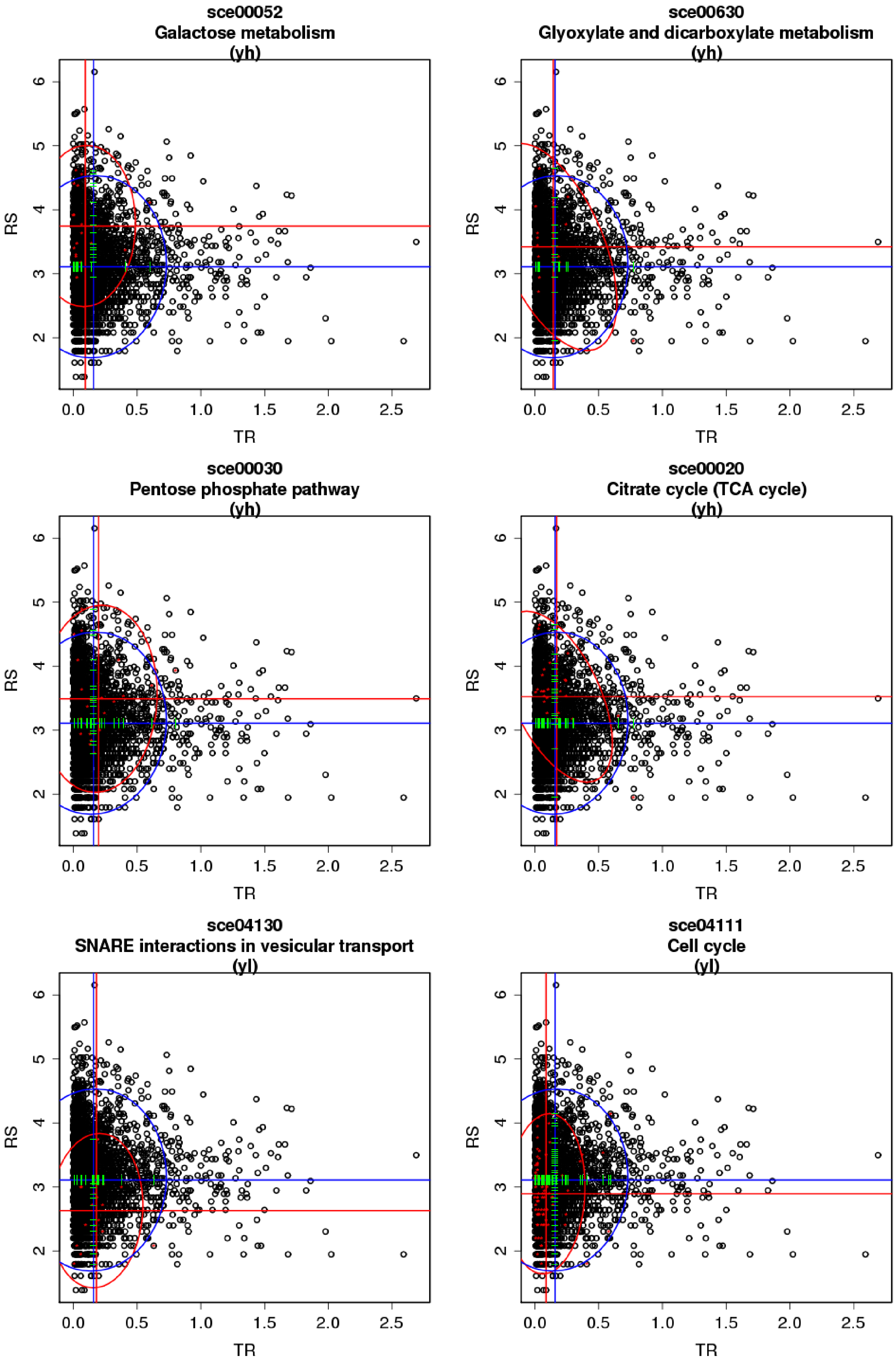
sce00052
Galactose metabolism
(yh)
sce00630
Glyoxylate and dicarboxylate metabolism
(yh)
sce00030
Pentose phosphate pathway
(yh)
sce00020
Citrate cycle (TCA cycle)
(yh)
sce04130
SNARE interactions in vesicular transport
(yl)
sce04111
Cell cycle
(yl)
RS
TR
0.0
0.5
1.0
1.5
2.0
2.5
2
3
4
5
6

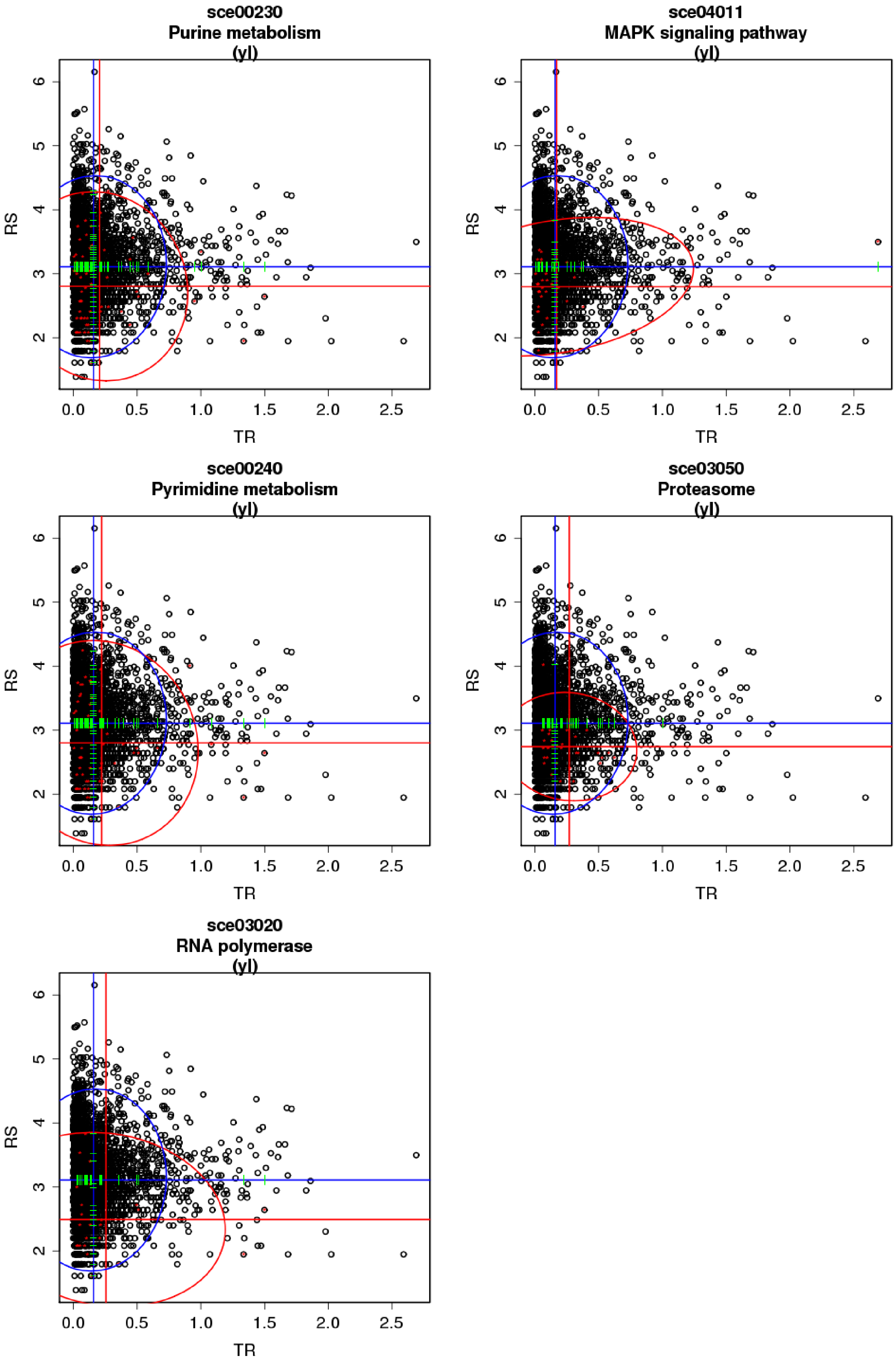
sce00230
Purine metabolism
(yl)
sce04011
MAPK signaling pathway
(yl)
sce00240
Pyrimidine metabolism
(yl)
sce03050
Proteasome
(yl)
sce03020
RNA polymerase
(yl)
RS
TR
0.0
0.5
1.0
1.5
2.0
2.5
2
3
4
5
6
