# Supplementary material for: Multidimensional Gene Set Analysis of Genomic Data
Source: PLoS One. 2010 Apr 27;5(4):e10348. doi: 10.1371/journal.pone.0010348 (PMC2860497; doi:10.1371/journal.pone.0010348)
Supplement: File S2 — A) GO Biological Process terms and B) KEGG pathways, significant for alternative splicing and differential gene expression and their interaction, along with the corresponding graphical representations. In the plots blue lines intersect in the mean of the distribution of all the values and red lines intersect in the mean of the distribution of values of the genes corresponding to the term analysed. Blue ellipse delimits the confidence interval for all the values and red ellipse delimits the confidence interval for the term analysed. The red ellipse marks the trend of the relationship between both variables. (9.22 MB PDF) [file pone.0010348.s008.pdf]

Combined functional profiling of alternative splicing and differential gene expression using GO Biological Process

154 significant modules

LOR indicates Log Odds Ratio of the coefficient or the interaction

p indicates FDR adjusted p-value

	LOR si	LOR dif	LOR Inter	p si	p dif	p Inter	pattern	name
GO:0006767	0.15	-0.15	0.14	1.00	0.61	0.04	b13	water-soluble vitamin metabolic process
GO:0045216	0.29	-0.04	0.17	1.00	0.95	0.02	b13	cell-cell junction assembly and maintenance
GO:0007043	0.38	-0.03	0.18	1.00	0.97	0.02	b13	cell-cell junction assembly
GO:0048706	0.20	0.08	0.17	1.00	0.89	0.03	b13	embryonic skeletal development
GO:0007034	0.32	-0.18	0.17	1.00	0.65	0.02	b13	vacuolar transport
GO:0007041	0.32	-0.10	0.18	1.00	0.86	0.01	b13	lysosomal transport
GO:0048704	0.23	0.12	0.19	1.00	0.84	0.02	b13	embryonic skeletal morphogenesis
GO:0048705	0.17	0.10	0.17	1.00	0.85	0.02	b13	skeletal morphogenesis
GO:0016197	0.08	0.10	0.15	1.00	0.79	0.02	b13	endosome transport
GO:0030155	0.01	-0.16	-0.15	1.00	0.43	0.01	b24	regulation of cell adhesion
GO:0045785	-0.04	0.06	-0.18	1.00	0.94	0.02	b24	positive regulation of cell adhesion
GO:0030032	-0.16	-0.17	-0.18	1.00	0.72	0.03	b24	lamellipodium biogenesis
GO:0050792	0.65	0.27	-0.05	0.01	0.61	1.00	xh	regulation of viral reproduction
GO:0001709	0.16	0.33	-0.02	1.00	0.05	1.00	yh	cell fate determination
GO:0019226	0.05	0.15	-0.03	1.00	0.03	1.00	yh	transmission of nerve impulse
GO:0007416	0.32	0.34	0.01	0.89	0.02	1.00	yh	synaptogenesis
GO:0065004	-0.06	0.19	0.04	1.00	0.04	1.00	yh	protein-DNA complex assembly
GO:0006325	-0.04	0.19	0.06	1.00	0.00	1.00	yh	establishment and/or maintenance of chromatin architecture
GO:0006333	-0.08	0.21	0.06	1.00	0.01	1.00	yh	chromatin assembly or disassembly
GO:0008286	-0.05	0.36	0.02	1.00	0.00	1.00	yh	insulin receptor signaling pathway
GO:0051648	-0.16	0.41	0.05	1.00	0.02	1.00	yh	vesicle localization
GO:0016339	0.09	0.37	0.06	1.00	0.03	1.00	yh	calcium-dependent cell-cell adhesion
GO:0030239	0.21	0.39	-0.05	1.00	0.04	1.00	yh	myofibril assembly
GO:0051146	0.18	0.36	-0.02	1.00	0.02	1.00	yh	striated muscle cell differentiation
GO:0055002	0.21	0.39	-0.05	1.00	0.04	1.00	yh	striated muscle cell development
GO:0048009	-0.32	0.52	0.07	1.00	0.00	1.00	yh	insulin-like growth factor receptor signaling pathway
GO:0000087	0.05	-0.34	0.00	1.00	0.00	1.00	yl	M phase of mitotic cell cycle
GO:0000278	0.03	-0.28	0.02	1.00	0.00	1.00	yl	mitotic cell cycle
GO:0000279	-0.02	-0.30	-0.01	1.00	0.00	1.00	yl	M phase
GO:0022402	-0.00	-0.23	-0.01	1.00	0.00	1.00	yl	cell cycle process
GO:0022403	-0.02	-0.23	-0.00	1.00	0.00	1.00	yl	cell cycle phase
GO:0006396	-0.01	-0.24	0.03	1.00	0.00	1.00	yl	RNA processing
GO:0051301	0.09	-0.21	0.06	1.00	0.02	1.00	yl	cell division
GO:0007067	0.05	-0.33	-0.01	1.00	0.00	1.00	yl	mitosis
GO:0007599	0.12	-0.28	0.01	1.00	0.00	1.00	yl	hemostasis
GO:0042060	0.09	-0.29	0.03	1.00	0.00	1.00	yl	wound healing
GO:0050817	0.13	-0.26	0.00	1.00	0.01	1.00	yl	coagulation
GO:0050878	0.09	-0.26	0.00	1.00	0.01	1.00	yl	regulation of body fluid levels
GO:0007596	0.11	-0.27	0.01	1.00	0.01	1.00	yl	blood coagulation
GO:0046483	0.10	-0.30	0.05	1.00	0.02	1.00	yl	heterocycle metabolic process
GO:0006457	0.07	-0.27	0.04	1.00	0.00	1.00	yl	protein folding
GO:0006118	-0.04	-0.15	-0.05	1.00	0.01	1.00	yl	
GO:0006461	0.05	-0.16	0.03	1.00	0.04	1.00	yl	protein complex assembly
GO:0007088	-0.01	-0.37	-0.08	1.00	0.04	1.00	yl	regulation of mitosis
GO:0051726	0.06	-0.22	0.02	1.00	0.00	1.00	yl	regulation of cell cycle
GO:0007160	-0.07	-0.28	-0.02	1.00	0.02	1.00	yl	cell-matrix adhesion
GO:0031589	-0.07	-0.27	-0.02	1.00	0.02	1.00	yl	cell-substrate adhesion
GO:0019221	0.07	-0.35	-0.04	1.00	0.04	1.00	yl	cytokine and chemokine mediated signaling pathway
GO:0008610	0.06	-0.26	-0.01	1.00	0.00	1.00	yl	lipid biosynthetic process
GO:0006694	0.15	-0.35	0.03	1.00	0.00	1.00	yl	steroid biosynthetic process
GO:0007398	-0.01	-0.53	-0.03	1.00	0.00	1.00	yl	ectoderm development
GO:0008544	0.04	-0.51	-0.03	1.00	0.00	1.00	yl	epidermis development
GO:0009888	-0.07	-0.31	-0.05	1.00	0.00	1.00	yl	tissue development
GO:0009913	0.09	-0.34	-0.03	1.00	0.04	1.00	yl	epidermal cell differentiation
GO:0031424	0.05	-0.38	-0.03	1.00	0.03	1.00	yl	keratinization
GO:0006399	-0.00	-0.30	0.01	1.00	0.00	1.00	yl	tRNA metabolic process
GO:0006418	-0.05	-0.31	-0.04	1.00	0.03	1.00	yl	tRNA aminoacylation for protein translation
GO:0006519	0.02	-0.17	0.04	1.00	0.02	1.00	yl	amino acid and derivative metabolic process
GO:0006520	-0.00	-0.21	0.04	1.00	0.01	1.00	yl	amino acid metabolic process
GO:0043038	-0.05	-0.31	-0.04	1.00	0.03	1.00	yl	amino acid activation
GO:0043039	-0.05	-0.31	-0.04	1.00	0.03	1.00	yl	tRNA aminoacylation
GO:0065002	0.09	-0.33	0.03	1.00	0.02	1.00	yl	intracellular protein transport across a membrane
GO:0006096	-0.21	-0.34	0.00	1.00	0.02	1.00	yl	glycolysis
GO:0006954	0.12	-0.17	-0.04	0.89	0.03	1.00	yl	inflammatory response
GO:0043623	0.02	-0.34	0.01	1.00	0.01	1.00	yl	cellular protein complex assembly
GO:0043549	0.03	-0.19	-0.07	1.00	0.03	1.00	yl	regulation of kinase activity
GO:0051338	0.03	-0.18	-0.07	1.00	0.03	1.00	yl	regulation of transferase activity
GO:0045859	0.06	-0.23	-0.05	1.00	0.00	1.00	yl	regulation of protein kinase activity
GO:0009259	-0.18	-0.29	0.00	1.00	0.02	1.00	yl	ribonucleotide metabolic process
GO:0055086	-0.08	-0.18	0.03	1.00	0.05	1.00	yl	nucleobase, nucleoside and nucleotide metabolic process
GO:0006732	0.01	-0.31	-0.01	1.00	0.00	1.00	yl	coenzyme metabolic process

GO:0006752	0.10	-0.44	0.04	1.00	0.04	1.00	yl	group transfer coenzyme metabolic process
GO:0009108	0.11	-0.32	0.03	1.00	0.04	1.00	yl	coenzyme biosynthetic process
GO:0051186	0.03	-0.24	0.01	1.00	0.01	1.00	yl	cofactor metabolic process
GO:0051188	0.08	-0.24	0.04	1.00	0.05	1.00	yl	cofactor biosynthetic process
GO:0006260	-0.03	-0.23	0.03	1.00	0.01	1.00	yl	DNA replication
GO:0006917	0.02	-0.23	-0.04	1.00	0.01	1.00	yl	induction of apoptosis
GO:0008629	-0.06	-0.47	-0.04	1.00	0.00	1.00	yl	induction of apoptosis by intracellular signals
GO:0012502	0.02	-0.23	-0.04	1.00	0.01	1.00	yl	induction of programmed cell death
GO:0042770	-0.08	-0.34	-0.05	1.00	0.03	1.00	yl	DNA damage response, signal transduction
GO:0043065	-0.01	-0.20	-0.03	1.00	0.02	1.00	yl	positive regulation of apoptosis
GO:0043068	-0.01	-0.19	-0.03	1.00	0.02	1.00	yl	positive regulation of programmed cell death
GO:0008630	-0.09	-0.47	-0.06	1.00	0.01	1.00	yl	DNA damage response, signal transduction resulting in induction of apoptosis
GO:0016072	-0.03	-0.48	0.00	1.00	0.00	1.00	yl	rRNA metabolic process
GO:0022613	-0.05	-0.34	-0.00	1.00	0.00	1.00	yl	ribonucleoprotein complex biogenesis and assembly
GO:0042254	-0.03	-0.49	0.00	1.00	0.00	1.00	yl	ribosome biogenesis
GO:0006364	-0.00	-0.49	0.01	1.00	0.00	1.00	yl	rRNA processing
GO:0001525	0.14	-0.25	-0.04	1.00	0.02	1.00	yl	angiogenesis
GO:0001568	0.15	-0.20	-0.03	0.89	0.04	1.00	yl	blood vessel development
GO:0001944	0.14	-0.22	-0.02	1.00	0.02	1.00	yl	vasculature development
GO:0048514	0.16	-0.21	-0.04	0.90	0.04	1.00	yl	blood vessel morphogenesis
GO:0048646	0.14	-0.21	-0.03	1.00	0.04	1.00	yl	anatomical structure formation
GO:0007005	-0.04	-0.40	-0.03	1.00	0.00	1.00	yl	mitochondrion organization and biogenesis
GO:0033002	0.23	-0.47	0.05	1.00	0.02	1.00	yl	muscle cell proliferation
GO:0048659	0.31	-0.56	0.07	1.00	0.00	1.00	yl	smooth muscle cell proliferation
GO:0048660	0.27	-0.58	0.06	1.00	0.00	1.00	yl	regulation of smooth muscle cell proliferation
GO:0006626	0.02	-0.57	0.01	1.00	0.00	1.00	yl	protein targeting to mitochondrion
GO:0001523	0.12	-0.54	0.01	1.00	0.02	1.00	yl	retinoid metabolic process
GO:0006720	-0.04	-0.56	-0.02	1.00	0.00	1.00	yl	isoprenoid metabolic process
GO:0006775	-0.12	-0.46	-0.05	1.00	0.02	1.00	yl	fat-soluble vitamin metabolic process
GO:0006776	-0.09	-0.55	-0.03	1.00	0.00	1.00	yl	vitamin A metabolic process
GO:0016101	0.12	-0.54	0.01	1.00	0.02	1.00	yl	diterpenoid metabolic process
GO:0006119	-0.01	-0.27	0.01	1.00	0.02	1.00	yl	oxidative phosphorylation
GO:0042773	0.14	-0.36	0.04	1.00	0.01	1.00	yl	ATP synthesis coupled electron transport
GO:0042775	0.05	-0.37	0.03	1.00	0.01	1.00	yl	organelle ATP synthesis coupled electron transport
GO:0006120	0.06	-0.38	0.04	1.00	0.01	1.00	yl	mitochondrial electron transport, NADH to ubiquinone
GO:0000226	-0.02	-0.31	0.01	1.00	0.00	1.00	yl	microtubule cytoskeleton organization and biogenesis
GO:0006084	-0.08	-0.45	-0.04	1.00	0.00	1.00	yl	acetyl-CoA metabolic process
GO:0009060	0.03	-0.44	-0.01	1.00	0.00	1.00	yl	aerobic respiration
GO:0009109	0.04	-0.52	0.00	1.00	0.00	1.00	yl	coenzyme catabolic process
GO:0045333	-0.00	-0.40	-0.04	1.00	0.01	1.00	yl	cellular respiration
GO:0046356	0.06	-0.49	-0.00	1.00	0.00	1.00	yl	acetyl-CoA catabolic process
GO:0051187	0.06	-0.46	0.01	1.00	0.00	1.00	yl	cofactor catabolic process
GO:0006099	0.06	-0.49	-0.00	1.00	0.00	1.00	yl	tricarboxylic acid cycle
GO:0000070	0.08	-0.42	0.05	1.00	0.03	1.00	yl	mitotic sister chromatid segregation
GO:0000819	0.09	-0.40	0.05	1.00	0.04	1.00	yl	sister chromatid segregation
GO:0007059	0.02	-0.32	0.06	1.00	0.04	1.00	yl	chromosome segregation
GO:0008284	-0.04	-0.19	-0.04	1.00	0.02	1.00	yl	positive regulation of cell proliferation
GO:0030031	-0.08	-0.32	-0.08	1.00	0.02	1.00	yl	cell projection biogenesis
GO:0033673	-0.02	-0.37	-0.03	1.00	0.00	1.00	yl	negative regulation of kinase activity
GO:0043086	-0.10	-0.29	-0.05	1.00	0.02	1.00	yl	negative regulation of catalytic activity
GO:0051348	-0.02	-0.36	-0.03	1.00	0.01	1.00	yl	negative regulation of transferase activity
GO:0006469	-0.02	-0.37	-0.03	1.00	0.00	1.00	yl	negative regulation of protein kinase activity
GO:0043405	0.06	-0.26	-0.05	1.00	0.05	1.00	yl	regulation of MAP kinase activity
GO:0009123	-0.23	-0.43	0.00	1.00	0.04	1.00	yl	nucleoside monophosphate metabolic process
GO:0009124	-0.23	-0.43	0.00	1.00	0.04	1.00	yl	nucleoside monophosphate biosynthetic process
GO:0051052	0.01	-0.30	0.02	1.00	0.05	1.00	yl	regulation of DNA metabolic process
GO:0042330	0.16	-0.30	-0.04	0.90	0.00	1.00	yl	taxis
GO:0006935	0.16	-0.30	-0.04	0.90	0.00	1.00	yl	chemotaxis
GO:0018149	0.14	-0.55	-0.03	1.00	0.00	1.00	yl	peptide cross-linking
GO:0030216	-0.15	-0.70	-0.08	1.00	0.00	1.00	yl	keratinocyte differentiation
GO:0000079	-0.04	-0.39	-0.05	1.00	0.01	1.00	yl	regulation of cyclin-dependent protein kinase activity
GO:0006100	-0.13	-0.63	-0.02	1.00	0.00	1.00	yl	
GO:0000059	-0.02	-0.52	0.01	1.00	0.00	1.00	yl	protein import into nucleus, docking
GO:0031099	-0.24	-0.47	0.02	1.00	0.02	1.00	yl	regeneration
GO:0031109	-0.04	-0.39	-0.03	1.00	0.05	1.00	yl	microtubule polymerization or depolymerization
GO:0006695	-0.18	-0.50	-0.02	1.00	0.00	1.00	yl	cholesterol biosynthetic process
GO:0016126	-0.11	-0.50	-0.01	1.00	0.00	1.00	yl	sterol biosynthetic process
GO:0001836	-0.29	-0.51	-0.12	1.00	0.02	1.00	yl	release of cytochrome c from mitochondria
GO:0046112	-0.12	-0.54	-0.01	1.00	0.03	1.00	yl	nucleobase biosynthetic process
GO:0006690	-0.04	-0.38	-0.06	1.00	0.02	1.00	yl	icosanoid metabolic process
GO:0009166	-0.05	-0.54	-0.01	1.00	0.00	1.00	yl	nucleotide catabolic process
GO:0006633	-0.06	-0.33	-0.06	1.00	0.01	1.00	yl	fatty acid biosynthetic process
GO:0032886	-0.06	-0.41	-0.03	1.00	0.04	1.00	yl	regulation of microtubule-based process
GO:0051346	-0.30	-0.57	-0.14	1.00	0.00	1.00	yl	negative regulation of hydrolase activity
GO:0008299	-0.17	-0.60	-0.05	1.00	0.00	1.00	yl	isoprenoid biosynthetic process
GO:0001937	-0.21	-0.53	-0.07	1.00	0.03	1.00	yl	negative regulation of endothelial cell proliferation
GO:0007250	0.15	-0.51	-0.07	1.00	0.03	1.00	yl	activation of NF-kappaB-inducing kinase activity
GO:0032602	-0.06	-0.51	-0.12	1.00	0.05	1.00	yl	chemokine production
GO:0050755	-0.08	-0.57	-0.12	1.00	0.01	1.00	yl	chemokine metabolic process
GO:0042033	-0.08	-0.57	-0.12	1.00	0.01	1.00	yl	chemokine biosynthetic process
GO:0022408	-0.13	-0.54	-0.06	1.00	0.03	1.00	yl	negative regulation of cell-cell adhesion
GO:0045428	0.28	-0.53	0.04	1.00	0.02	1.00	yl	regulation of nitric oxide biosynthetic process
GO:0043407	-0.17	-0.43	-0.02	1.00	0.02	1.00	yl	negative regulation of MAP kinase activity

Table 1: LOR: log odds ratio; p: adjusted p-value FDR

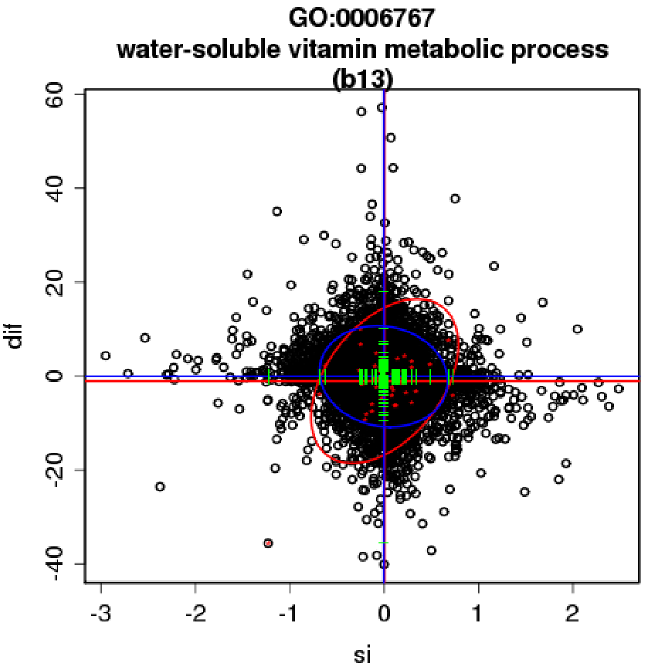
GO:0006767
water-soluble vitamin metabolic process
(b13)
dif
si

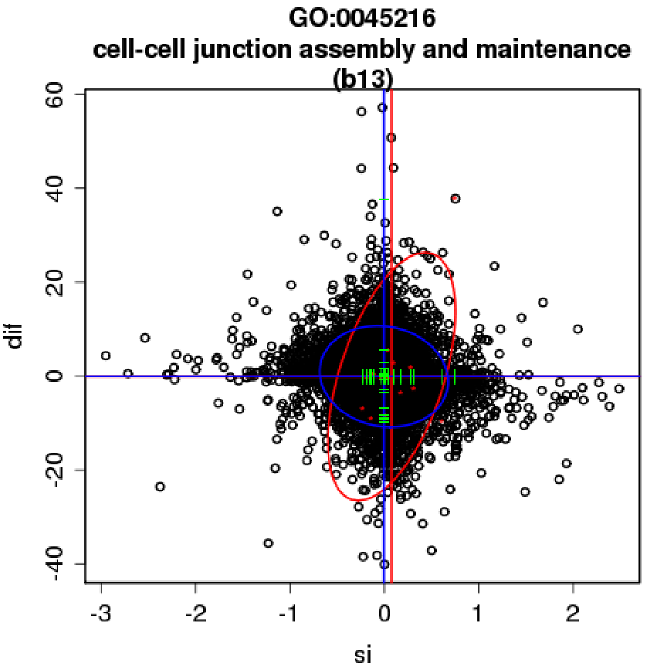
GO:0045216
cell-cell junction assembly and maintenance
(b13)
dif
si

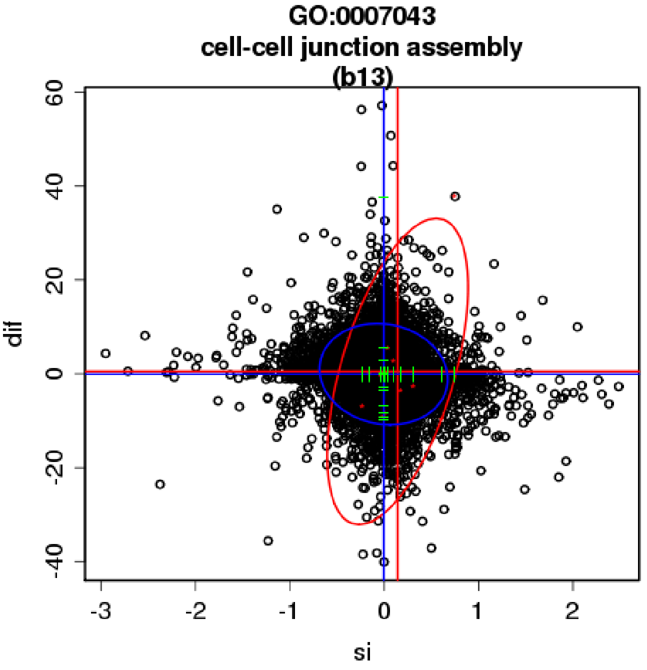
GO:0007043
cell-cell junction assembly
(b13)
dif
si

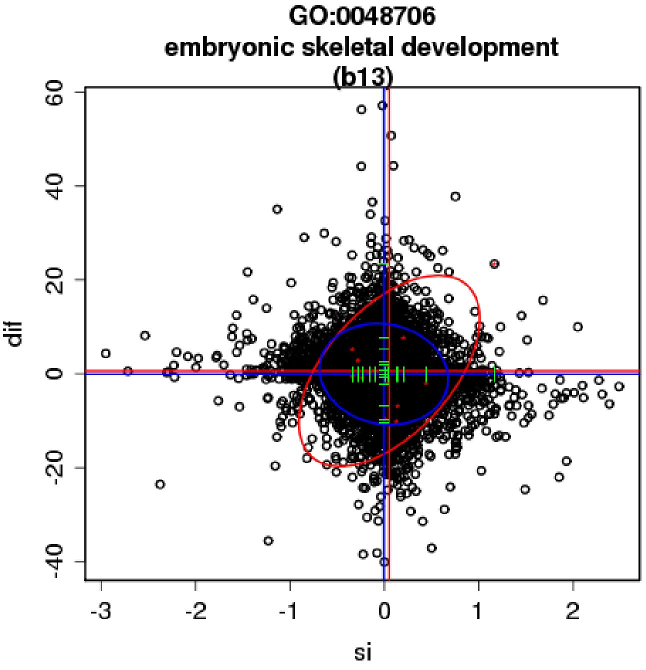
GO:0048706
embryonic skeletal development
(b13)
dif
si

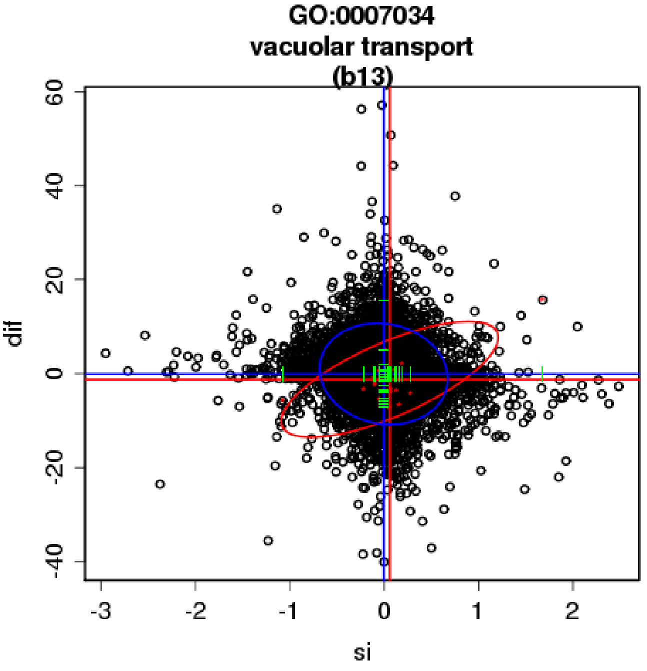
GO:0007034
vacuolar transport
(b13)
dif
si

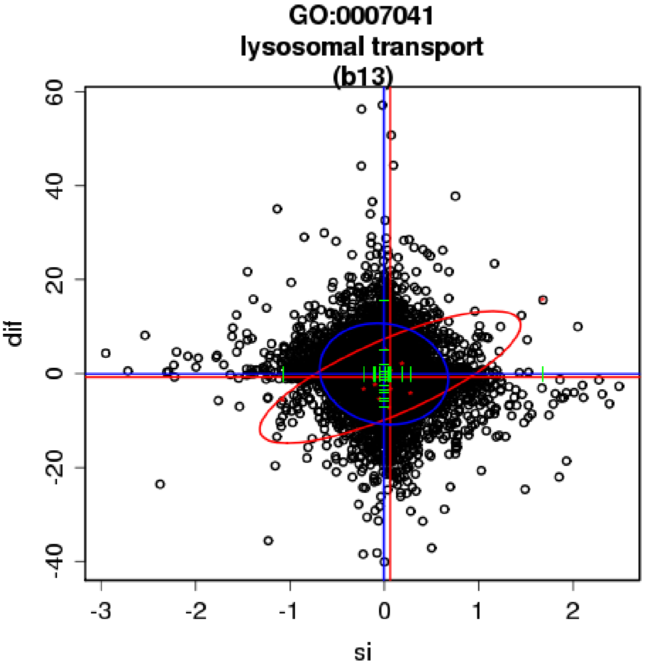
GO:0007041
lysosomal transport
(b13)
dif
si

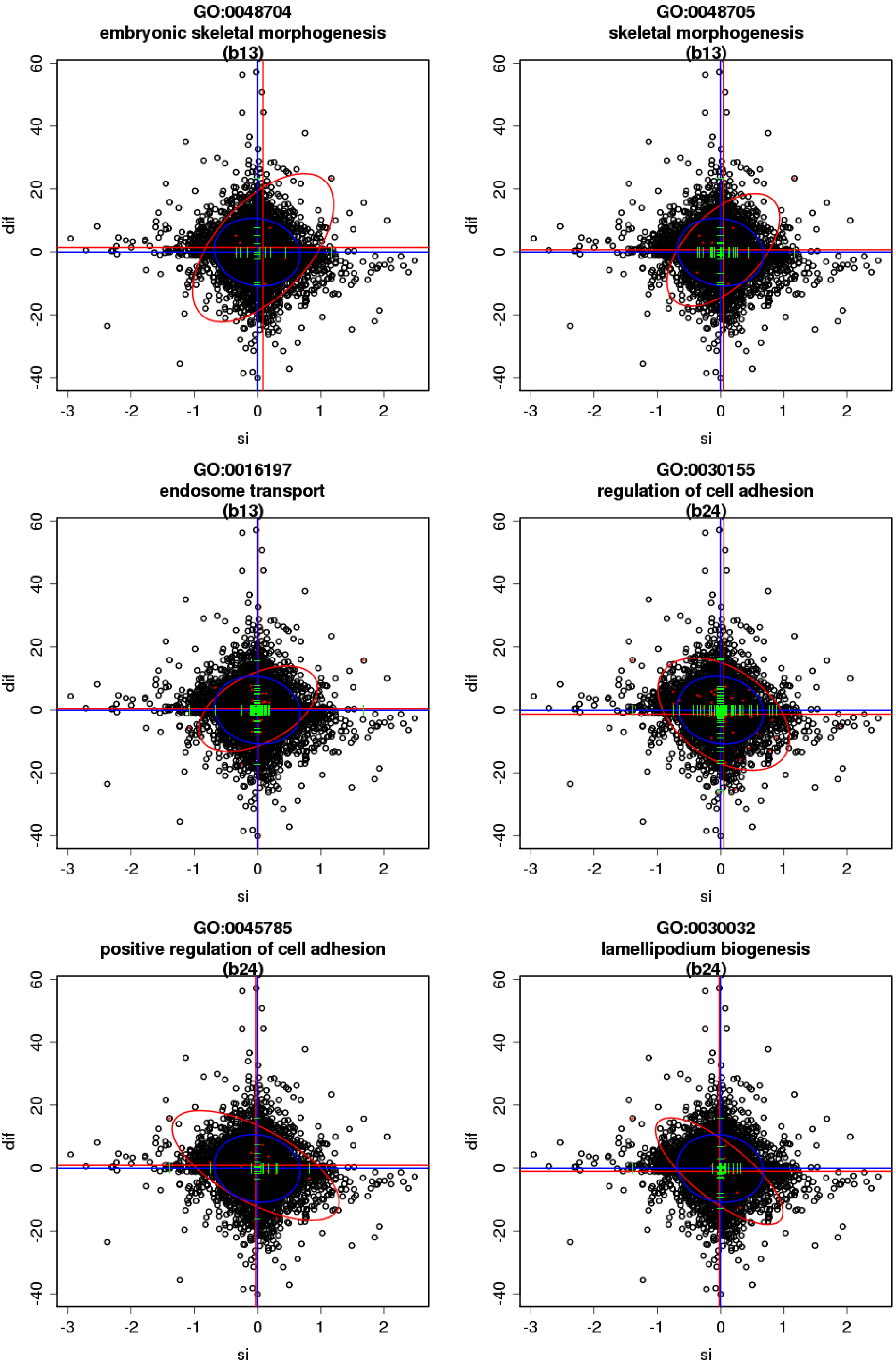
GO:0048704
embryonic skeletal morphogenesis
(b13)
GO:0048705
skeletal morphogenesis
(b13)
GO:0016197
endosome transport
(b13)
GO:0030155
regulation of cell adhesion
(b24)
GO:0045785
positive regulation of cell adhesion
(b24)
GO:0030032
lamellipodium biogenesis
(b24)
dif
si
60
40
20
0
-20
-40
-3
-2
-1
0
1
2

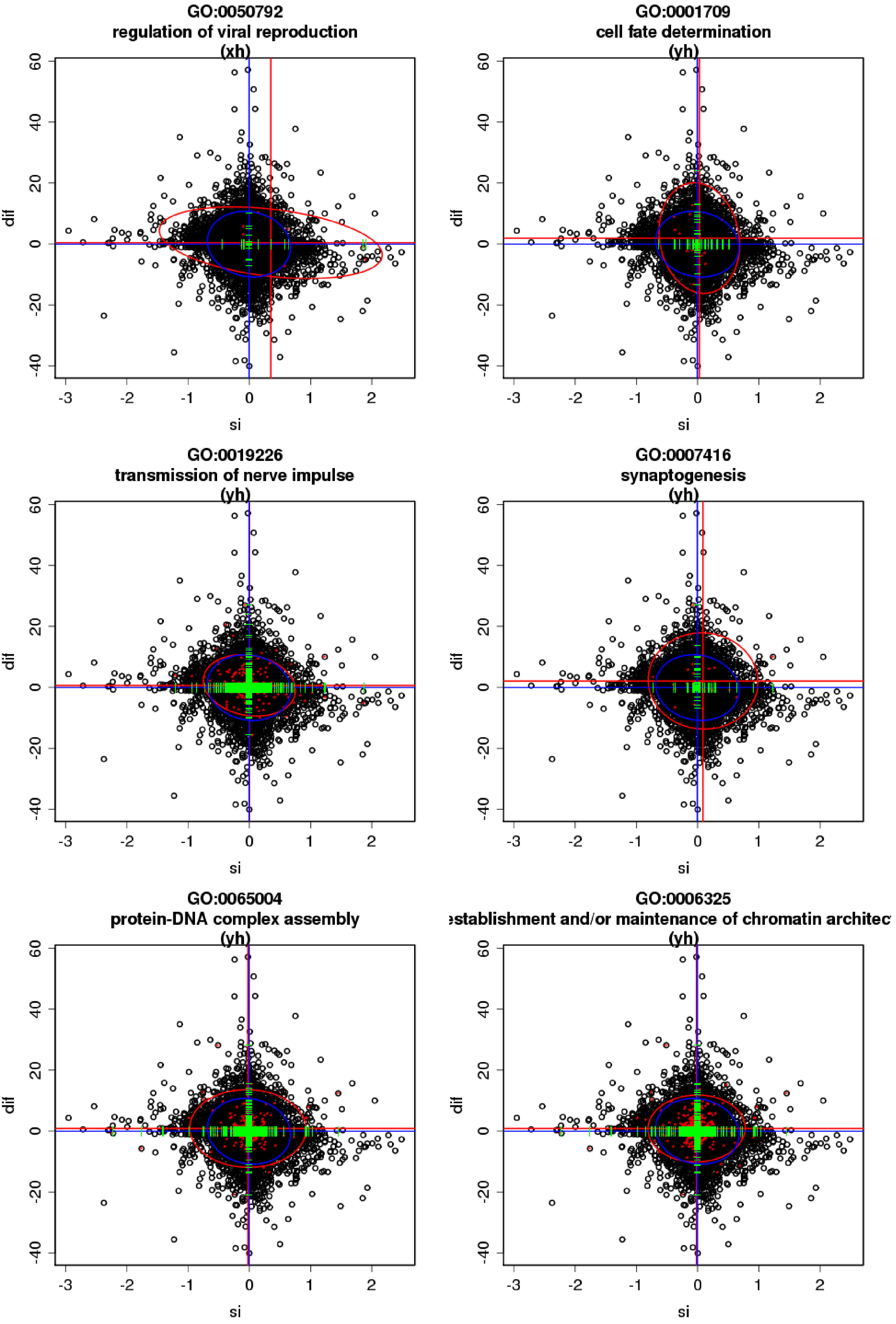
GO:0050792
regulation of viral reproduction
(xh)
GO:0001709
cell fate determination
(yh)
GO:0019226
transmission of nerve impulse
(yh)
GO:0007416
synaptogenesis
(yh)
GO:0065004
protein-DNA complex assembly
(yh)
GO:0006325
establishment and/or maintenance of chromatin architec
(yh)
dif
si
60
40
20
0
-20
-40
-3
-2
-1
0
1
2

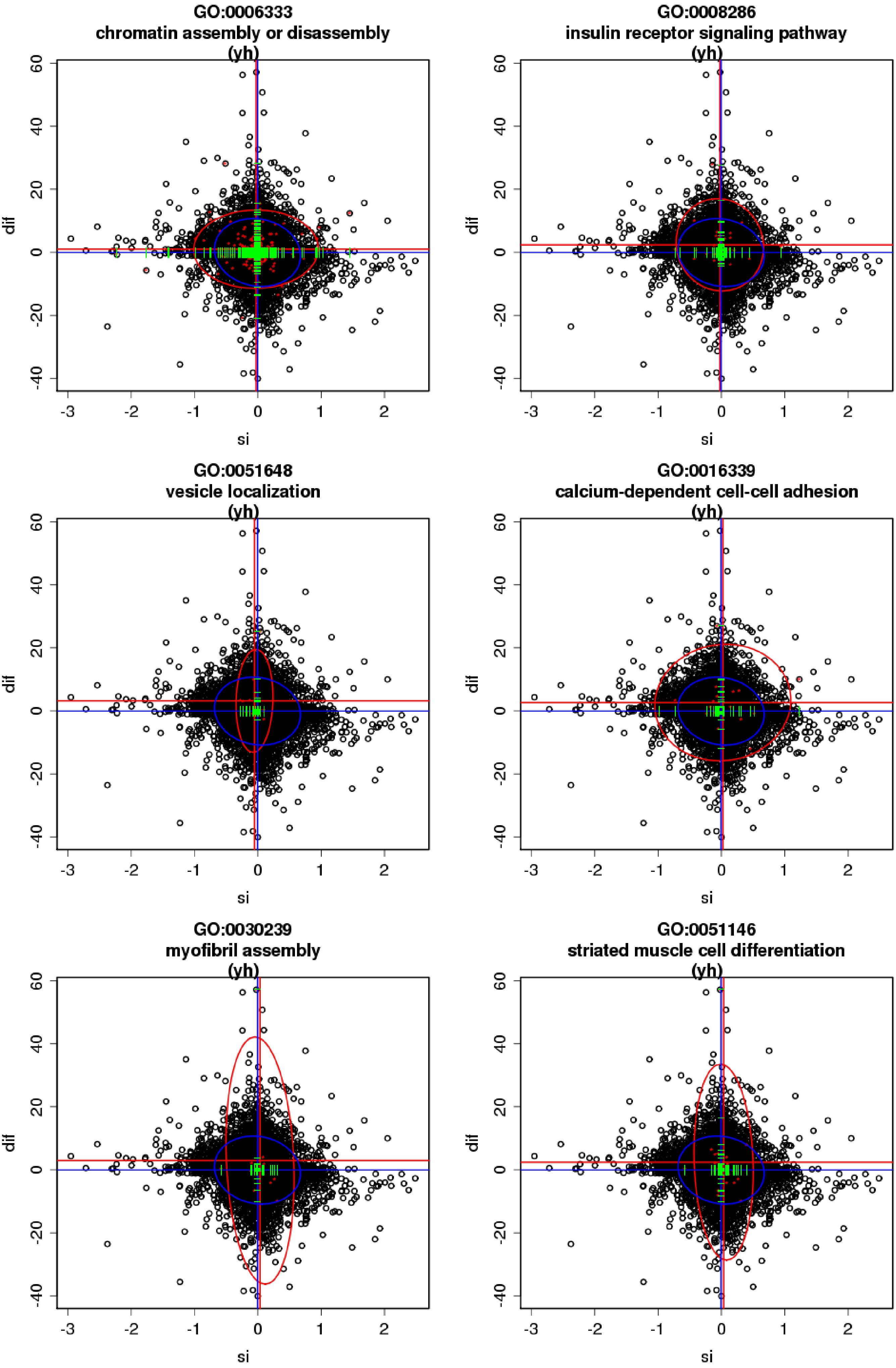
GO:0006333
chromatin assembly or disassembly
(yh)
GO:0008286
insulin receptor signaling pathway
(yh)
GO:0051648
vesicle localization
(yh)
GO:0016339
calcium-dependent cell-cell adhesion
(yh)
GO:0030239
myofibril assembly
(yh)
GO:0051146
striated muscle cell differentiation
(yh)
dif
si
60
40
20
0
-20
-40
-3
-2
-1
0
1
2

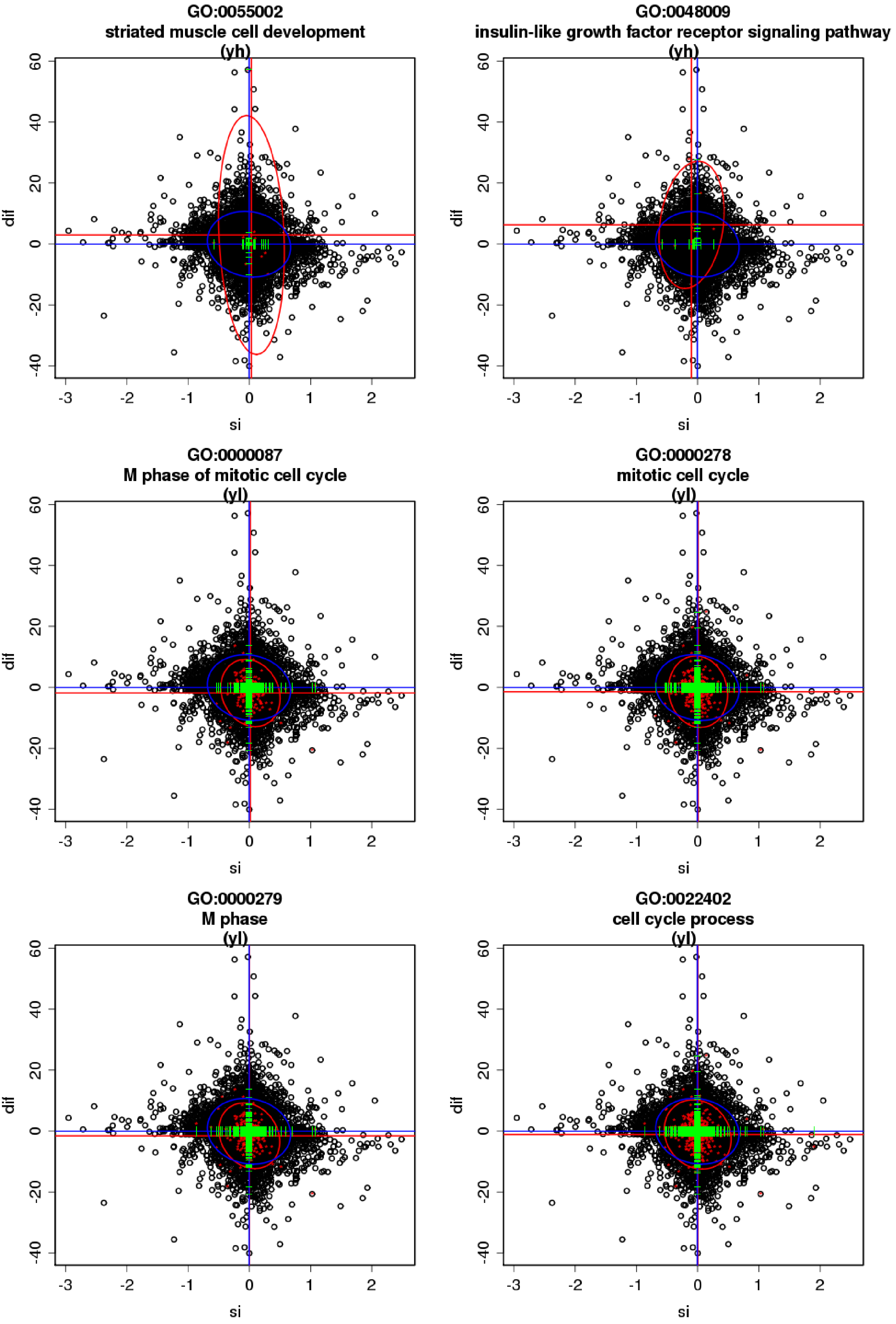
GO:0055002
striated muscle cell development
(yh)
GO:0048009
insulin-like growth factor receptor signaling pathway
(yh)
GO:0000087
M phase of mitotic cell cycle
(yl)
GO:0000278
mitotic cell cycle
(yl)
GO:0000279
M phase
(yl)
GO:0022402
cell cycle process
(yl)
dif
si
60
40
20
0
-20
-40
-3
-2
-1
0
1
2

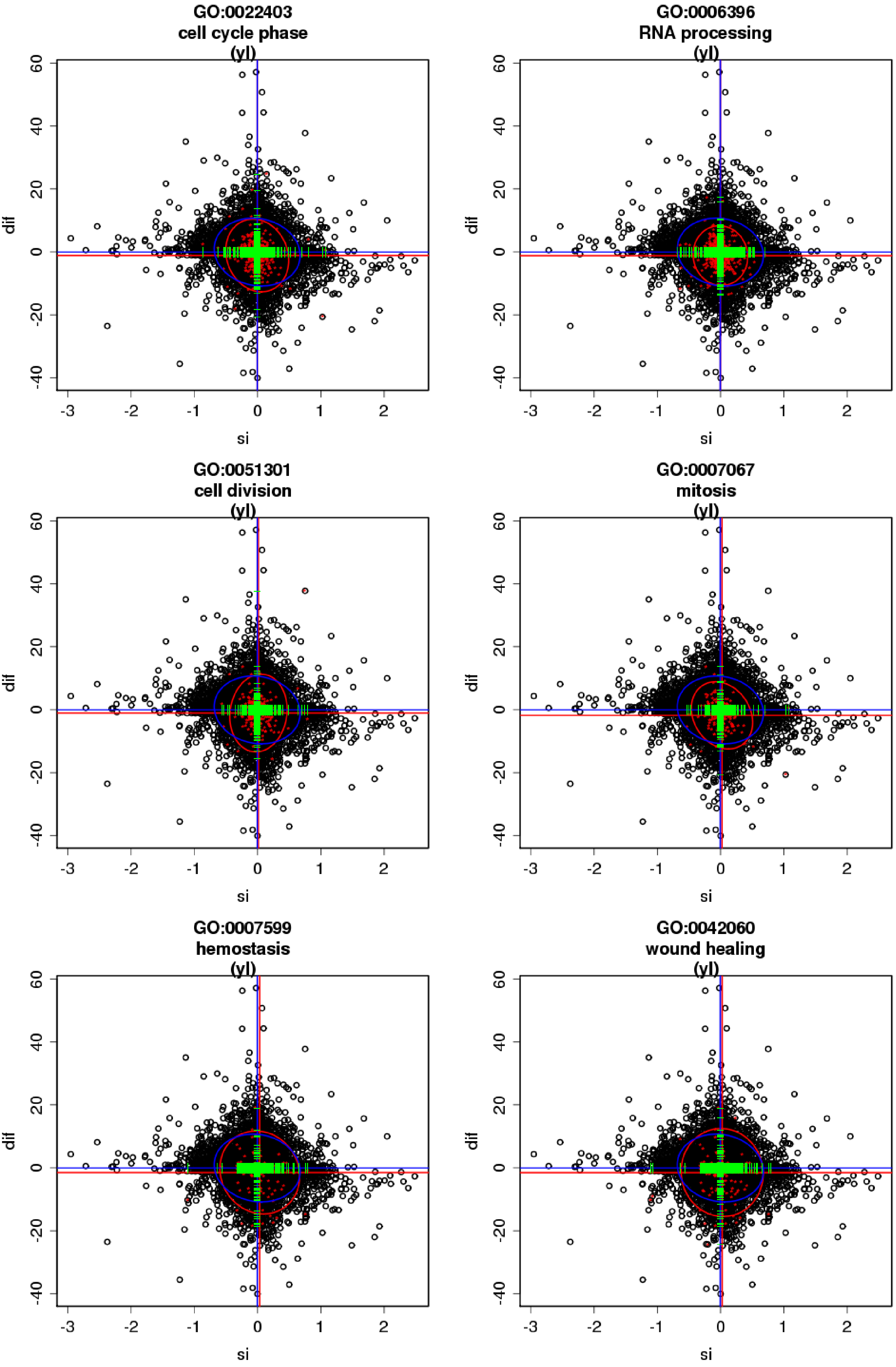
GO:0022403
cell cycle phase
(yl)
GO:0006396
RNA processing
(yl)
GO:0051301
cell division
(yl)
GO:0007067
mitosis
(yl)
GO:0007599
hemostasis
(yl)
GO:0042060
wound healing
(yl)
dif
si
60
40
20
0
-20
-40
-3
-2
-1
0
1
2

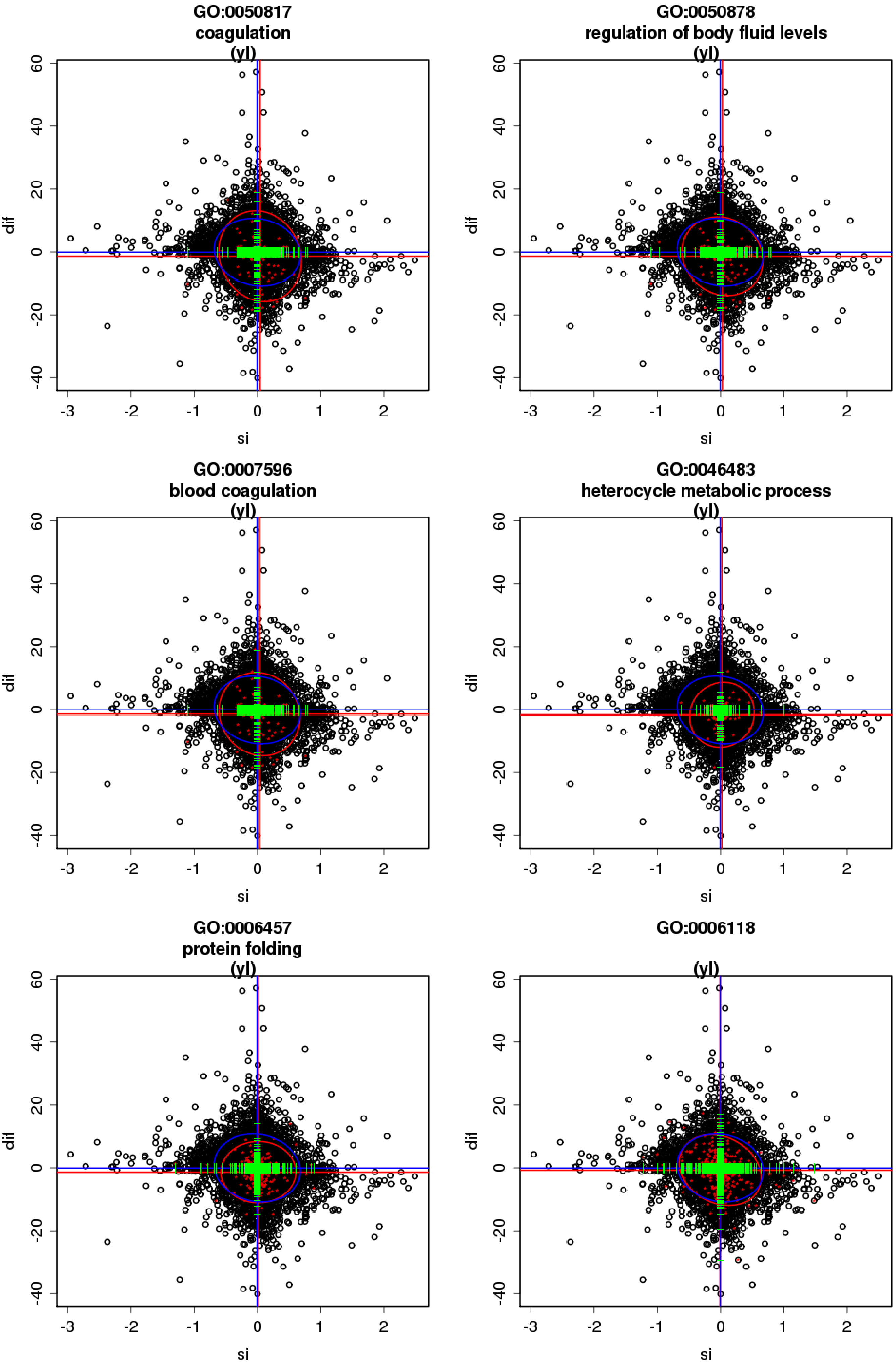
GO:0050817
coagulation
(yl)
GO:0050878
regulation of body fluid levels
(yl)
GO:0007596
blood coagulation
(yl)
GO:0046483
heterocycle metabolic process
(yl)
GO:0006457
protein folding
(yl)
GO:0006118
(yl)
dif
si
60
40
20
0
-20
-40
-3
-2
-1
0
1
2

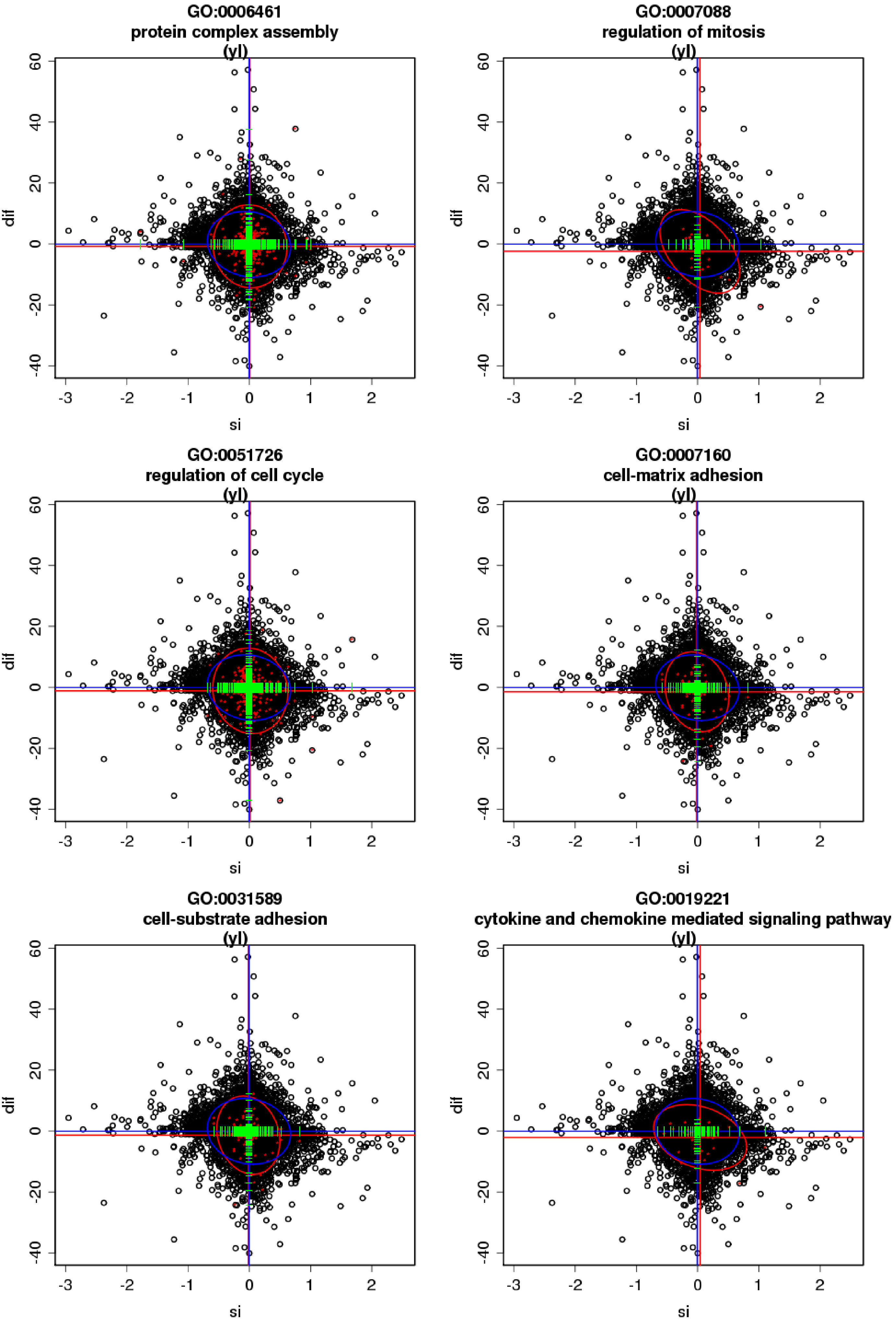
GO:0006461
protein complex assembly
(yl)
GO:0007088
regulation of mitosis
(yl)
GO:0051726
regulation of cell cycle
(yl)
GO:0007160
cell-matrix adhesion
(yl)
GO:0031589
cell-substrate adhesion
(yl)
GO:0019221
cytokine and chemokine mediated signaling pathway
(yl)
dif
si
60
40
20
0
-20
-40
-3
-2
-1
0
1
2

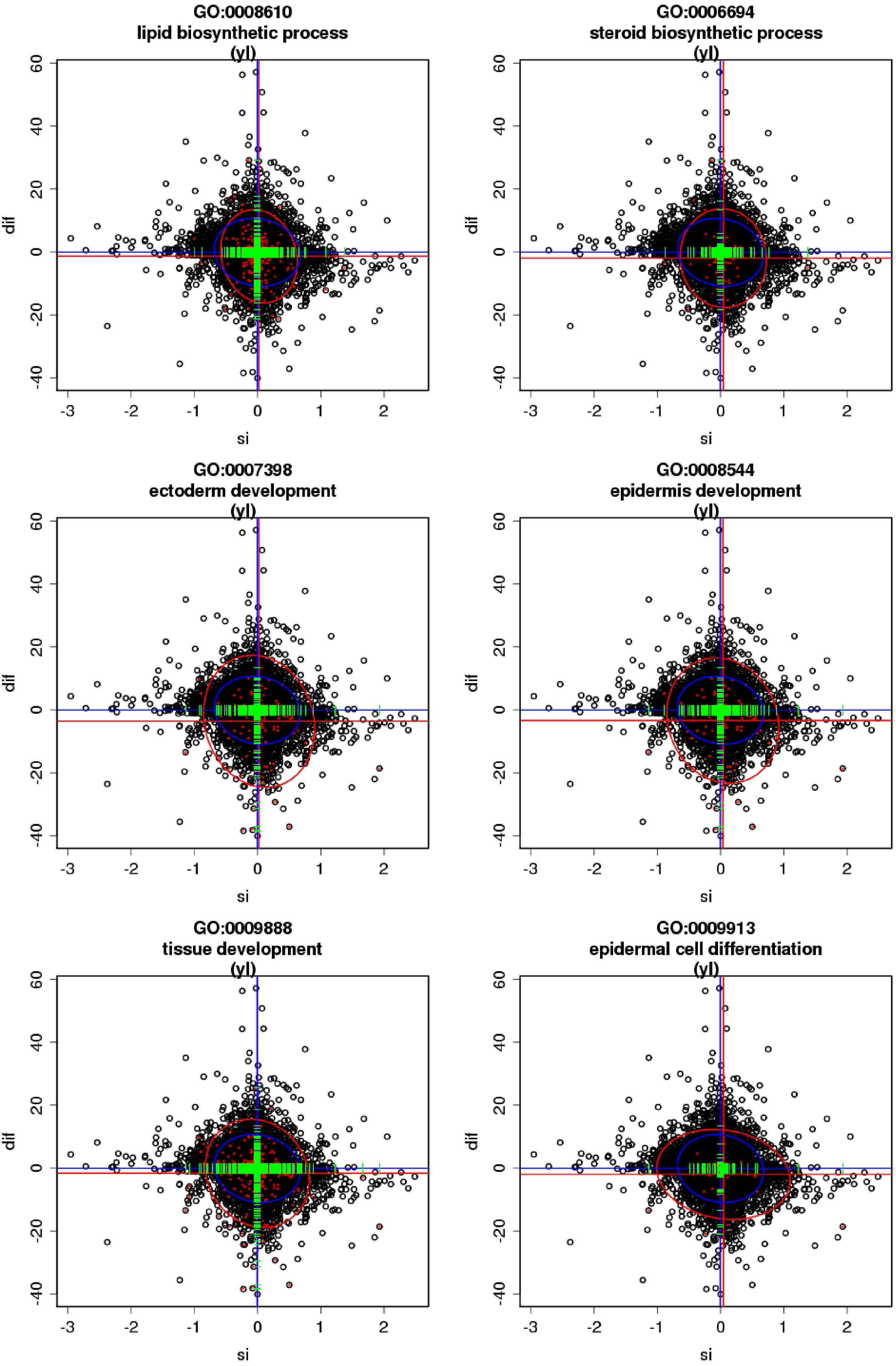
GO:0008610
lipid biosynthetic process
(yl)
GO:0006694
steroid biosynthetic process
(yl)
GO:0007398
ectoderm development
(yl)
GO:0008544
epidermis development
(yl)
GO:0009888
tissue development
(yl)
GO:0009913
epidermal cell differentiation
(yl)
dif
si
60
40
20
0
-20
-40
-3
-2
-1
0
1
2

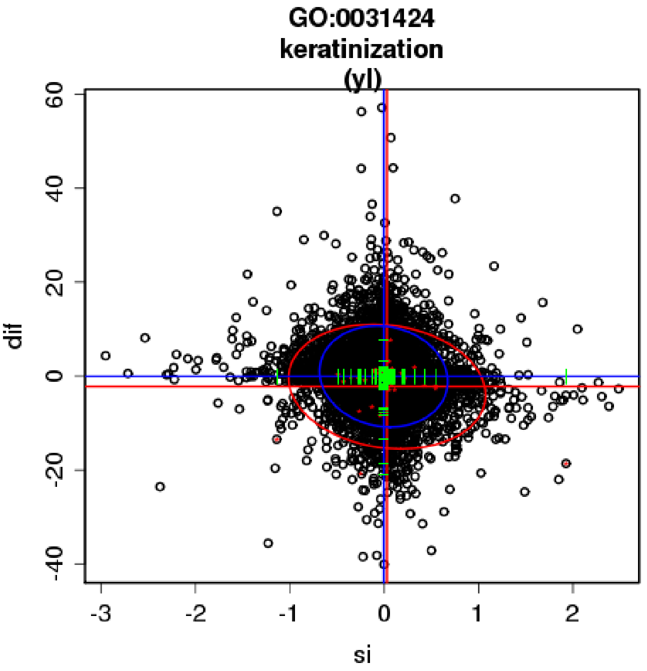
GO:0031424
keratinization
(yl)
dif
si
60
40
20
0
-20
-40
-3
-2
-1
0
1
2

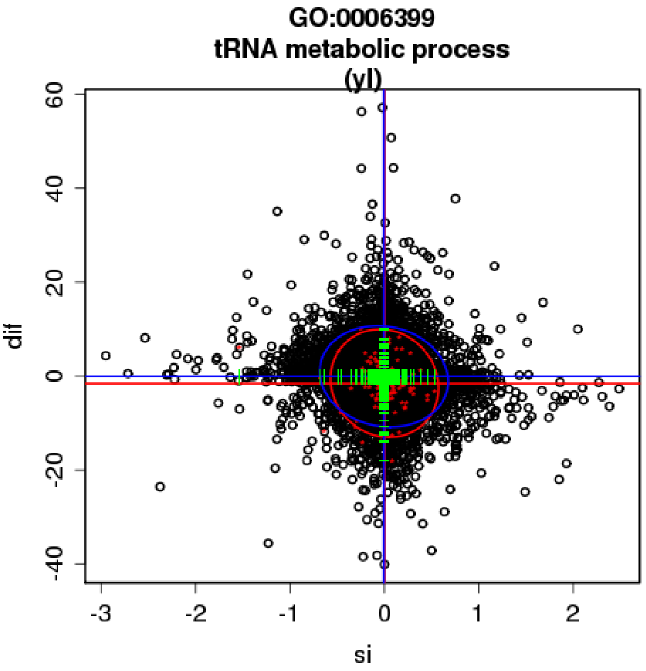
GO:0006399
tRNA metabolic process
(yl)
dif
si
60
40
20
0
-20
-40
-3
-2
-1
0
1
2

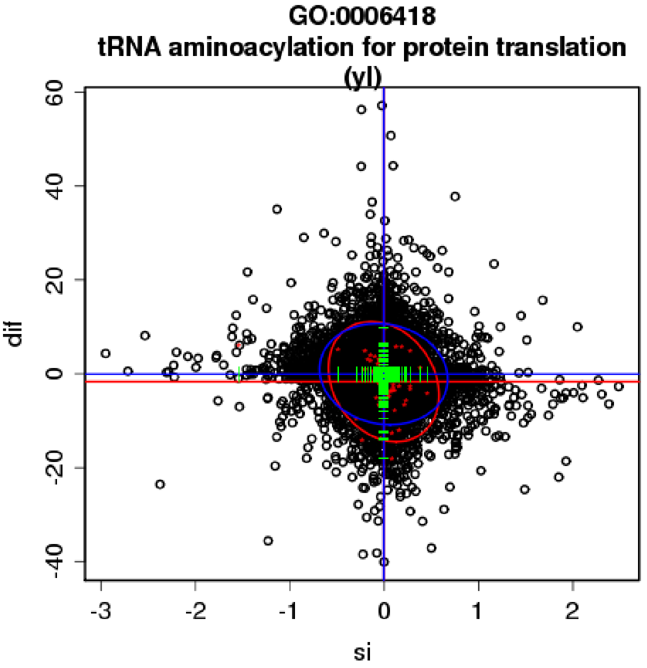
GO:0006418
tRNA aminoacylation for protein translation
(yl)
dif
si
60
40
20
0
-20
-40
-3
-2
-1
0
1
2

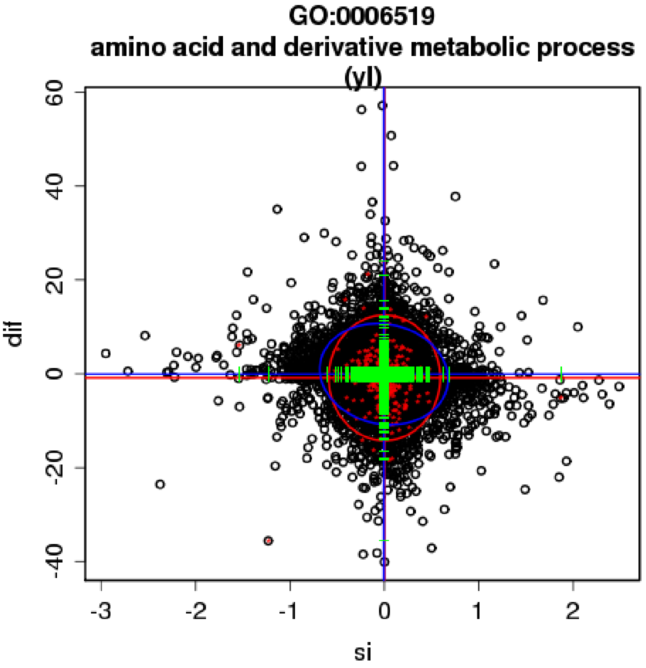
GO:0006519
amino acid and derivative metabolic process
(yl)
dif
si
60
40
20
0
-20
-40
-3
-2
-1
0
1
2

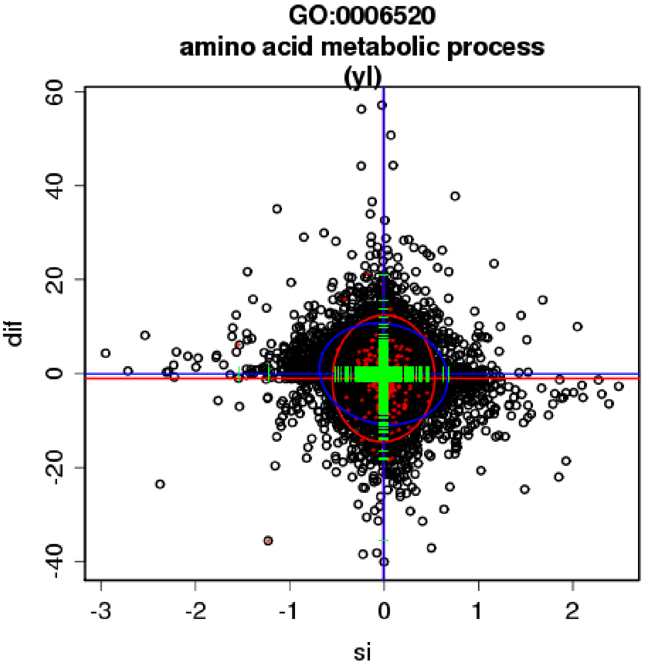
GO:0006520
amino acid metabolic process
(yl)
dif
si
60
40
20
0
-20
-40
-3
-2
-1
0
1
2

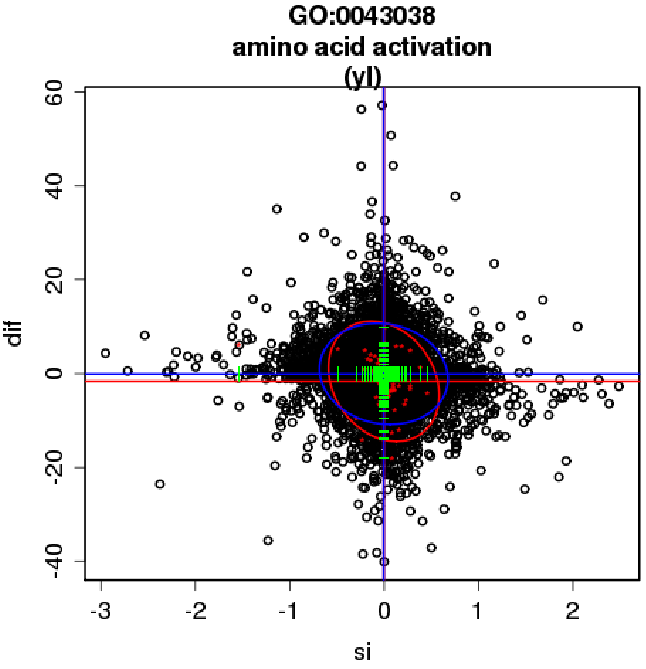
GO:0043038
amino acid activation
(yl)
dif
si
60
40
20
0
-20
-40
-3
-2
-1
0
1
2

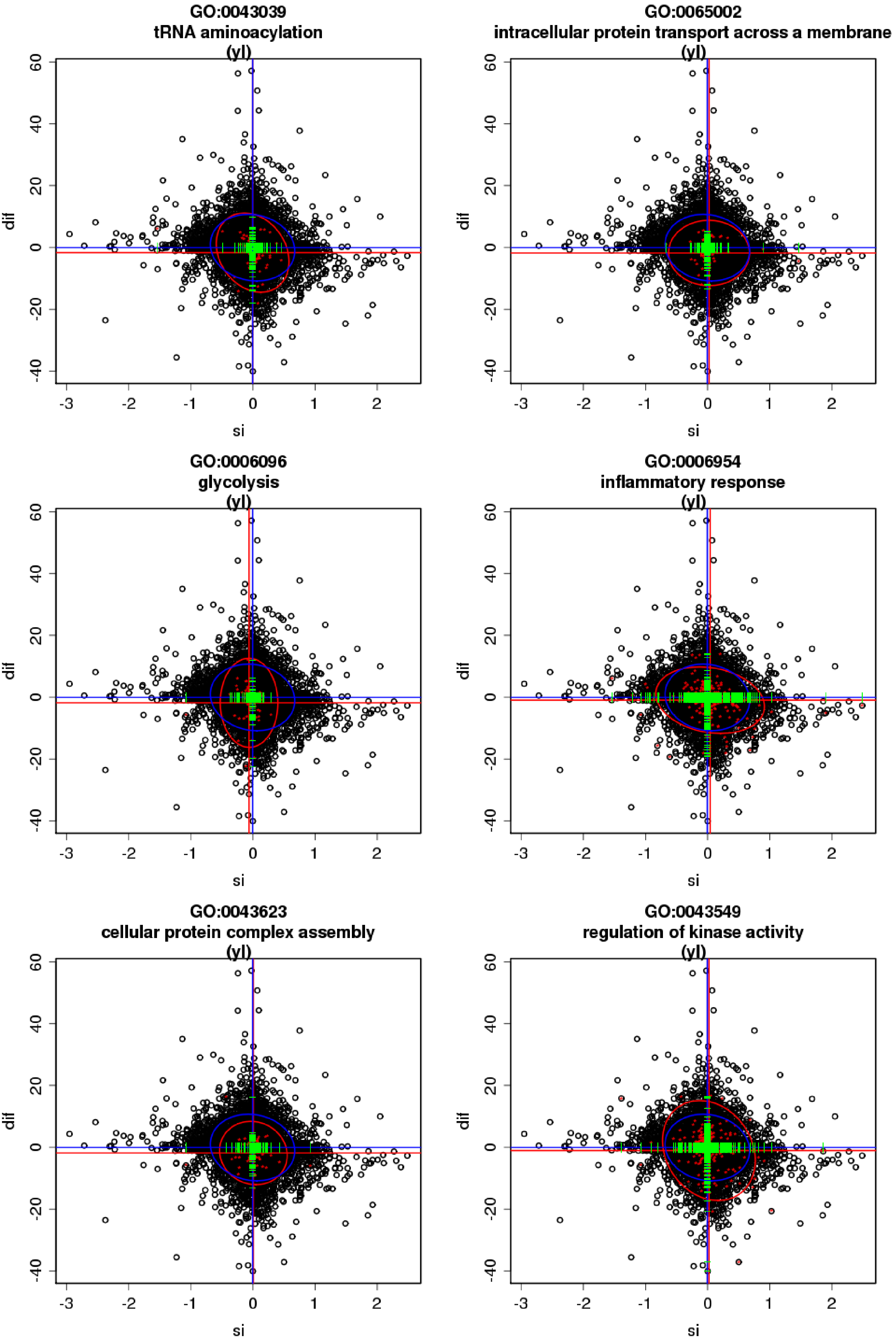
GO:0043039
tRNA aminoacylation
(yl)
GO:0065002
intracellular protein transport across a membrane
(yl)
GO:0006096
glycolysis
(yl)
GO:0006954
inflammatory response
(yl)
GO:0043623
cellular protein complex assembly
(yl)
GO:0043549
regulation of kinase activity
(yl)
dif
si
60
40
20
0
-20
-40
-3
-2
-1
0
1
2

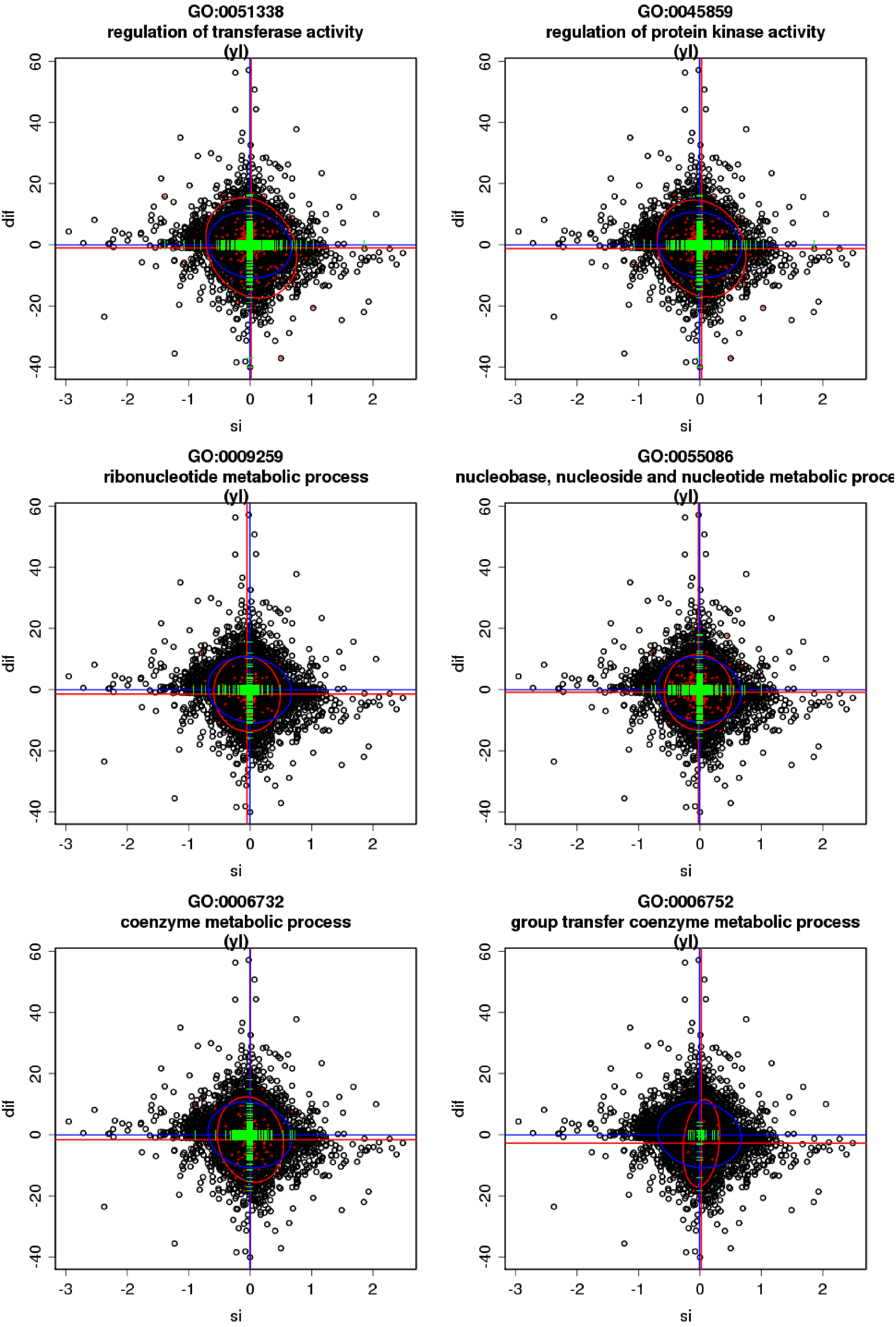
GO:0051338
regulation of transferase activity
(yl)
dif
si
GO:0045859
regulation of protein kinase activity
(yl)
dif
si
GO:0009259
ribonucleotide metabolic process
(yl)
dif
si
GO:0055086
nucleobase, nucleoside and nucleotide metabolic proce
(yl)
dif
si
GO:0006732
coenzyme metabolic process
(yl)
dif
si
GO:0006752
group transfer coenzyme metabolic process
(yl)
dif
si

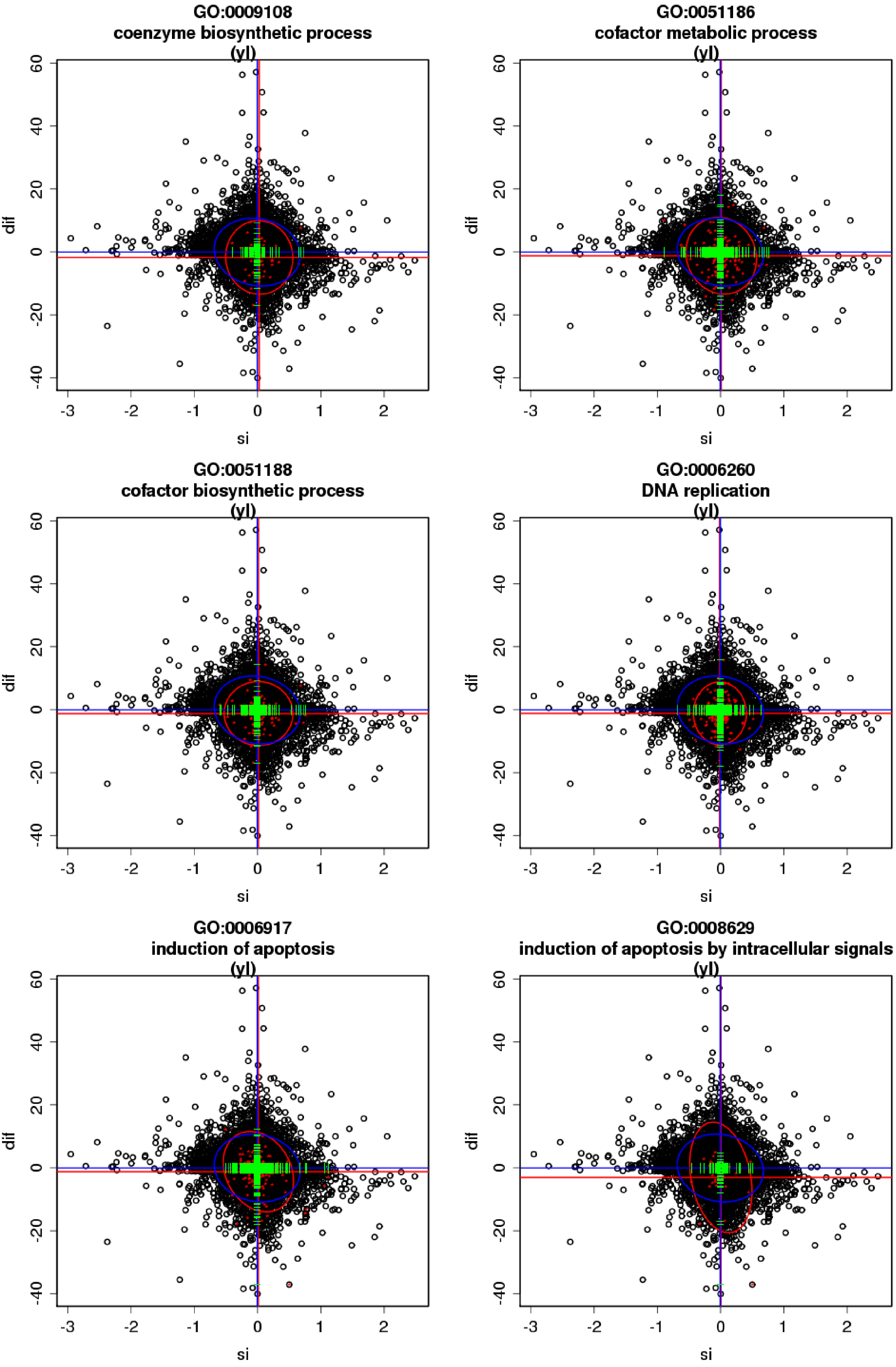
GO:0009108
coenzyme biosynthetic process
(yl)
GO:0051186
cofactor metabolic process
(yl)
GO:0051188
cofactor biosynthetic process
(yl)
GO:0006260
DNA replication
(yl)
GO:0006917
induction of apoptosis
(yl)
GO:0008629
induction of apoptosis by intracellular signals
(yl)
dif
si
60
40
20
0
-20
-40
-3
-2
-1
0
1
2

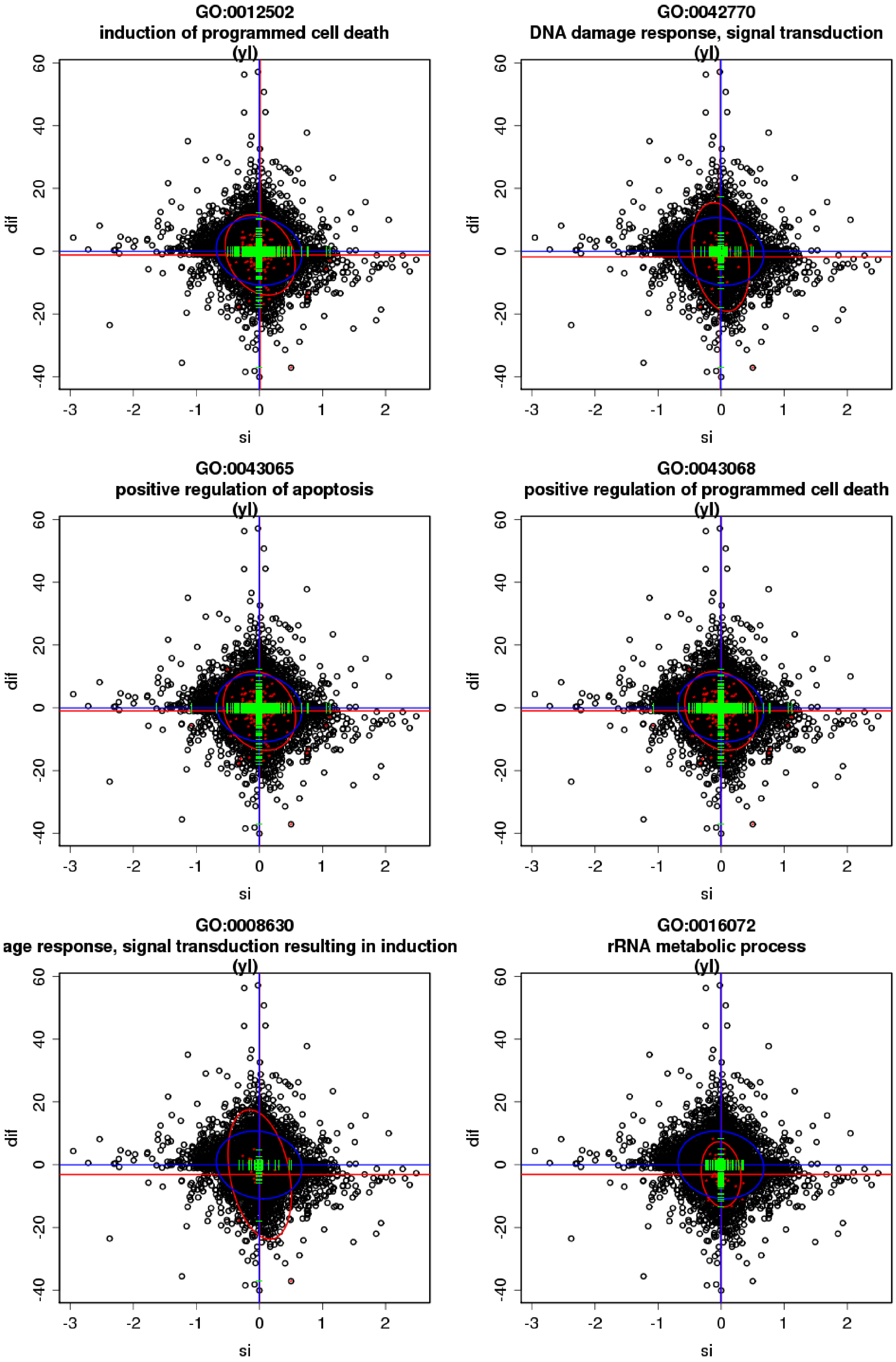
GO:0012502
induction of programmed cell death
(yl)
GO:0042770
DNA damage response, signal transduction
(yl)
GO:0043065
positive regulation of apoptosis
(yl)
GO:0043068
positive regulation of programmed cell death
(yl)
GO:0008630
age response, signal transduction resulting in induction
(yl)
GO:0016072
rRNA metabolic process
(yl)
dif
si
60
40
20
0
-20
-40
-3
-2
-1
0
1
2

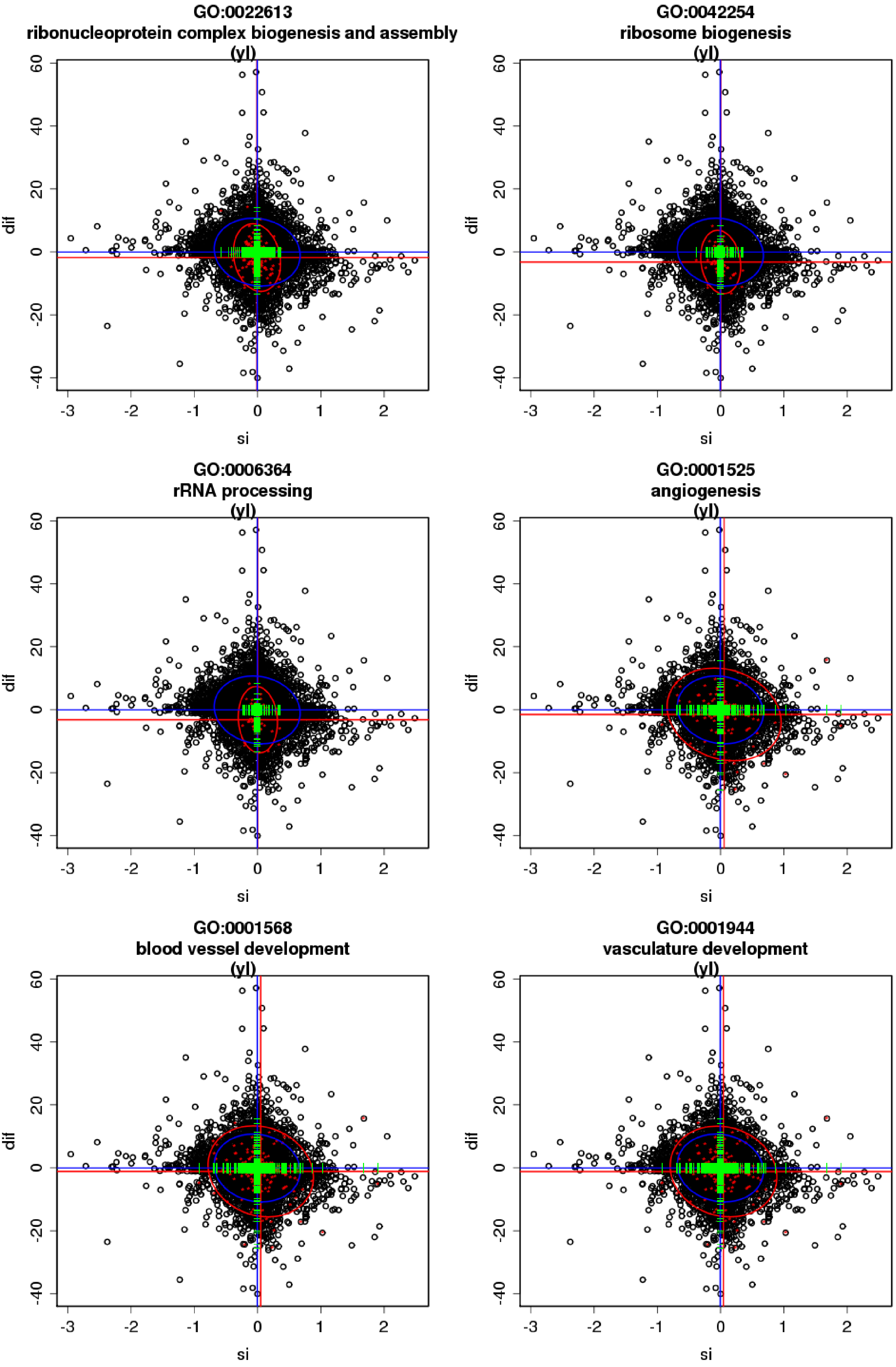
GO:0022613
ribonucleoprotein complex biogenesis and assembly
(yl)
GO:0042254
ribosome biogenesis
(yl)
GO:0006364
rRNA processing
(yl)
GO:0001525
angiogenesis
(yl)
GO:0001568
blood vessel development
(yl)
GO:0001944
vasculature development
(yl)
dif
si
60
40
20
0
-20
-40
-3
-2
-1
0
1
2

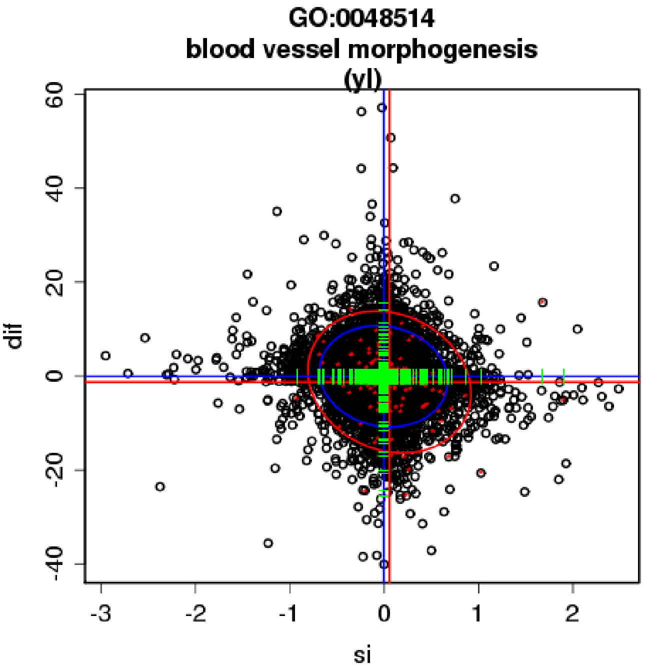
GO:0048514
blood vessel morphogenesis
(yl)
dif
60
40
20
0
-20
-40
-3
-2
-1
0
1
2
si

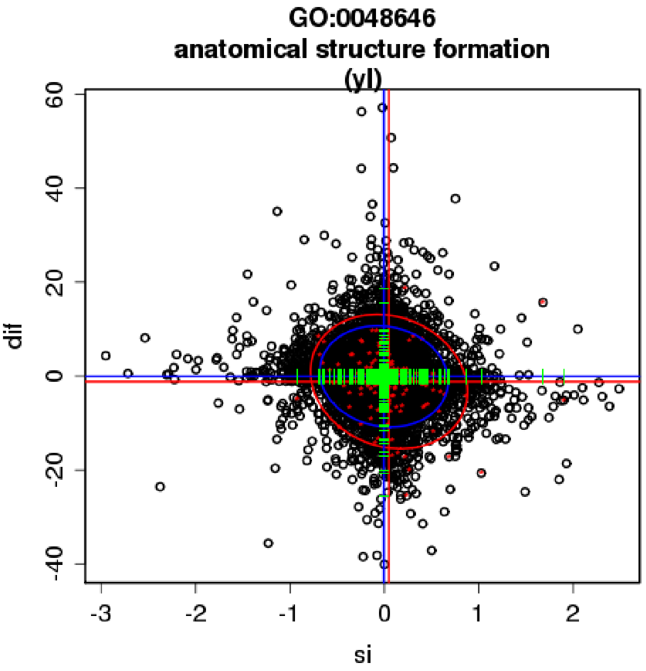
GO:0048646
anatomical structure formation
(yl)
dif
60
40
20
0
-20
-40
-3
-2
-1
0
1
2
si

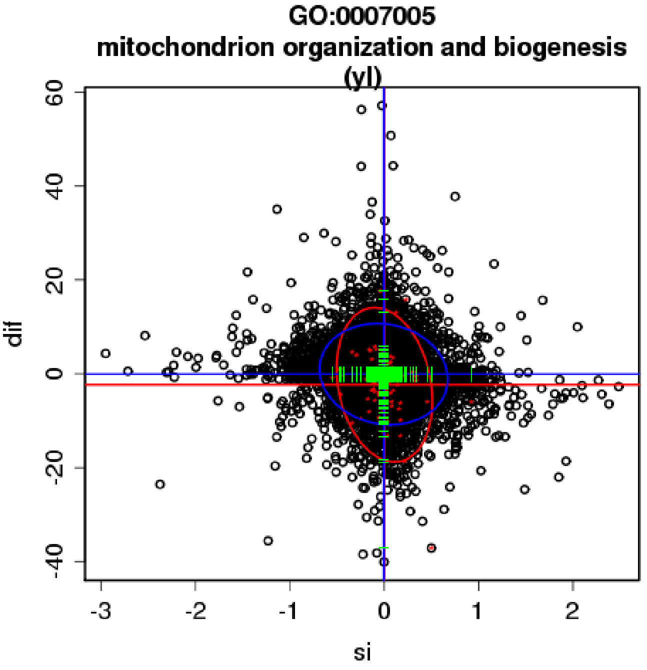
GO:0007005
mitochondrion organization and biogenesis
(yl)
dif
60
40
20
0
-20
-40
-3
-2
-1
0
1
2
si

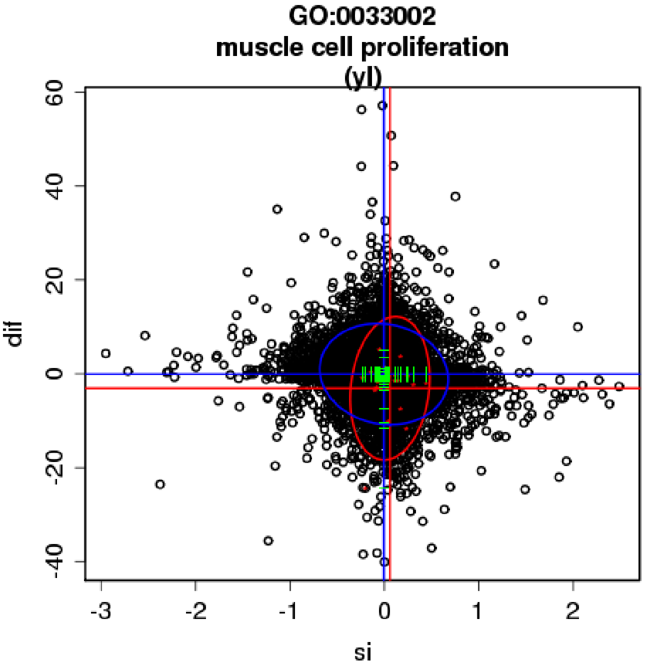
GO:0033002
muscle cell proliferation
(yl)
dif
60
40
20
0
-20
-40
-3
-2
-1
0
1
2
si

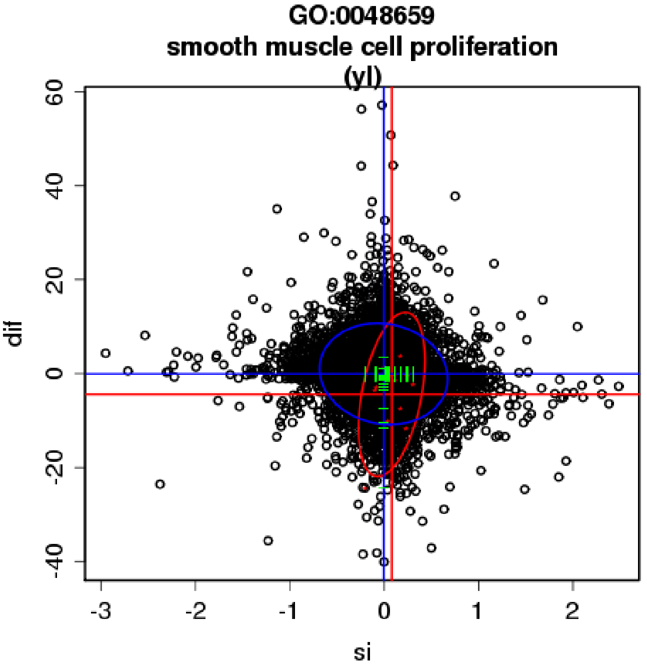
GO:0048659
smooth muscle cell proliferation
(yl)
dif
60
40
20
0
-20
-40
-3
-2
-1
0
1
2
si

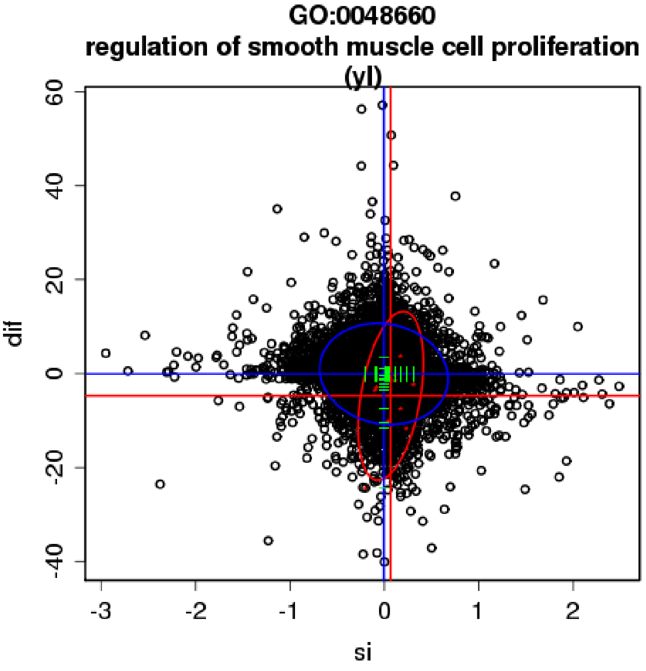
GO:0048660
regulation of smooth muscle cell proliferation
(yl)
dif
60
40
20
0
-20
-40
-3
-2
-1
0
1
2
si

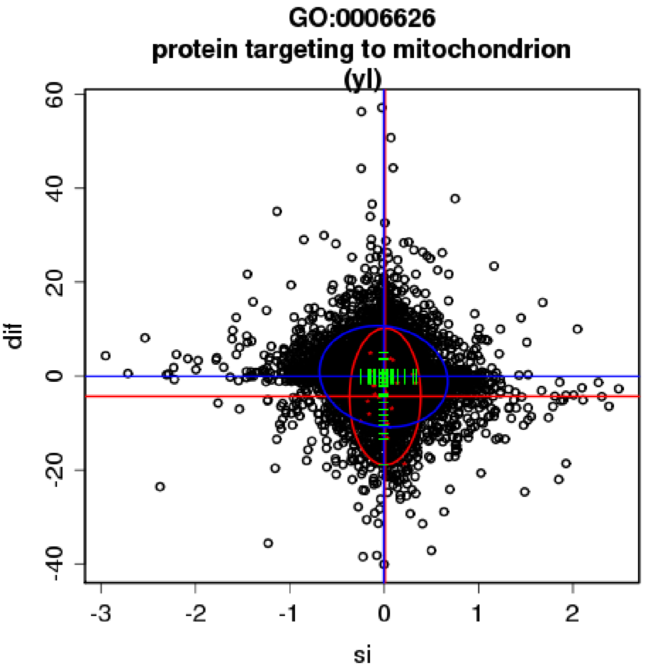
GO:0006626
protein targeting to mitochondrion
(yl)
dif
si
60
40
20
0
-20
-40
-3
-2
-1
0
1
2

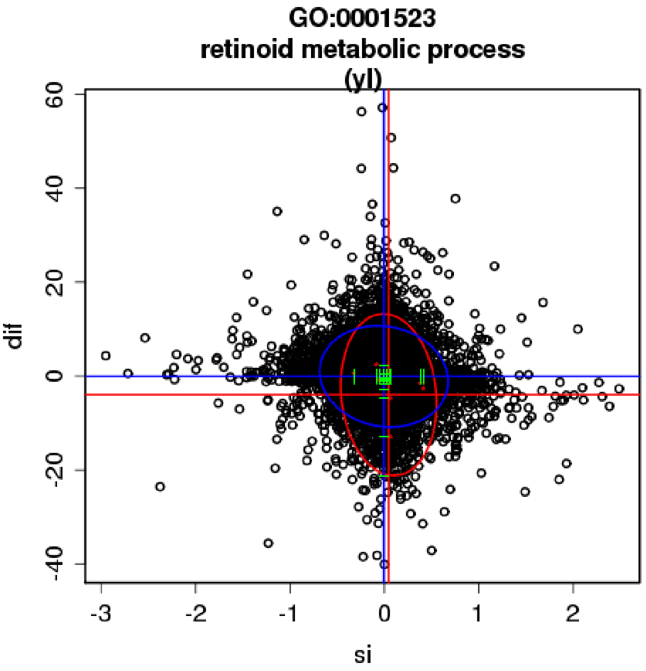
GO:0001523
retinoid metabolic process
(yl)
dif
si
60
40
20
0
-20
-40
-3
-2
-1
0
1
2

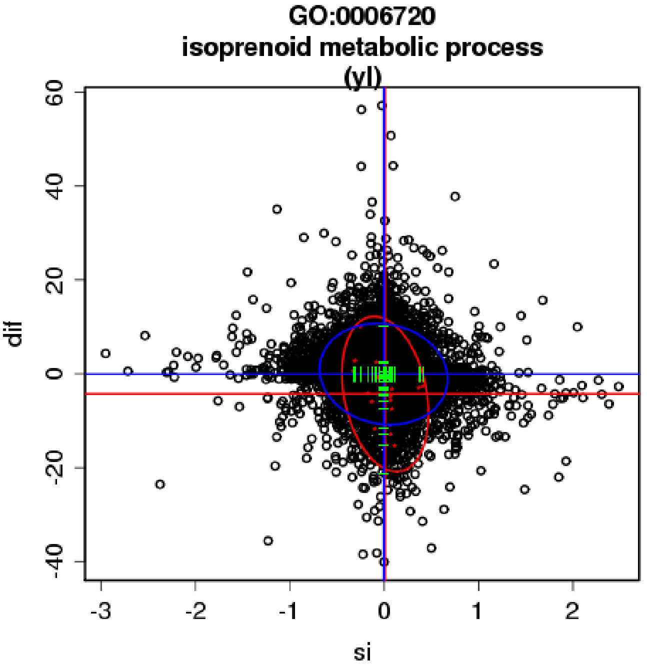
GO:0006720
isoprenoid metabolic process
(yl)
dif
si
60
40
20
0
-20
-40
-3
-2
-1
0
1
2

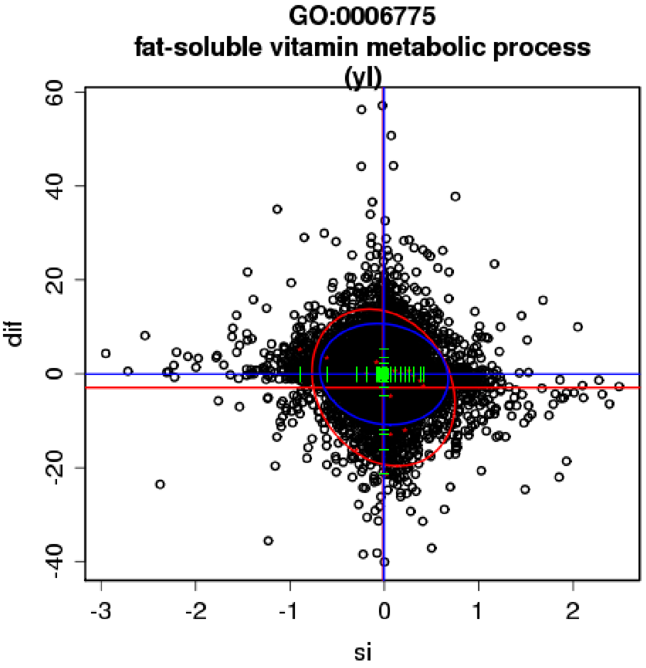
GO:0006775
fat-soluble vitamin metabolic process
(yl)
dif
si
60
40
20
0
-20
-40
-3
-2
-1
0
1
2

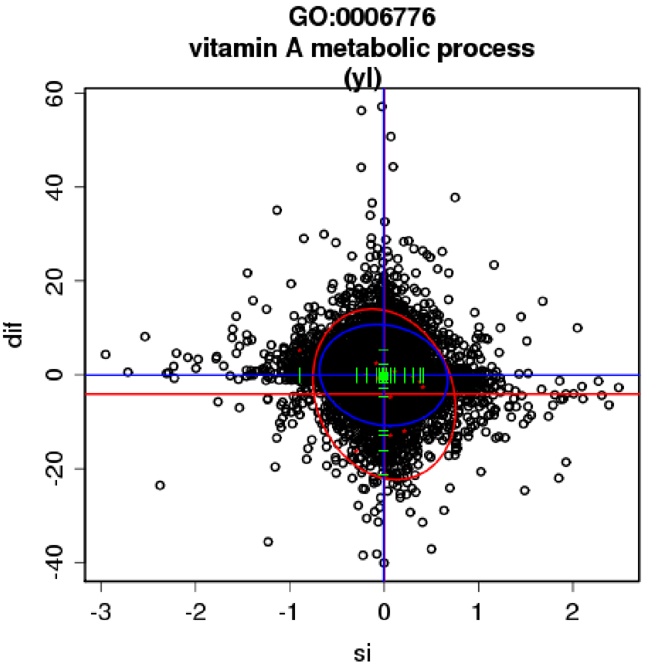
GO:0006776
vitamin A metabolic process
(yl)
dif
si
60
40
20
0
-20
-40
-3
-2
-1
0
1
2

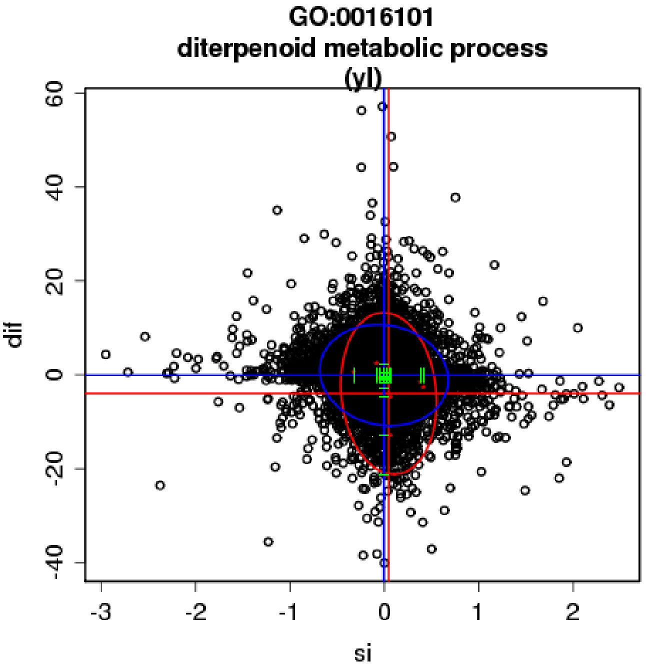
GO:0016101
diterpenoid metabolic process
(yl)
dif
si
60
40
20
0
-20
-40
-3
-2
-1
0
1
2

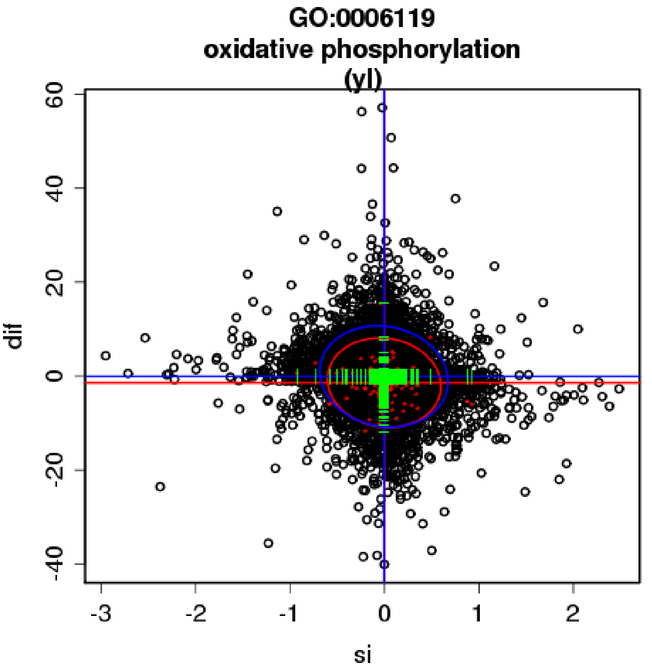
GO:0006119
oxidative phosphorylation
(yl)
dif
si
60
40
20
0
-20
-40
-3
-2
-1
0
1
2

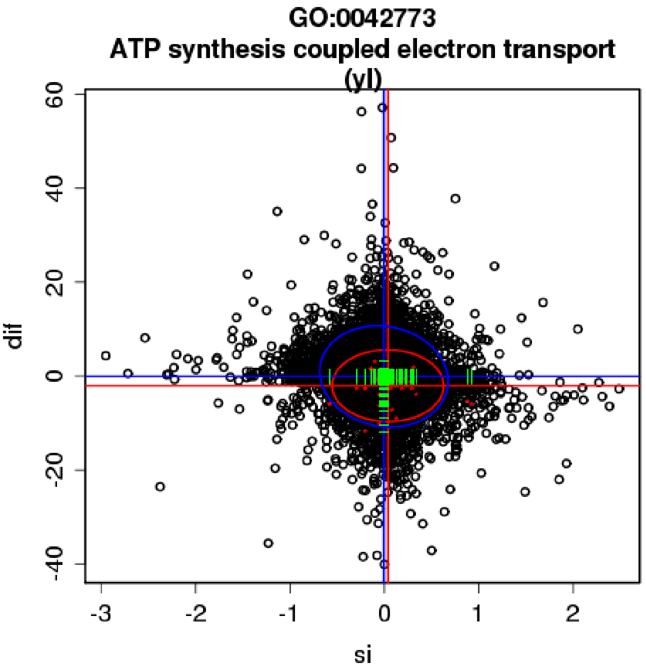
GO:0042773
ATP synthesis coupled electron transport
(yl)
dif
si
60
40
20
0
-20
-40
-3
-2
-1
0
1
2

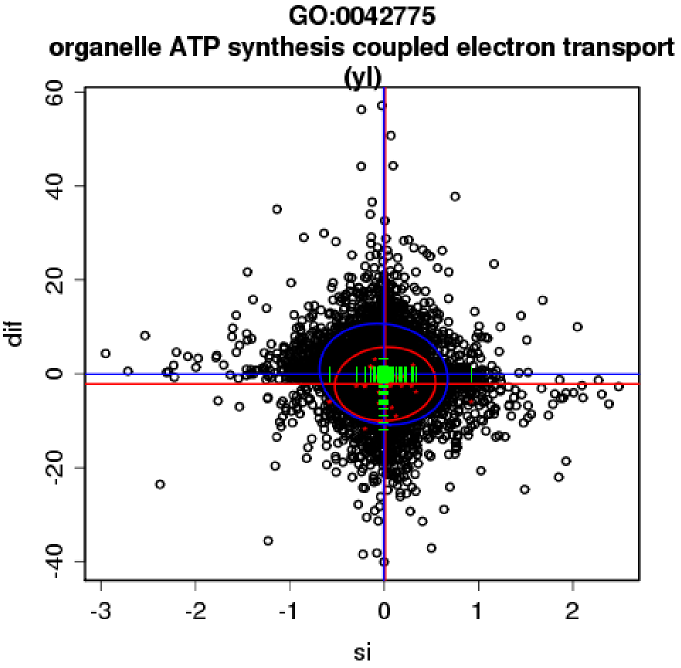
GO:0042775
organelle ATP synthesis coupled electron transport
(yl)
dif
si
60
40
20
0
-20
-40
-3
-2
-1
0
1
2

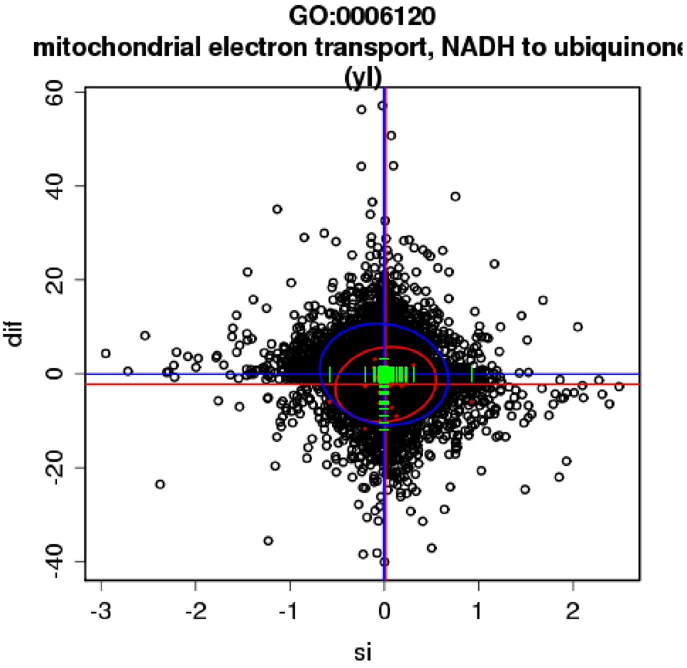
GO:0006120
mitochondrial electron transport, NADH to ubiquinone
(yl)
dif
si
60
40
20
0
-20
-40
-3
-2
-1
0
1
2

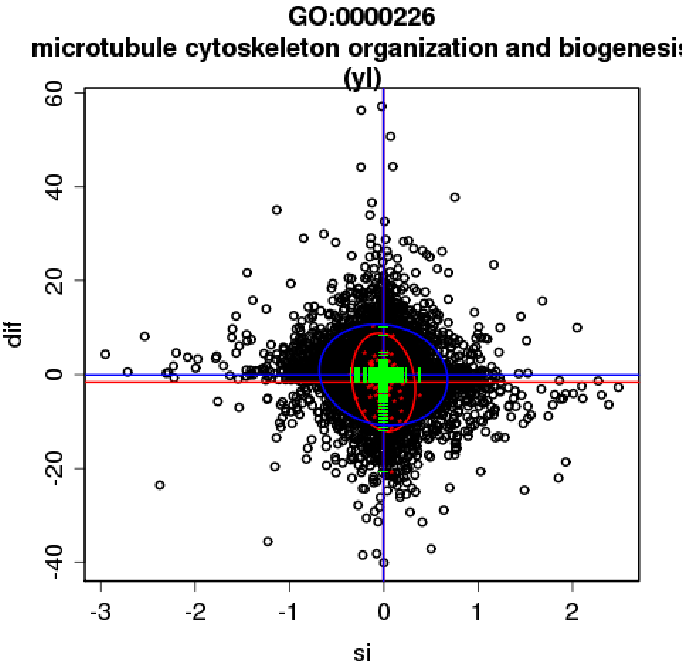
GO:0000226
microtubule cytoskeleton organization and biogenesis
(yl)
dif
si
60
40
20
0
-20
-40
-3
-2
-1
0
1
2

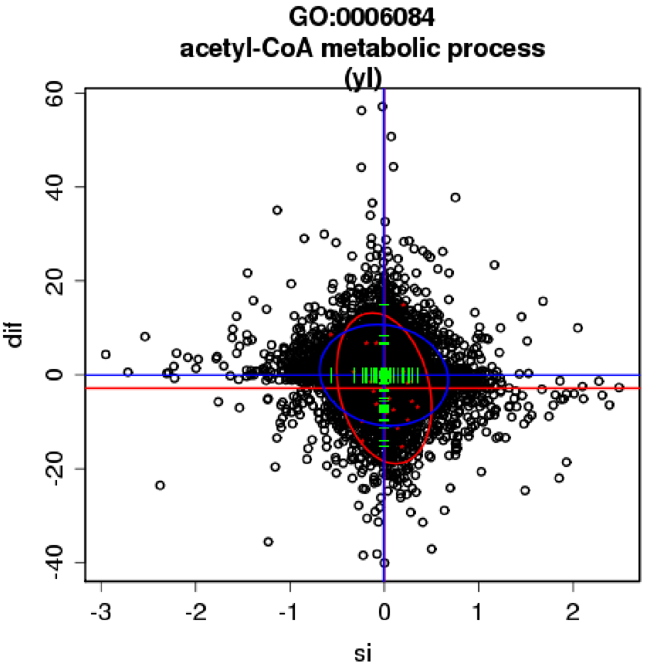
GO:0006084
acetyl-CoA metabolic process
(yl)
dif
si
60
40
20
0
-20
-40
-3
-2
-1
0
1
2

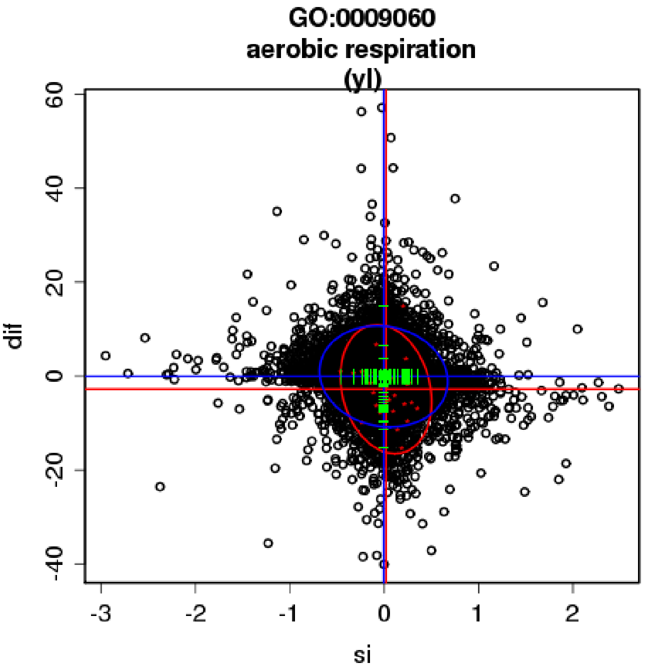
GO:0009060
aerobic respiration
(yl)
dif
si
60
40
20
0
-20
-40
-3
-2
-1
0
1
2

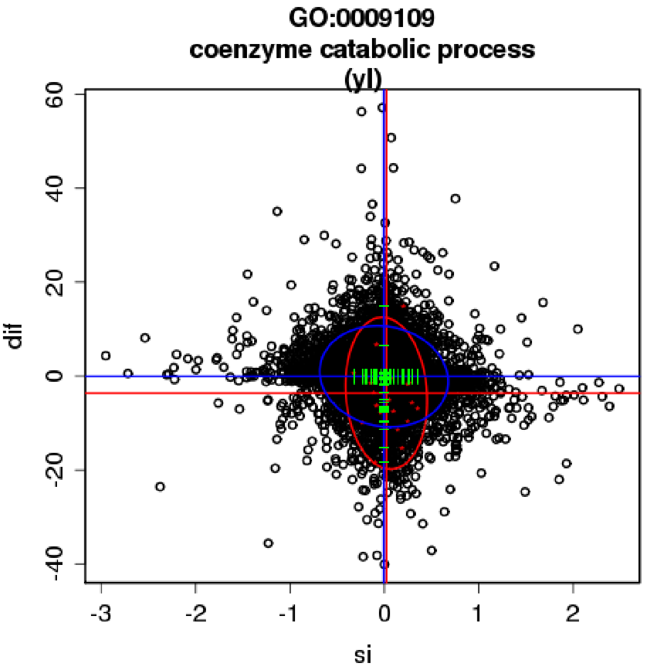
GO:0009109
coenzyme catabolic process
(yl)
dif
si
60
40
20
0
-20
-40
-3
-2
-1
0
1
2

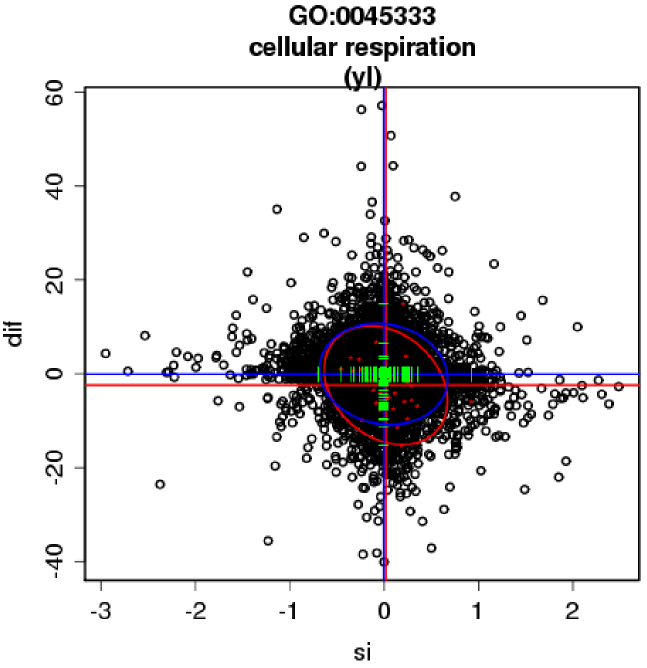
GO:0045333
cellular respiration
(yl)
dif
si
60
40
20
0
-20
-40
-3
-2
-1
0
1
2

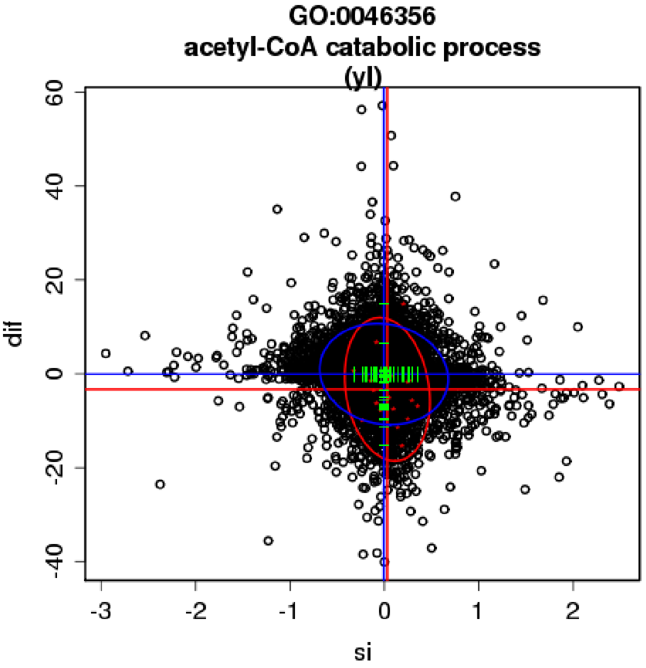
GO:0046356
acetyl-CoA catabolic process
(yl)
dif
si
60
40
20
0
-20
-40
-3
-2
-1
0
1
2

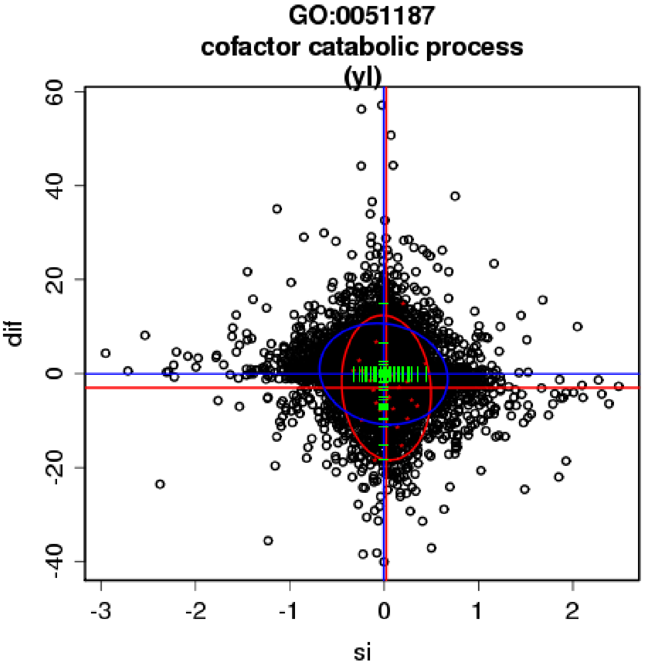
GO:0051187
cofactor catabolic process
(yl)
dif
si
60
40
20
0
-20
-40
-3
-2
-1
0
1
2

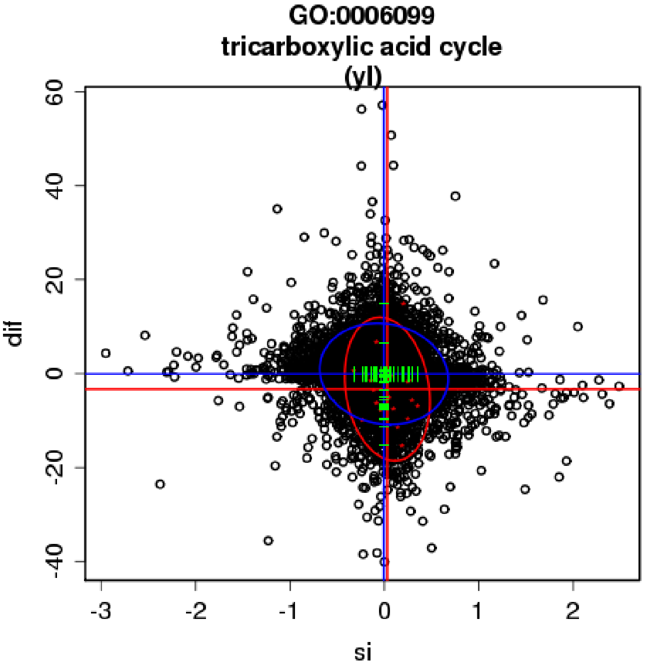
GO:0006099
tricarboxylic acid cycle
(yl)
dif
si
60
40
20
0
-20
-40
-3
-2
-1
0
1
2

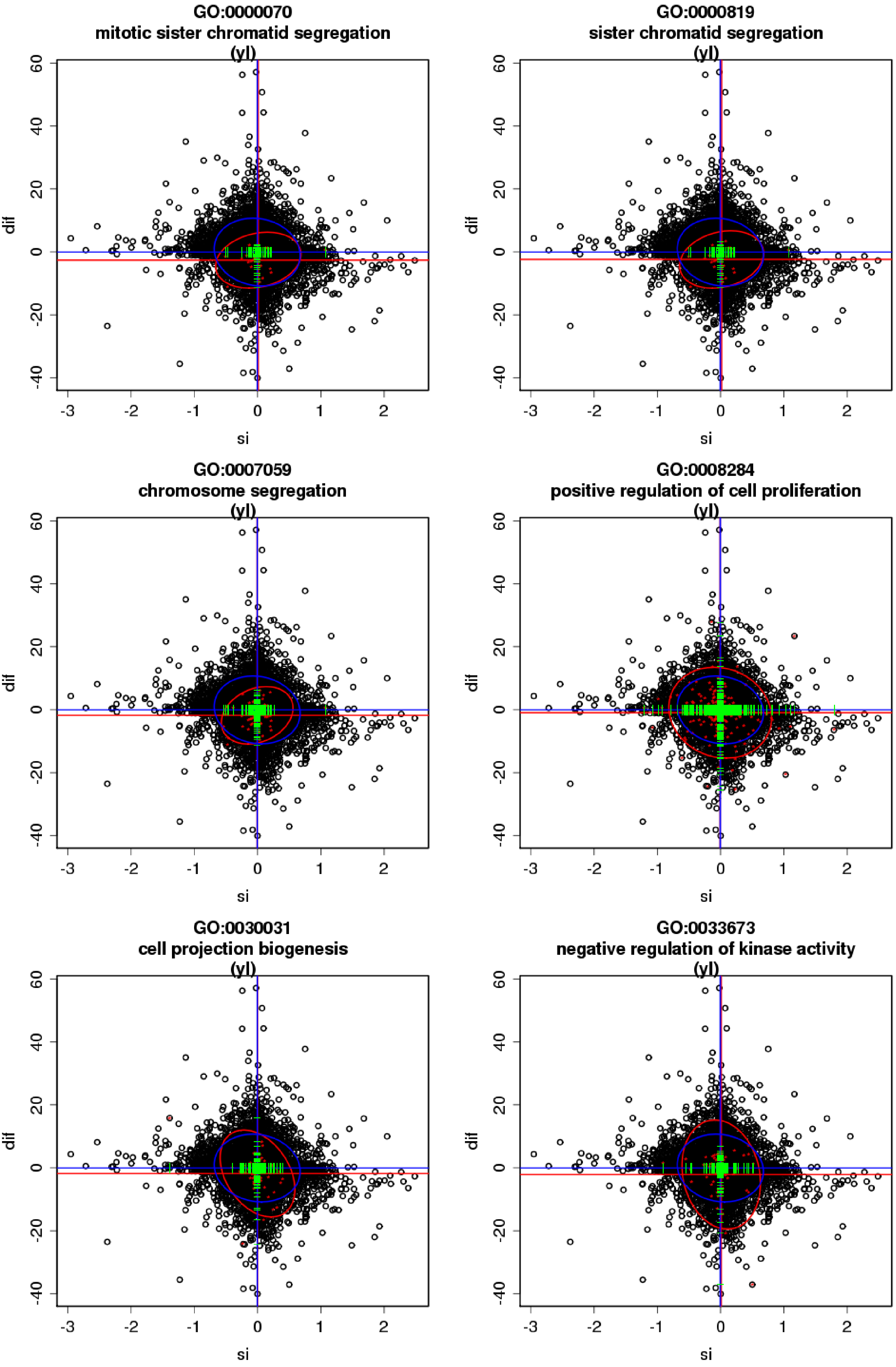
GO:0000070
mitotic sister chromatid segregation
(yl)
GO:0000819
sister chromatid segregation
(yl)
GO:0007059
chromosome segregation
(yl)
GO:0008284
positive regulation of cell proliferation
(yl)
GO:0030031
cell projection biogenesis
(yl)
GO:0033673
negative regulation of kinase activity
(yl)
dif
si
60
40
20
0
-20
-40
-3
-2
-1
0
1
2

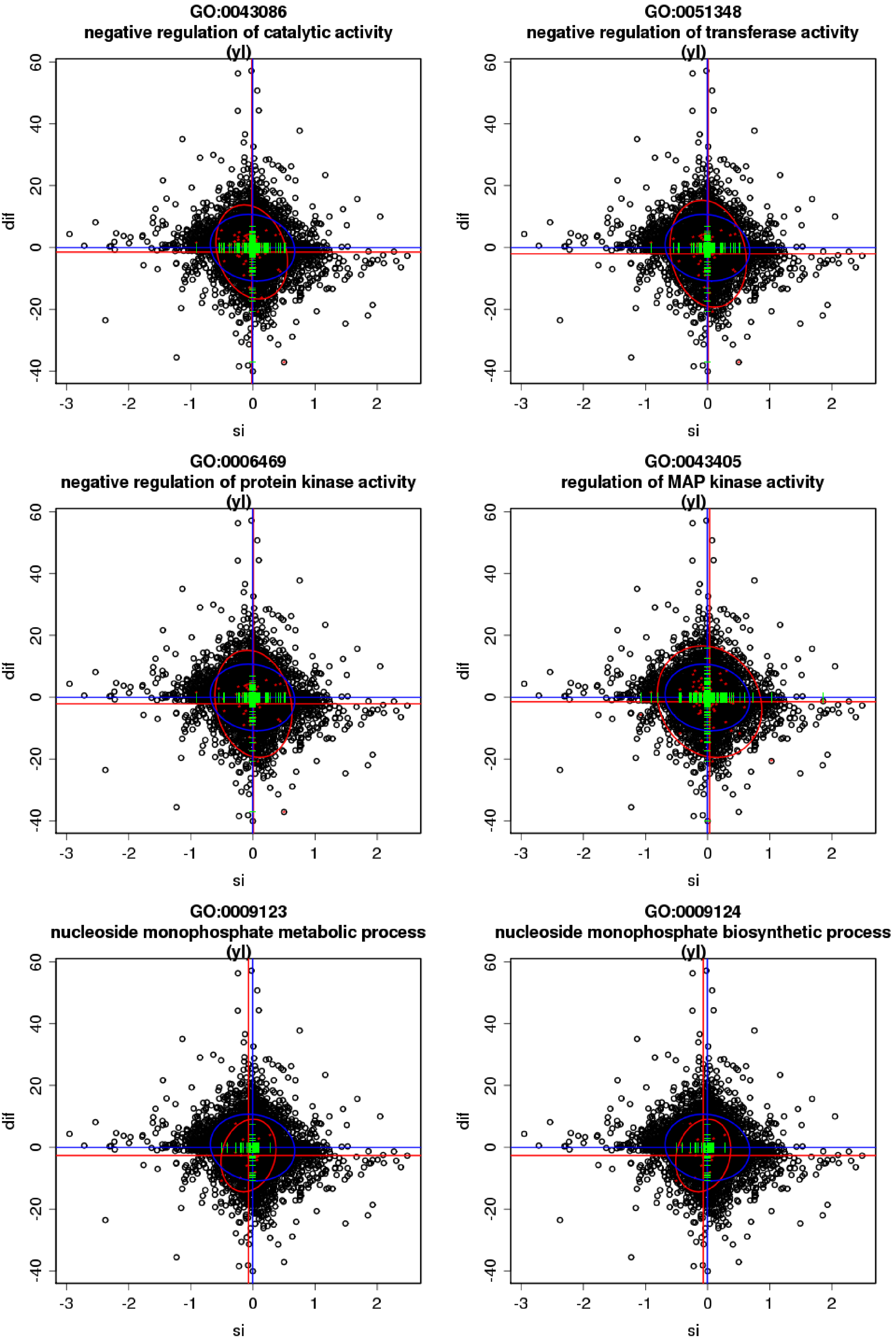
GO:0043086
negative regulation of catalytic activity
(yl)
GO:0051348
negative regulation of transferase activity
(yl)
GO:0006469
negative regulation of protein kinase activity
(yl)
GO:0043405
regulation of MAP kinase activity
(yl)
GO:0009123
nucleoside monophosphate metabolic process
(yl)
GO:0009124
nucleoside monophosphate biosynthetic process
(yl)
dif
si
60
40
20
0
-20
-40
-3
-2
-1
0
1
2

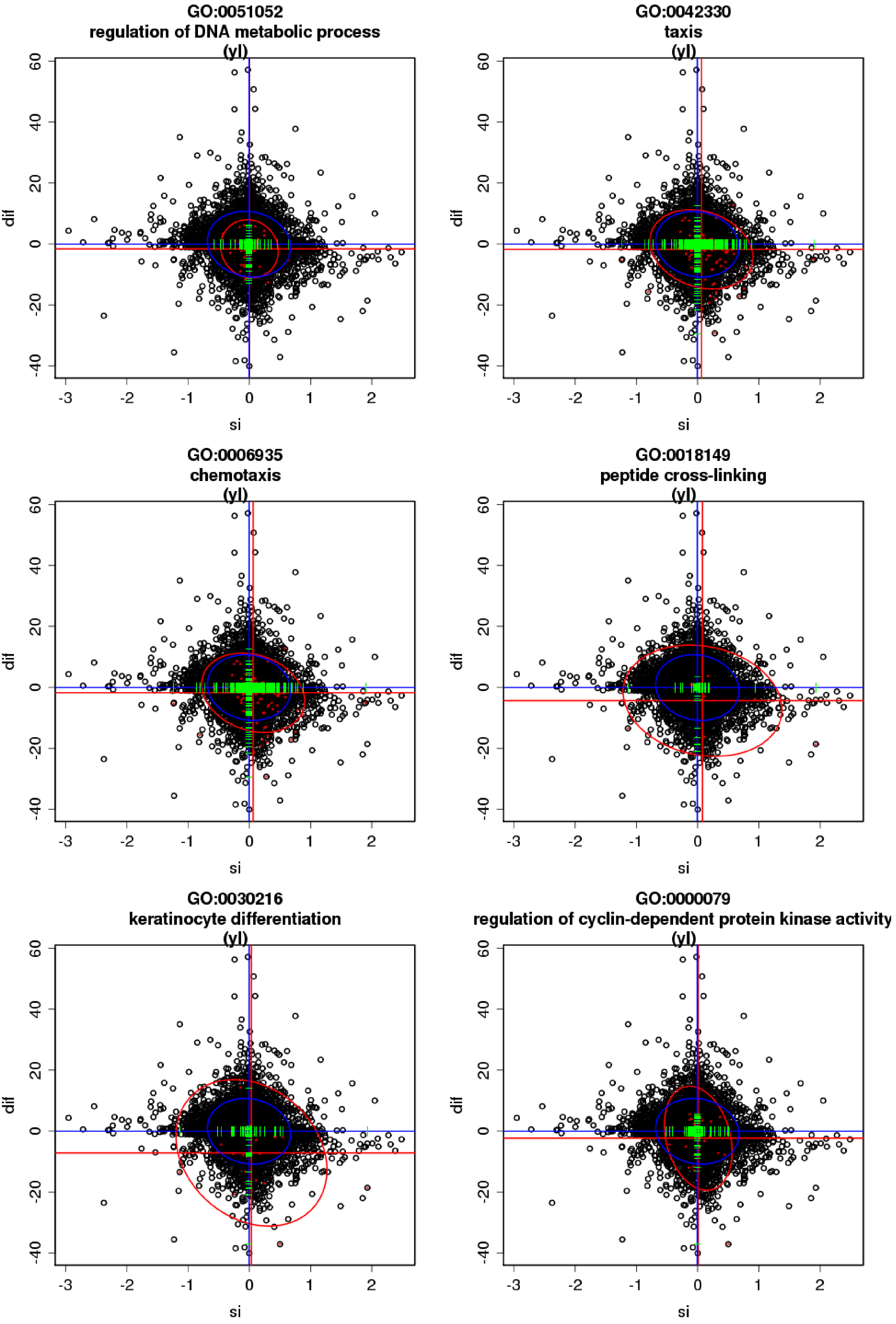
GO:0051052
regulation of DNA metabolic process
(yl)
GO:0042330
taxis
(yl)
GO:0006935
chemotaxis
(yl)
GO:0018149
peptide cross-linking
(yl)
GO:0030216
keratinocyte differentiation
(yl)
GO:0000079
regulation of cyclin-dependent protein kinase activity
(yl)
dif
si

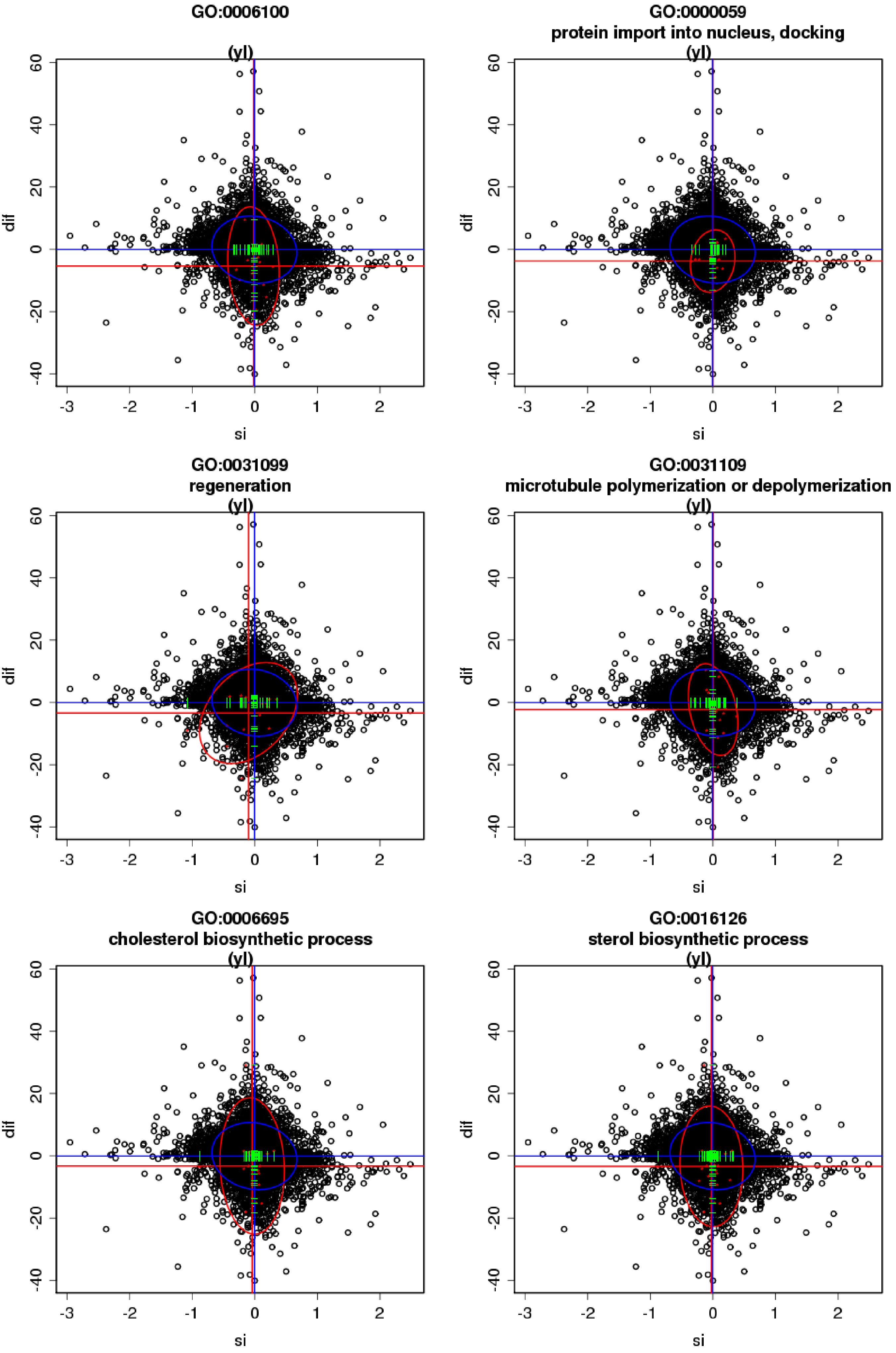
GO:0006100
(yl)
dif
si
GO:0000059
protein import into nucleus, docking
(yl)
dif
si
GO:0031099
regeneration
(yl)
dif
si
GO:0031109
microtubule polymerization or depolymerization
(yl)
dif
si
GO:0006695
cholesterol biosynthetic process
(yl)
dif
si
GO:0016126
sterol biosynthetic process
(yl)
dif
si
60
40
20
0
-20
-40
-3
-2
-1
0
1
2

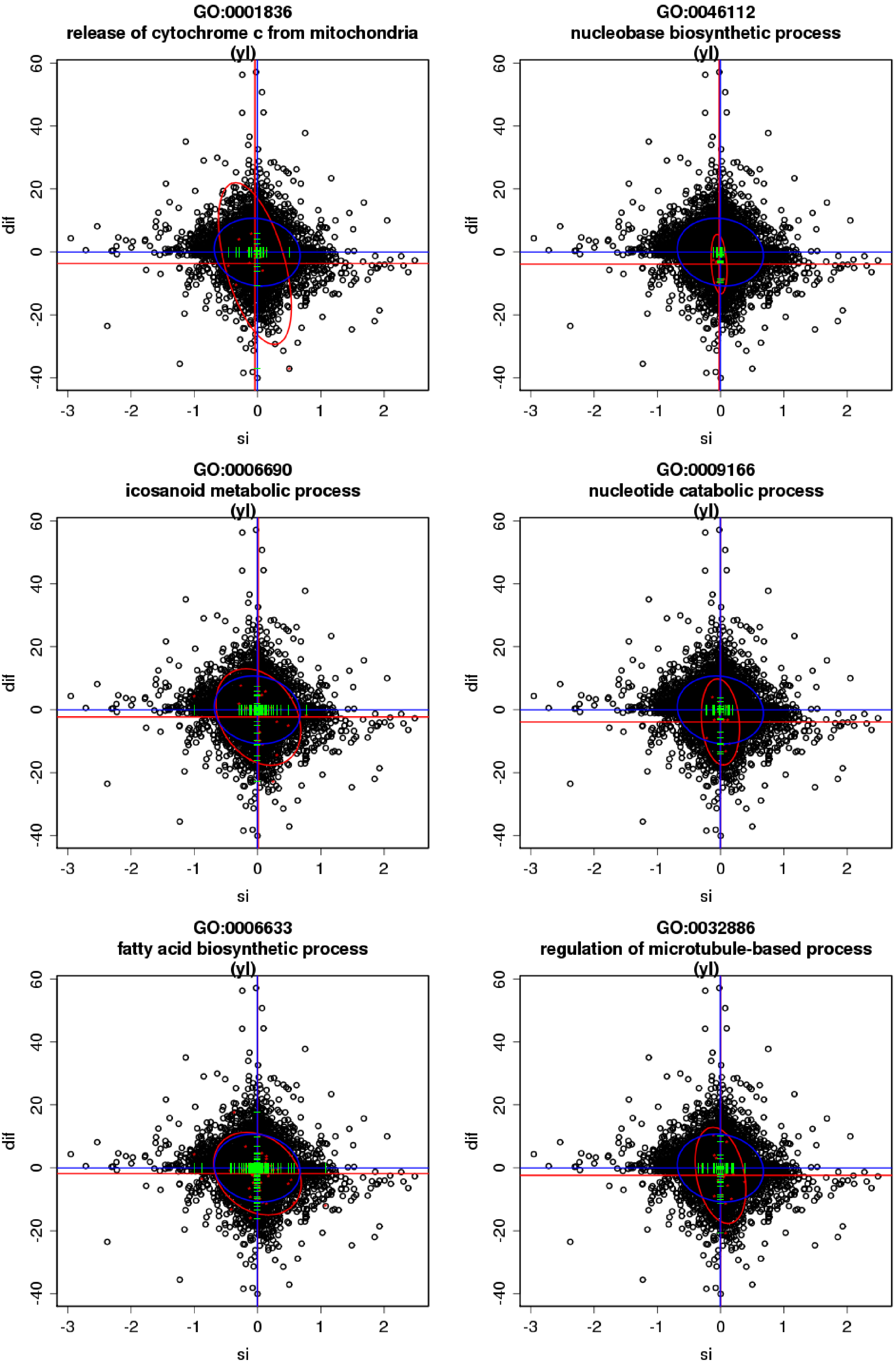
GO:0001836
release of cytochrome c from mitochondria
(yl)
GO:0046112
nucleobase biosynthetic process
(yl)
GO:0006690
icosanoid metabolic process
(yl)
GO:0009166
nucleotide catabolic process
(yl)
GO:0006633
fatty acid biosynthetic process
(yl)
GO:0032886
regulation of microtubule-based process
(yl)
dif
si
60
40
20
0
-20
-40
-3
-2
-1
0
1
2

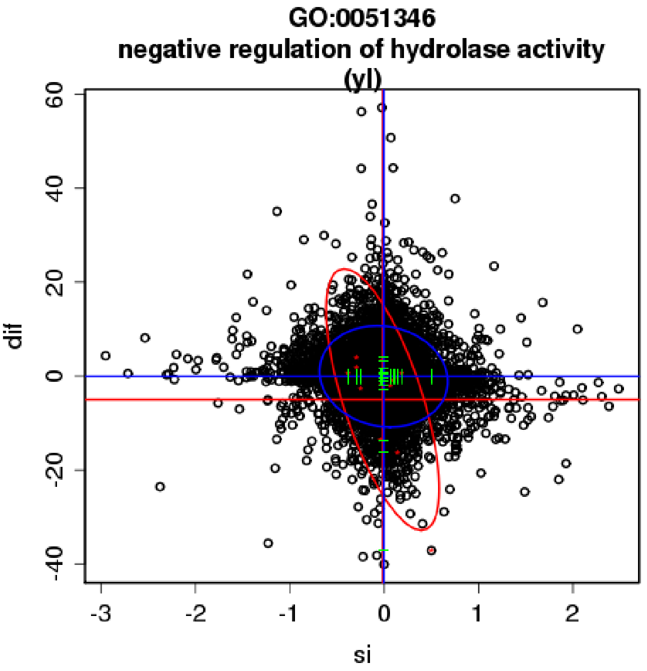
GO:0051346
negative regulation of hydrolase activity
(yl)
dif
si

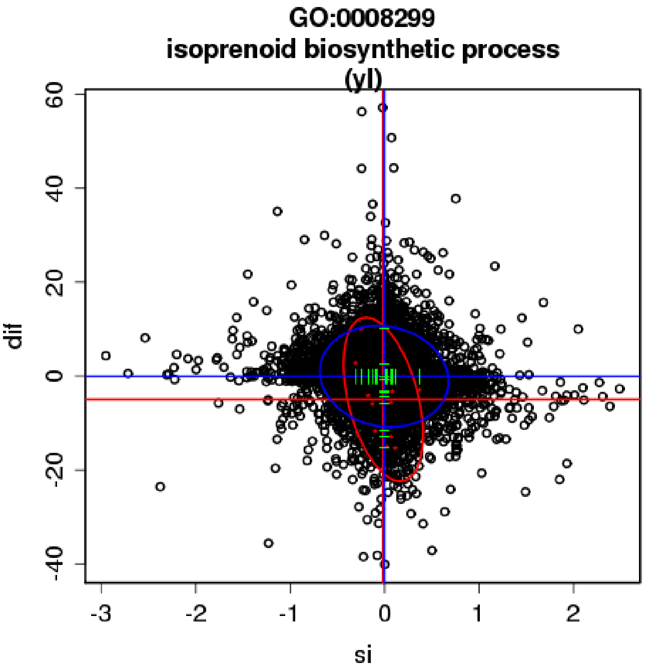
GO:0008299
isoprenoid biosynthetic process
(yl)
dif
si

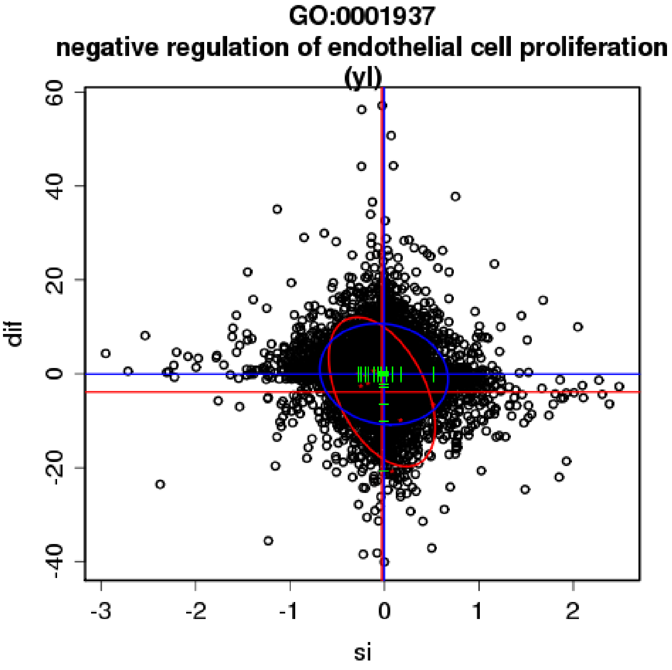
GO:0001937
negative regulation of endothelial cell proliferation
(yl)
dif
si

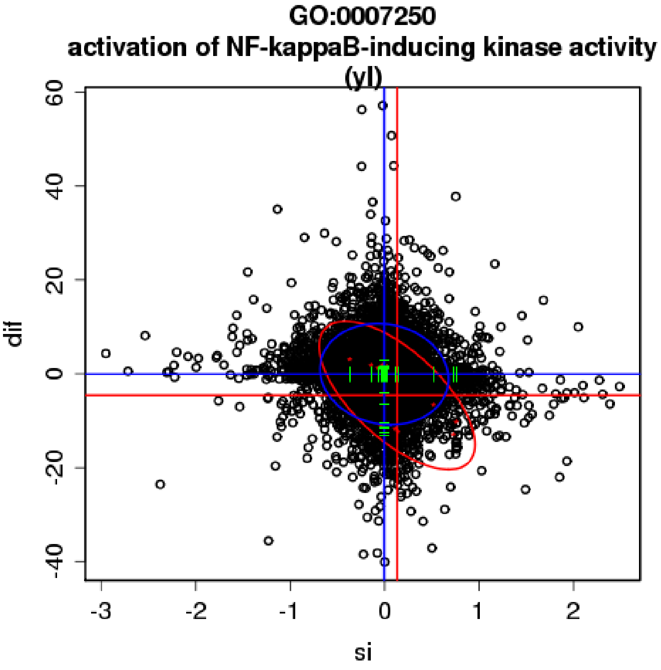
GO:0007250
activation of NF-kappaB-inducing kinase activity
(yl)
dif
si

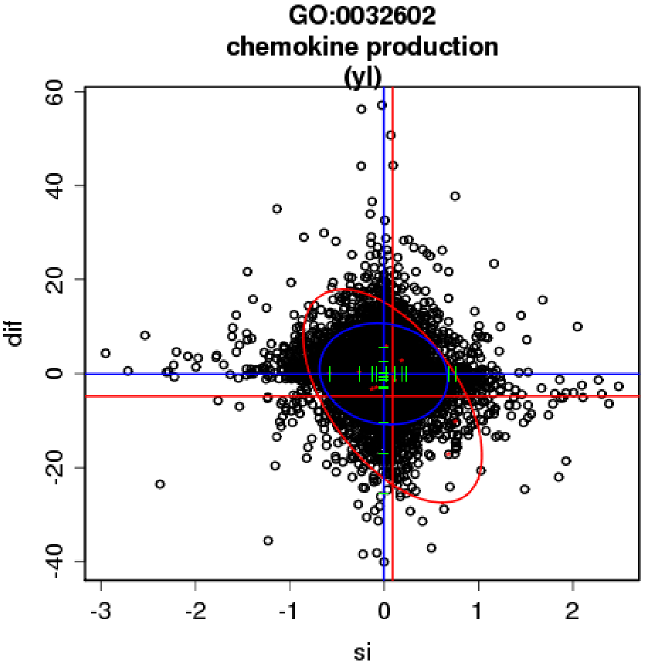
GO:0032602
chemokine production
(yl)
dif
si

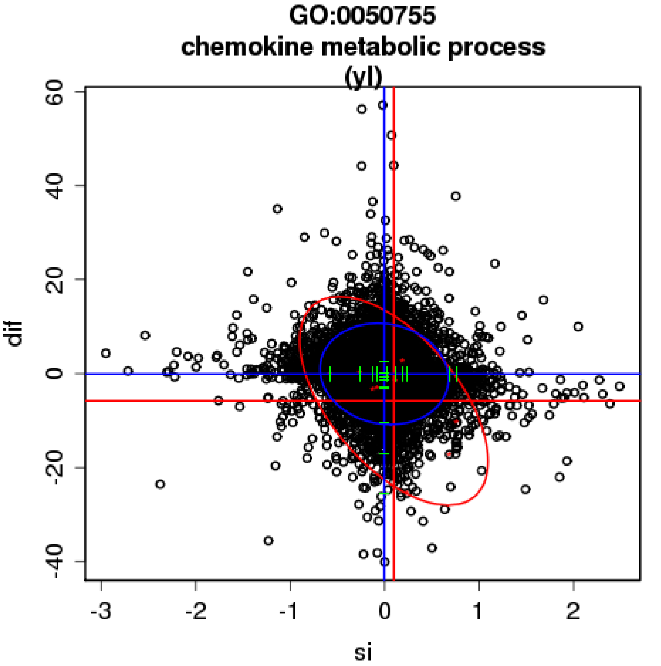
GO:0050755
chemokine metabolic process
(yl)
dif
si

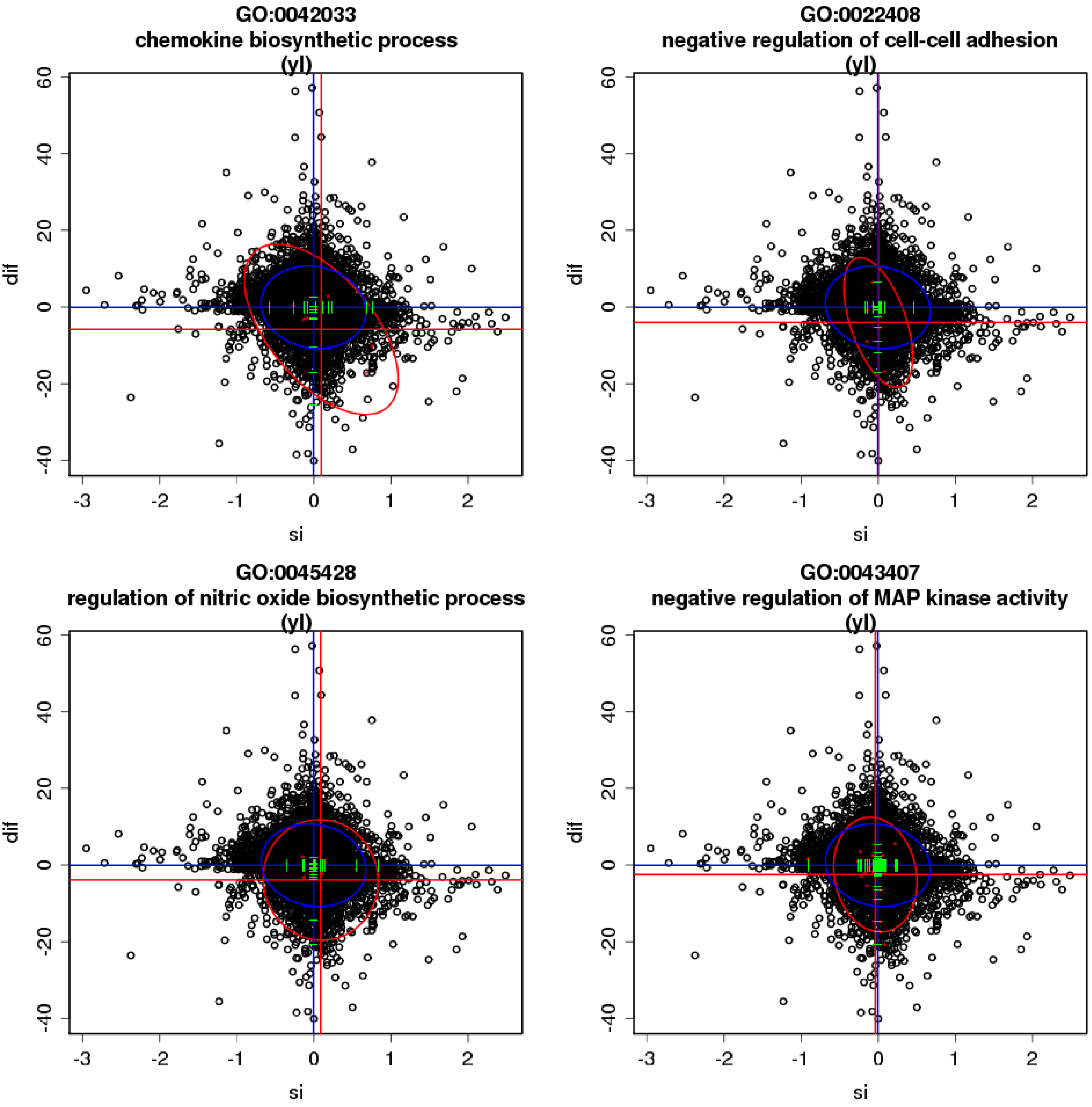
GO:0042033
chemokine biosynthetic process
(yl)
GO:0022408
negative regulation of cell-cell adhesion
(yl)
GO:0045428
regulation of nitric oxide biosynthetic process
(yl)
GO:0043407
negative regulation of MAP kinase activity
(yl)
dif
si

Combined functional profiling of alternative splicing and differential gene expression using KEGG pathways

18 significant modules

LOR indicates Log Odds Ratio of the coefficient or the interaction

p indicates FDR adjusted p-value

	LOR si	LOR dif	LOR Inter	p si	p dif	p Inter	pattern	name
hsa04670	-0.09	0.39	0.09	0.98	0.01	1.00	yh	Leukocyte transendothelial migration
hsa04530	-0.06	0.31	0.11	0.98	0.04	1.00	yh	Tight junction
hsa04660	-0.09	0.33	0.06	0.98	0.05	1.00	yh	T cell receptor signaling pathway
hsa04664	0.36	0.37	-0.02	0.59	0.05	1.00	yh	Fc epsilon RI signaling pathway
hsa00562	0.12	0.38	-0.02	0.98	0.04	1.00	yh	Inositol phosphate metabolism
hsa04210	-0.09	-0.48	-0.04	0.98	0.00	1.00	yl	Apoptosis
hsa04512	-0.18	-0.49	-0.03	0.98	0.01	1.00	yl	ECM-receptor interaction
hsa04060	0.33	-0.31	0.02	0.06	0.01	1.00	yl	Cytokine-cytokine receptor interaction
hsa04360	-0.36	-0.40	-0.05	0.45	0.04	1.00	yl	Axon guidance
hsa01040	-0.31	-0.60	-0.02	0.98	0.04	1.00	yl	Biosynthesis of unsaturated fatty acids
hsa00220	0.03	-0.66	-0.00	0.98	0.00	1.00	yl	Urea cycle and metabolism of amino groups
hsa04940	0.48	-0.50	0.05	0.45	0.03	1.00	yl	Type I diabetes mellitus
hsa00010	-0.02	-0.45	0.03	0.98	0.05	1.00	yl	Glycolysis / Gluconeogenesis
hsa00620	-0.41	-0.73	-0.04	0.98	0.00	1.00	yl	Pyruvate metabolism
hsa00640	0.01	-0.69	0.01	0.99	0.00	1.00	yl	Propanoate metabolism
hsa00903	-0.19	-0.68	-0.03	0.98	0.00	1.00	yl	Limonene and pinene degradation
hsa00100	0.08	-0.72	-0.00	0.98	0.00	1.00	yl	Biosynthesis of steroids
hsa00020	0.33	-0.63	0.05	0.98	0.00	1.00	yl	Citrate cycle (TCA cycle)

Table 1: LOR: log odds ratio; p: adjusted p-value FDR

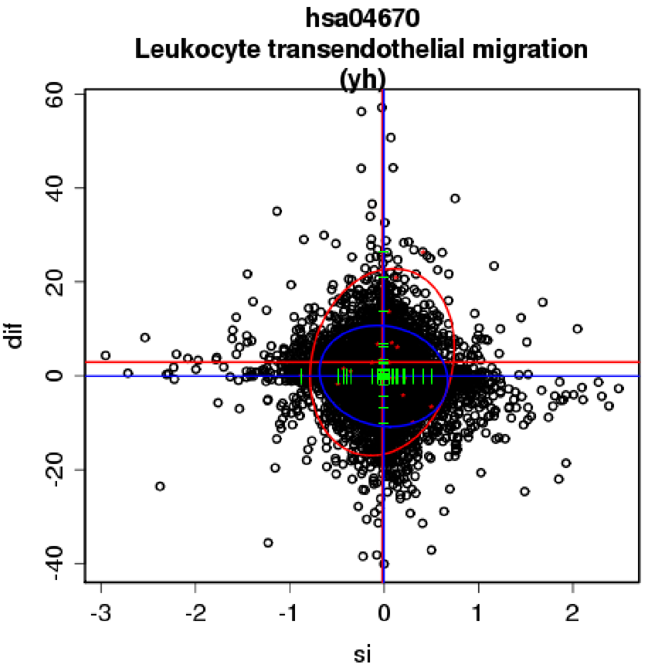

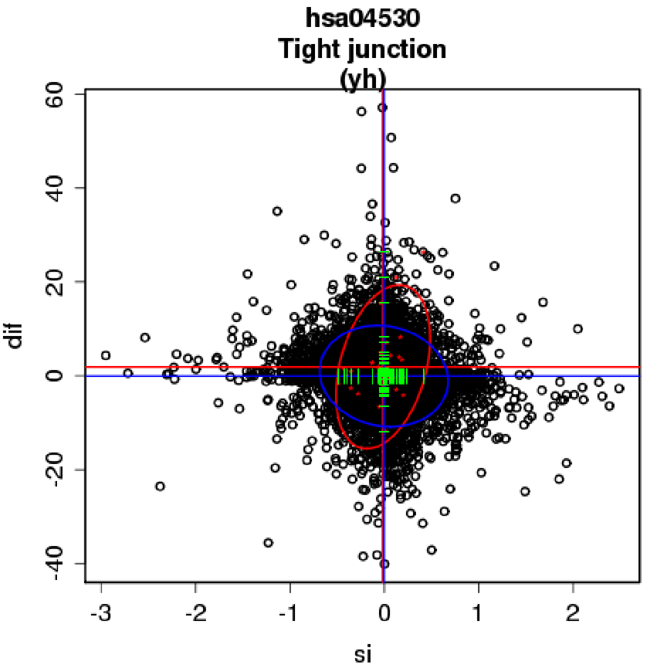

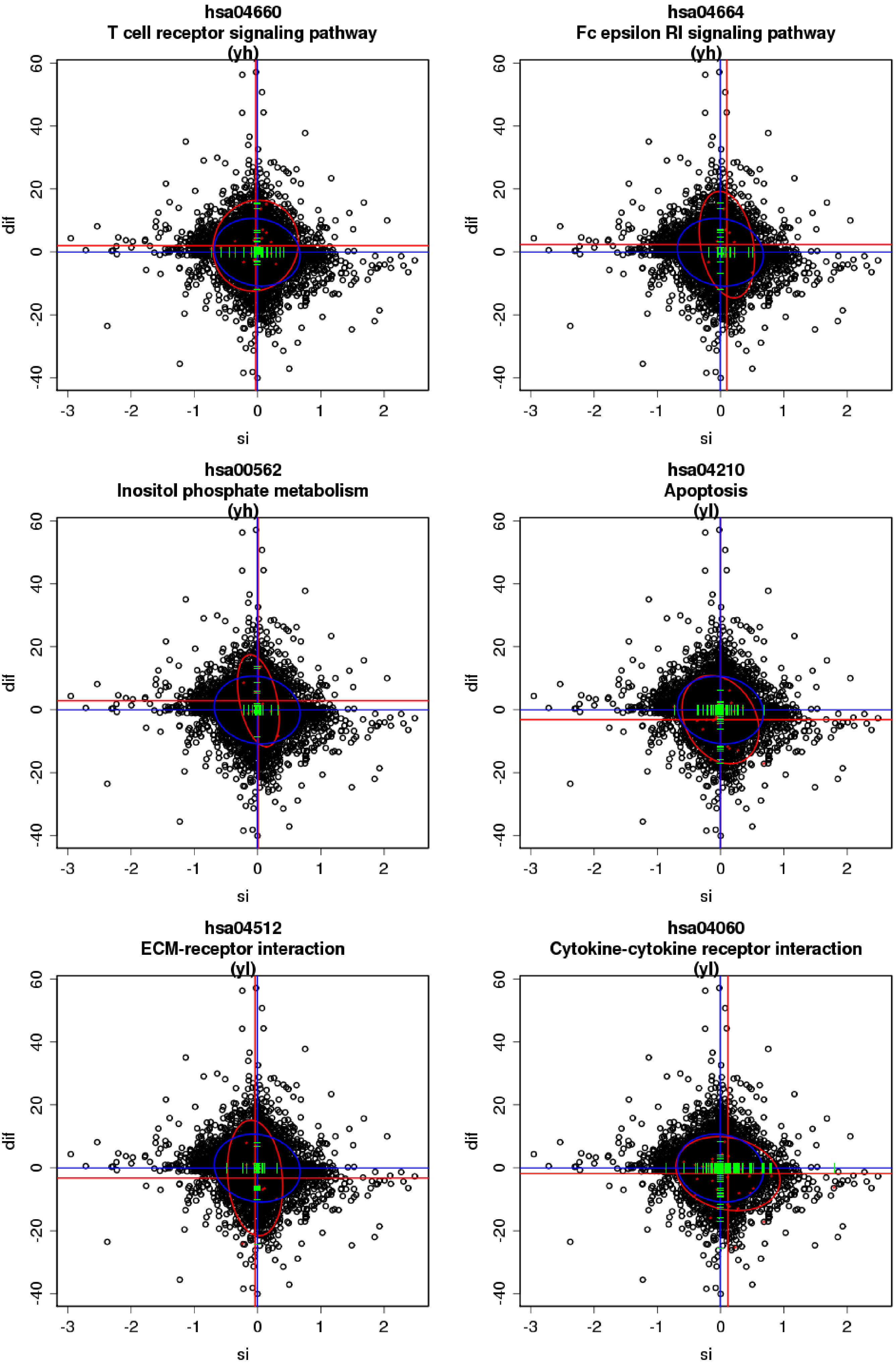
hsa04660
T cell receptor signaling pathway
(yh)
hsa04664
Fc epsilon RI signaling pathway
(yh)
hsa00562
Inositol phosphate metabolism
(yh)
hsa04210
Apoptosis
(yl)
hsa04512
ECM-receptor interaction
(yl)
hsa04060
Cytokine-cytokine receptor interaction
(yl)
dif
si
60
40
20
0
-20
-40
-3
-2
-1
0
1
2

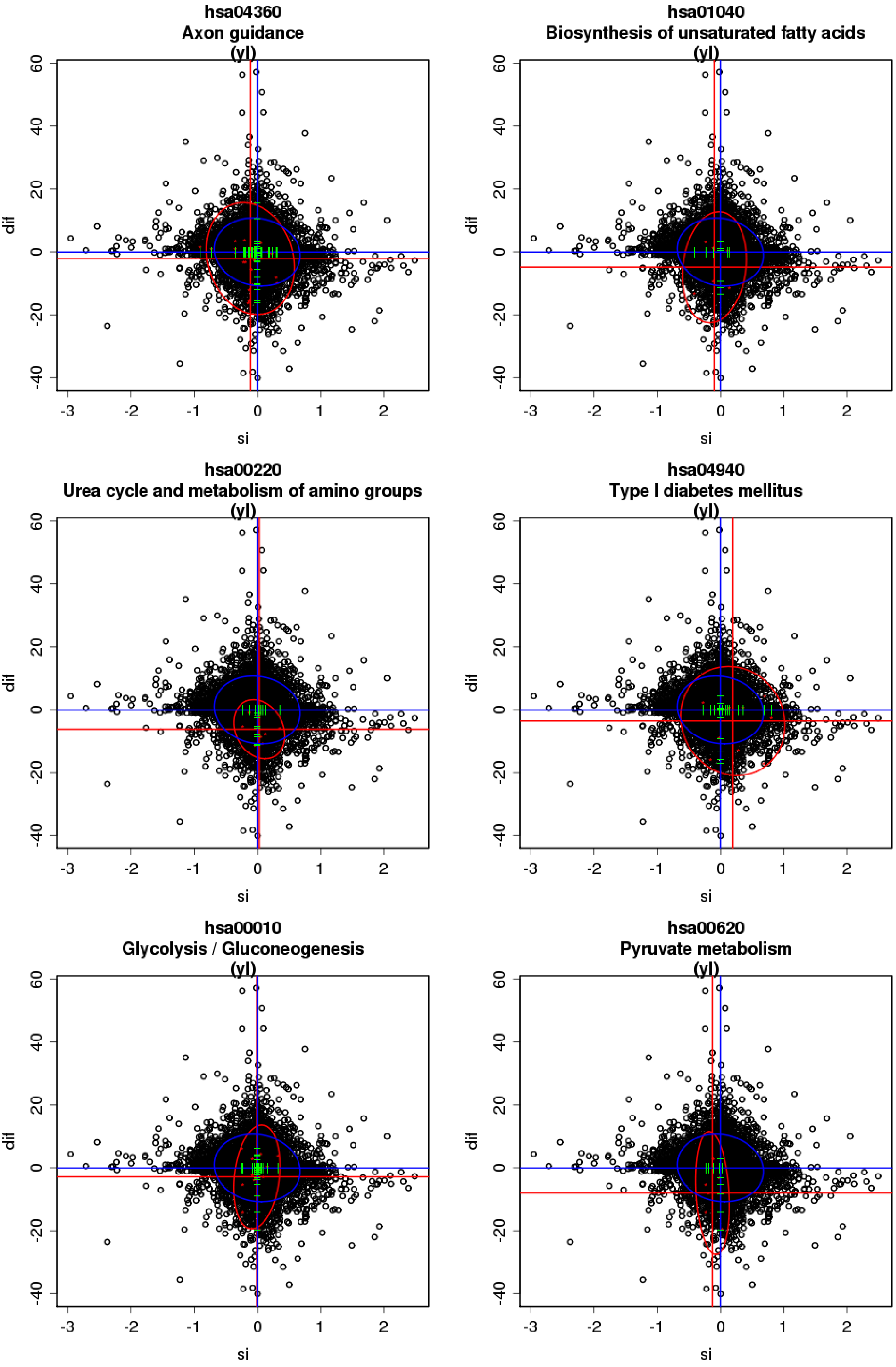
hsa04360
Axon guidance
(yl)
hsa01040
Biosynthesis of unsaturated fatty acids
(yl)
hsa00220
Urea cycle and metabolism of amino groups
(yl)
hsa04940
Type I diabetes mellitus
(yl)
hsa00010
Glycolysis / Gluconeogenesis
(yl)
hsa00620
Pyruvate metabolism
(yl)
dif
si
-40
-20
0
20
40
60
-3
-2
-1
0
1
2

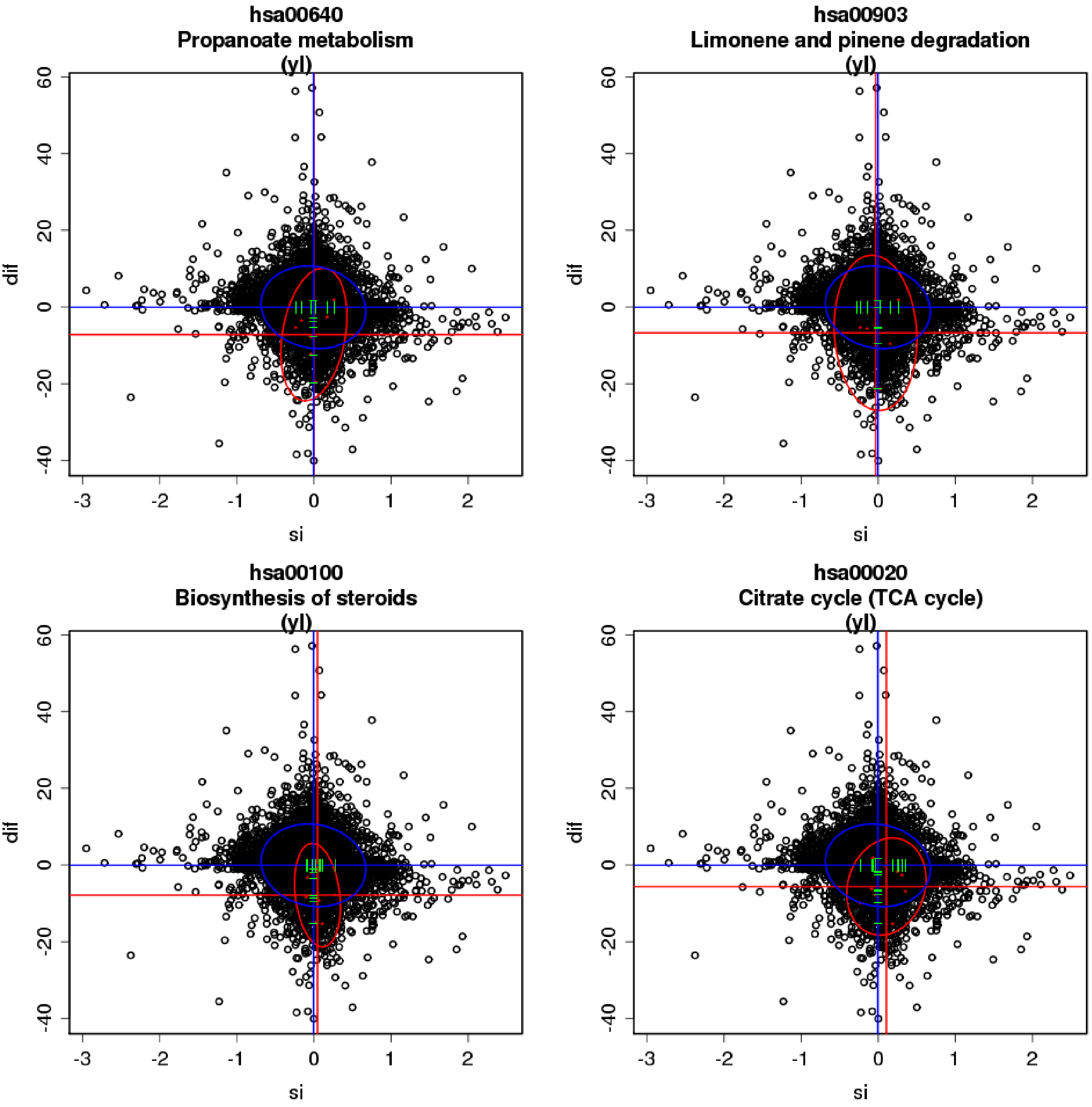
hsa00640
Propanoate metabolism
(yl)
hsa00903
Limonene and pinene degradation
(yl)
hsa00100
Biosynthesis of steroids
(yl)
hsa00020
Citrate cycle (TCA cycle)
(yl)
dif
si
60
40
20
0
-20
-40
-3
-2
-1
0
1
2
