# Supplementary material for: Multidimensional Gene Set Analysis of Genomic Data
Source: PLoS One. 2010 Apr 27;5(4):e10348. doi: 10.1371/journal.pone.0010348 (PMC2860497; doi:10.1371/journal.pone.0010348)

Combined functional profiling of dermatitis and psoriasis datasets using GO Biological Process

465 significant modules

LOR indicates Log Odds Ratio of the coefficient or the interaction

p indicates FDR adjusted p-value

	LOR t d	LOR t p	LOR Inter	p t d	p t p	p Inter	pattern	name
GO:0022403	-0.13	0.36	0.11	0.11	0.00	0.01	q1i	cell cycle phase
GO:0000279	-0.06	0.37	0.12	0.55	0.00	0.03	q1i	M phase
GO:0051301	-0.10	0.25	0.15	0.36	0.00	0.00	q1i	cell division
GO:0000087	-0.11	0.40	0.12	0.32	0.00	0.05	q1i	M phase of mitotic cell cycle
GO:0048015	0.08	0.07	0.16	0.72	0.68	0.05	b13	phosphoinositide-mediated signaling
GO:0000302	0.24	-0.06	0.29	0.59	0.85	0.00	b13	response to reactive oxygen species
GO:0032945	0.43	0.33	-0.79	0.26	0.39	0.00	b24	negative regulation of mononuclear cell proliferation
GO:0050672	0.43	0.33	-0.79	0.26	0.39	0.00	b24	negative regulation of lymphocyte proliferation
GO:0048589	-0.19	-0.06	-0.59	0.53	0.91	0.04	b24	developmental growth
GO:0007028	0.21	-0.11	-0.75	0.47	0.83	0.00	b24	cytoplasm organization and biogenesis
GO:0007043	0.07	-0.50	-0.91	0.86	0.22	0.00	b24	cell-cell junction assembly
GO:0045216	0.12	-0.26	-0.86	0.75	0.59	0.00	b24	cell-cell junction assembly and maintenance
GO:0009607	0.35	0.19	0.05	0.00	0.02	0.69	q1f	response to biotic stimulus
GO:0051704	0.31	0.16	0.05	0.00	0.03	0.68	q1f	multi-organism process
GO:0045893	-0.23	0.24	-0.05	0.00	0.00	0.78	q2f	positive regulation of transcription, DNA-dependent
GO:0045935	-0.19	0.23	-0.05	0.01	0.00	0.72	q2f	positive regulation of nucleobase, nucleoside, nucleotide and nucleic acid metabolic process
GO:0045941	-0.22	0.23	-0.05	0.00	0.00	0.78	q2f	positive regulation of transcription
GO:0051254	-0.23	0.24	-0.05	0.00	0.00	0.78	q2f	positive regulation of RNA metabolic process
GO:0016481	-0.36	0.18	0.05	0.00	0.02	0.61	q2f	negative regulation of transcription
GO:0045892	-0.31	0.21	0.01	0.00	0.02	0.99	q2f	negative regulation of transcription, DNA-dependent
GO:0045934	-0.36	0.19	0.04	0.00	0.01	0.75	q2f	negative regulation of nucleobase, nucleoside, nucleotide and nucleic acid metabolic process
GO:0051253	-0.33	0.22	0.00	0.00	0.01	0.99	q2f	negative regulation of RNA metabolic process
GO:0009719	-0.20	0.30	0.04	0.00	0.00	0.73	q2f	response to endogenous stimulus
GO:0006281	-0.23	0.19	-0.01	0.01	0.03	0.99	q2f	DNA repair
GO:0006974	-0.22	0.29	0.05	0.00	0.00	0.68	q2f	response to DNA damage stimulus
GO:0006512	-0.42	0.16	-0.01	0.00	0.03	0.95	q2f	
GO:0016567	-0.41	0.36	-0.04	0.00	0.01	0.93	q2f	protein ubiquitination
GO:0032446	-0.38	0.29	-0.03	0.00	0.05	0.94	q2f	protein modification by small protein conjugation
GO:0016125	-0.34	0.31	-0.05	0.02	0.03	0.87	q2f	sterol metabolic process
GO:0006913	-0.28	0.43	-0.07	0.00	0.00	0.71	q2f	nucleocytoplasmic transport
GO:0051169	-0.28	0.43	-0.07	0.00	0.00	0.71	q2f	nuclear transport
GO:0006605	-0.23	0.35	-0.01	0.01	0.00	0.97	q2f	protein targeting
GO:0006606	-0.27	0.39	-0.03	0.05	0.00	0.94	q2f	protein import into nucleus
GO:0006886	-0.18	0.27	0.00	0.00	0.00	0.99	q2f	intracellular protein transport
GO:0043285	-0.18	0.18	0.02	0.02	0.03	0.93	q2f	biopolymer catabolic process
GO:0008544	-0.29	0.40	0.12	0.01	0.00	0.13	q2f	epidermis development
GO:0030163	-0.23	0.20	0.02	0.01	0.04	0.94	q2f	protein catabolic process
GO:0016071	-0.32	0.27	-0.09	0.00	0.00	0.48	q2f	mRNA metabolic process
GO:0008380	-0.37	0.34	-0.11	0.00	0.00	0.47	q2f	RNA splicing
GO:0006397	-0.34	0.32	-0.11	0.00	0.00	0.39	q2f	mRNA processing
GO:0006403	-0.34	0.35	-0.06	0.03	0.02	0.85	q2f	RNA localization
GO:0015931	-0.39	0.40	-0.04	0.01	0.00	0.93	q2f	nucleobase, nucleoside, nucleotide and nucleic acid transport
GO:0050657	-0.36	0.38	-0.05	0.02	0.01	0.90	q2f	nucleic acid transport
GO:0051236	-0.36	0.38	-0.05	0.02	0.01	0.90	q2f	establishment of RNA localization
GO:0050658	-0.36	0.38	-0.05	0.02	0.01	0.90	q2f	RNA transport
GO:0051028	-0.39	0.34	-0.04	0.02	0.04	0.94	q2f	mRNA transport
GO:0022613	-0.31	0.39	0.00	0.00	0.00	0.99	q2f	ribonucleoprotein complex biogenesis and assembly
GO:0006413	-0.50	0.39	0.05	0.00	0.00	0.81	q2f	translational initiation
GO:0022618	-0.48	0.40	-0.02	0.00	0.00	0.95	q2f	ribonucleoprotein complex assembly
GO:0000082	-0.51	0.47	0.07	0.02	0.02	0.82	q2f	G1/S transition of mitotic cell cycle
GO:0006888	-0.46	0.41	0.03	0.00	0.00	0.92	q2f	ER to Golgi vesicle-mediated transport
GO:0040029	-0.44	0.39	0.01	0.01	0.02	0.99	q2f	regulation of gene expression, epigenetic
GO:0000075	-0.40	0.63	0.01	0.00	0.00	0.98	q2f	cell cycle checkpoint
GO:0007093	-0.43	0.57	0.05	0.02	0.00	0.87	q2f	mitotic cell cycle checkpoint
GO:0006470	-0.24	0.25	-0.10	0.03	0.03	0.65	q2f	protein amino acid dephosphorylation
GO:0007567	-0.80	0.64	0.04	0.01	0.02	0.95	q2f	parturition
GO:0044257	-0.39	0.30	0.05	0.00	0.00	0.80	q2f	cellular protein catabolic process
GO:0006511	-0.40	0.29	0.05	0.00	0.00	0.80	q2f	ubiquitin-dependent protein catabolic process
GO:0019941	-0.39	0.30	0.05	0.00	0.00	0.78	q2f	modification-dependent protein catabolic process
GO:0043632	-0.39	0.30	0.05	0.00	0.00	0.78	q2f	modification-dependent macromolecule catabolic process

GO:0051603	-0.39	0.30	0.05	0.00	0.00	0.79	q2f	proteolysis involved in cellular protein catabolic process
GO:0000375	-0.44	0.36	-0.14	0.00	0.01	0.61	q2f	RNA splicing, via transesterification reactions
GO:0000377	-0.44	0.36	-0.14	0.00	0.01	0.61	q2f	RNA splicing, via transesterification reactions with bulged adenosine as nucleophile
GO:0000398	-0.44	0.36	-0.14	0.00	0.01	0.61	q2f	nuclear mRNA splicing, via spliceosome
GO:0045646	-0.66	0.81	-0.21	0.05	0.00	0.71	q2f	regulation of erythrocyte differentiation
GO:0030216	-0.48	0.61	0.15	0.02	0.00	0.38	q2f	keratinocyte differentiation
GO:0016573	-0.83	-0.71	-0.60	0.00	0.04	0.08	q3f	histone acetylation
GO:0006892	-0.63	-0.80	-0.56	0.02	0.01	0.13	q3f	post-Golgi vesicle-mediated transport
GO:0015698	0.50	-0.28	-0.27	0.00	0.05	0.22	q4f	inorganic anion transport
GO:0016477	0.26	-0.20	0.08	0.00	0.02	0.52	q4f	cell migration
GO:0051239	0.22	-0.22	-0.03	0.01	0.00	0.87	q4f	regulation of multicellular organismal process
GO:0030182	0.20	-0.34	0.04	0.04	0.00	0.81	q4f	neuron differentiation
GO:0016051	0.33	-0.42	-0.05	0.03	0.00	0.90	q4f	carbohydrate biosynthetic process
GO:0030029	0.25	-0.26	0.09	0.01	0.00	0.47	q4f	actin filament-based process
GO:0030036	0.23	-0.28	0.08	0.02	0.00	0.54	q4f	actin cytoskeleton organization and biogenesis
GO:0016337	0.23	-0.22	0.03	0.02	0.01	0.87	q4f	cell-cell adhesion
GO:0031032	0.73	-0.88	0.10	0.02	0.00	0.84	q4f	actomyosin structure organization and biogenesis
GO:0048627	0.85	-0.80	0.11	0.00	0.00	0.80	q4f	myoblast development
GO:0048628	0.85	-0.80	0.11	0.00	0.00	0.80	q4f	myoblast maturation
GO:0007600	0.18	-0.23	-0.02	0.01	0.00	0.90	q4f	sensory perception
GO:0031589	0.36	-0.29	0.10	0.02	0.05	0.60	q4f	cell-substrate adhesion
GO:0050953	0.23	-0.34	-0.02	0.05	0.00	0.97	q4f	sensory perception of light stimulus
GO:0007601	0.23	-0.34	-0.02	0.05	0.00	0.97	q4f	visual perception
GO:0043062	0.60	-0.62	0.01	0.00	0.00	0.99	q4f	extracellular structure organization and biogenesis
GO:0030198	0.67	-0.69	0.05	0.00	0.00	0.91	q4f	extracellular matrix organization and biogenesis
GO:0051046	0.49	-0.42	0.04	0.03	0.04	0.94	q4f	regulation of secretion
GO:0006817	0.87	-0.61	-0.10	0.00	0.00	0.81	q4f	phosphate transport
GO:0032963	0.94	-0.72	0.07	0.00	0.01	0.93	q4f	collagen metabolic process
GO:0006957	1.12	-0.88	0.17	0.02	0.03	0.79	q4f	complement activation, alternative pathway
GO:0006812	0.16	0.05	-0.09	0.02	0.59	0.47	xh	cation transport
GO:0030001	0.18	-0.02	-0.07	0.02	0.91	0.64	xh	metal ion transport
GO:0019932	0.21	0.04	0.06	0.02	0.77	0.64	xh	second-messenger-mediated signaling
GO:0006820	0.47	-0.16	-0.26	0.00	0.28	0.15	xh	anion transport
GO:0033238	0.44	-0.12	0.14	0.05	0.62	0.54	xh	regulation of amine metabolic process
GO:0001932	0.51	-0.15	0.15	0.02	0.55	0.48	xh	regulation of protein amino acid phosphorylation
GO:0031401	0.58	0.11	0.02	0.04	0.80	0.99	xh	positive regulation of protein modification process
GO:0006521	0.50	-0.15	0.15	0.03	0.56	0.51	xh	regulation of amino acid metabolic process
GO:0033240	0.76	-0.05	0.08	0.01	0.91	0.90	xh	positive regulation of amine metabolic process
GO:0001934	0.77	-0.09	0.09	0.01	0.85	0.86	xh	positive regulation of protein amino acid phosphorylation
GO:0050730	0.53	-0.14	0.15	0.04	0.62	0.60	xh	regulation of peptidyl-tyrosine phosphorylation
GO:0045764	0.77	-0.09	0.09	0.01	0.85	0.86	xh	positive regulation of amino acid metabolic process
GO:0050731	0.91	-0.07	0.07	0.01	0.89	0.93	xh	positive regulation of peptidyl-tyrosine phosphorylation
GO:0002682	0.27	-0.04	-0.11	0.02	0.85	0.66	xh	regulation of immune system process
GO:0002684	0.32	-0.08	-0.05	0.01	0.62	0.90	xh	positive regulation of immune system process
GO:0048583	0.27	-0.01	-0.12	0.03	0.99	0.66	xh	regulation of response to stimulus
GO:0048584	0.37	-0.06	-0.07	0.01	0.79	0.82	xh	positive regulation of response to stimulus
GO:0051240	0.30	-0.13	-0.01	0.01	0.37	0.99	xh	positive regulation of multicellular organismal process
GO:0002694	0.33	0.13	-0.15	0.03	0.51	0.66	xh	regulation of leukocyte activation
GO:0050865	0.33	0.13	-0.15	0.03	0.51	0.66	xh	regulation of cell activation
GO:0051241	0.36	-0.26	-0.09	0.04	0.17	0.82	xh	negative regulation of multicellular organismal process
GO:0051249	0.38	0.15	-0.16	0.01	0.46	0.63	xh	regulation of lymphocyte activation
GO:0050878	0.31	-0.18	0.03	0.02	0.19	0.94	xh	regulation of body fluid levels
GO:0007599	0.32	-0.16	0.02	0.03	0.30	0.95	xh	hemostasis
GO:0009611	0.39	-0.04	0.09	0.00	0.67	0.17	xh	response to wounding
GO:0050817	0.34	-0.17	0.03	0.02	0.30	0.93	xh	coagulation
GO:0006959	0.63	0.03	0.02	0.00	0.86	0.94	xh	humoral immune response
GO:0051707	0.50	0.20	0.03	0.00	0.05	0.90	xh	response to other organism
GO:0007596	0.32	-0.14	0.03	0.03	0.42	0.93	xh	blood coagulation
GO:0019730	0.77	0.16	-0.11	0.00	0.47	0.78	xh	antimicrobial humoral response
GO:0007610	0.28	-0.03	0.08	0.00	0.73	0.32	xh	behavior
GO:0007626	0.33	0.00	0.08	0.00	0.99	0.54	xh	locomotory behavior
GO:0042330	0.46	0.18	0.01	0.00	0.24	0.99	xh	taxis
GO:0002250	0.40	-0.06	-0.03	0.01	0.81	0.95	xh	adaptive immune response
GO:0002252	0.32	0.03	-0.01	0.02	0.87	0.99	xh	immune effector process
GO:0002443	0.39	0.12	-0.08	0.01	0.55	0.82	xh	leukocyte mediated immunity
GO:0002449	0.38	0.07	-0.07	0.02	0.76	0.84	xh	lymphocyte mediated immunity
GO:0002460	0.40	-0.06	-0.03	0.01	0.81	0.95	xh	adaptive immune response based on somatic recombination of immune receptors built from immunoglobulin superfamily domains
GO:0016064	0.53	-0.25	-0.08	0.00	0.27	0.89	xh	immunoglobulin mediated immune response
GO:0019724	0.53	-0.25	-0.08	0.00	0.27	0.89	xh	B cell mediated immunity
GO:0002696	0.46	-0.05	0.04	0.01	0.84	0.94	xh	positive regulation of leukocyte activation
GO:0045191	0.98	-0.22	-0.31	0.00	0.75	0.78	xh	regulation of isotype switching
GO:0045830	1.16	0.18	-0.56	0.00	0.82	0.56	xh	positive regulation of isotype switching
GO:0045911	1.16	0.18	-0.56	0.00	0.82	0.56	xh	positive regulation of DNA recombination
GO:0048291	1.33	0.44	-0.61	0.00	0.62	0.56	xh	isotype switching to IgG isotypes
GO:0048302	1.33	0.44	-0.61	0.00	0.62	0.56	xh	regulation of isotype switching to IgG isotypes
GO:0050867	0.46	-0.05	0.04	0.01	0.84	0.94	xh	positive regulation of cell activation
GO:0051054	0.73	0.50	-0.54	0.02	0.12	0.23	xh	positive regulation of DNA metabolic process
GO:0051251	0.45	-0.02	0.01	0.01	0.92	0.99	xh	positive regulation of lymphocyte activation
GO:0006968	0.47	0.16	-0.10	0.00	0.42	0.78	xh	cellular defense response
GO:0006935	0.46	0.18	0.01	0.00	0.24	0.99	xh	chemotaxis
GO:0006954	0.49	0.00	0.09	0.00	0.99	0.41	xh	inflammatory response
GO:0005996	0.33	0.00	0.05	0.00	1.00	0.81	xh	monosaccharide metabolic process
GO:0044262	0.22	-0.07	0.03	0.00	0.47	0.84	xh	cellular carbohydrate metabolic process
GO:0015980	0.21	-0.07	0.03	0.00	0.43	0.82	xh	energy derivation by oxidation of organic compounds
GO:0019318	0.33	-0.01	0.05	0.00	0.96	0.80	xh	hexose metabolic process
GO:0006006	0.34	-0.02	0.04	0.01	0.91	0.90	xh	glucose metabolic process
GO:0030003	0.22	-0.05	0.02	0.05	0.71	0.93	xh	cellular cation homeostasis
GO:0055065	0.27	-0.08	0.03	0.01	0.53	0.91	xh	metal ion homeostasis
GO:0055080	0.22	-0.09	0.02	0.04	0.47	0.94	xh	cation homeostasis
GO:0006875	0.27	-0.08	0.03	0.01	0.53	0.91	xh	cellular metal ion homeostasis

GO:0030005	0.28	-0.08	0.02	0.01	0.59	0.94	xh	cellular di-, tri-valent inorganic cation homeostasis
GO:0055066	0.28	-0.08	0.02	0.01	0.59	0.94	xh	di-, tri-valent inorganic cation homeostasis
GO:0055074	0.29	-0.09	0.03	0.02	0.57	0.90	xh	calcium ion homeostasis
GO:0006874	0.29	-0.09	0.03	0.02	0.57	0.90	xh	cellular calcium ion homeostasis
GO:0045214	1.16	-0.69	-0.11	0.02	0.26	0.95	xh	sarcomere organization
GO:0048646	0.28	-0.00	0.00	0.01	0.97	0.99	xh	anatomical structure formation
GO:0007160	0.35	-0.28	0.11	0.03	0.05	0.56	xh	cell-matrix adhesion
GO:0051480	0.44	-0.08	0.08	0.01	0.73	0.80	xh	cytosolic calcium ion homeostasis
GO:0007204	0.45	-0.04	0.07	0.01	0.85	0.83	xh	elevation of cytosolic calcium ion concentration
GO:0032103	0.73	-0.47	0.08	0.03	0.17	0.90	xh	positive regulation of response to external stimulus
GO:0050920	1.14	0.12	-0.13	0.04	0.91	0.94	xh	regulation of chemotaxis
GO:0006897	0.22	-0.04	-0.04	0.04	0.78	0.87	xh	endocytosis
GO:0010324	0.22	-0.04	-0.04	0.04	0.78	0.87	xh	membrane invagination
GO:0019935	0.32	-0.05	-0.16	0.01	0.83	0.53	xh	cyclic-nucleotide-mediated signaling
GO:0007187	0.38	-0.04	-0.20	0.00	0.85	0.47	xh	G-protein signaling, coupled to cyclic nucleotide second messenger
GO:0007188	0.34	-0.17	-0.18	0.03	0.37	0.60	xh	G-protein signaling, coupled to cAMP nucleotide second messenger
GO:0002526	0.48	-0.32	-0.04	0.01	0.09	0.94	xh	acute inflammatory response
GO:0015672	0.22	0.08	-0.15	0.01	0.48	0.32	xh	monovalent inorganic cation transport
GO:0006813	0.34	-0.08	-0.09	0.01	0.66	0.78	xh	potassium ion transport
GO:0051346	0.83	-0.11	0.23	0.03	0.82	0.47	xh	negative regulation of hydrolase activity
GO:0019882	0.78	0.01	-0.08	0.00	0.97	0.83	xh	antigen processing and presentation
GO:0048002	0.66	0.03	-0.08	0.00	0.91	0.90	xh	antigen processing and presentation of peptide antigen
GO:0002474	0.58	0.03	-0.05	0.01	0.91	0.94	xh	antigen processing and presentation of peptide antigen via MHC class I
GO:0048514	0.23	0.02	0.00	0.04	0.91	1.00	xh	blood vessel morphogenesis
GO:0001654	0.59	0.18	-0.18	0.02	0.60	0.78	xh	eye development
GO:0046530	1.05	0.44	-0.45	0.00	0.42	0.60	xh	photoreceptor cell differentiation
GO:0048592	0.72	0.50	-0.32	0.02	0.16	0.64	xh	eye morphogenesis
GO:0001754	1.08	0.47	-0.46	0.00	0.40	0.59	xh	eye photoreceptor cell differentiation
GO:0042461	1.05	0.44	-0.45	0.00	0.42	0.60	xh	photoreceptor cell development
GO:0042462	1.08	0.47	-0.46	0.00	0.40	0.59	xh	eye photoreceptor cell development
GO:0007586	0.44	0.02	0.11	0.00	0.92	0.53	xh	digestion
GO:0002274	0.88	0.52	-0.32	0.00	0.17	0.63	xh	myeloid leukocyte activation
GO:0001525	0.33	0.03	-0.00	0.00	0.85	0.99	xh	angiogenesis
GO:0050792	0.93	-0.14	0.01	0.02	0.84	0.99	xh	regulation of viral reproduction
GO:0045069	1.46	0.55	-0.40	0.00	0.51	0.75	xh	regulation of viral genome replication
GO:0007159	0.97	0.26	-0.17	0.04	0.73	0.90	xh	leukocyte adhesion
GO:0007416	0.65	-0.20	-0.07	0.03	0.64	0.94	xh	synaptogenesis
GO:0045576	1.39	0.05	-0.11	0.00	0.96	0.94	xh	mast cell activation
GO:0006007	0.54	0.12	-0.02	0.00	0.66	0.99	xh	glucose catabolic process
GO:0016052	0.43	0.06	0.05	0.00	0.78	0.82	xh	carbohydrate catabolic process
GO:0019320	0.53	0.13	0.01	0.00	0.55	0.99	xh	hexose catabolic process
GO:0044275	0.43	0.06	0.05	0.00	0.78	0.82	xh	cellular carbohydrate catabolic process
GO:0046164	0.48	0.02	0.09	0.00	0.94	0.69	xh	alcohol catabolic process
GO:0046365	0.53	0.13	0.01	0.00	0.55	0.99	xh	monosaccharide catabolic process
GO:0006096	0.64	-0.02	0.05	0.00	0.95	0.90	xh	glycolysis
GO:0044259	0.83	-0.34	0.06	0.02	0.42	0.94	xh	multicellular organismal macromolecule metabolic process
GO:0044236	0.86	-0.48	0.11	0.00	0.12	0.81	xh	multicellular organismal metabolic process
GO:0044243	0.91	-0.39	0.04	0.01	0.32	0.97	xh	multicellular organismal catabolic process
GO:0044256	0.83	-0.34	0.06	0.02	0.42	0.94	xh	protein digestion
GO:0044266	0.83	-0.34	0.06	0.02	0.42	0.94	xh	multicellular organismal macromolecule catabolic process
GO:0044268	0.83	-0.34	0.06	0.02	0.42	0.94	xh	multicellular organismal protein metabolic process
GO:0044254	0.83	-0.34	0.06	0.02	0.42	0.94	xh	multicellular organismal protein catabolic process
GO:0030574	0.83	-0.34	0.06	0.02	0.42	0.94	xh	collagen catabolic process
GO:0009617	0.54	0.10	0.10	0.00	0.70	0.69	xh	response to bacterium
GO:0042742	0.53	0.05	0.14	0.01	0.85	0.49	xh	defense response to bacterium
GO:0043010	1.43	-0.40	0.11	0.00	0.62	0.93	xh	camera-type eye development
GO:0051606	0.64	-0.10	-0.18	0.00	0.78	0.73	xh	detection of stimulus
GO:0009581	0.92	-0.16	-0.22	0.00	0.73	0.78	xh	detection of external stimulus
GO:0000270	0.90	-0.02	-0.05	0.00	0.97	0.97	xh	peptidoglycan metabolic process
GO:0043542	1.13	-0.10	0.29	0.01	0.86	0.48	xh	endothelial cell migration
GO:0051208	1.53	0.85	-0.52	0.00	0.22	0.60	xh	sequestering of calcium ion
GO:0051238	1.24	-0.59	0.18	0.00	0.18	0.72	xh	sequestering of metal ion
GO:0009306	0.52	-0.18	-0.02	0.02	0.53	0.99	xh	protein secretion
GO:0009582	0.77	-0.24	-0.31	0.00	0.56	0.66	xh	detection of abiotic stimulus
GO:0050906	0.81	-0.20	-0.40	0.00	0.69	0.58	xh	detection of stimulus involved in sensory perception
GO:0009583	0.92	-0.14	-0.26	0.00	0.83	0.80	xh	detection of light stimulus
GO:0009584	0.92	-0.14	-0.26	0.00	0.83	0.80	xh	detection of visible light
GO:0050962	0.93	-0.16	-0.25	0.00	0.81	0.80	xh	detection of light stimulus involved in sensory perception
GO:0007602	0.97	-0.19	-0.23	0.00	0.78	0.82	xh	phototransduction
GO:0050908	0.93	-0.16	-0.25	0.00	0.81	0.80	xh	detection of light stimulus involved in visual perception
GO:0002504	1.21	-0.18	-0.11	0.00	0.79	0.93	xh	antigen processing and presentation of peptide or polysaccharide antigen via MHC class II
GO:0045045	-0.24	0.13	-0.01	0.00	0.16	0.99	xl	
GO:0006325	-0.16	-0.05	-0.04	0.04	0.63	0.81	xl	establishment and/or maintenance of chromatin architecture
GO:0051276	-0.15	0.06	-0.00	0.03	0.48	0.99	xl	chromosome organization and biogenesis
GO:0016568	-0.35	-0.01	-0.09	0.00	0.98	0.65	xl	chromatin modification
GO:0007264	-0.14	0.08	0.07	0.05	0.34	0.40	xl	small GTPase mediated signal transduction
GO:0006631	-0.25	-0.12	-0.06	0.01	0.39	0.80	xl	fatty acid metabolic process
GO:0008202	-0.22	0.11	-0.01	0.03	0.37	0.97	xl	steroid metabolic process
GO:0030183	-0.41	0.37	0.01	0.04	0.06	0.99	xl	B cell differentiation
GO:0045786	-0.26	-0.04	0.05	0.00	0.73	0.78	xl	negative regulation of cell cycle
GO:0007179	-0.38	-0.37	-0.32	0.05	0.12	0.39	xl	transforming growth factor beta receptor signaling pathway
GO:0008610	-0.24	-0.06	-0.09	0.00	0.66	0.58	xl	lipid biosynthetic process
GO:0007050	-0.35	-0.16	0.02	0.01	0.39	0.98	xl	cell cycle arrest
GO:0048193	-0.41	0.20	0.01	0.00	0.10	0.99	xl	Golgi vesicle transport
GO:0006473	-0.76	-0.49	-0.44	0.00	0.15	0.26	xl	protein amino acid acetylation
GO:0016569	-0.54	-0.19	-0.37	0.00	0.55	0.23	xl	covalent chromatin modification
GO:0016570	-0.54	-0.19	-0.37	0.00	0.55	0.23	xl	histone modification
GO:0043543	-0.67	-0.44	-0.32	0.00	0.14	0.47	xl	protein amino acid acylation
GO:0032273	-0.81	0.66	-0.04	0.03	0.07	0.97	xl	positive regulation of protein polymerization
GO:0051495	-0.69	0.50	-0.00	0.02	0.13	0.99	xl	positive regulation of cytoskeleton organization and biogenesis
GO:0030518	-0.35	0.05	-0.02	0.03	0.84	0.98	xl	steroid hormone receptor signaling pathway
GO:0030522	-0.34	0.03	-0.02	0.03	0.89	0.99	xl	intracellular receptor-mediated signaling pathway
GO:0006694	-0.39	0.16	-0.11	0.00	0.42	0.77	xl	steroid biosynthetic process

GO:0045682	-0.78	0.07	0.03	0.02	0.91	0.99	xl	regulation of epidermis development
GO:0006505	-0.65	-0.39	-0.26	0.02	0.39	0.73	xl	GPI anchor metabolic process
GO:0006506	-0.65	-0.39	-0.26	0.02	0.39	0.73	xl	GPI anchor biosynthetic process
GO:0030384	-0.44	-0.13	-0.14	0.03	0.73	0.80	xl	phosphoinositide metabolic process
GO:0000245	-0.42	0.26	-0.22	0.03	0.31	0.63	xl	spliceosome assembly
GO:0006376	-0.66	0.34	-0.20	0.01	0.38	0.78	xl	mRNA splice site selection
GO:0006383	-0.45	0.18	-0.09	0.05	0.59	0.88	xl	transcription from RNA polymerase III promoter
GO:0009081	-0.82	0.47	-0.10	0.00	0.26	0.91	xl	branched chain family amino acid metabolic process
GO:0016126	-0.69	0.42	-0.17	0.00	0.08	0.72	xl	sterol biosynthetic process
GO:0006695	-0.70	0.08	-0.37	0.00	0.85	0.47	xl	cholesterol biosynthetic process
GO:0048515	-0.51	-0.02	-0.17	0.03	0.97	0.80	xl	spermatid differentiation
GO:0015669	-0.89	-0.63	-0.50	0.00	0.19	0.45	xl	gas transport
GO:0015671	-0.89	-0.63	-0.50	0.00	0.19	0.45	xl	oxygen transport
GO:0010212	-0.79	0.14	-0.32	0.03	0.85	0.78	xl	response to ionizing radiation
GO:0007031	-0.59	-0.13	-0.19	0.01	0.80	0.79	xl	peroxisome organization and biogenesis
GO:0001542	-0.79	0.43	-0.26	0.05	0.51	0.81	xl	ovulation from ovarian follicle
GO:0030728	-0.84	0.54	-0.30	0.03	0.35	0.78	xl	ovulation
GO:0006414	-0.69	0.37	-0.18	0.00	0.29	0.78	xl	translational elongation
GO:0006163	0.18	0.34	-0.22	0.35	0.04	0.48	yh	purine nucleotide metabolic process
GO:0006144	0.93	0.98	-0.50	0.10	0.00	0.47	yh	purine base metabolic process
GO:0009259	0.11	0.34	-0.17	0.58	0.03	0.59	yh	ribonucleotide metabolic process
GO:0045944	-0.19	0.25	-0.09	0.09	0.01	0.66	yh	positive regulation of transcription from RNA polymerase II promoter
GO:0006520	0.03	0.20	0.01	0.81	0.02	0.97	yh	amino acid metabolic process
GO:0032268	-0.10	0.21	0.10	0.29	0.00	0.06	yh	regulation of cellular protein metabolic process
GO:0032270	0.18	0.32	-0.09	0.31	0.04	0.78	yh	positive regulation of cellular protein metabolic process
GO:0051246	-0.10	0.21	0.09	0.26	0.00	0.13	yh	regulation of protein metabolic process
GO:0051247	0.16	0.30	-0.09	0.37	0.05	0.79	yh	positive regulation of protein metabolic process
GO:0009889	-0.19	0.21	0.01	0.06	0.03	0.97	yh	regulation of biosynthetic process
GO:0031326	-0.19	0.21	0.01	0.06	0.03	0.97	yh	regulation of cellular biosynthetic process
GO:0001775	0.16	0.25	-0.05	0.13	0.01	0.81	yh	cell activation
GO:0045321	0.15	0.26	-0.04	0.19	0.01	0.84	yh	leukocyte activation
GO:0046649	0.13	0.25	-0.03	0.28	0.01	0.93	yh	lymphocyte activation
GO:0050854	0.48	0.74	-0.34	0.38	0.04	0.67	yh	regulation of antigen receptor-mediated signaling pathway
GO:0050856	0.48	0.74	-0.34	0.38	0.04	0.67	yh	regulation of T cell receptor signaling pathway
GO:0051094	-0.06	0.21	0.03	0.49	0.00	0.79	yh	positive regulation of developmental process
GO:0043068	-0.07	0.27	0.04	0.52	0.00	0.80	yh	positive regulation of programmed cell death
GO:0006417	-0.21	0.29	0.02	0.05	0.00	0.93	yh	regulation of translation
GO:0042098	0.02	0.43	-0.07	0.95	0.05	0.90	yh	T cell proliferation
GO:0001816	0.13	0.32	-0.10	0.47	0.03	0.75	yh	cytokine production
GO:0042089	0.06	0.36	-0.13	0.82	0.04	0.75	yh	cytokine biosynthetic process
GO:0042107	0.07	0.35	-0.13	0.79	0.05	0.75	yh	cytokine metabolic process
GO:0043065	-0.07	0.27	0.04	0.51	0.00	0.80	yh	positive regulation of apoptosis
GO:0007243	0.09	0.16	0.01	0.28	0.03	0.95	yh	protein kinase cascade
GO:0051052	0.07	0.41	-0.16	0.76	0.01	0.60	yh	regulation of DNA metabolic process
GO:0043388	-0.21	0.77	-0.16	0.59	0.00	0.73	yh	positive regulation of DNA binding
GO:0051090	-0.28	0.49	-0.06	0.24	0.01	0.90	yh	regulation of transcription factor activity
GO:0051091	-0.23	0.84	-0.16	0.58	0.00	0.75	yh	positive regulation of transcription factor activity
GO:0051099	-0.25	0.73	-0.14	0.48	0.00	0.78	yh	positive regulation of binding
GO:0051101	-0.28	0.45	-0.08	0.18	0.01	0.82	yh	regulation of DNA binding
GO:0007249	0.15	0.36	-0.05	0.30	0.00	0.82	yh	I-kappaB kinase/NF-kappaB cascade
GO:0006282	-0.43	0.93	-0.21	0.47	0.00	0.78	yh	regulation of DNA repair
GO:0051092	0.01	0.86	-0.23	0.99	0.00	0.67	yh	positive regulation of NF-kappaB transcription factor activity
GO:0030099	-0.10	0.26	0.01	0.56	0.05	0.97	yh	myeloid cell differentiation
GO:0030218	-0.40	0.42	-0.02	0.06	0.04	0.97	yh	erythrocyte differentiation
GO:0034101	-0.40	0.42	-0.02	0.06	0.04	0.97	yh	erythrocyte homeostasis
GO:0048534	-0.07	0.18	0.03	0.59	0.05	0.84	yh	hemopoietic or lymphoid organ development
GO:0048872	-0.28	0.46	-0.06	0.17	0.01	0.88	yh	homeostasis of number of cells
GO:0043069	-0.04	0.19	0.05	0.76	0.02	0.72	yh	negative regulation of programmed cell death
GO:0007260	0.34	0.78	-0.15	0.48	0.01	0.80	yh	tyrosine phosphorylation of STAT protein
GO:0043066	-0.04	0.20	0.05	0.73	0.01	0.73	yh	negative regulation of apoptosis
GO:0017038	-0.25	0.41	-0.02	0.05	0.00	0.95	yh	protein import
GO:0051170	-0.25	0.39	-0.03	0.06	0.00	0.94	yh	nuclear import
GO:0009057	-0.03	0.15	0.04	0.74	0.03	0.75	yh	macromolecule catabolic process
GO:0022404	-0.38	0.65	-0.28	0.31	0.02	0.63	yh	molting cycle process
GO:0042303	-0.32	0.65	-0.27	0.42	0.01	0.63	yh	molting cycle
GO:0042633	-0.32	0.65	-0.27	0.42	0.01	0.63	yh	hair cycle
GO:0007005	0.10	0.32	0.02	0.65	0.03	0.97	yh	mitochondrion organization and biogenesis
GO:0008632	0.17	0.31	0.04	0.37	0.05	0.89	yh	apoptotic program
GO:0022405	-0.38	0.65	-0.28	0.31	0.02	0.63	yh	hair cycle process
GO:0001942	-0.38	0.65	-0.28	0.31	0.02	0.63	yh	hair follicle development
GO:0006916	-0.07	0.22	0.06	0.52	0.01	0.67	yh	anti-apoptosis
GO:0065002	-0.23	0.53	-0.01	0.44	0.01	0.99	yh	intracellular protein transport across a membrane
GO:0000060	-0.23	0.53	-0.01	0.44	0.01	0.99	yh	protein import into nucleus, translocation
GO:0009615	0.17	0.34	0.07	0.35	0.02	0.78	yh	response to virus
GO:0006405	-0.20	0.44	-0.12	0.47	0.04	0.80	yh	RNA export from nucleus
GO:0051168	-0.18	0.51	-0.13	0.40	0.00	0.72	yh	nuclear export
GO:0012502	-0.05	0.26	0.04	0.70	0.00	0.81	yh	induction of programmed cell death
GO:0051325	-0.23	0.38	0.10	0.13	0.00	0.50	yh	interphase
GO:0051329	-0.23	0.39	0.10	0.12	0.00	0.48	yh	interphase of mitotic cell cycle
GO:0006917	-0.05	0.26	0.04	0.68	0.00	0.81	yh	induction of apoptosis
GO:0042254	0.09	0.34	0.00	0.71	0.04	0.99	yh	ribosome biogenesis
GO:0006611	-0.17	0.60	-0.15	0.69	0.02	0.80	yh	protein export from nucleus
GO:0006260	-0.13	0.28	0.06	0.22	0.00	0.63	yh	DNA replication
GO:0031570	-0.21	0.59	0.01	0.37	0.00	0.99	yh	DNA integrity checkpoint
GO:0042770	-0.10	0.54	0.03	0.66	0.00	0.93	yh	DNA damage response, signal transduction
GO:0000077	-0.26	0.57	0.02	0.25	0.00	0.97	yh	DNA damage checkpoint
GO:0009314	-0.11	0.30	0.10	0.52	0.03	0.50	yh	response to radiation
GO:0007126	-0.12	0.34	0.01	0.56	0.03	0.99	yh	meiosis
GO:0051321	-0.11	0.35	0.01	0.59	0.02	0.99	yh	meiotic cell cycle
GO:0051327	-0.12	0.34	0.01	0.56	0.03	0.99	yh	M phase of meiotic cell cycle
GO:0051262	-0.43	0.55	-0.06	0.13	0.02	0.93	yh	protein tetramerization
GO:0006261	-0.04	0.29	-0.02	0.86	0.03	0.95	yh	DNA-dependent DNA replication
GO:0046466	-0.39	0.53	0.14	0.21	0.02	0.60	yh	membrane lipid catabolic process
GO:0007164	-0.61	0.84	-0.30	0.26	0.02	0.71	yh	establishment of tissue polarity
GO:0000226	-0.12	0.29	0.12	0.48	0.03	0.38	yh	microtubule cytoskeleton organization and biogenesis
GO:0007017	-0.02	0.23	0.11	0.90	0.01	0.15	yh	microtubule-based process
GO:0032271	-0.46	0.58	-0.03	0.15	0.03	0.98	yh	regulation of protein polymerization
GO:0007346	-0.21	0.36	0.10	0.22	0.01	0.49	yh	regulation of mitotic cell cycle
GO:0007067	-0.11	0.41	0.12	0.32	0.00	0.09	yh	mitosis
GO:0007088	-0.08	0.47	0.07	0.76	0.00	0.78	yh	regulation of mitosis

GO:0006767	-0.08	0.45	-0.10	0.81	0.03	0.82	yh	water-soluble vitamin metabolic process
GO:0006769	0.11	0.59	-0.14	0.81	0.05	0.82	yh	nicotinamide metabolic process
GO:0006691	-0.30	0.62	-0.19	0.48	0.04	0.80	yh	leukotriene metabolic process
GO:0043449	-0.30	0.62	-0.19	0.48	0.04	0.80	yh	alkene metabolic process
GO:0043450	-0.39	0.65	-0.17	0.36	0.04	0.81	yh	alkene biosynthetic process
GO:0019370	-0.39	0.65	-0.17	0.36	0.04	0.81	yh	leukotriene biosynthetic process
GO:0046034	0.04	0.43	-0.29	0.88	0.04	0.48	yh	ATP metabolic process
GO:0044265	-0.08	0.18	0.07	0.40	0.02	0.48	yh	cellular macromolecule catabolic process
GO:0045840	0.96	1.18	-0.44	0.23	0.00	0.56	yh	positive regulation of mitosis
GO:0001906	0.23	0.67	-0.15	0.64	0.03	0.82	yh	cell killing
GO:0001909	0.33	0.68	-0.15	0.51	0.05	0.82	yh	leukocyte mediated cytotoxicity
GO:0002228	0.33	0.68	-0.15	0.51	0.05	0.82	yh	natural killer cell mediated immunity
GO:0042267	0.33	0.68	-0.15	0.51	0.05	0.82	yh	natural killer cell mediated cytotoxicity
GO:0006984	-0.50	0.55	0.07	0.14	0.05	0.90	yh	ER-nuclear signaling pathway
GO:0045639	-0.27	0.53	-0.27	0.41	0.03	0.60	yh	positive regulation of myeloid cell differentiation
GO:0030833	-0.47	0.71	-0.10	0.23	0.01	0.89	yh	regulation of actin filament polymerization
GO:0006818	-0.00	0.43	-0.17	1.00	0.01	0.61	yh	hydrogen transport
GO:0015992	0.02	0.36	-0.16	0.94	0.04	0.67	yh	proton transport
GO:0006275	0.04	0.50	-0.19	0.92	0.05	0.75	yh	regulation of DNA replication
GO:0045648	-0.61	1.06	-0.29	0.25	0.00	0.60	yh	positive regulation of erythrocyte differentiation
GO:0006270	-0.04	0.56	-0.04	0.91	0.01	0.94	yh	DNA replication initiation
GO:0002456	-0.08	0.79	-0.13	0.91	0.01	0.85	yh	T cell mediated immunity
GO:0051648	-0.05	0.52	0.01	0.91	0.05	0.99	yh	vesicle localization
GO:0051650	-0.07	0.53	0.02	0.87	0.05	0.99	yh	establishment of vesicle localization
GO:0048730	-0.38	0.46	0.13	0.08	0.01	0.51	yh	epidermis morphogenesis
GO:0009411	-0.36	0.62	0.13	0.19	0.00	0.61	yh	response to UV
GO:0048284	-0.20	0.72	-0.11	0.77	0.05	0.91	yh	organelle fusion
GO:0008634	-0.55	0.82	-0.11	0.24	0.01	0.88	yh	negative regulation of survival gene product expression
GO:0008053	-0.34	0.76	-0.07	0.59	0.04	0.94	yh	mitochondrial fusion
GO:0006739	-0.02	0.67	-0.20	0.99	0.03	0.79	yh	NADP metabolic process
GO:0001819	0.33	0.65	-0.35	0.47	0.04	0.60	yh	positive regulation of cytokine production
GO:0000086	-0.09	0.61	0.01	0.85	0.03	0.99	yh	G2/M transition of mitotic cell cycle
GO:0007059	-0.16	0.47	0.04	0.52	0.01	0.93	yh	chromosome segregation
GO:0031069	-0.27	0.89	-0.26	0.73	0.00	0.77	yh	hair follicle morphogenesis
GO:0006302	-0.24	0.52	-0.11	0.37	0.01	0.81	yh	double-strand break repair
GO:0007032	-0.40	1.16	-0.33	0.56	0.00	0.54	yh	endosome organization and biogenesis
GO:0007051	0.33	0.55	0.04	0.37	0.05	0.95	yh	spindle organization and biogenesis
GO:0032635	0.37	0.97	-0.56	0.65	0.00	0.39	yh	interleukin-6 production
GO:0042226	0.37	0.97	-0.56	0.65	0.00	0.39	yh	interleukin-6 biosynthetic process
GO:0008593	-0.69	0.82	-0.15	0.06	0.00	0.80	yh	regulation of Notch signaling pathway
GO:0030330	0.27	0.62	-0.07	0.52	0.03	0.93	yh	DNA damage response, signal transduction by p53 class mediator
GO:0030968	-0.61	0.72	0.01	0.10	0.01	0.99	yh	endoplasmic reticulum unfolded protein response
GO:0046479	0.43	0.91	-0.15	0.60	0.03	0.87	yh	glycosphingolipid catabolic process
GO:0006829	0.02	0.78	-0.36	0.98	0.04	0.71	yh	zinc ion transport
GO:0007020	-0.76	0.75	-0.22	0.06	0.04	0.78	yh	microtubule nucleation
GO:0001501	0.12	-0.19	0.01	0.25	0.04	0.99	yl	skeletal development
GO:0003012	0.05	-0.28	-0.10	0.72	0.01	0.67	yl	muscle system process
GO:0006936	0.05	-0.28	-0.10	0.72	0.01	0.67	yl	muscle contraction
GO:0019226	0.13	-0.25	-0.14	0.14	0.00	0.24	yl	transmission of nerve impulse
GO:0006939	0.11	-0.49	-0.10	0.74	0.04	0.85	yl	smooth muscle contraction
GO:0007417	-0.09	-0.22	0.03	0.44	0.02	0.90	yl	central nervous system development
GO:0022008	0.11	-0.30	0.02	0.27	0.00	0.90	yl	neurogenesis
GO:0048699	0.15	-0.32	0.02	0.12	0.00	0.93	yl	generation of neurons
GO:0007420	0.01	-0.25	-0.02	0.97	0.05	0.96	yl	brain development
GO:0030900	-0.02	-0.56	-0.10	0.95	0.01	0.82	yl	forebrain development
GO:0008285	-0.05	-0.19	0.03	0.70	0.04	0.87	yl	negative regulation of cell proliferation
GO:0021700	0.09	-0.32	-0.04	0.66	0.05	0.93	yl	developmental maturation
GO:0032990	0.13	-0.20	-0.01	0.24	0.05	0.99	yl	cell part morphogenesis
GO:0030030	0.13	-0.20	-0.01	0.24	0.05	0.99	yl	cell projection organization and biogenesis
GO:0031175	0.15	-0.24	0.03	0.28	0.03	0.93	yl	neurite development
GO:0048666	0.18	-0.26	0.01	0.13	0.01	0.99	yl	neuron development
GO:0048858	0.13	-0.20	-0.01	0.24	0.05	0.99	yl	cell projection morphogenesis
GO:0007517	0.03	-0.33	0.00	0.84	0.00	0.99	yl	muscle development
GO:0014706	0.01	-0.30	-0.02	0.95	0.03	0.97	yl	striated muscle development
GO:0008361	-0.04	-0.25	0.02	0.78	0.02	0.95	yl	regulation of cell size
GO:0040007	-0.10	-0.24	-0.05	0.34	0.01	0.81	yl	growth
GO:0016049	-0.02	-0.28	0.02	0.88	0.01	0.94	yl	cell growth
GO:0007519	0.03	-0.29	-0.03	0.90	0.05	0.94	yl	skeletal muscle development
GO:0000271	0.20	-0.59	-0.11	0.63	0.04	0.90	yl	polysaccharide biosynthetic process
GO:0033692	0.20	-0.59	-0.11	0.63	0.04	0.90	yl	cellular polysaccharide biosynthetic process
GO:0007268	0.14	-0.26	-0.18	0.12	0.00	0.13	yl	synaptic transmission
GO:0009566	0.06	-0.48	-0.30	0.82	0.03	0.51	yl	fertilization
GO:0007338	0.06	-0.48	-0.29	0.82	0.03	0.53	yl	single fertilization
GO:0048741	0.23	-0.44	-0.04	0.30	0.01	0.95	yl	skeletal muscle fiber development
GO:0048747	0.23	-0.44	-0.04	0.30	0.01	0.95	yl	muscle fiber development
GO:0030239	0.74	-0.95	0.08	0.06	0.00	0.90	yl	myofibril assembly
GO:0045445	0.38	-0.50	0.01	0.15	0.02	0.99	yl	myoblast differentiation
GO:0051146	0.37	-0.69	0.01	0.35	0.02	0.99	yl	striated muscle cell differentiation
GO:0055001	0.74	-0.95	0.08	0.06	0.00	0.90	yl	muscle cell development
GO:0055002	0.74	-0.95	0.08	0.06	0.00	0.90	yl	striated muscle cell development
GO:0040008	0.02	-0.30	-0.01	0.88	0.00	0.97	yl	regulation of growth
GO:0035295	0.19	-0.24	0.06	0.18	0.04	0.78	yl	tube development
GO:0006022	0.18	-0.61	-0.15	0.52	0.00	0.77	yl	aminoglycan metabolic process
GO:0030203	0.16	-0.58	-0.14	0.56	0.00	0.78	yl	glycosaminoglycan metabolic process
GO:0001558	0.07	-0.29	0.01	0.70	0.01	0.98	yl	regulation of cell growth
GO:0003013	0.17	-0.39	-0.06	0.18	0.00	0.81	yl	circulatory system process
GO:0008015	0.17	-0.39	-0.06	0.18	0.00	0.81	yl	blood circulation
GO:0002541	0.50	-0.58	-0.07	0.08	0.01	0.93	yl	activation of plasma proteins during acute inflammatory response
GO:0006956	0.50	-0.58	-0.07	0.08	0.01	0.93	yl	complement activation
GO:0006575	0.27	-0.30	0.10	0.13	0.04	0.63	yl	amino acid derivative metabolic process
GO:0018958	0.19	-0.63	-0.25	0.58	0.01	0.68	yl	phenol metabolic process
GO:0003018	0.29	-0.49	-0.06	0.34	0.05	0.94	yl	vascular process in circulatory system
GO:0019229	0.59	-1.12	-0.14	0.36	0.00	0.90	yl	regulation of vasoconstriction
GO:0035150	0.32	-0.50	-0.05	0.30	0.04	0.95	yl	regulation of tube size
GO:0042310	0.51	-0.80	-0.10	0.27	0.01	0.93	yl	vasoconstriction
GO:0050880	0.32	-0.50	-0.05	0.30	0.04	0.95	yl	regulation of blood vessel size
GO:0006584	0.21	-0.65	-0.24	0.54	0.01	0.71	yl	catecholamine metabolic process
GO:0019216	0.48	-0.63	0.08	0.16	0.02	0.89	yl	regulation of lipid metabolic process
GO:0030111	-0.12	-0.56	0.06	0.73	0.02	0.90	yl	regulation of Wnt receptor signaling pathway
GO:0016358	0.53	-0.52	-0.05	0.07	0.05	0.96	yl	dendrite development
GO:0006721	-0.56	-0.93	-0.34	0.25	0.02	0.67	yl	terpenoid metabolic process
GO:0016101	-0.38	-0.98	-0.28	0.52	0.02	0.78	yl	diterpenoid metabolic process
GO:0001523	-0.38	-0.98	-0.28	0.52	0.02	0.78	yl	retinoid metabolic process
GO:0006778	-0.02	-0.92	-0.46	0.99	0.00	0.46	yl	porphyrin metabolic process
GO:0033013	-0.02	-0.92	-0.46	0.99	0.00	0.46	yl	tetrapyrrole metabolic process
GO:0006779	-0.06	-0.71	-0.27	0.93	0.03	0.75	yl	porphyrin biosynthetic process

GO:0033014	-0.06	-0.71	-0.27	0.93	0.03	0.75	yl	tetrapyrrole biosynthetic process
GO:0042168	0.08	-0.93	-0.43	0.87	0.00	0.51	yl	heme metabolic process
GO:0006783	0.04	-0.71	-0.23	0.93	0.04	0.80	yl	heme biosynthetic process
GO:0006937	-0.06	-0.66	-0.11	0.89	0.01	0.84	yl	regulation of muscle contraction
GO:0001936	-0.08	-0.95	-0.23	0.92	0.02	0.81	yl	regulation of endothelial cell proliferation
GO:0006662	0.32	-0.77	-0.12	0.50	0.01	0.90	yl	glycerol ether metabolic process
GO:0009612	0.49	-0.87	-0.41	0.45	0.05	0.75	yl	response to mechanical stimulus
GO:0042311	0.74	-0.80	0.10	0.08	0.01	0.88	yl	vasodilation
GO:0006638	0.38	-0.87	-0.21	0.47	0.01	0.82	yl	neutral lipid metabolic process
GO:0006639	0.38	-0.87	-0.21	0.47	0.01	0.82	yl	acylglycerol metabolic process
GO:0046486	0.37	-0.90	-0.12	0.46	0.00	0.90	yl	glycerolipid metabolic process
GO:0030178	0.34	-0.90	0.08	0.44	0.00	0.90	yl	negative regulation of Wnt receptor signaling pathway
GO:0046928	0.11	-0.89	-0.17	0.87	0.02	0.86	yl	regulation of neurotransmitter secretion
GO:0048168	-0.46	-0.97	-0.20	0.43	0.02	0.82	yl	regulation of neuronal synaptic plasticity
GO:0007398	-0.27	0.38	0.13	0.01	0.00	0.04	no_rel	ectoderm development
GO:0000278	-0.17	0.36	0.12	0.03	0.00	0.00	no_rel	mitotic cell cycle

Table 1: LOR: log odds ratio; p: adjusted p-value FDR

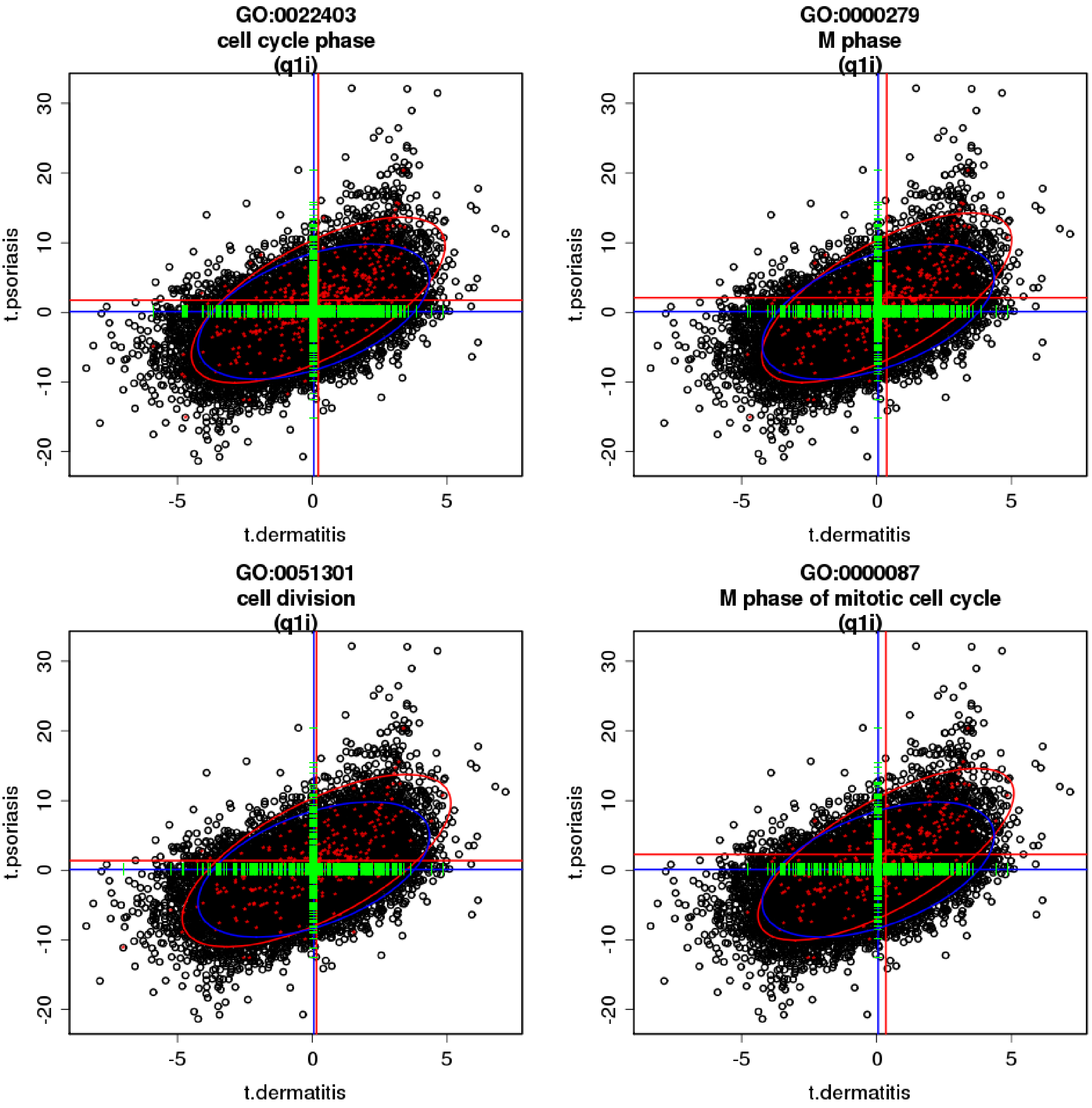

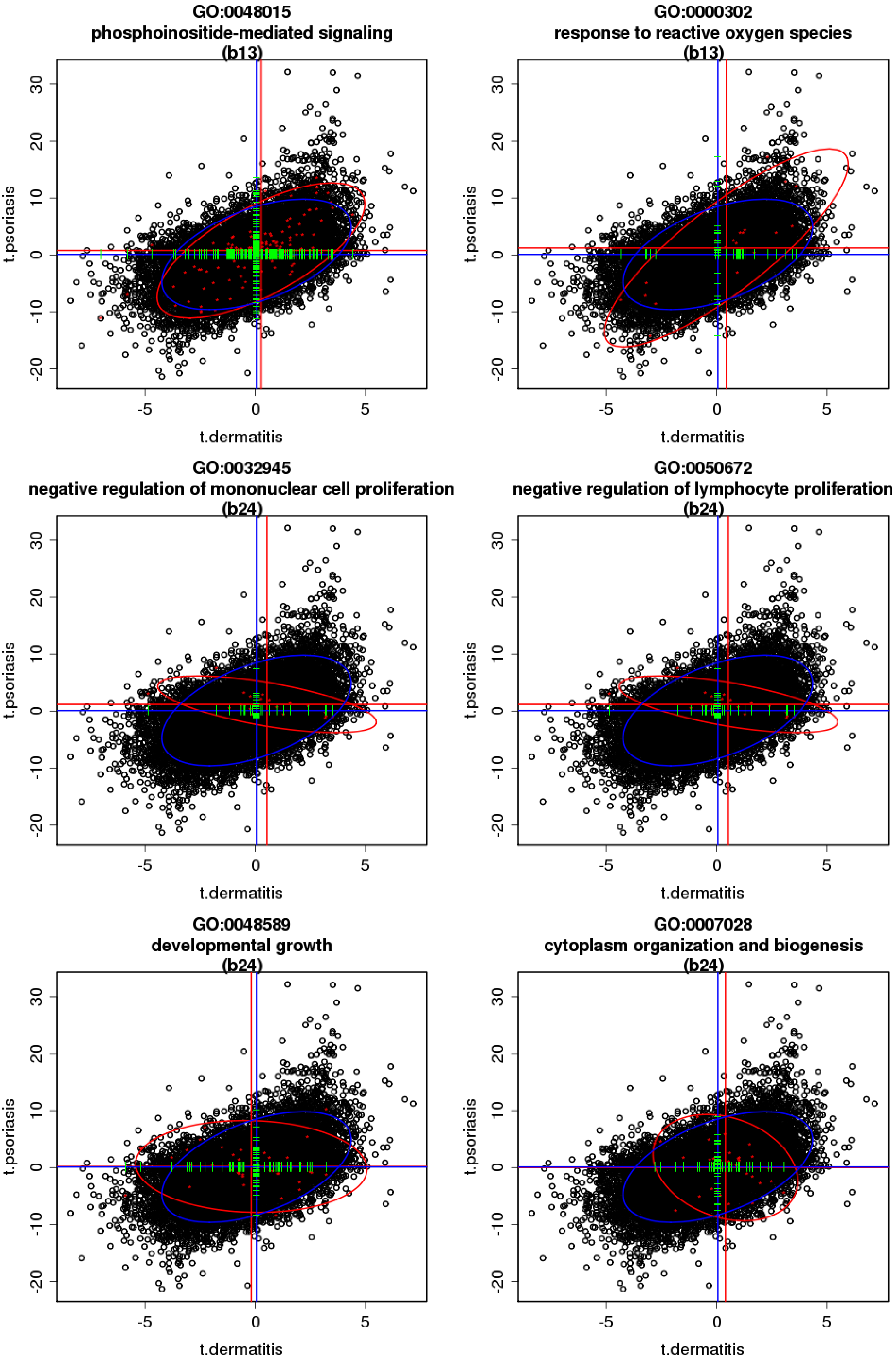
GO:0048015
phosphoinositide-mediated signaling
(b13)
GO:0000302
response to reactive oxygen species
(b13)
GO:0032945
negative regulation of mononuclear cell proliferation
(b24)
GO:0050672
negative regulation of lymphocyte proliferation
(b24)
GO:0048589
developmental growth
(b24)
GO:0007028
cytoplasm organization and biogenesis
(b24)
t.psoriasis
t.dermatitis

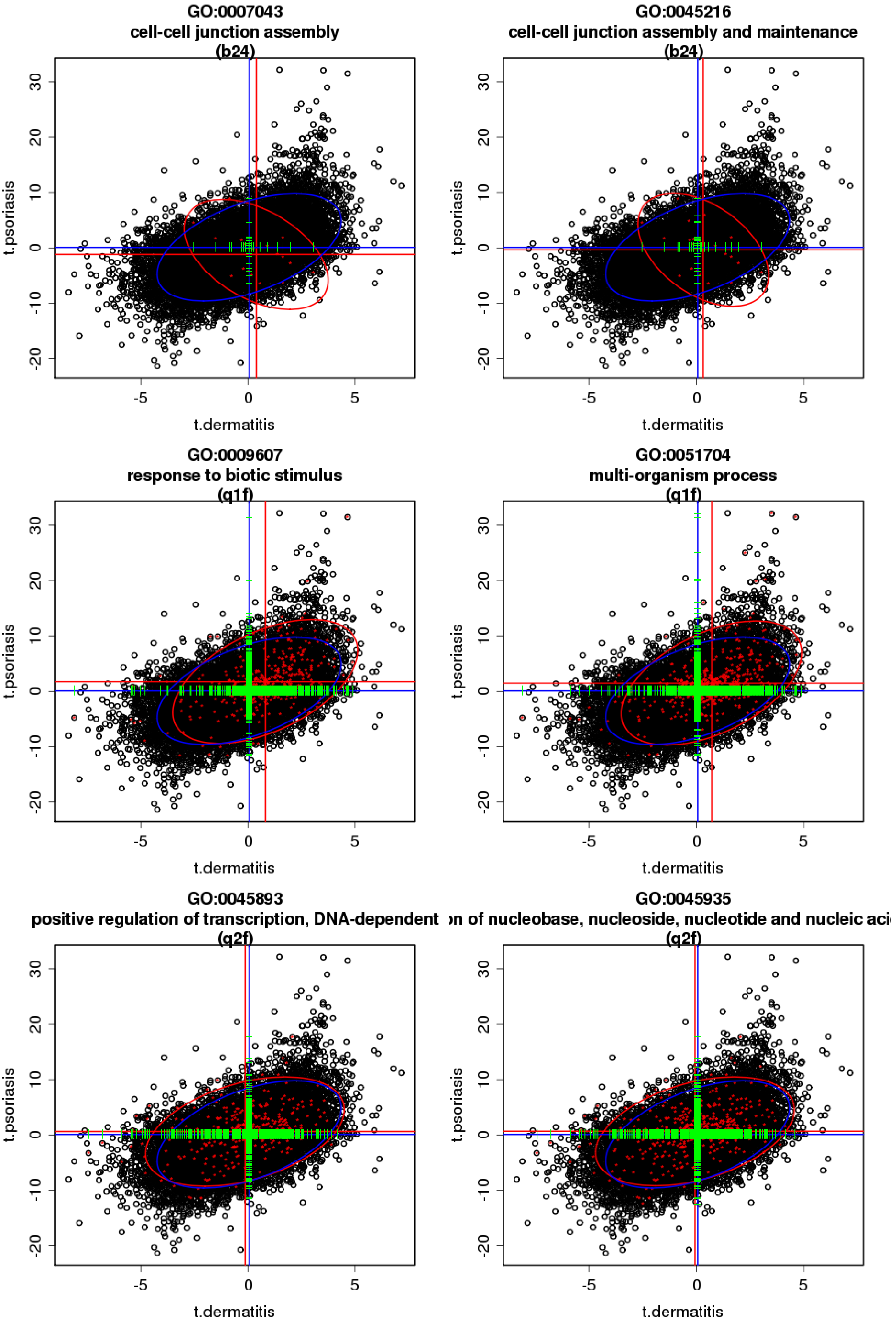
GO:0007043
cell-cell junction assembly
(b24)
t.psoriasis
t.dermatitis
GO:0045216
cell-cell junction assembly and maintenance
(b24)
t.psoriasis
t.dermatitis
GO:0009607
response to biotic stimulus
(q1f)
t.psoriasis
t.dermatitis
GO:0051704
multi-organism process
(q1f)
t.psoriasis
t.dermatitis
GO:0045893
positive regulation of transcription, DNA-dependent
(q2f)
t.psoriasis
t.dermatitis
GO:0045935
on of nucleobase, nucleoside, nucleotide and nucleic aci
(q2f)
t.psoriasis
t.dermatitis

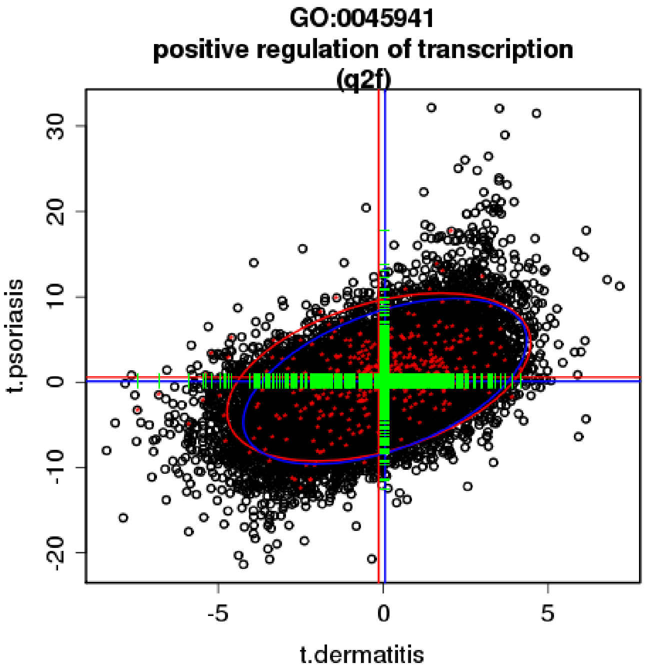
GO:0045941
positive regulation of transcription
(q2f)
t.psoriasis
t.dermatitis
30
20
10
0
-10
-20
-5
0
5

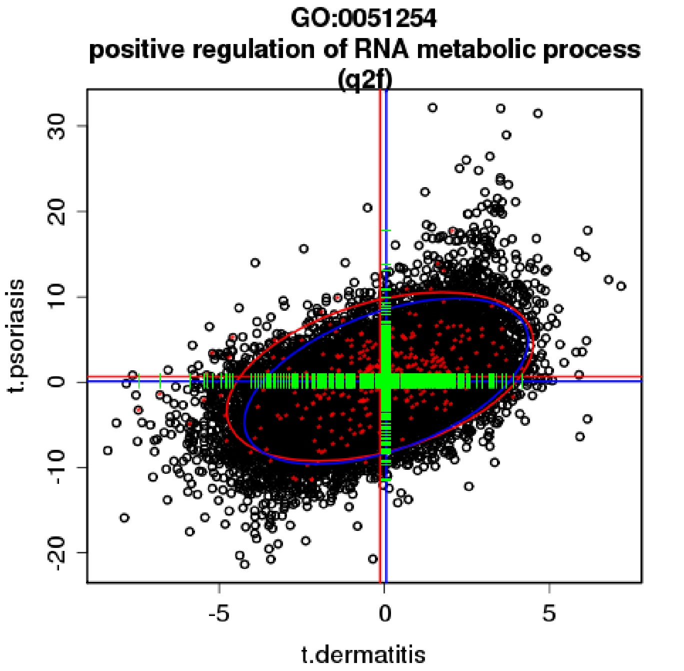
GO:0051254
positive regulation of RNA metabolic process
(q2f)
t.psoriasis
t.dermatitis
30
20
10
0
-10
-20
-5
0
5

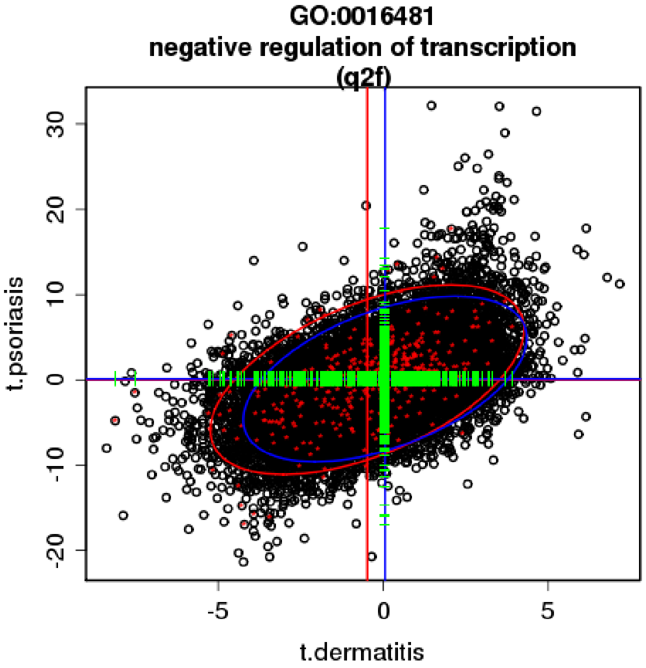
GO:0016481
negative regulation of transcription
(q2f)
t.psoriasis
t.dermatitis
30
20
10
0
-10
-20
-5
0
5

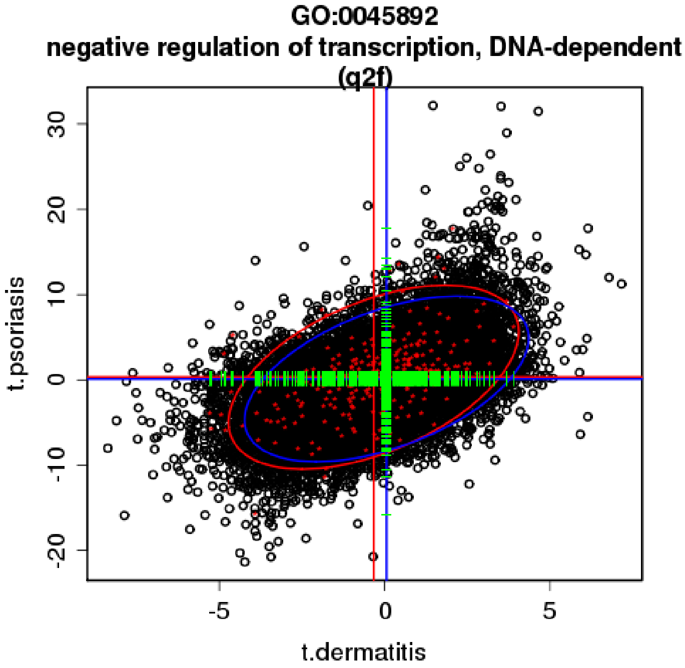
GO:0045892
negative regulation of transcription, DNA-dependent
(q2f)
t.psoriasis
t.dermatitis
30
20
10
0
-10
-20
-5
0
5

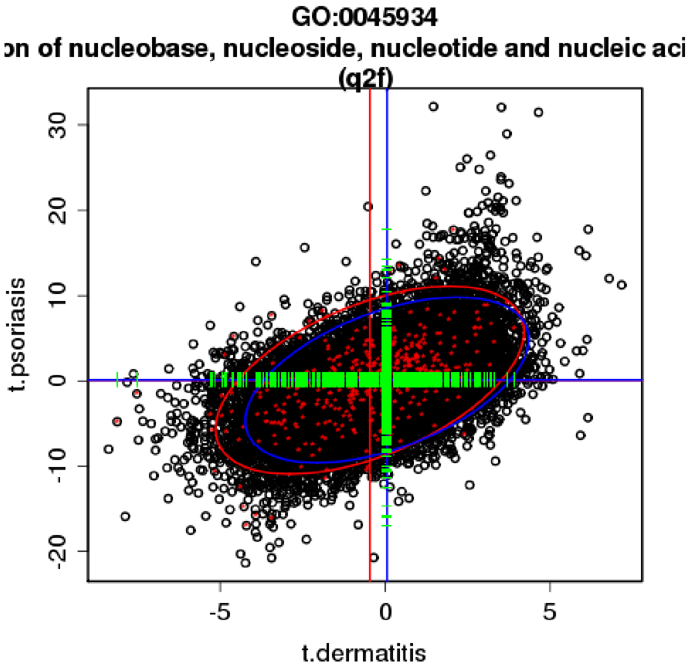
GO:0045934
on of nucleobase, nucleoside, nucleotide and nucleic aci
(q2f)
t.psoriasis
t.dermatitis
30
20
10
0
-10
-20
-5
0
5

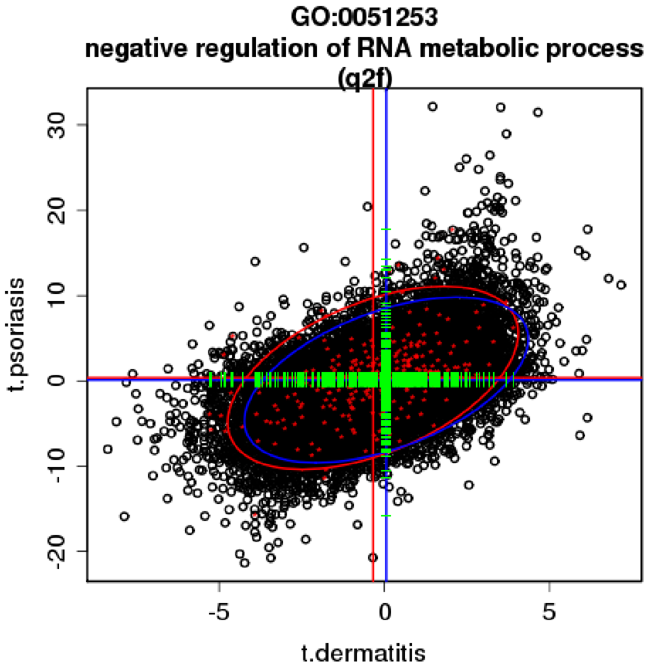
GO:0051253
negative regulation of RNA metabolic process
(q2f)
t.psoriasis
t.dermatitis
30
20
10
0
-10
-20
-5
0
5

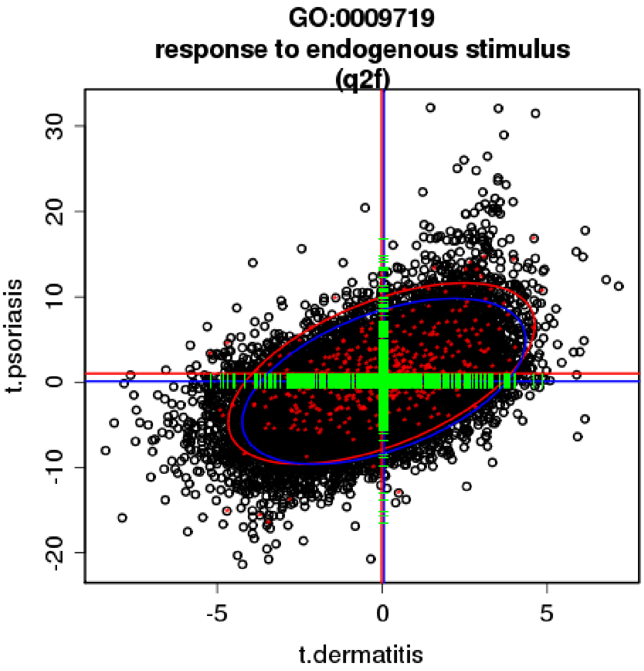
GO:0009719
response to endogenous stimulus
(q2f)
t.psoriasis
t.dermatitis

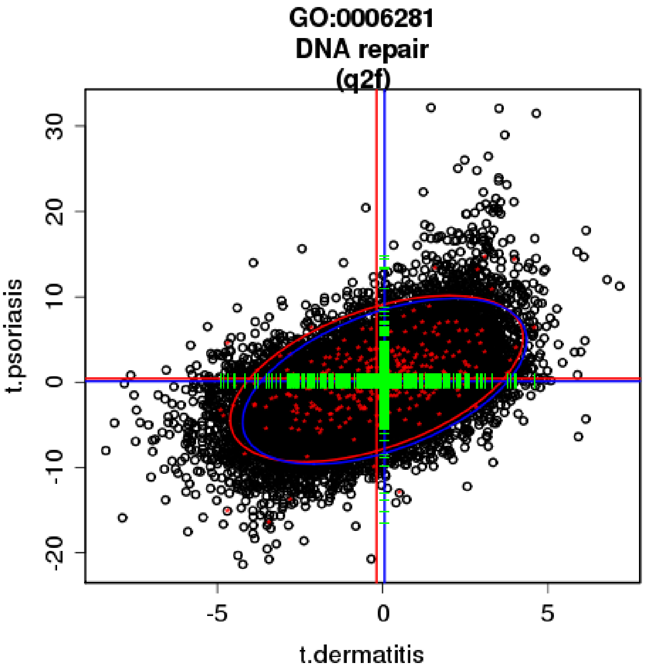
GO:0006281
DNA repair
(q2f)
t.psoriasis
t.dermatitis

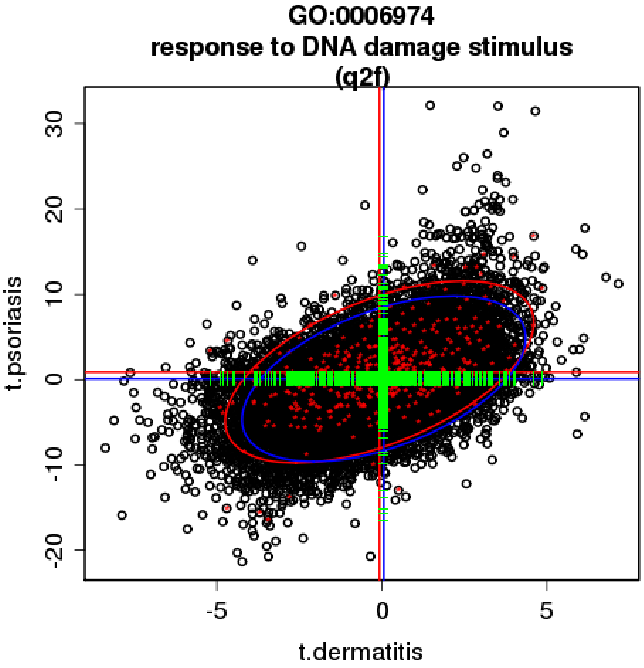
GO:0006974
response to DNA damage stimulus
(q2f)
t.psoriasis
t.dermatitis

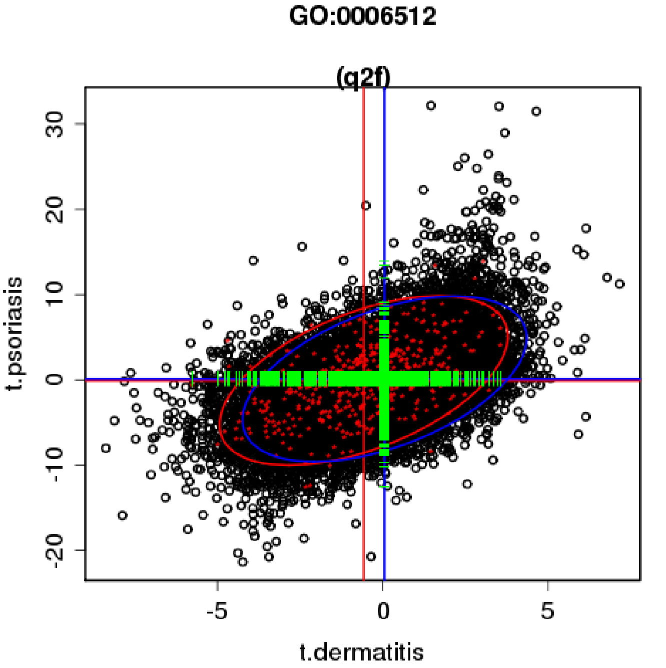
GO:0006512
(q2f)
t.psoriasis
t.dermatitis

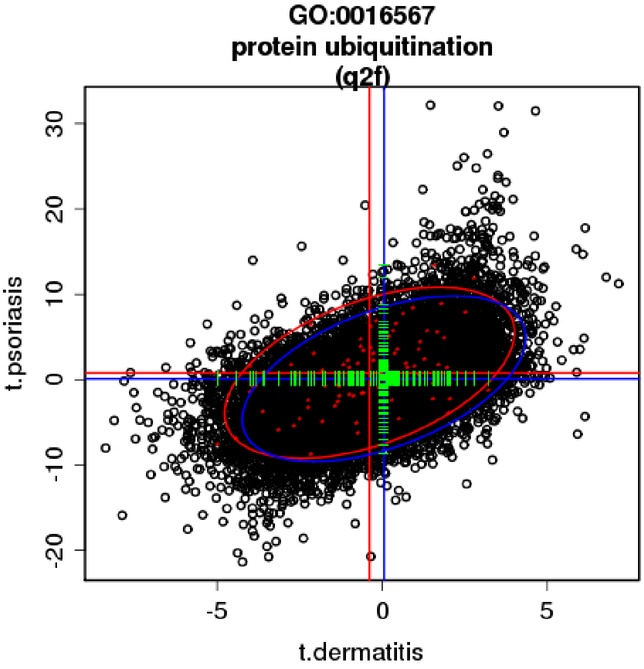
GO:0016567
protein ubiquitination
(q2f)
t.psoriasis
t.dermatitis

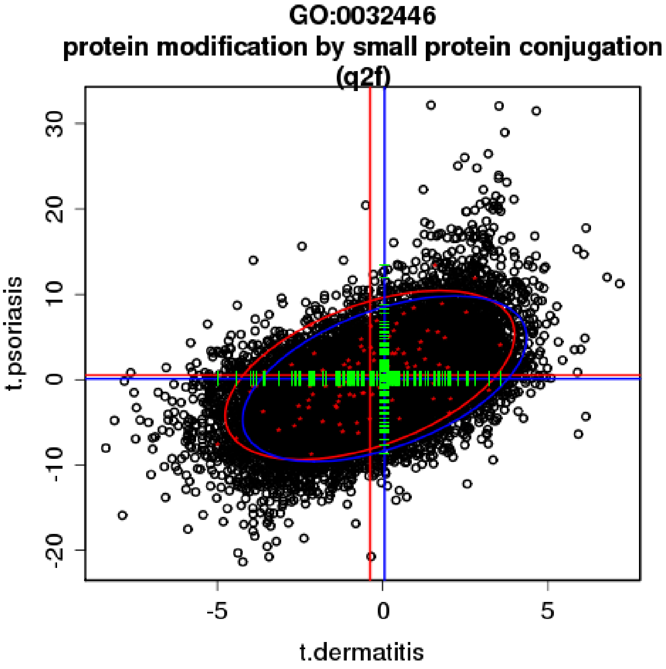
GO:0032446
protein modification by small protein conjugation
(q2f)
t.psoriasis
t.dermatitis

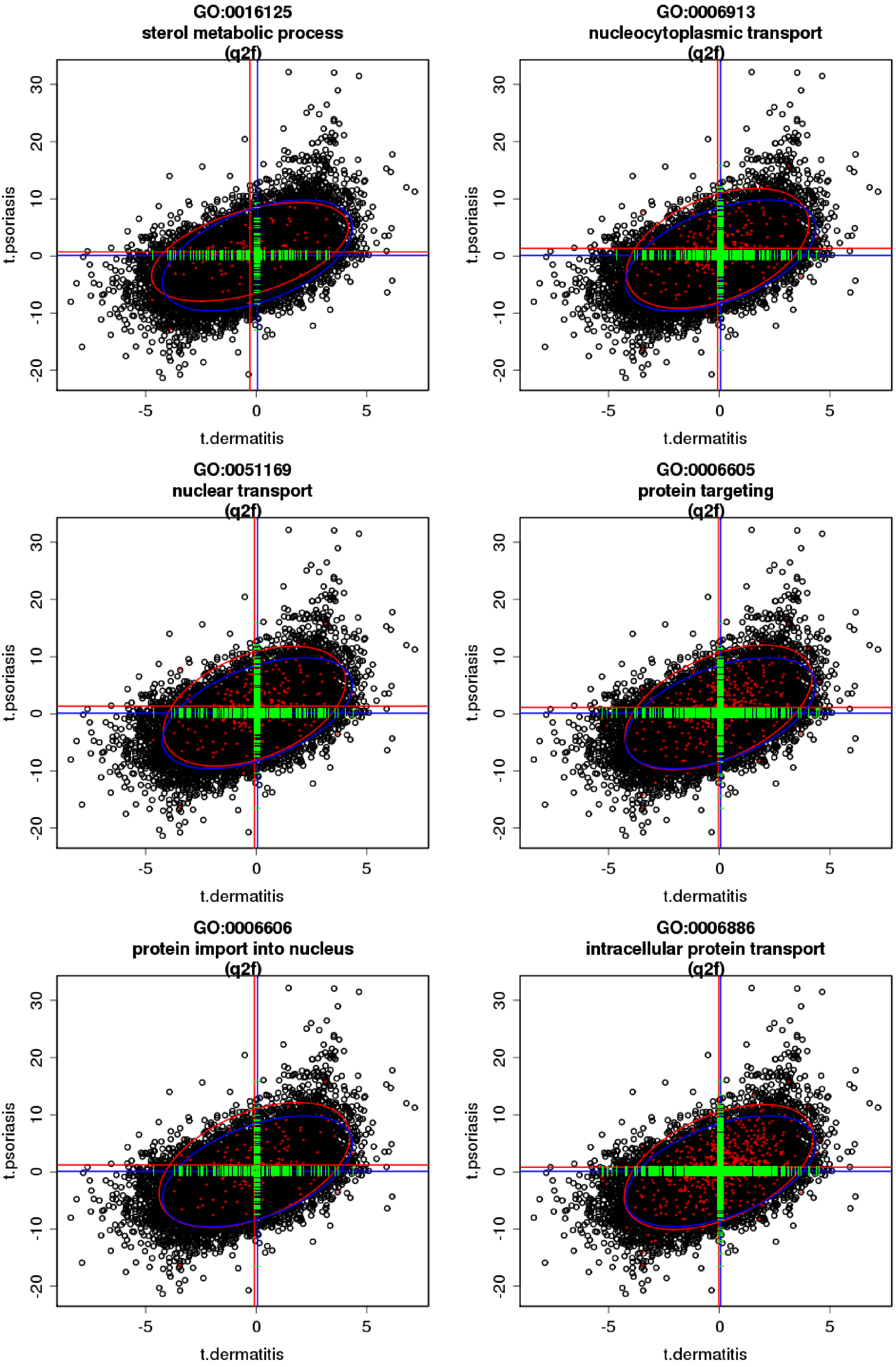
GO:0016125
sterol metabolic process
(q2f)
GO:0006913
nucleocytoplasmic transport
(q2f)
GO:0051169
nuclear transport
(q2f)
GO:0006605
protein targeting
(q2f)
GO:0006606
protein import into nucleus
(q2f)
GO:0006886
intracellular protein transport
(q2f)
t.psoriasis
t.dermatitis
-20
-10
0
10
20
30
-5
0
5

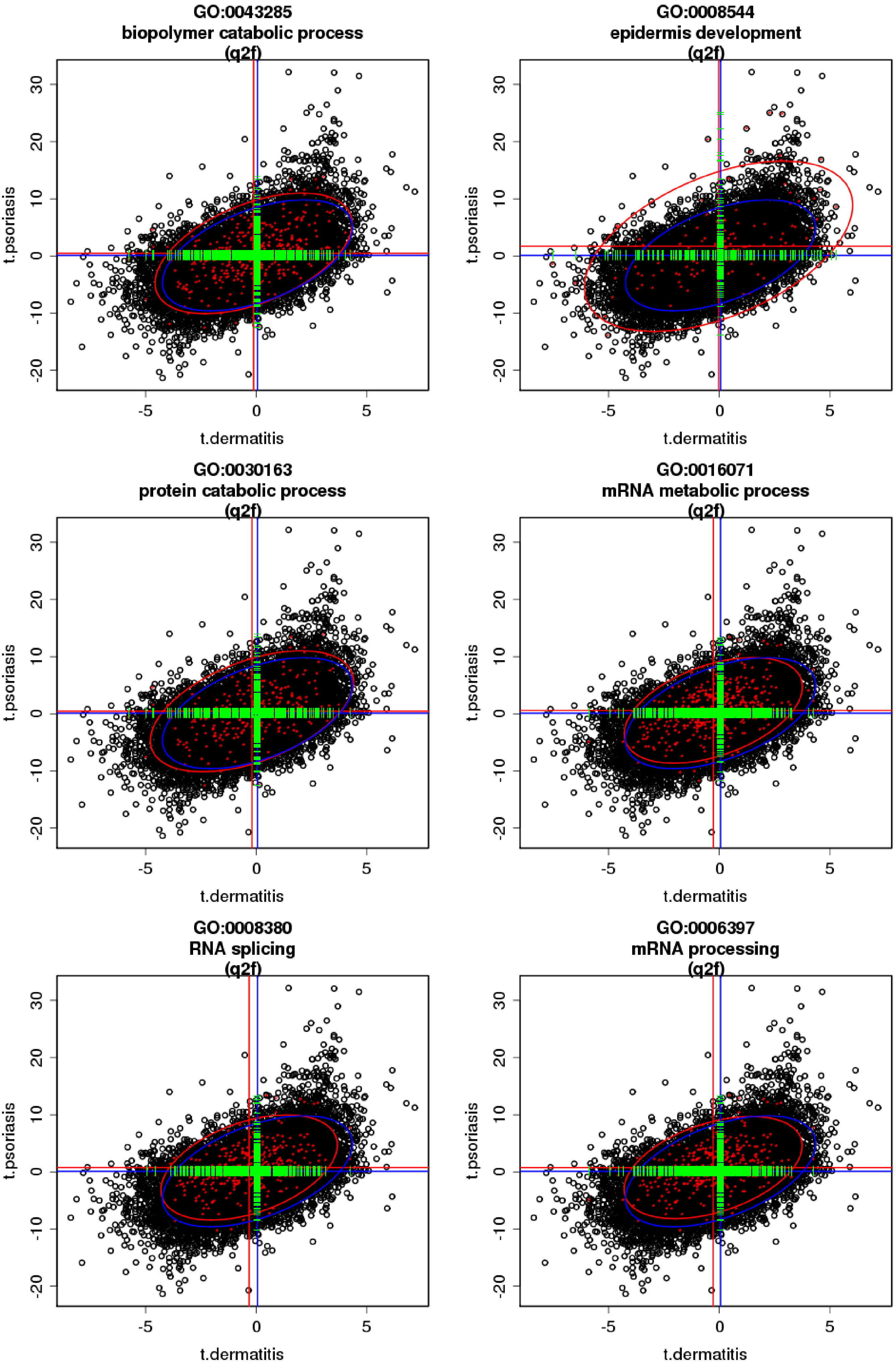
GO:0043285
biopolymer catabolic process
(q2f)
GO:0008544
epidermis development
(q2f)
GO:0030163
protein catabolic process
(q2f)
GO:0016071
mRNA metabolic process
(q2f)
GO:0008380
RNA splicing
(q2f)
GO:0006397
mRNA processing
(q2f)
t.psoriasis
t.dermatitis
-20
-10
0
10
20
30
-5
0
5

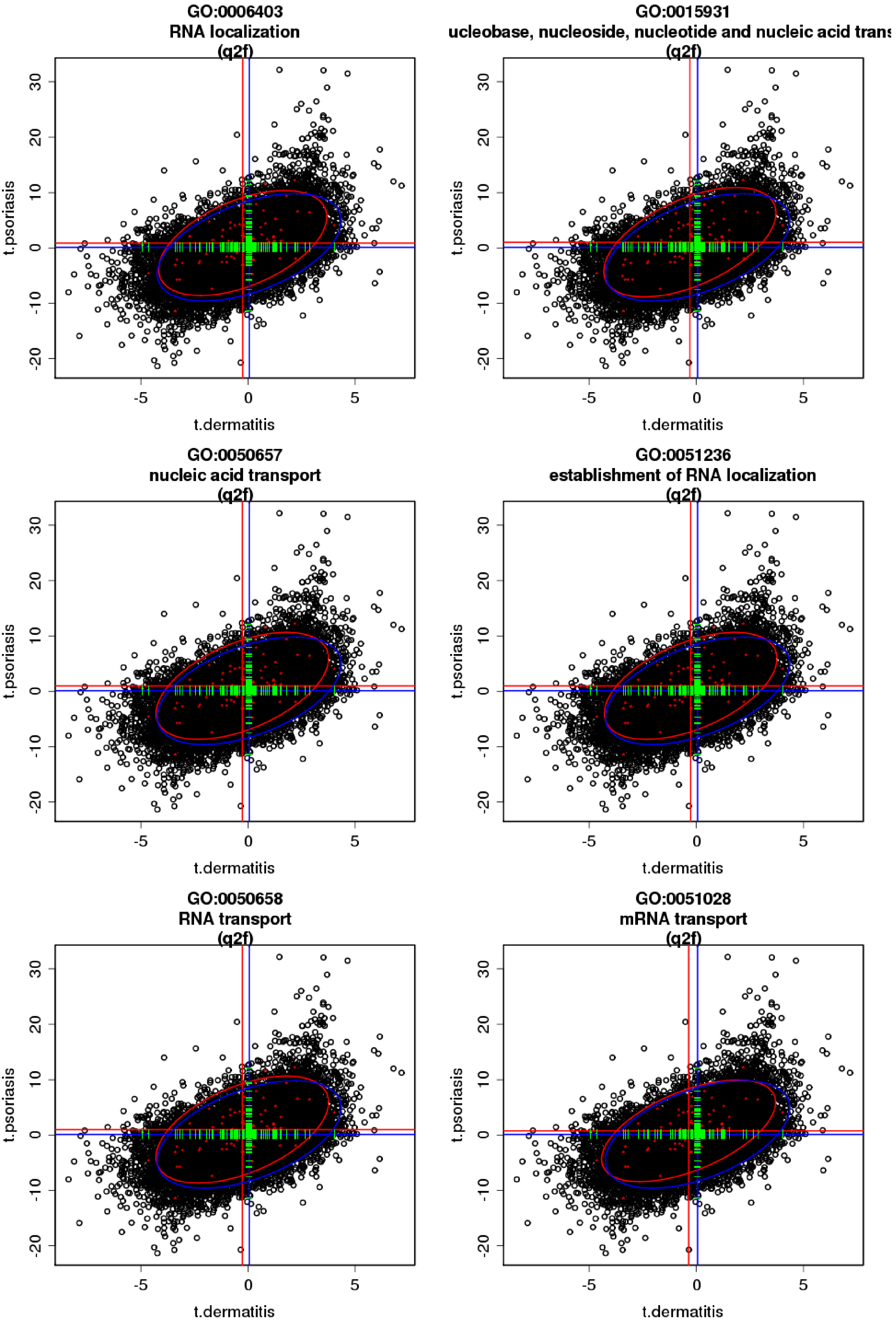
GO:0006403
RNA localization
(q2f)
GO:0015931
ucleobase, nucleoside, nucleotide and nucleic acid trans
(q2f)
GO:0050657
nucleic acid transport
(q2f)
GO:0051236
establishment of RNA localization
(q2f)
GO:0050658
RNA transport
(q2f)
GO:0051028
mRNA transport
(q2f)
t.psoriasis
t.dermatitis
-20
-10
0
10
20
30
-5
0
5

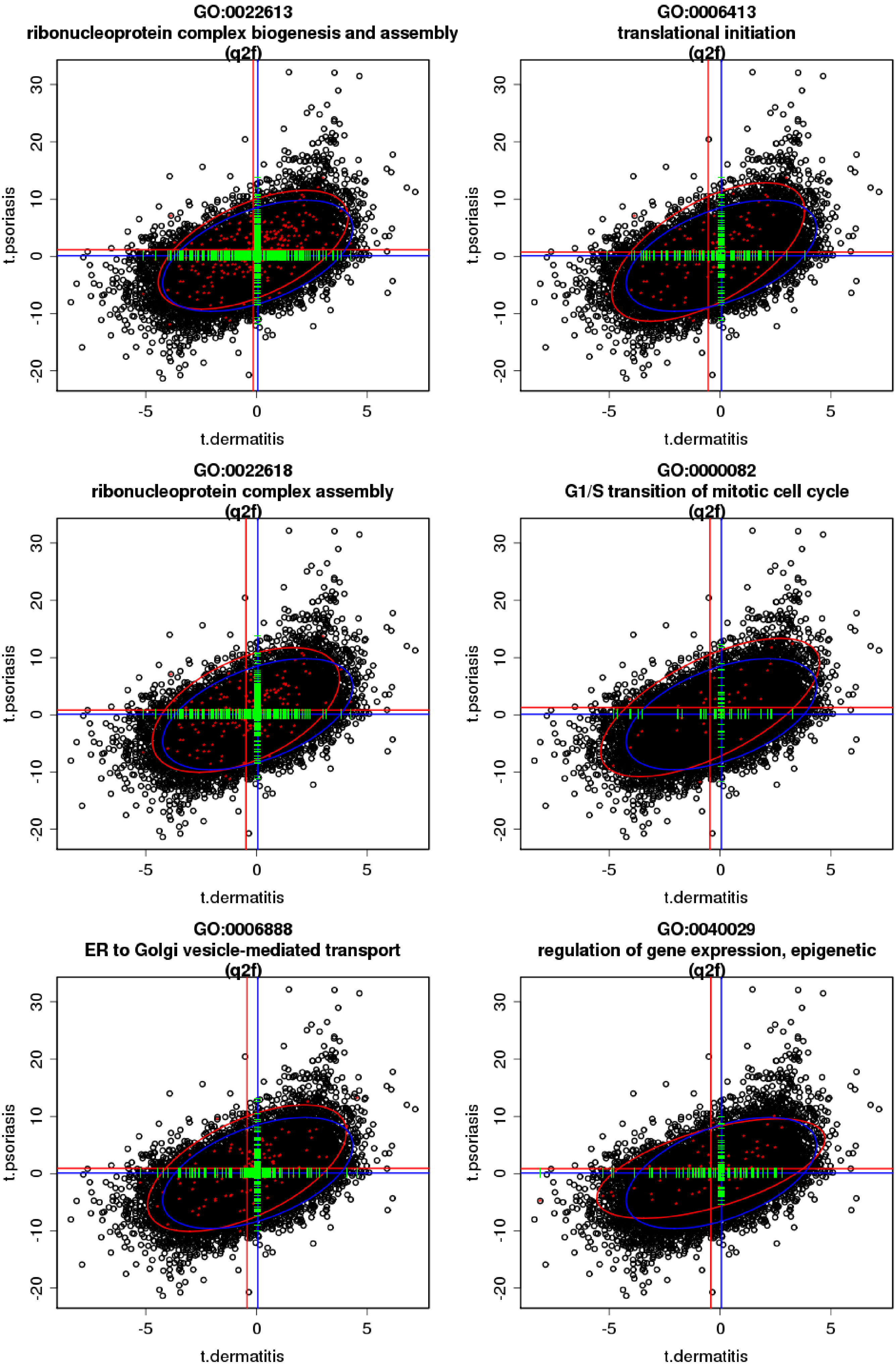
GO:0022613
ribonucleoprotein complex biogenesis and assembly
(q2f)
GO:0006413
translational initiation
(q2f)
GO:0022618
ribonucleoprotein complex assembly
(q2f)
GO:0000082
G1/S transition of mitotic cell cycle
(q2f)
GO:0006888
ER to Golgi vesicle-mediated transport
(q2f)
GO:0040029
regulation of gene expression, epigenetic
(q2f)
t.psoriasis
t.dermatitis
-20
-10
0
10
20
30
-5
0
5

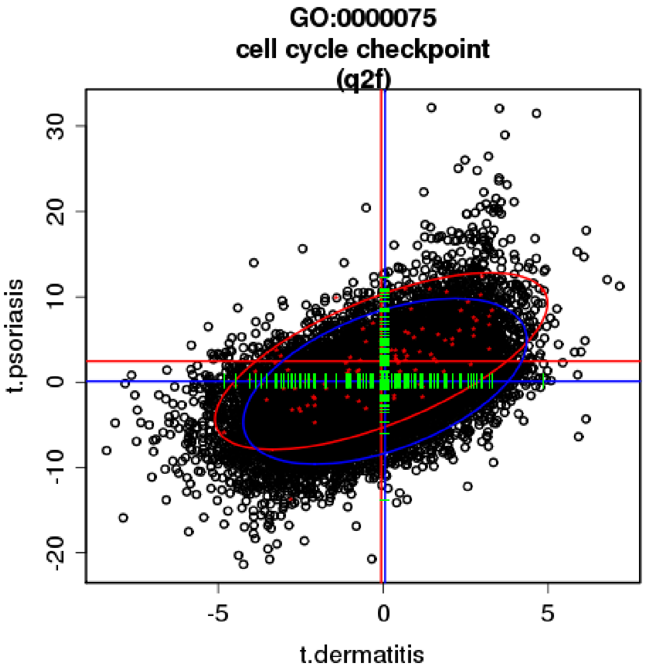
GO:0000075
cell cycle checkpoint
(q2f)
t.psoriasis
t.dermatitis
-20 -10 0 10 20 30
-5 0 5

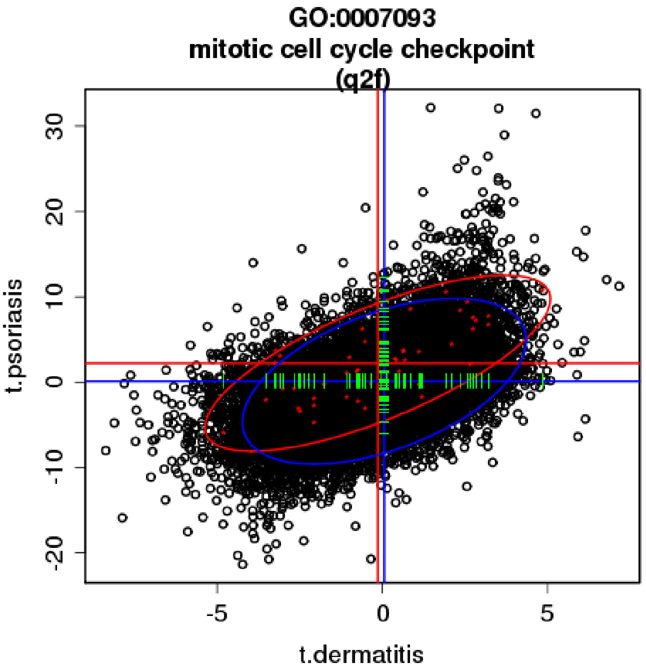
GO:0007093
mitotic cell cycle checkpoint
(q2f)
t.psoriasis
t.dermatitis
-20 -10 0 10 20 30
-5 0 5

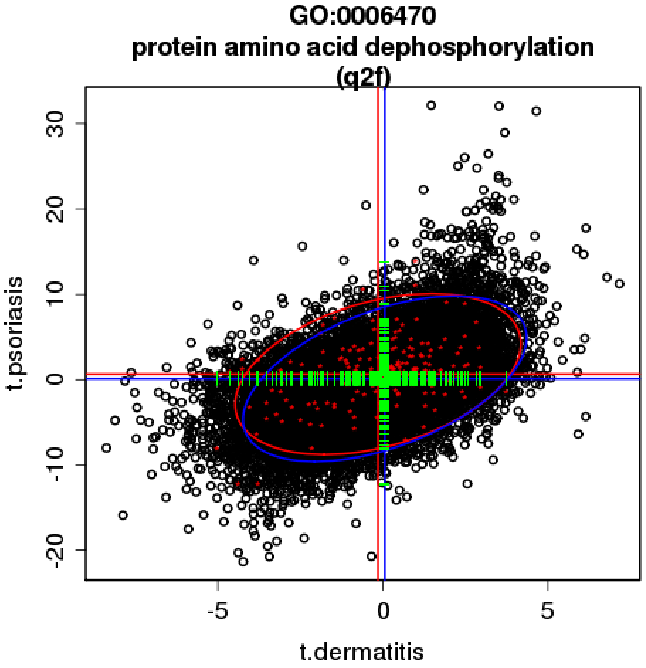
GO:0006470
protein amino acid dephosphorylation
(q2f)
t.psoriasis
t.dermatitis
-20 -10 0 10 20 30
-5 0 5

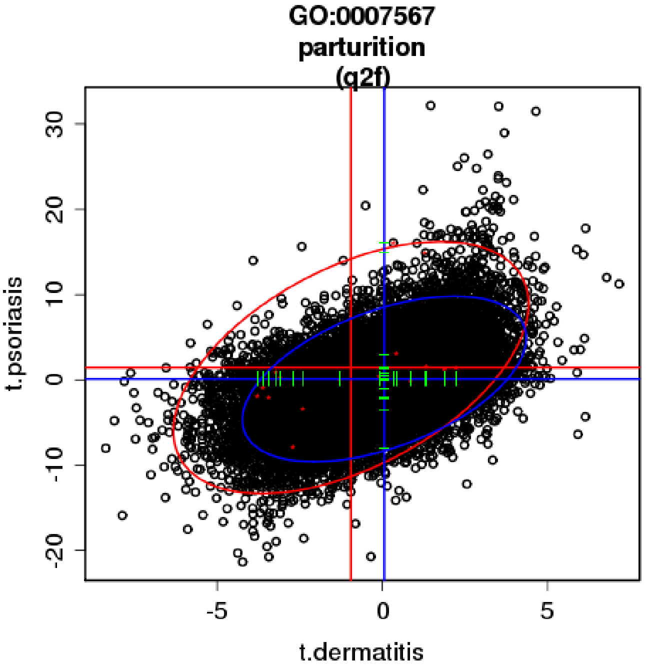
GO:0007567
parturition
(q2f)
t.psoriasis
t.dermatitis
-20 -10 0 10 20 30
-5 0 5

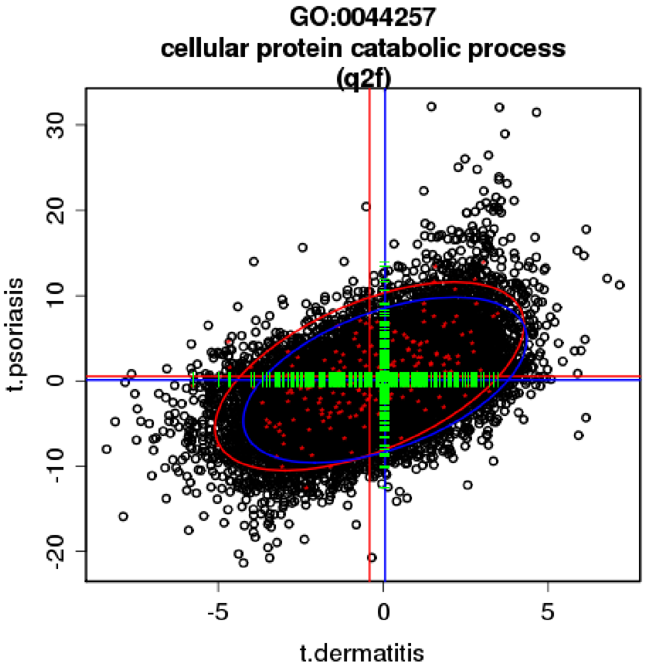
GO:0044257
cellular protein catabolic process
(q2f)
t.psoriasis
t.dermatitis
-20 -10 0 10 20 30
-5 0 5

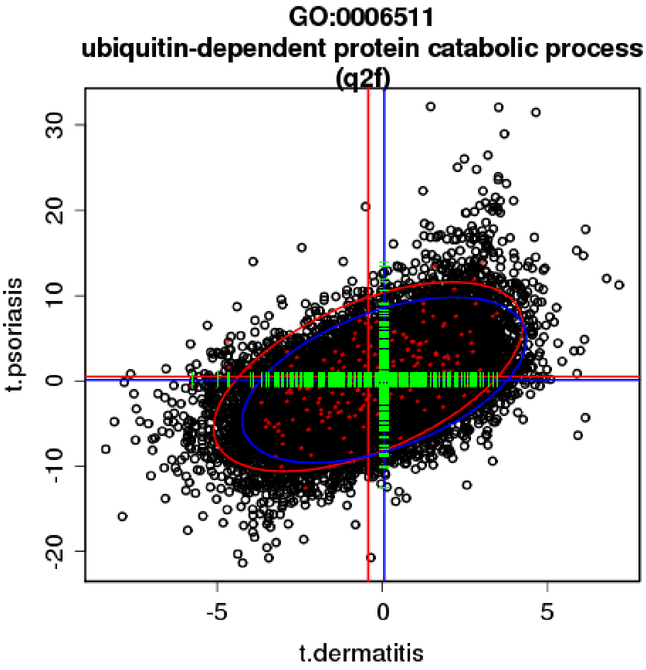
GO:0006511
ubiquitin-dependent protein catabolic process
(q2f)
t.psoriasis
t.dermatitis
-20 -10 0 10 20 30
-5 0 5

GO:0019941
modification-dependent protein catabolic process
(q2f)

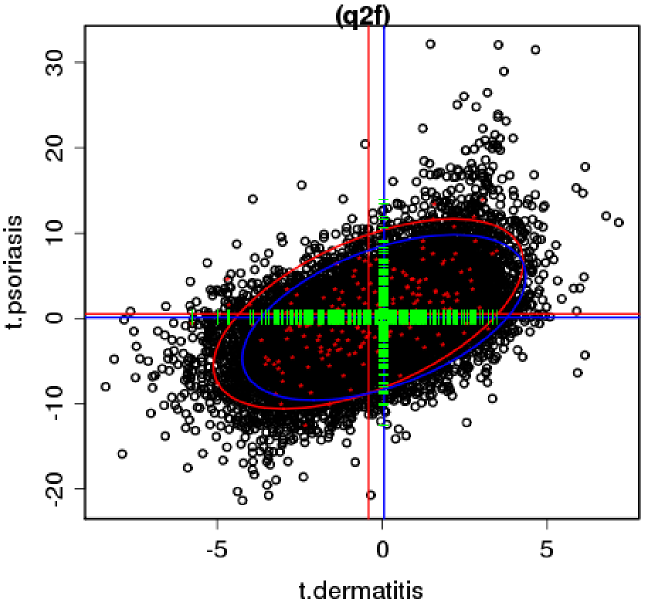

GO:0043632
modification-dependent macromolecule catabolic proce
(q2f)

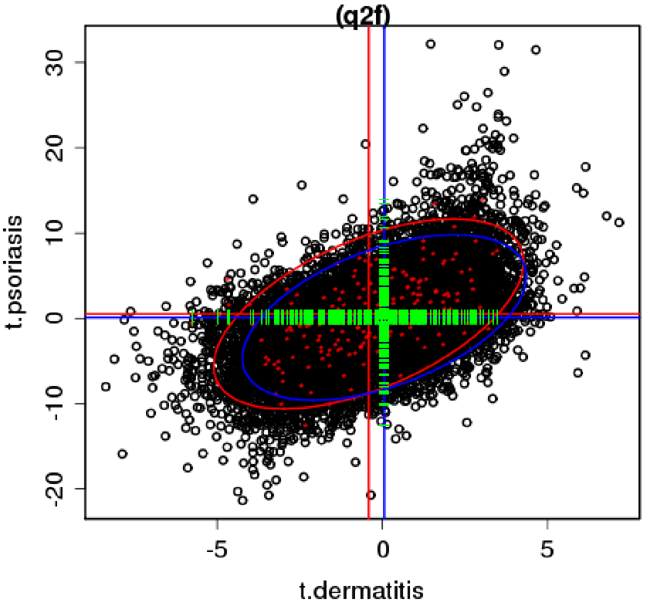

GO:0051603
proteolysis involved in cellular protein catabolic proce:
(q2f)

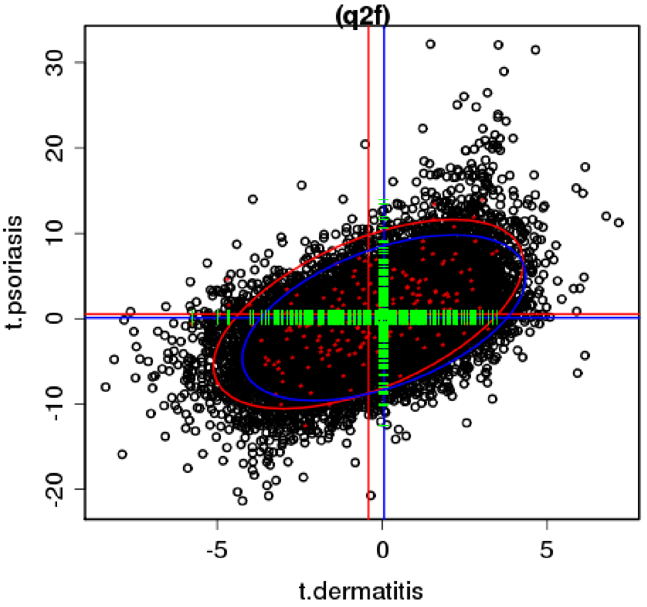

GO:0000375
RNA splicing, via transesterification reactions
(q2f)

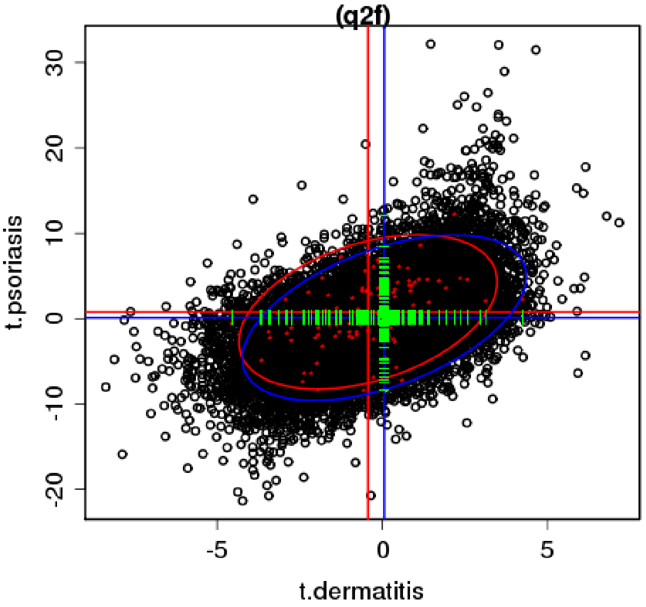

GO:0000377
j, via transesterification reactions with bulged adenosine
(q2f)

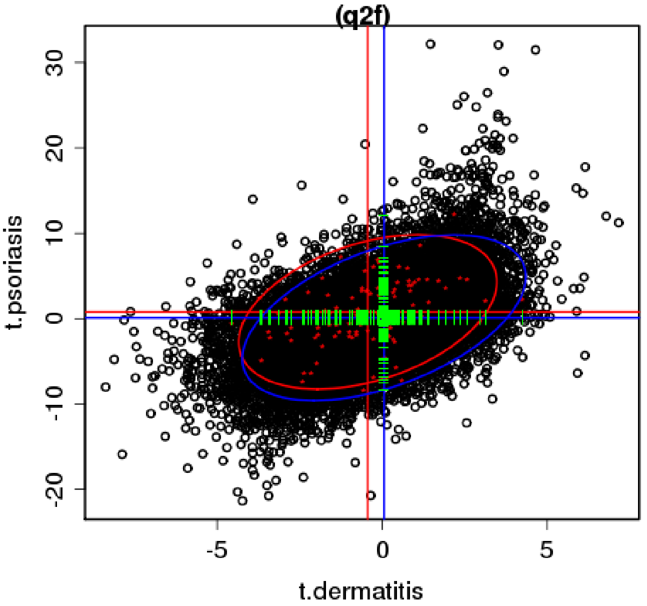

GO:0000398
nuclear mRNA splicing, via spliceosome
(q2f)

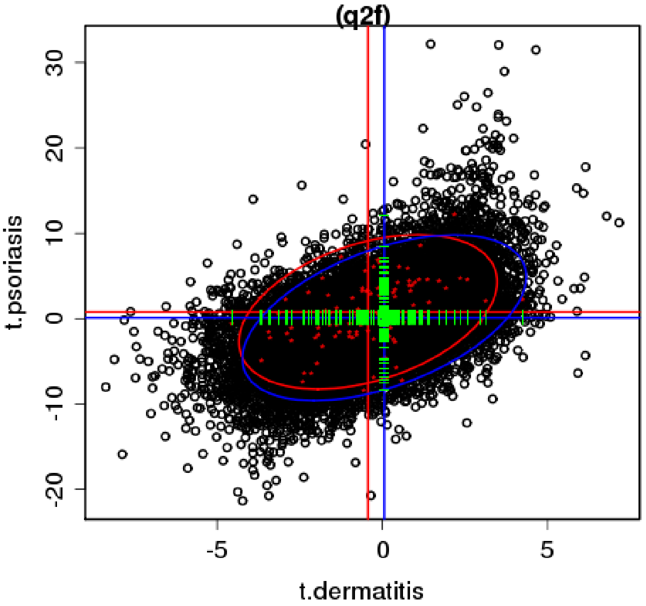

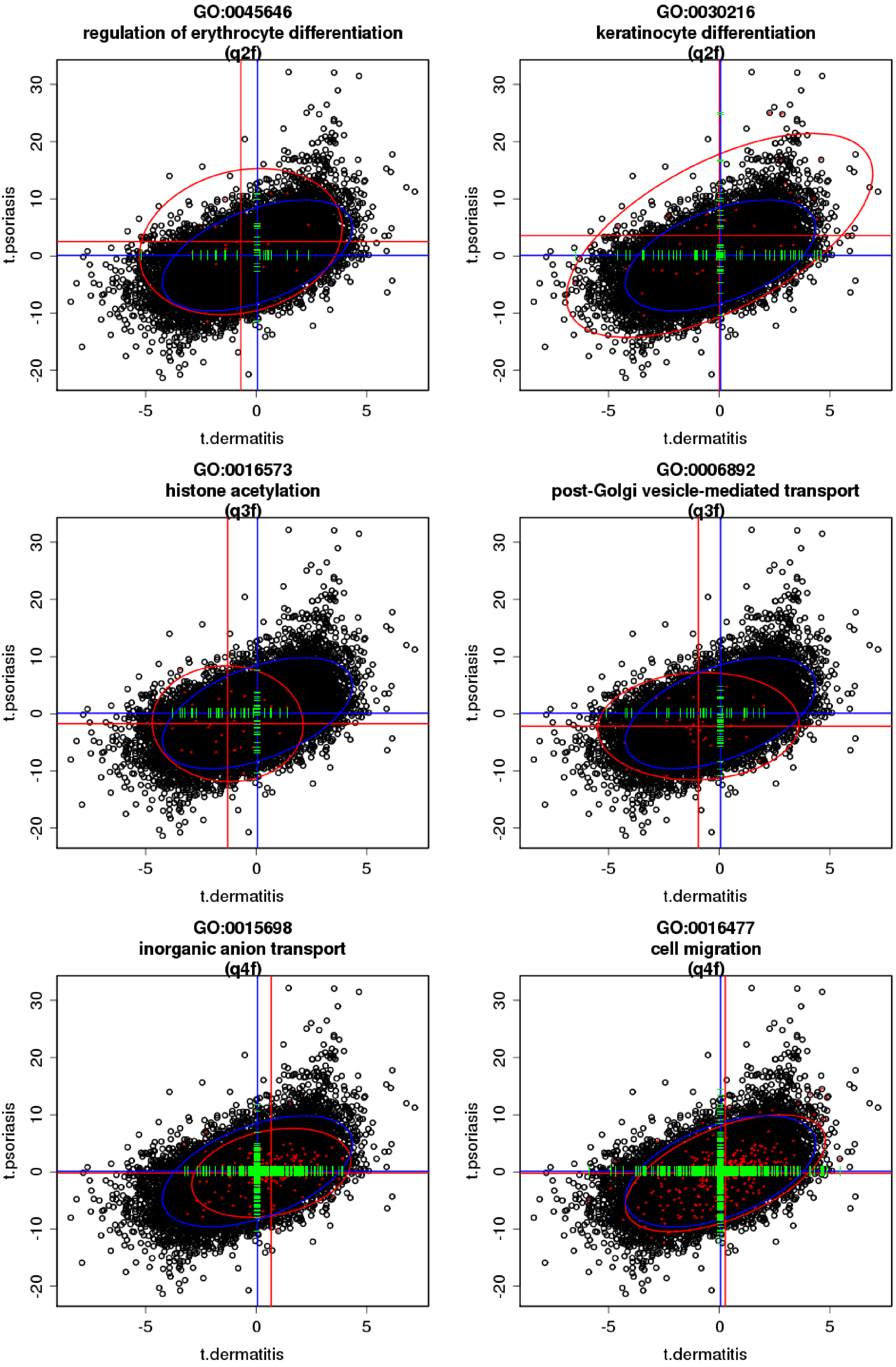
GO:0045646
regulation of erythrocyte differentiation
(q2f)
GO:0030216
keratinocyte differentiation
(q2f)
GO:0016573
histone acetylation
(q3f)
GO:0006892
post-Golgi vesicle-mediated transport
(q3f)
GO:0015698
inorganic anion transport
(q4f)
GO:0016477
cell migration
(q4f)
t.psoriasis
t.dermatitis
-20
-10
0
10
20
30
-5
0
5

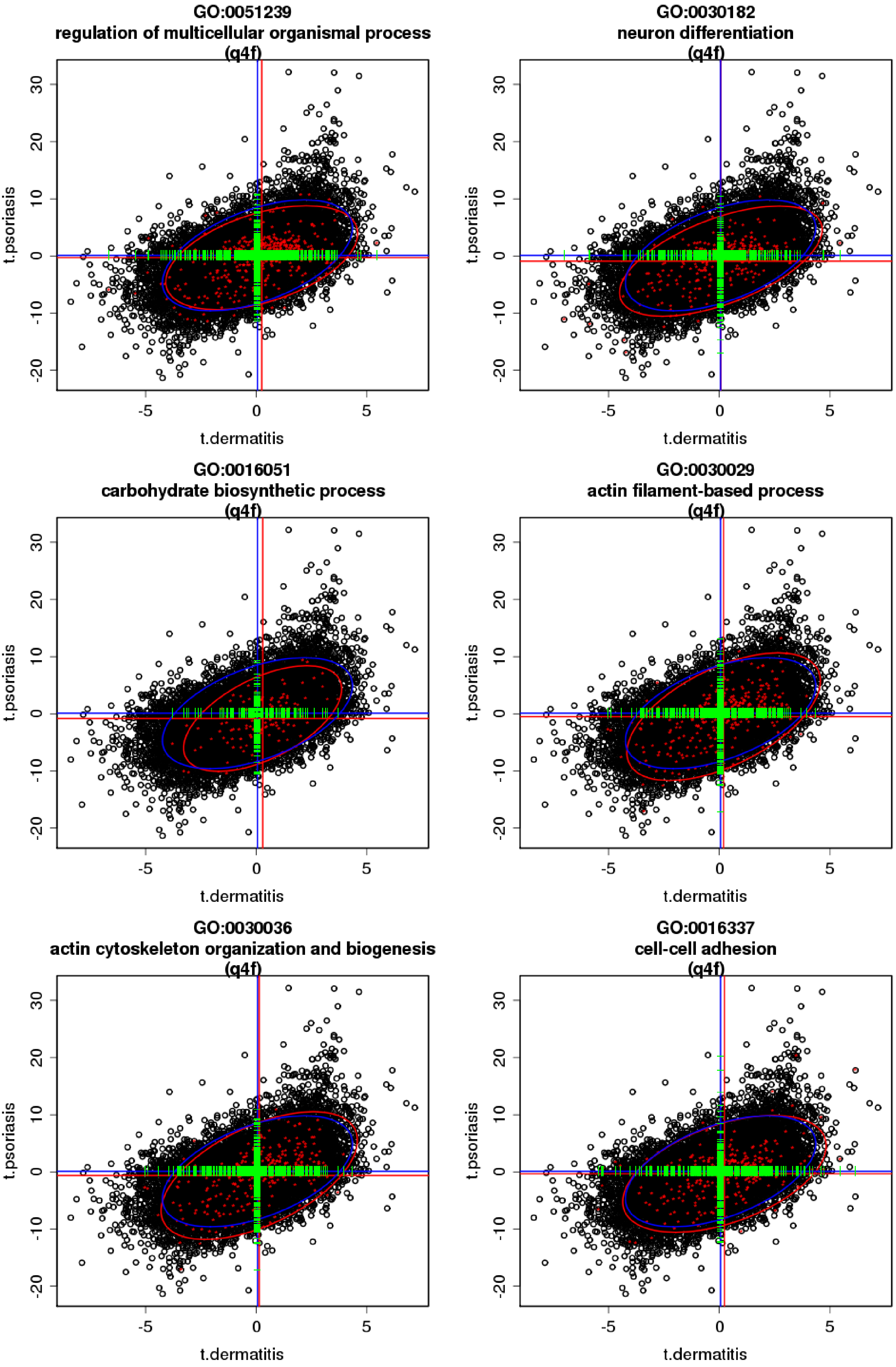
GO:0051239
regulation of multicellular organismal process
(q4f)
GO:0030182
neuron differentiation
(q4f)
GO:0016051
carbohydrate biosynthetic process
(q4f)
GO:0030029
actin filament-based process
(q4f)
GO:0030036
actin cytoskeleton organization and biogenesis
(q4f)
GO:0016337
cell-cell adhesion
(q4f)
t.psoriasis
t.dermatitis
-20
-10
0
10
20
30
-5
0
5

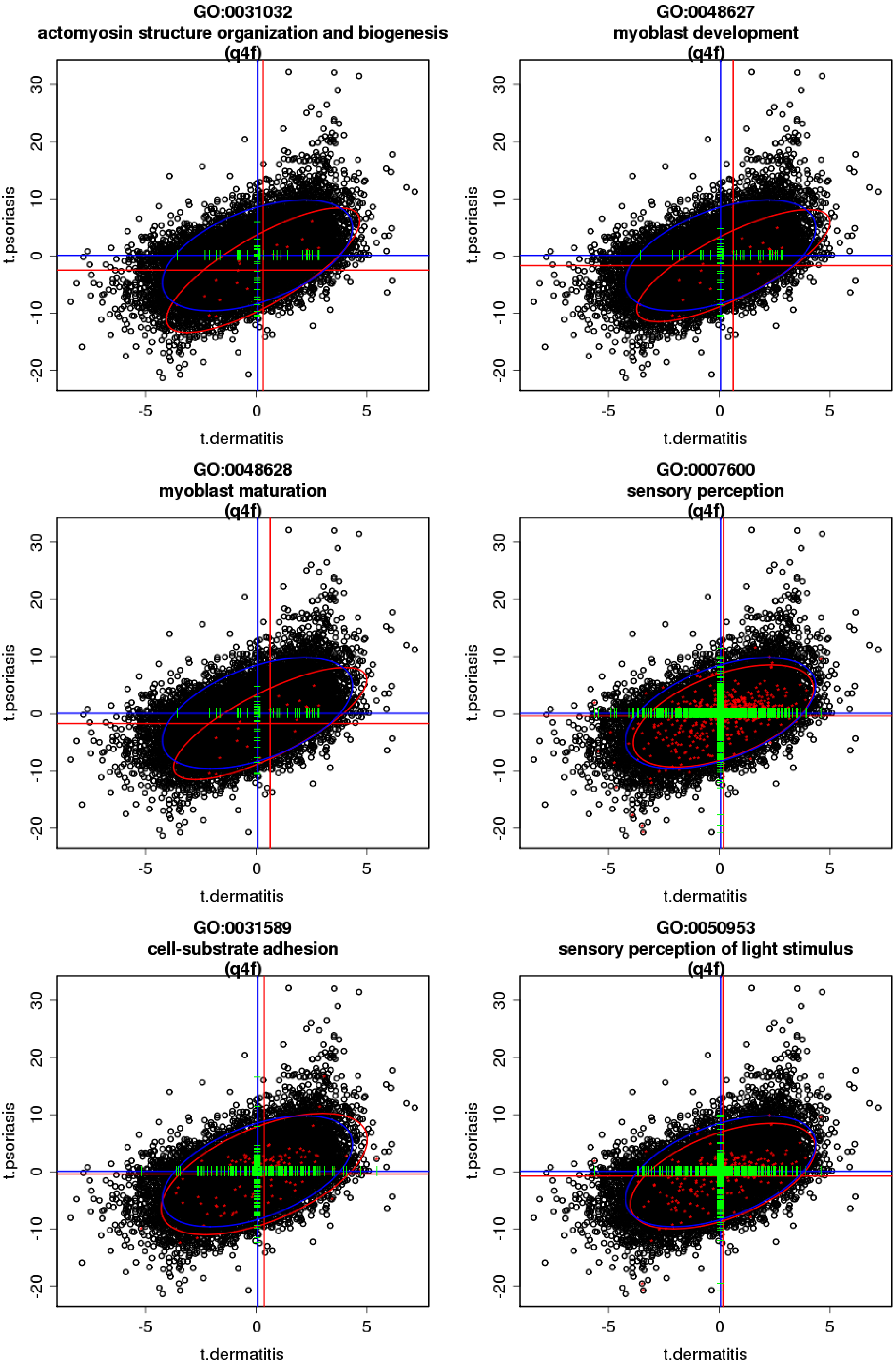
GO:0031032
actomyosin structure organization and biogenesis
(q4f)
GO:0048627
myoblast development
(q4f)
GO:0048628
myoblast maturation
(q4f)
GO:0007600
sensory perception
(q4f)
GO:0031589
cell-substrate adhesion
(q4f)
GO:0050953
sensory perception of light stimulus
(q4f)
t.psoriasis
t.dermatitis
-20
-10
0
10
20
30
-5
0
5

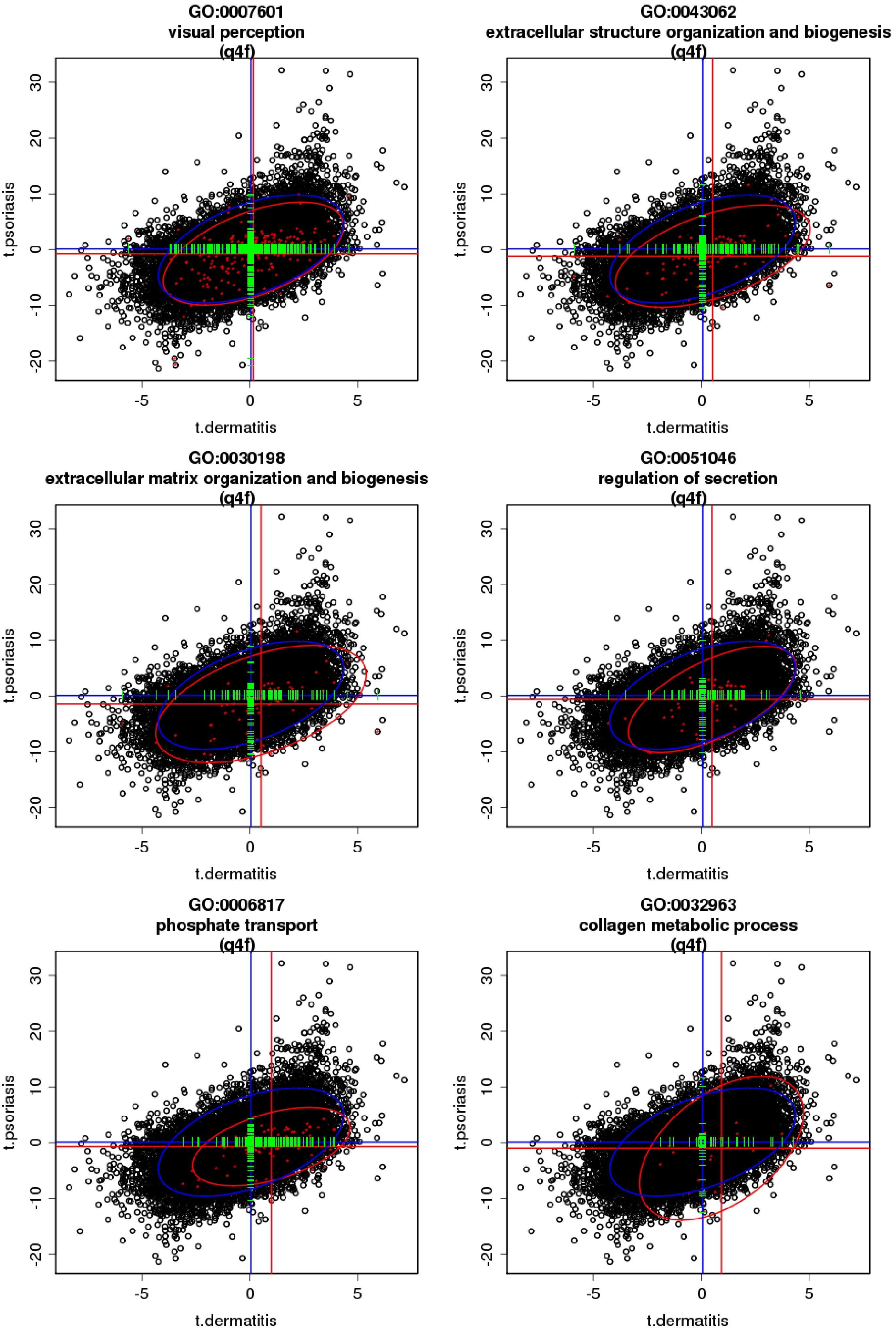

GO:0007601
visual perception
(q4f)
GO:0043062
extracellular structure organization and biogenesis
(q4f)
GO:0030198
extracellular matrix organization and biogenesis
(q4f)
GO:0051046
regulation of secretion
(q4f)
GO:0006817
phosphate transport
(q4f)
GO:0032963
collagen metabolic process
(q4f)
t.psoriasis
t.dermatitis
-20
-10
0
10
20
30
-5
0
5

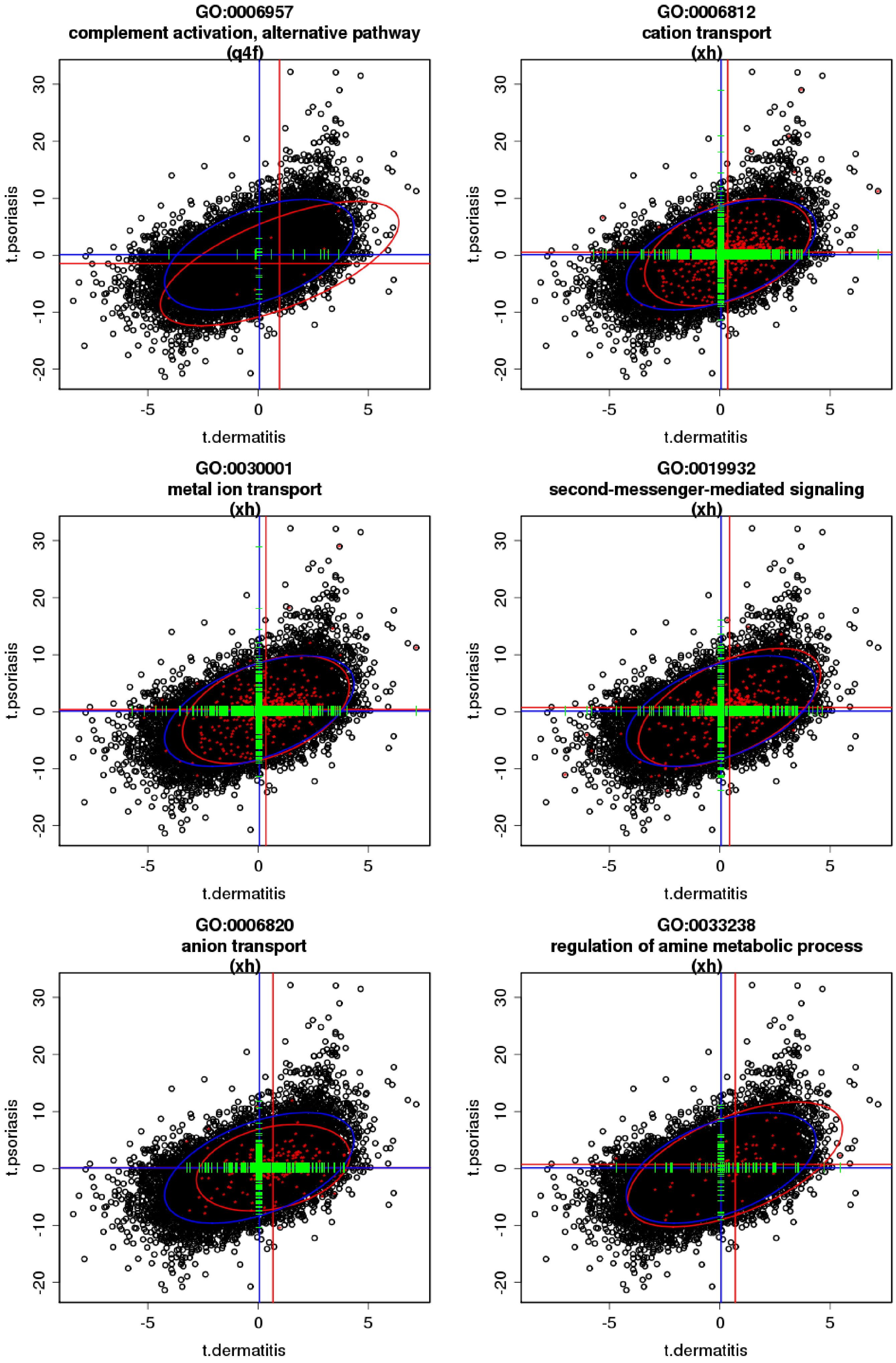
GO:0006957
complement activation, alternative pathway
(q4f)
t.psoriasis
t.dermatitis
GO:0006812
cation transport
(xh)
t.psoriasis
t.dermatitis
GO:0030001
metal ion transport
(xh)
t.psoriasis
t.dermatitis
GO:0019932
second-messenger-mediated signaling
(xh)
t.psoriasis
t.dermatitis
GO:0006820
anion transport
(xh)
t.psoriasis
t.dermatitis
GO:0033238
regulation of amine metabolic process
(xh)
t.psoriasis
t.dermatitis

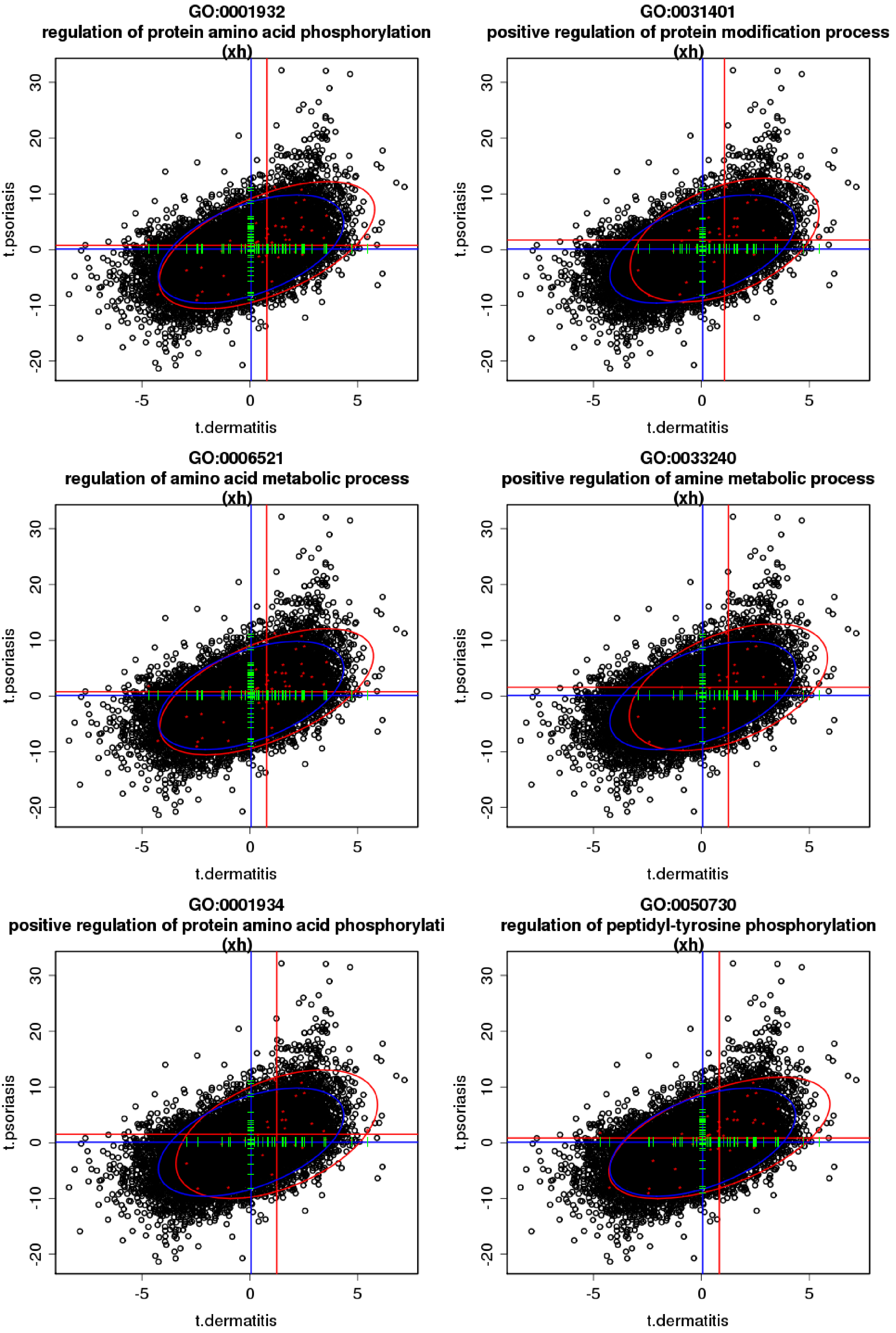
GO:0001932
regulation of protein amino acid phosphorylation
(xh)
t.psoriasis
t.dermatitis
GO:0031401
positive regulation of protein modification process
(xh)
t.psoriasis
t.dermatitis
GO:0006521
regulation of amino acid metabolic process
(xh)
t.psoriasis
t.dermatitis
GO:0033240
positive regulation of amine metabolic process
(xh)
t.psoriasis
t.dermatitis
GO:0001934
positive regulation of protein amino acid phosphorylati
(xh)
t.psoriasis
t.dermatitis
GO:0050730
regulation of peptidyl-tyrosine phosphorylation
(xh)
t.psoriasis
t.dermatitis

GO:0045764
positive regulation of amino acid metabolic process
(xh)

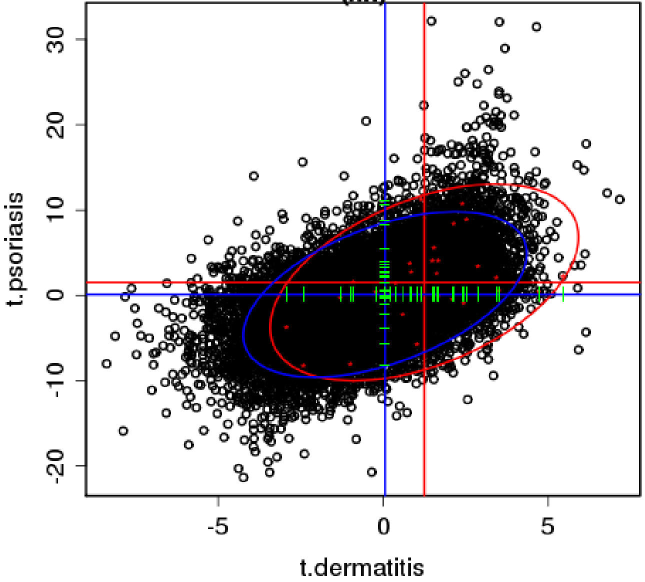

GO:0050731
positive regulation of peptidyl-tyrosine phosphorylatic
(xh)

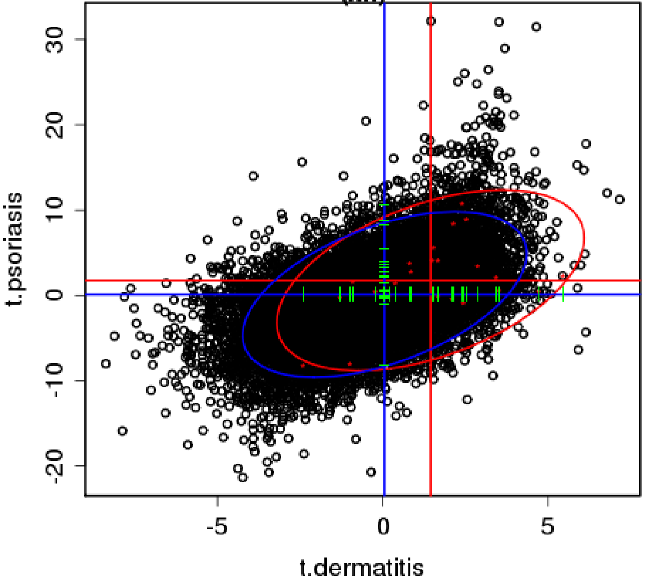

GO:0002682
regulation of immune system process
(xh)

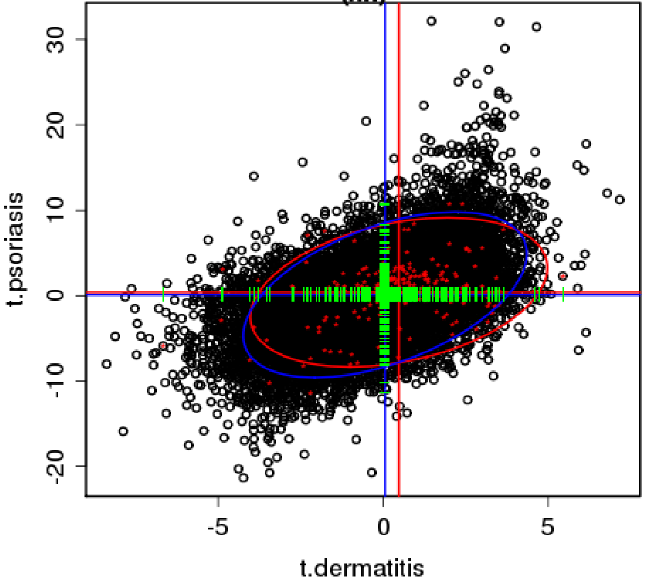

GO:0002684
positive regulation of immune system process
(xh)

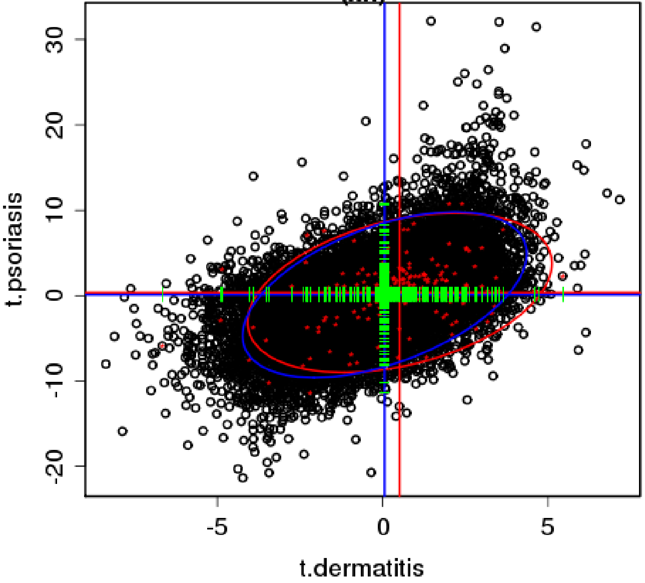

GO:0048583
regulation of response to stimulus
(xh)

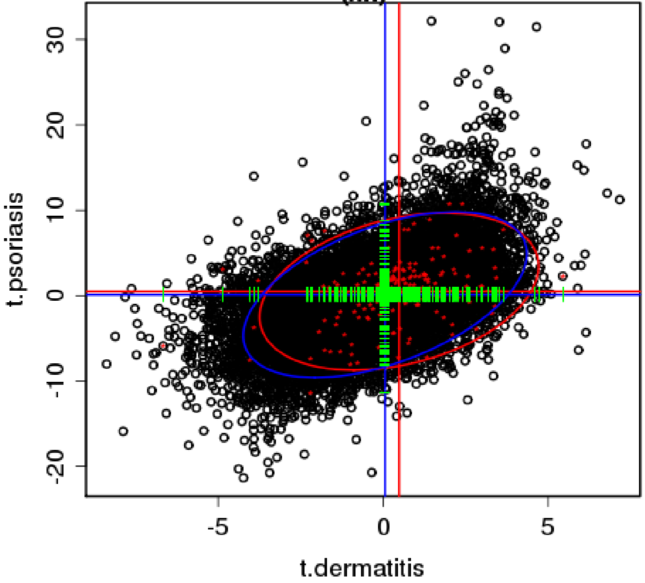

GO:0048584
positive regulation of response to stimulus
(xh)

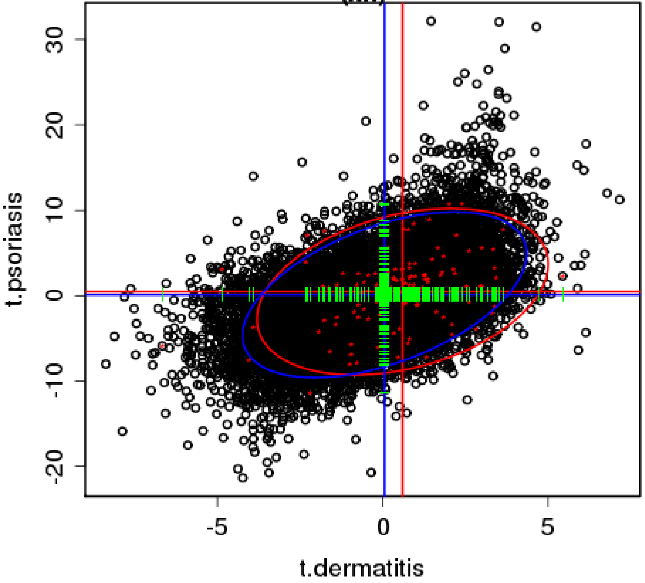

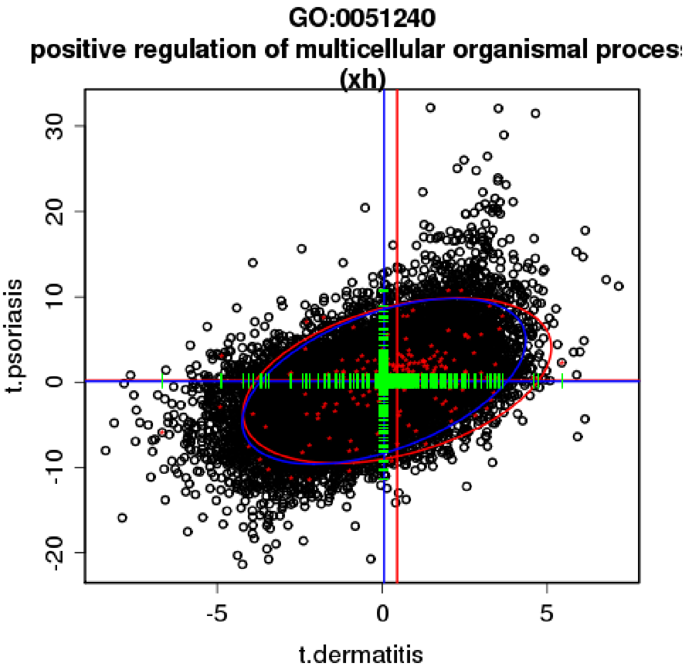
GO:0051240
positive regulation of multicellular organismal proces
(xh)
t.psoriasis
t.dermatitis
30
20
10
0
-10
-20
-5
0
5

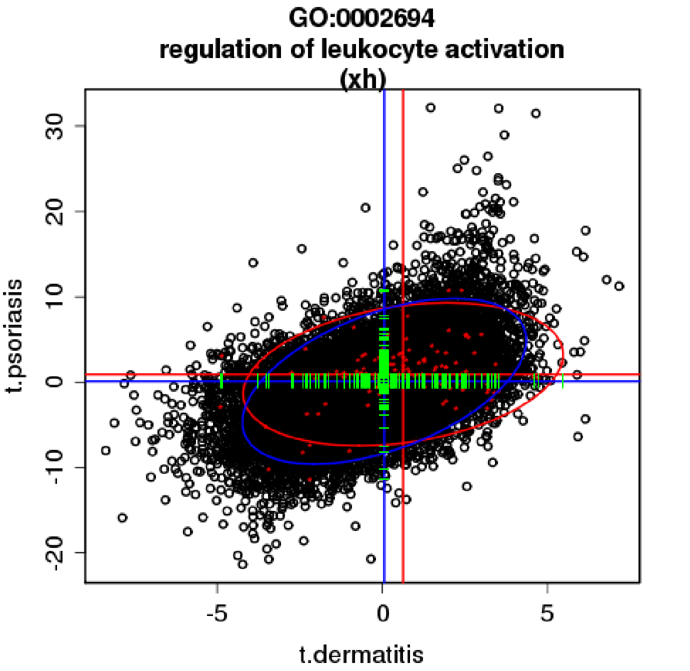
GO:0002694
regulation of leukocyte activation
(xh)
t.psoriasis
t.dermatitis
30
20
10
0
-10
-20
-5
0
5

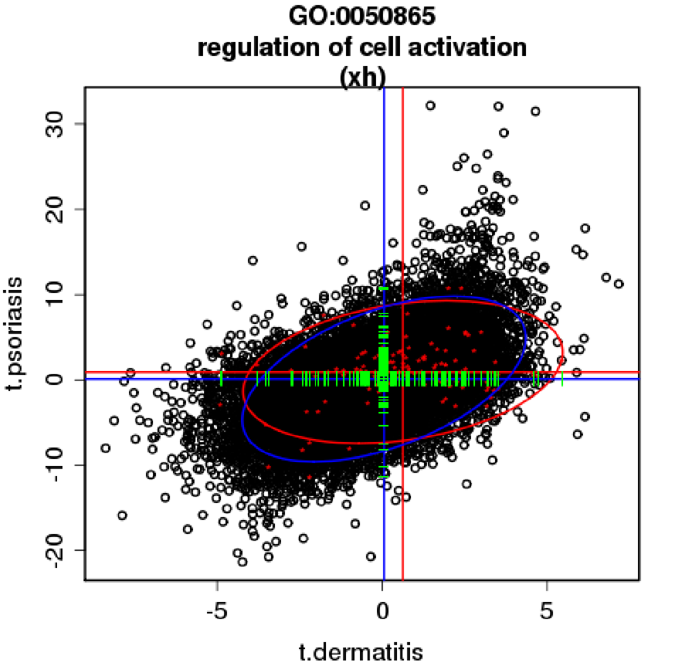
GO:0050865
regulation of cell activation
(xh)
t.psoriasis
t.dermatitis
30
20
10
0
-10
-20
-5
0
5

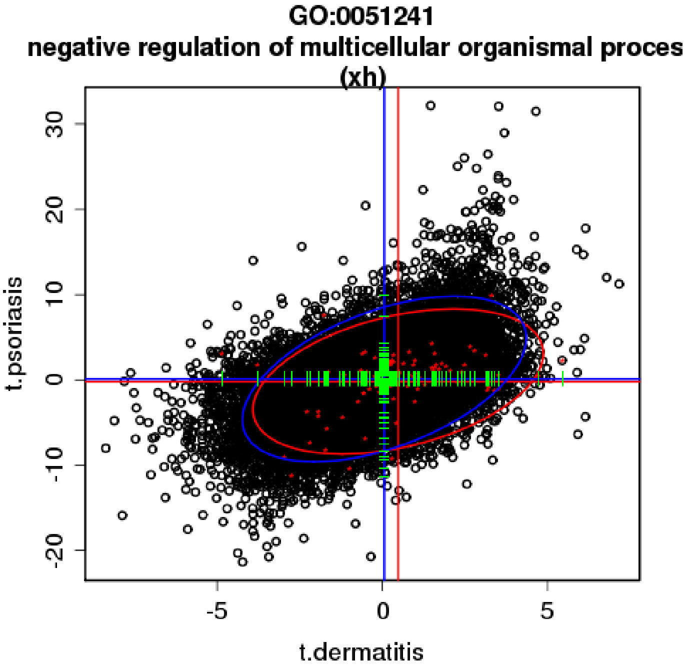
GO:0051241
negative regulation of multicellular organismal proces
(xh)
t.psoriasis
t.dermatitis
30
20
10
0
-10
-20
-5
0
5

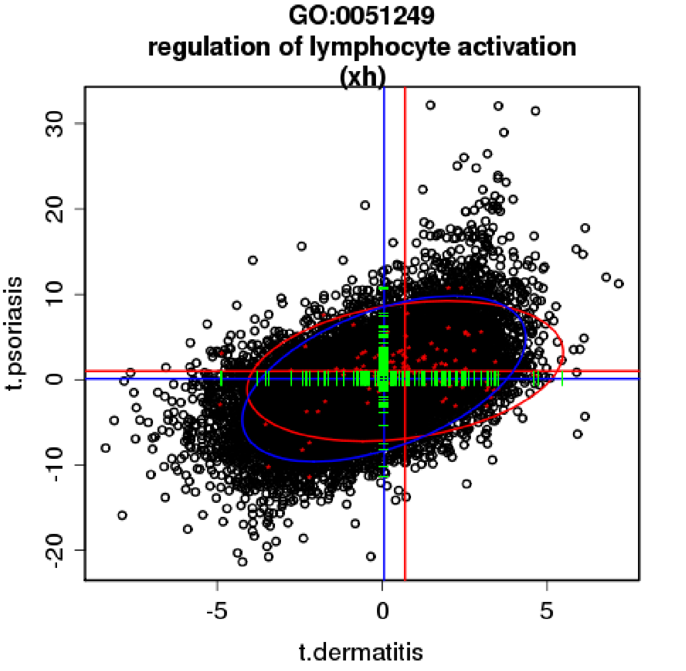
GO:0051249
regulation of lymphocyte activation
(xh)
t.psoriasis
t.dermatitis
30
20
10
0
-10
-20
-5
0
5

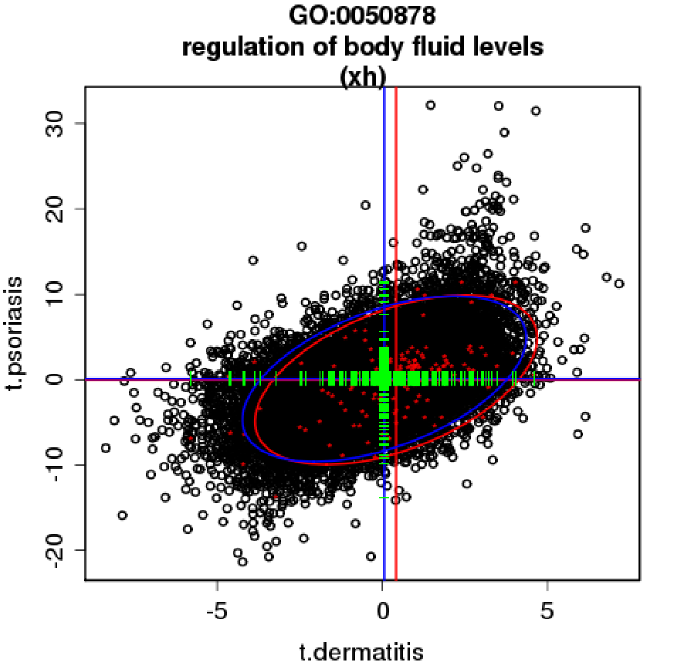
GO:0050878
regulation of body fluid levels
(xh)
t.psoriasis
t.dermatitis
30
20
10
0
-10
-20
-5
0
5

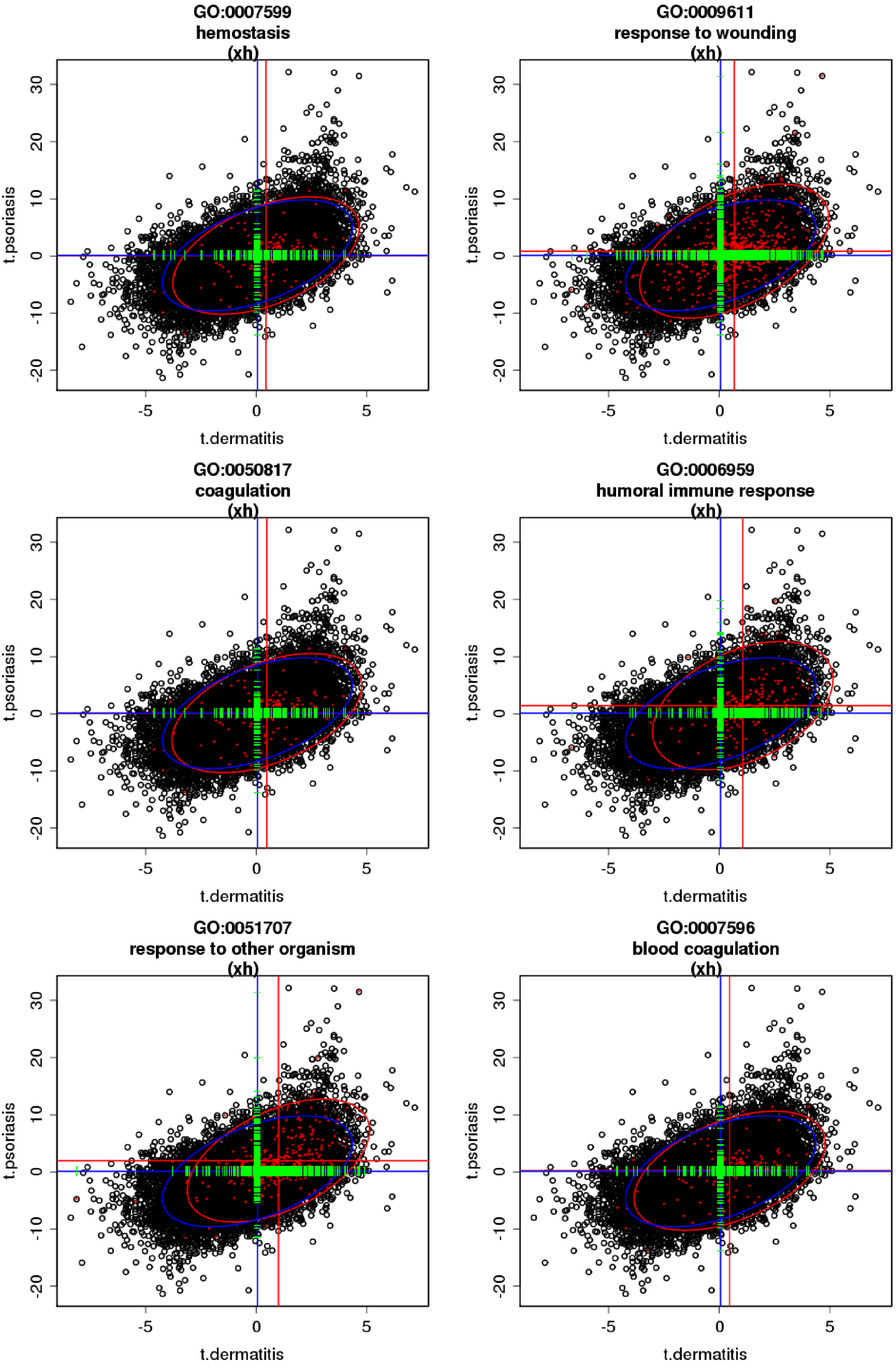
GO:0007599
hemostasis
(xh)
GO:0009611
response to wounding
(xh)
GO:0050817
coagulation
(xh)
GO:0006959
humoral immune response
(xh)
GO:0051707
response to other organism
(xh)
GO:0007596
blood coagulation
(xh)
t.psoriasis
t.dermatitis
-20
-10
0
10
20
30
-5
0
5

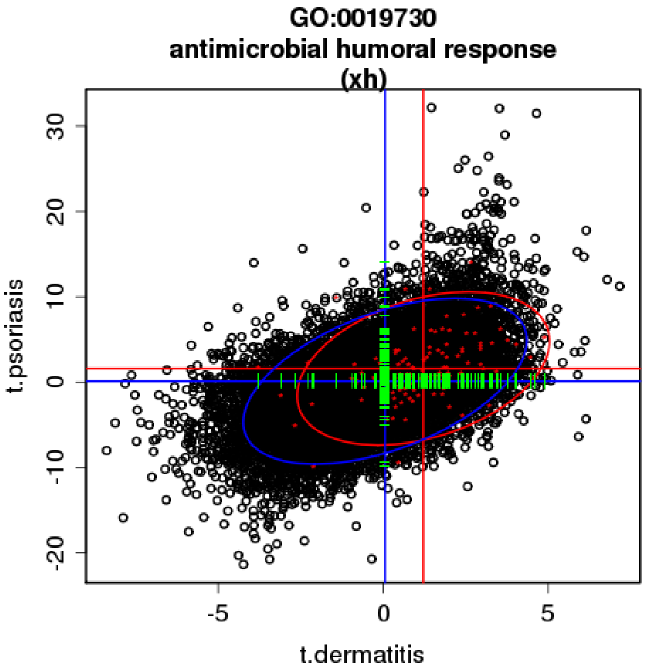
GO:0019730
antimicrobial humoral response
(xh)
t.psoriasis
30
20
10
0
-10
-20
-5
0
5
t.dermatitis

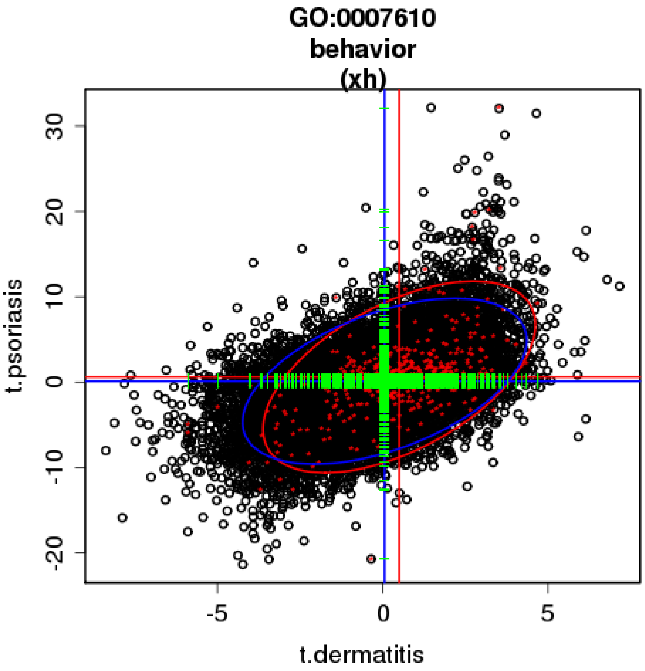
GO:0007610
behavior
(xh)
t.psoriasis
30
20
10
0
-10
-20
-5
0
5
t.dermatitis

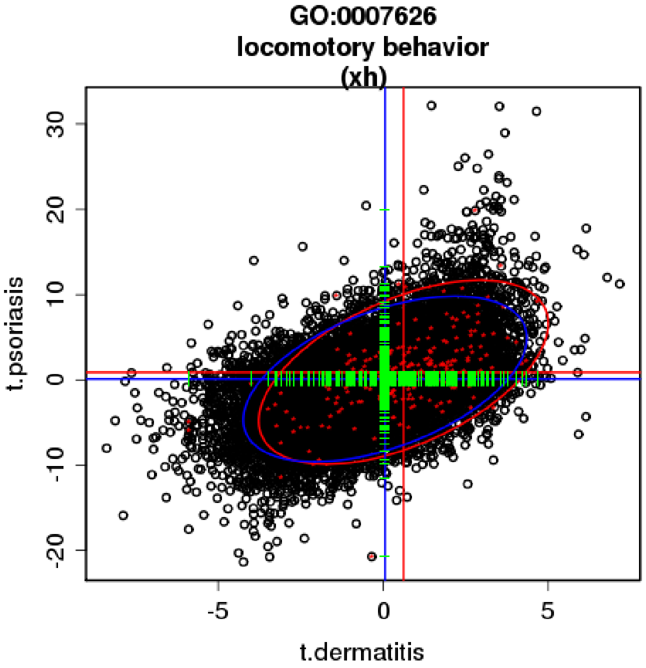
GO:0007626
locomotory behavior
(xh)
t.psoriasis
30
20
10
0
-10
-20
-5
0
5
t.dermatitis

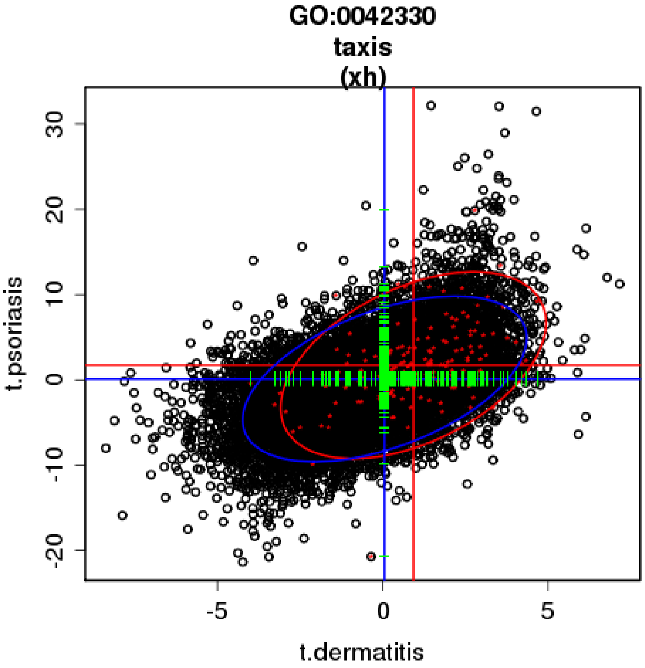
GO:0042330
taxis
(xh)
t.psoriasis
30
20
10
0
-10
-20
-5
0
5
t.dermatitis

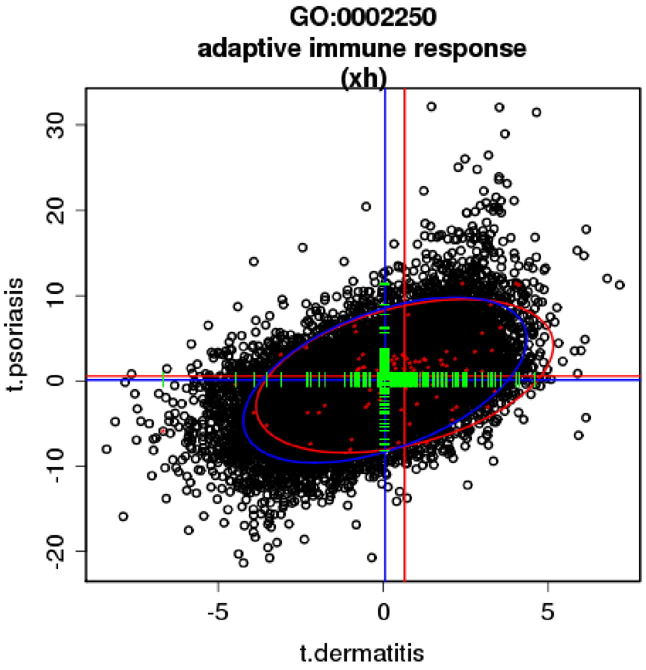
GO:0002250
adaptive immune response
(xh)
t.psoriasis
30
20
10
0
-10
-20
-5
0
5
t.dermatitis

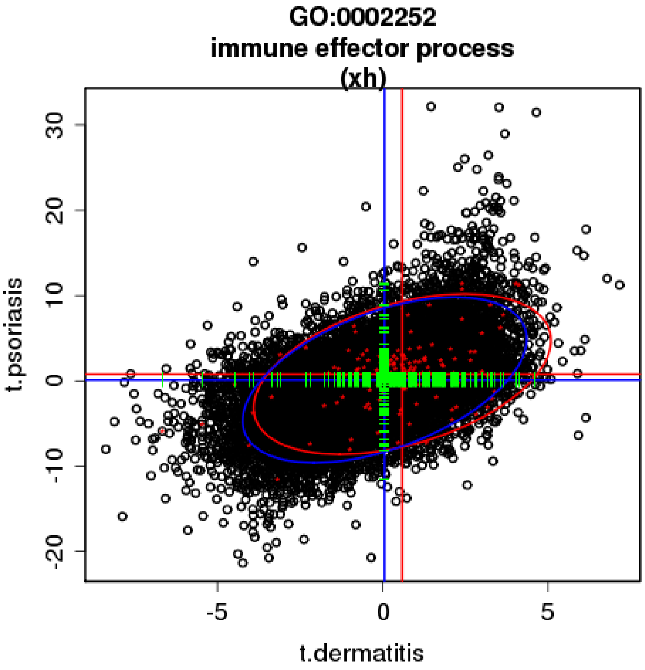
GO:0002252
immune effector process
(xh)
t.psoriasis
30
20
10
0
-10
-20
-5
0
5
t.dermatitis

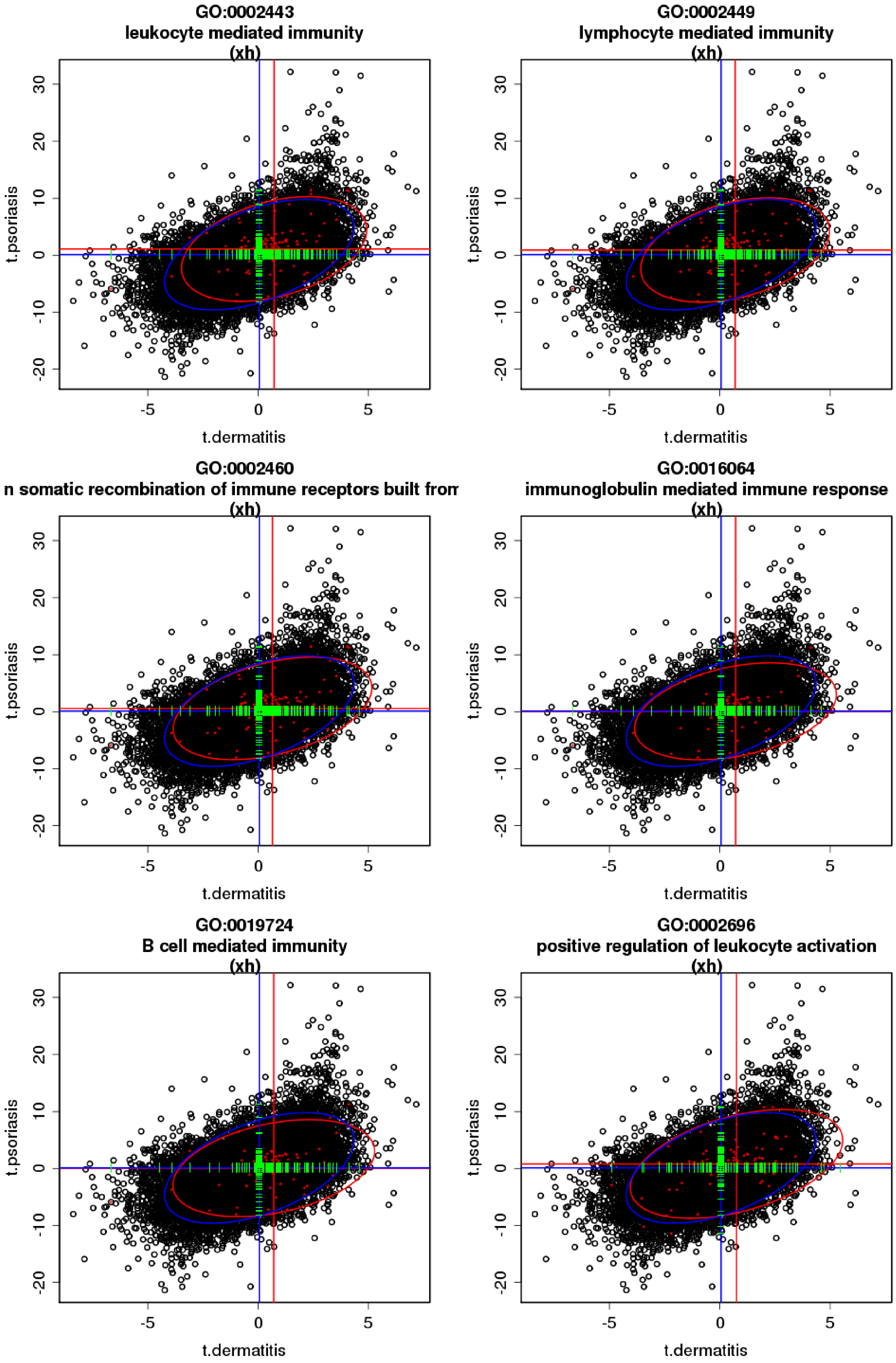
GO:0002443
leukocyte mediated immunity
(xh)
GO:0002449
lymphocyte mediated immunity
(xh)
GO:0002460
n somatic recombination of immune receptors built from
(xh)
GO:0016064
immunoglobulin mediated immune response
(xh)
GO:0019724
B cell mediated immunity
(xh)
GO:0002696
positive regulation of leukocyte activation
(xh)
t.psoriasis
t.dermatitis
-20
-10
0
10
20
30
-5
0
5

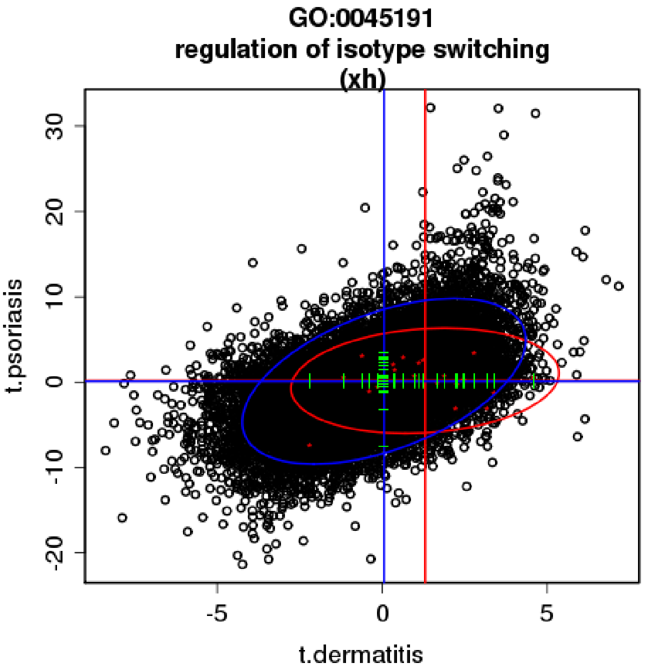
GO:0045191
regulation of isotype switching
(xh)
t.psoriasis
t.dermatitis

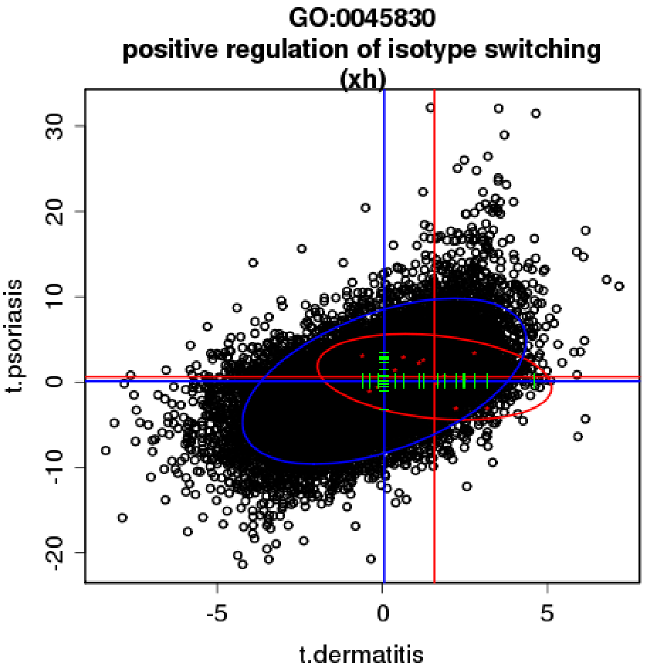
GO:0045830
positive regulation of isotype switching
(xh)
t.psoriasis
t.dermatitis

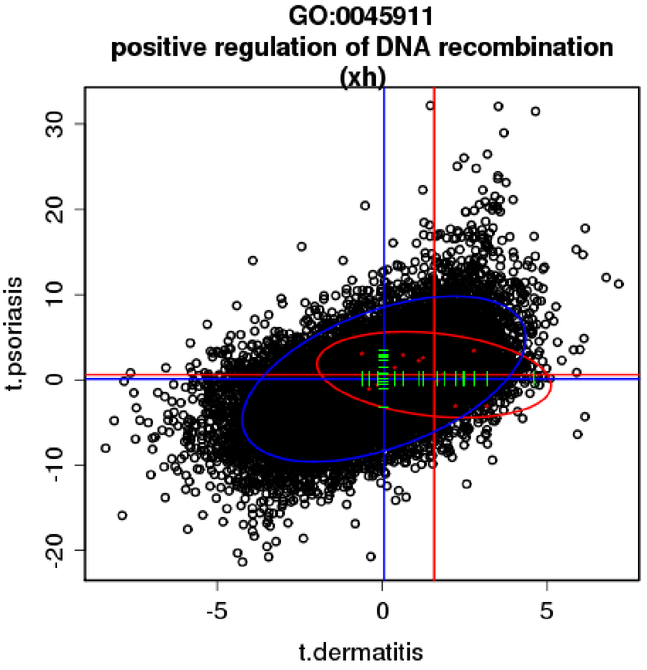
GO:0045911
positive regulation of DNA recombination
(xh)
t.psoriasis
t.dermatitis

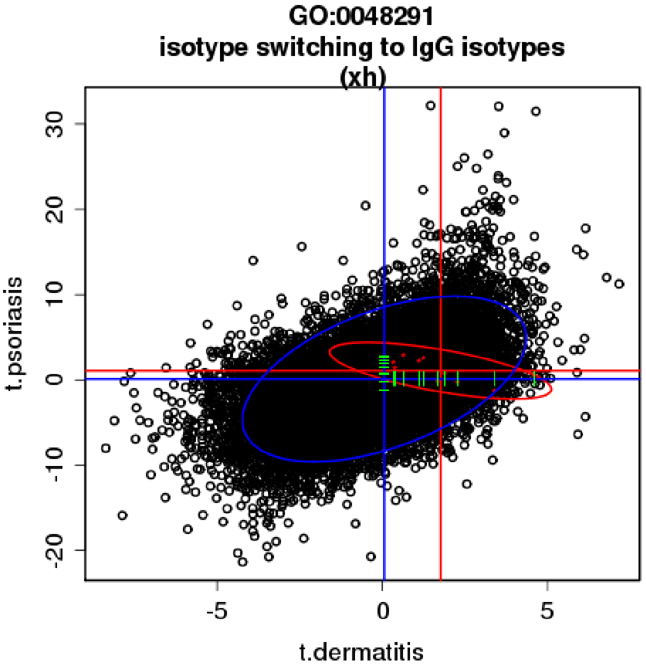
GO:0048291
isotype switching to IgG isotypes
(xh)
t.psoriasis
t.dermatitis

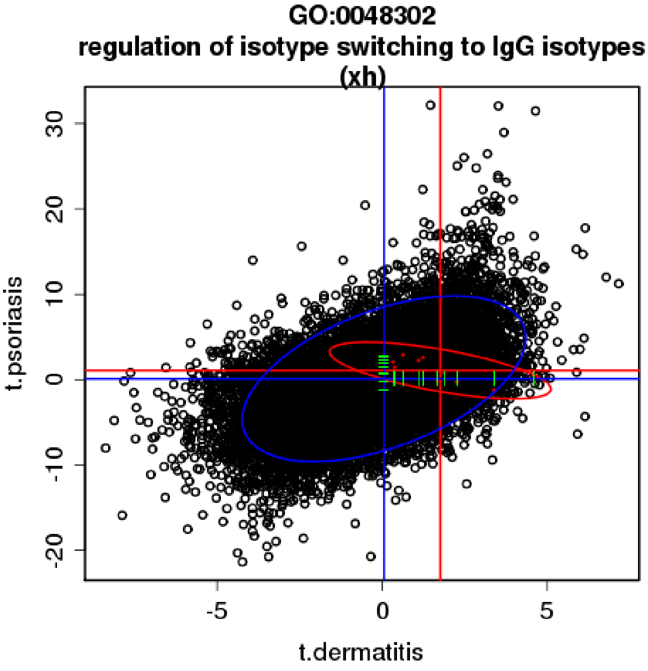
GO:0048302
regulation of isotype switching to IgG isotypes
(xh)
t.psoriasis
t.dermatitis

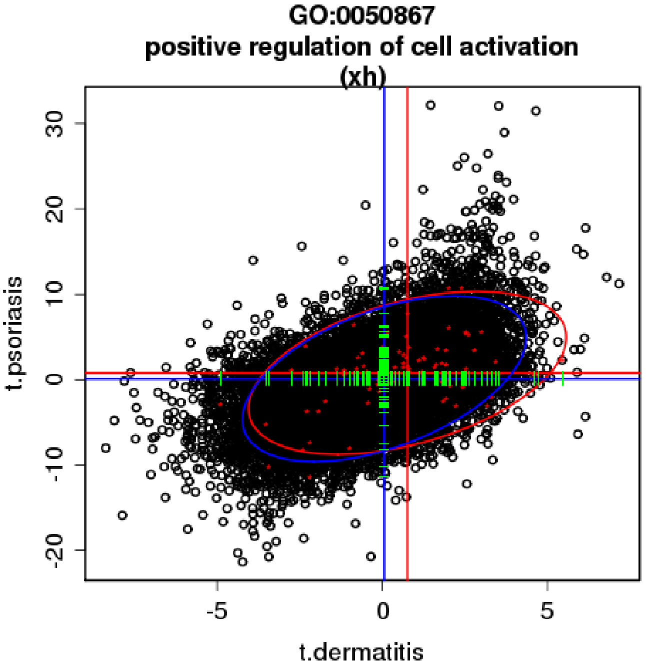
GO:0050867
positive regulation of cell activation
(xh)
t.psoriasis
t.dermatitis

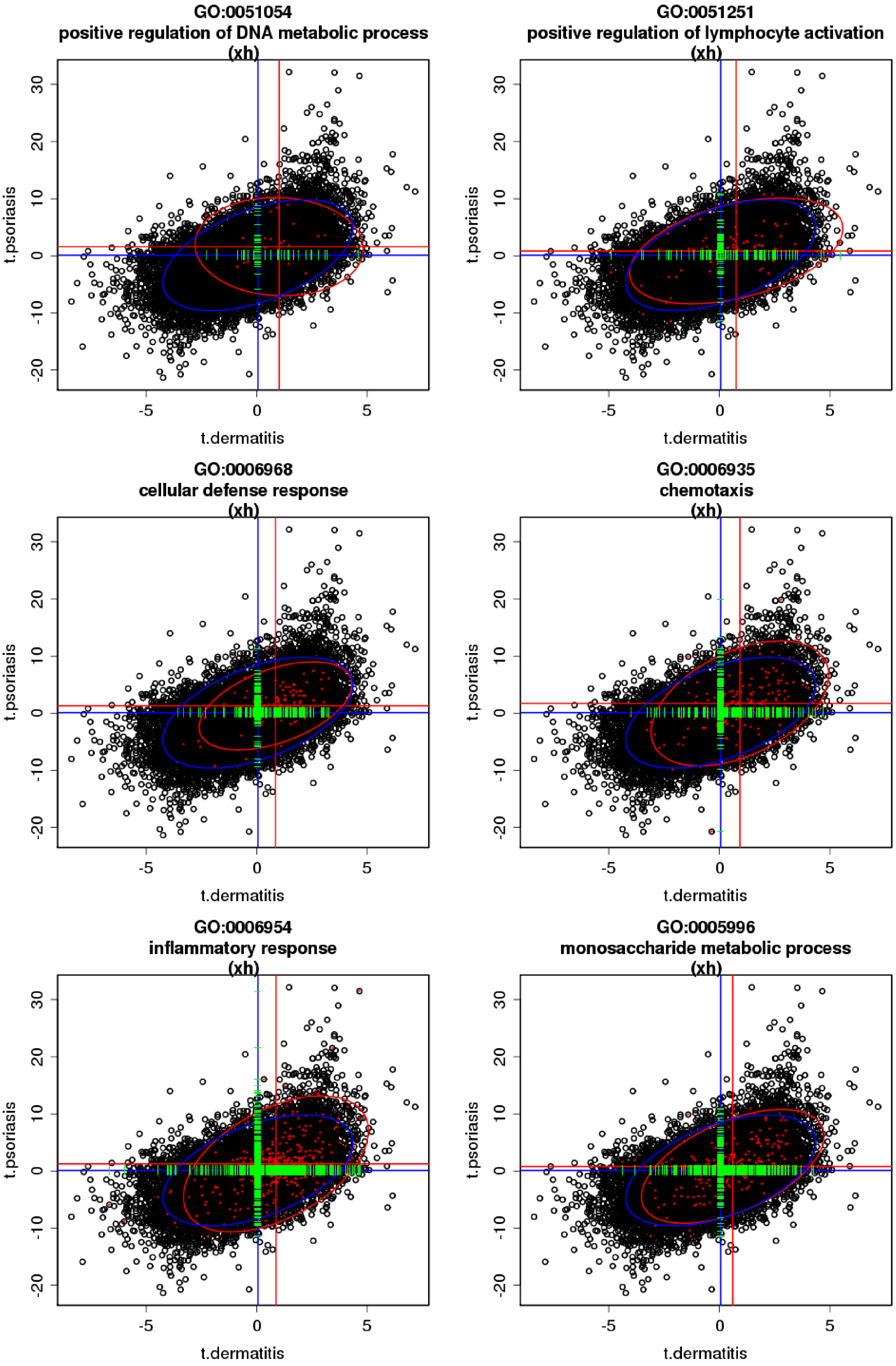

GO:0051054
positive regulation of DNA metabolic process
(xh)
GO:0051251
positive regulation of lymphocyte activation
(xh)
GO:0006968
cellular defense response
(xh)
GO:0006935
chemotaxis
(xh)
GO:0006954
inflammatory response
(xh)
GO:0005996
monosaccharide metabolic process
(xh)
t.psoriasis
t.dermatitis
-20
-10
0
10
20
30
-5
0
5

GO:0044262
cellular carbohydrate metabolic process
(xh)

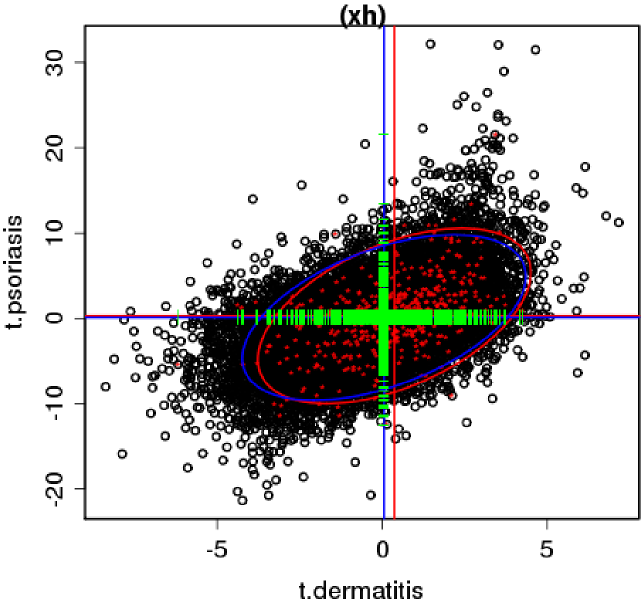

GO:0015980
energy derivation by oxidation of organic compounds
(xh)

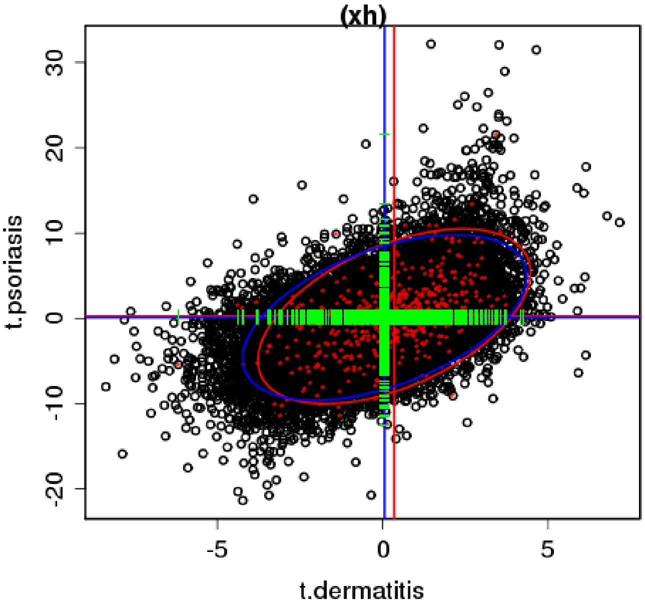

GO:0019318
hexose metabolic process
(xh)

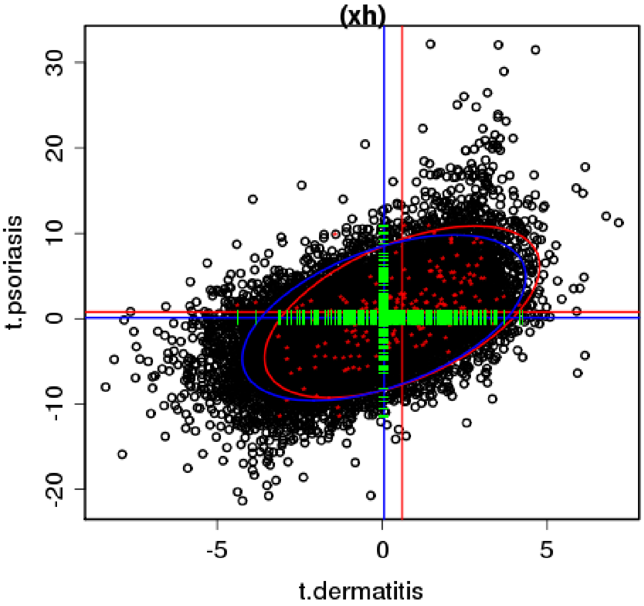

GO:0006006
glucose metabolic process
(xh)

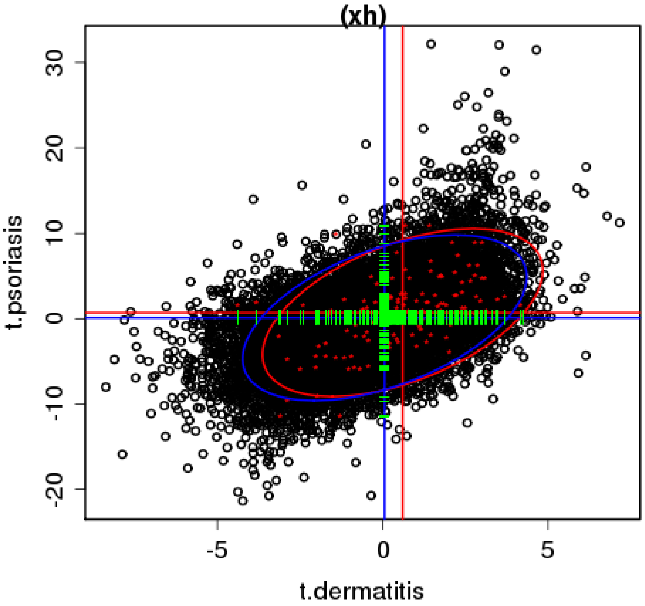

GO:0030003
cellular cation homeostasis
(xh)

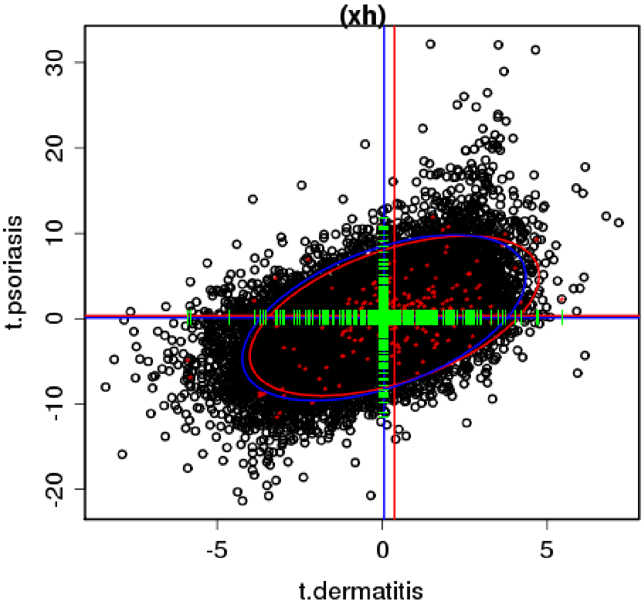

GO:0055065
metal ion homeostasis
(xh)

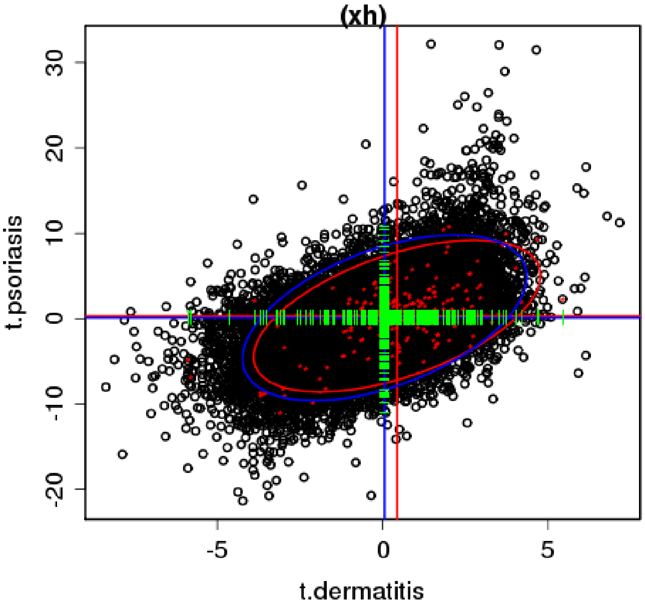

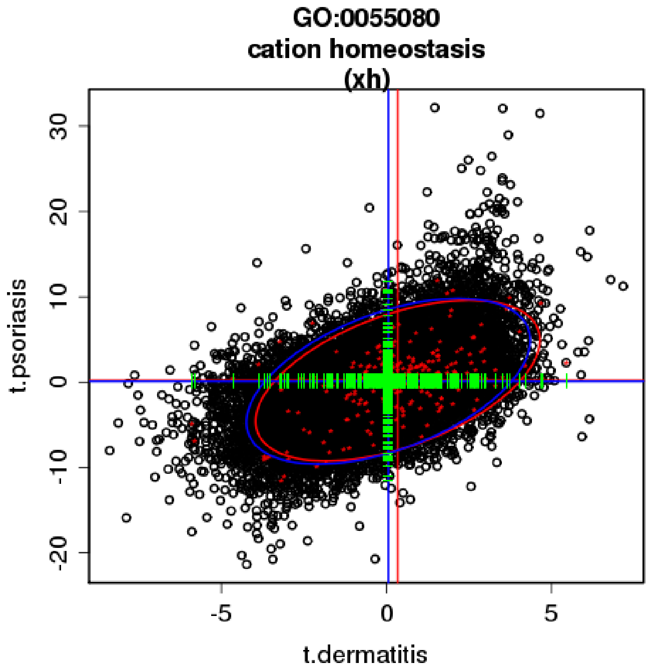
GO:0055080
cation homeostasis
(xh)
t.psoriasis
t.dermatitis
-20
-10
0
10
20
30
-5
0
5

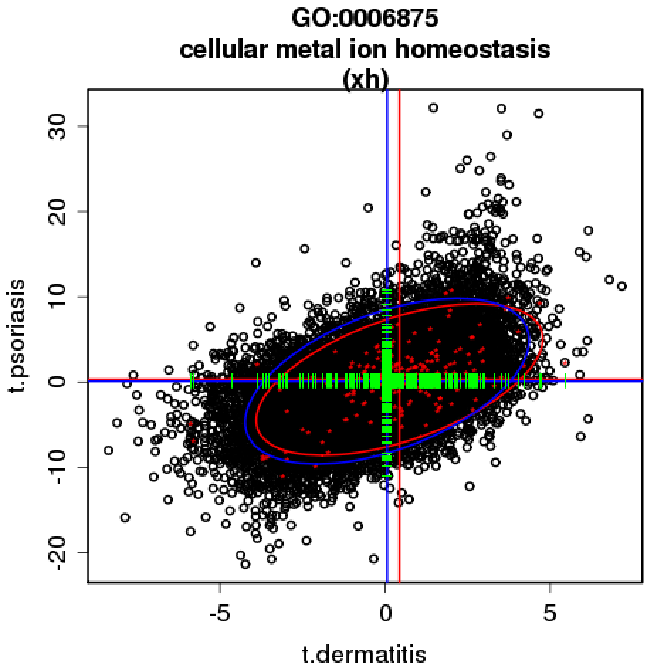
GO:0006875
cellular metal ion homeostasis
(xh)
t.psoriasis
t.dermatitis
-20
-10
0
10
20
30
-5
0
5

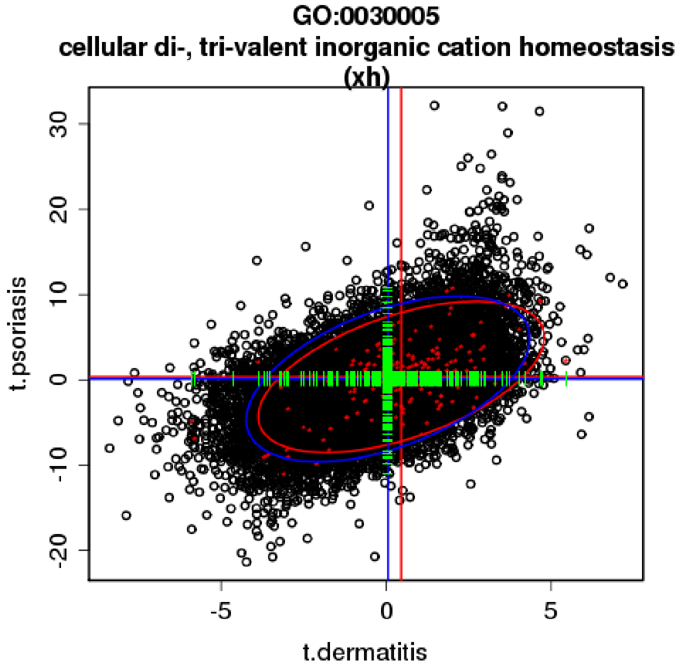
GO:0030005
cellular di-, tri-valent inorganic cation homeostasis
(xh)
t.psoriasis
t.dermatitis
-20
-10
0
10
20
30
-5
0
5

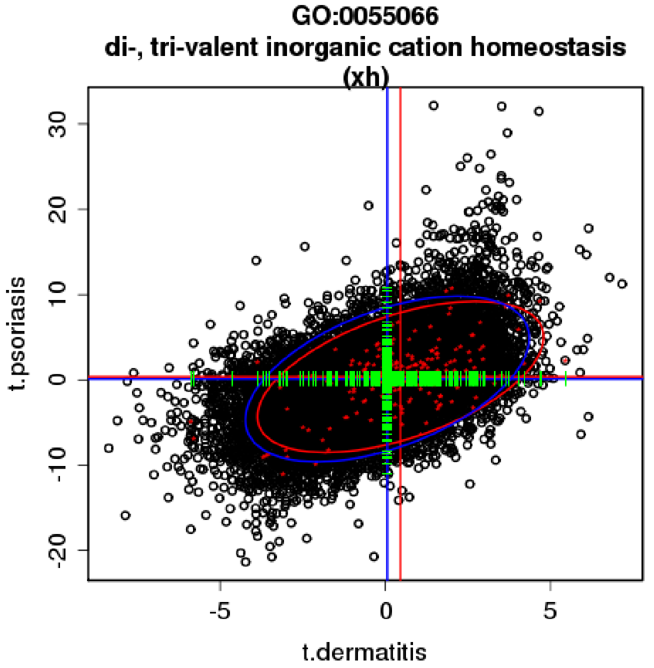
GO:0055066
di-, tri-valent inorganic cation homeostasis
(xh)
t.psoriasis
t.dermatitis
-20
-10
0
10
20
30
-5
0
5

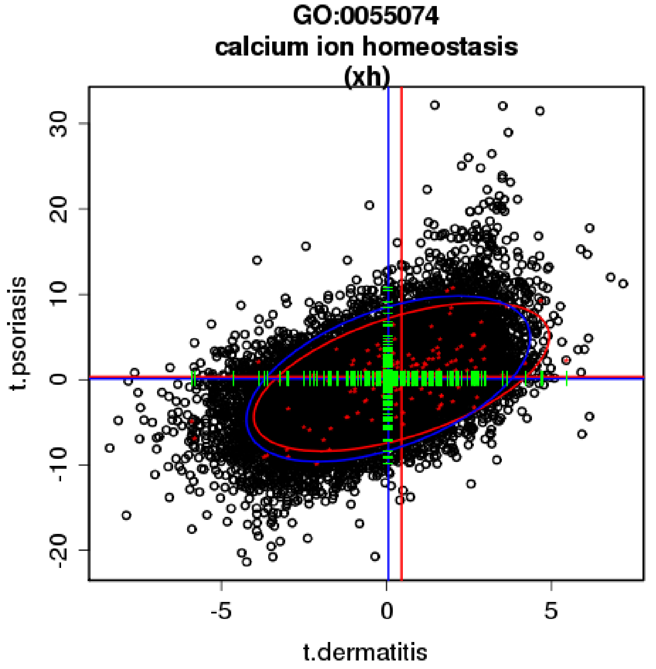
GO:0055074
calcium ion homeostasis
(xh)
t.psoriasis
t.dermatitis
-20
-10
0
10
20
30
-5
0
5

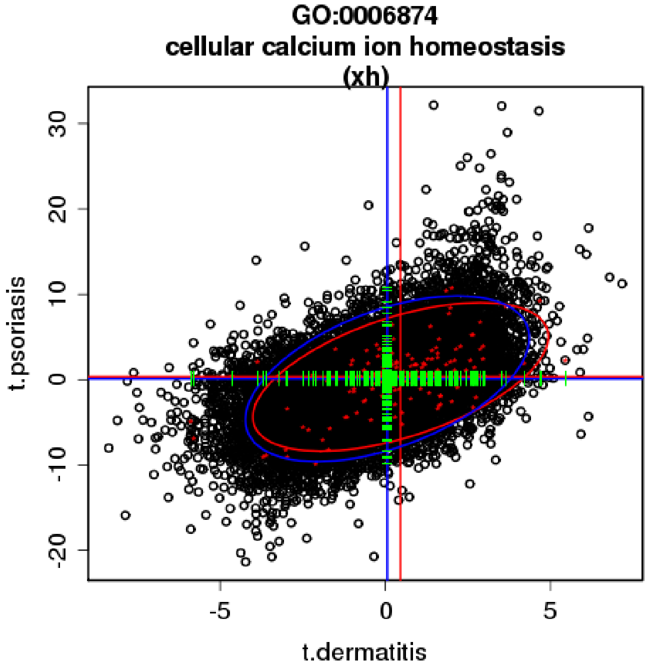
GO:0006874
cellular calcium ion homeostasis
(xh)
t.psoriasis
t.dermatitis
-20
-10
0
10
20
30
-5
0
5

GO:0045214
sarcomere organization
(xh)

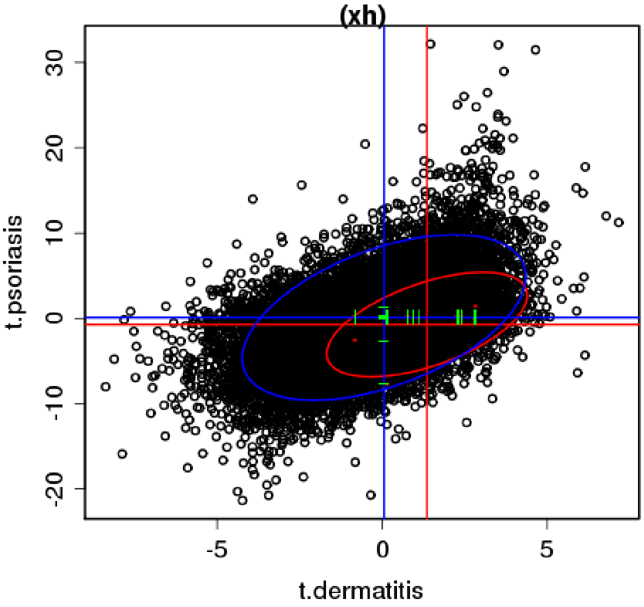

GO:0048646
anatomical structure formation
(xh)

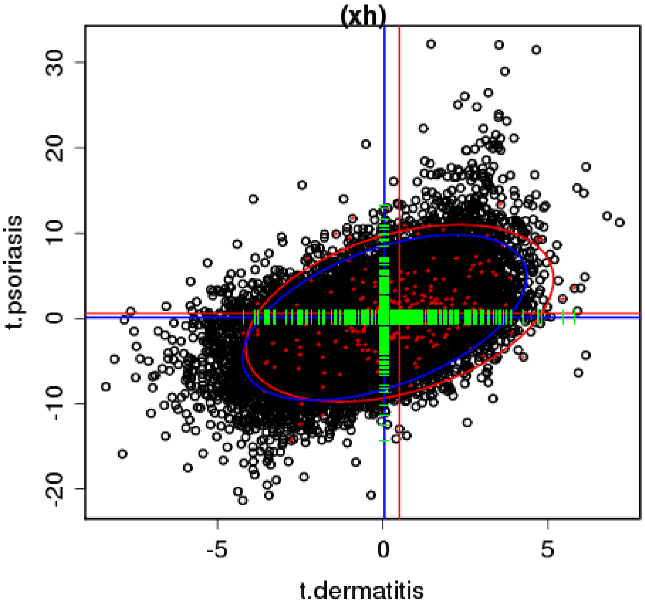

GO:0007160
cell-matrix adhesion
(xh)

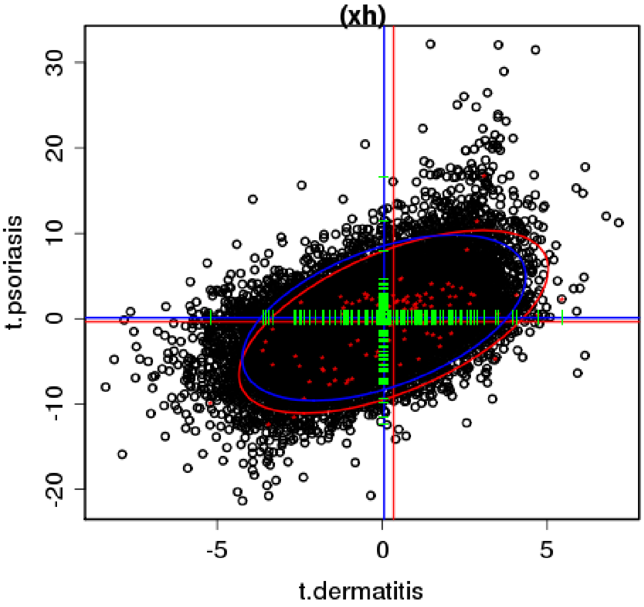

GO:0051480
cytosolic calcium ion homeostasis
(xh)

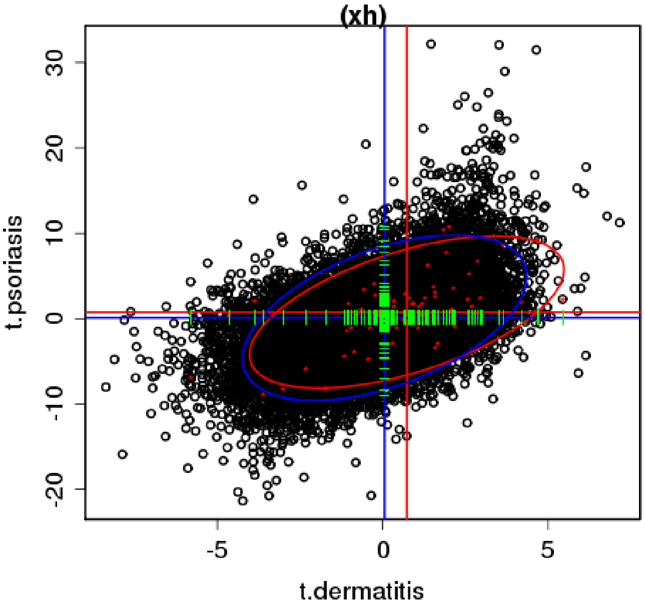

GO:0007204
elevation of cytosolic calcium ion concentration
(xh)

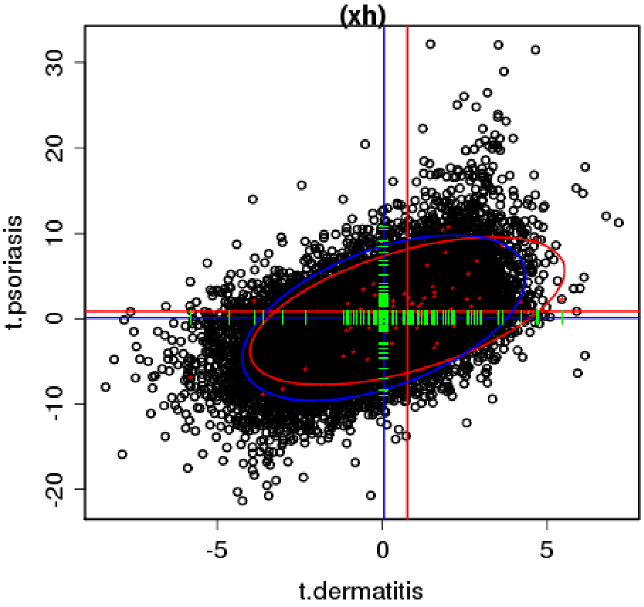

GO:0032103
positive regulation of response to external stimulus
(xh)

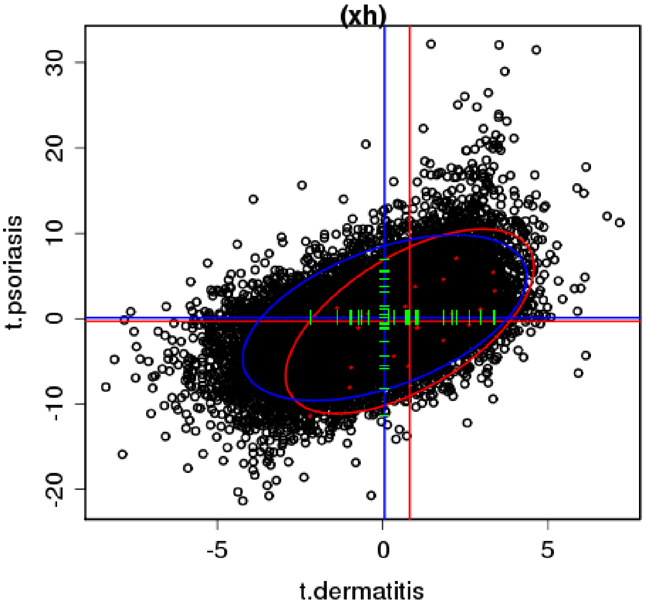

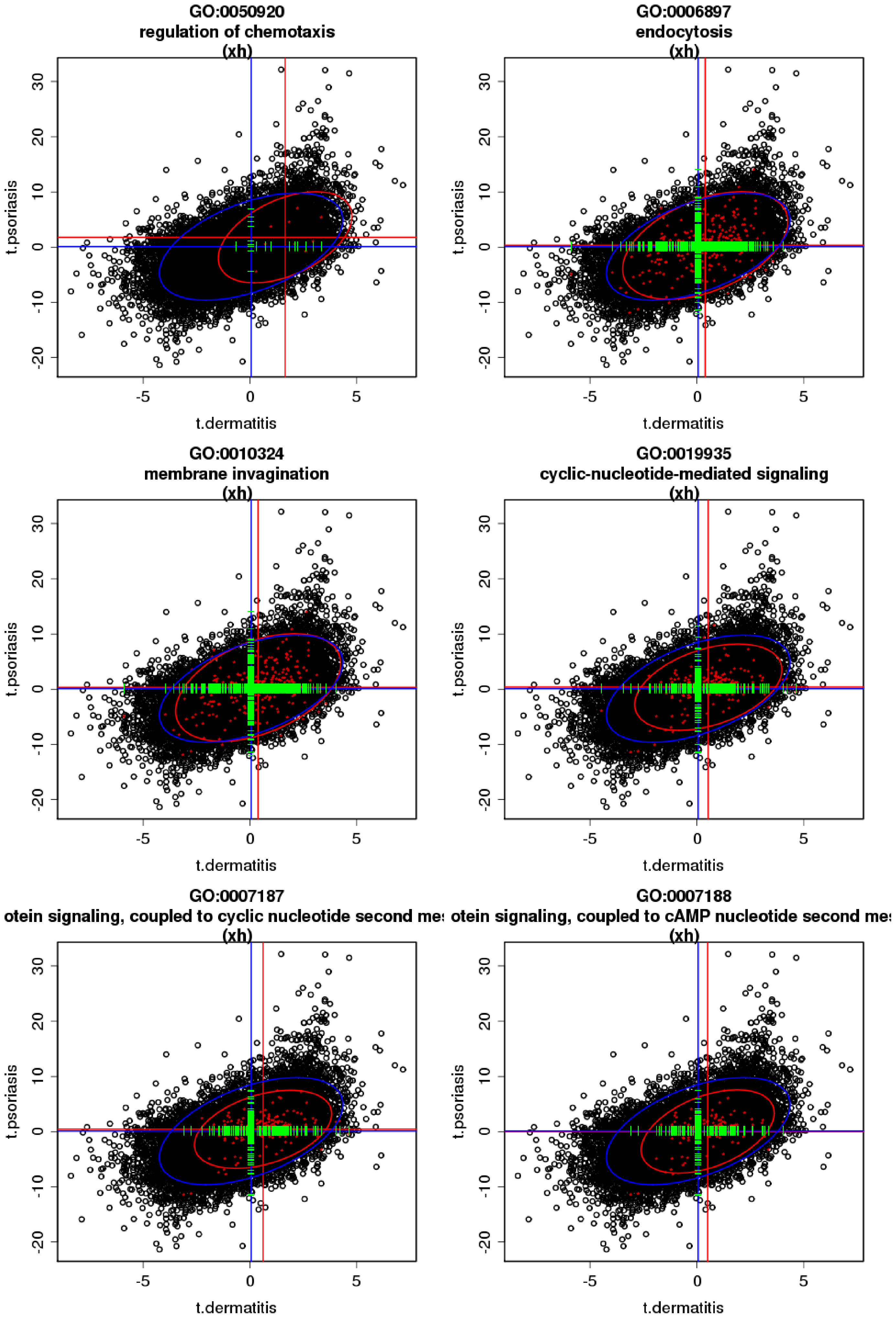
GO:0050920
regulation of chemotaxis
(xh)
GO:0006897
endocytosis
(xh)
GO:0010324
membrane invagination
(xh)
GO:0019935
cyclic-nucleotide-mediated signaling
(xh)
GO:0007187
otein signaling, coupled to cyclic nucleotide second me:
(xh)
GO:0007188
otein signaling, coupled to cAMP nucleotide second me:
(xh)
t.psoriasis
t.dermatitis
30
20
10
0
-10
-20
-5
0
5

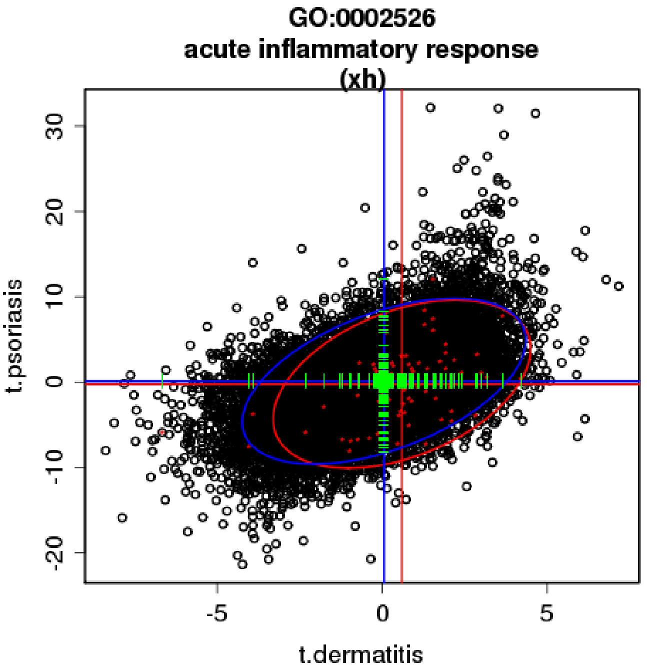
GO:0002526
acute inflammatory response
(xh)
t.psoriasis
t.dermatitis
-20
-10
0
10
20
30
-5
0
5

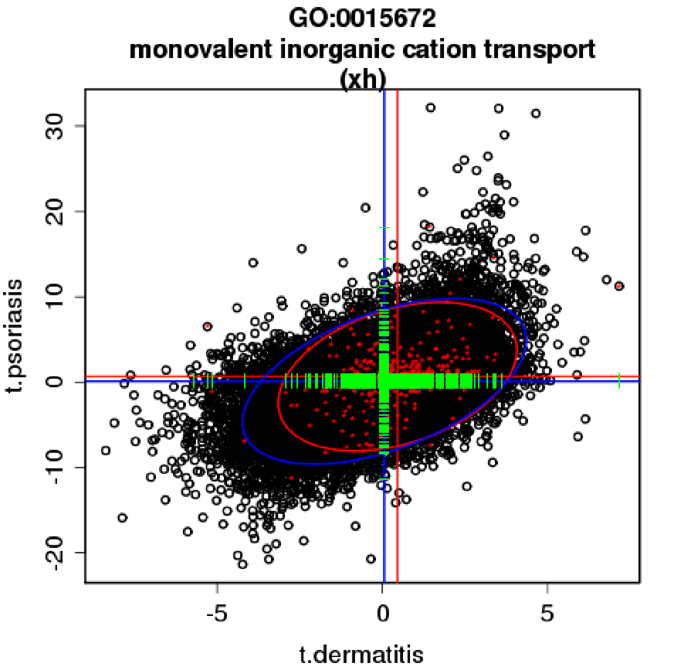
GO:0015672
monovalent inorganic cation transport
(xh)
t.psoriasis
t.dermatitis
-20
-10
0
10
20
30
-5
0
5

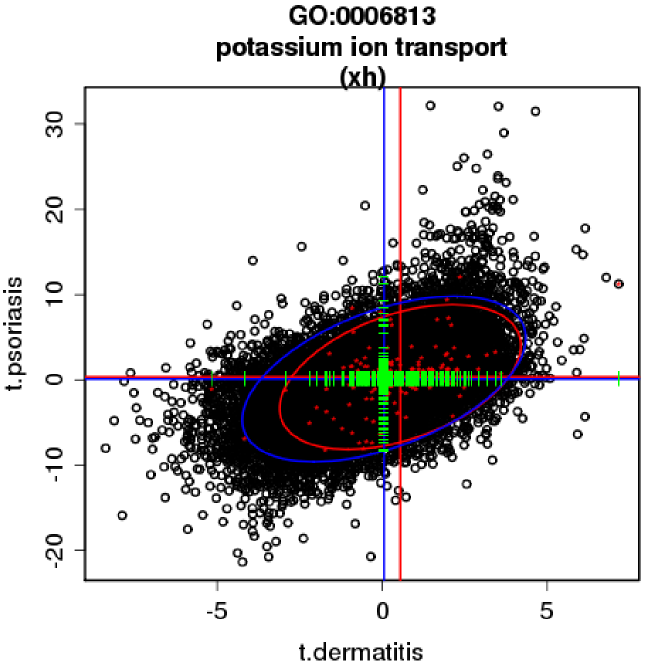
GO:0006813
potassium ion transport
(xh)
t.psoriasis
t.dermatitis
-20
-10
0
10
20
30
-5
0
5

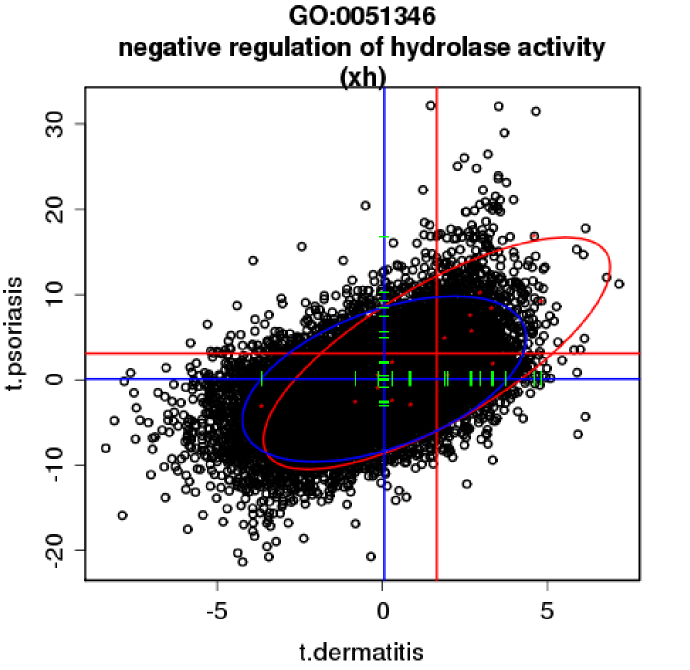
GO:0051346
negative regulation of hydrolase activity
(xh)
t.psoriasis
t.dermatitis
-20
-10
0
10
20
30
-5
0
5

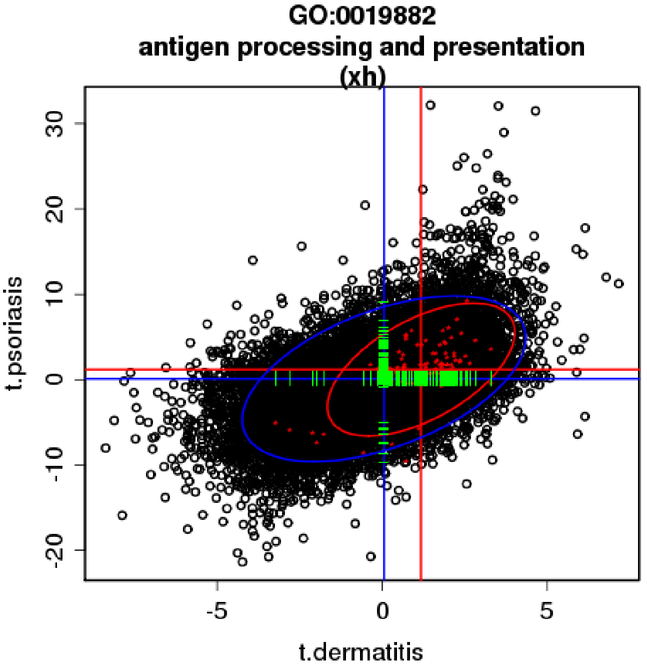
GO:0019882
antigen processing and presentation
(xh)
t.psoriasis
t.dermatitis
-20
-10
0
10
20
30
-5
0
5

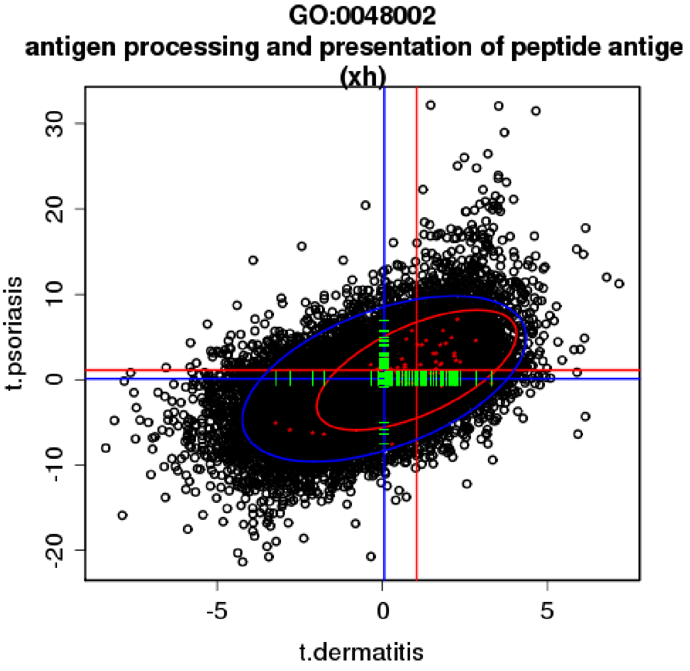
GO:0048002
antigen processing and presentation of peptide antige
(xh)
t.psoriasis
t.dermatitis
-20
-10
0
10
20
30
-5
0
5

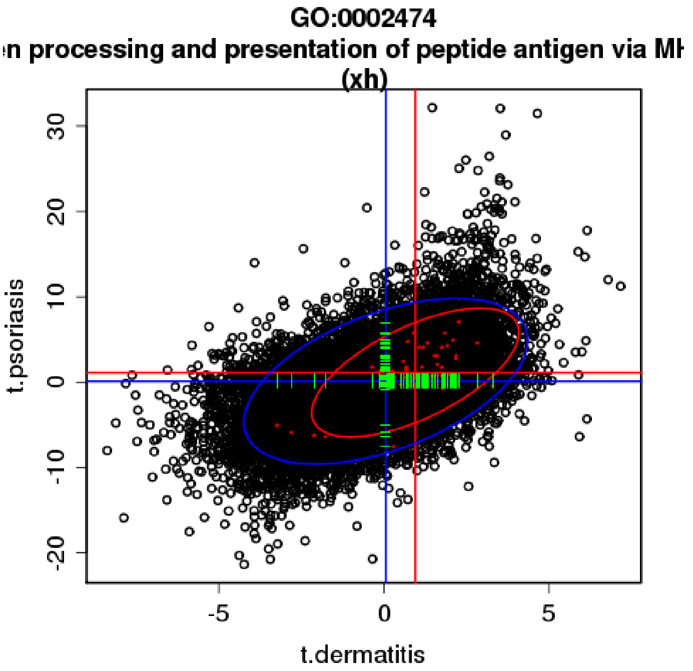
GO:0002474
n processing and presentation of peptide antigen via MH
(xh)
t.psoriasis
t.dermatitis
30
20
10
0
-10
-20
-5
0
5

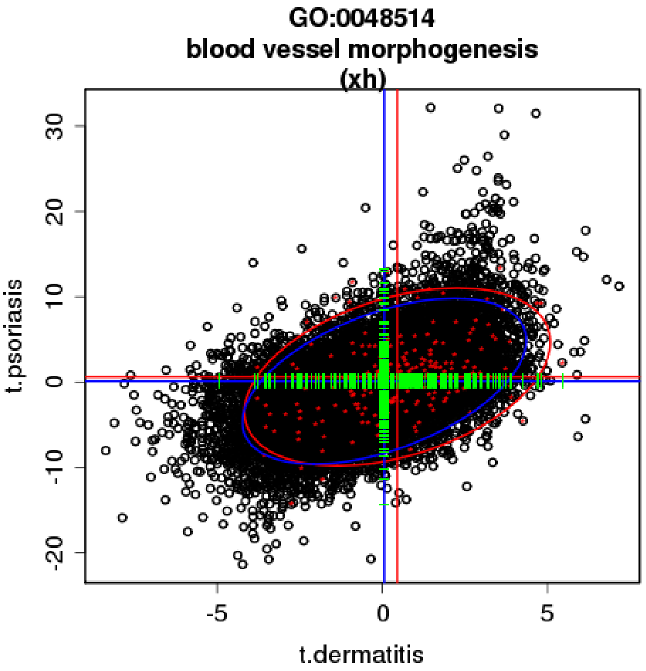
GO:0048514
blood vessel morphogenesis
(xh)
t.psoriasis
t.dermatitis
30
20
10
0
-10
-20
-5
0
5

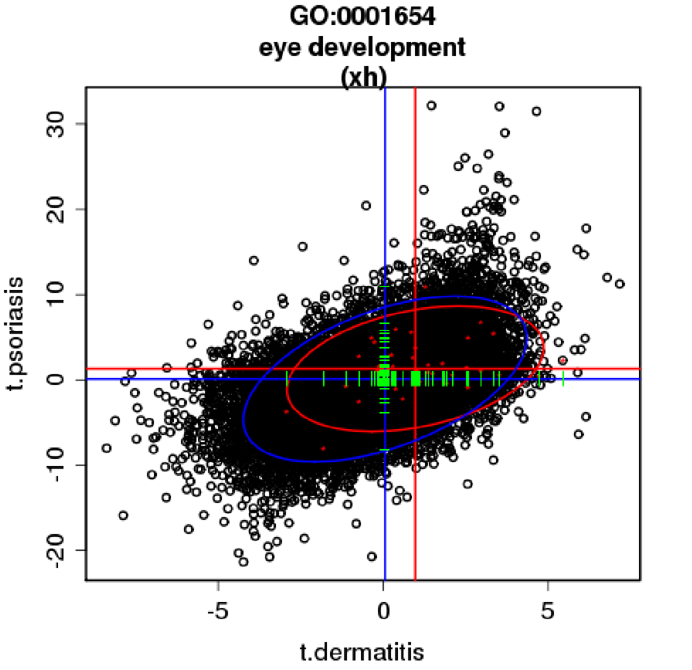
GO:0001654
eye development
(xh)
t.psoriasis
t.dermatitis
30
20
10
0
-10
-20
-5
0
5

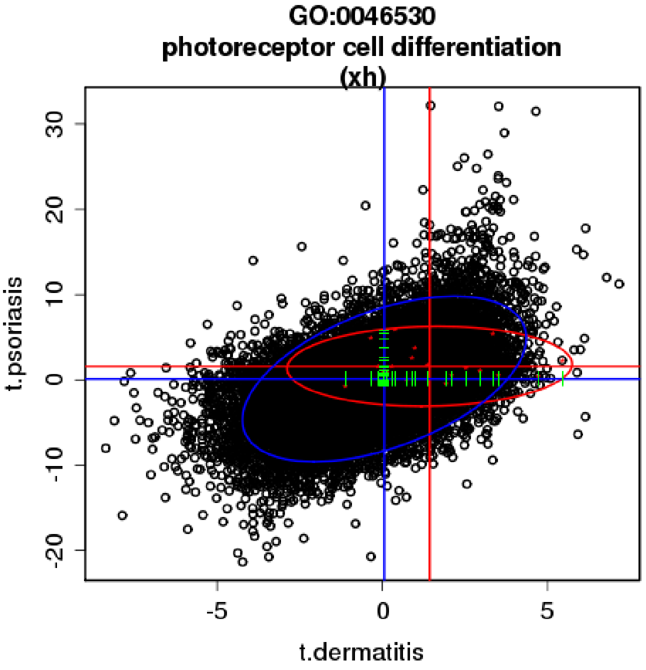
GO:0046530
photoreceptor cell differentiation
(xh)
t.psoriasis
t.dermatitis
30
20
10
0
-10
-20
-5
0
5

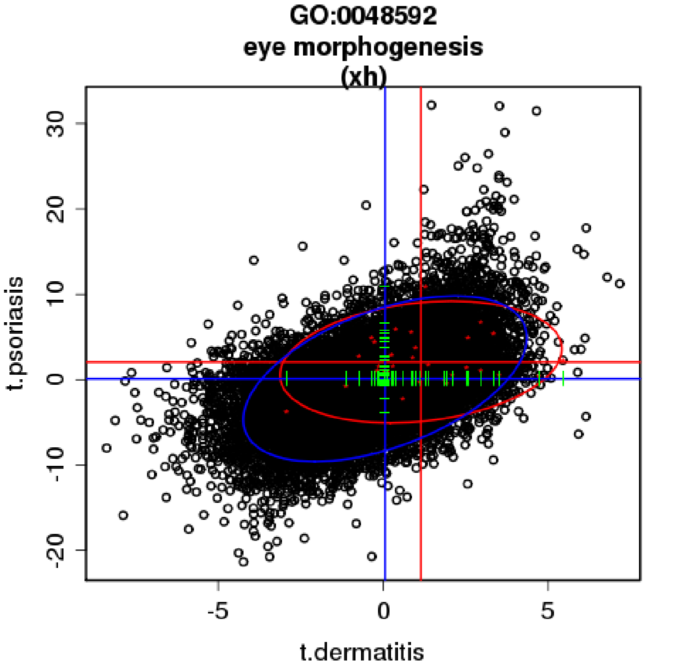
GO:0048592
eye morphogenesis
(xh)
t.psoriasis
t.dermatitis
30
20
10
0
-10
-20
-5
0
5

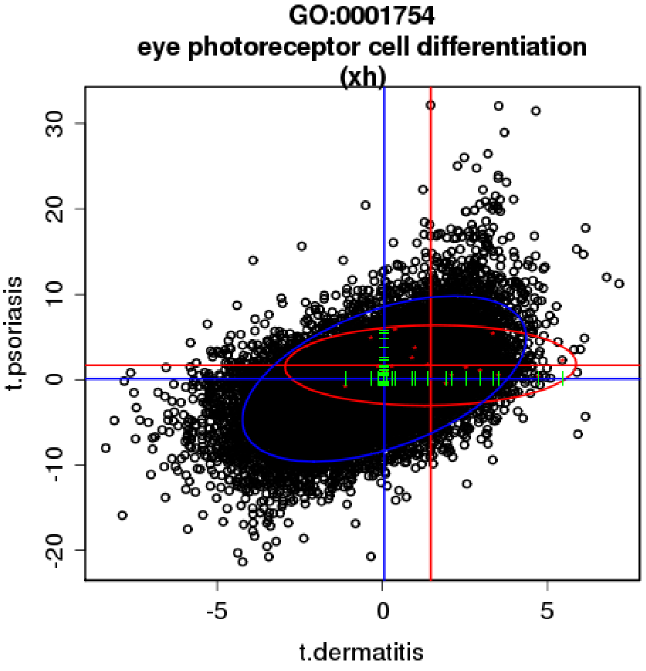
GO:0001754
eye photoreceptor cell differentiation
(xh)
t.psoriasis
t.dermatitis
30
20
10
0
-10
-20
-5
0
5

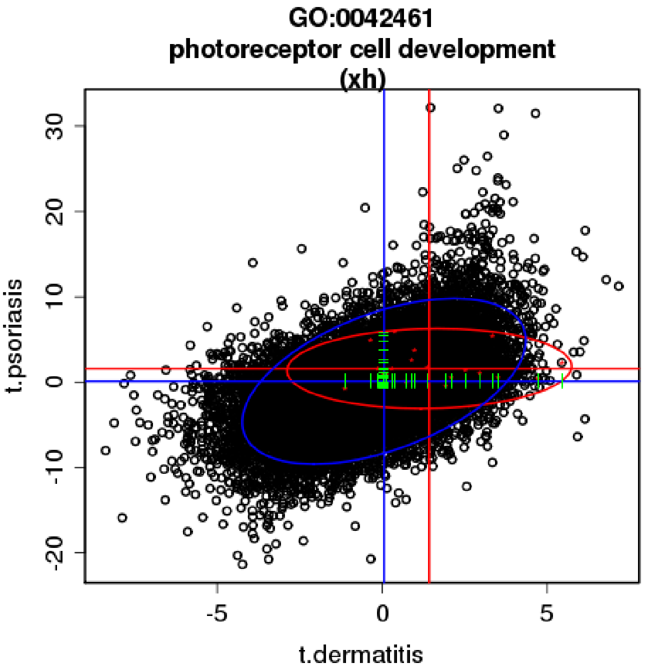
GO:0042461
photoreceptor cell development
(xh)
t.psoriasis
t.dermatitis

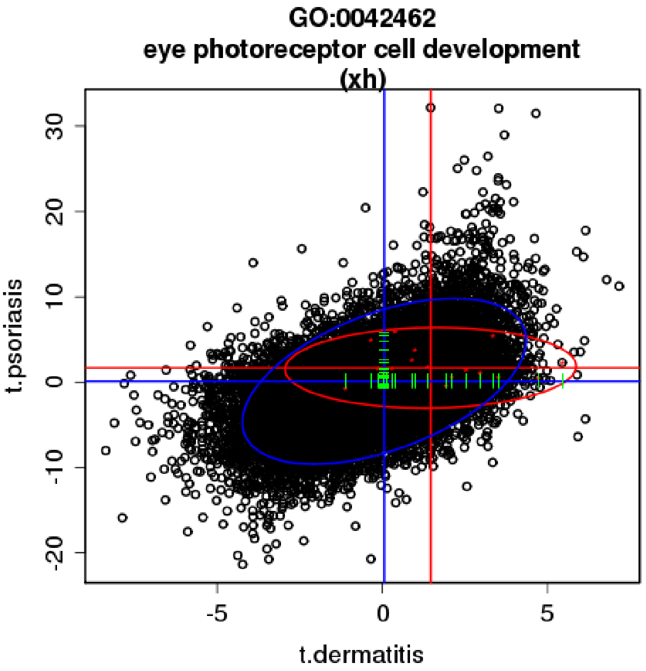
GO:0042462
eye photoreceptor cell development
(xh)
t.psoriasis
t.dermatitis

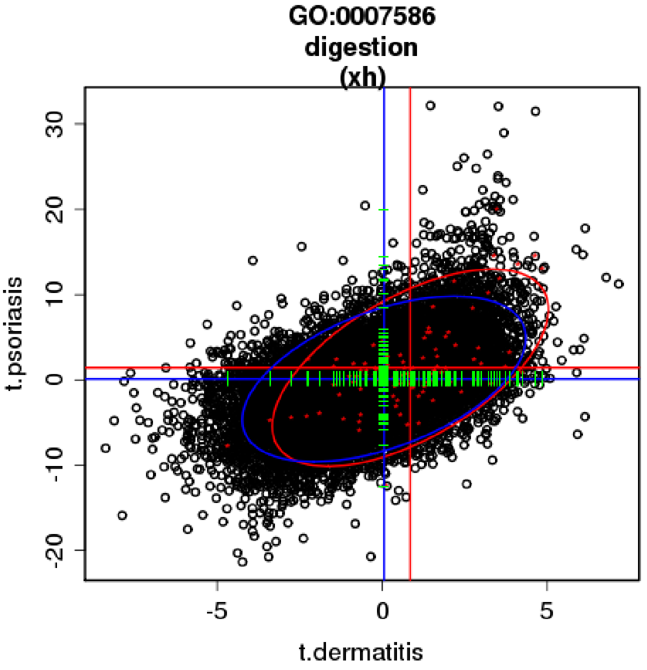
GO:0007586
digestion
(xh)
t.psoriasis
t.dermatitis

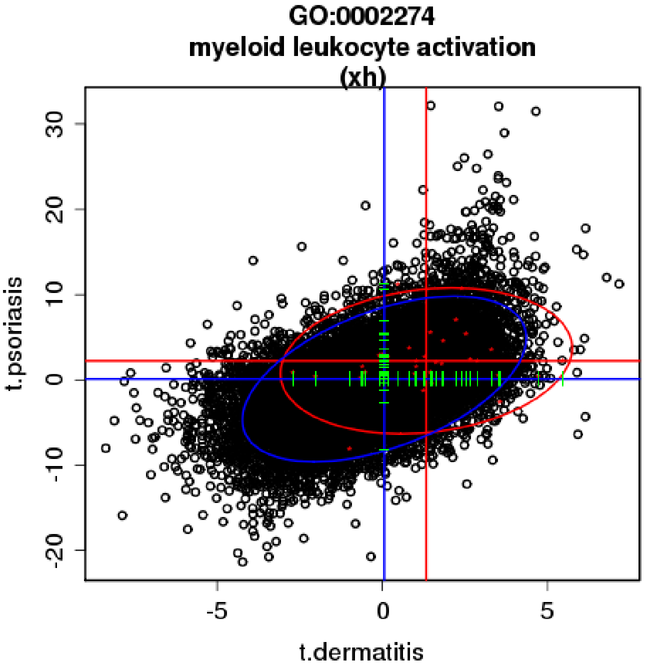
GO:0002274
myeloid leukocyte activation
(xh)
t.psoriasis
t.dermatitis

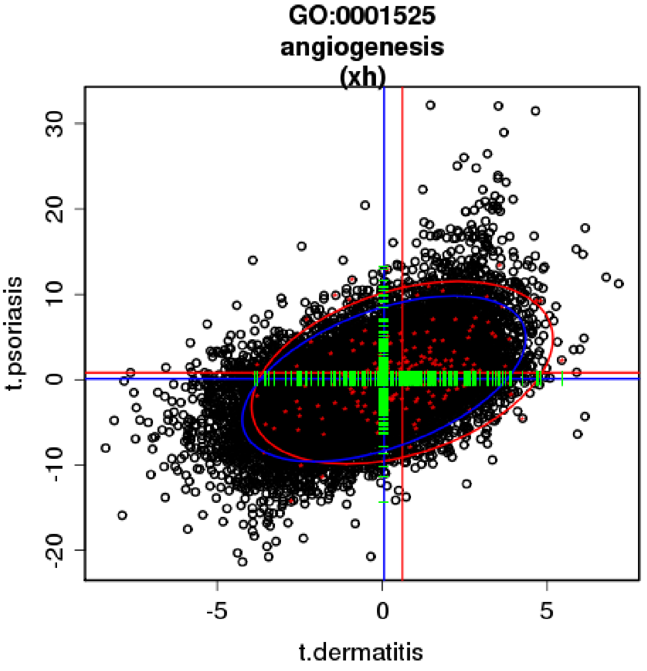
GO:0001525
angiogenesis
(xh)
t.psoriasis
t.dermatitis

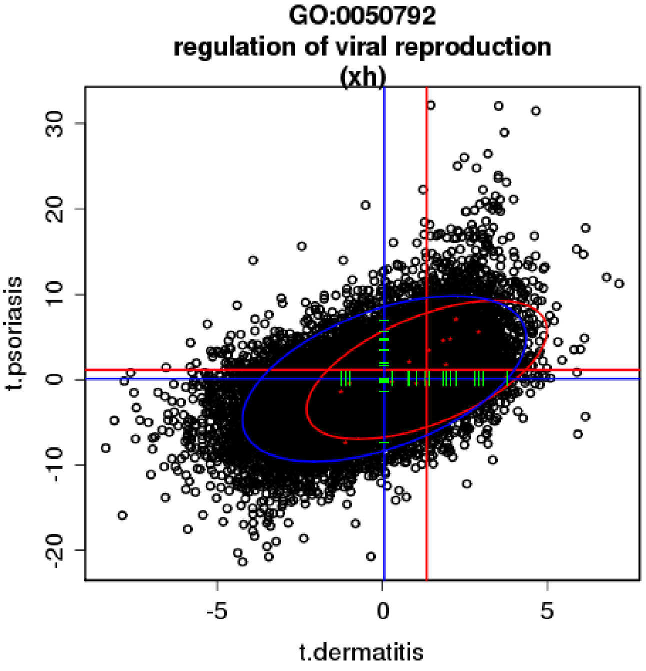
GO:0050792
regulation of viral reproduction
(xh)
t.psoriasis
t.dermatitis

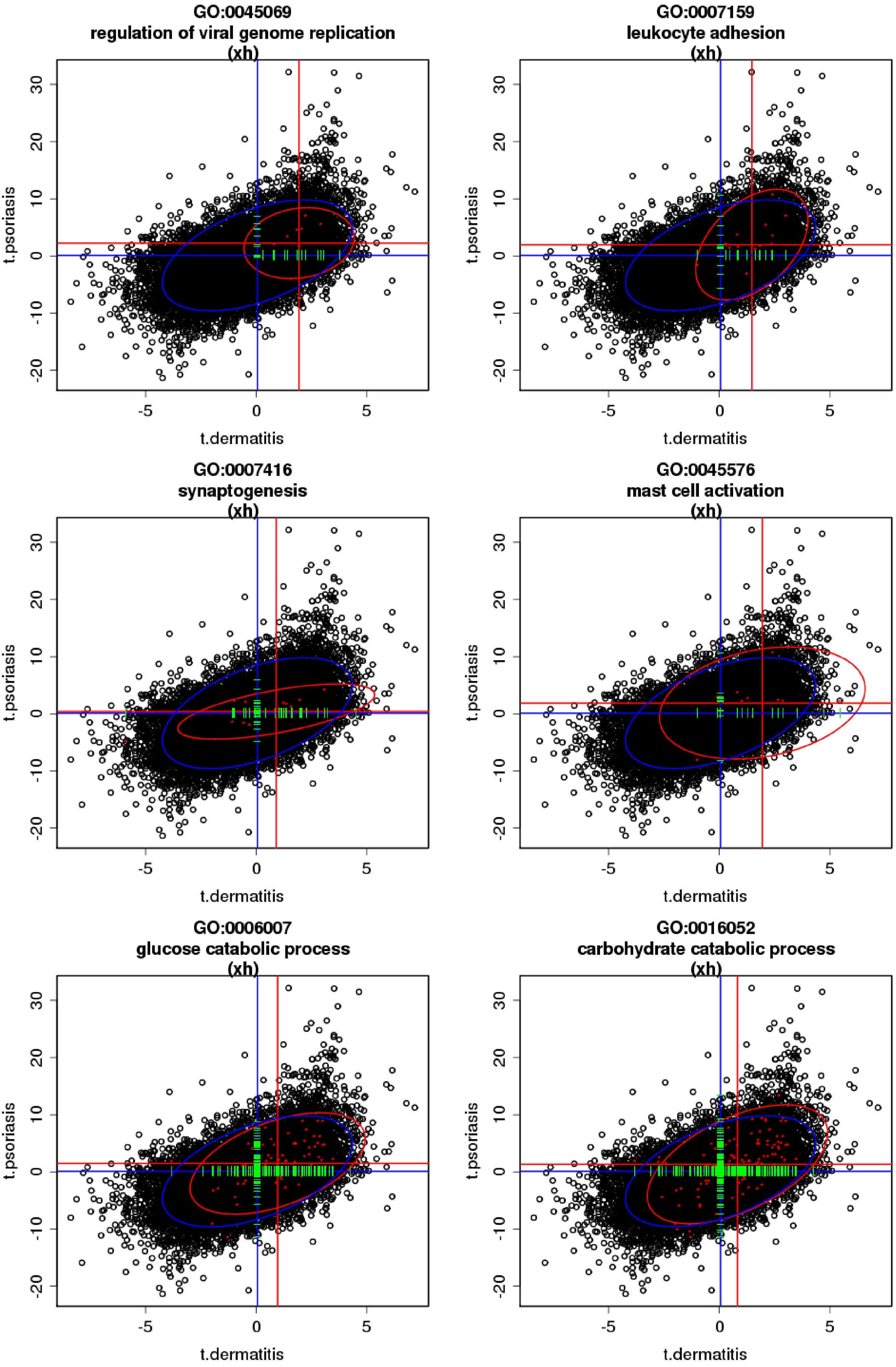
GO:0045069
regulation of viral genome replication
(xh)
GO:0007159
leukocyte adhesion
(xh)
GO:0007416
synaptogenesis
(xh)
GO:0045576
mast cell activation
(xh)
GO:0006007
glucose catabolic process
(xh)
GO:0016052
carbohydrate catabolic process
(xh)
t.psoriasis
t.dermatitis
-20
-10
0
10
20
30
-5
0
5

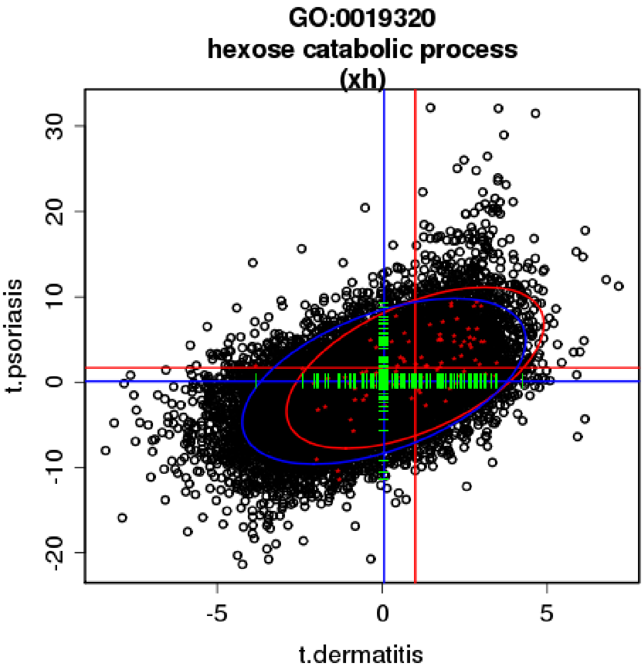
GO:0019320
hexose catabolic process
(xh)
t.psoriasis
t.dermatitis

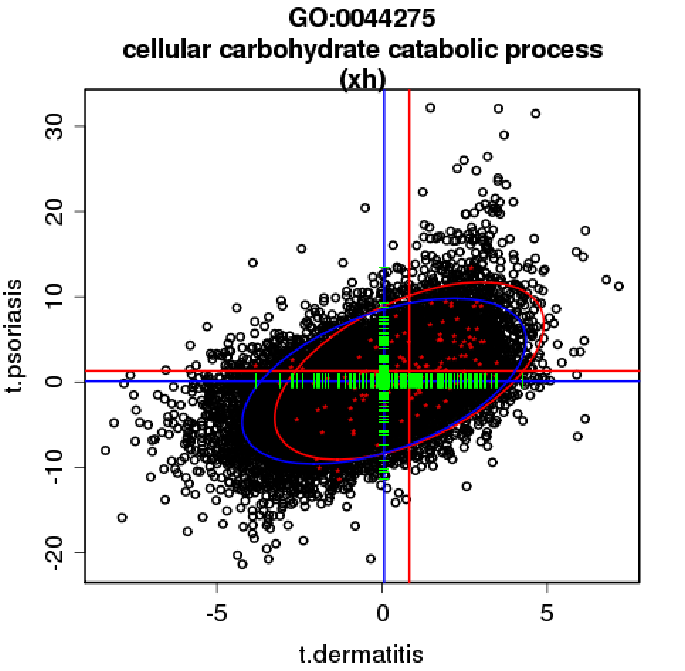
GO:0044275
cellular carbohydrate catabolic process
(xh)
t.psoriasis
t.dermatitis

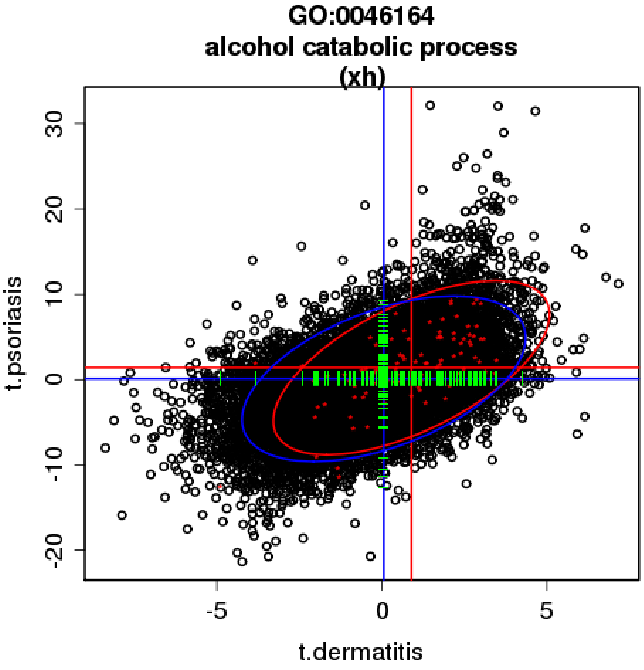
GO:0046164
alcohol catabolic process
(xh)
t.psoriasis
t.dermatitis

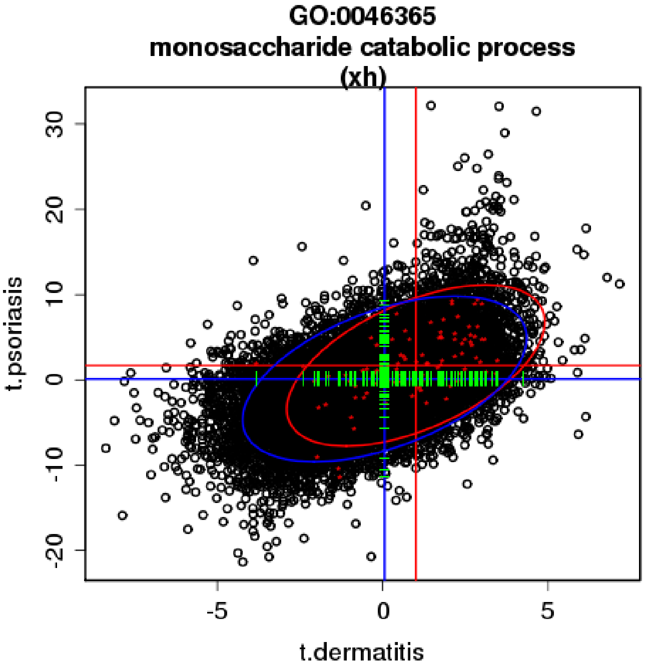
GO:0046365
monosaccharide catabolic process
(xh)
t.psoriasis
t.dermatitis

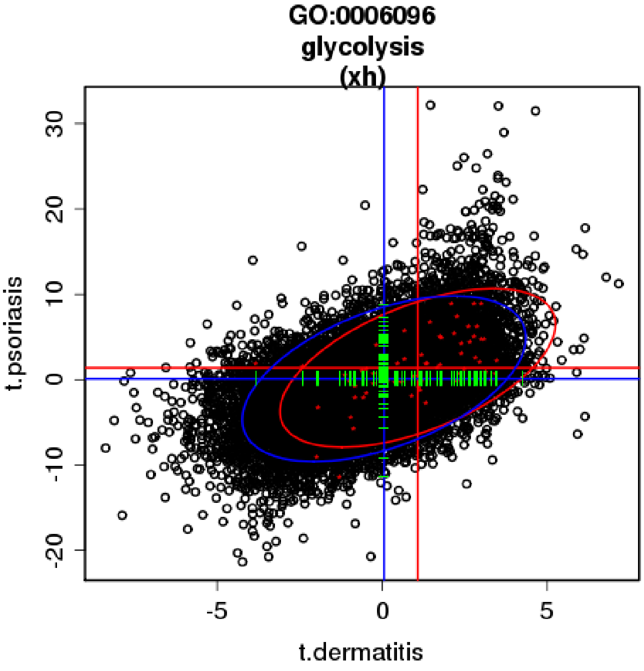
GO:0006096
glycolysis
(xh)
t.psoriasis
t.dermatitis

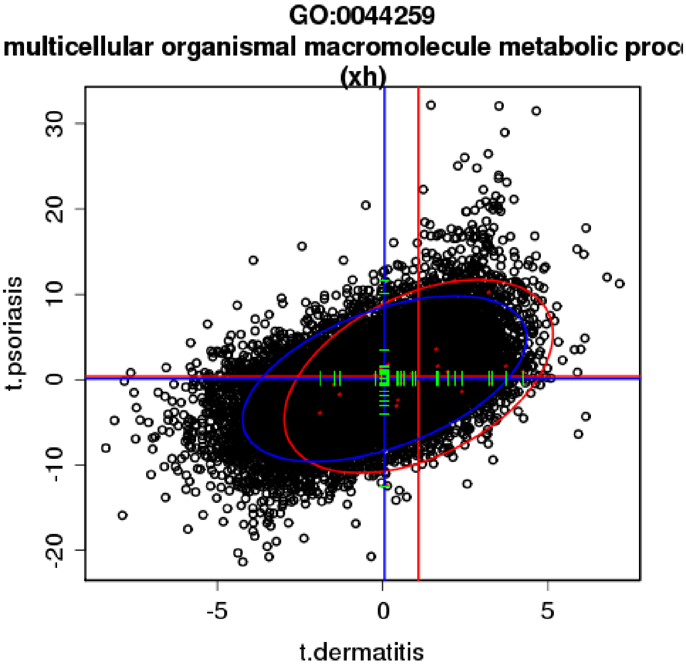
GO:0044259
multicellular organismal macromolecule metabolic proc
(xh)
t.psoriasis
t.dermatitis

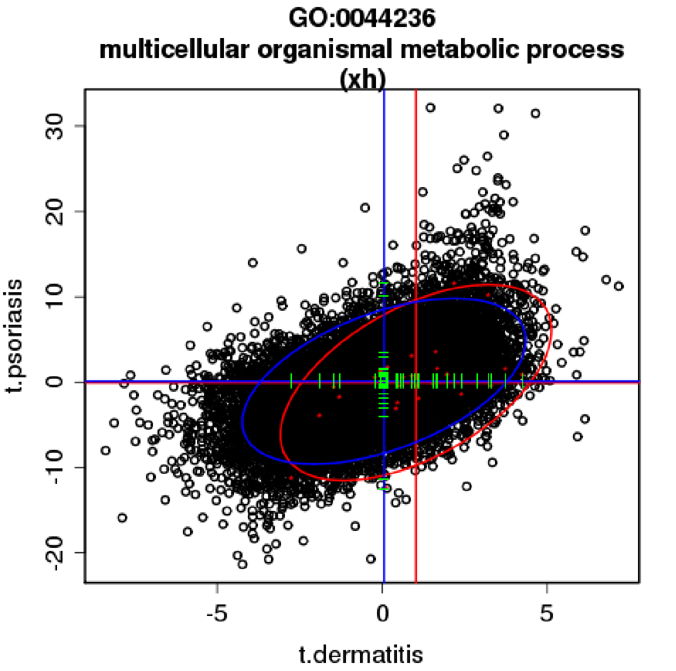
GO:0044236
multicellular organismal metabolic process
(xh)
t.psoriasis
t.dermatitis

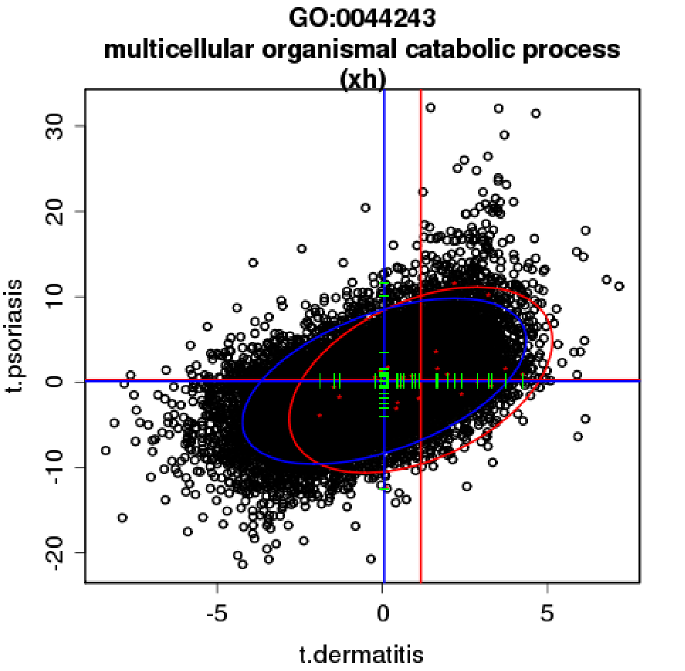
GO:0044243
multicellular organismal catabolic process
(xh)
t.psoriasis
t.dermatitis

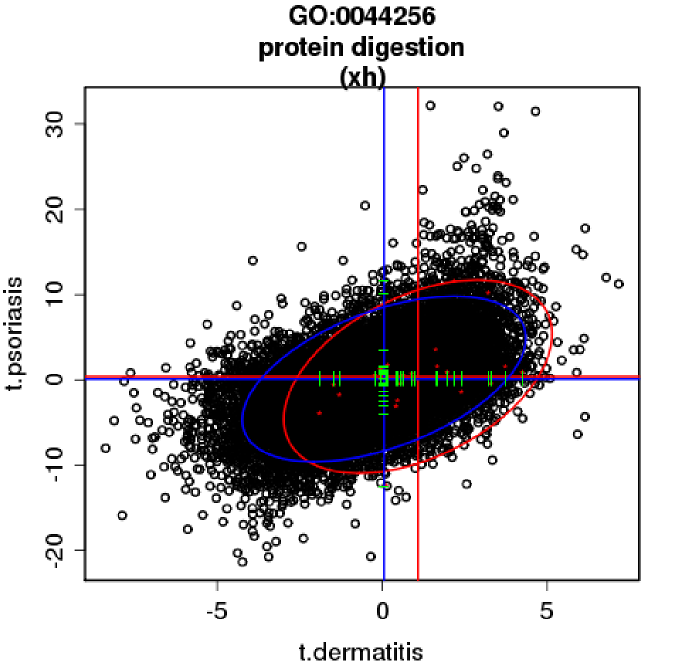
GO:0044256
protein digestion
(xh)
t.psoriasis
t.dermatitis

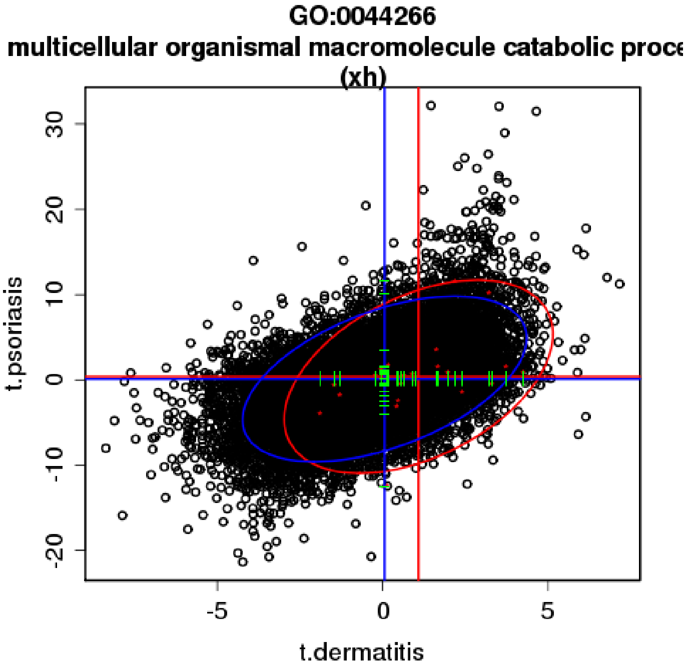
GO:0044266
multicellular organismal macromolecule catabolic proce
(xh)
t.psoriasis
t.dermatitis

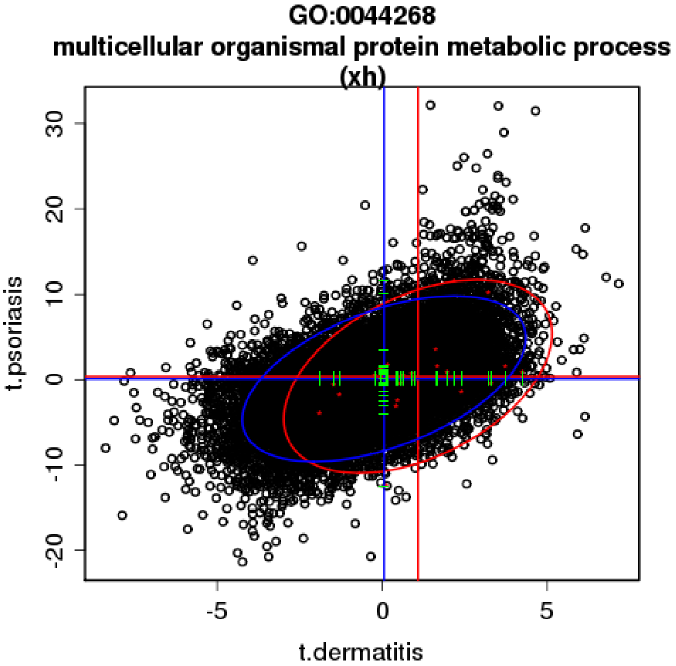
GO:0044268
multicellular organismal protein metabolic process
(xh)
t.psoriasis
t.dermatitis

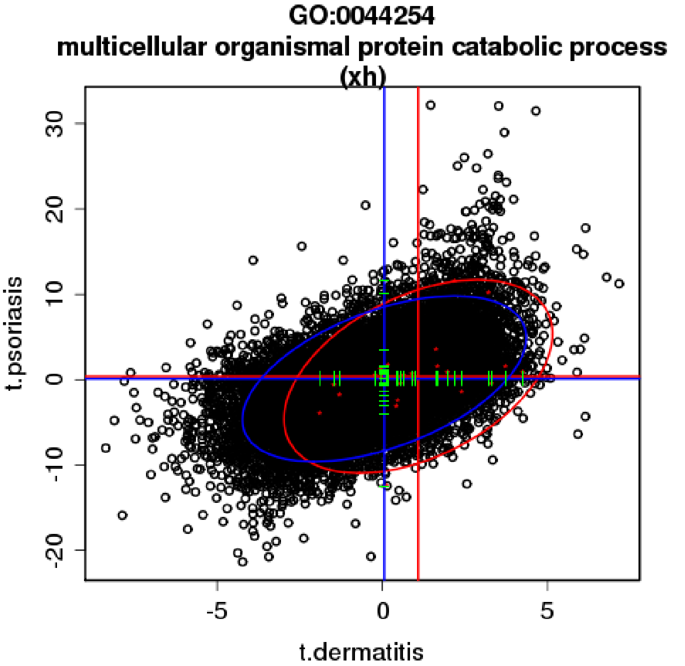
GO:0044254
multicellular organismal protein catabolic process
(xh)
t.psoriasis
t.dermatitis

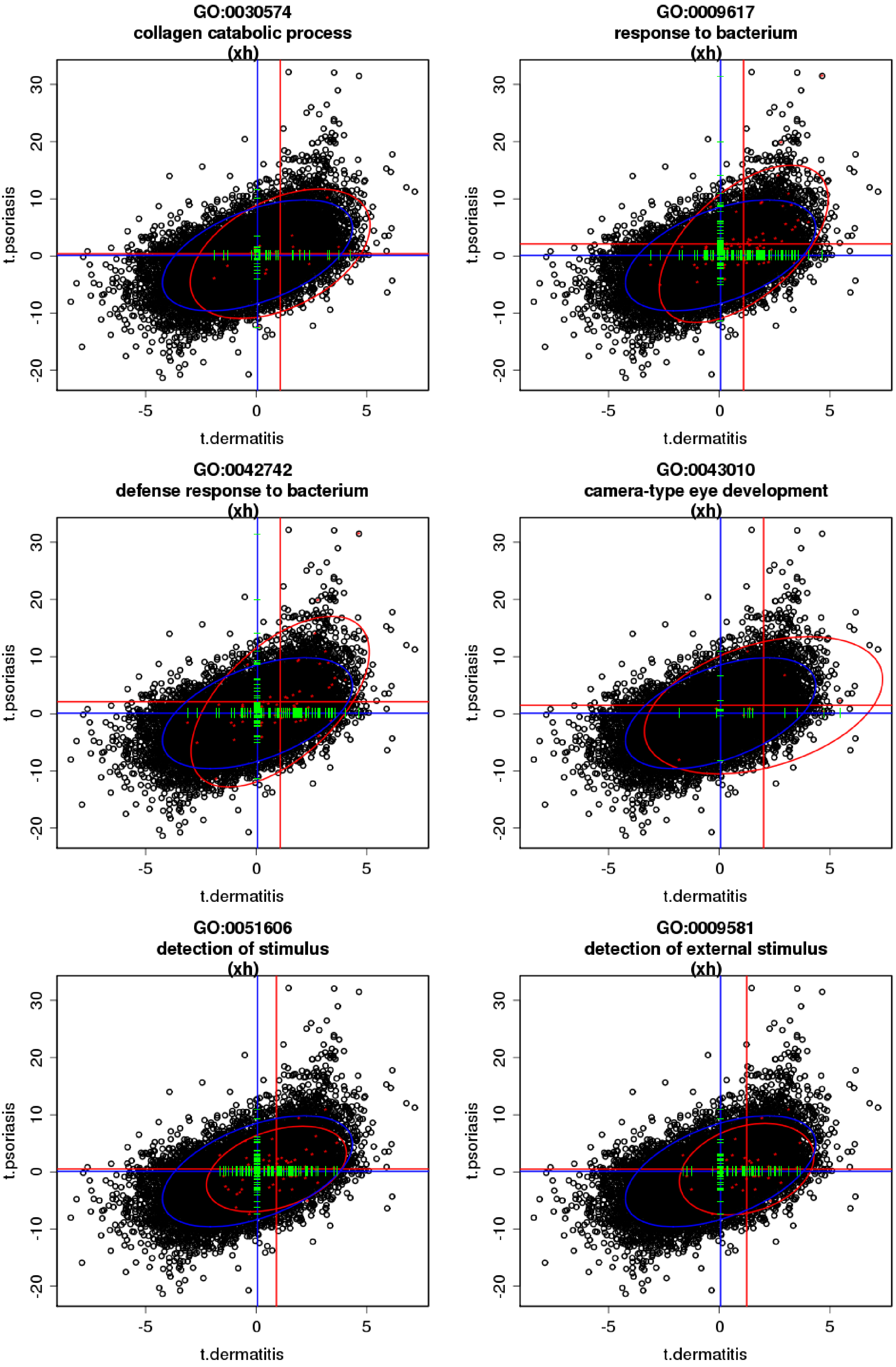
GO:0030574
collagen catabolic process
(xh)
GO:0009617
response to bacterium
(xh)
GO:0042742
defense response to bacterium
(xh)
GO:0043010
camera-type eye development
(xh)
GO:0051606
detection of stimulus
(xh)
GO:0009581
detection of external stimulus
(xh)
t.psoriasis
t.dermatitis
-20
-10
0
10
20
30
-5
0
5

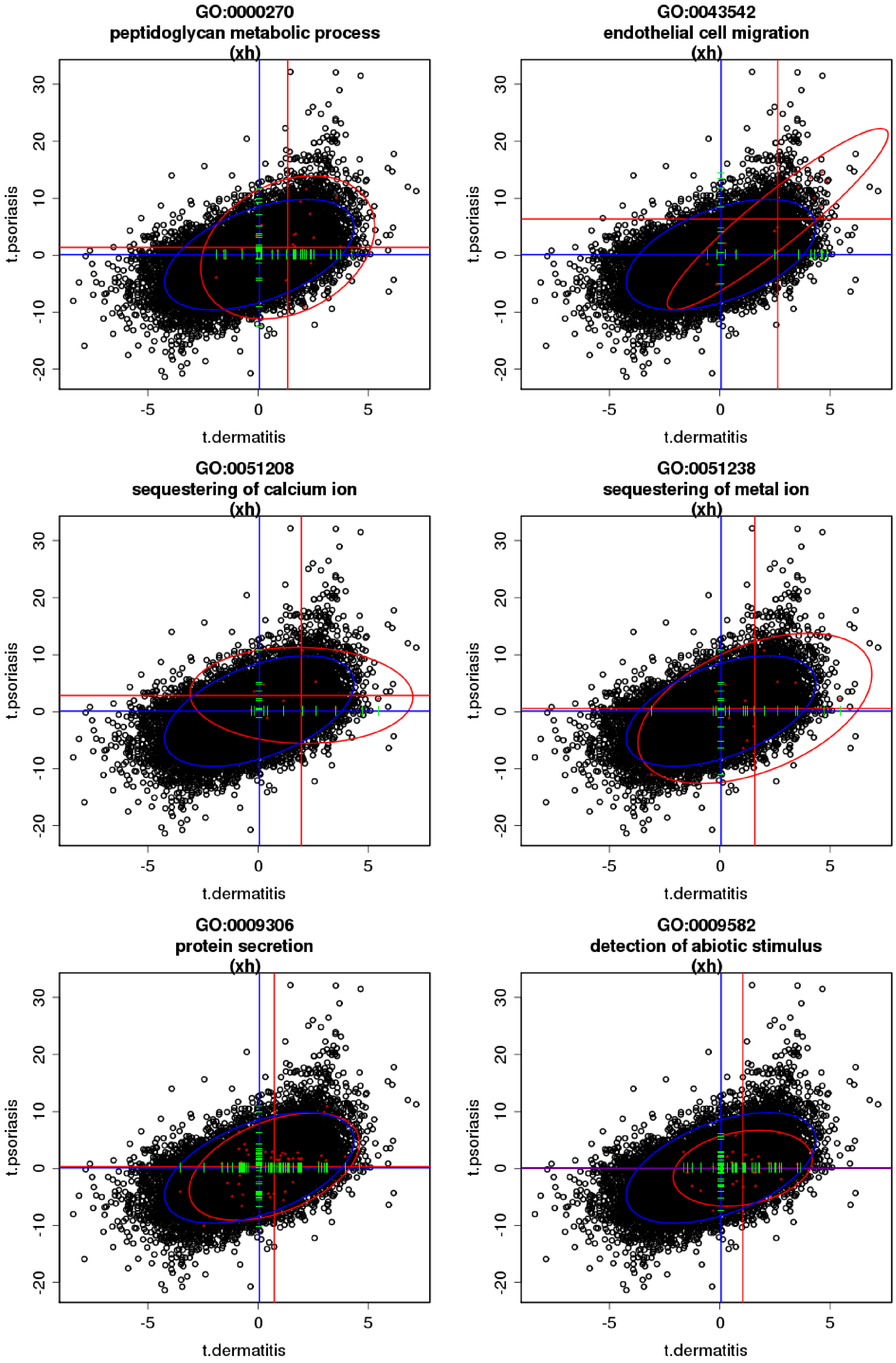
GO:0000270
peptidoglycan metabolic process
(xh)
t.psoriasis
t.dermatitis
GO:0043542
endothelial cell migration
(xh)
t.psoriasis
t.dermatitis
GO:0051208
sequestering of calcium ion
(xh)
t.psoriasis
t.dermatitis
GO:0051238
sequestering of metal ion
(xh)
t.psoriasis
t.dermatitis
GO:0009306
protein secretion
(xh)
t.psoriasis
t.dermatitis
GO:0009582
detection of abiotic stimulus
(xh)
t.psoriasis
t.dermatitis

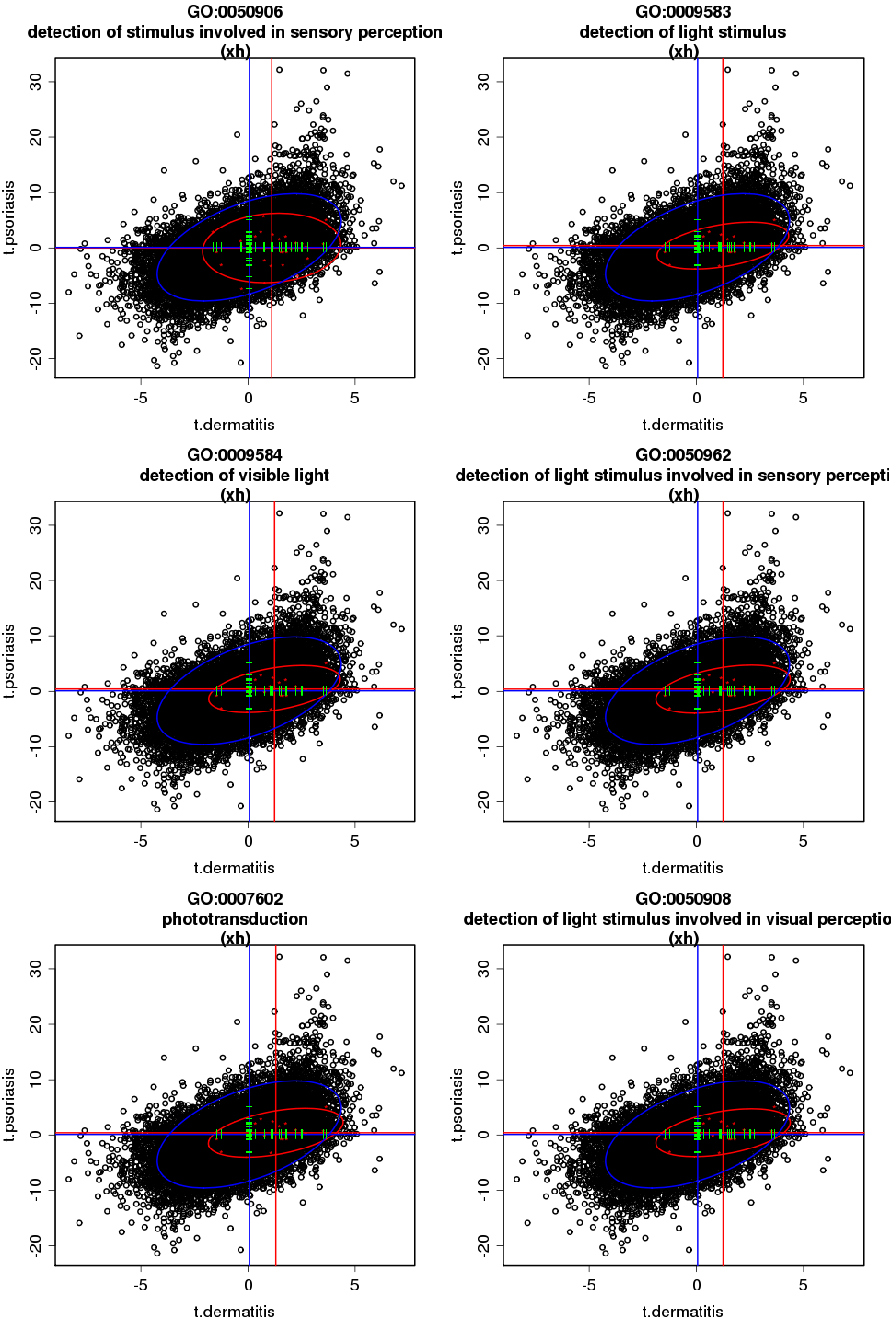
GO:0050906
detection of stimulus involved in sensory perception
(xh)
t.psoriasis
t.dermatitis
GO:0009583
detection of light stimulus
(xh)
t.psoriasis
t.dermatitis
GO:0009584
detection of visible light
(xh)
t.psoriasis
t.dermatitis
GO:0050962
detection of light stimulus involved in sensory percepti
(xh)
t.psoriasis
t.dermatitis
GO:0007602
phototransduction
(xh)
t.psoriasis
t.dermatitis
GO:0050908
detection of light stimulus involved in visual perceptio
(xh)
t.psoriasis
t.dermatitis

GO:0002504
sing and presentation of peptide or polysaccharide antig
(xh)

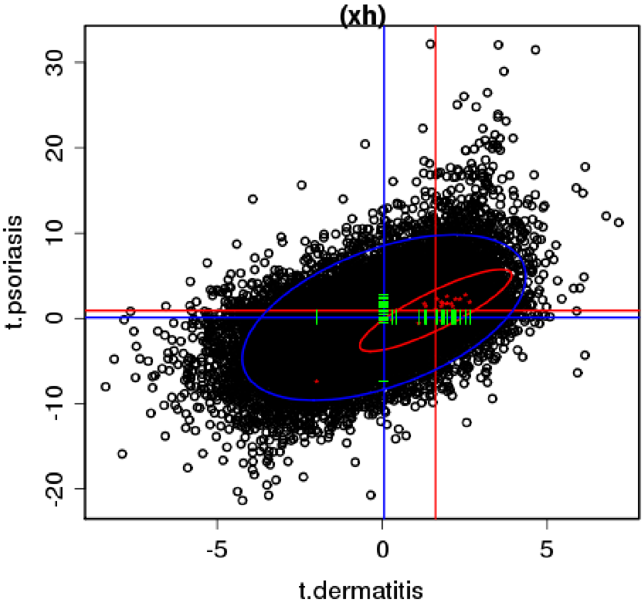

GO:0045045
(xl)

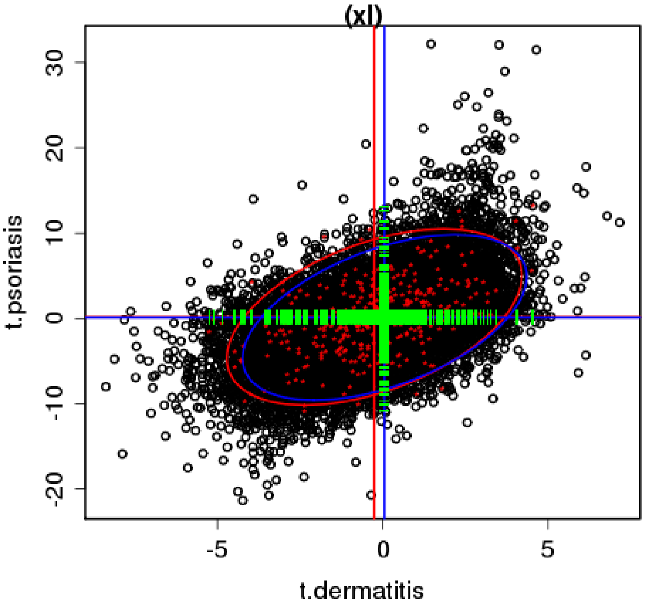

GO:0006325
establishment and/or maintenance of chromatin architec
(xl)

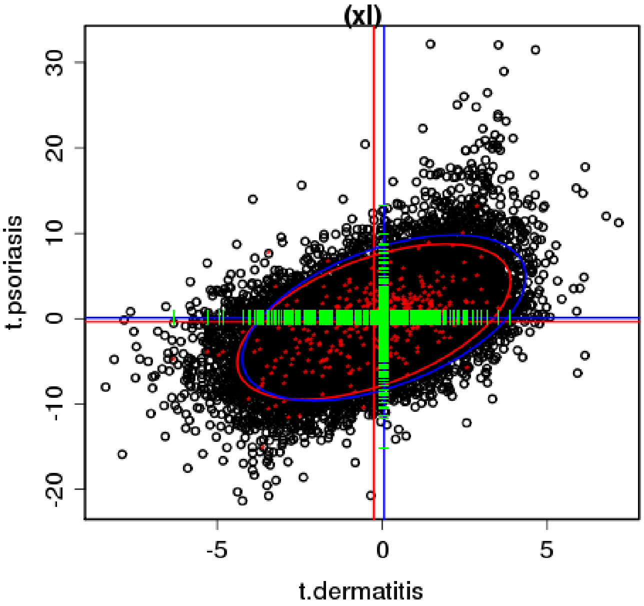

GO:0051276
chromosome organization and biogenesis
(xl)

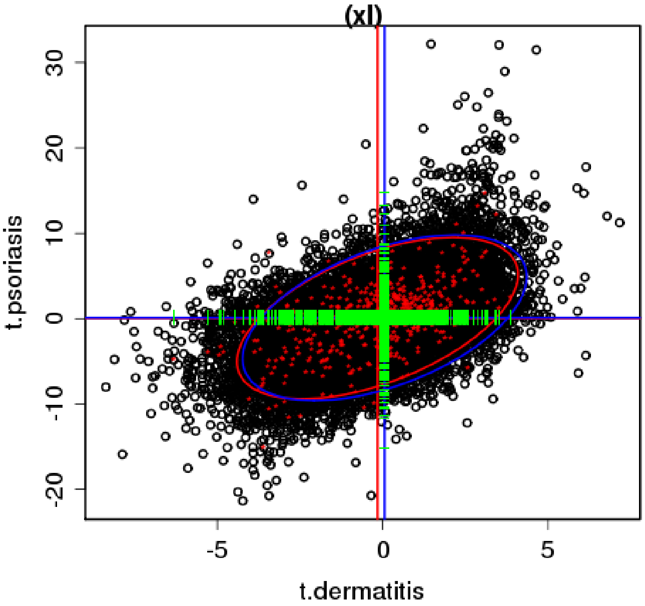

GO:0016568
chromatin modification
(xl)

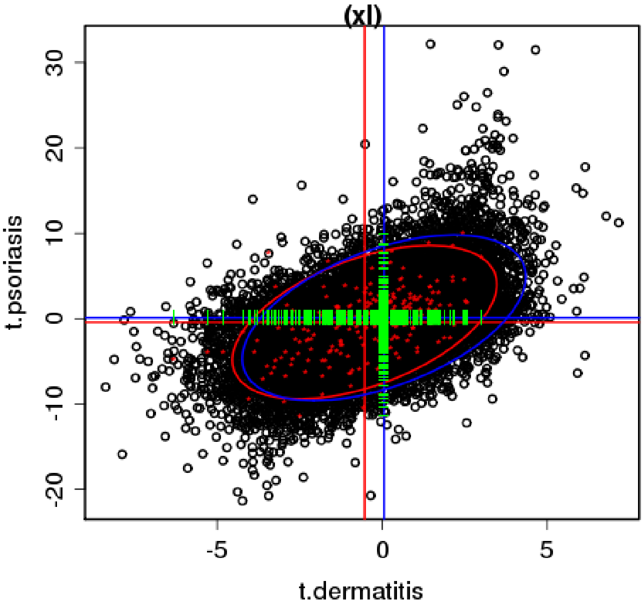

GO:0007264
small GTPase mediated signal transduction
(xl)

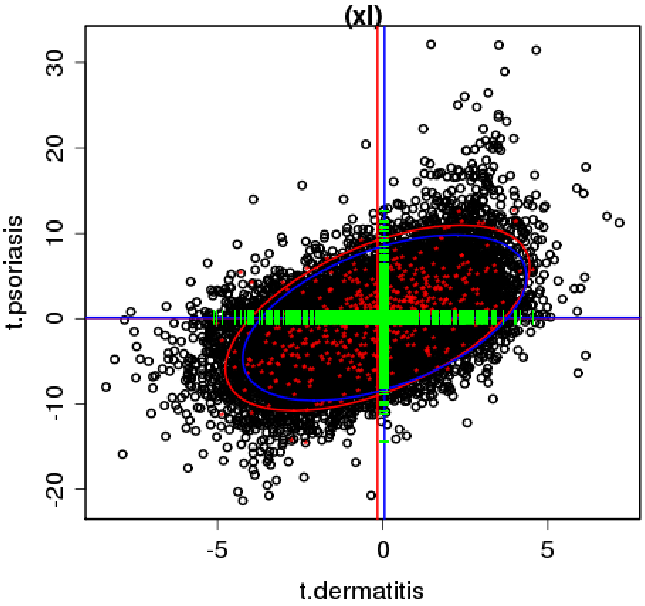

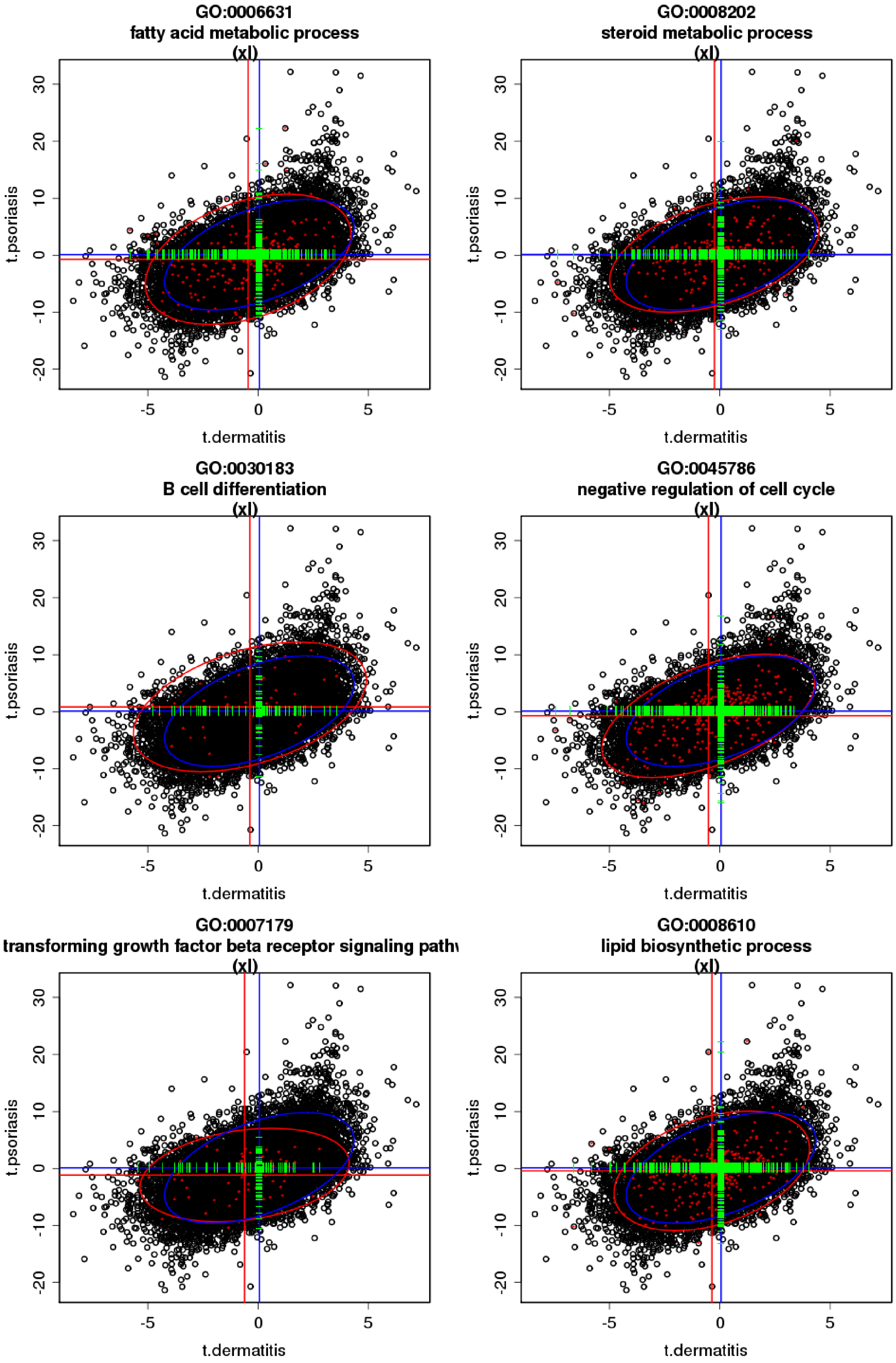
GO:0006631
fatty acid metabolic process
(xl)
GO:0008202
steroid metabolic process
(xl)
GO:0030183
B cell differentiation
(xl)
GO:0045786
negative regulation of cell cycle
(xl)
GO:0007179
transforming growth factor beta receptor signaling pathv
(xl)
GO:0008610
lipid biosynthetic process
(xl)
t.psoriasis
t.dermatitis
-20
-10
0
10
20
30
-5
0
5

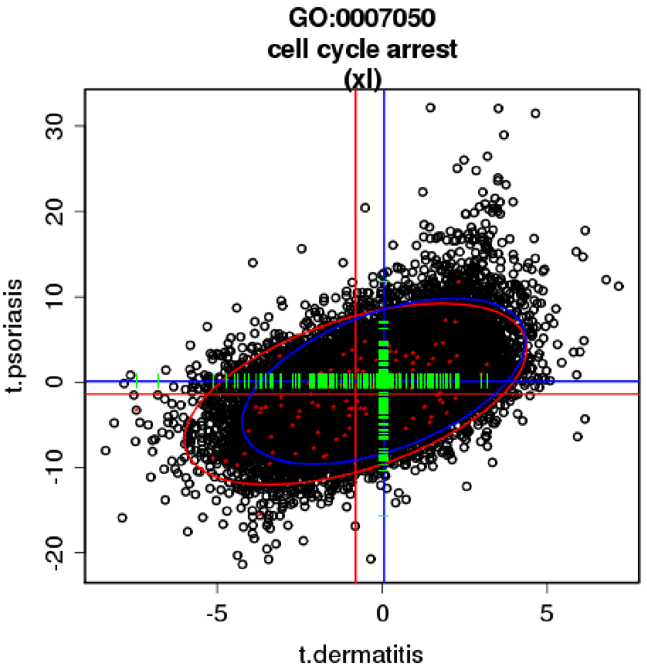
GO:0007050
cell cycle arrest
(xl)
t.psoriasis
t.dermatitis
30
20
10
0
-10
-20
-5
0
5

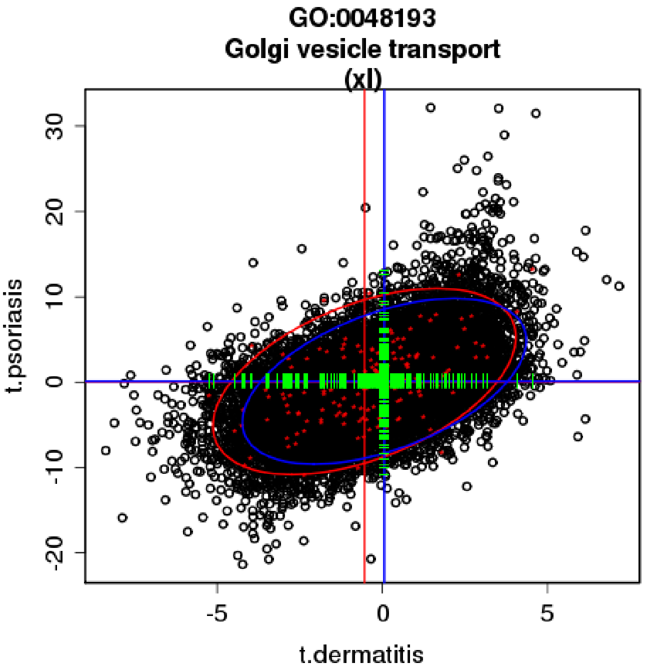
GO:0048193
Golgi vesicle transport
(xl)
t.psoriasis
t.dermatitis
30
20
10
0
-10
-20
-5
0
5

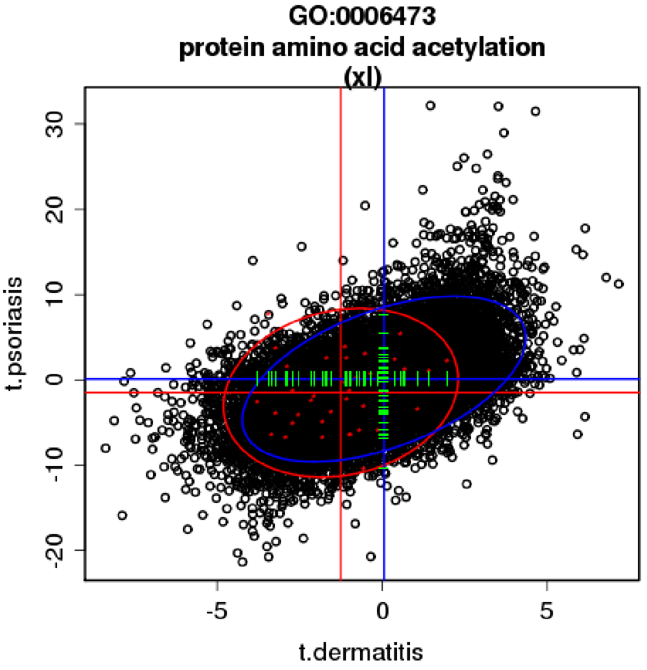
GO:0006473
protein amino acid acetylation
(xl)
t.psoriasis
t.dermatitis
30
20
10
0
-10
-20
-5
0
5

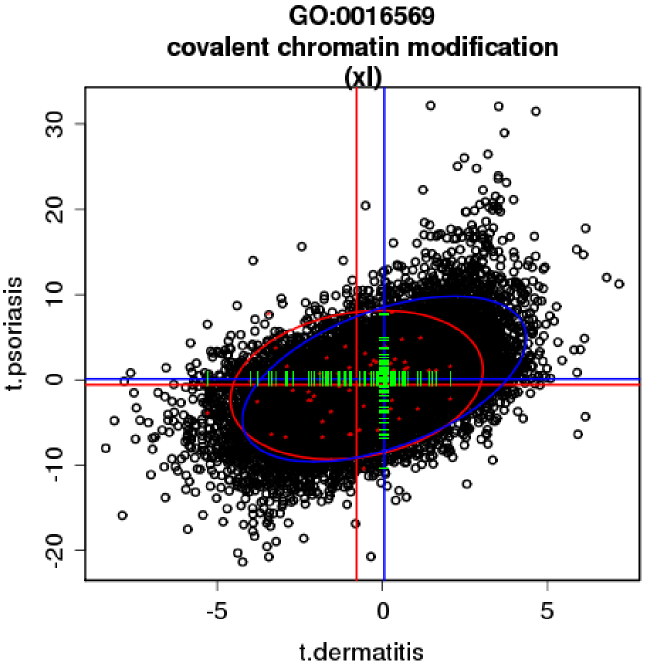
GO:0016569
covalent chromatin modification
(xl)
t.psoriasis
t.dermatitis
30
20
10
0
-10
-20
-5
0
5

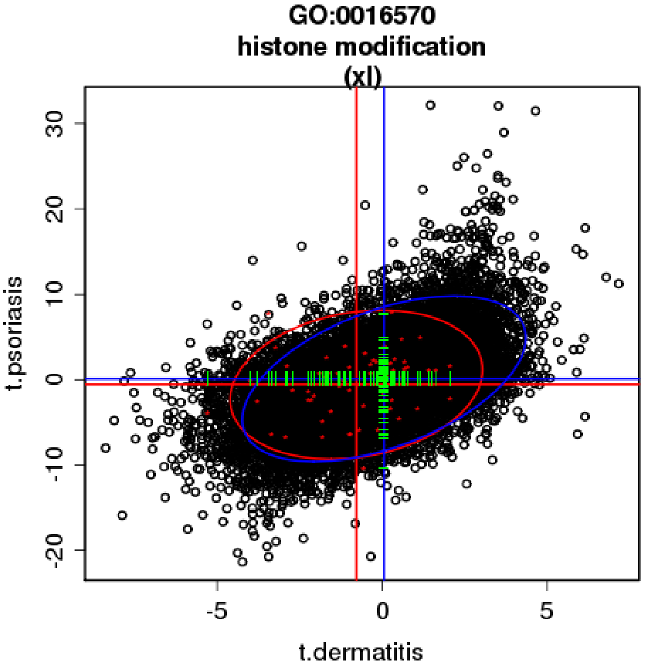
GO:0016570
histone modification
(xl)
t.psoriasis
t.dermatitis
30
20
10
0
-10
-20
-5
0
5

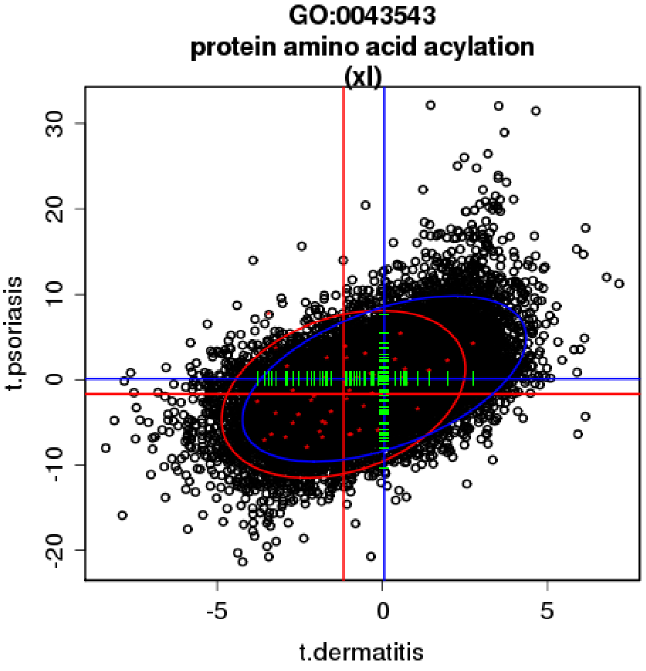
GO:0043543
protein amino acid acylation
(xl)
t.psoriasis
t.dermatitis
30
20
10
0
-10
-20
-5
0
5

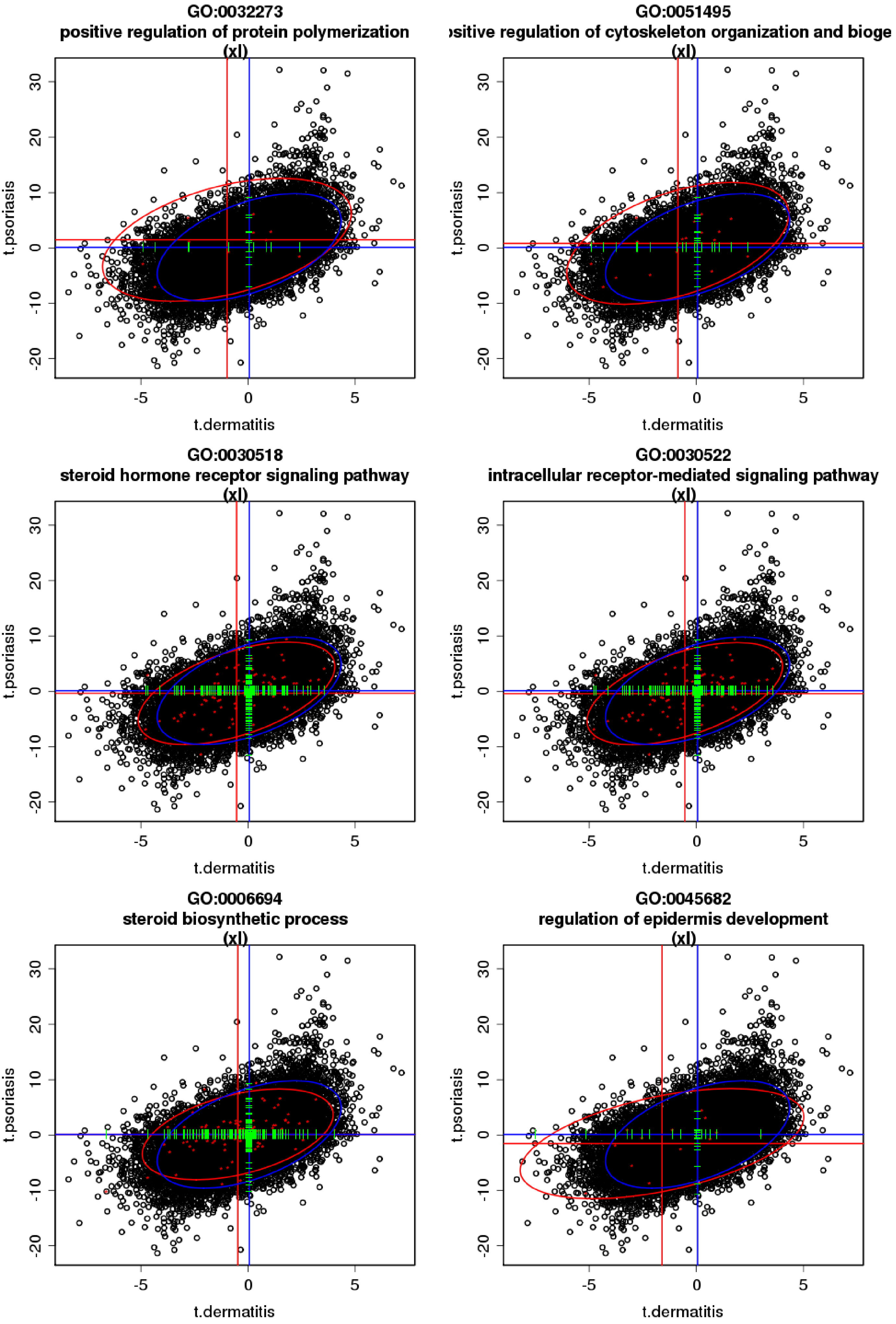

GO:0032273
positive regulation of protein polymerization
(xl)
t.psoriasis
t.dermatitis
GO:0051495
ɔsitive regulation of cytoskeleton organization and bioge
(xl)
t.psoriasis
t.dermatitis
GO:0030518
steroid hormone receptor signaling pathway
(xl)
t.psoriasis
t.dermatitis
GO:0030522
intracellular receptor-mediated signaling pathway
(xl)
t.psoriasis
t.dermatitis
GO:0006694
steroid biosynthetic process
(xl)
t.psoriasis
t.dermatitis
GO:0045682
regulation of epidermis development
(xl)
t.psoriasis
t.dermatitis

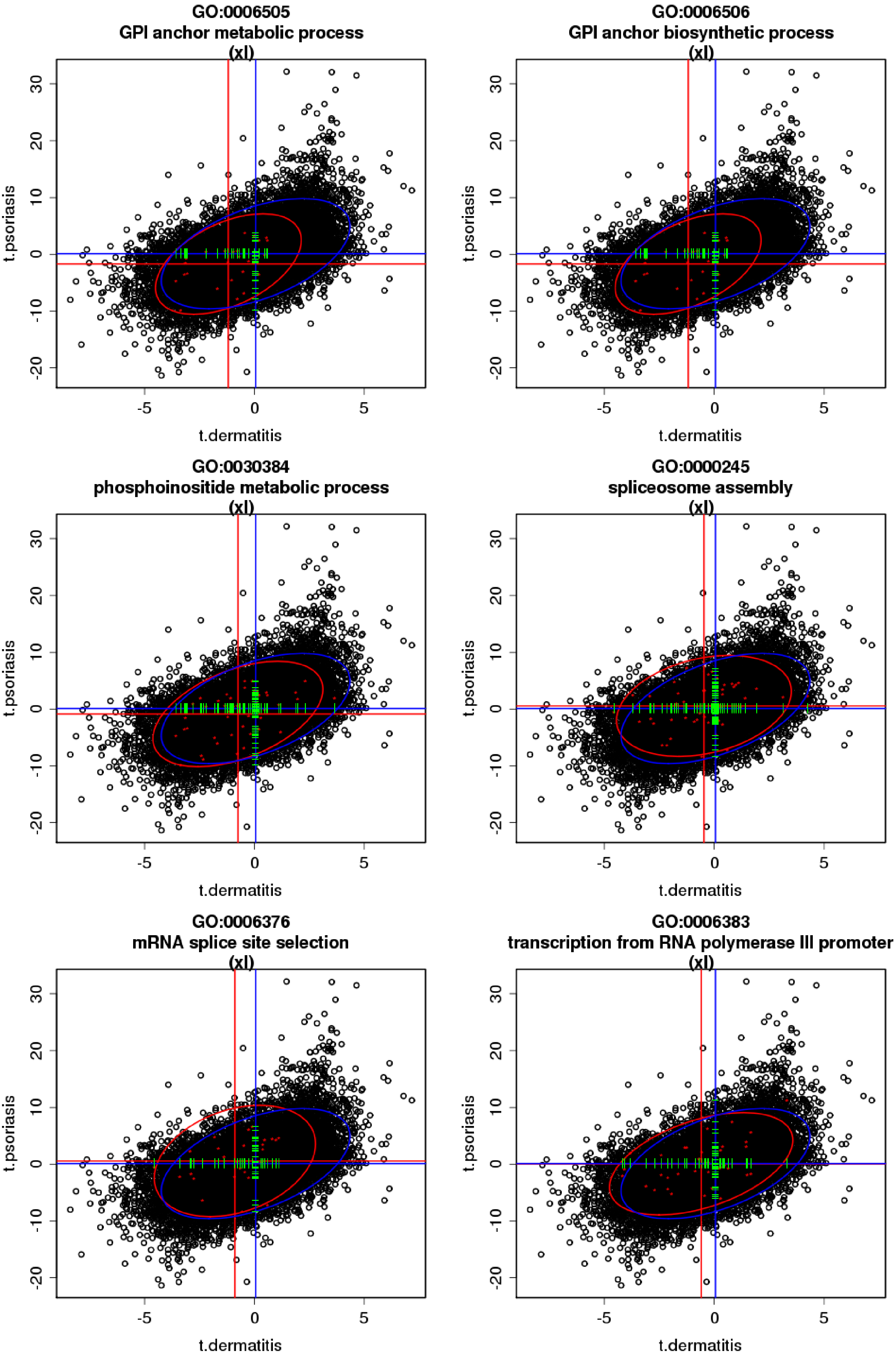
GO:0006505
GPI anchor metabolic process
(xl)
GO:0006506
GPI anchor biosynthetic process
(xl)
GO:0030384
phosphoinositide metabolic process
(xl)
GO:0000245
spliceosome assembly
(xl)
GO:0006376
mRNA splice site selection
(xl)
GO:0006383
transcription from RNA polymerase III promoter
(xl)
t.psoriasis
t.dermatitis
-20
-10
0
10
20
30
-5
0
5

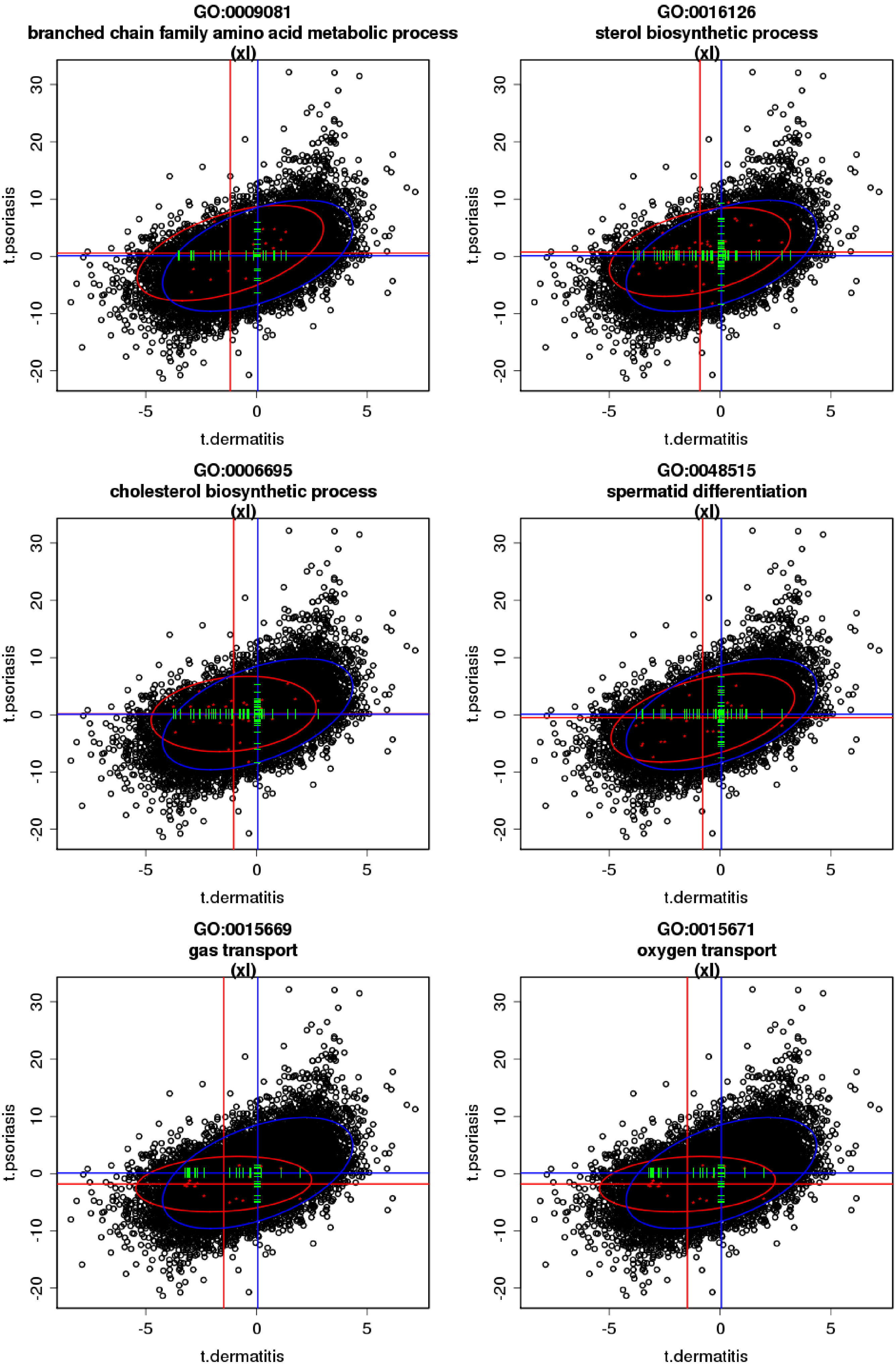
GO:0009081
branched chain family amino acid metabolic process
(xl)
GO:0016126
sterol biosynthetic process
(xl)
GO:0006695
cholesterol biosynthetic process
(xl)
GO:0048515
spermatid differentiation
(xl)
GO:0015669
gas transport
(xl)
GO:0015671
oxygen transport
(xl)
t.psoriasis
t.dermatitis
-20
-10
0
10
20
30
-5
0
5

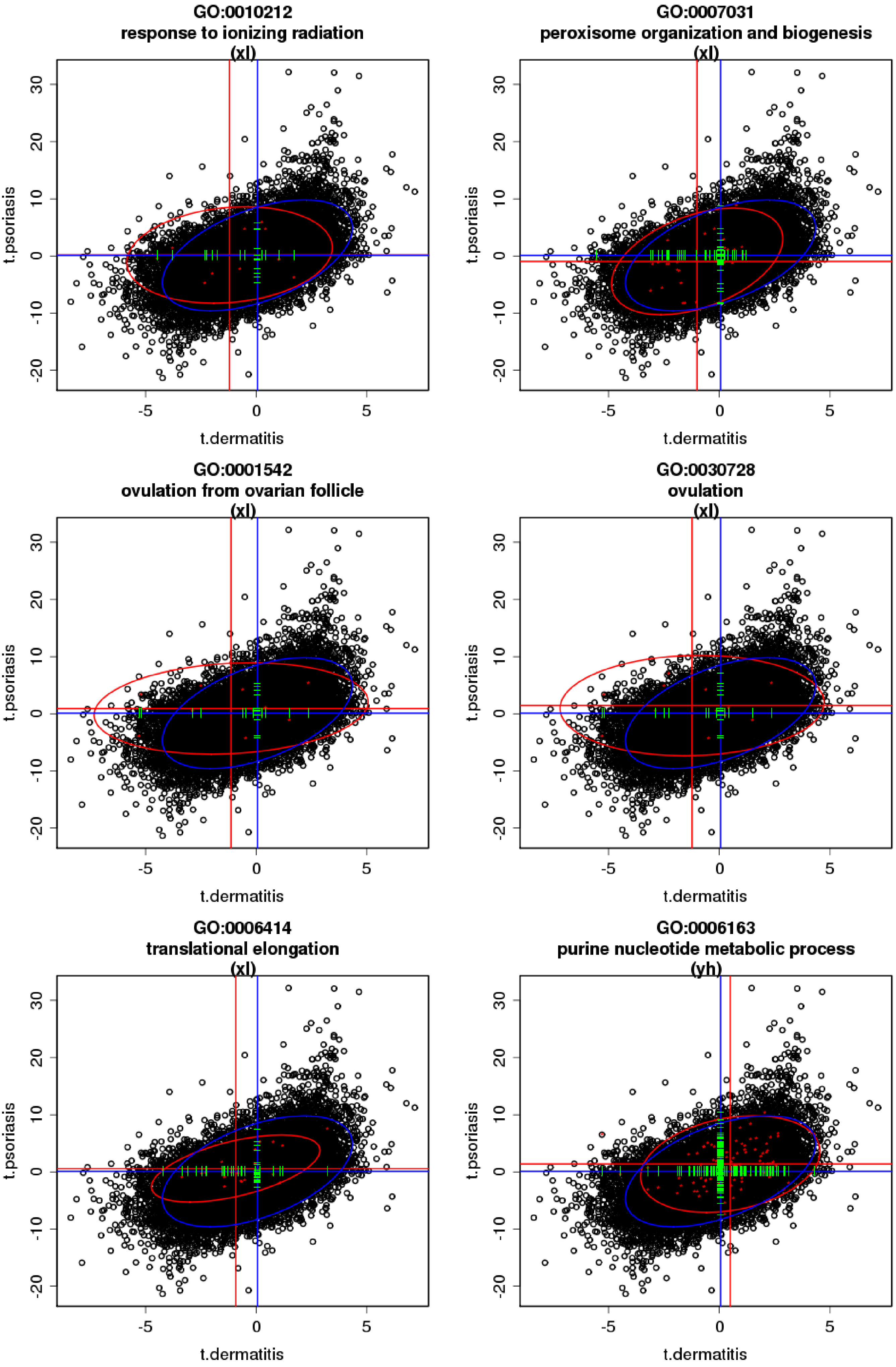

GO:0010212
response to ionizing radiation
(xl)
GO:0007031
peroxisome organization and biogenesis
(xl)
GO:0001542
ovulation from ovarian follicle
(xl)
GO:0030728
ovulation
(xl)
GO:0006414
translational elongation
(xl)
GO:0006163
purine nucleotide metabolic process
(yh)
t.psoriasis
t.dermatitis
-20
-10
0
10
20
30
-5
0
5

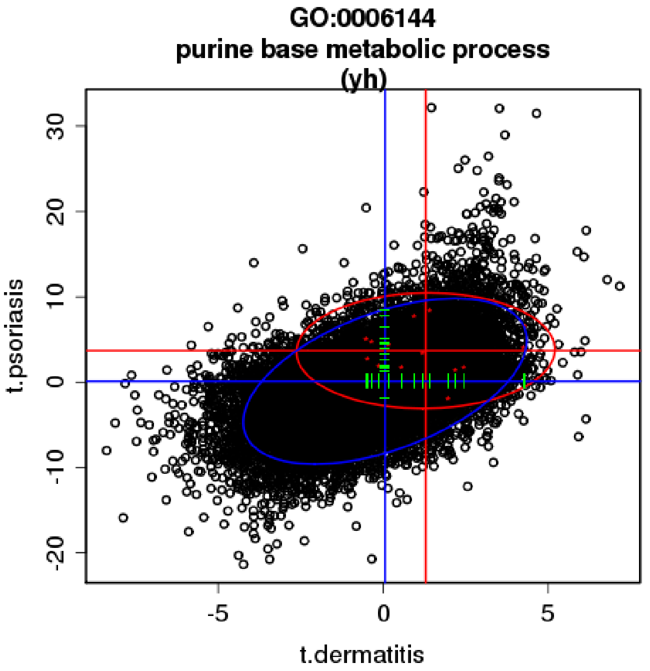
GO:0006144
purine base metabolic process
(yh)
t.psoriasis
t.dermatitis

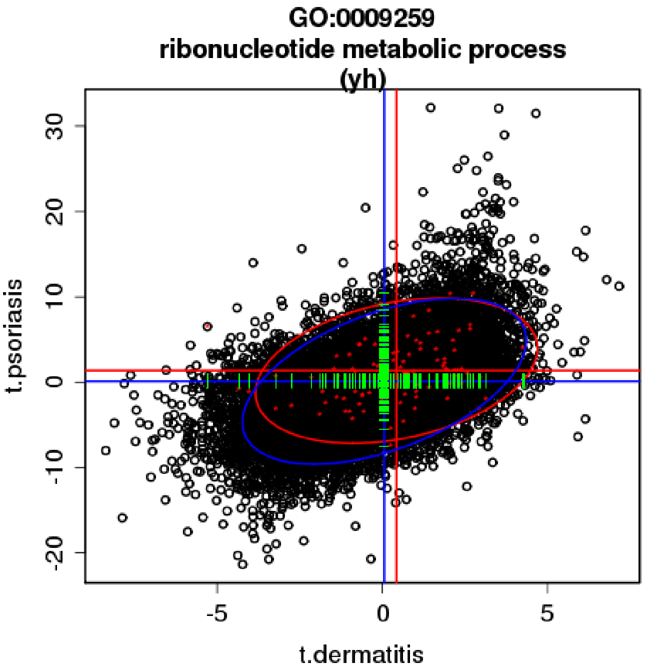
GO:0009259
ribonucleotide metabolic process
(yh)
t.psoriasis
t.dermatitis

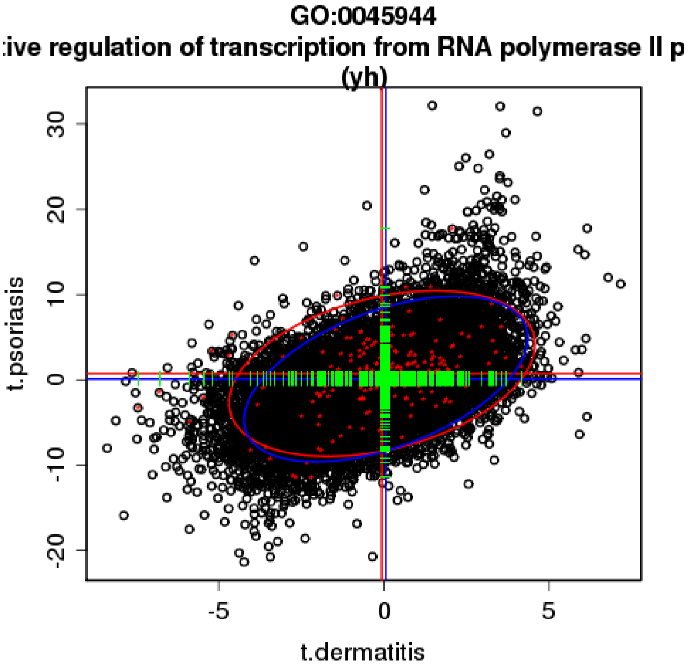
GO:0045944
:ive regulation of transcription from RNA polymerase II p
(yh)
t.psoriasis
t.dermatitis

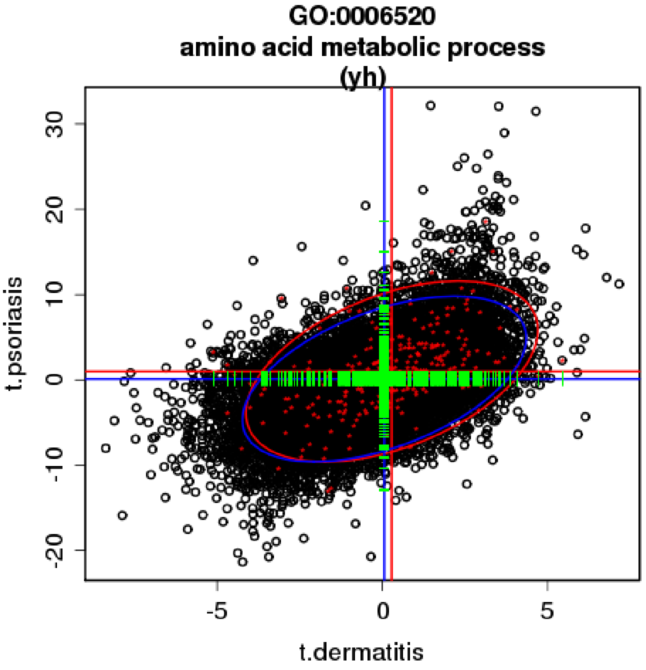
GO:0006520
amino acid metabolic process
(yh)
t.psoriasis
t.dermatitis

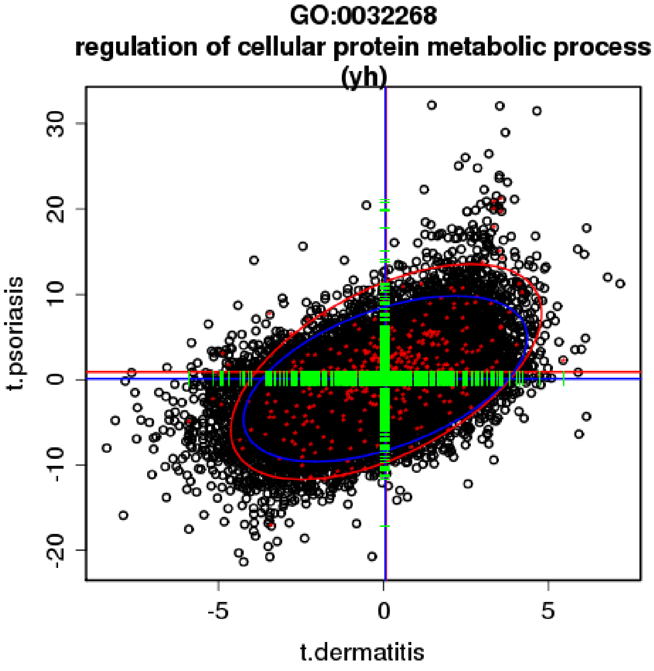
GO:0032268
regulation of cellular protein metabolic process
(yh)
t.psoriasis
t.dermatitis

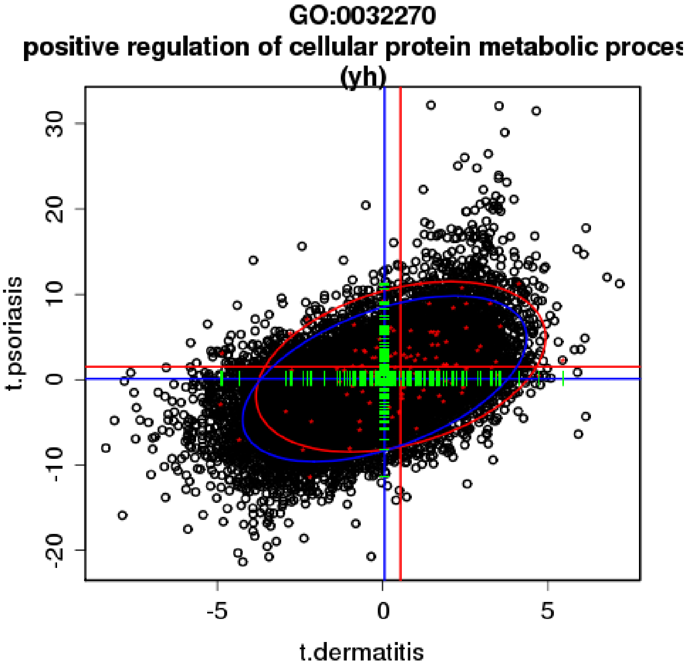
GO:0032270
positive regulation of cellular protein metabolic proces
(yh)
t.psoriasis
t.dermatitis

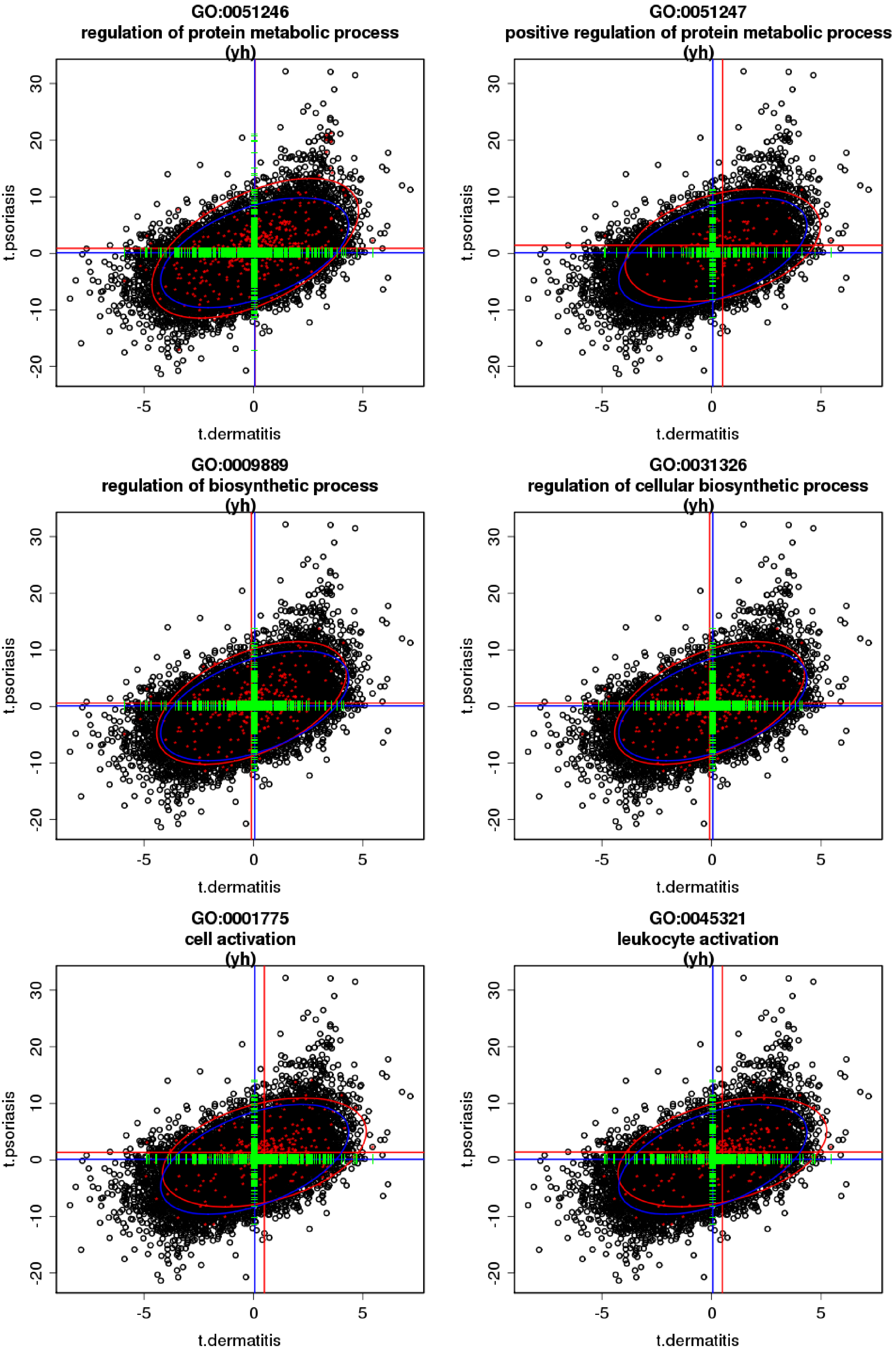
GO:0051246
regulation of protein metabolic process
(yh)
GO:0051247
positive regulation of protein metabolic process
(yh)
GO:0009889
regulation of biosynthetic process
(yh)
GO:0031326
regulation of cellular biosynthetic process
(yh)
GO:0001775
cell activation
(yh)
GO:0045321
leukocyte activation
(yh)
t.psoriasis
t.dermatitis
-20
-10
0
10
20
30
-5
0
5

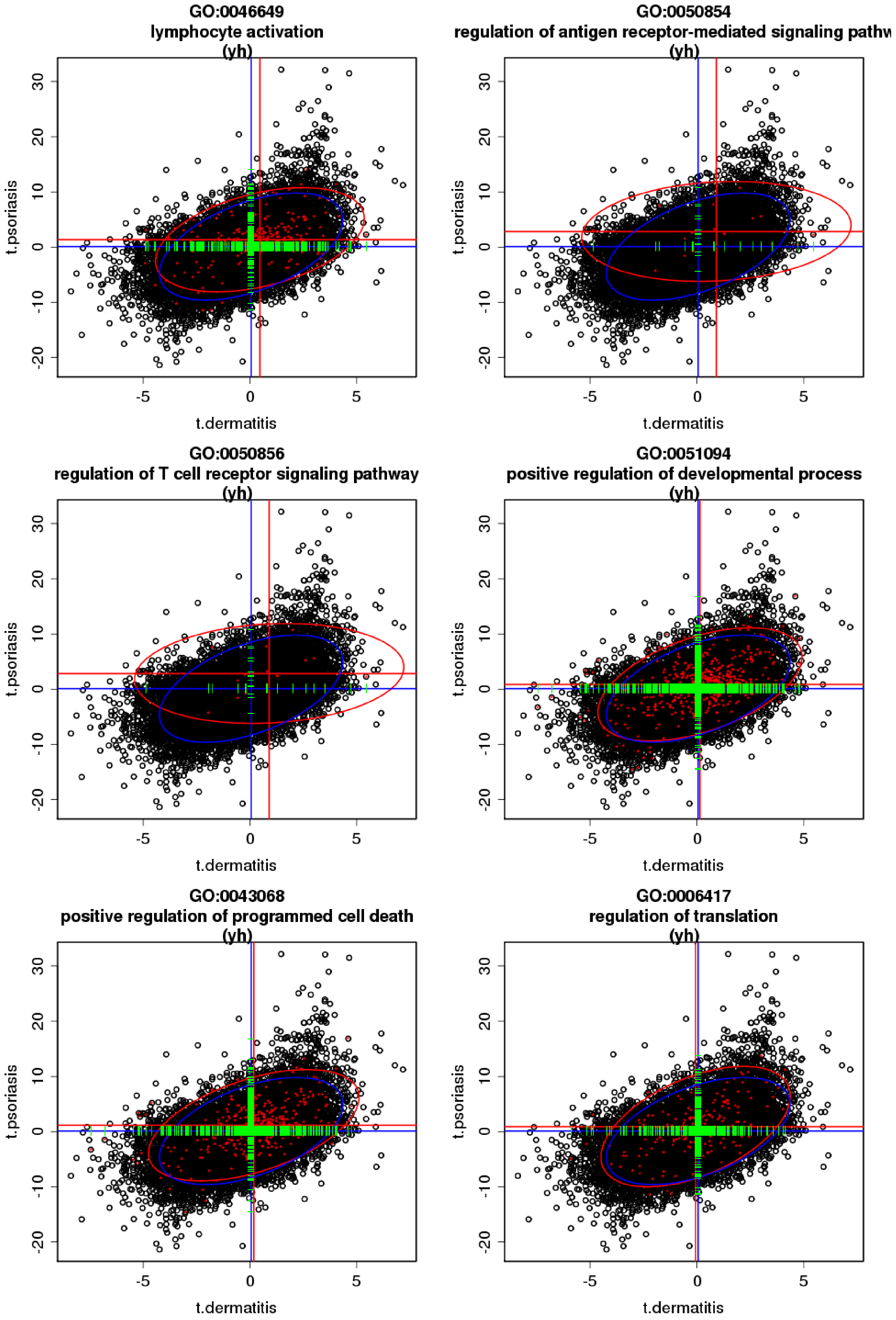
GO:0046649
lymphocyte activation
(yh)
t.psoriasis
t.dermatitis
GO:0050854
regulation of antigen receptor-mediated signaling pathw
(yh)
t.psoriasis
t.dermatitis
GO:0050856
regulation of T cell receptor signaling pathway
(yh)
t.psoriasis
t.dermatitis
GO:0051094
positive regulation of developmental process
(yh)
t.psoriasis
t.dermatitis
GO:0043068
positive regulation of programmed cell death
(yh)
t.psoriasis
t.dermatitis
GO:0006417
regulation of translation
(yh)
t.psoriasis
t.dermatitis

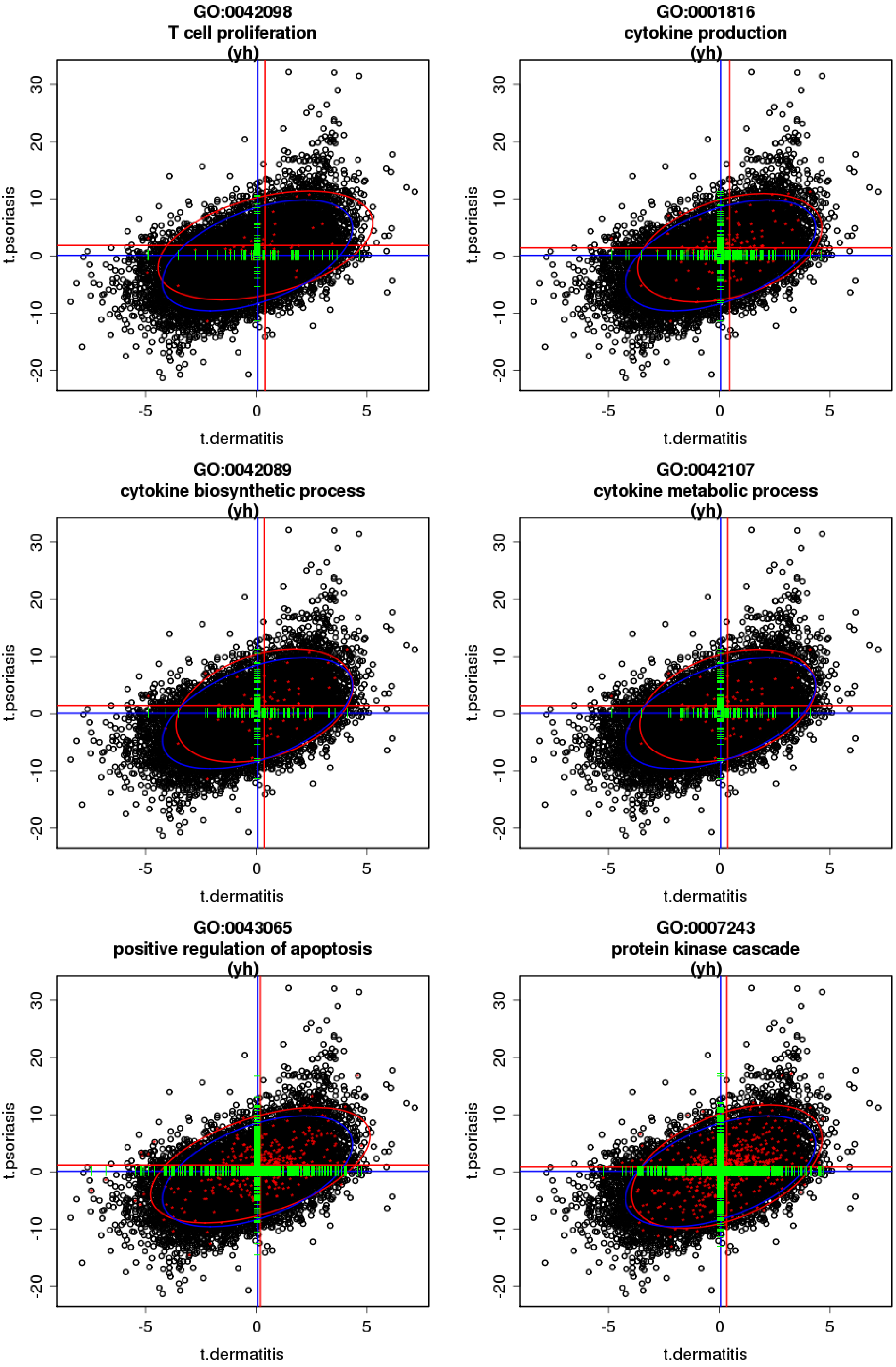
GO:0042098
T cell proliferation
(yh)
GO:0001816
cytokine production
(yh)
GO:0042089
cytokine biosynthetic process
(yh)
GO:0042107
cytokine metabolic process
(yh)
GO:0043065
positive regulation of apoptosis
(yh)
GO:0007243
protein kinase cascade
(yh)
t.psoriasis
t.dermatitis

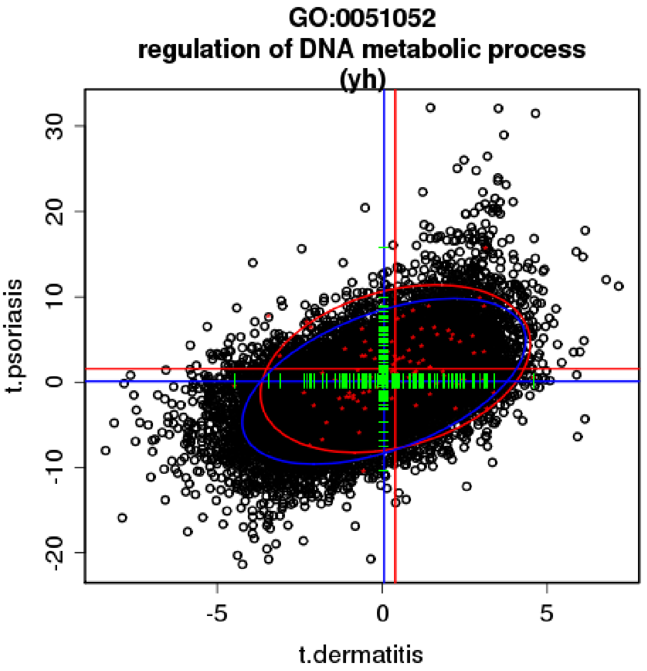
GO:0051052
regulation of DNA metabolic process
(yh)
t.psoriasis
t.dermatitis
-5
0
5
-20
-10
0
10
20
30

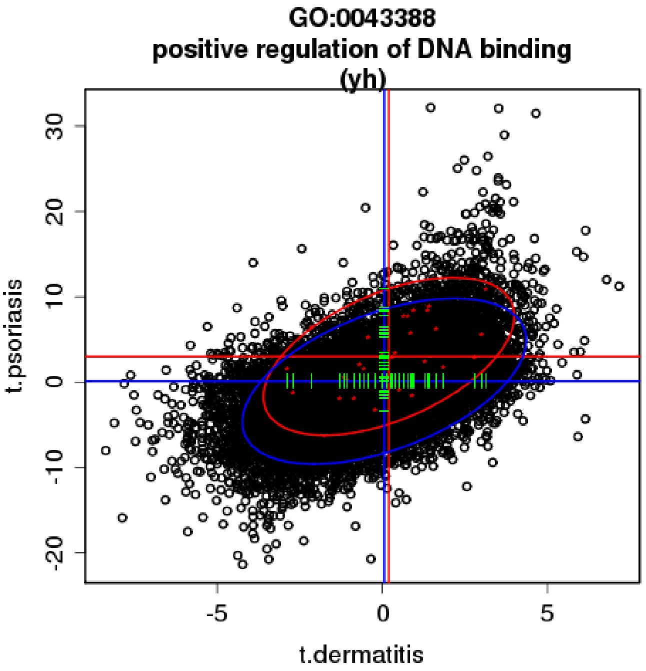
GO:0043388
positive regulation of DNA binding
(yh)
t.psoriasis
t.dermatitis
-5
0
5
-20
-10
0
10
20
30

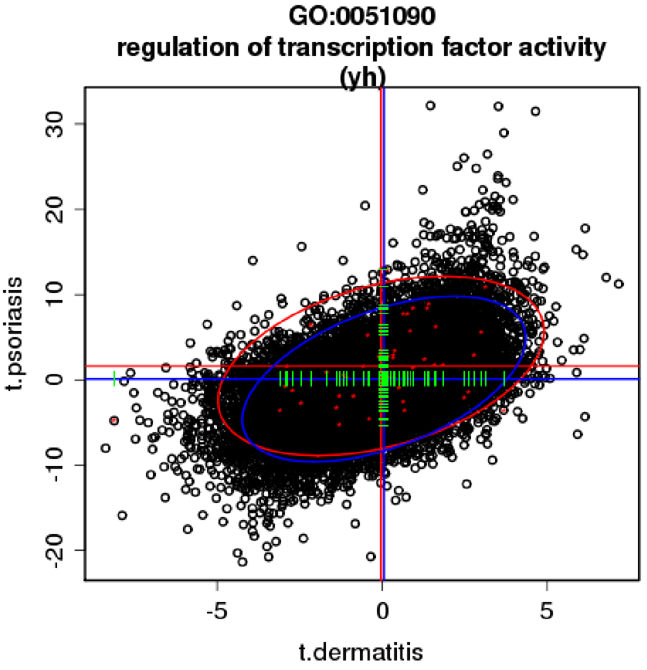
GO:0051090
regulation of transcription factor activity
(yh)
t.psoriasis
t.dermatitis
-5
0
5
-20
-10
0
10
20
30

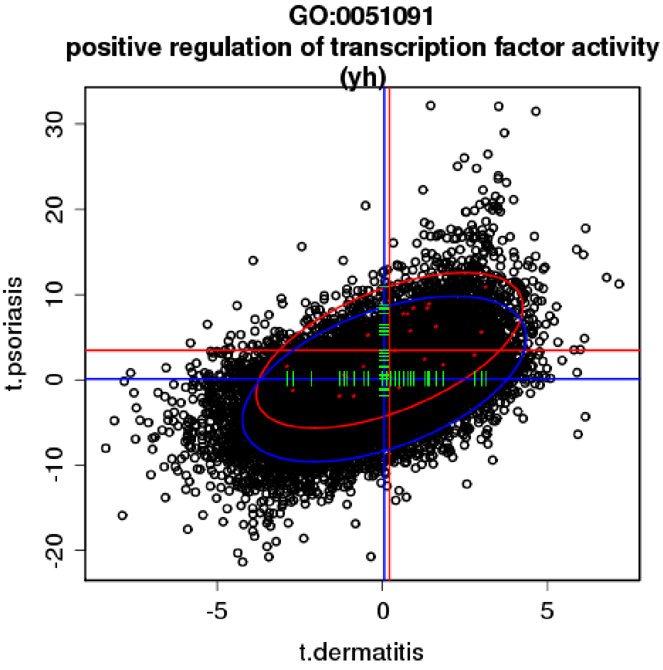
GO:0051091
positive regulation of transcription factor activity
(yh)
t.psoriasis
t.dermatitis
-5
0
5
-20
-10
0
10
20
30

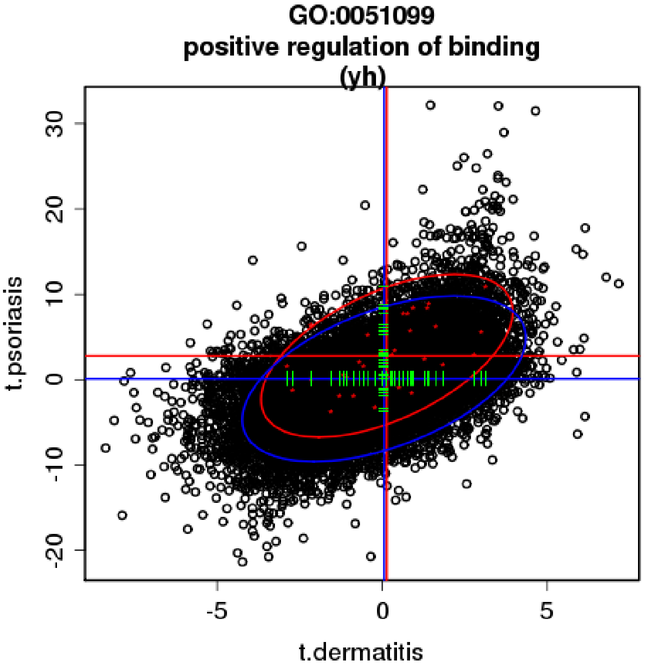
GO:0051099
positive regulation of binding
(yh)
t.psoriasis
t.dermatitis
-5
0
5
-20
-10
0
10
20
30

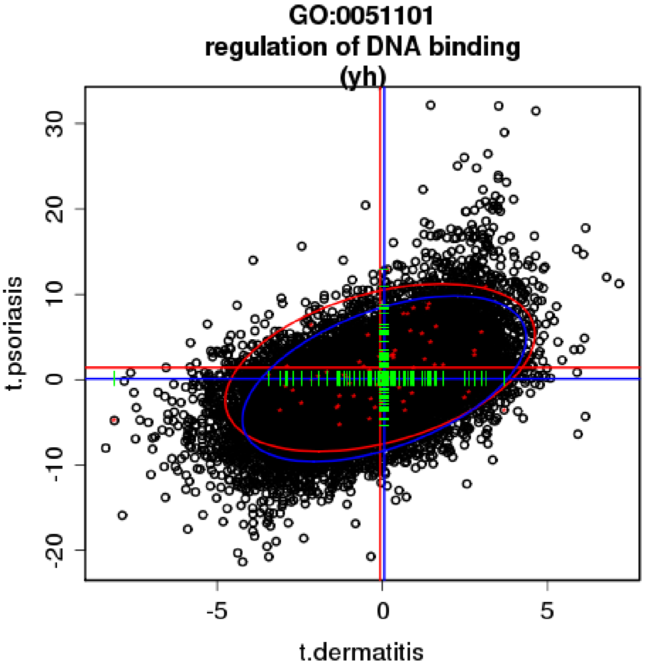
GO:0051101
regulation of DNA binding
(yh)
t.psoriasis
t.dermatitis
-5
0
5
-20
-10
0
10
20
30

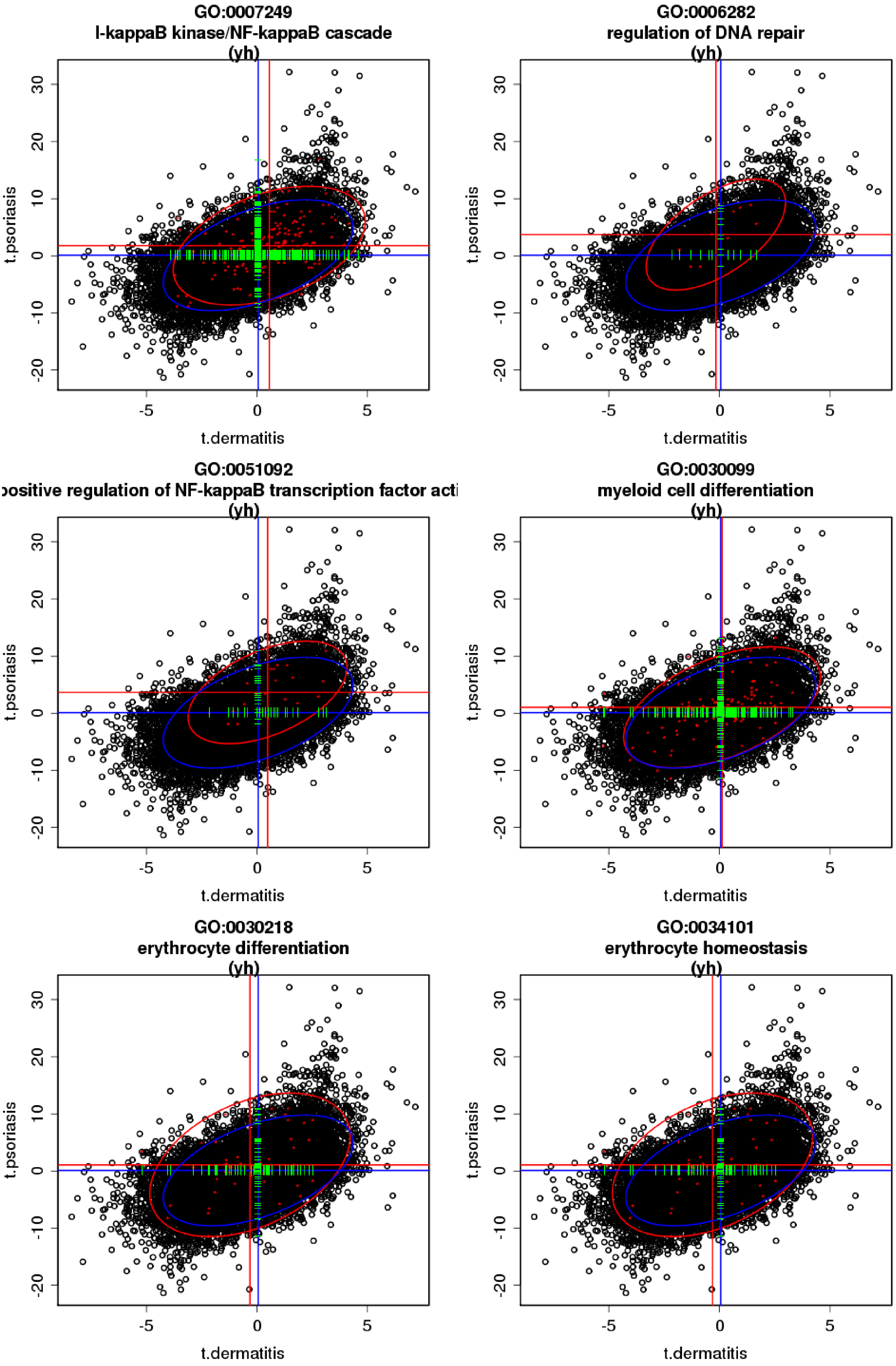
GO:0007249
I-kappaB kinase/NF-kappaB cascade
(yh)
GO:0006282
regulation of DNA repair
(yh)
GO:0051092
ositive regulation of NF-kappaB transcription factor acti
(yh)
GO:0030099
myeloid cell differentiation
(yh)
GO:0030218
erythrocyte differentiation
(yh)
GO:0034101
erythrocyte homeostasis
(yh)
t.psoriasis
t.dermatitis
30
20
10
0
-10
-20
-5
0
5

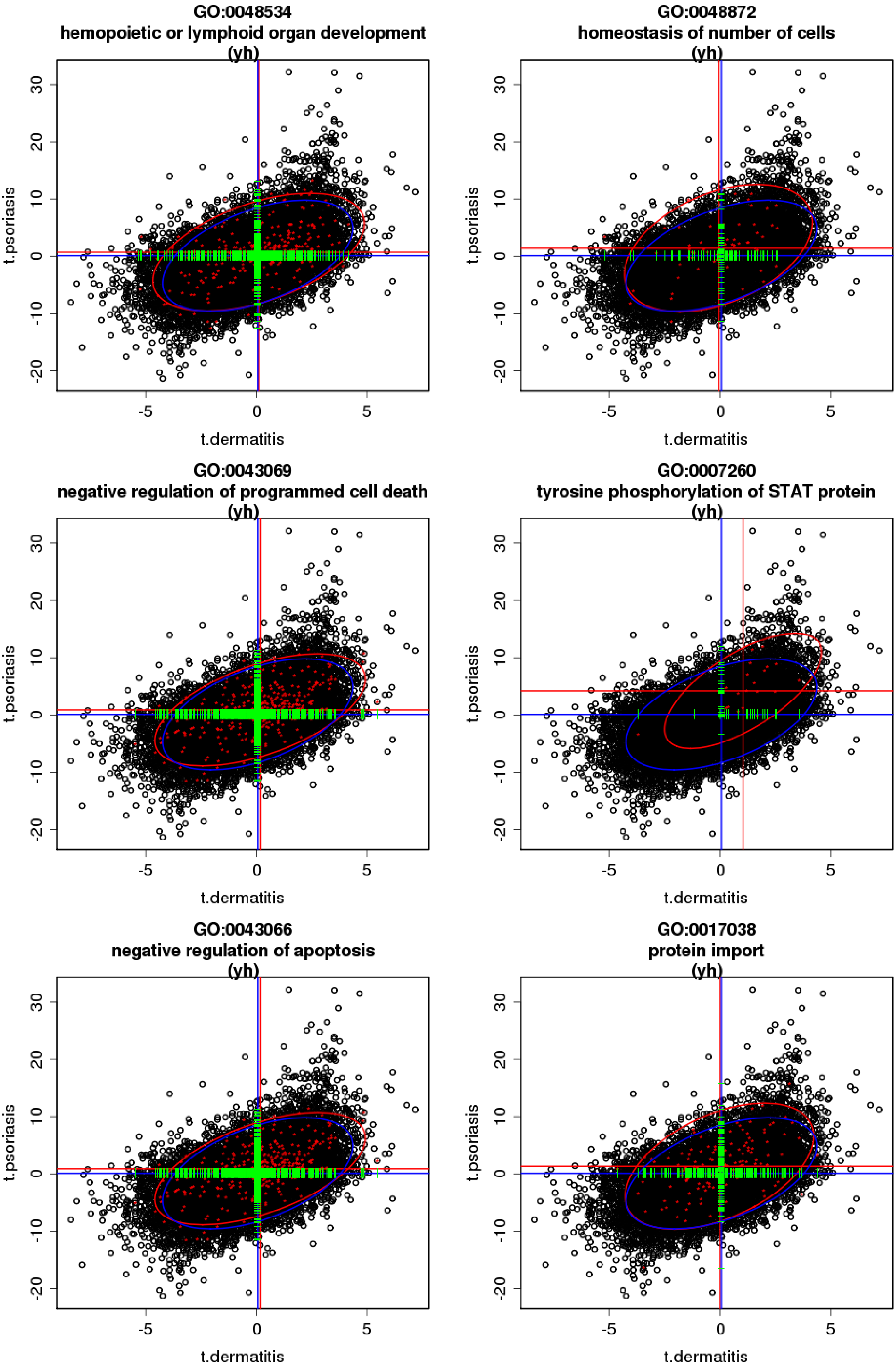
GO:0048534
hemopoietic or lymphoid organ development
(yh)
GO:0048872
homeostasis of number of cells
(yh)
GO:0043069
negative regulation of programmed cell death
(yh)
GO:0007260
tyrosine phosphorylation of STAT protein
(yh)
GO:0043066
negative regulation of apoptosis
(yh)
GO:0017038
protein import
(yh)
t.psoriasis
t.dermatitis
-20
-10
0
10
20
30
-5
0
5

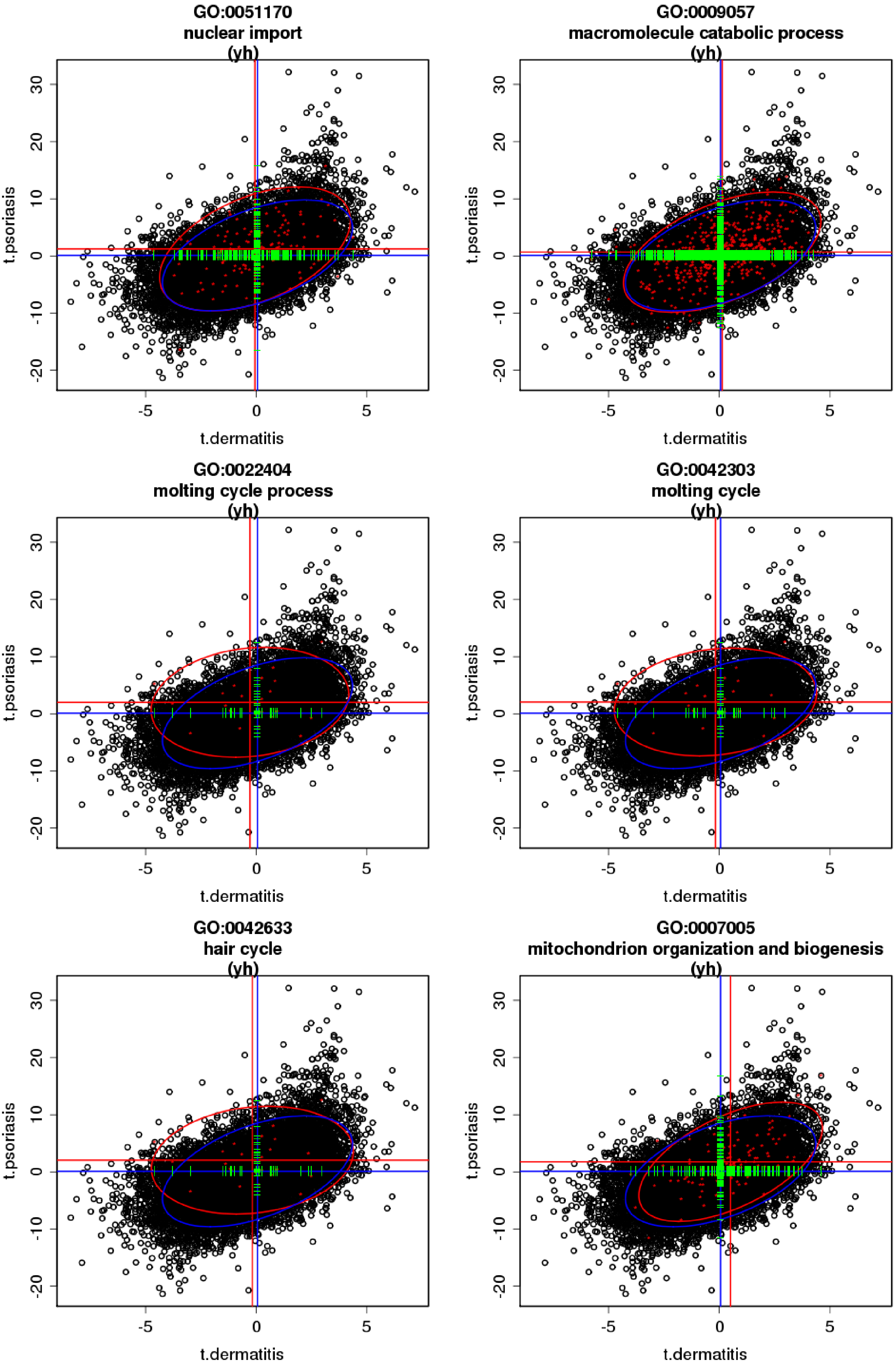
GO:0051170
nuclear import
(yh)
t.psoriasis
t.dermatitis
GO:0009057
macromolecule catabolic process
(yh)
t.psoriasis
t.dermatitis
GO:0022404
molting cycle process
(yh)
t.psoriasis
t.dermatitis
GO:0042303
molting cycle
(yh)
t.psoriasis
t.dermatitis
GO:0042633
hair cycle
(yh)
t.psoriasis
t.dermatitis
GO:0007005
mitochondrion organization and biogenesis
(yh)
t.psoriasis
t.dermatitis

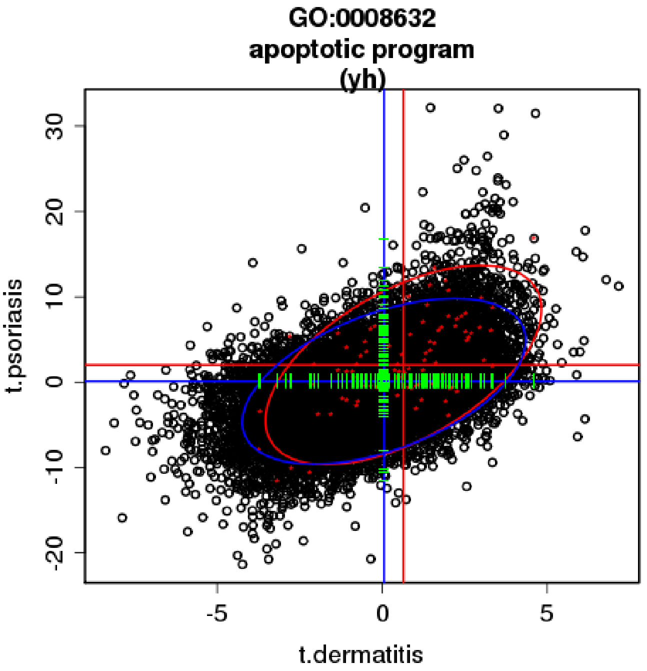
GO:0008632
apoptotic program
(yh)
t.psoriasis
t.dermatitis

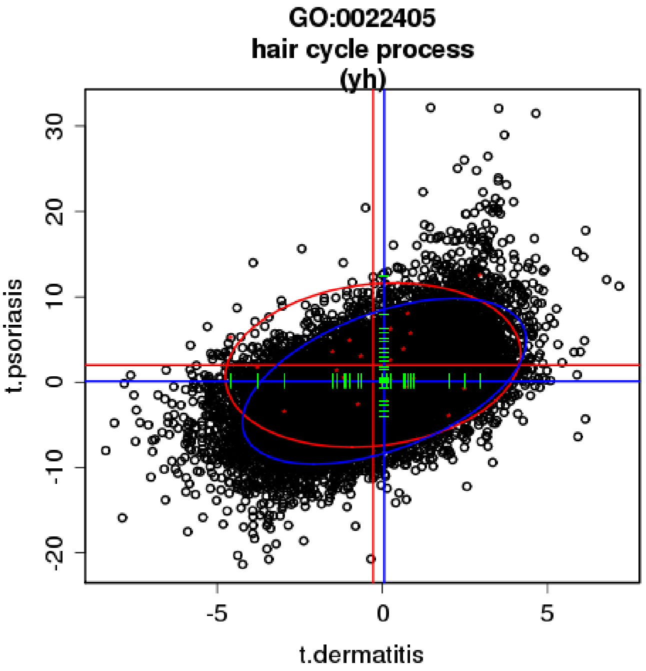
GO:0022405
hair cycle process
(yh)
t.psoriasis
t.dermatitis

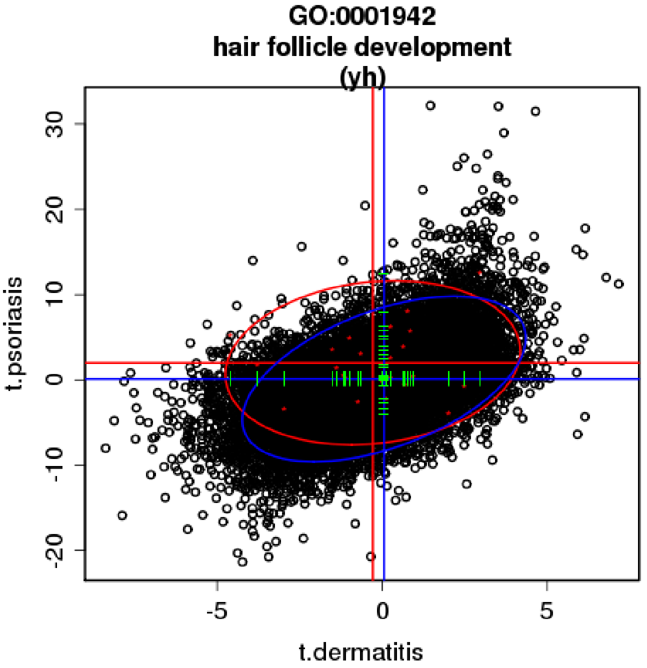
GO:0001942
hair follicle development
(yh)
t.psoriasis
t.dermatitis

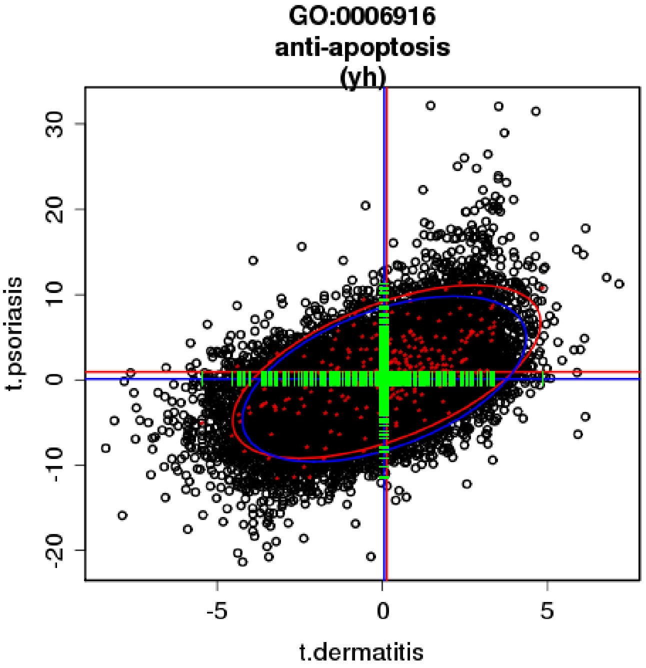
GO:0006916
anti-apoptosis
(yh)
t.psoriasis
t.dermatitis

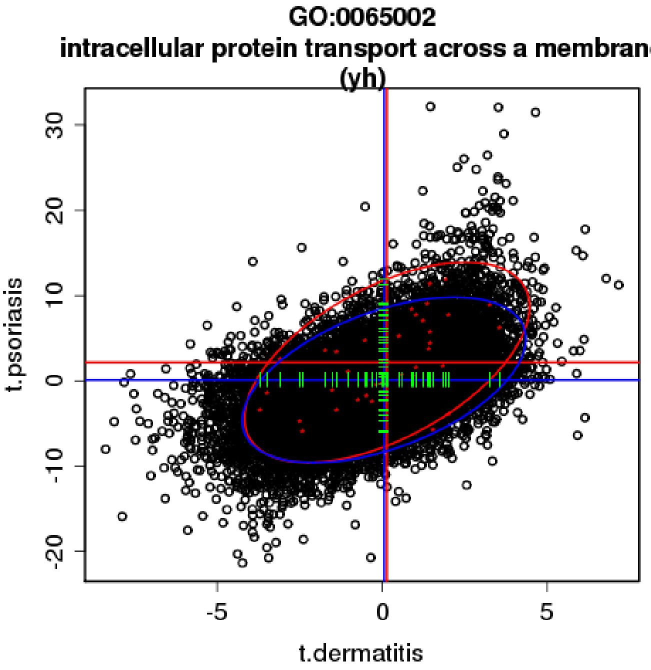
GO:0065002
intracellular protein transport across a membrane
(yh)
t.psoriasis
t.dermatitis

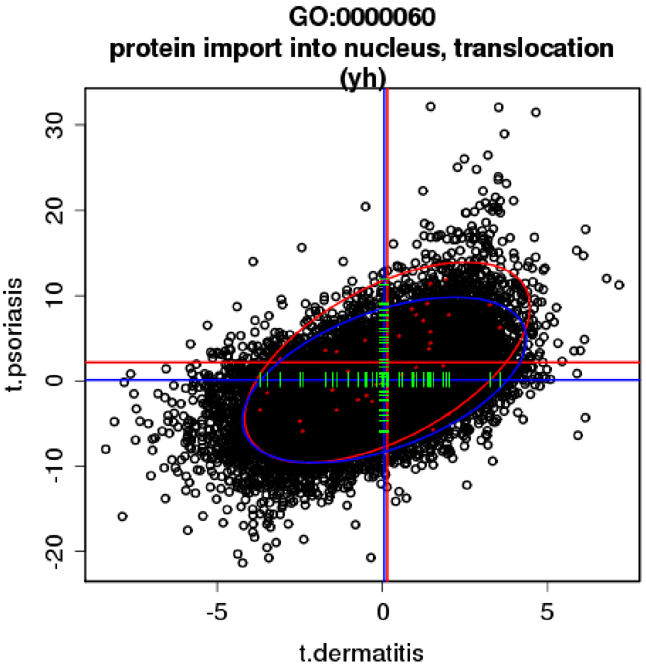
GO:0000060
protein import into nucleus, translocation
(yh)
t.psoriasis
t.dermatitis

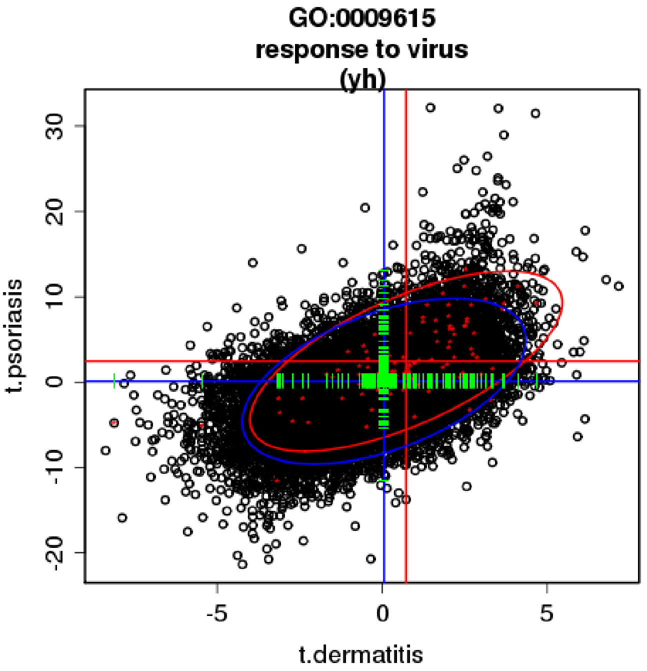
GO:0009615
response to virus
(yh)
t.psoriasis
t.dermatitis

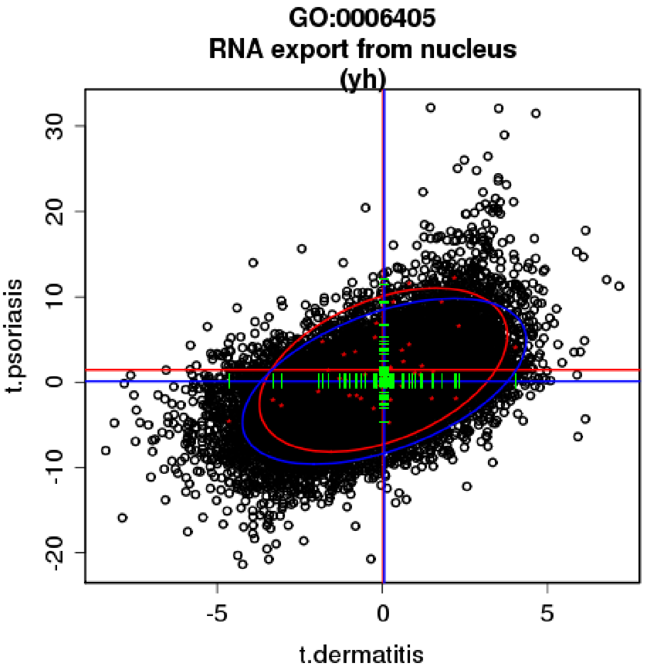
GO:0006405
RNA export from nucleus
(yh)
t.psoriasis
t.dermatitis

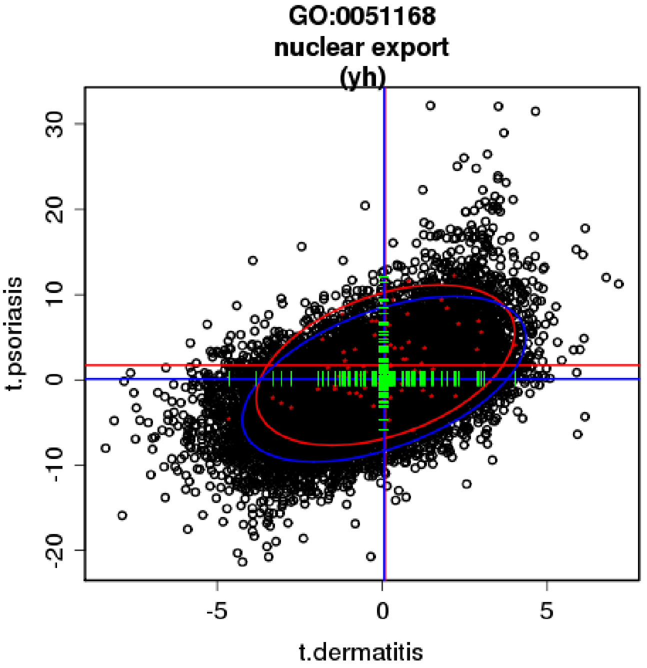
GO:0051168
nuclear export
(yh)
t.psoriasis
t.dermatitis

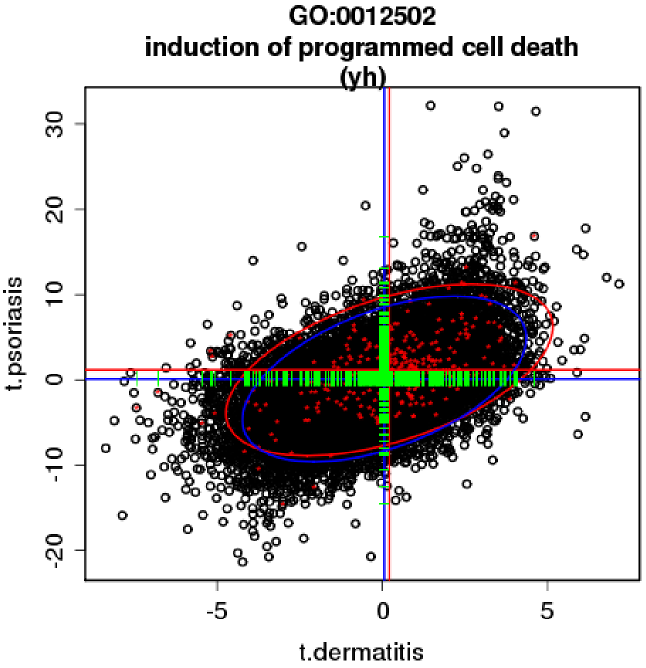
GO:0012502
induction of programmed cell death
(yh)
t.psoriasis
t.dermatitis

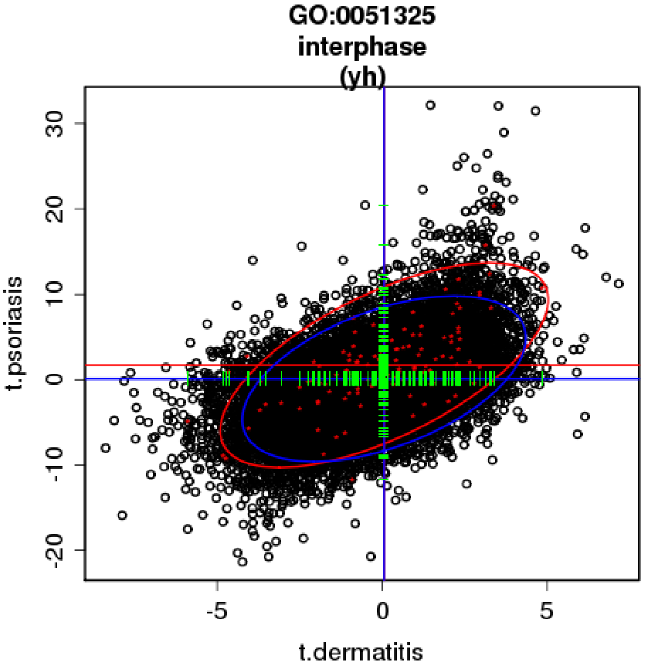
GO:0051325
interphase
(yh)
t.psoriasis
t.dermatitis

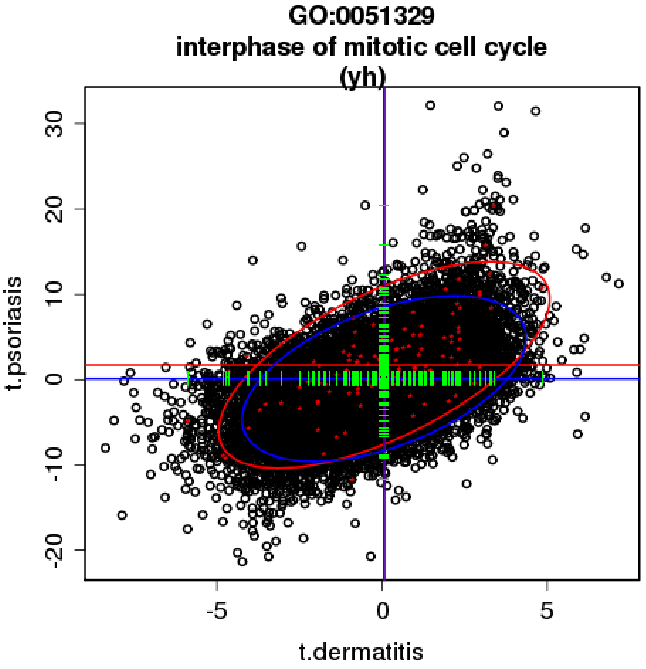
GO:0051329
interphase of mitotic cell cycle
(yh)
t.psoriasis
t.dermatitis

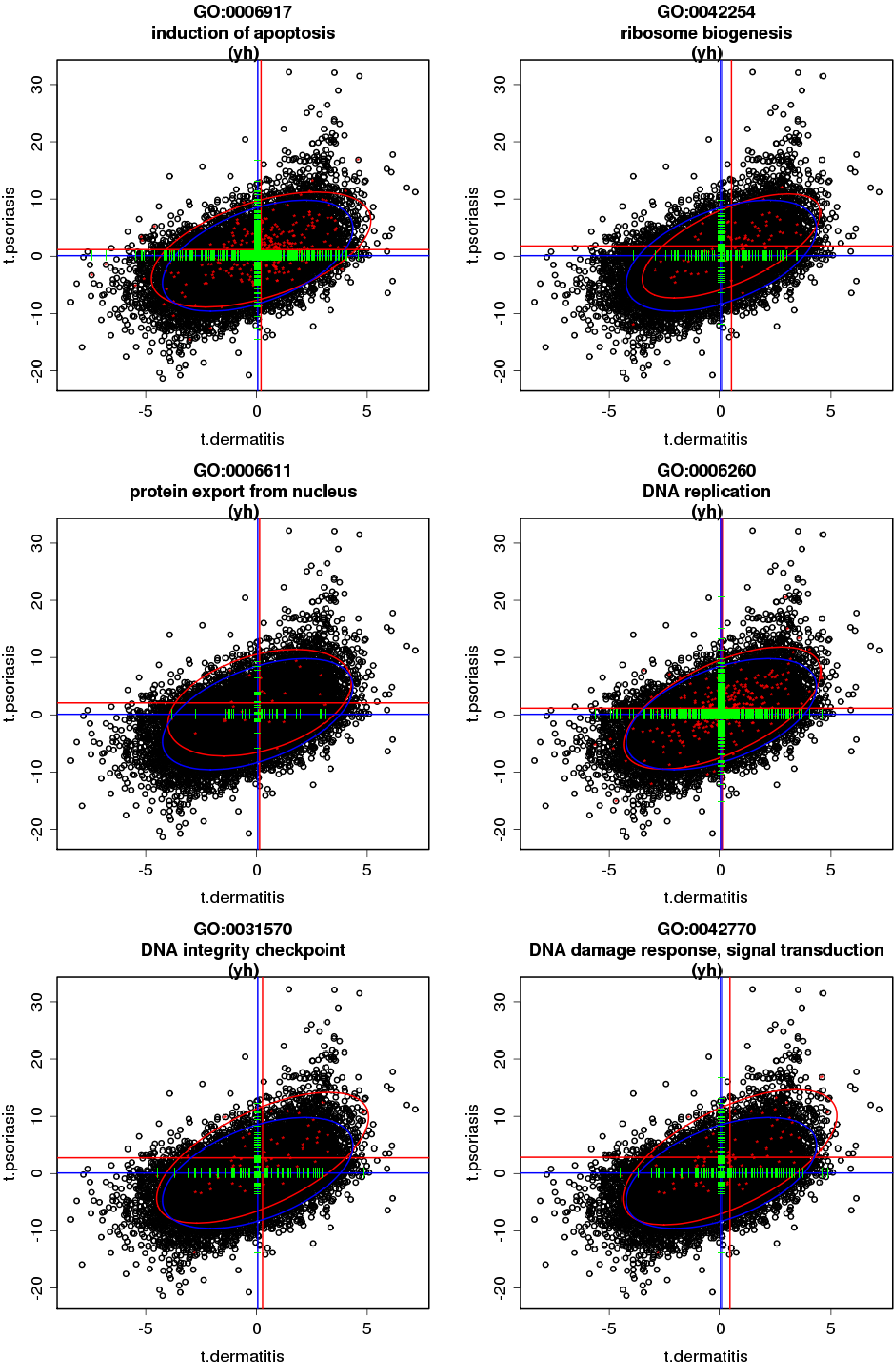
GO:0006917
induction of apoptosis
(yh)
GO:0042254
ribosome biogenesis
(yh)
GO:0006611
protein export from nucleus
(yh)
GO:0006260
DNA replication
(yh)
GO:0031570
DNA integrity checkpoint
(yh)
GO:0042770
DNA damage response, signal transduction
(yh)
t.psoriasis
t.dermatitis
-20
-10
0
10
20
30
-5
0
5

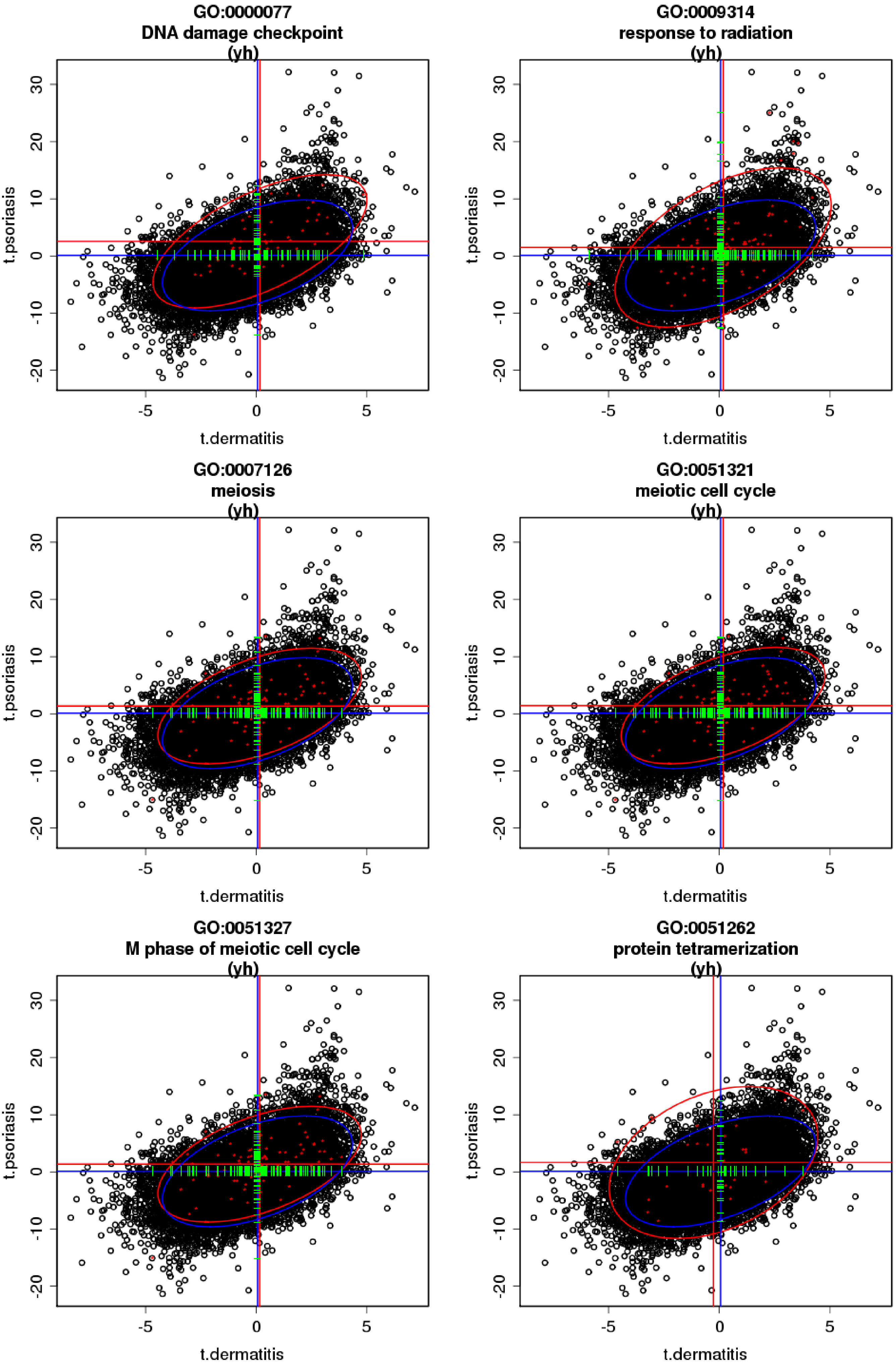
GO:0000077
DNA damage checkpoint
(yh)
GO:0009314
response to radiation
(yh)
GO:0007126
meiosis
(yh)
GO:0051321
meiotic cell cycle
(yh)
GO:0051327
M phase of meiotic cell cycle
(yh)
GO:0051262
protein tetramerization
(yh)
t.psoriasis
t.dermatitis
-20
-10
0
10
20
30
-5
0
5

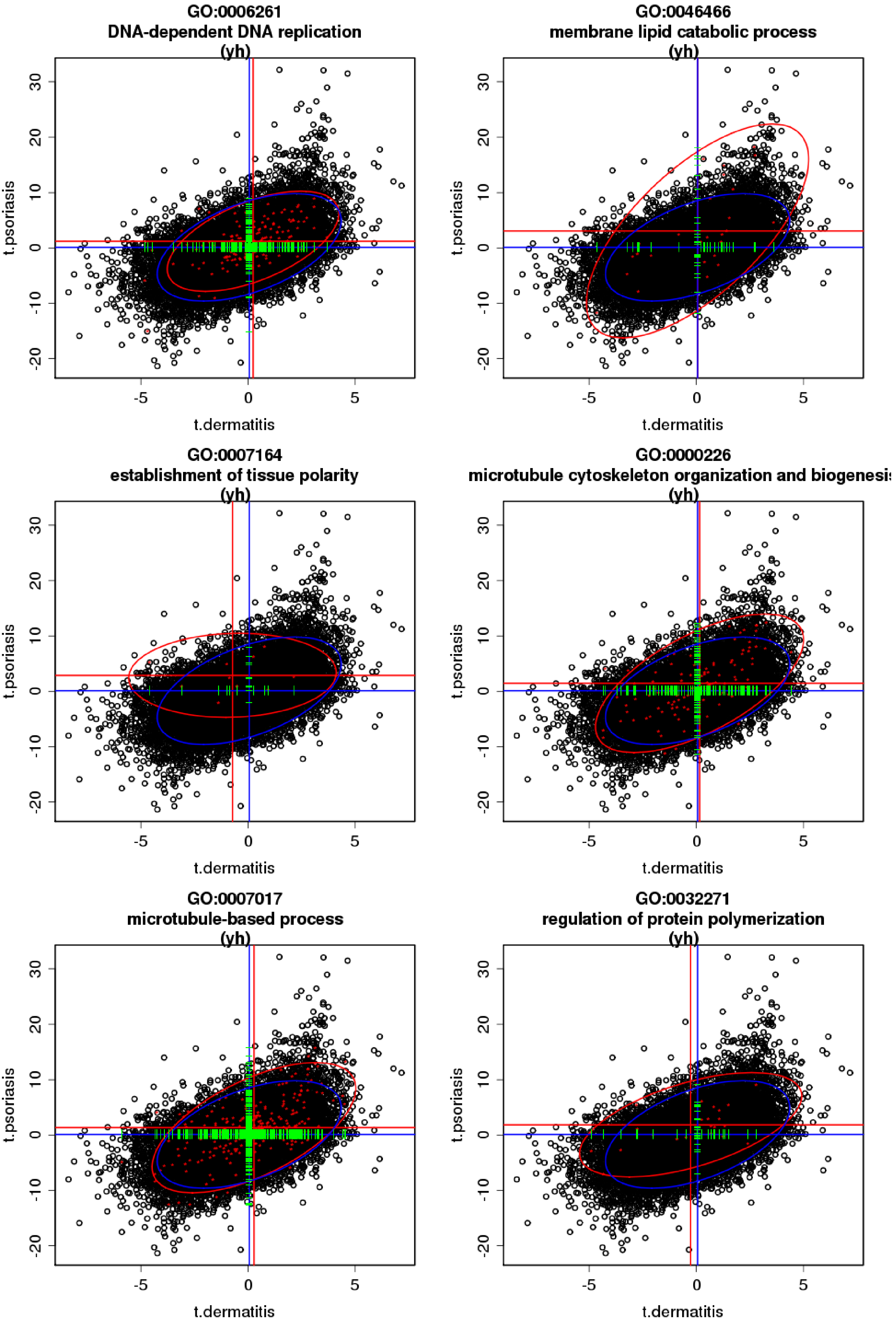
GO:0006261
DNA-dependent DNA replication
(yh)
GO:0046466
membrane lipid catabolic process
(yh)
GO:0007164
establishment of tissue polarity
(yh)
GO:0000226
microtubule cytoskeleton organization and biogenesi
(yh)
GO:0007017
microtubule-based process
(yh)
GO:0032271
regulation of protein polymerization
(yh)
t.psoriasis
t.dermatitis
-20
-10
0
10
20
30
-5
0
5

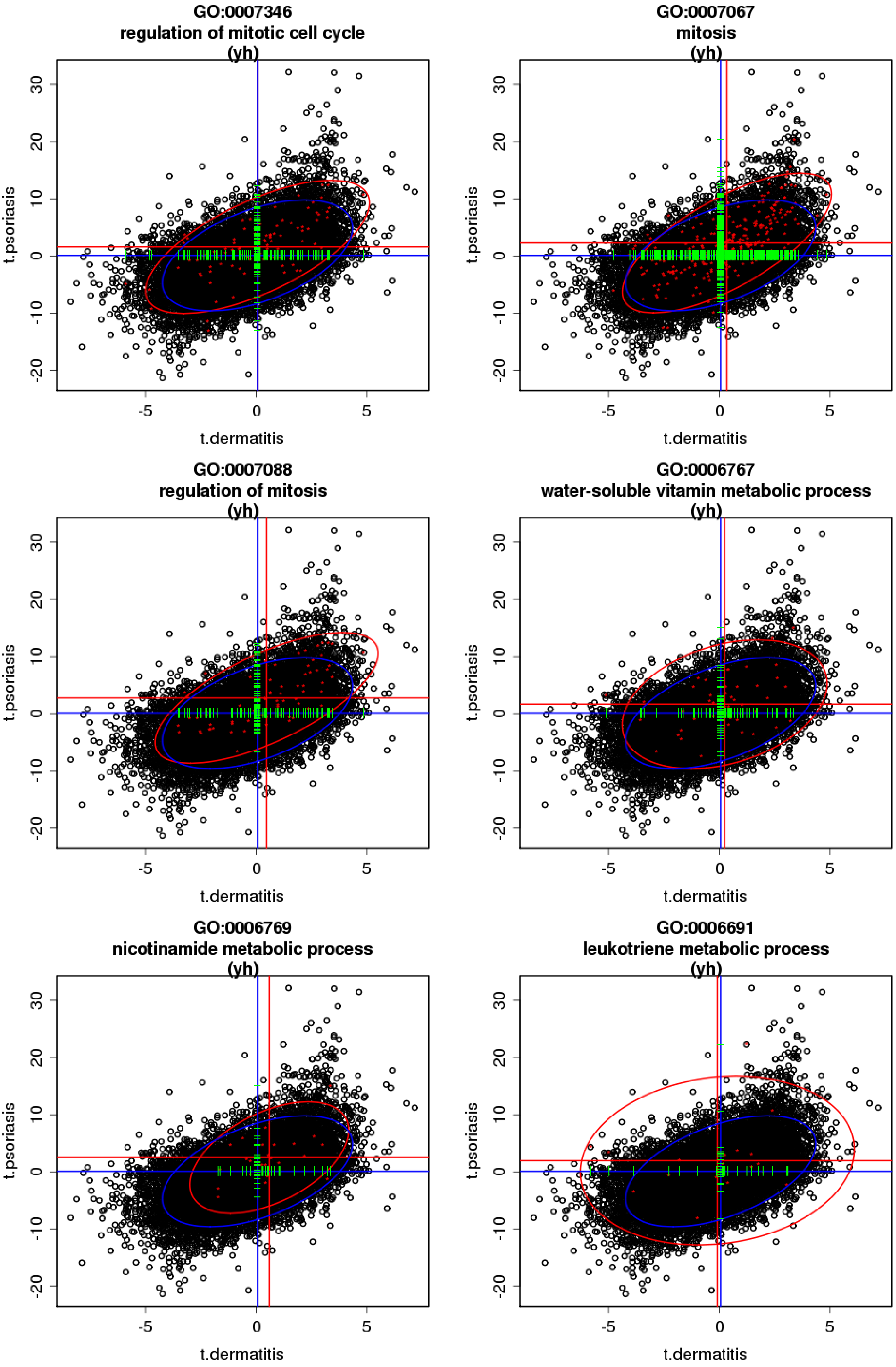
GO:0007346
regulation of mitotic cell cycle
(yh)
GO:0007067
mitosis
(yh)
GO:0007088
regulation of mitosis
(yh)
GO:0006767
water-soluble vitamin metabolic process
(yh)
GO:0006769
nicotinamide metabolic process
(yh)
GO:0006691
leukotriene metabolic process
(yh)
t.psoriasis
t.dermatitis
-20
-10
0
10
20
30
-5
0
5

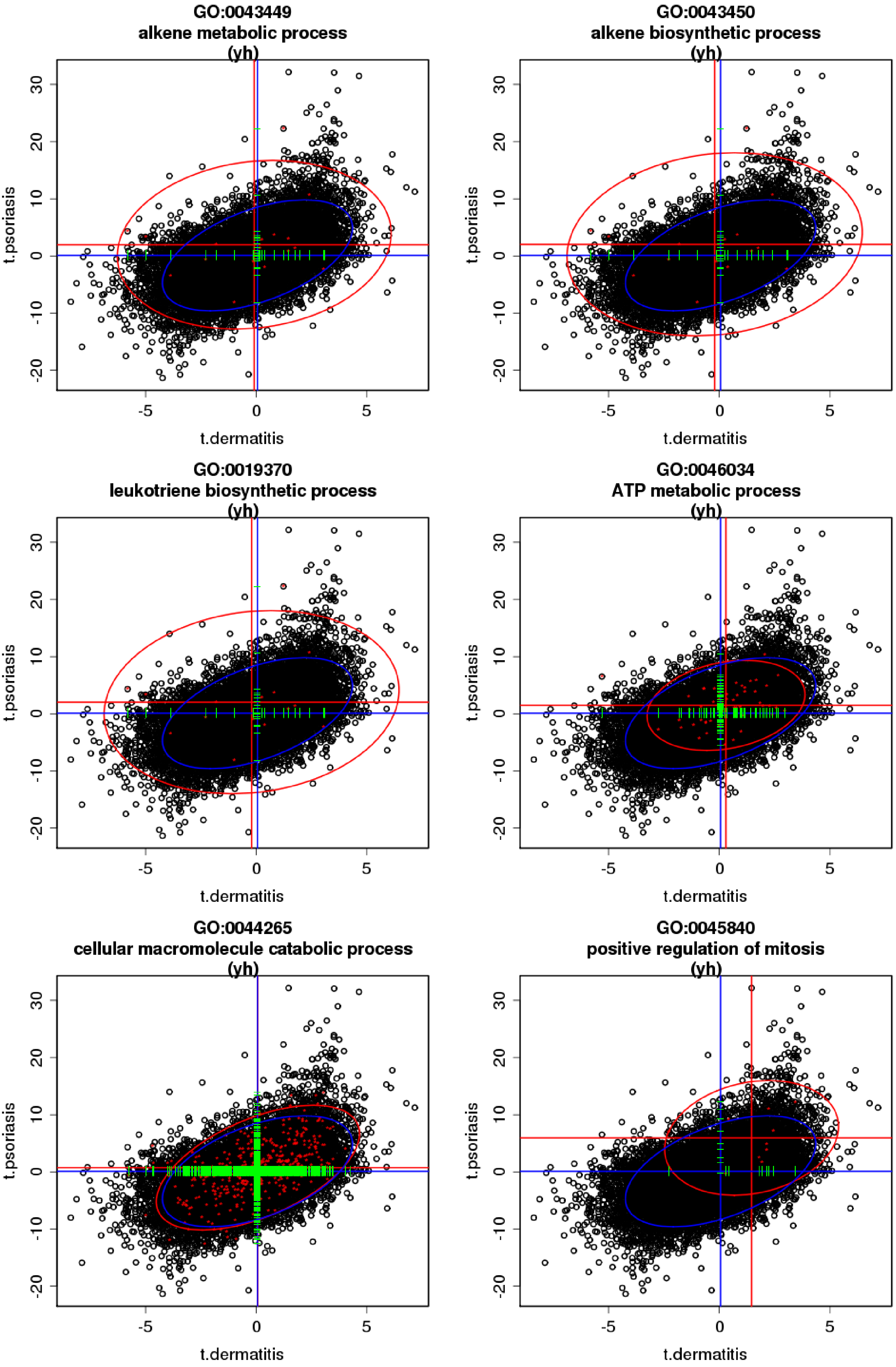
GO:0043449
alkene metabolic process
(yh)
t.psoriasis
t.dermatitis
GO:0043450
alkene biosynthetic process
(yh)
t.psoriasis
t.dermatitis
GO:0019370
leukotriene biosynthetic process
(yh)
t.psoriasis
t.dermatitis
GO:0046034
ATP metabolic process
(yh)
t.psoriasis
t.dermatitis
GO:0044265
cellular macromolecule catabolic process
(yh)
t.psoriasis
t.dermatitis
GO:0045840
positive regulation of mitosis
(yh)
t.psoriasis
t.dermatitis
-20
-10
0
10
20
30
-5
0
5

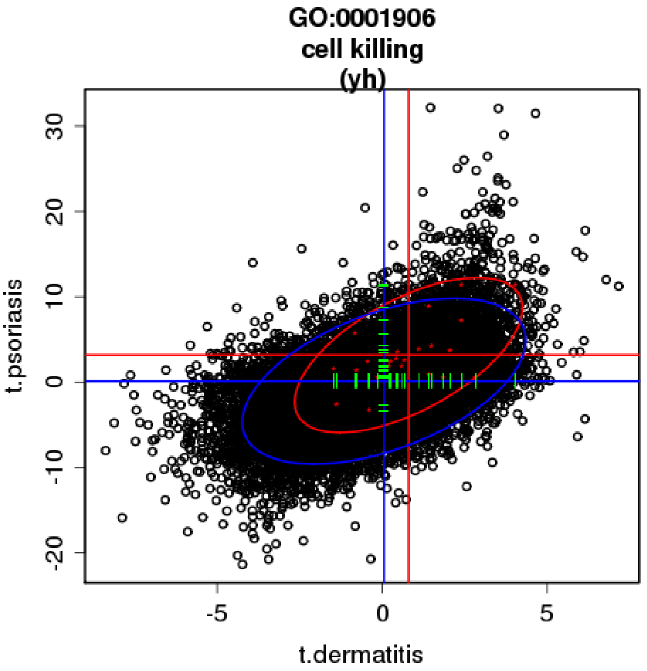
GO:0001906
cell killing
(yh)
t.psoriasis
t.dermatitis
30
20
10
0
-10
-20
-5
0
5

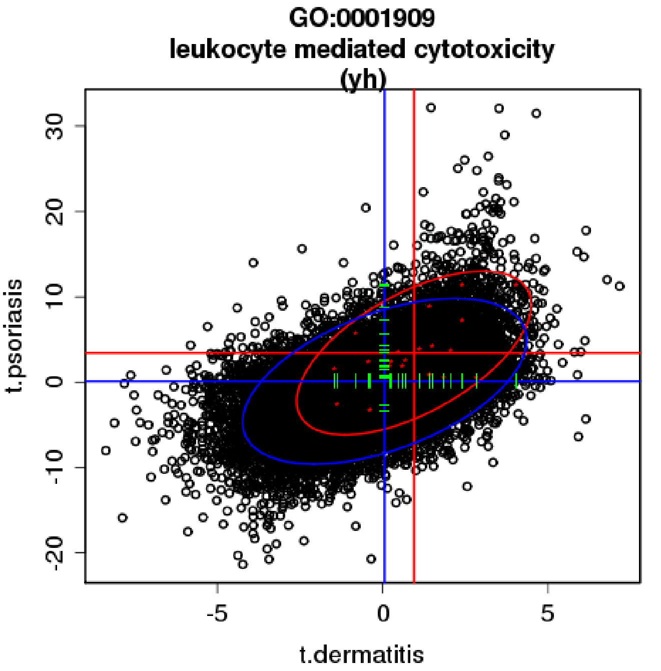
GO:0001909
leukocyte mediated cytotoxicity
(yh)
t.psoriasis
t.dermatitis
30
20
10
0
-10
-20
-5
0
5

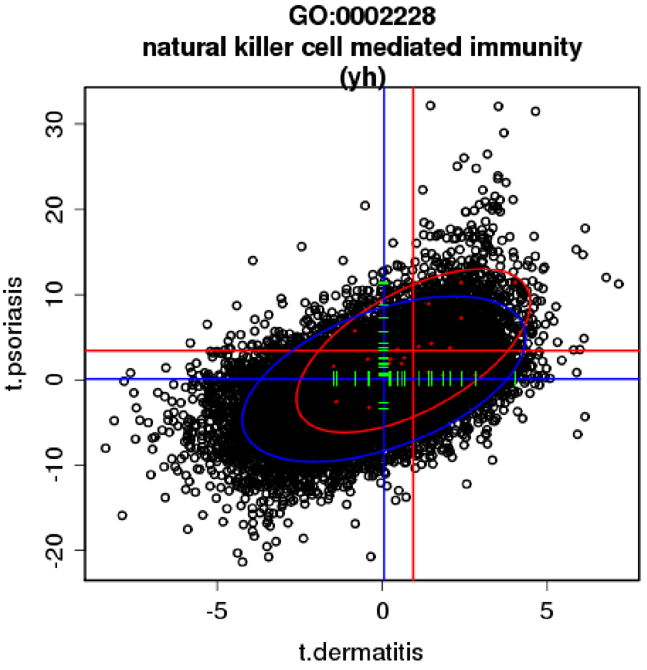
GO:0002228
natural killer cell mediated immunity
(yh)
t.psoriasis
t.dermatitis
30
20
10
0
-10
-20
-5
0
5

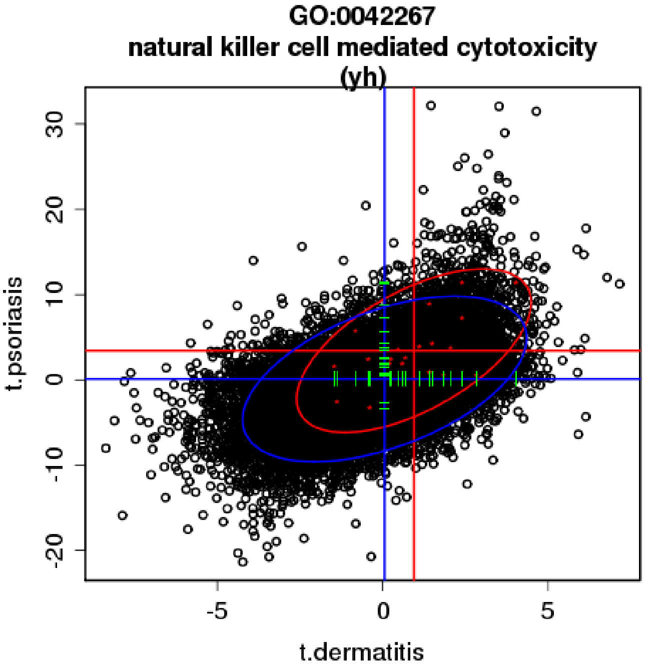
GO:0042267
natural killer cell mediated cytotoxicity
(yh)
t.psoriasis
t.dermatitis
30
20
10
0
-10
-20
-5
0
5

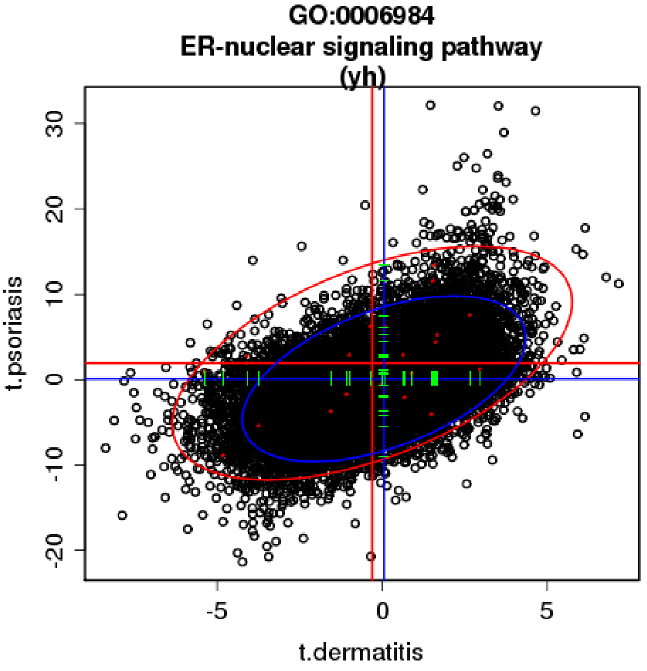
GO:0006984
ER-nuclear signaling pathway
(yh)
t.psoriasis
t.dermatitis
30
20
10
0
-10
-20
-5
0
5

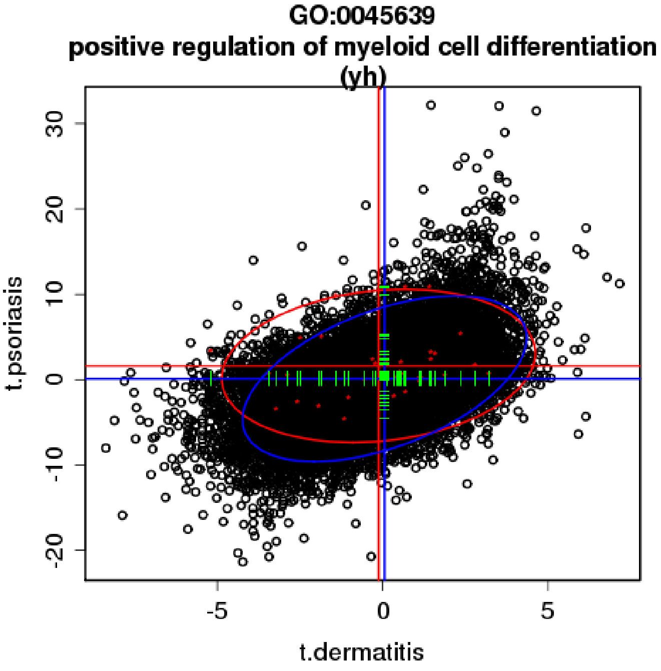
GO:0045639
positive regulation of myeloid cell differentiation
(yh)
t.psoriasis
t.dermatitis
30
20
10
0
-10
-20
-5
0
5

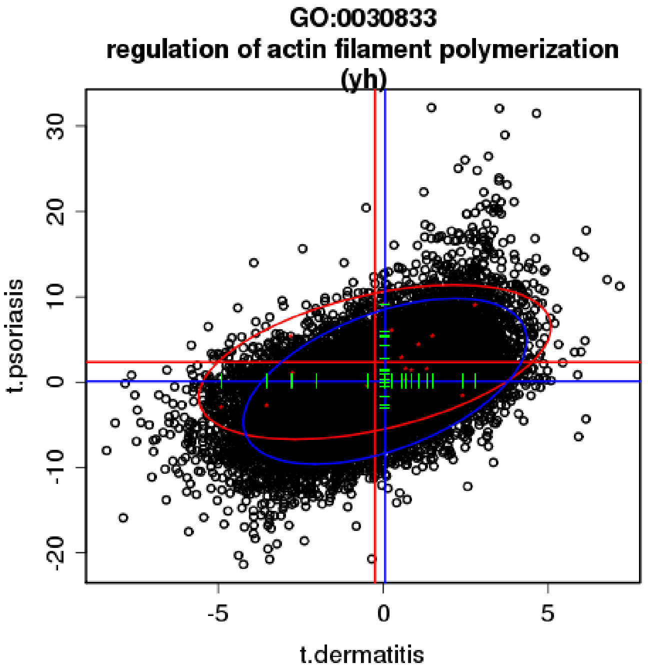
GO:0030833
regulation of actin filament polymerization
(yh)
t.psoriasis
t.dermatitis
-20
-10
0
10
20
30
-5
0
5
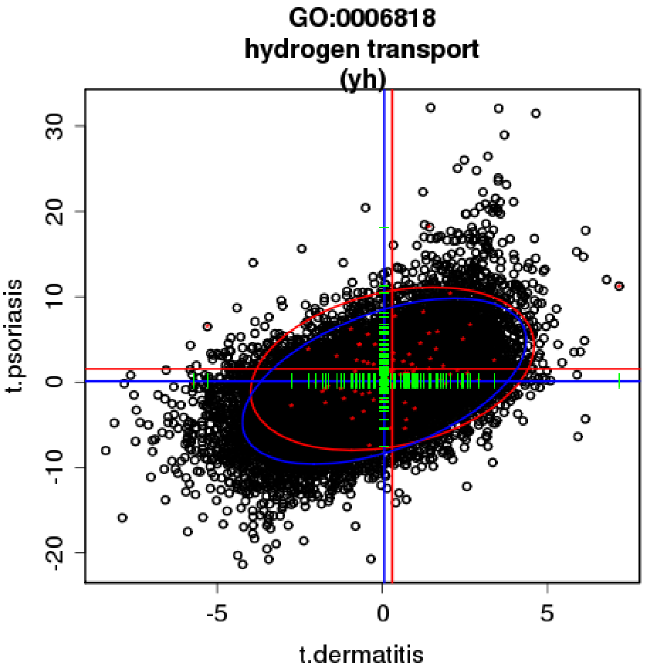
GO:0006818
hydrogen transport
(yh)
t.psoriasis
t.dermatitis
-20
-10
0
10
20
30
-5
0
5
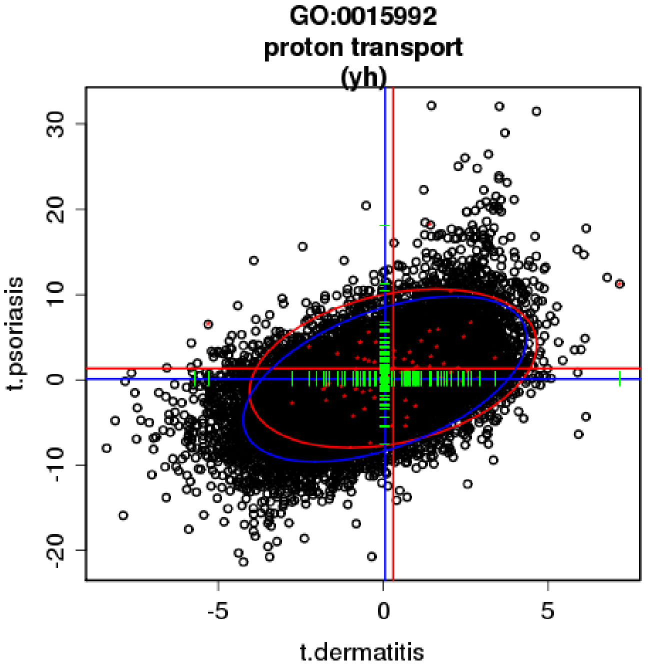
GO:0015992
proton transport
(yh)
t.psoriasis
t.dermatitis
-20
-10
0
10
20
30
-5
0
5
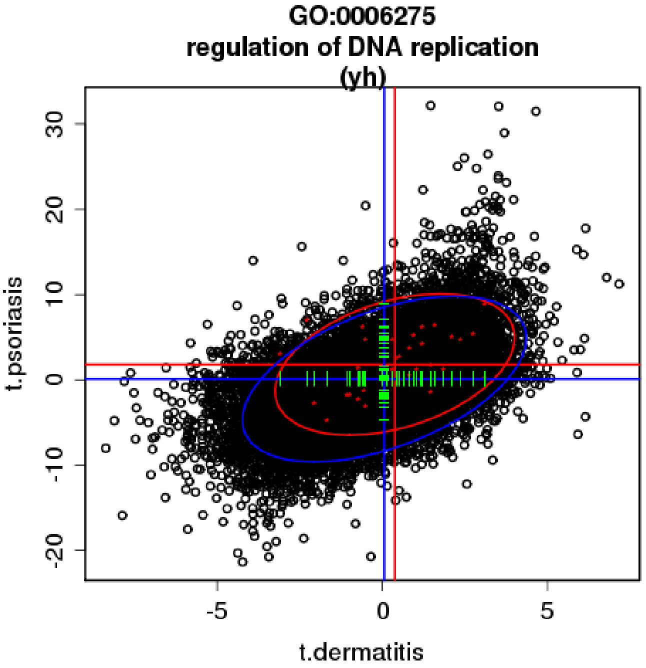
GO:0006275
regulation of DNA replication
(yh)
t.psoriasis
t.dermatitis
-20
-10
0
10
20
30
-5
0
5
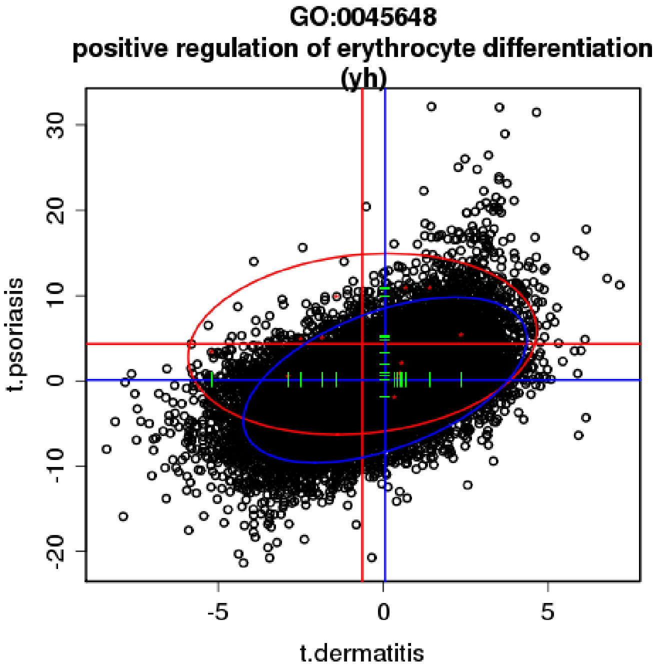
GO:0045648
positive regulation of erythrocyte differentiation
(yh)
t.psoriasis
t.dermatitis
-20
-10
0
10
20
30
-5
0
5
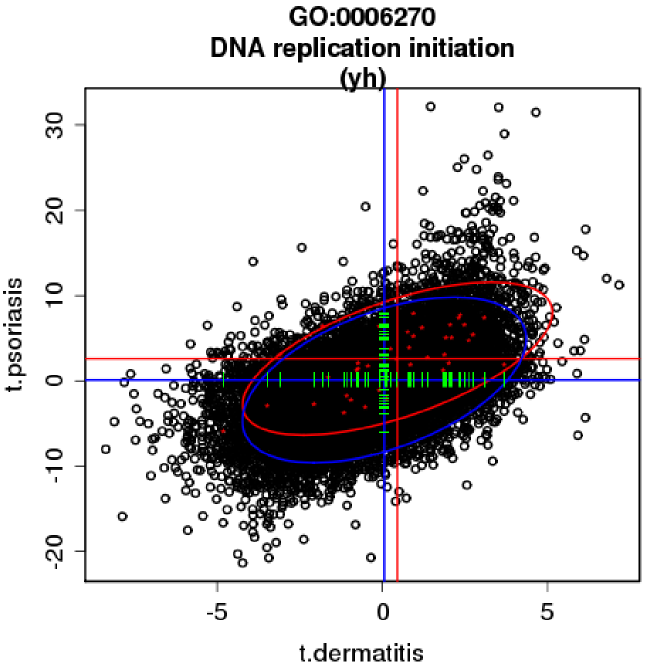
GO:0006270
DNA replication initiation
(yh)
t.psoriasis
t.dermatitis
-20
-10
0
10
20
30
-5
0
5

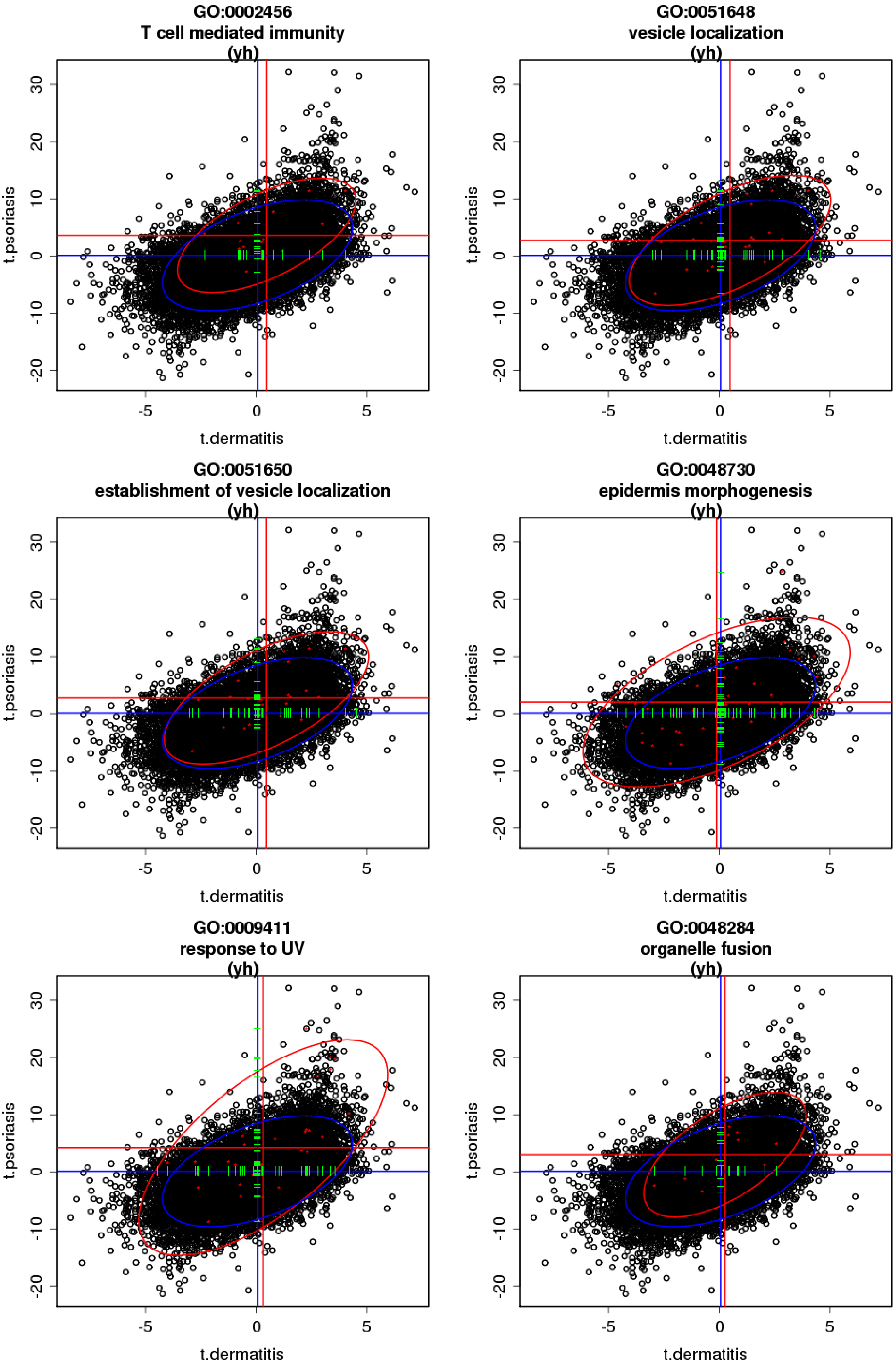
GO:0002456
T cell mediated immunity
(yh)
t.psoriasis
t.dermatitis
GO:0051648
vesicle localization
(yh)
t.psoriasis
t.dermatitis
GO:0051650
establishment of vesicle localization
(yh)
t.psoriasis
t.dermatitis
GO:0048730
epidermis morphogenesis
(yh)
t.psoriasis
t.dermatitis
GO:0009411
response to UV
(yh)
t.psoriasis
t.dermatitis
GO:0048284
organelle fusion
(yh)
t.psoriasis
t.dermatitis

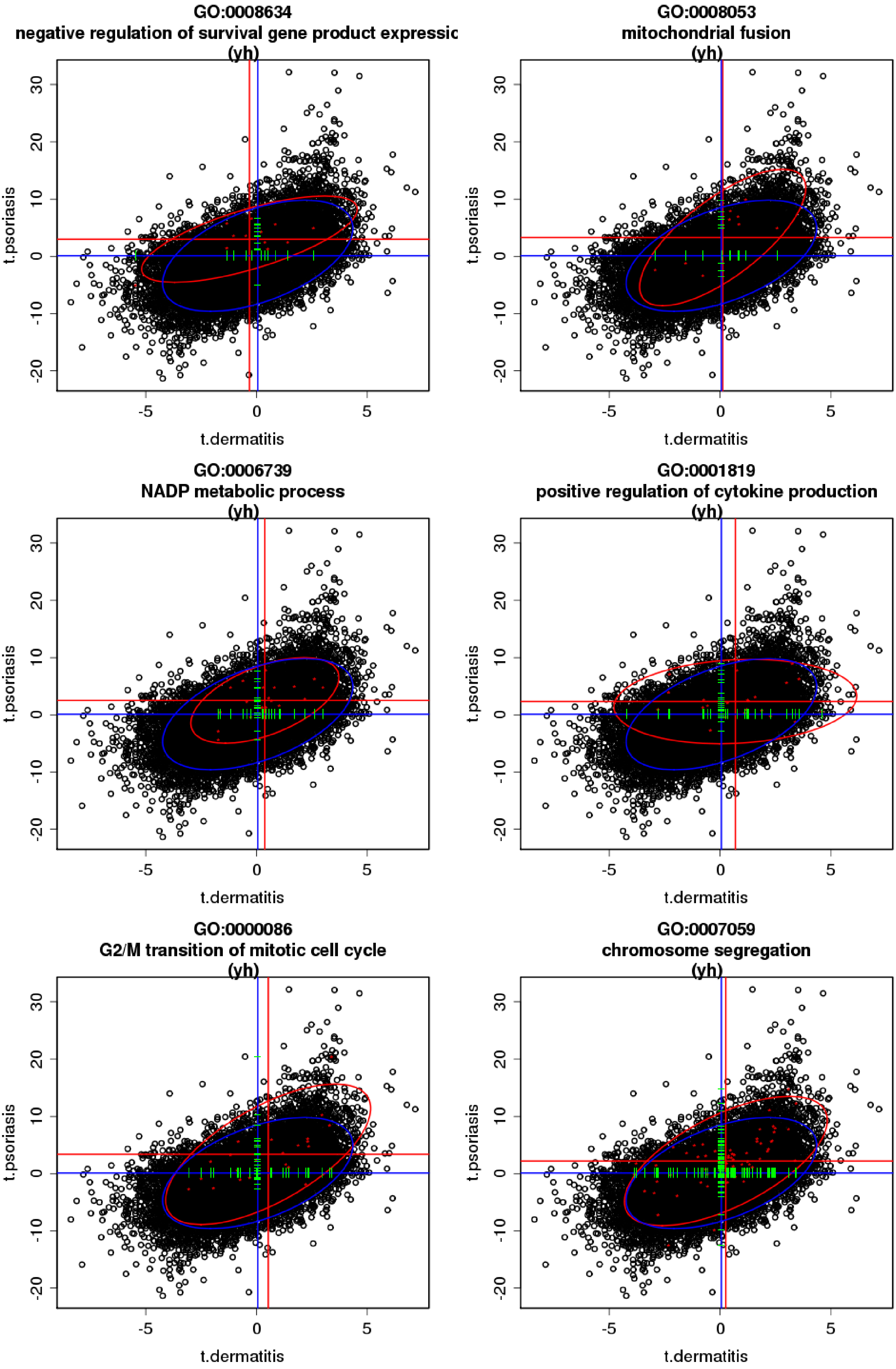

GO:0008634
negative regulation of survival gene product expressic
(yh)
t.psoriasis
t.dermatitis
GO:0008053
mitochondrial fusion
(yh)
t.psoriasis
t.dermatitis
GO:0006739
NADP metabolic process
(yh)
t.psoriasis
t.dermatitis
GO:0001819
positive regulation of cytokine production
(yh)
t.psoriasis
t.dermatitis
GO:0000086
G2/M transition of mitotic cell cycle
(yh)
t.psoriasis
t.dermatitis
GO:0007059
chromosome segregation
(yh)
t.psoriasis
t.dermatitis

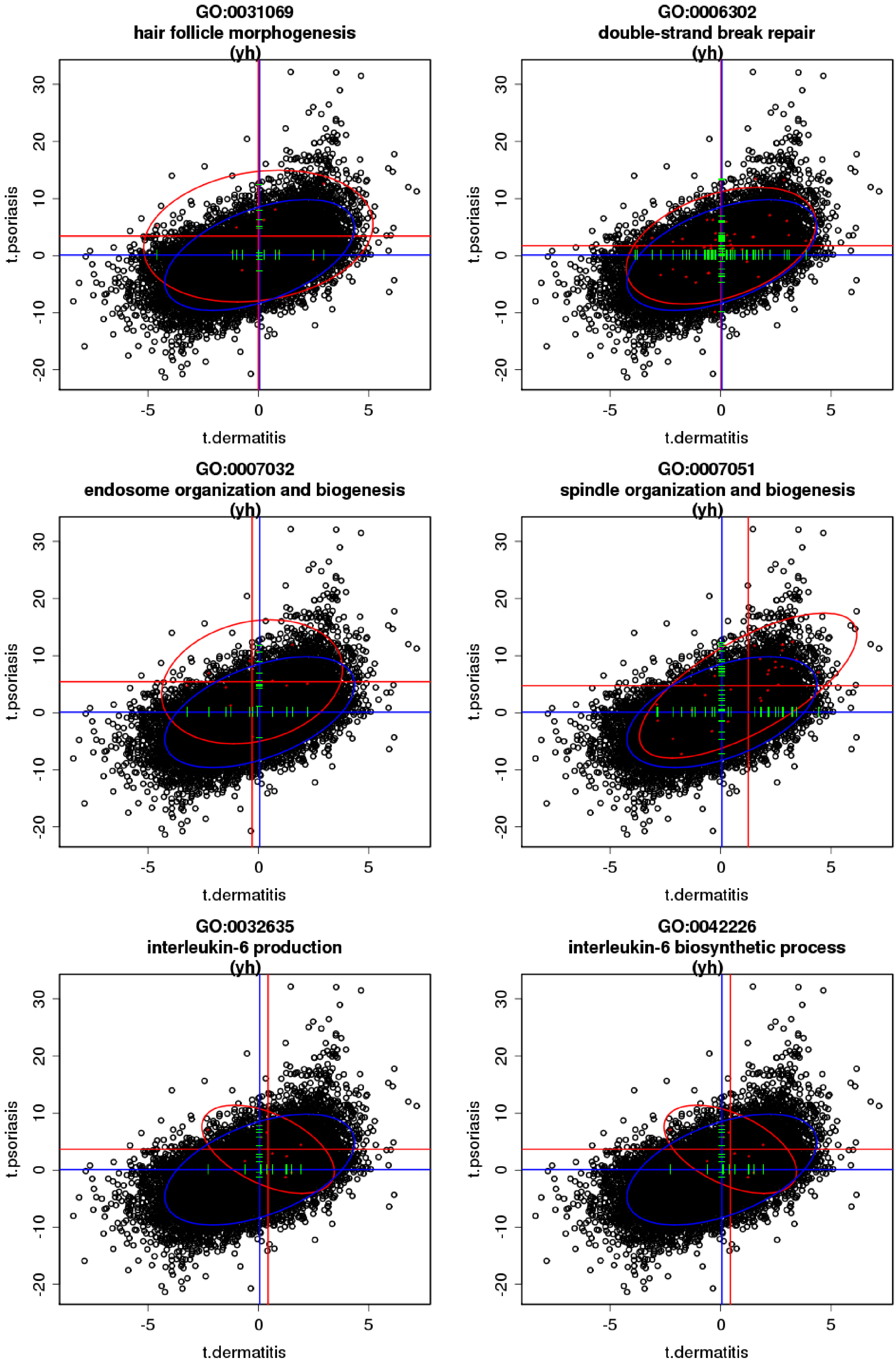
GO:0031069
hair follicle morphogenesis
(yh)
GO:0006302
double-strand break repair
(yh)
GO:0007032
endosome organization and biogenesis
(yh)
GO:0007051
spindle organization and biogenesis
(yh)
GO:0032635
interleukin-6 production
(yh)
GO:0042226
interleukin-6 biosynthetic process
(yh)
t.psoriasis
t.dermatitis
-20
-10
0
10
20
30
-5
0
5

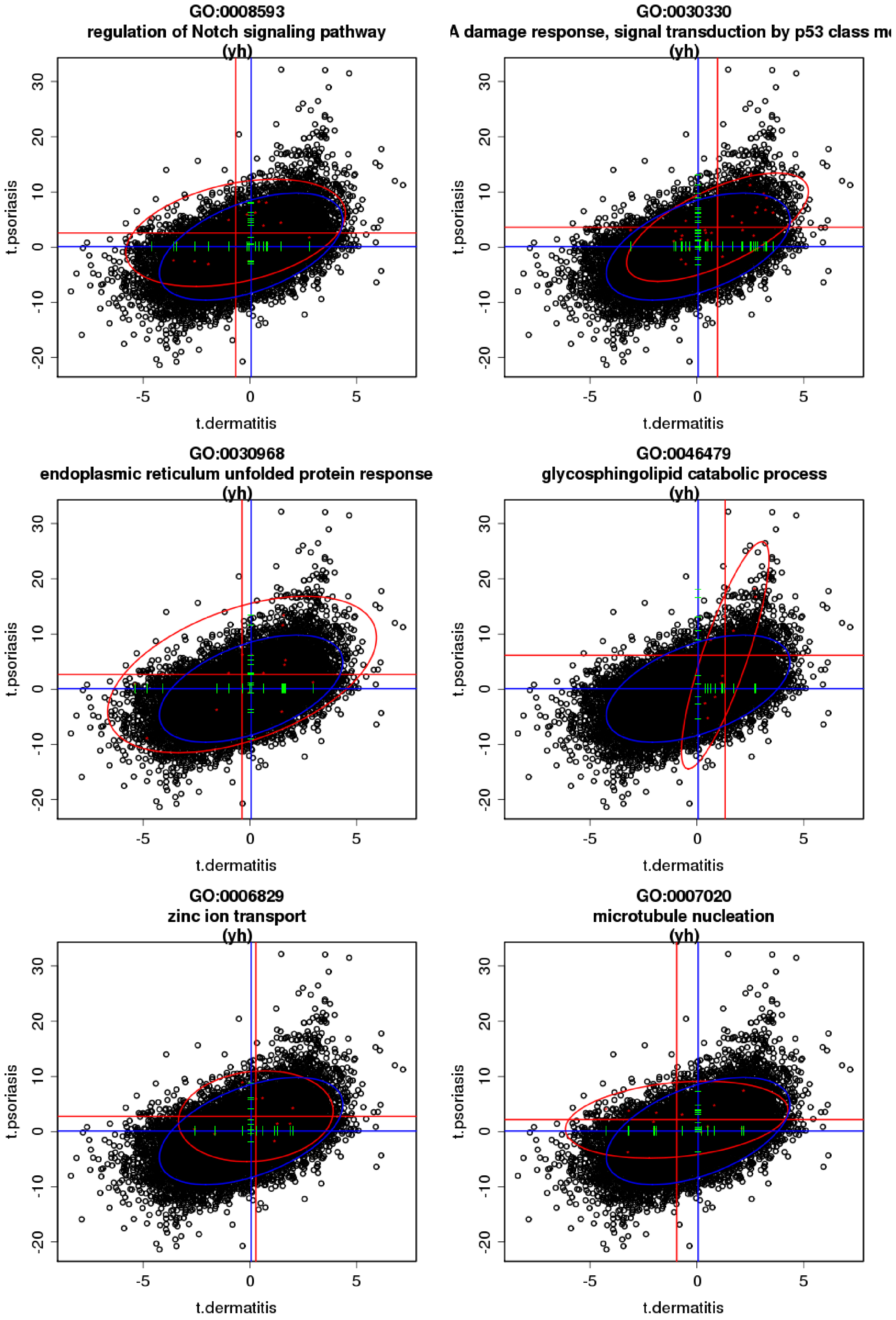
GO:0008593
regulation of Notch signaling pathway
(yh)
GO:0030330
A damage response, signal transduction by p53 class m
(yh)
GO:0030968
endoplasmic reticulum unfolded protein response
(yh)
GO:0046479
glycosphingolipid catabolic process
(yh)
GO:0006829
zinc ion transport
(yh)
GO:0007020
microtubule nucleation
(yh)
t.psoriasis
t.dermatitis
-20
-10
0
10
20
30
-5
0
5

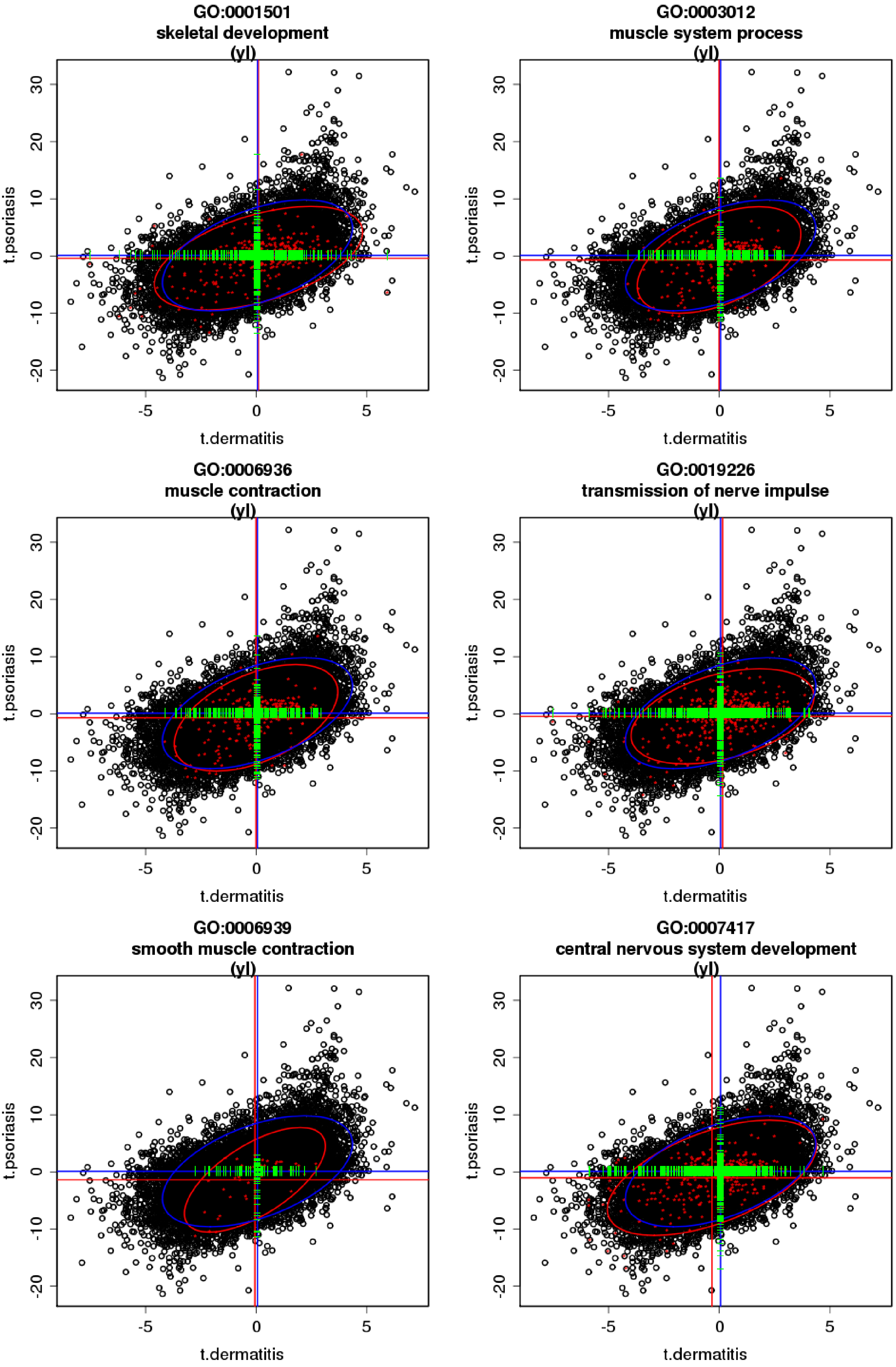
GO:0001501
skeletal development
(yl)
GO:0003012
muscle system process
(yl)
GO:0006936
muscle contraction
(yl)
GO:0019226
transmission of nerve impulse
(yl)
GO:0006939
smooth muscle contraction
(yl)
GO:0007417
central nervous system development
(yl)
t.psoriasis
t.dermatitis
-20
-10
0
10
20
30
-5
0
5

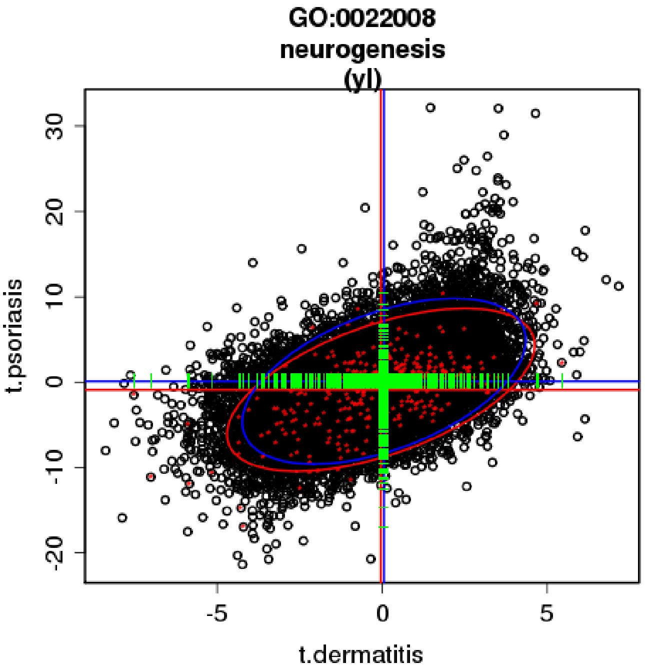
GO:0022008
neurogenesis
(yl)
t.psoriasis
t.dermatitis
30
20
10
0
-10
-20
-5
0
5

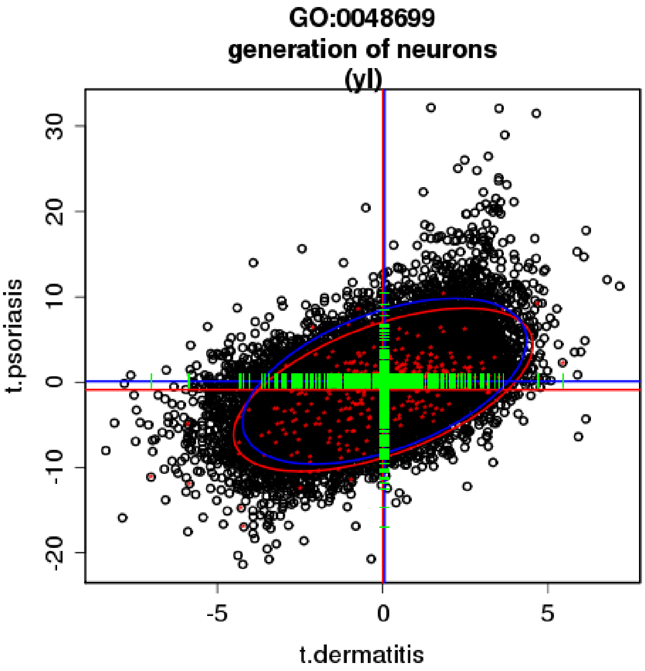
GO:0048699
generation of neurons
(yl)
t.psoriasis
t.dermatitis
30
20
10
0
-10
-20
-5
0
5

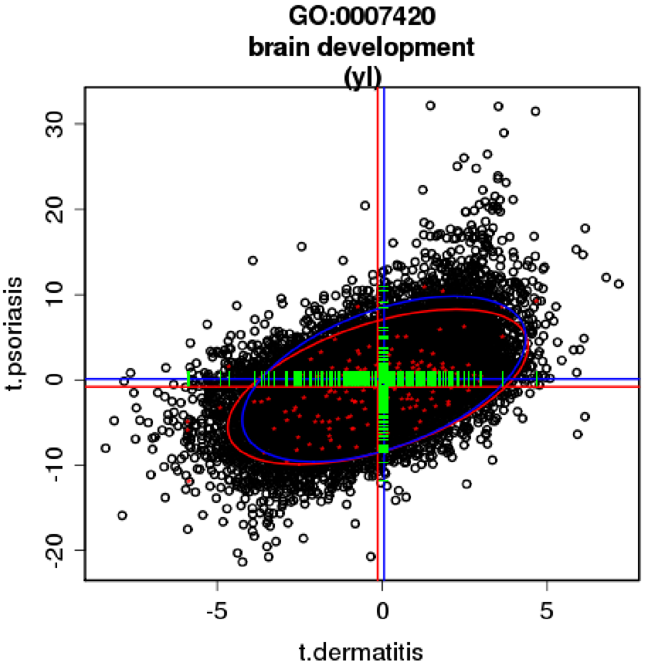
GO:0007420
brain development
(yl)
t.psoriasis
t.dermatitis
30
20
10
0
-10
-20
-5
0
5

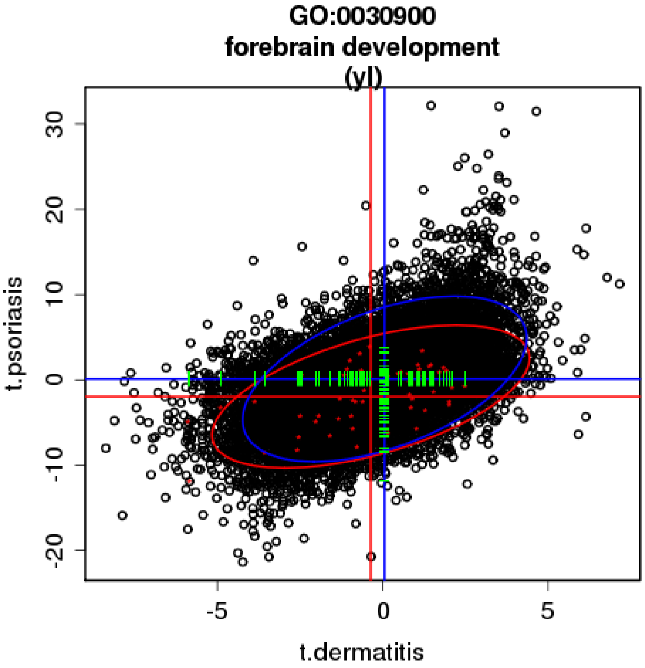
GO:0030900
forebrain development
(yl)
t.psoriasis
t.dermatitis
30
20
10
0
-10
-20
-5
0
5

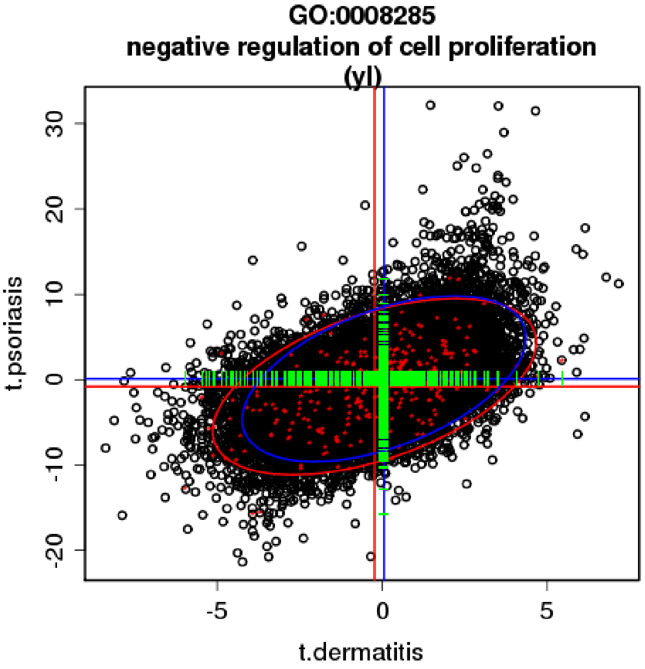
GO:0008285
negative regulation of cell proliferation
(yl)
t.psoriasis
t.dermatitis
30
20
10
0
-10
-20
-5
0
5

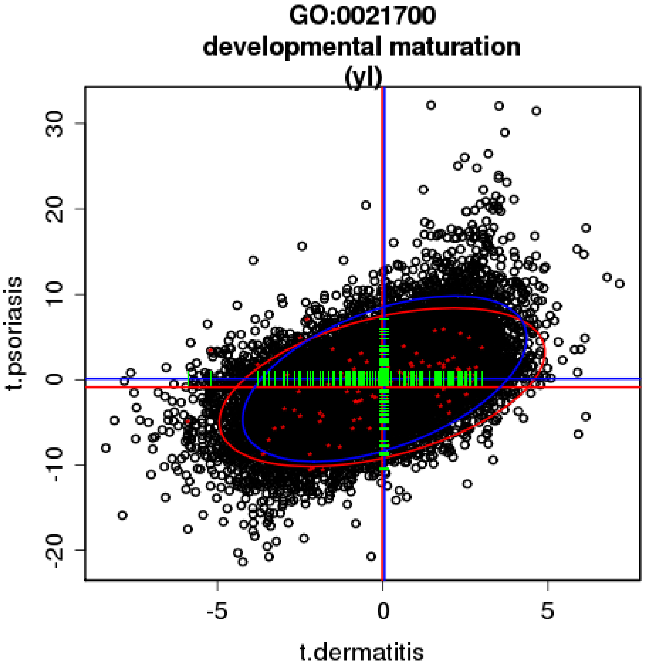
GO:0021700
developmental maturation
(yl)
t.psoriasis
t.dermatitis
30
20
10
0
-10
-20
-5
0
5

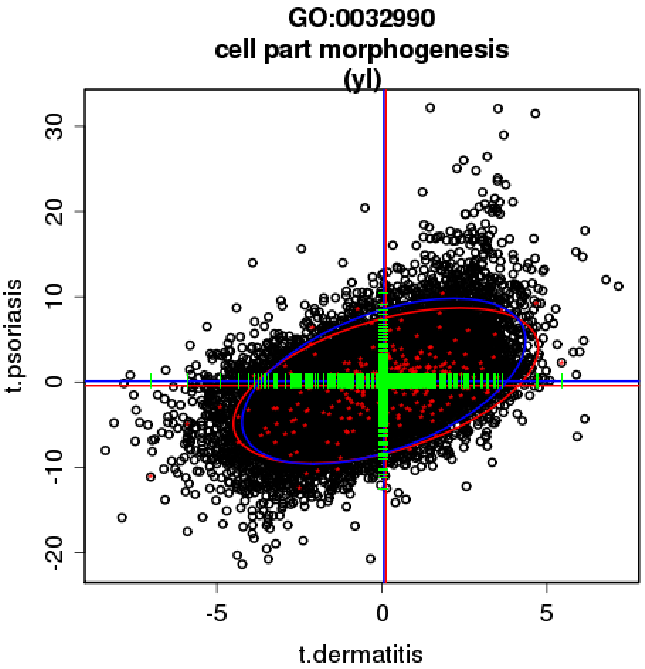
GO:0032990
cell part morphogenesis
(yl)
t.psoriasis
30
20
10
0
-10
-20
-5
0
5
t.dermatitis

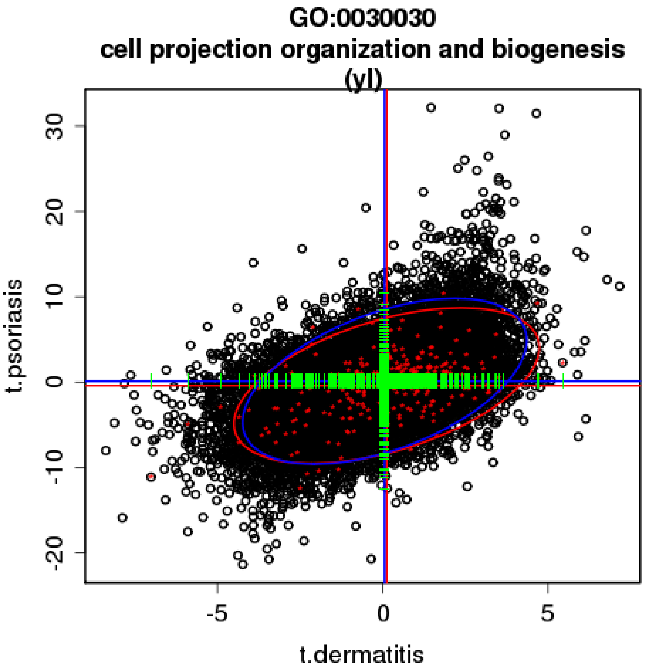
GO:0030030
cell projection organization and biogenesis
(yl)
t.psoriasis
30
20
10
0
-10
-20
-5
0
5
t.dermatitis

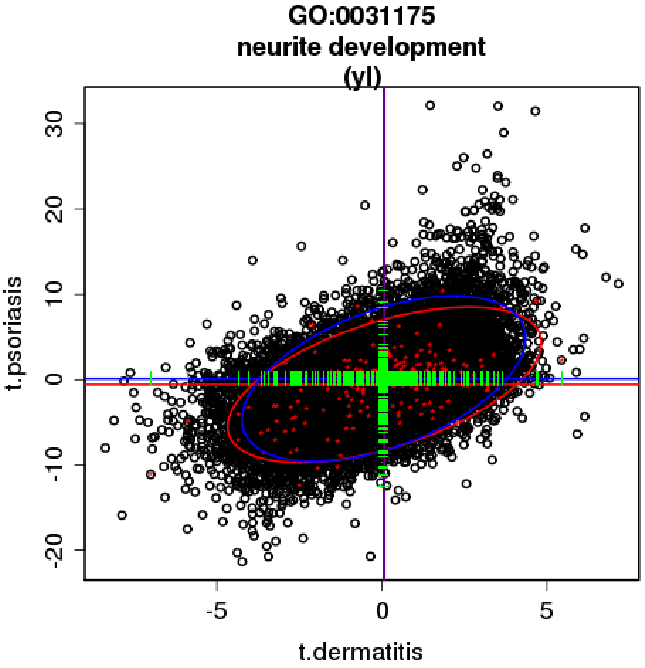
GO:0031175
neurite development
(yl)
t.psoriasis
30
20
10
0
-10
-20
-5
0
5
t.dermatitis

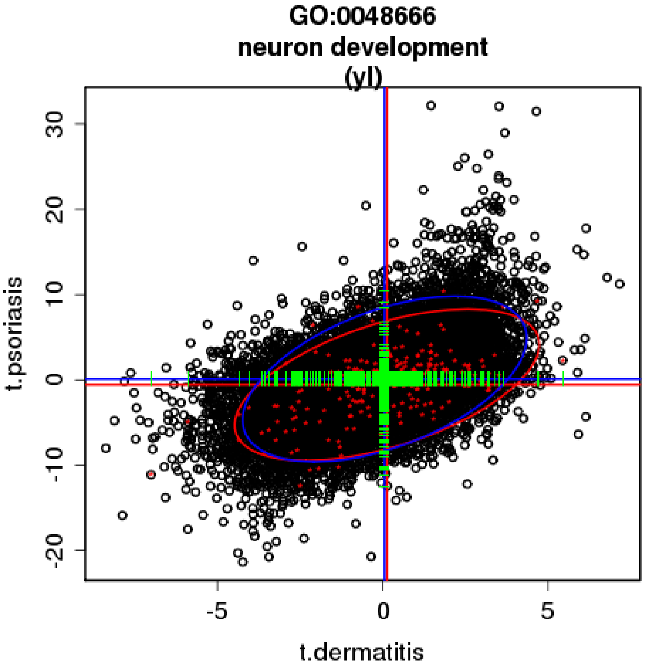
GO:0048666
neuron development
(yl)
t.psoriasis
30
20
10
0
-10
-20
-5
0
5
t.dermatitis

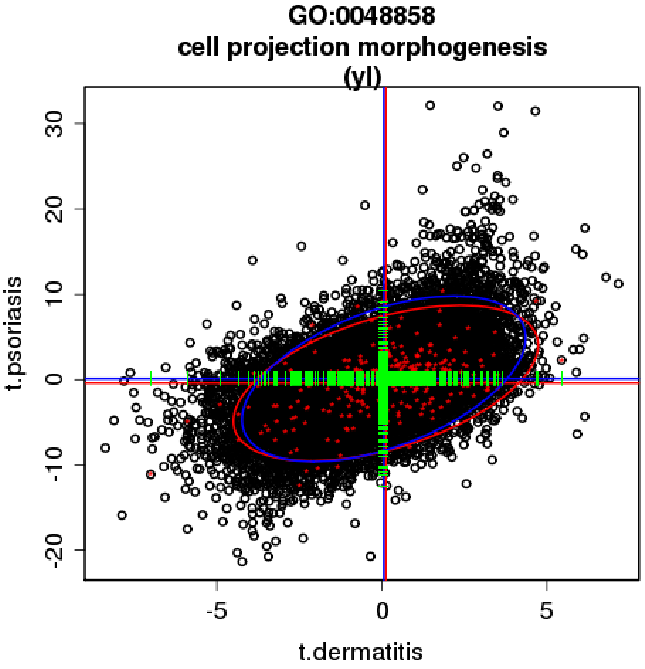
GO:0048858
cell projection morphogenesis
(yl)
t.psoriasis
30
20
10
0
-10
-20
-5
0
5
t.dermatitis

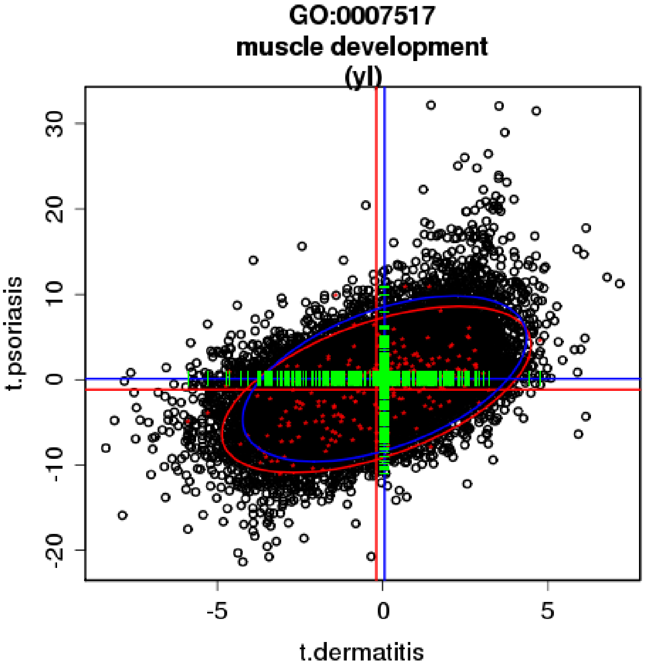
GO:0007517
muscle development
(yl)
t.psoriasis
30
20
10
0
-10
-20
-5
0
5
t.dermatitis

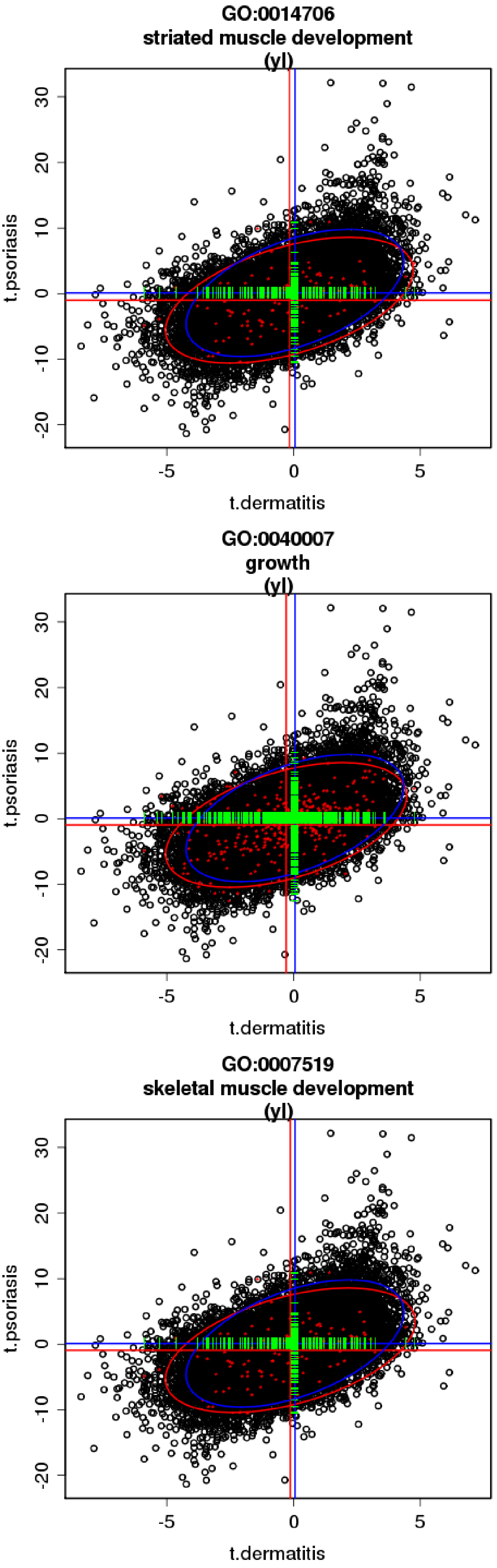

GO:0014706
striated muscle development
(yl)
GO:0008361
regulation of cell size
(yl)
GO:0040007
growth
(yl)
GO:0016049
cell growth
(yl)
GO:0007519
skeletal muscle development
(yl)
GO:0000271
polysaccharide biosynthetic process
(yl)
t.psoriasis
t.dermatitis

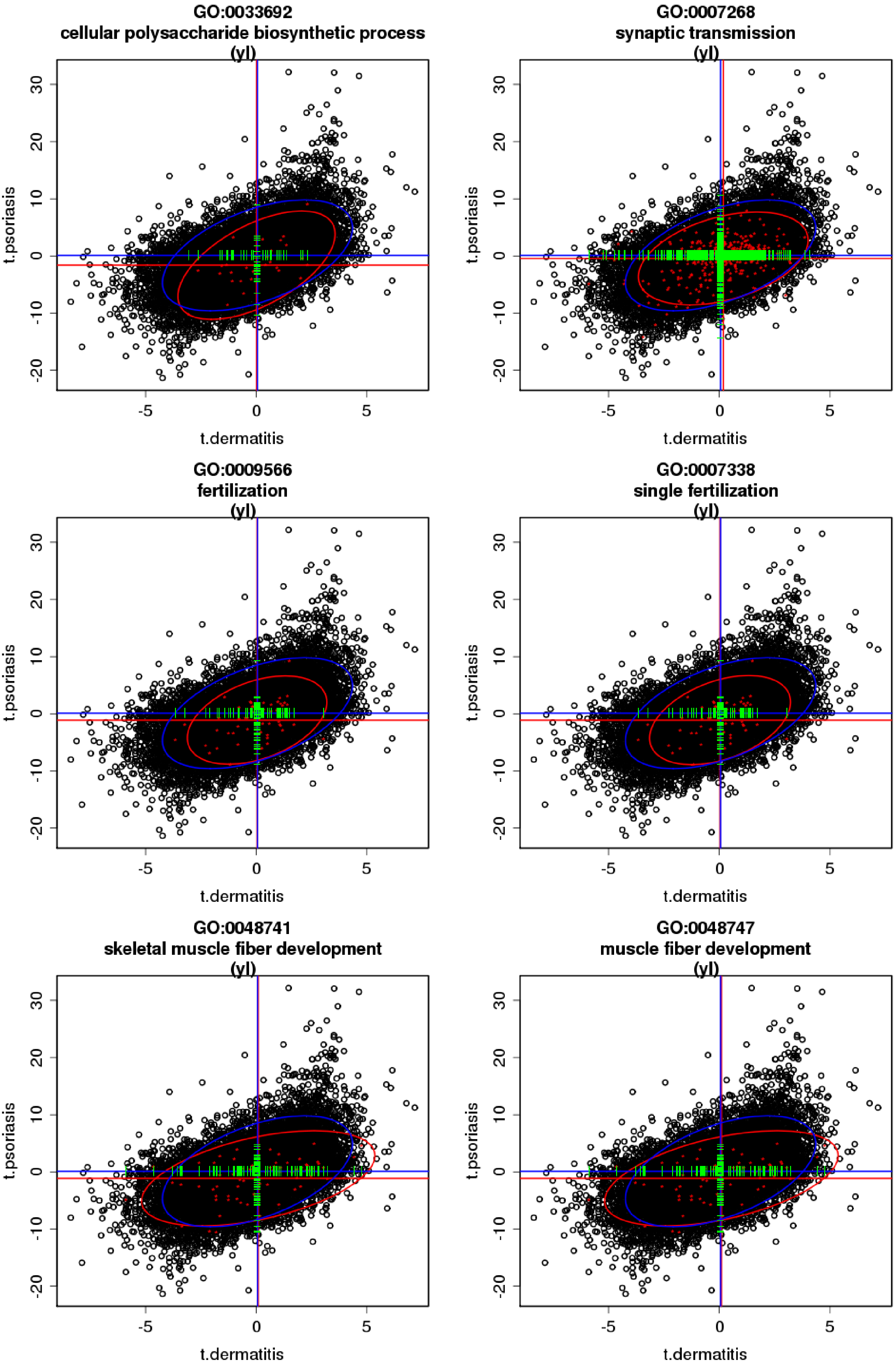
GO:0033692
cellular polysaccharide biosynthetic process
(yl)
t.psoriasis
t.dermatitis
GO:0007268
synaptic transmission
(yl)
t.psoriasis
t.dermatitis
GO:0009566
fertilization
(yl)
t.psoriasis
t.dermatitis
GO:0007338
single fertilization
(yl)
t.psoriasis
t.dermatitis
GO:0048741
skeletal muscle fiber development
(yl)
t.psoriasis
t.dermatitis
GO:0048747
muscle fiber development
(yl)
t.psoriasis
t.dermatitis

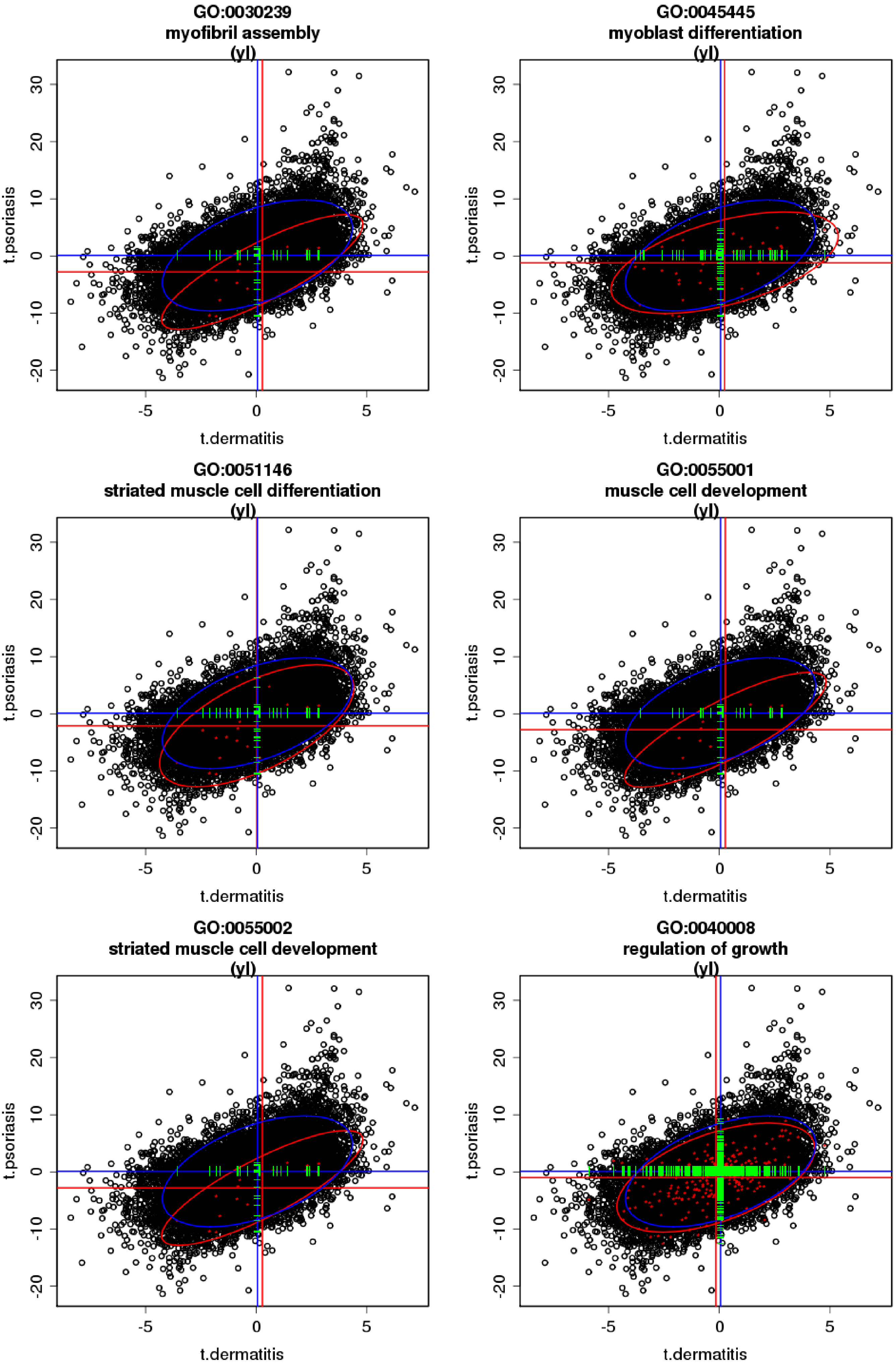
GO:0030239
myofibril assembly
(yl)
GO:0045445
myoblast differentiation
(yl)
GO:0051146
striated muscle cell differentiation
(yl)
GO:0055001
muscle cell development
(yl)
GO:0055002
striated muscle cell development
(yl)
GO:0040008
regulation of growth
(yl)
t.psoriasis
t.dermatitis
-20
-10
0
10
20
30
-5
0
5

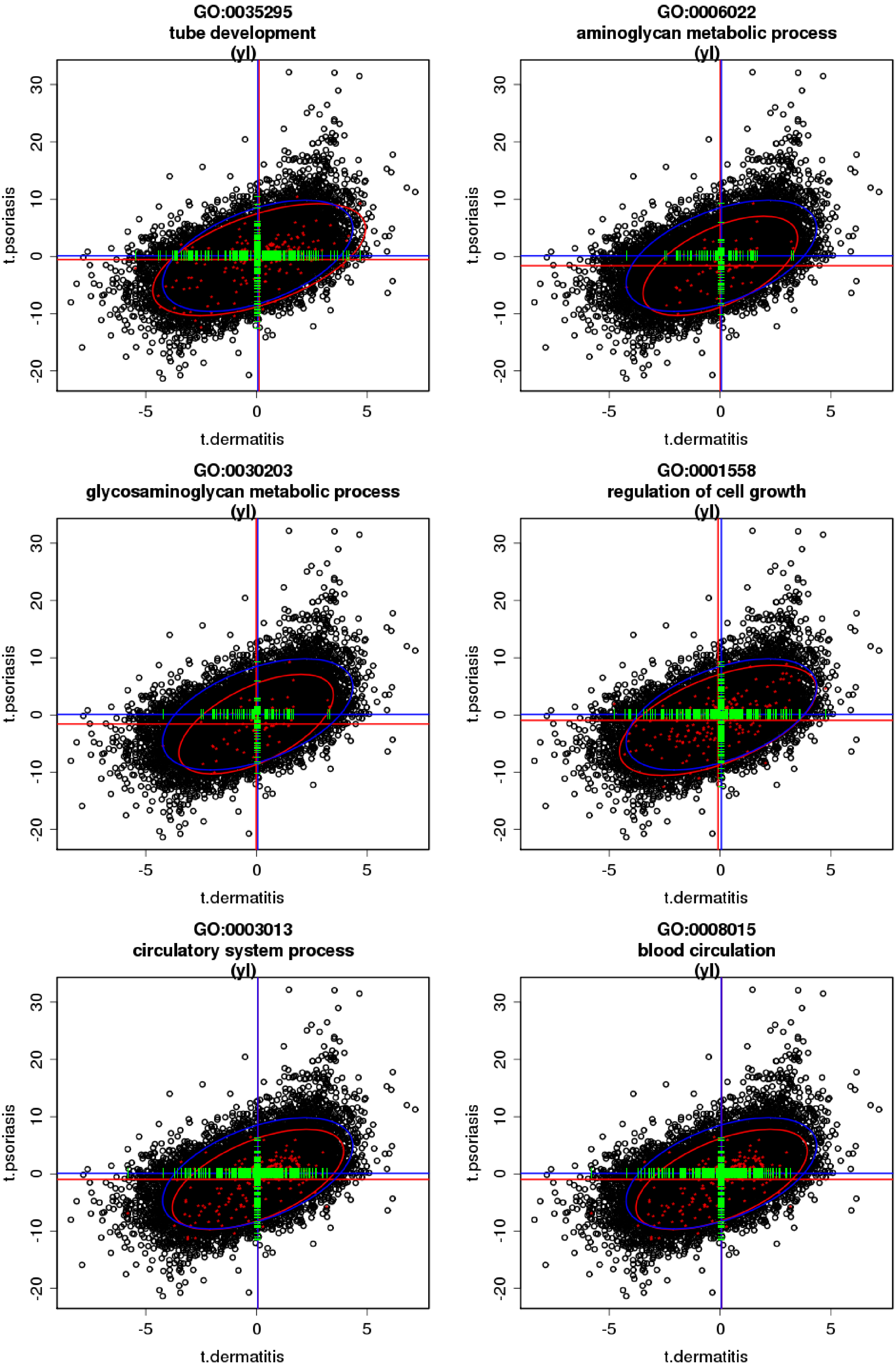

GO:0035295
tube development
(yl)
t.psoriasis
t.dermatitis
GO:0006022
aminoglycan metabolic process
(yl)
t.psoriasis
t.dermatitis
GO:0030203
glycosaminoglycan metabolic process
(yl)
t.psoriasis
t.dermatitis
GO:0001558
regulation of cell growth
(yl)
t.psoriasis
t.dermatitis
GO:0003013
circulatory system process
(yl)
t.psoriasis
t.dermatitis
GO:0008015
blood circulation
(yl)
t.psoriasis
t.dermatitis

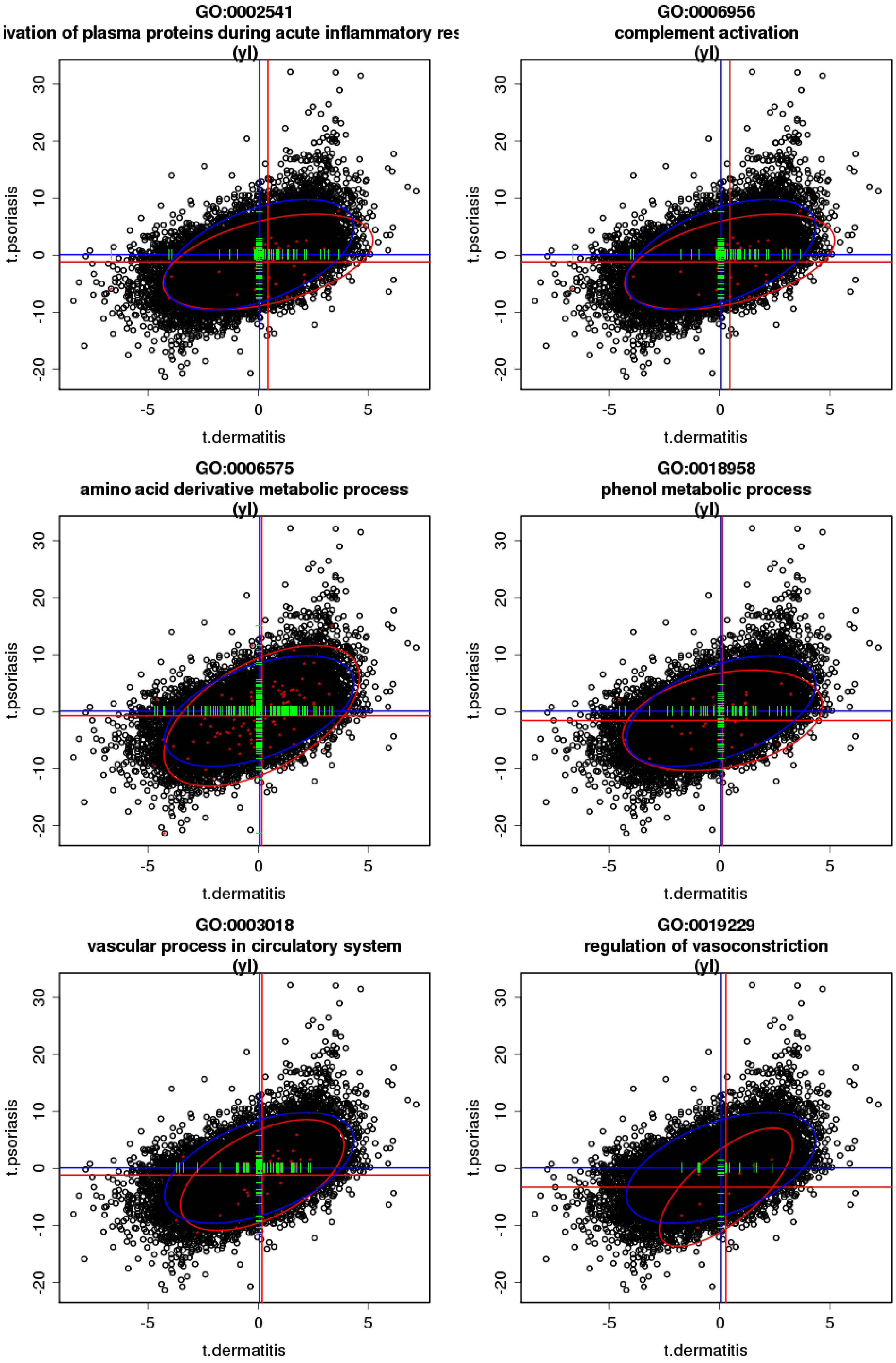
GO:0002541
ivation of plasma proteins during acute inflammatory res
(yl)
GO:0006956
complement activation
(yl)
GO:0006575
amino acid derivative metabolic process
(yl)
GO:0018958
phenol metabolic process
(yl)
GO:0003018
vascular process in circulatory system
(yl)
GO:0019229
regulation of vasoconstriction
(yl)
t.psoriasis
t.dermatitis
30
20
10
0
-10
-20
-5
0
5

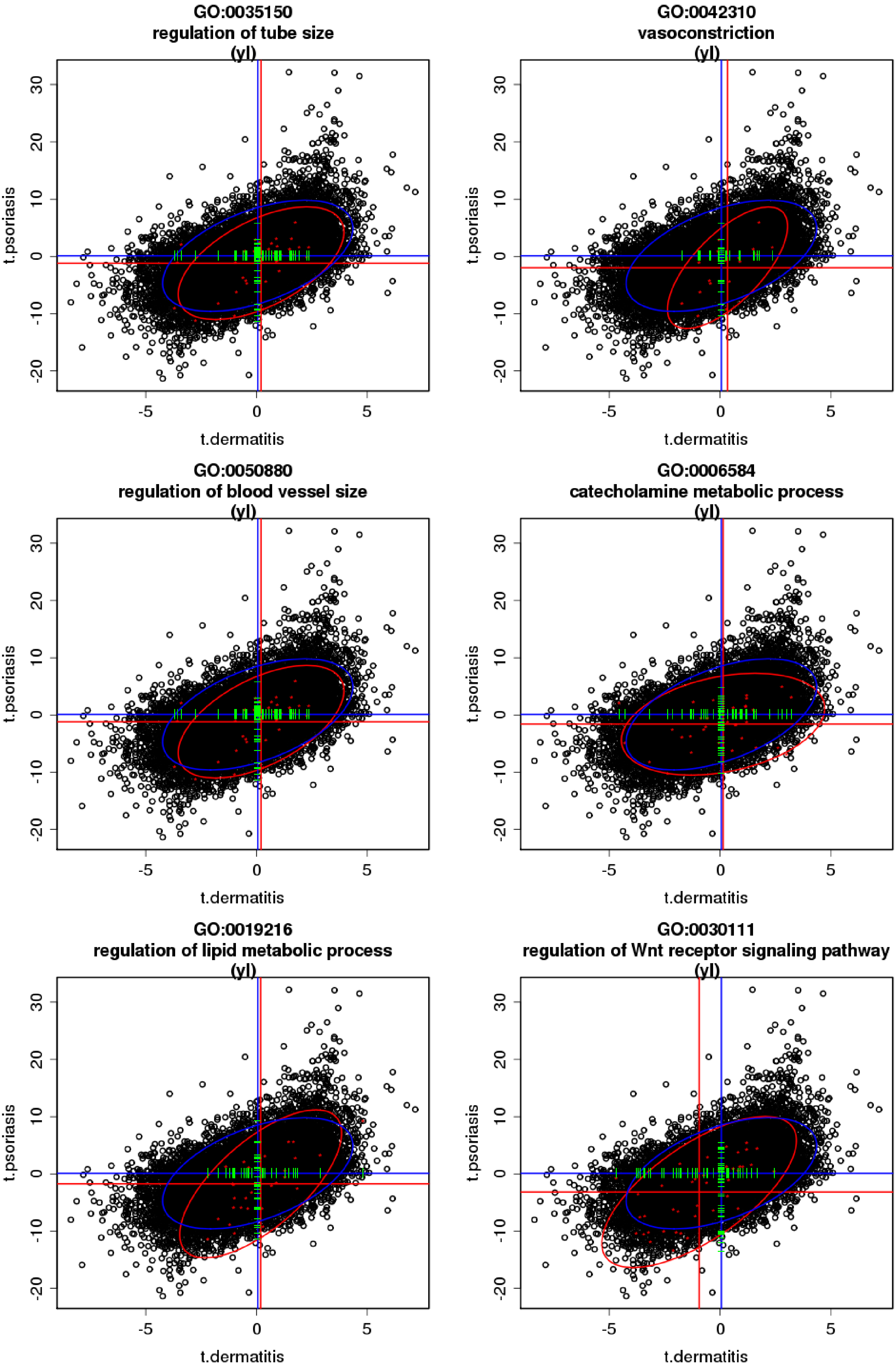
GO:0035150
regulation of tube size
(yl)
GO:0042310
vasoconstriction
(yl)
GO:0050880
regulation of blood vessel size
(yl)
GO:0006584
catecholamine metabolic process
(yl)
GO:0019216
regulation of lipid metabolic process
(yl)
GO:0030111
regulation of Wnt receptor signaling pathway
(yl)
t.psoriasis
t.dermatitis
-20
-10
0
10
20
30
-5
0
5

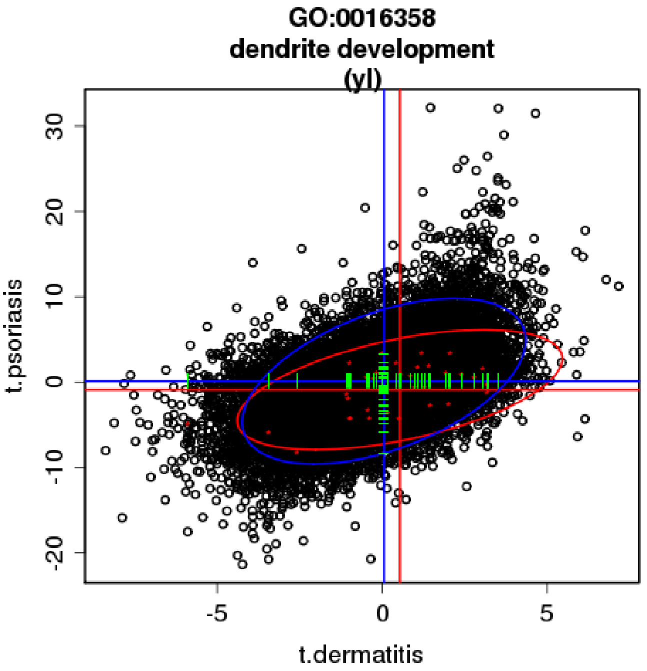
GO:0016358
dendrite development
(yl)
t.psoriasis
t.dermatitis
-5
0
5
-20
-10
0
10
20
30

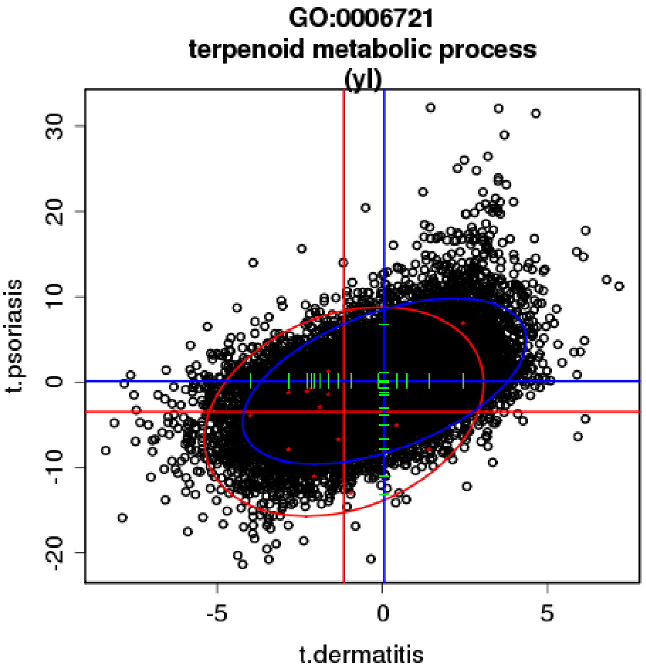
GO:0006721
terpenoid metabolic process
(yl)
t.psoriasis
t.dermatitis
-5
0
5
-20
-10
0
10
20
30

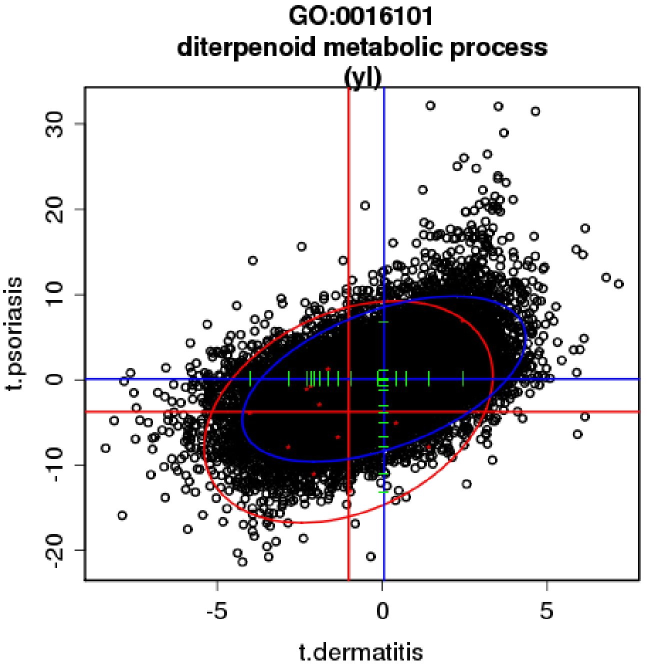
GO:0016101
diterpenoid metabolic process
(yl)
t.psoriasis
t.dermatitis
-5
0
5
-20
-10
0
10
20
30

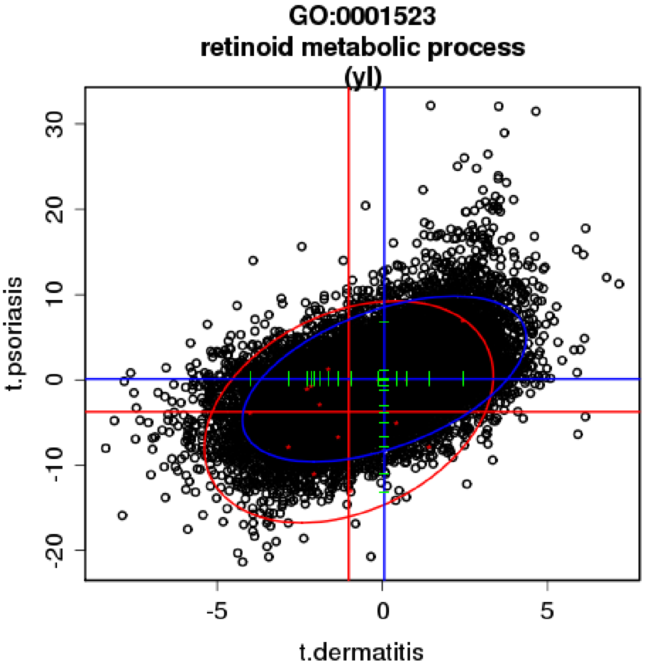
GO:0001523
retinoid metabolic process
(yl)
t.psoriasis
t.dermatitis
-5
0
5
-20
-10
0
10
20
30

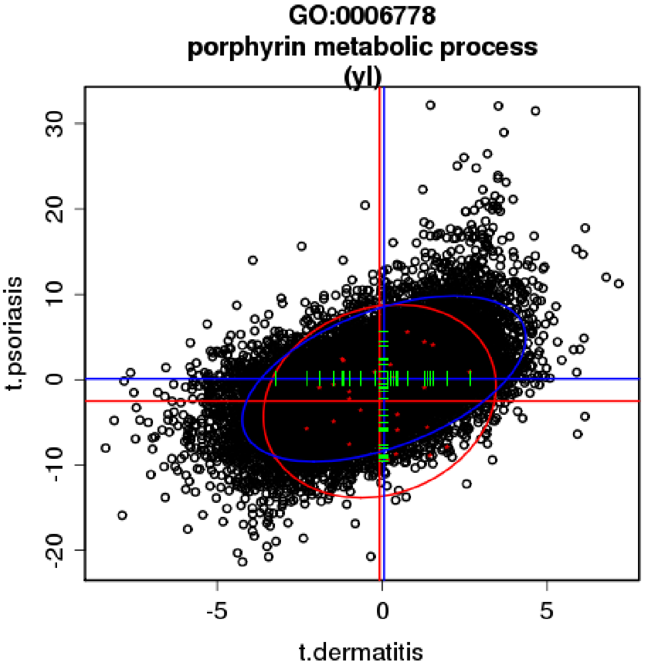
GO:0006778
porphyrin metabolic process
(yl)
t.psoriasis
t.dermatitis
-5
0
5
-20
-10
0
10
20
30

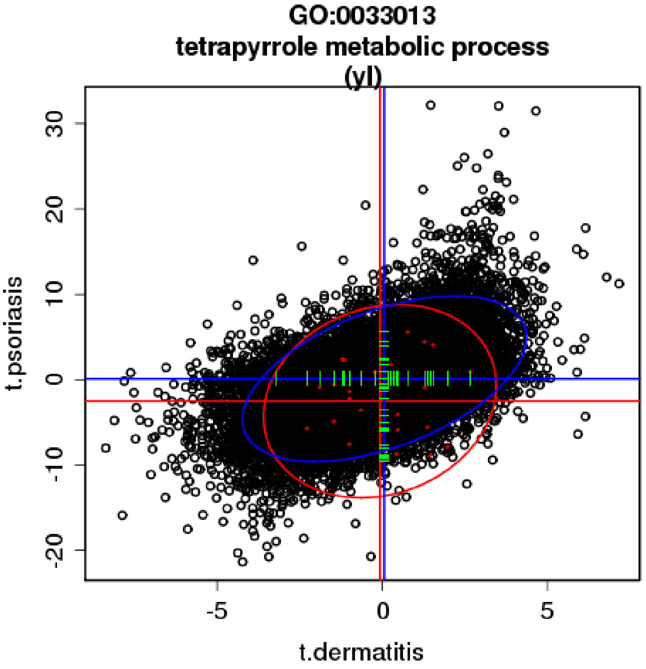
GO:0033013
tetrapyrrole metabolic process
(yl)
t.psoriasis
t.dermatitis
-5
0
5
-20
-10
0
10
20
30

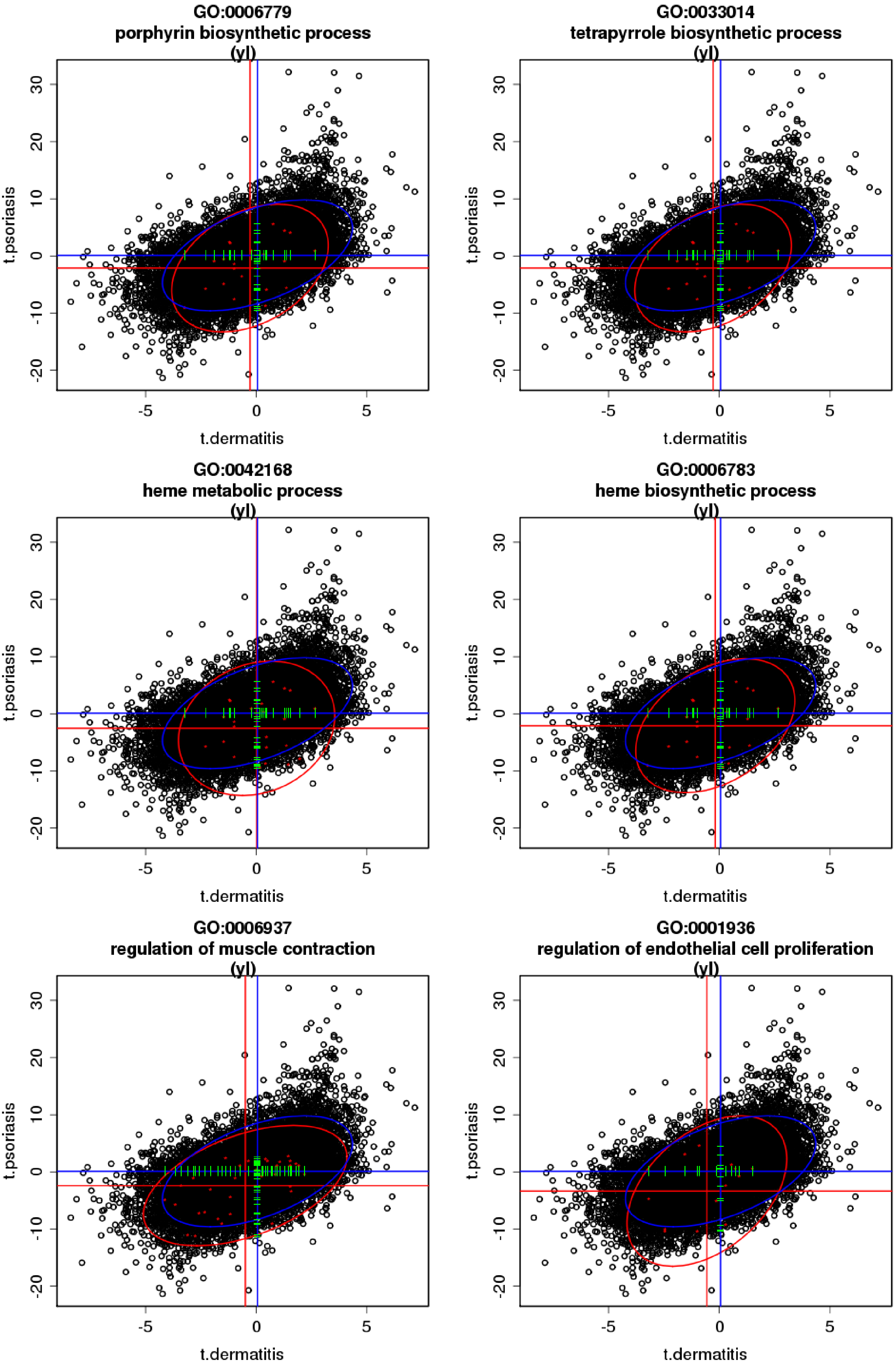
GO:0006779
porphyrin biosynthetic process
(yl)
GO:0033014
tetrapyrrole biosynthetic process
(yl)
GO:0042168
heme metabolic process
(yl)
GO:0006783
heme biosynthetic process
(yl)
GO:0006937
regulation of muscle contraction
(yl)
GO:0001936
regulation of endothelial cell proliferation
(yl)
t.psoriasis
t.dermatitis
-20
-10
0
10
20
30
-5
0
5

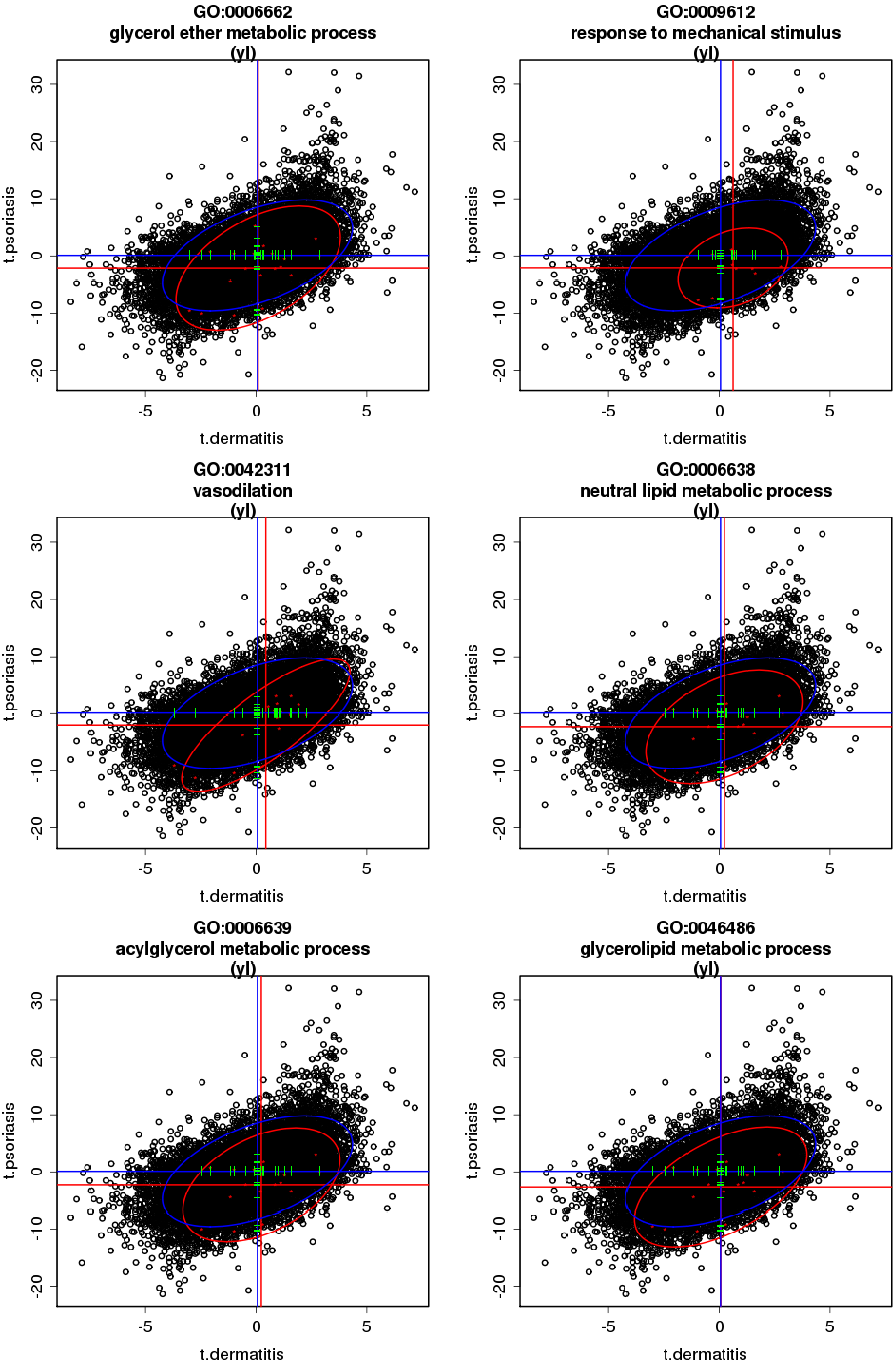
GO:0006662
glycerol ether metabolic process
(yl)
t.psoriasis
t.dermatitis
GO:0009612
response to mechanical stimulus
(yl)
t.psoriasis
t.dermatitis
GO:0042311
vasodilation
(yl)
t.psoriasis
t.dermatitis
GO:0006638
neutral lipid metabolic process
(yl)
t.psoriasis
t.dermatitis
GO:0006639
acylglycerol metabolic process
(yl)
t.psoriasis
t.dermatitis
GO:0046486
glycerolipid metabolic process
(yl)
t.psoriasis
t.dermatitis

GO:0030178
negative regulation of Wnt receptor signaling pathway
(yl)

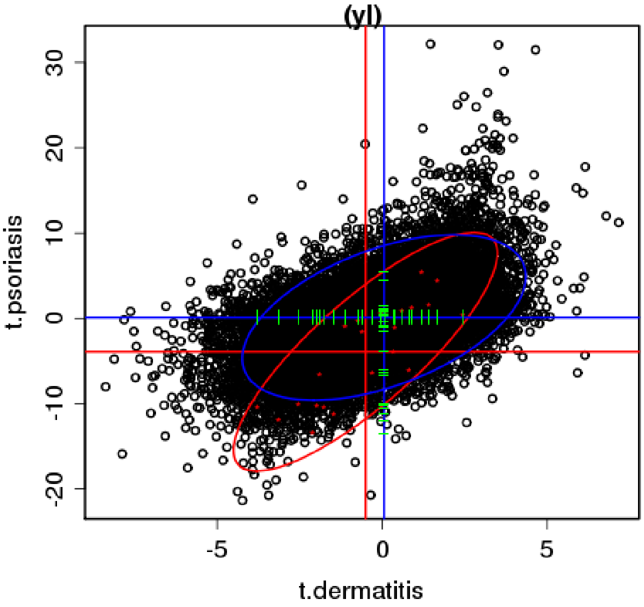

GO:0046928
regulation of neurotransmitter secretion
(yl)

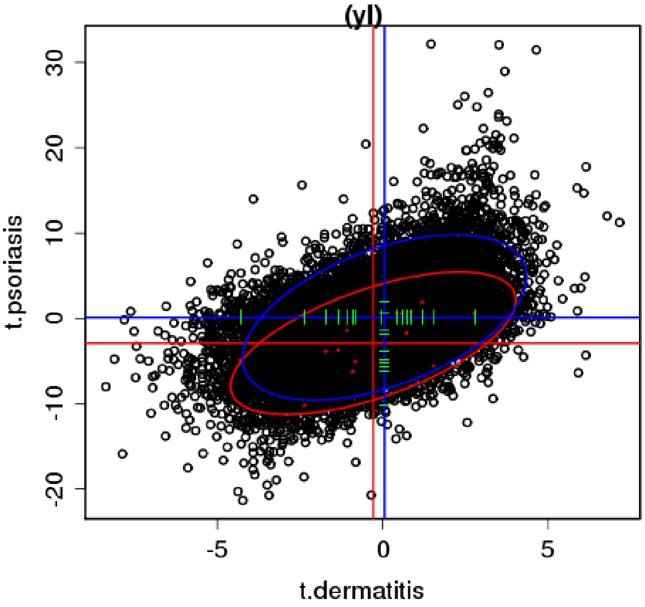

GO:0048168
regulation of neuronal synaptic plasticity
(yl)

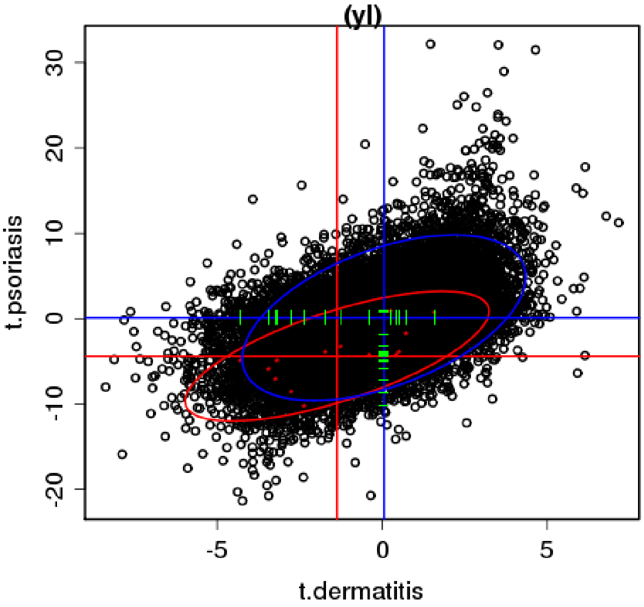

GO:0007398
ectoderm development
(no_rel)

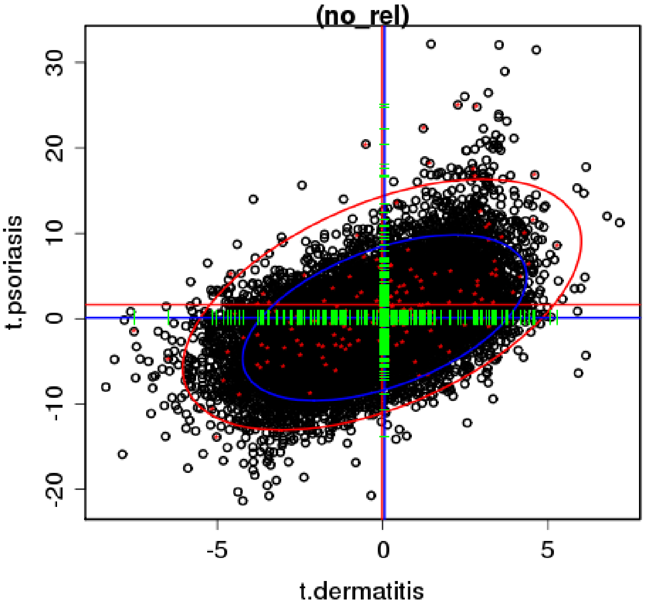

GO:0000278
mitotic cell cycle
(no_rel)

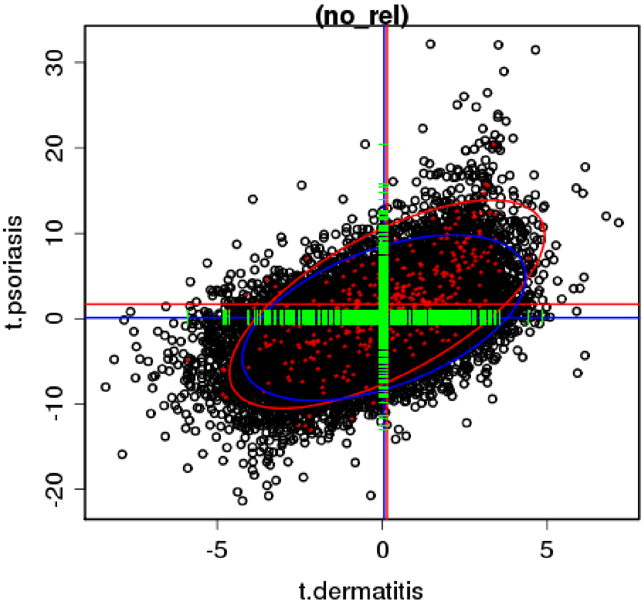

Supplement: File S3 — A) GO Biological Process terms, and B) KEGG pathways, significant for differential gene expression in dermatitis and psoriasis case-control studies and their interaction, along with the corresponding graphical representations. In the plots blue lines intersect in the mean of the distribution of all the values and red lines intersect in the mean of the distribution of values of the genes corresponding to the term analysed. Blue ellipse delimits the confidence interval for all the values and red ellipse delimits the confidence interval for the term analysed. The red ellipse marks the trend of the relationship between both variables. (30.60 MB ZIP) [file pone.0010348.s009.zip › Montaner_File_S3A-GO_dermatitis_and_psoriasis_bp.pdf]
