# Supplementary material for: Multidimensional Gene Set Analysis of Genomic Data
Source: PLoS One. 2010 Apr 27;5(4):e10348. doi: 10.1371/journal.pone.0010348 (PMC2860497; doi:10.1371/journal.pone.0010348)

Combined functional profiling of dermatitis and psoriasis datasets using KEGG pathways

31 significant modules

LOR indicates Log Odds Ratio of the coefficient or the interaction

p indicates FDR adjusted p-value

	LOR t d	LOR t p	LOR Inter	p t d	p t p	p Inter	pattern	name
00920	-0.35	-1.37	-1.02	0.74	0.00	0.03	q4i	Sulfur metabolism
04080	0.11	-0.03	0.17	0.70	0.91	0.04	b13	Neuroactive ligand-receptor interaction
00120	-0.08	-0.04	0.31	0.91	0.93	0.01	b13	Bile acid biosynthesis
00310	0.24	-0.15	0.34	0.76	0.82	0.00	b13	Lysine degradation
00632	-0.30	-0.57	-0.97	0.78	0.53	0.03	b24	Benzoate degradation via CoA ligation
04060	0.70	0.03	-0.09	0.00	0.91	0.85	xh	Cytokine-cytokine receptor interaction
04640	0.56	-0.17	0.07	0.02	0.62	0.93	xh	Hematopoietic cell lineage
00010	0.96	-0.53	0.24	0.00	0.08	0.06	xh	Glycolysis / Gluconeogenesis
04650	0.72	0.35	-0.22	0.00	0.12	0.31	xh	Natural killer cell mediated cytotoxicity
04612	0.77	0.42	-0.31	0.00	0.10	0.19	xh	Antigen processing and presentation
04210	0.62	0.06	-0.04	0.01	0.91	0.96	xh	Apoptosis
05050	1.36	-0.76	0.28	0.01	0.14	0.18	xh	Dentatorubropallidoluysian atrophy (DRPLA)
04940	1.01	0.10	-0.22	0.00	0.90	0.72	xh	Type I diabetes mellitus
05040	1.01	-0.19	-0.25	0.01	0.90	0.93	xh	Huntington's disease
04662	0.68	-0.34	0.15	0.04	0.34	0.70	xh	B cell receptor signaling pathway
05131	0.83	-0.13	-0.05	0.02	0.90	0.96	xh	Pathogenic Escherichia coli infection - EPEC
05130	0.83	-0.13	-0.05	0.02	0.90	0.96	xh	Pathogenic Escherichia coli infection - EHEC
04514	0.57	-0.30	0.10	0.00	0.18	0.72	xh	Cell adhesion molecules (CAMs)
00030	1.15	0.72	-0.49	0.04	0.31	0.57	xh	Pentose phosphate pathway
04930	1.14	0.64	-0.55	0.00	0.22	0.19	xh	Type II diabetes mellitus
01040	-1.11	-1.00	-0.63	0.02	0.18	0.27	xl	Biosynthesis of unsaturated fatty acids
03010	-1.24	-0.46	-0.47	0.00	0.66	0.48	xl	Ribosome
00251	-0.82	0.62	-0.08	0.02	0.17	0.96	xl	Glutamate metabolism
00600	-0.70	0.79	-0.05	0.17	0.05	0.96	yh	Sphingolipid metabolism
00900	-0.85	0.98	-0.11	0.17	0.01	0.94	yh	Terpenoid biosynthesis
00040	0.86	1.06	-0.48	0.30	0.01	0.41	yh	Pentose and glucuronate interconversions
05120	0.04	0.89	-0.34	0.94	0.00	0.31	yh	Epithelial cell signaling in Helicobacter pylori infection
00100	-0.72	0.74	-0.10	0.08	0.05	0.94	yh	Biosynthesis of steroids
05110	0.94	0.97	-0.62	0.10	0.01	0.07	yh	Vibrio cholerae infection
00620	0.56	-1.30	-0.04	0.52	0.00	0.98	yl	Pyruvate metabolism
00630	0.14	-1.30	-0.44	0.91	0.01	0.81	yl	Glyoxylate and dicarboxylate metabolism

Table 1: LOR: log odds ratio; p: adjusted p-value FDR

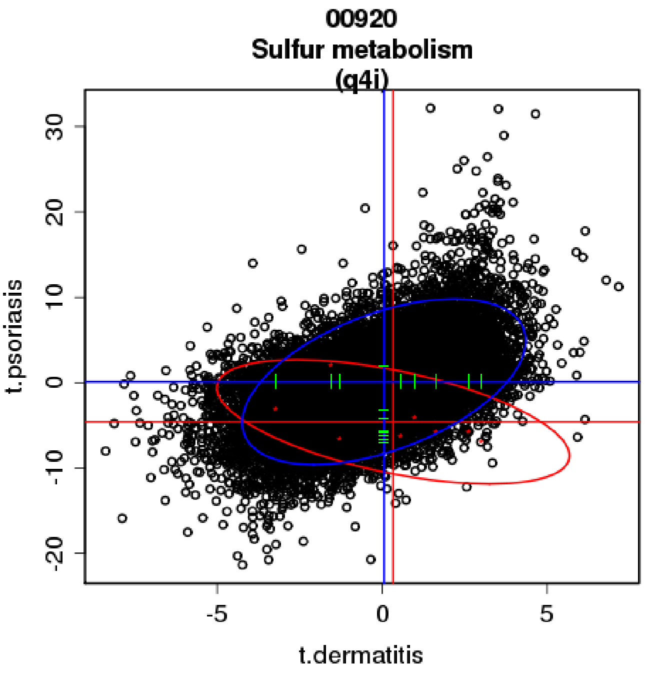

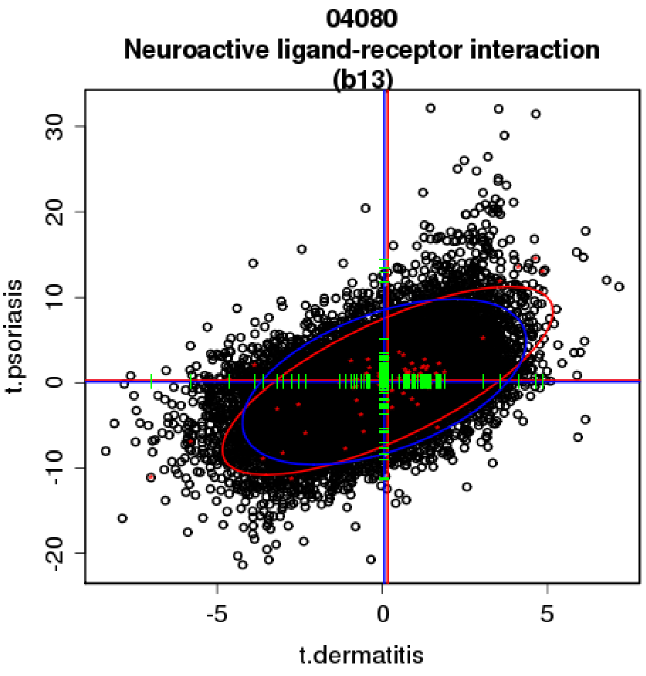

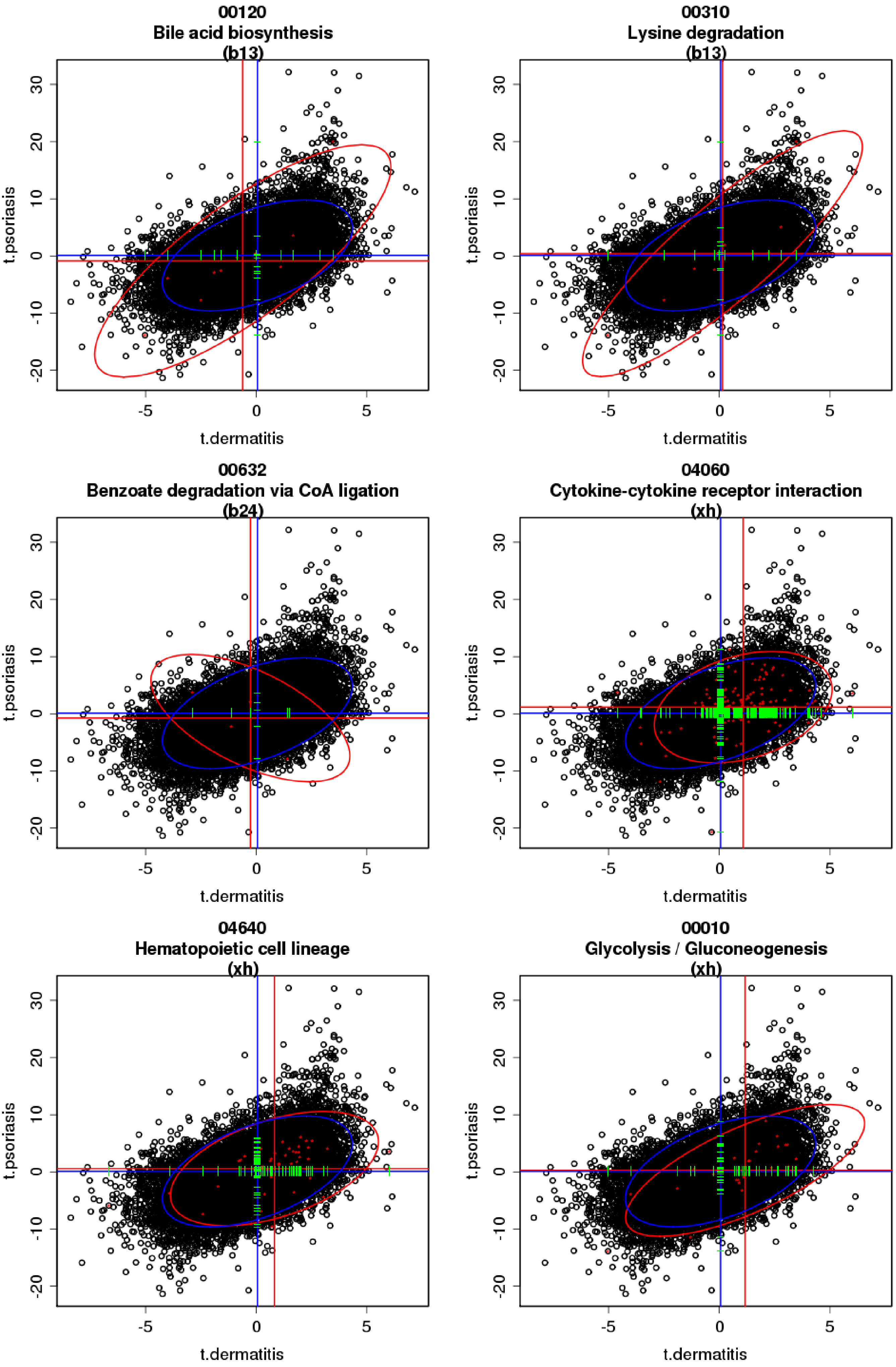
00120
Bile acid biosynthesis
(b13)
00310
Lysine degradation
(b13)
00632
Benzoate degradation via CoA ligation
(b24)
04060
Cytokine-cytokine receptor interaction
(xh)
04640
Hematopoietic cell lineage
(xh)
00010
Glycolysis / Gluconeogenesis
(xh)
t.psoriasis
t.dermatitis

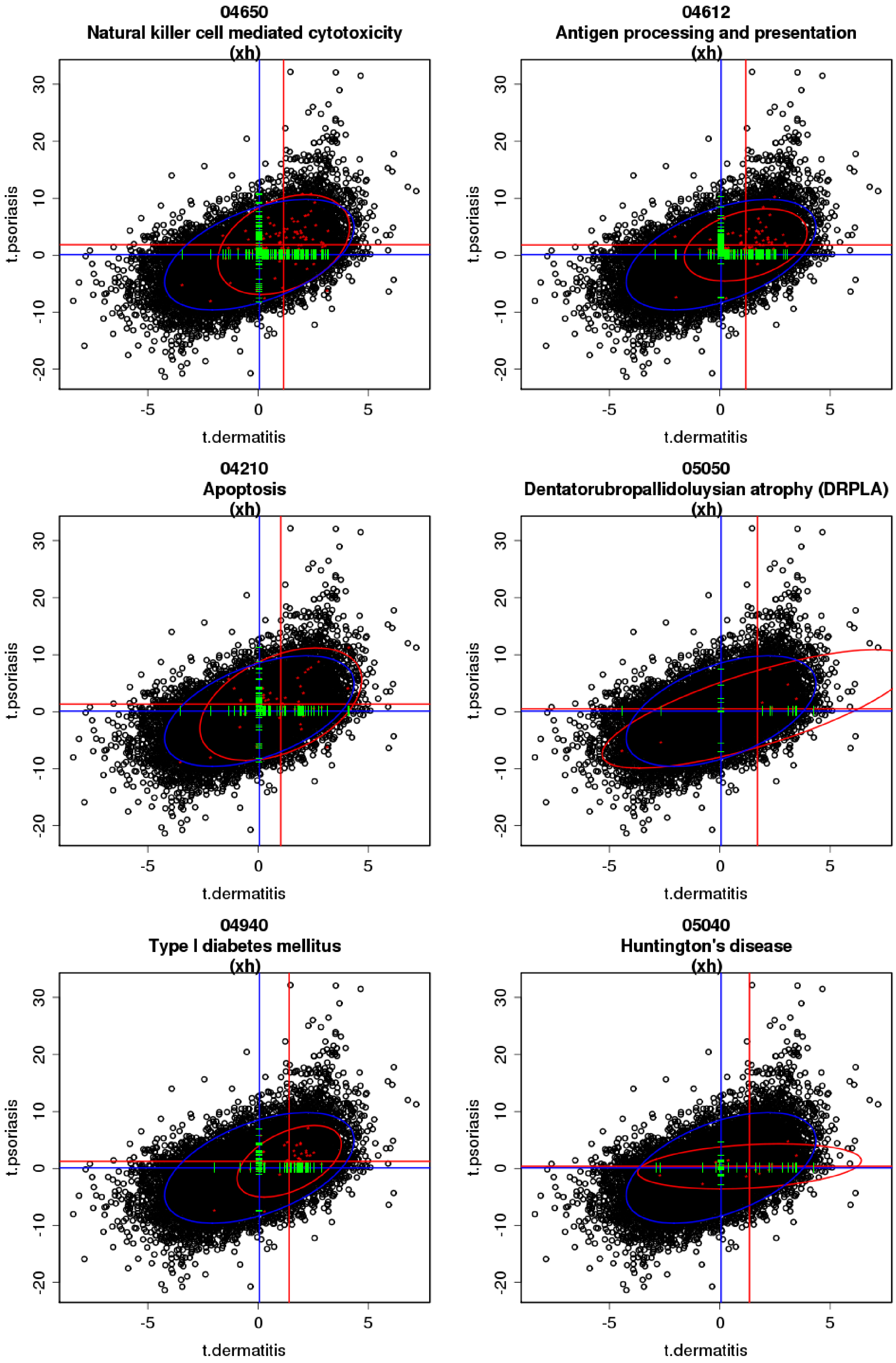
04650
Natural killer cell mediated cytotoxicity
(xh)
04612
Antigen processing and presentation
(xh)
04210
Apoptosis
(xh)
05050
Dentatorubropallidoluysian atrophy (DRPLA)
(xh)
04940
Type I diabetes mellitus
(xh)
05040
Huntington's disease
(xh)
t.psoriasis
t.dermatitis
-20
-10
0
10
20
30
-5
0
5

04662
B cell receptor signaling pathway
(xh)

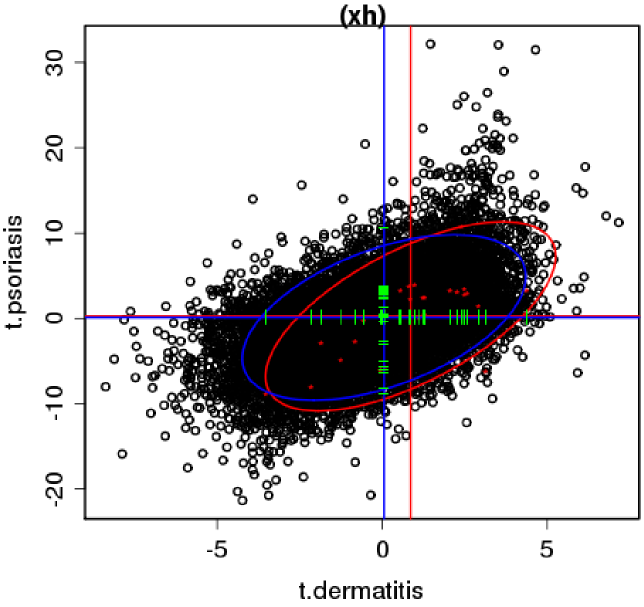

05131
Pathogenic Escherichia coli infection - EPEC
(xh)

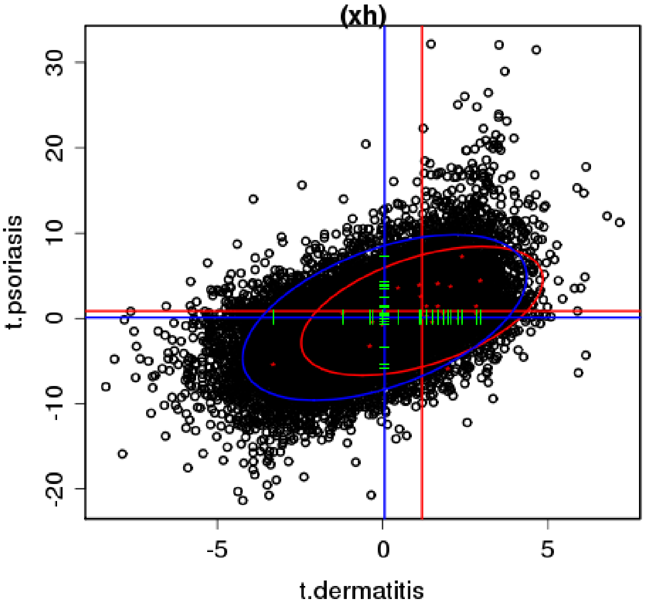

05130
Pathogenic Escherichia coli infection - EHEC
(xh)

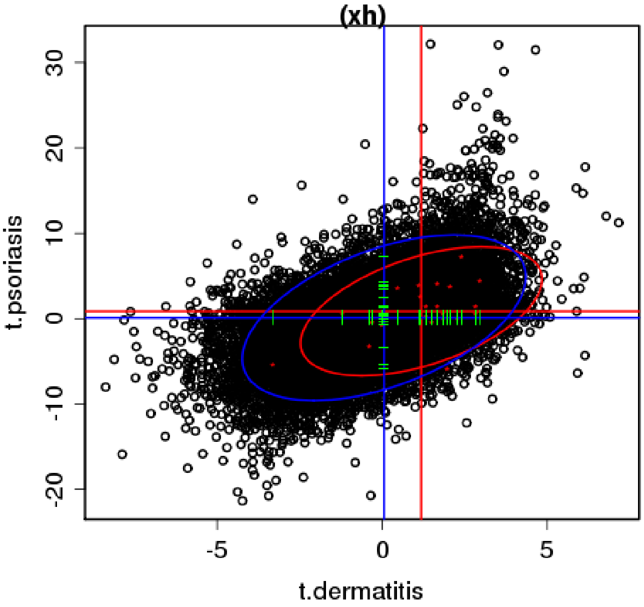

04514
Cell adhesion molecules (CAMs)
(xh)

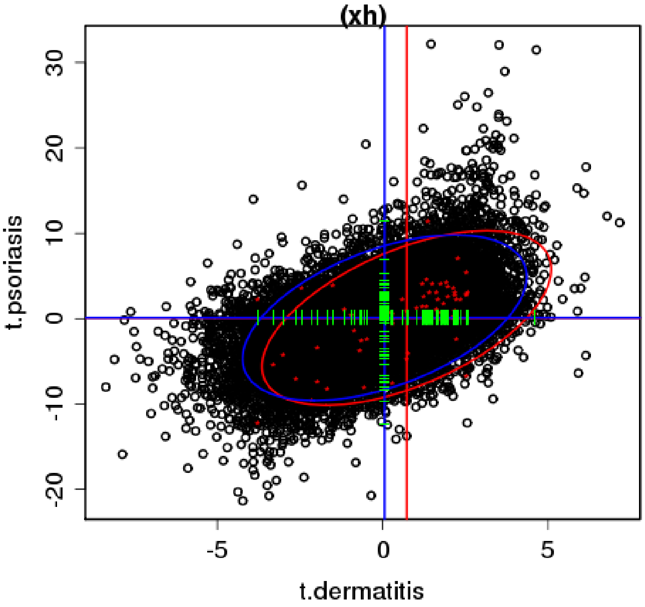

00030
Pentose phosphate pathway
(xh)

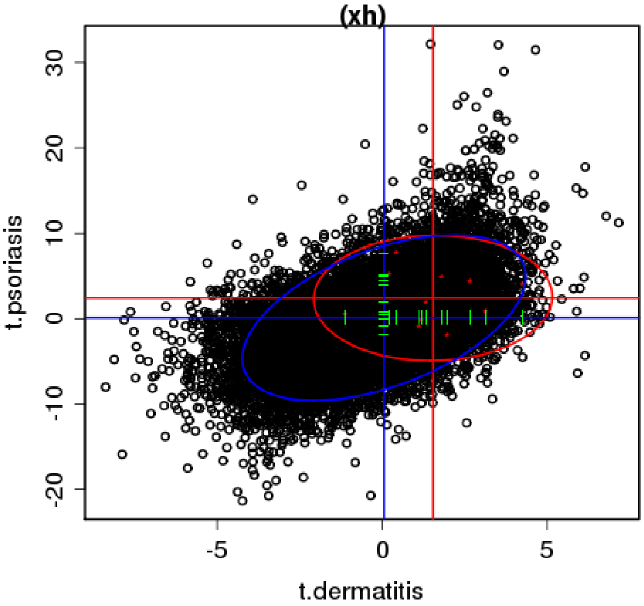

04930
Type II diabetes mellitus
(xh)

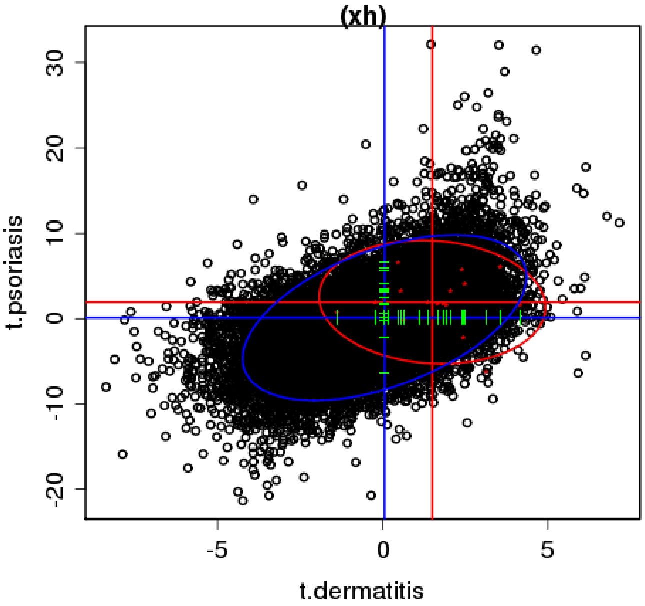

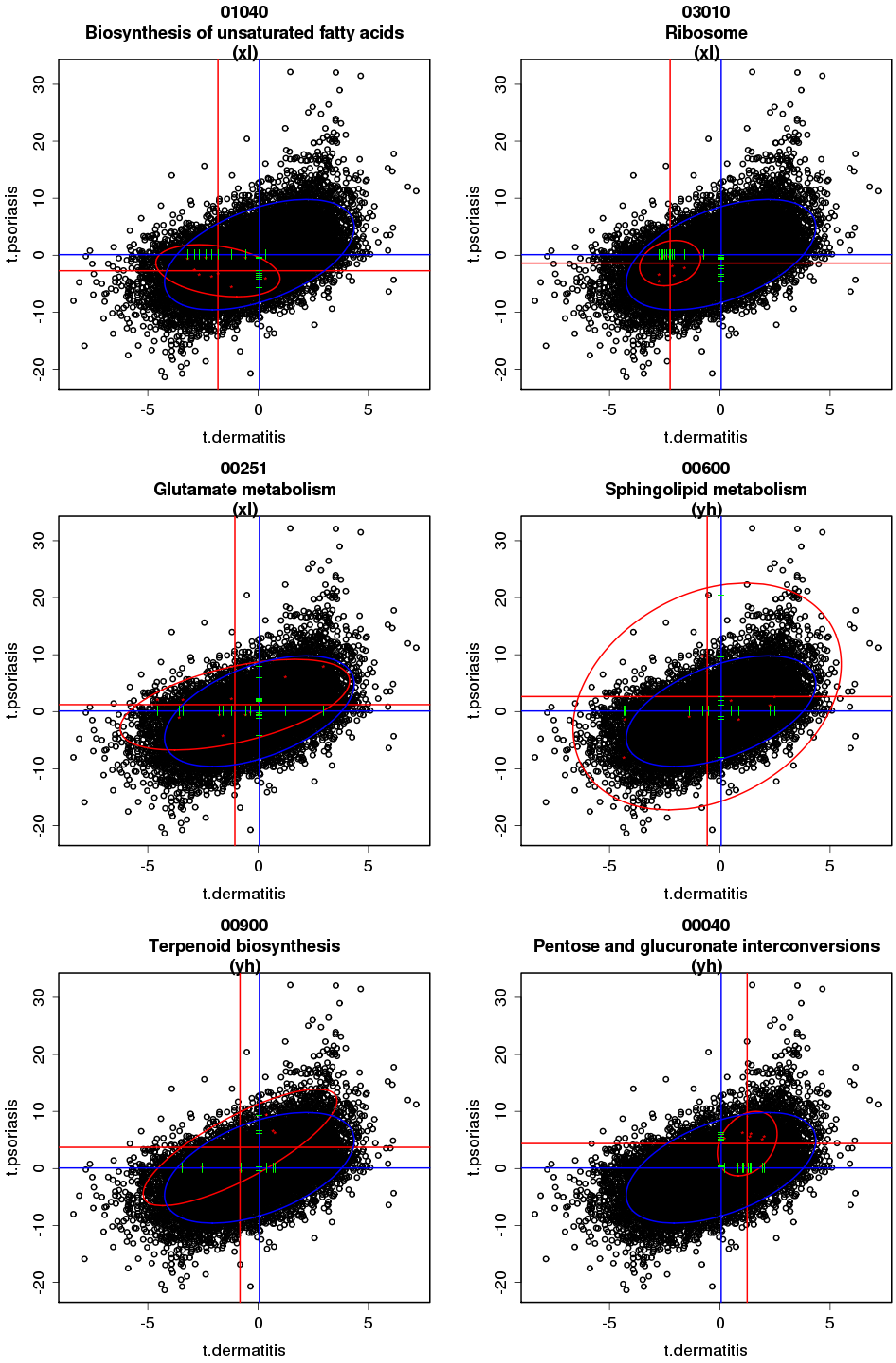
01040
Biosynthesis of unsaturated fatty acids
(xl)
03010
Ribosome
(xl)
00251
Glutamate metabolism
(xl)
00600
Sphingolipid metabolism
(yh)
00900
Terpenoid biosynthesis
(yh)
00040
Pentose and glucuronate interconversions
(yh)
t.psoriasis
t.dermatitis
-20
-10
0
10
20
30
-5
0
5

05120
Epithelial cell signaling in Helicobacter pylori infectio
(yh)

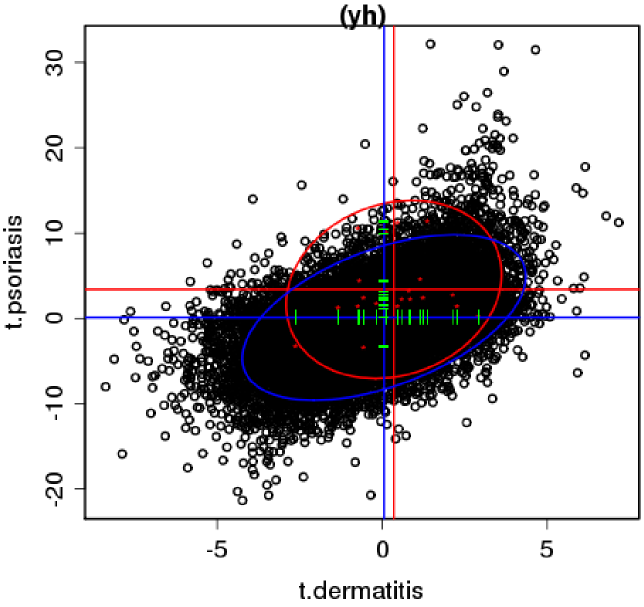

00100
Biosynthesis of steroids
(yh)

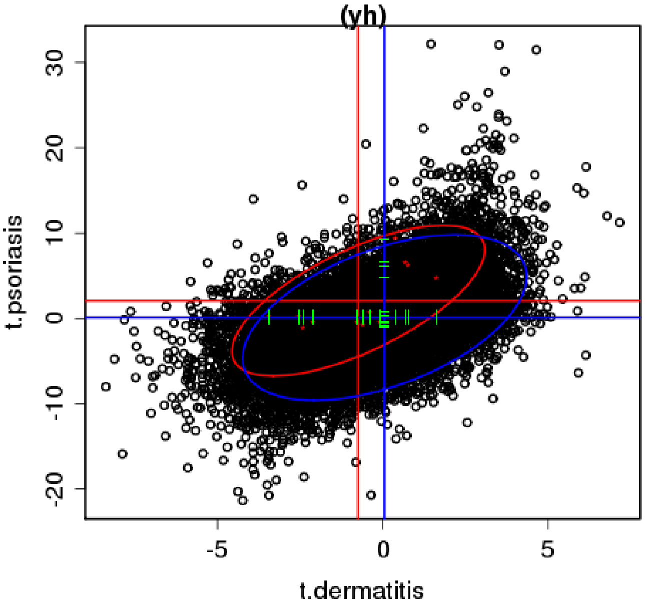

05110
Vibrio cholerae infection
(yh)

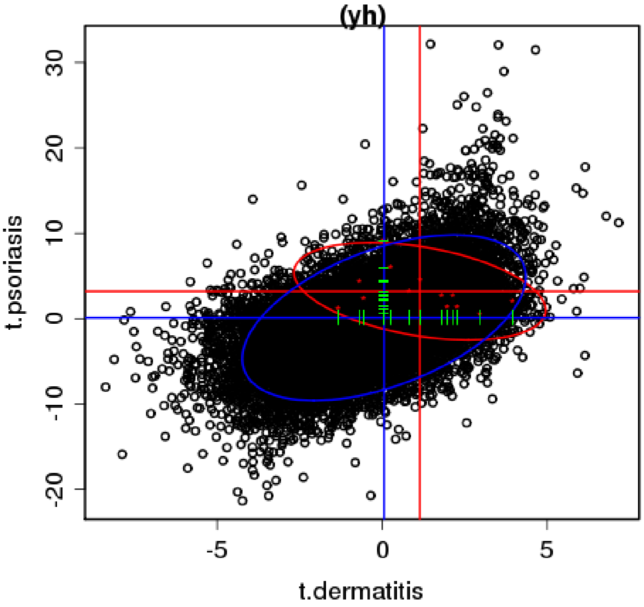

00620
Pyruvate metabolism
(yl)

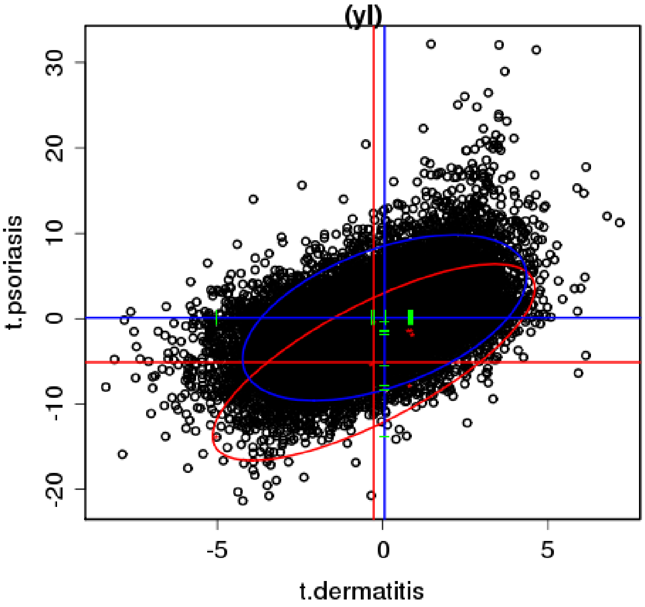

00630
Glyoxylate and dicarboxylate metabolism
(yl)

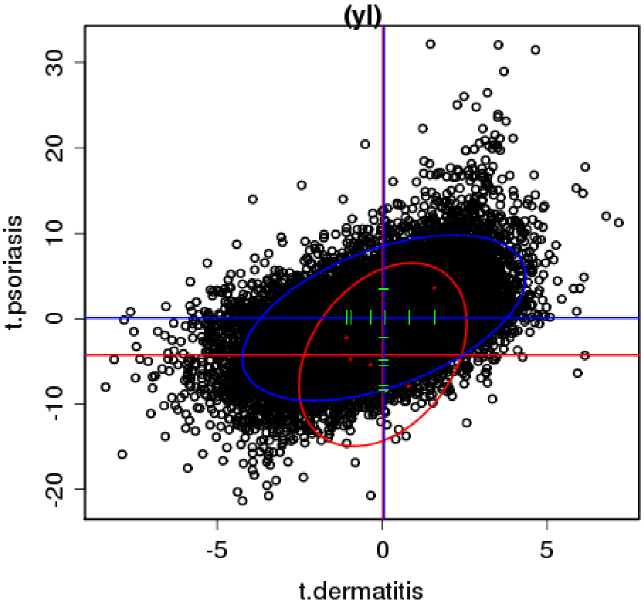

Supplement: File S3 — A) GO Biological Process terms, and B) KEGG pathways, significant for differential gene expression in dermatitis and psoriasis case-control studies and their interaction, along with the corresponding graphical representations. In the plots blue lines intersect in the mean of the distribution of all the values and red lines intersect in the mean of the distribution of values of the genes corresponding to the term analysed. Blue ellipse delimits the confidence interval for all the values and red ellipse delimits the confidence interval for the term analysed. The red ellipse marks the trend of the relationship between both variables. (30.60 MB ZIP) [file pone.0010348.s009.zip › Montaner_File_S3B-KEGG_dermatitis_and_psoriasis.pdf]
