# Supplementary material for: Multidimensional Gene Set Analysis of Genomic Data
Source: PLoS One. 2010 Apr 27;5(4):e10348. doi: 10.1371/journal.pone.0010348 (PMC2860497; doi:10.1371/journal.pone.0010348)

Combined functional profiling of SNP association and copy number in lumminal B tumours using GO Biological Process

8 significant modules

LOR indicates Log Odds Ratio of the coefficient or the interaction

p indicates FDR adjusted p-value

	LOR cgems	LOR cn luminalB	LOR Inter	p cgems	p cn luminalB	p Inter	pattern	name
GO:0015807	-0.09	-0.85	-0.59	0.98	0.46	0.04	b24	L-amino acid transport
GO:0032228	-0.63	-1.21	-0.68	0.65	0.24	0.01	b24	regulation of synaptic transmission, GABAergic
GO:0050805	-0.94	-1.24	-0.63	0.22	0.24	0.04	b24	negative regulation of synaptic transmission
GO:0051932	-0.82	-1.35	-0.67	0.49	0.17	0.02	b24	synaptic transmission, GABAergic
GO:0042398	-0.77	-0.02	0.12	0.04	0.99	1.00	xl	amino acid derivative biosynthetic process
GO:0042401	-0.93	0.12	0.20	0.01	0.98	1.00	xl	biogenic amine biosynthetic process
GO:0030216	0.20	0.41	-0.03	0.80	0.03	1.00	yh	keratinocyte differentiation
GO:0031424	0.29	0.59	-0.01	0.81	0.00	1.00	yh	keratinization

Table 1: LOR: log odds ratio; p: adjusted p-value FDR

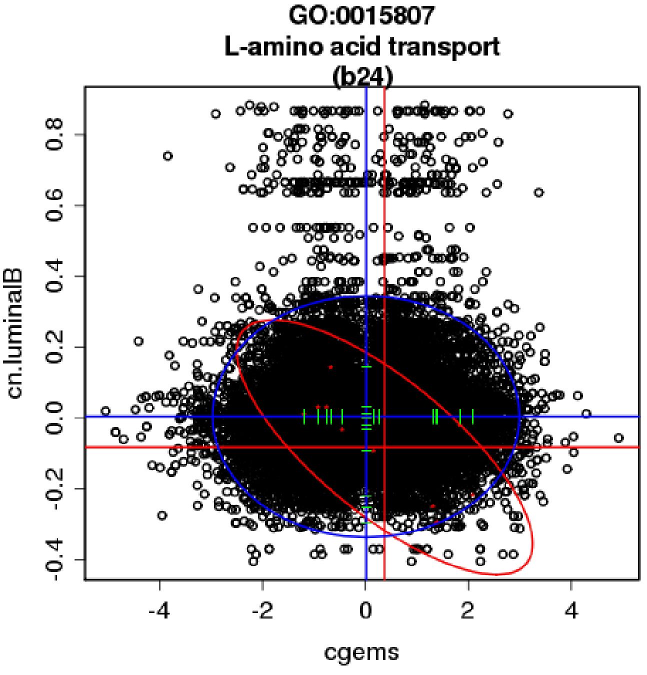

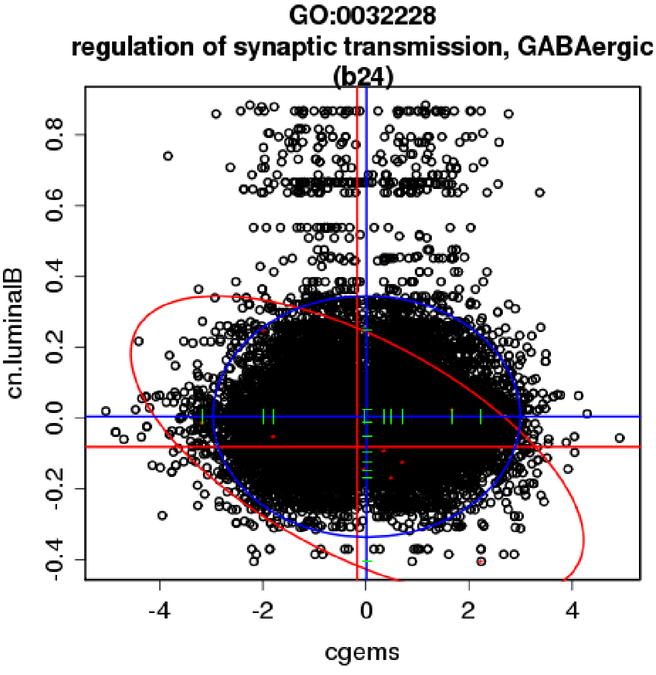

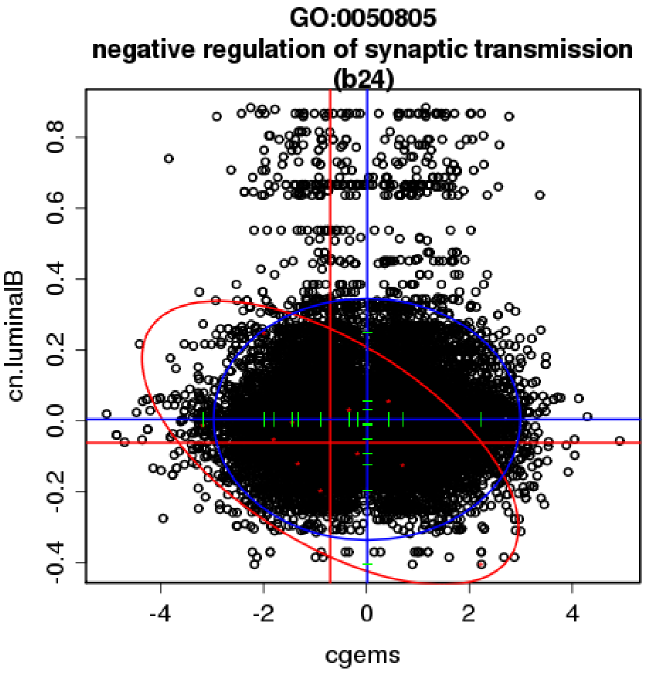
GO:0050805
negative regulation of synaptic transmission
(b24)
cn.luminalB
cgems

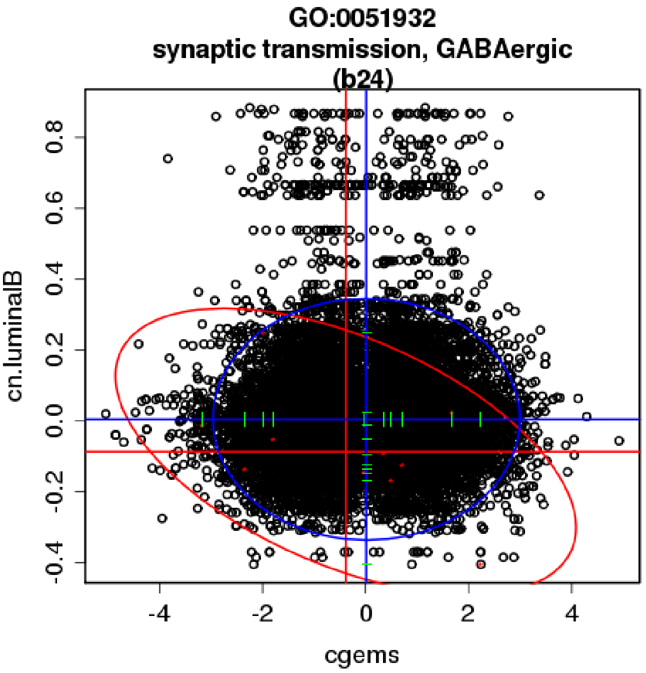
GO:0051932
synaptic transmission, GABAergic
(b24)
cn.luminalB
cgems

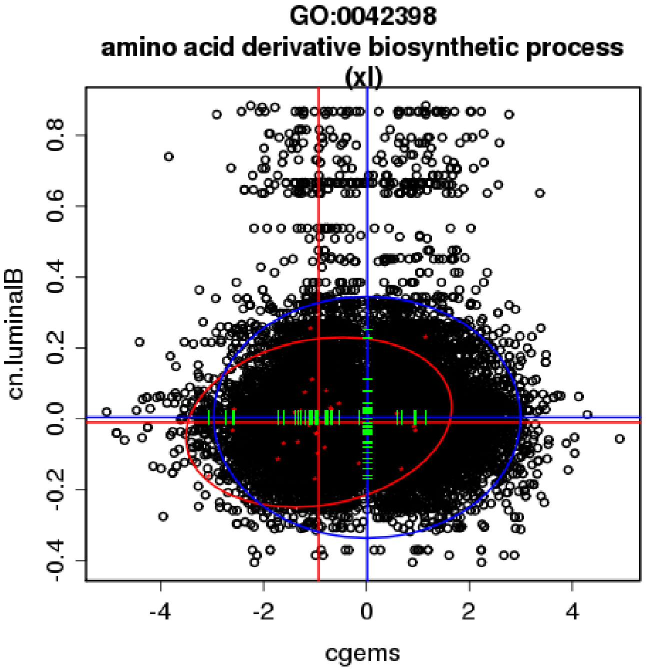
GO:0042398
amino acid derivative biosynthetic process
(xl)
cn.luminalB
cgems

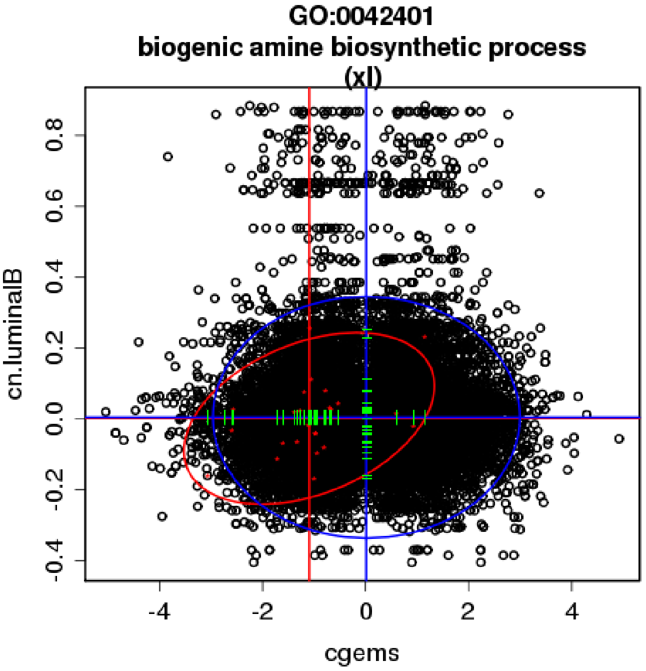
GO:0042401
biogenic amine biosynthetic process
(xl)
cn.luminalB
cgems

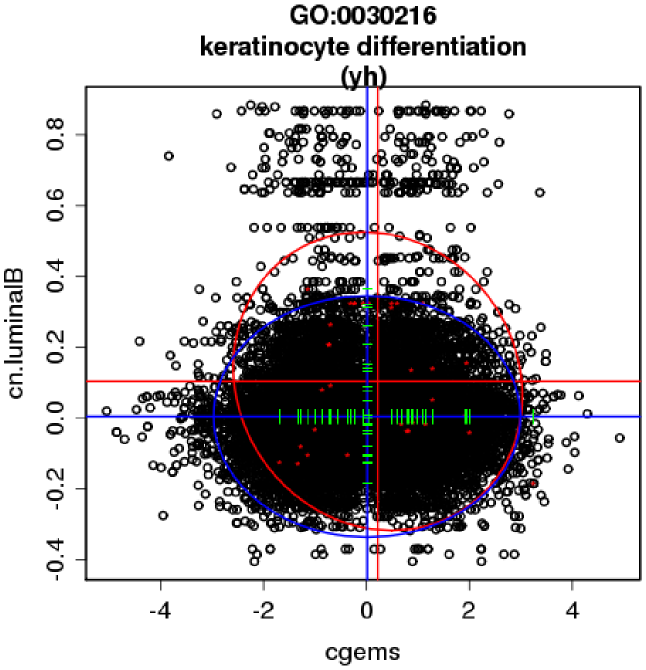
GO:0030216
keratinocyte differentiation
(yh)
cn.luminalB
cgems

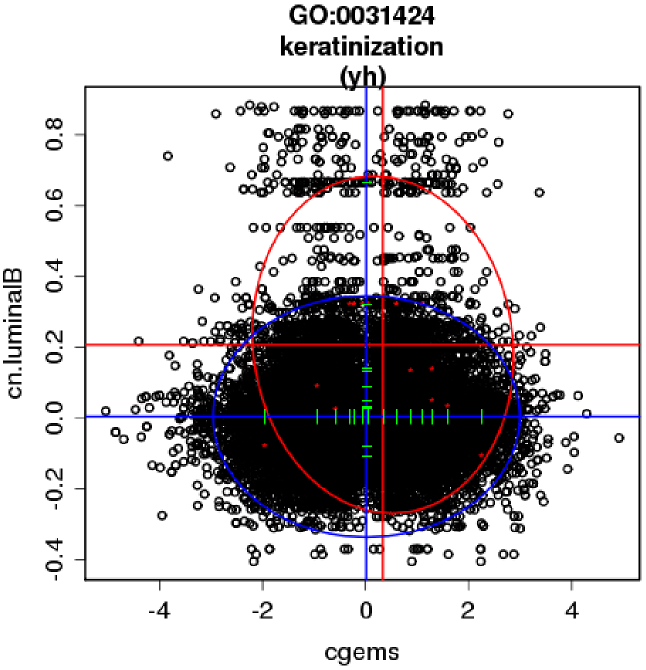
GO:0031424
keratinization
(yh)
cn.luminalB
cgems

Combined functional profiling of SNP association and copy number in lumminal B tumours using KEGG pathways

1 significant modules

LOR indicates Log Odds Ratio of the coefficient or the interaction

p indicates FDR adjusted p-value

	LOR cgems	LOR cn luminalB	LOR Inter	p cgems	p cn luminalB	p Inter	pattern	name
hsa05217	-0.28	-0.11	0.56	0.59	0.89	0.01	b13	Basal cell carcinoma

Table 1: LOR: log odds ratio; p: adjusted p-value FDR

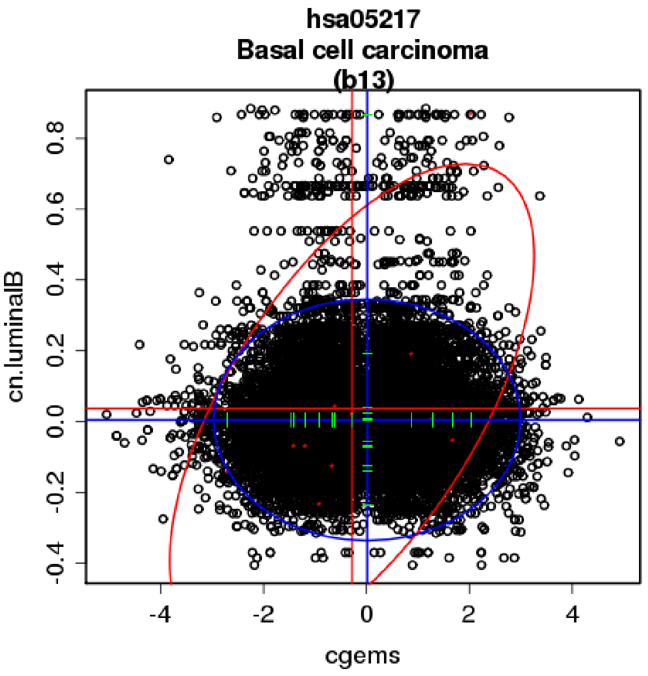

Supplement: File S4 — A) GO Biological Process terms, and B) KEGG pathways, significant for gene association (derived from genotyping, see text) association data and genomic copy number in breast cancer and their interaction, along with the corresponding graphical representations. In the plots blue lines intersect in the mean of the distribution of all the values and red lines intersect in the mean of the distribution of values of the genes corresponding to the term analysed. Blue ellipse delimits the confidence interval for all the values and red ellipse delimits the confidence interval for the term analysed. The red ellipse marks the trend of the relationship between both variables. (0.80 MB PDF) [file pone.0010348.s010.pdf]
