# Supplementary material for: Multidimensional Gene Set Analysis of Genomic Data
Source: PLoS One. 2010 Apr 27;5(4):e10348. doi: 10.1371/journal.pone.0010348 (PMC2860497; doi:10.1371/journal.pone.0010348)
Supplement: File S5 — A) GO Biological Process terms, and B) KEGG pathways, significant for prognosis and differential expression in a case-control study of breast cancer and their interaction, along with the corresponding graphical representations. In the plots blue lines intersect in the mean of the distribution of all the values and red lines intersect in the mean of the distribution of values of the genes corresponding to the term analysed. Blue ellipse delimits the confidence interval for all the values and red ellipse delimits the confidence interval for the term analysed. The red ellipse marks the trend of the relationship between both variables. (2.22 MB PDF) [file pone.0010348.s011.pdf]

Combined functional profiling of differential gene expression and prognosis using GO Biological Process

29 significant modules

LOR indicates Log Odds Ratio of the coefficient or the interaction

p indicates FDR adjusted p-value

	LOR normal vs tumor	LOR prognosis	LOR Inter	p normal vs tumor	p prognosis	p Inter	pattern	name
GO:0000087	-0.45	-0.08	-0.42	0.01	0.81	0.00	q2i	M phase of mitotic cell cycle
GO:0000279	-0.53	-0.07	-0.38	0.04	0.85	0.00	q2i	M phase
GO:0000910	-0.27	-0.09	-0.57	0.01	0.95	0.00	q2i	cytokinesis
GO:0007067	-0.47	-0.07	-0.40	0.04	0.90	0.00	q2i	mitosis
GO:0022618	-0.22	-0.33	-0.42	0.03	0.21	0.00	q2i	ribonucleoprotein complex assembly
GO:0051301	-0.38	-0.00	-0.38	0.01	0.99	0.00	q2i	cell division
GO:0051726	-0.01	0.05	-0.22	0.03	0.91	0.01	q2i	regulation of cell cycle
GO:0045638	0.09	-0.35	-0.60	0.01	0.65	0.04	q4i	negative regulation of myeloid cell differentiation
GO:0000226	-0.08	0.16	-0.31	0.11	0.47	0.02	b24	microtubule cytoskeleton organization and biogenesis
GO:0000278	-0.34	0.04	-0.28	0.11	0.94	0.00	b24	mitotic cell cycle
GO:0007346	-0.30	-0.08	-0.39	0.07	0.90	0.00	b24	regulation of mitotic cell cycle
GO:0022403	-0.42	-0.00	-0.31	0.09	0.99	0.00	b24	cell cycle phase
GO:0042254	-0.40	-0.45	-0.42	0.19	0.10	0.01	b24	ribosome biogenesis
GO:0006412	0.06	-0.28	-0.20	0.02	0.01	0.07	q4f	translation
GO:0006414	0.45	-1.12	-0.43	0.00	0.00	0.28	q4f	translational elongation
GO:0042312	0.45	0.08	-0.51	0.03	0.97	0.22	xh	regulation of vasodilation
GO:0000209	-0.25	0.55	0.13	0.94	0.01	1.00	yh	protein polyubiquitination
GO:0006066	0.08	0.20	-0.02	0.97	0.02	1.00	yh	alcohol metabolic process
GO:0010033	0.05	0.29	0.00	0.99	0.02	1.00	yh	response to organic substance
GO:0032944	-0.17	-0.70	0.06	0.97	0.02	1.00	yl	regulation of mononuclear cell proliferation
GO:0042098	-0.18	-0.61	0.08	0.95	0.04	1.00	yl	T cell proliferation
GO:0042110	0.03	-0.38	0.14	0.75	0.03	0.86	yl	T cell activation
GO:0042129	-0.33	-0.74	-0.02	0.99	0.05	1.00	yl	regulation of T cell proliferation
GO:0045321	-0.04	-0.28	0.06	0.92	0.03	1.00	yl	leukocyte activation
GO:0046649	-0.06	-0.33	0.07	0.89	0.02	1.00	yl	lymphocyte activation
GO:0046651	-0.19	-0.49	-0.05	0.99	0.05	1.00	yl	lymphocyte proliferation
GO:0050670	-0.17	-0.70	0.06	0.97	0.02	1.00	yl	regulation of lymphocyte proliferation
GO:0051249	-0.06	-0.44	0.24	0.52	0.04	0.71	yl	regulation of lymphocyte activation
GO:0022613	-0.32	-0.35	-0.39	0.01	0.03	0.00	no_rel	ribonucleoprotein complex biogenesis and assembly

Table 1: LOR: log odds ratio; p: adjusted p-value FDR

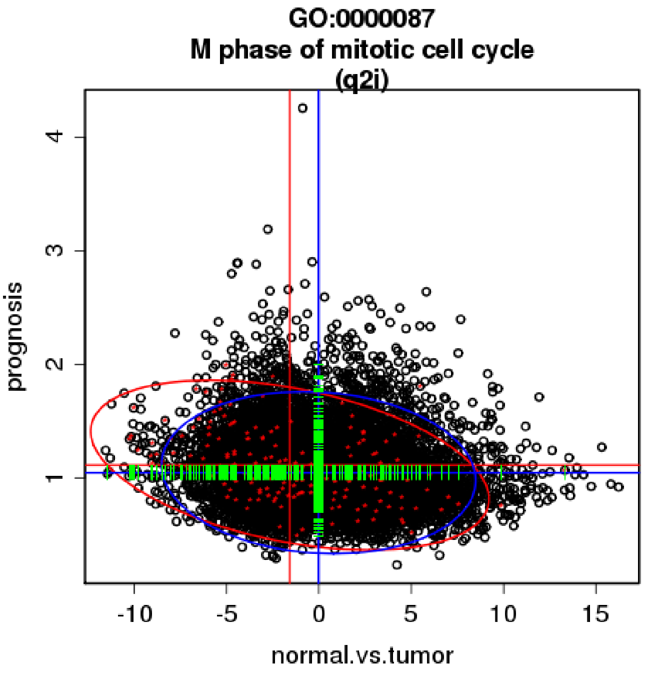

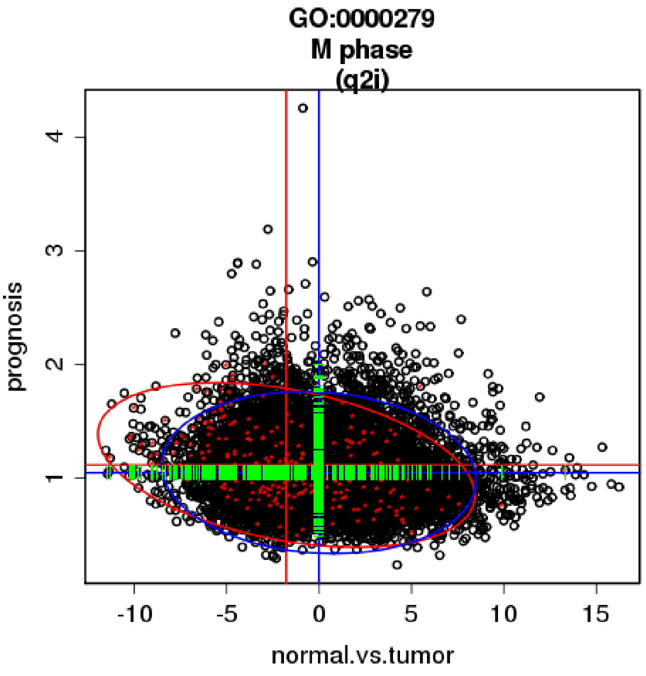

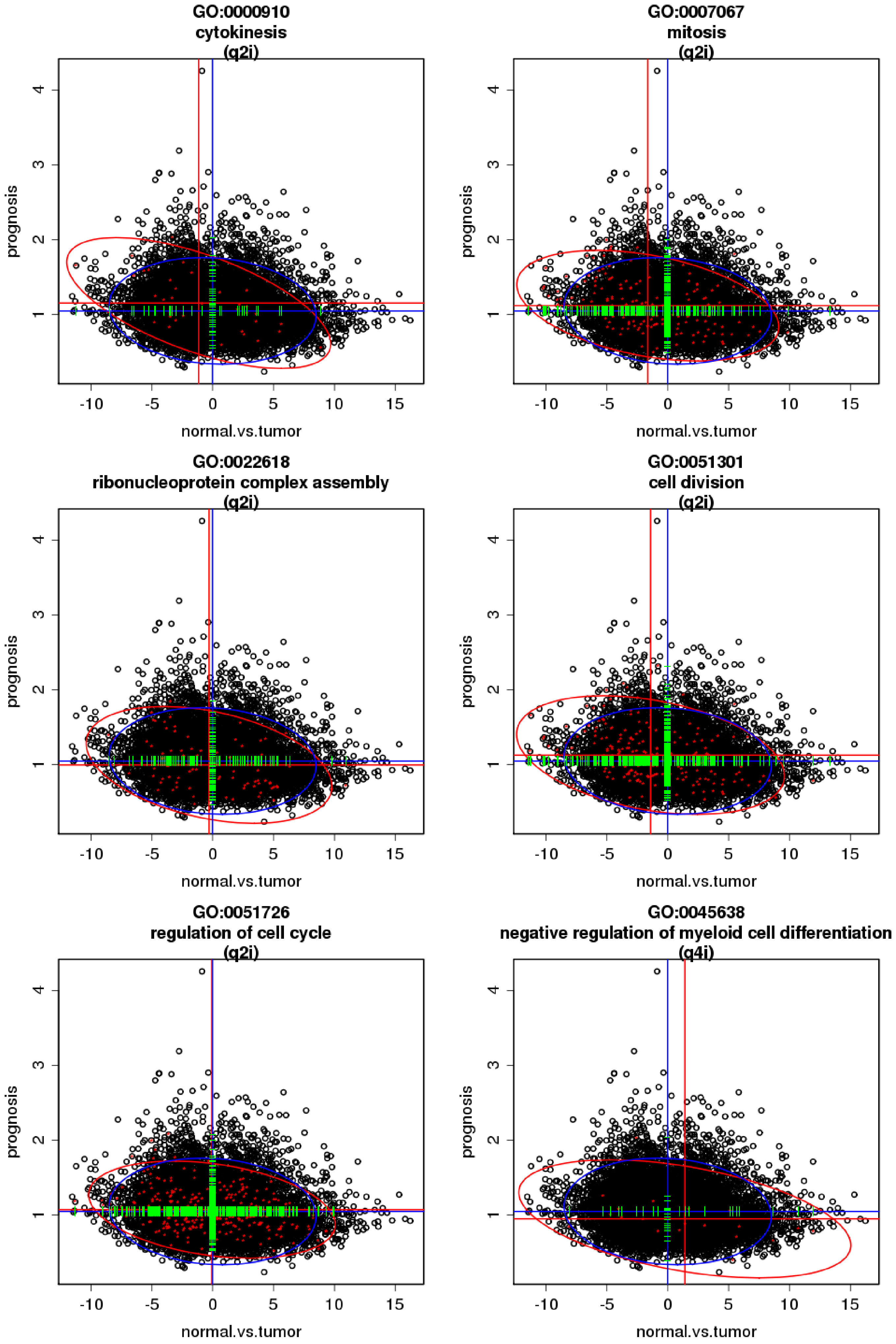
GO:0000910
cytokinesis
(q2i)
GO:0007067
mitosis
(q2i)
GO:0022618
ribonucleoprotein complex assembly
(q2i)
GO:0051301
cell division
(q2i)
GO:0051726
regulation of cell cycle
(q2i)
GO:0045638
negative regulation of myeloid cell differentiation
(q4i)
prognosis
normal.vs.tumor
-10
-5
0
5
10
15
1
2
3
4

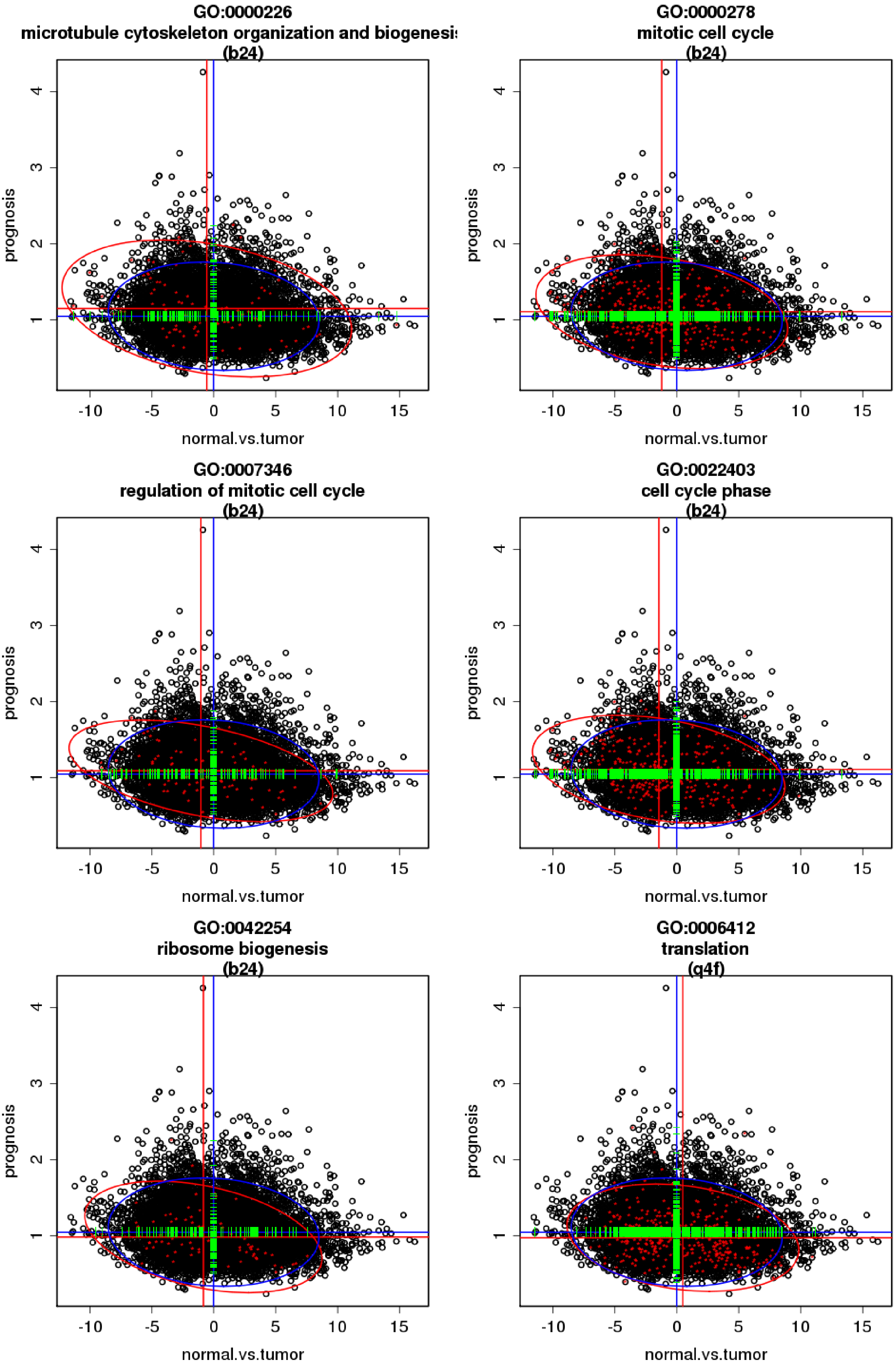

GO:0000226
microtubule cytoskeleton organization and biogenesis
(b24)
GO:0000278
mitotic cell cycle
(b24)
GO:0007346
regulation of mitotic cell cycle
(b24)
GO:0022403
cell cycle phase
(b24)
GO:0042254
ribosome biogenesis
(b24)
GO:0006412
translation
(q4f)
prognosis
normal.vs.tumor
-10
-5
0
5
10
15
1
2
3
4

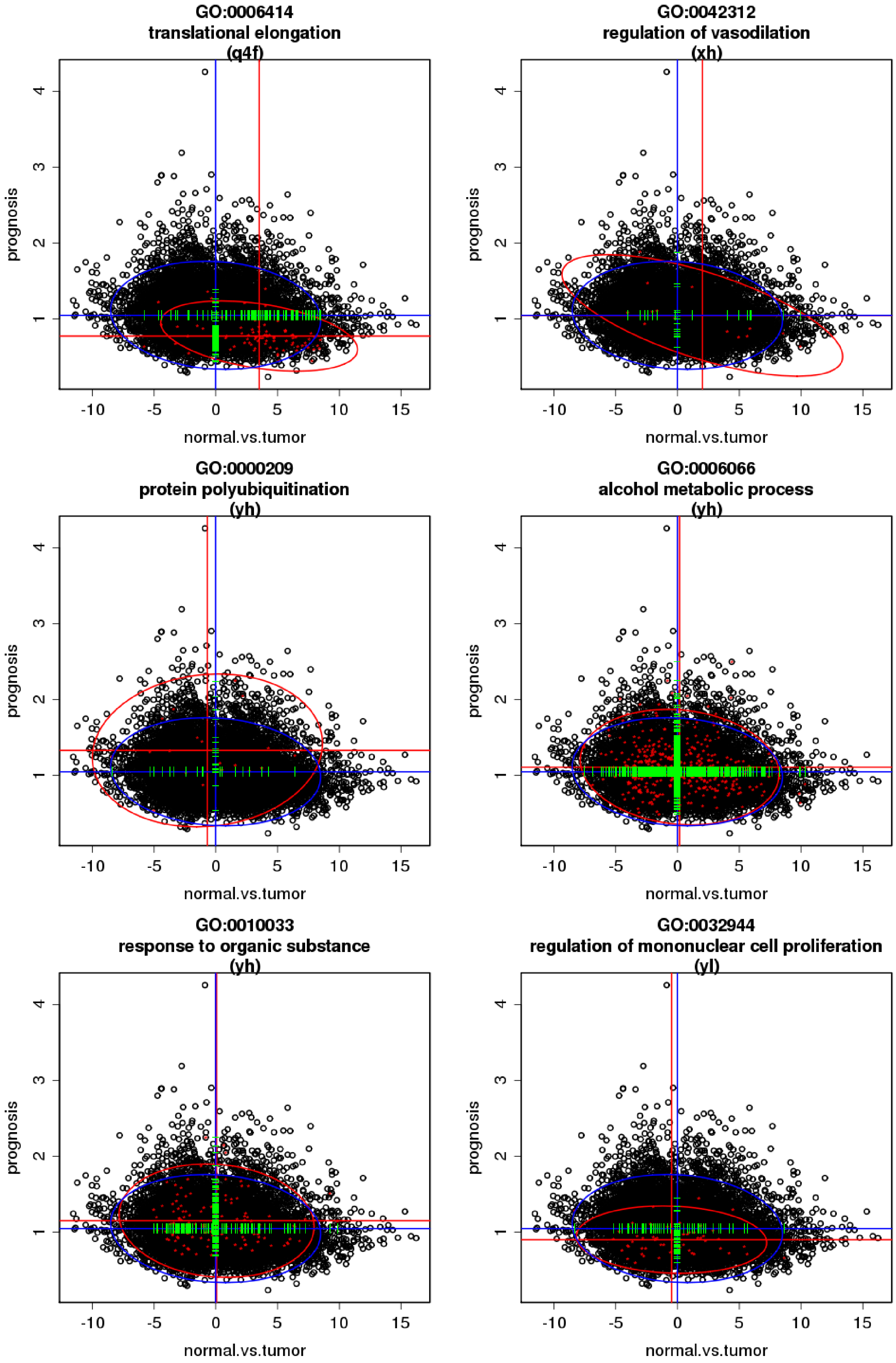
GO:0006414
translational elongation
(q4f)
GO:0042312
regulation of vasodilation
(xh)
GO:0000209
protein polyubiquitination
(yh)
GO:0006066
alcohol metabolic process
(yh)
GO:0010033
response to organic substance
(yh)
GO:0032944
regulation of mononuclear cell proliferation
(yl)
prognosis
normal.vs.tumor
-10
-5
0
5
10
15
1
2
3
4

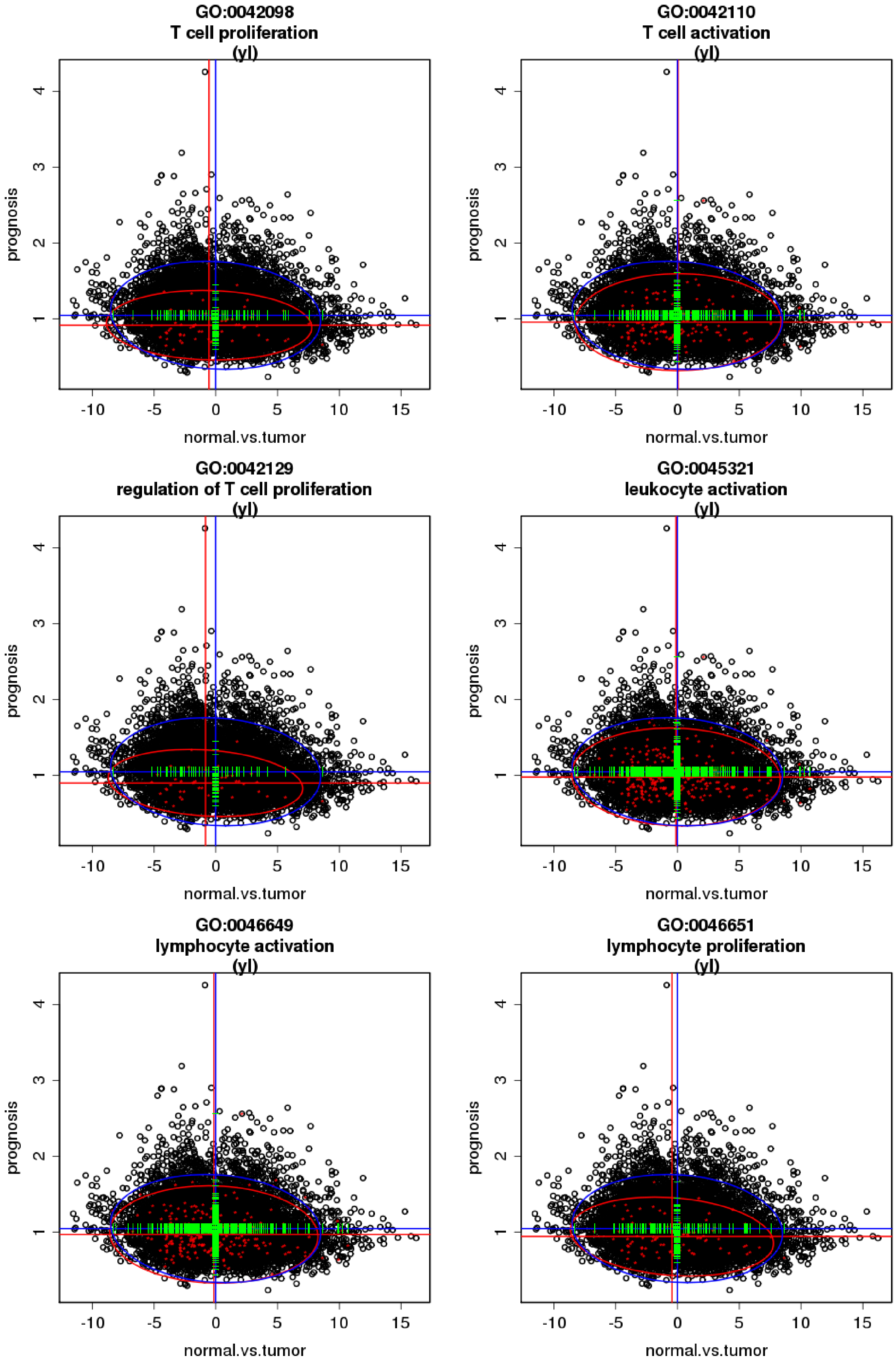
GO:0042098
T cell proliferation
(yl)
GO:0042110
T cell activation
(yl)
GO:0042129
regulation of T cell proliferation
(yl)
GO:0045321
leukocyte activation
(yl)
GO:0046649
lymphocyte activation
(yl)
GO:0046651
lymphocyte proliferation
(yl)
prognosis
normal.vs.tumor
-10
-5
0
5
10
15
1
2
3
4

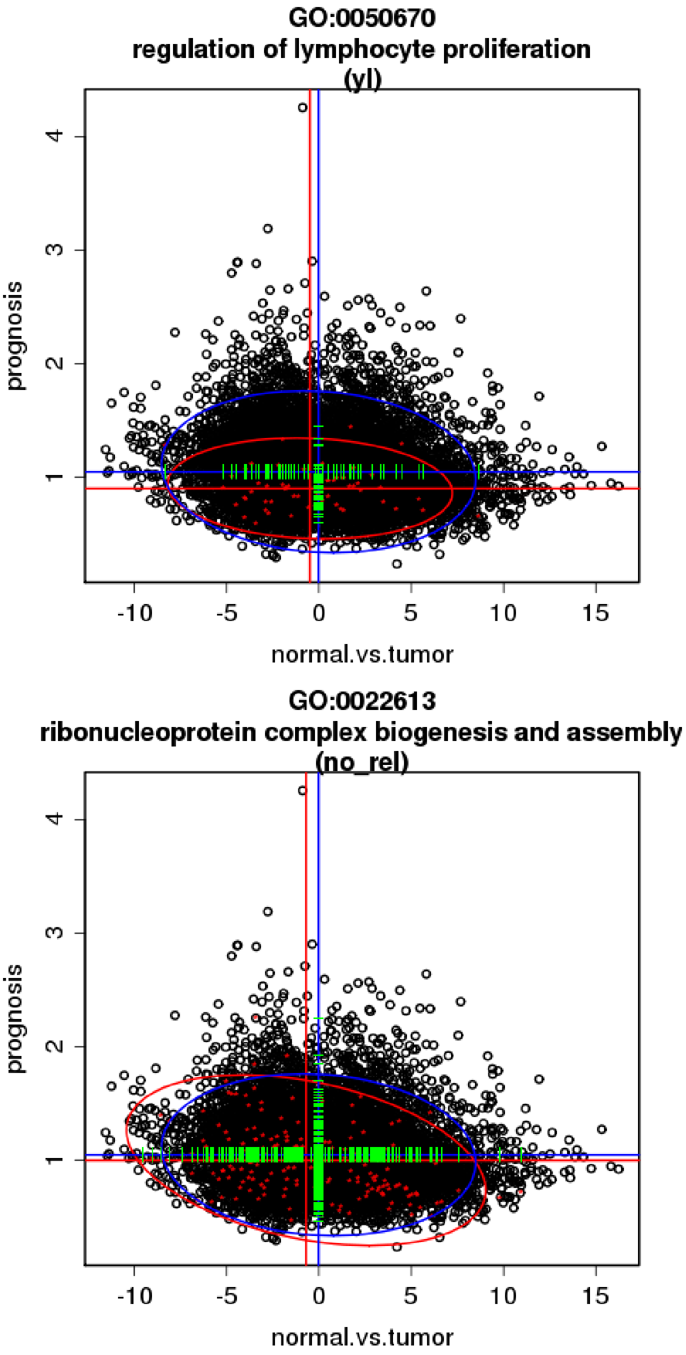
GO:0050670
regulation of lymphocyte proliferation
(yl)
prognosis
normal.vs.tumor
-10
-5
0
5
10
15
1
2
3
4
GO:0051249
regulation of lymphocyte activation
(yl)
prognosis
normal.vs.tumor
GO:0022613
ribonucleoprotein complex biogenesis and assembly
(no_rel)
prognosis
normal.vs.tumor

Combined functional profiling of differential gene expression and prognosis using KEGG pathways

6 significant modules

LOR indicates Log Odds Ratio of the coefficient or the interaction

p indicates FDR adjusted p-value

	LOR normal vs tumor	LOR prognosis	LOR Inter	p normal vs tumor	p prognosis	p Inter	pattern	name
hsa04660	-0.26	-0.18	0.53	0.03	0.81	0.04	q3i	T cell receptor signaling pathway
hsa04662	-0.36	-0.05	0.58	0.03	0.90	0.04	q3i	B cell receptor signaling pathway
hsa04150	0.67	0.30	-0.48	0.00	0.57	0.05	q4i	mTOR signaling pathway
hsa04140	-0.77	-0.85	-0.78	0.10	0.31	0.01	b24	Regulation of autophagy
hsa04370	-0.55	0.55	0.29	0.43	0.01	0.75	yh	VEGF signaling pathway
hsa03010	0.11	-1.65	-0.49	0.43	0.04	0.75	yl	Ribosome

Table 1: LOR: log odds ratio; p: adjusted p-value FDR

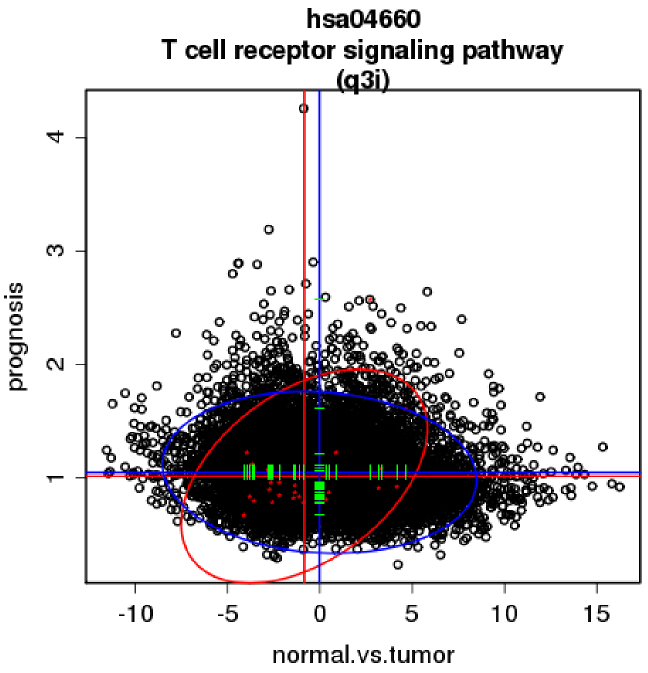

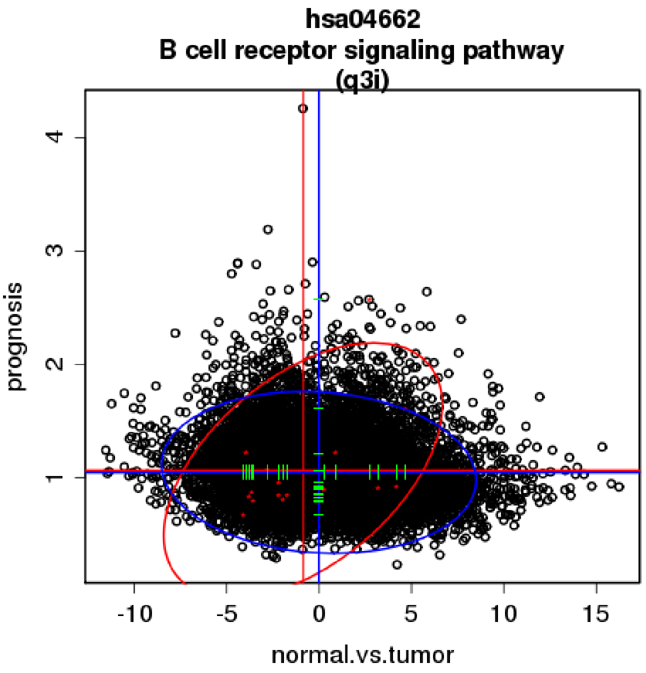

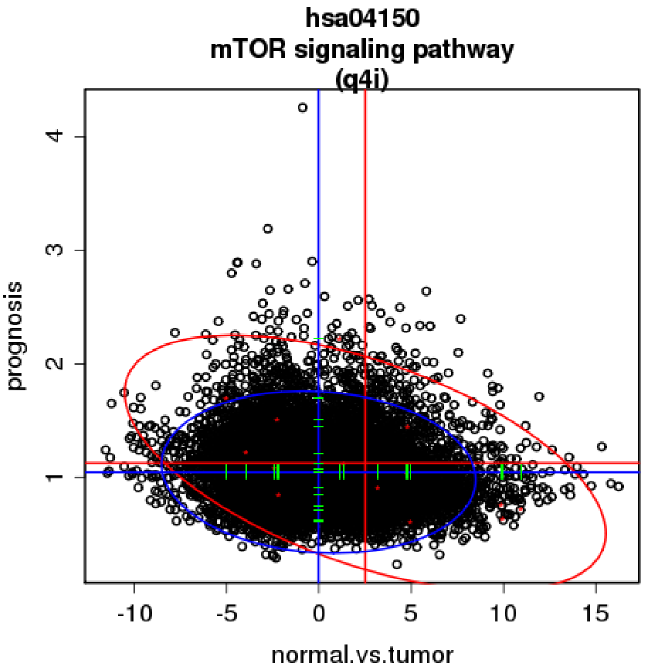
hsa04150
mTOR signaling pathway
(q4i)
prognosis
normal.vs.tumor

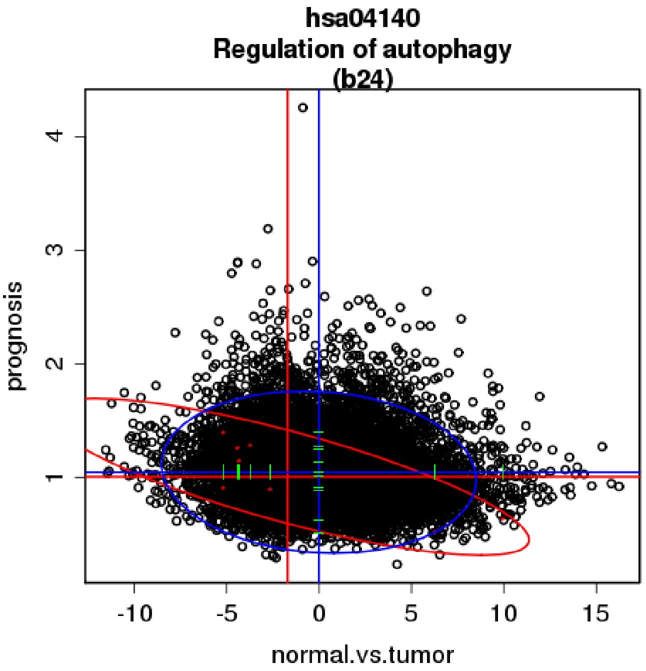
hsa04140
Regulation of autophagy
(b24)
prognosis
normal.vs.tumor

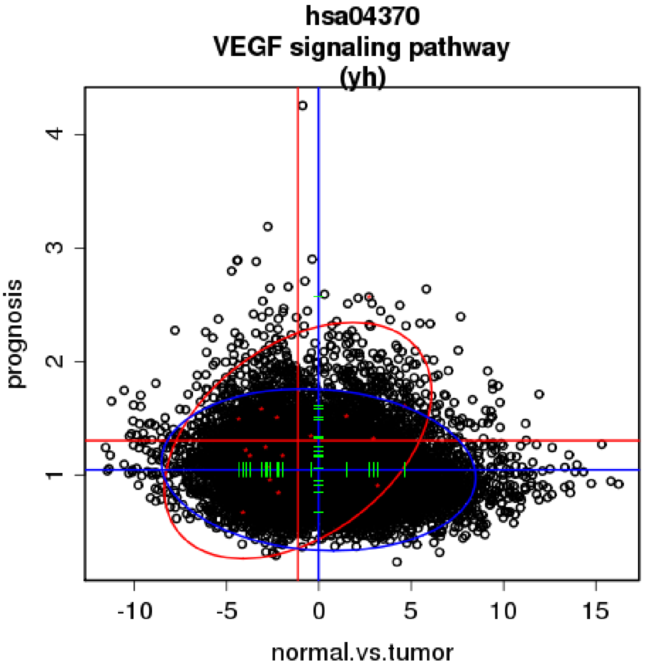
hsa04370
VEGF signaling pathway
(yh)
prognosis
normal.vs.tumor

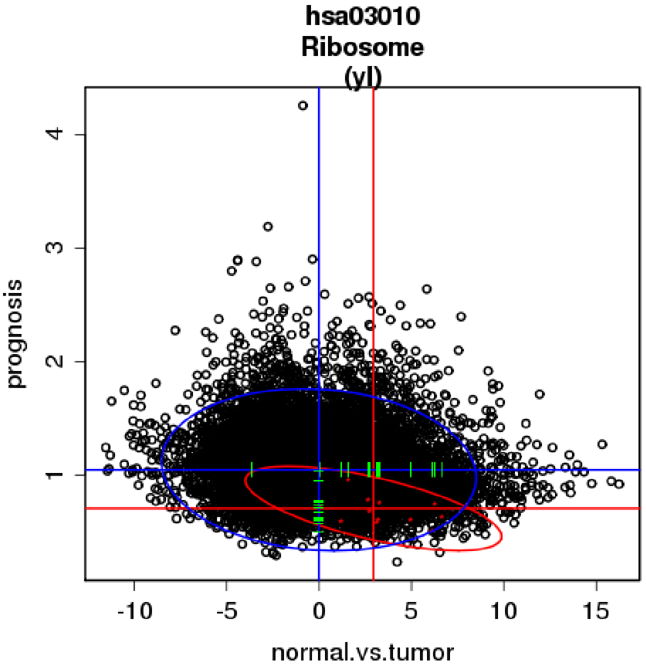
hsa03010
Ribosome
(yl)
prognosis
normal.vs.tumor
