# Supplementary material for: Multidimensional Gene Set Analysis of Genomic Data
Source: PLoS One. 2010 Apr 27;5(4):e10348. doi: 10.1371/journal.pone.0010348 (PMC2860497; doi:10.1371/journal.pone.0010348)
Supplement: File S6 — Interaction simulation study. A simulation study of the bias in p-value estimates for the interaction term of the bivariate logistic model. (0.15 MB DOC) [file pone.0010348.s012.doc]

**Multi Dimensional Gene Set Analysis**

David Montaner and Joaquín Dopazo.

Supplementary material

File S6

**A simulation study of the bias in p-value estimates**

**for the interaction term of the bivariate logistic model.**

Montaner et al. [1] collected microarray data for 3034 human samples measured under the most diverse biological conditions. They combined all information into a huge data matrix of gene expressions and used it to estimate internal correlation of Gene Ontology Biological Process terms.

We use this same data set to simulate microarray case-control experiments where differentially expressed genes do not exist but the real biological correlation structure between genes is preserved. We applied our Multi Dimensional Gene Set Analysis to these simulated data sets in order to estimate false positive rates for the interaction term and their relationship with the internal correlation of the tested GO term.

500 data sets where randomly sampled (with no replacement) from the 3034 available microarrays. Each of these 500 datasets was constituted by:

- 10 arrays randomly labeled as *cases* within the condition **A**
- 10 different arrays randomly labeled as *controls* within the condition **A**
- 10 different arrays randomly labeled as *cases* within the condition **B**
- 10 different arrays randomly labeled as *controls* within the condition **B**

Two t-test statistics were computed for each data set, one comparing cases to controls within condition A and the second one comparing cases to controls within condition B. Hence, we simulated 500 datasets of bidimensional ranking statistics.

Since no consistent biological differences are expected in the simulated data sets, no gene is expected to be differentially expressed and no gene module is expected to be enriched in any of the comparisons. However, as the data come from real biological samples, the true correlation structure is kept in all 500 datasets and hence should be dragged to the computed t-statistics.

Real GO Biological Process annotation of the genes in the data set was collected form [Ensembl](http://www.ensembl.org/index.html). and filtered by size (as represented within the 10866 transcripts available in the data) 1870 Biological Process of sizes between 10 and 500 where kept for this study. Internal correlation of each GO Biological Process term was estimated by the median correlation between all pairs of genes within the GO term.

All 1870 GO terms where tested over the 500 simulated ranking statistics using our bivariate logistic model. Then, for each GO term p-values of the interaction were summarized using the median values across the 500 simulations. Therefore we obtained a median p-value estimate for each Biological Process.

Median p-value of the interaction term is plotted against median internal correlation for each of the 1870 GO terms in the figure below.


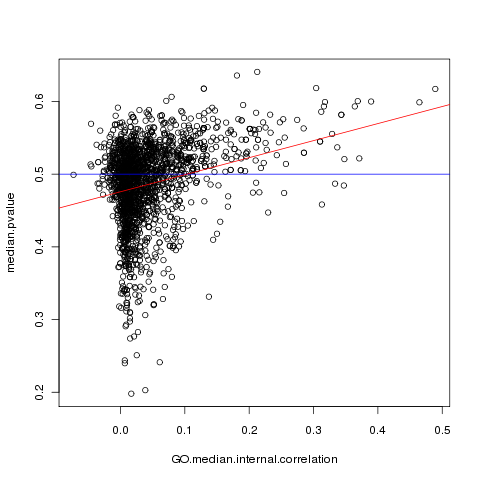


It can be appreciated in the graph that first, the already described bias of p-values being smaller than it would be expected in a random experiment with no enriched modules. Second, and not so much expected, that the bias in p-values decreases as the internal correlation of the GO terms increases. This pattern is just the opposite to what it was previously described for univariant models [2] and it is most probably due to the fact that, in general, the interaction term is a correction of the estimated main effects. In general higher p-values in the interaction term would correspond to lower p-values of the main effects an those will be associated to higher internal correlation of the GO term, as previously described by Goeman [2].

1. Montaner D, Minguez P, Al-Shahrour F, Dopazo J: **Gene set internal coherence in the context of functional profiling**. *BMC Genomics* 2009, **10**(1):197.

2. Goeman JJ, Buhlmann P: **Analyzing gene expression data in terms of gene sets: methodological issues**. *Bioinformatics* 2007, **23**(8):980-987.
